# Whole genome sequencing in the Middle Eastern Qatari population identifies genetic associations with 45 clinically relevant traits

**DOI:** 10.1038/s41467-021-21381-3

**Published:** 2021-02-23

**Authors:** Gaurav Thareja, Yasser Al-Sarraj, Aziz Belkadi, Maryam Almotawa, The Qatar Genome Program Research (QGPR) Consortium, Said Ismail, Wadha Al-Muftah, Radja Badji, Hamdi Mbarek, Dima Darwish, Tasnim Fadl, Heba Yasin, Maryem Ennaifar, Rania Abdellatif, Fatima Alkuwari, Muhammad Alvi, Yasser Al-Sarraj, Chadi Saad, Asmaa Althani, Eleni Fethnou, Fatima Qafoud, Eiman Alkhayat, Nahla Afifi, Sara Tomei, Wei Liu, Stephan Lorenz, Najeeb Syed, Hakeem Almabrazi, Fazulur Rehaman Vempalli, Ramzi Temanni, Tariq Abu Saqri, Mohammedhusen Khatib, Mehshad Hamza, Tariq Abu Zaid, Ahmed El Khouly, Tushar Pathare, Shafeeq Poolat, Rashid Al-Ali, Omar Albagha, Souhaila Al-Khodor, Mashael Alshafai, Ramin Badii, Lotfi Chouchane, Xavier Estivill, Khalid Fakhro, Younes Mokrab, Jithesh Puthen, Zohreh Tatari, Karsten Suhre, Omar M. E. Albagha

**Affiliations:** 1 Bioinformatics Core, Weill Cornell Medicine-Qatar, Education City, Doha, Qatar; 2 College of Health and Life Sciences, Hamad Bin Khalifa University, Education City, Doha, Qatar; 3 Qatar Biomedical Research Institute (QBRI), Hamad Bin Khalifa University, Doha, Qatar; 4 Department of Biophysics and Physiology, Weill Cornell Medicine, New York, NY USA; 5 Centre for Genomic and Experimental Medicine, Institute of Genetics and Molecular Medicine, University of Edinburgh, Edinburgh, UK; 6 Qatar Genome Program, Qatar Foundation Research Development and Innovation, Qatar Foundation, Doha, Qatar; 7 Qatar Biobank for Medical Research, Qatar Foundation, Doha, Qatar; 8 Sidra Medicine, Integrated Genomics Services, Out-Patient Clinic, Doha, Qatar; 9 Sidra Medicine, Applied Bioinformatics Core—Integrated Genomics Services - Research Branch, Doha, Qatar; 10 Sidra Medicine, Biomedical Informatics—Research Branch, Doha, Qatar; 11 Sidra Medicine, Maternal and Child Health Program, Doha, Qatar; 12 College of Health Sciences, Qatar University, Doha, Qatar; 13 Molecular Genetics Laboratory, Hamad Medical Corporation, Doha, Qatar; 14 Departments of Genetic Medicine, Microbiology and Immunology, Weill Cornell Medicine-Qatar, Doha, Qatar; 15 Sidra Medicine, Quantitative Genomics Laboratories, Doha, Qatar; 16 Sidra Medicine, Human Genetics Department, Doha, Qatar; 17 Sidra Medicine, Computational Genomics and Data Science Laboratory, Doha, Qatar; 18 Sidra Medicine, Clinical Research Center, Doha, Qatar

**Keywords:** Genome-wide association studies, Genetics research

## Abstract

Clinical laboratory tests play a pivotal role in medical decision making, but little is known about their genetic variability between populations. We report a genome-wide association study with 45 clinically relevant traits from the population of Qatar using a whole genome sequencing approach in a discovery set of 6218 individuals and replication in 7768 subjects. Trait heritability is more similar between Qatari and European populations (*r* = 0.81) than with Africans (*r* = 0.44). We identify 281 distinct variant-trait-associations at genome wide significance that replicate known associations. Allele frequencies for replicated loci show higher correlations with European (*r* = 0.94) than with African (*r* = 0.85) or Japanese (*r* = 0.80) populations. We find differences in linkage disequilibrium patterns and in effect sizes of the replicated loci compared to previous reports. We also report 17 novel and Qatari-predominate signals providing insights into the biological pathways regulating these traits. We observe that European-derived polygenic scores (PGS) have reduced predictive performance in the Qatari population which could have implications for the translation of PGS between populations and their future application in precision medicine.

## Abstract (web summary)

The genetic basis for traits can vary between populations. Here the authors report a genome wide association study with 45 clinically-relevant traits in individuals from Qatar, replicating many known loci and identifying new Qatari-predominant signals.

## Introduction

Genome-wide association studies (GWAS) have provided new insights into the genetic determinants of many clinically relevant traits and identified thousands of disease- or trait-associated genetic variants^1,2^. However, most of the published GWAS studies performed to-date are from European, or East Asian populations^3,4^. Middle Eastern populations are under-represented. Also, all GWAS conducted so far used genotyping arrays imputed on genome sequencing data from studies in which only few, if any, Middle Eastern genomes were present and therefore miss all population-specific signals. Large-scale GWAS of many traits and complex diseases in Africans and Asians indicated differences in the genetic architecture between populations, but included few, if any, study participants from Arab ethnicities. In addition, many trait-associated variants show differences in allele frequencies and effect sizes across populations^5–7^ which may complicate the derivation of polygenic scores. Recent studies have shown that polygenic risk scores derived from studies in European populations have lower predictive performance when applied to non-European populations^8^, providing strong argument for conducting GWAS in non-European populations that are less represented in previously published studies.

Here, we report the first comprehensive GWAS of 45 clinically relevant traits in a Middle Eastern population using a whole genome sequencing approach. We unveil differences in heritability of certain life-style related traits between populations, investigate differences in the genetic architecture of replicating loci, assess the performance of European-derived polygenic scores in Qatari population, and report novel trait associations that are predominant to the Middle Eastern population of Qatar.

## Results

### The qatar genome program (QGP)

The QGP is a population-based study designed to perform whole genome sequencing of the Qatar Biobank (QBB) participants^9^ with the aim to gain insights into the population structure and the genetic architecture of clinically relevant phenotypes in the Middle Eastern Qatari population. The present study is based on whole genome sequence data from 6218 participants of QBB and further replication in 7768 subjects from the second batch of QBB data. We performed a comprehensive heritability and genome-wide association study for 45 clinically relevant traits in the Middle Eastern population of Qatar. The investigated traits cover the following categories (Table 1): anthropometry (*N* = 3), electrolytes (*N* = 7), measures of enzyme activity or abundance (*N* = 5), blood coagulation-related traits (*N* = 4), blood cell composition (*N* = 9), lipid traits (*N* = 4), and other clinically-relevant biochemistry measurements (*N* = 13). A detailed description of the study population and phenotype assessment is provided in the methods section, Supplementary Data 1 and Supplementary Table 1. A pairwise correlation analysis of the analyzed traits (Supplementary Fig. 1) revealed correlations between related traits, such as the liver-derived enzymes ALT, AST, ALP, and GGT (abbreviations are given in Table 1), traits related to hemoglobin and red blood cells (Hb, Ht, MCV, MCH, MCHC, and Frtn), and traits related to iron metabolism, such as Fe, TIBC, Hb, and Ht.

**Table 1:** Summary of clinically-relevant quantitative traits investigated in this study.

Trait	Abbreviation	*n*	*h*^*2*^	GWS loci	
				Known	New
Anthropometric					
Sitting height	S-Height	6034	0.52	1	0
Height	Height	6044	0.59	1	0
Body mass index	BMI	6039	0.31	2	0
Electrolytes					
Calcium	Ca	6020	0.22	1	0
Chloride	Cl	6018	0.21	0	0
Iron	Fe	6010	0.13	1	0
Magnesium	Mg	5996	0.30	3	1
Potassium	K	6017	0.19	0	0
Sodium	Na	6018	0.16	0	0
Bicarbonate	HCO3	6017	0.24	0	0
Enzymes					
Alkaline phosphatase	ALP	6012	0.41	7	0
Alanine aminotransferase	ALT	6018	0.23	1	0
Aspartate aminotransferase	AST	6018	0.24	0	0
Creatine kinase	CK	5344	0.35	4	0
Gamma glutamyl transferase	GGT	4650	0.35	4	0
Coagulation					
Activated partial thromboplastin time	APTT	5988	0.43	24	0
Prothrombin time	PT	5989	0.48	23	1
International normalization ratio	INR	5987	0.35	17	1
Fibrinogen	Fbg	5984	0.35	4	0
Blood Cells					
White blood cell count	WBC	6007	0.48	42	5
Red blood cell count	RBC	6007	0.47	5	0
Mean corpuscular hemoglobin concentration	MCHC	6007	0.44	4	0
Mean corpuscular hemoglobin	MCH	6007	0.50	13	0
Mean corpuscular volume	MCV	6007	0.51	10	0
Platelet count	Plt	5935	0.46	4	0
Mean platelet volume	MPV	6006	0.57	11	0
Hematocrit	Ht	6007	0.21	0	0
Hemoglobin	Hb	6006	0.23	0	0
Lipids					
Total cholesterol	TCH	6017	0.22	6	0
High density lipoprotein cholesterol	HDL-C	6013	0.41	6	0
Low density lipoprotein cholesterol	LDL-C	5972	0.21	6	0
Triglycerides	TG	6017	0.31	8	1
Other biochemical					
Total protein	TPrt	6017	0.32	0	0
Albumin	Alb	6018	0.27	0	0
Ferritin	Frtn	5947	0.16	0	0
C-peptide	C-Pep	5925	0.19	0	0
Pro B-type natriuretic peptide	proBNP	5516	0.25	4	0
Total iron binding capacity	TIBC	6010	0.31	17	0
Unsaturated iron binding capacity	UIBC	5956	0.23	9	0
Total bilirubin	Tbil	6018	0.39	21	3
Uric acid	UA	6013	0.31	6	1
Folate	Flt	5944	0.33	3	0
Serum creatinine	sCr	6018	0.32	1	0
Homocysteine	Hcyst	5727	0.30	5	3
Vitamin B12	Vit-B12	5865	0.29	7	1

*n* sample size, *h*^*2*^ heritability, *GWS* genome-wide significant.

### Heritability of clinically relevant traits in the Qatari population

**Fig. 1:**
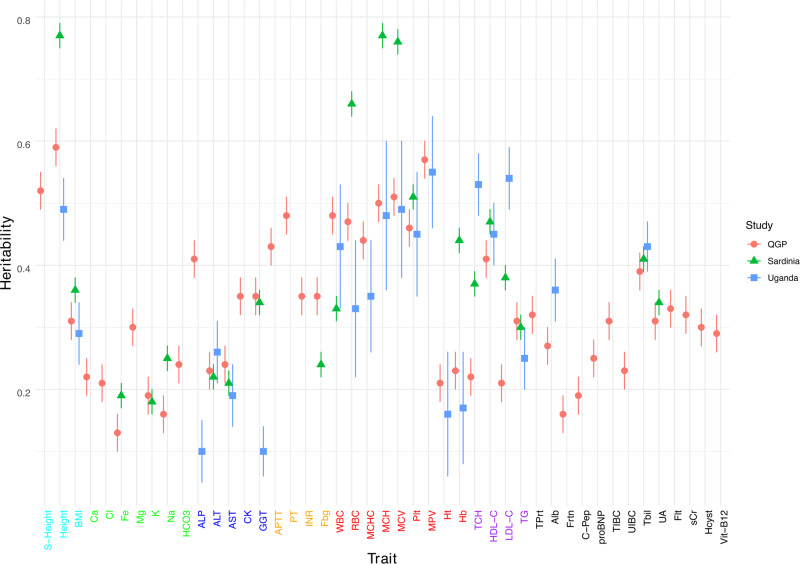
Heritability estimates of 45 clinically-relevant traits. Heritability estimates of 45 clinically-relevant traits in the Qatar Genome Program cohort (QGP; red markers) compared to estimates from Sardinian (green marker) and Ugandan (blue markers) populations. The heritability estimates in QGP was adjusted for age, gender, the first four population principal components and relatedness. The heritability estimates for several traits in QGP were significantly different from European^10^ and African^6^ Populations (Supplementary Table 2). Refer to Table 1 for trait abbreviations. Data are presented as mean ± SEM.

The proportion of variation that can be attributed to genetic factors (heritability) has been investigated for many clinically-relevant traits, but mainly in populations of European descent^10,11^. A recent study in the Ugandan population of Africa showed marked differences in heritability estimates for many complex traits compared to European populations^6^. For example, estimates of heritability for body height in Ugandan populations was significantly lower (49%) than those from European populations (77%) suggesting differences in genetic loci and/or proportion of environmental contribution. The heritability of most traits in Middle Eastern populations remains undetermined. We therefore performed a comprehensive assessment of the heritability (*h*^*2*^) of the 45 traits in the QGP data. We found that *h*^*2*^ estimates ranged from 13% for serum iron levels (Fe) to 59% for body height (Table 1). We compared our findings with heritability estimates from European^10,11^ and African populations^6^. Overall, the correlation of *h*^*2*^ between European and Middle Eastern populations was higher (*r* = 0.81) compared to the correlation between African and Middle Eastern populations (*r* = 0.44; Supplementary Fig. 2). For several traits, *h*^*2*^ estimates in the Middle Eastern population were significantly different from European and African populations (Fig. 1, Supplementary Tables 2 and 3). For example, estimates of *h*^*2*^ for height in QGP (59%) was lower than in European (77%; *P* = 6.0 × 10^−7^) but not significantly different from African populations (49%, *P* = 0.09). Similarly, heritability for BMI in QGP (31%) was lower than in Europeans; namely Sardinia (43%; *P* = 8.8 × 10^−4^) and Iceland (42%; *P* = 2.3 × 10^−3^). Interestingly, *h*^*2*^ for the liver enzyme GGT in QGP data (35%) was similar to the European populations (34%; *P* = 0.78) but significantly higher than in African populations (10%, *P* = 5.8 × 10^−7^). In contrast, estimates of heritability for cholesterol in QGP data (TCH = 22%; LDL-C = 21%) was significantly lower than values from European (TCH = 37%; *P* = 3.2 × 10^−5^, LDL-C = 38%; *P* = 2.4 × 10^−6^) or African (TCH = 53%; *P* = 1.1 × 10^−7^, LDL-C = 54%; *P* = 1.6 × 10^−8^) populations.

### Genome-wide association analysis of 45 complex traits

We performed genome-wide association analyses of 45 clinically-relevant quantitative traits using whole genome sequencing data for 6218 individuals from the QGP study. We focused on common and low frequency variants (MAF > 1%; *N* = 7,880,618) using linear mixed models correcting for age, sex, population principal components and relatedness (see methods). The genomic inflation factor (λ_GC_) ranged between 0.99 and 1.13 (mean ± S.D; 1.03 ± 0.03; Supplementary Table 4). Most analyzed traits (37 out of the 45 traits) showed very little inflation (λ_GC_ ≤ 1.04). Considerable inflation was only detected for traits that are well-known to have large polygenic architecture such as adult height (λ_GC_ = 1.13) and BMI (λ_GC_ = 1.09). Manhattan and quantile-quantile plots for the studied traits are presented in Supplementary Data 2.

**Fig. 2:**
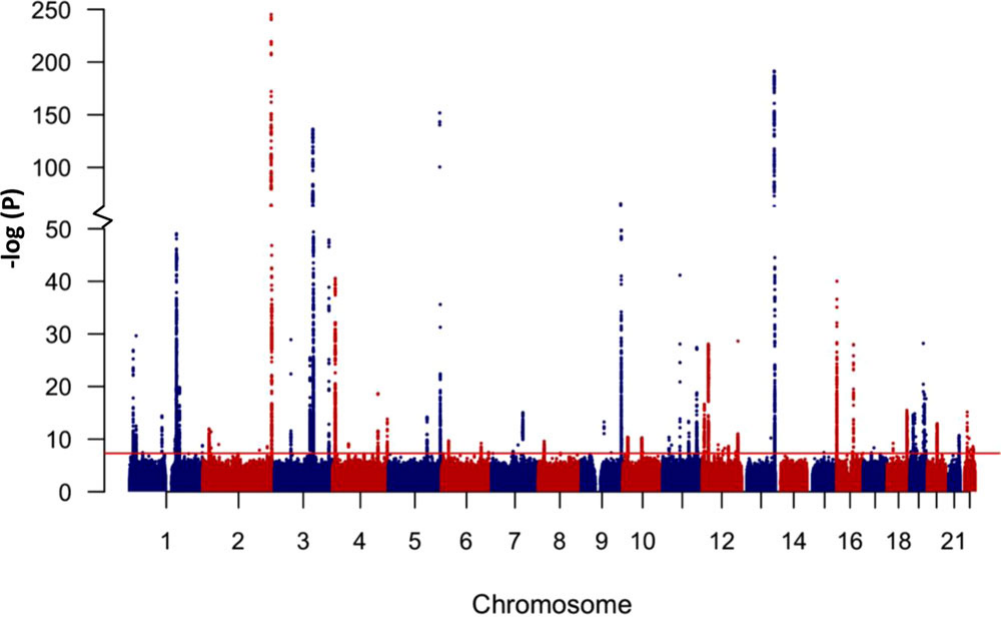
Manhattan plot of GWAS results from 45 clinically-relevant traits. The chromosomal position of genetic variants (*N* = 7,880,618) is plotted against –log_10_ (P). Analysis was performed using linear mixed models correcting for age, sex, population principal components and relatedness. The red horizontal line represents the threshold for genome-wide significance (*P* < 5.0 × 10^−8^).

**Fig. 3:**
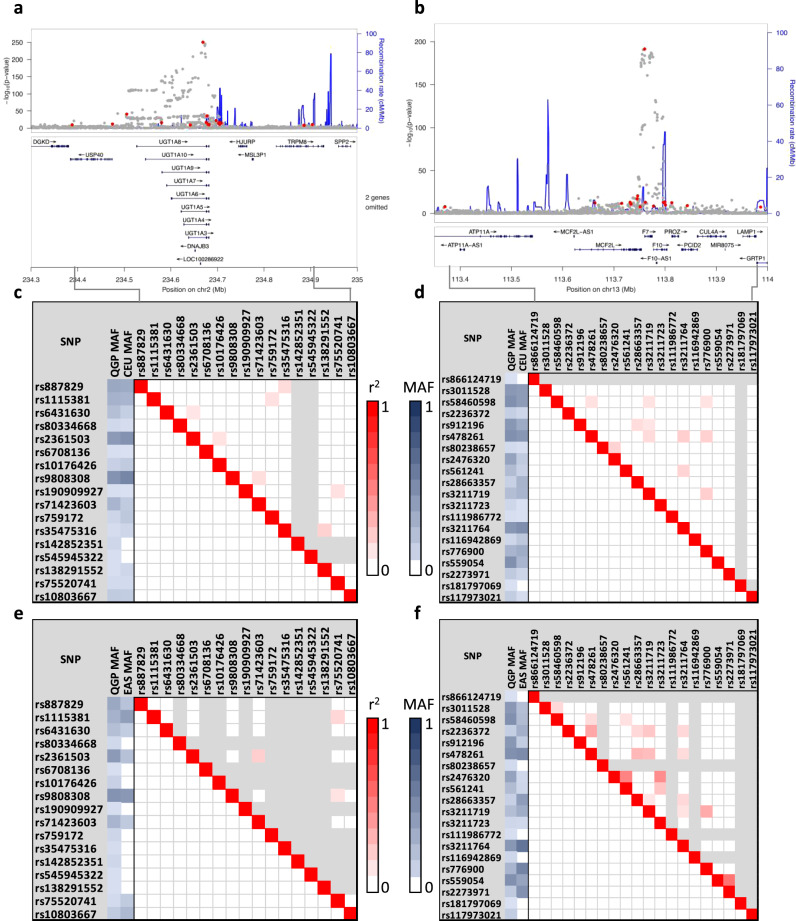
Comparison of allele frequency and linkage disequilibrium patterns. Example of multiple distinct signals identified at loci associated with clinically-relevant traits. **a** Regional association plots for total bilirubin (Tbil) locus on chromosome 2 and (**b**) the prothrombin time (PT) locus on chromosome 13. The plots show chromosomal positions of SNPs plotted against –log_10_ (P). Multiple distinct signals are shown as red circles, blue lines represent recombination rate. **c–f** Comparison of allele frequency and linkage disequilibrium for the distinct signals between QGP and European population (**c** and **d**) or between QGP and East Asian populations (**e** and **f**) for the Tbil and PT loci. Linkage disequilibrium patterns between the distinct signals from QGP data are shown below the red diagonal boxes and those from European (**c**, **d**) or East Asian (**e**, **f**) populations are shown above the diagonal red boxes. Grayed areas indicate monomorphic SNPs. MAF indicates minor allele frequency from QGP, European (CEU) or East Asian (EAS) populations.

Figure 2 shows a Manhattan plot comprising association data for all 45 studied traits. We identified 301 distinct variant-trait-associations that reached a genome wide significance level of *P* < 5.0 × 10^−8^ (Table 1 and Supplementary Data 3). For each trait, a distinct signal was defined as the variant with the lowest *P* value and not in linkage disequilibrium (LD; *r*^2^ < 0.1) with any other variant within a window of 10 Mb. Of the 301 identified genetic signals, 281 were located within ±500 kb of a previously reported variant for the same trait. We replicated many loci that are known to have consistent association in studies across various population ancestry^2^. Examples include the *SLC2A9* locus for uric acid (rs13129697; *P* = 2.8 × 10^−41^), the *UGT1A4* locus for total bilirubin (Tbil; rs887829; *P* = 3.5 × 10^−251^) and the *APOE* locus for low density lipoprotein-cholesterol (LDL-C; rs7412; *P* = 6.3 × 10^−29^). Of the 281 genome-wide significant variant-trait associations, 51 were observed for the same SNP as reported in the PhenoScanner^12^. For these SNPs it was possible to assess the direction of association (for variants with available effect-allele; *N* = 43) and all showed directionality of association consistent with previous reports. We also observed multiple distinct signals for many loci (Supplementary Data 3). For example, 17 distinct genome-wide significant signals were observed for total bilirubin (Tbil) in a 515 kb region on chromosome 2 (Fig. 3a). Notably, this Tbil locus harbors the complex *UGT1A* gene locus that encodes nine enzymes which differ in their N-termini as a result of splicing nine unique substrate-recognizing first exons into four shared exons. These enzymes are involved in transforming lipophilic substrates, such as bilirubin, into water soluble metabolites. Another example was prothrombin time (PT) for which 20 distinct genome-wide significant signals were detected in a 615 kb region on chromosome 13 (Fig. 3b) which harbors two coagulation factor genes: *F7* and *F10*. We investigated whether differences in linkage disequilibrium (LD) patterns between QGP data and other populations can account for differences in signal patterns. LD analysis of the Tbil and PT loci shows marked differences in LD patterns and allele frequencies between the European, East Asian and QGP populations (Fig. 3). For example, nine of the 17 distinct genome-wide significant signals from the Tbil locus are either monomorphic (*N* = 6) or very rare (MAF < 0.3%; *N* = 3) in the East Asian population whereas only two of the variants are very rare in Europeans.

To assess to which degree we replicate the effect sizes of known signals in QGP, we compared our results to previously published work focusing on a single large comprehensive GWAS of similar traits from the Biobank Japan project (BBJ)^7^ as a reference. We selected this study because it represents the largest and most comprehensive published GWAS of similar traits, and because the traits in the BBJ study were transformed similarly to our analysis, which allows a direct comparison of variant-level effect sizes for the identified loci (Z-Score or rank-based inverse normal transformation). In the BBJ study, Kanai et al.^7^ performed a GWAS of 58 clinically-relevant traits in study participants of East Asian descent. Of the 45 traits analyzed in the present study, 28 traits overlapped with those analyzed by the BBJ project. For these traits, the BBJ study identified a total of 907 trait-variant associations which include known loci from previous studies in various populations (*N* = 575; mainly Europeans) as well as new loci identified in the Japanese population (*N* = 332). Of the 907 association, we could evaluate 898 in QGP: 659 for which the same genetic variant was available in our data set (designated as group A variants) and 239 for which at least one proxy variant within 1 Mb in our data was in strong LD (*r*^2^ ≥ 0.8; *N* = 149; designated as group B variants) or exhibited some degree of LD (*r*^2^ = 0.1 to 0.8; *N* = 90; designated as group C variants) with the variant reported in BBJ (Table 2 and Supplementary Data 4). For 9 variants, no suitable proxy was found in our dataset.

**Table 2:** Summary of replicated loci compared to Biobank Japan data^7^.

Category^a^	No. of replicated loci		Signal within 500 kb^b^	Total replicated	Total loci
	*P* < 0.05	*P* < 5.6 × 10^−5^			
Group A	180	29	87	267	659
Group B	45	11	16	61	149
Group C	15	2	12	27	90
Total	240	42	115	355	898

^a^Group A refers to same variants as reported by Kanai et al^7^, Group B and C refer to proxy variants in strong LD (*r*^2^ ≥ 0.8) or shows some degree of LD (*r*^2^ = 0.1 to 0.8) with variants reported by Biobank Japan project^7^, respectively.

^b^Number of loci that did not show replication at *P*  <  0.05 but a significant signal was found within 500  kb for the same trait. *P* values obtained from GWAS analysis of QGP data using linear mixed models adjusting for age, sex, population principal components and relatedness.

**Fig. 4:**
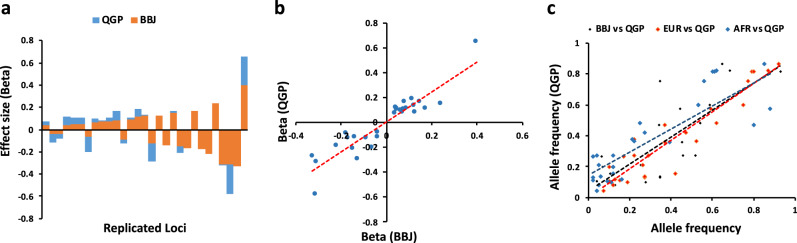
Comparison of allele frequency and effect size for known loci. **a** The effect size (Beta) for loci showing replication after correction for multiple testing in QGP (blue bars) compared to Biobank Japan project (BBJ^7^, orange bars). **b** Correlation of effect size for replicated loci between QGP and BBJ. **c** Correlation of allele frequency for replicated loci between QGP and BBJ (*r* = 0.80), QGP and European (EUR; *r* = 0.94), or QGP and African (AFR; *r* = 0.85) populations. Dotted lines represent lines of best fit from regression analysis.

The genetic architecture for many traits can vary between populations. Differences in allele frequency and /or effect size for many trait-associated variants are known to exist between populations^5^. Since polygenic risk scores estimated from one population may not be precisely applicable to other populations, we assessed replication, allele frequency and effect size for group A variants in QGP data. We found 29 loci that replicated at a Bonferroni-corrected significance threshold of *P* < 5.6 × 10^−5^ (0.05/898). All had consistent direction of effect. Comparison of effect size for the replicated loci showed a significant trend for higher effect sizes in QGP compared to BBJ (regression slope = 1.21; 95% CI = 1.01–1.42; *P* = 3.2 × 10^−12^; Fig. 4). Of the 29 replicated loci, 17 showed an effect size that is 20% larger in our data compared to BBJ. Comparison of allele frequencies for replicated loci shows higher correlation with European (*r* = 0.94) compared to African (*r* = 0.85) or Japanese (*r* = 0.80) populations (Fig. 4). Further analysis using colocalization testing (see methods) showed that out of the 29 replicated loci, 22 share the same association signal, whereas 7 had distinct signals between QGP and BBJ; highlighting differences in LD patterns between the two populations (Supplementary Data 5).

When using a nominal significance threshold (*P* < 0.05), 180 of the 659 group A variants show nominal evidence of replication and all but seven of these have consistent direction of effect with the variant reported in BBJ. Analysis of these loci revealed significant differences in the distribution of effect size compared to BBJ data (Supplementary Fig. 3). We found a significantly larger number of loci with an effect size (Beta) between 0.05 – 0.1 in our data (*N* = 80) compared to BBJ (*N* = 51; *P* = 6 × 10^−4^). Conversely, a larger number of loci with small effects (Beta < 0.05) was observed in BBJ (*n* = 97) compared to our data (*n* = 60; *P* = 2.7 × 10^−5^) but no significant difference was found for loci with an effect size > 0.1 (*P* > 0.05). Comparison of allele frequencies for these loci also shows higher correlation with European (*r* = 0.94) compared to African (*r* = 0.75) or Japanese population (*r* = 0.70). Colocalization analysis showed that 16 out of the 180 loci (8.9%) had distinct signals between QGP and BBJ (Supplementary Data 5).

A number of group B (11 out of 149) and group C variants (2 out of 90) showed evidence of replication for the same trait after correction for multiple testing (Table 2). In addition, a number of loci that did not show nominal replication (*P* > 0.05) contained a signal within ±500 kb with significant P-values for the same trait (*N* = 115), after correction for multiple testing (Table 2). For example, rs5030081 is associated with APTT in BBJ (*P* = 3.97 × 10^−49^) but not in QGP (*P* = 0.21), however, a SNP (rs1042445) located 63 kb upstream and not in LD with rs5030081 (*r*^2^ = 0.05) is significantly associated (*P* = 1.30 × 10^−48^) with the same trait in QGP. In total, of the 898 variants identified in the BBJ study, we identified 355 variants that showed evidence of replication either directly, through a proxy, or located in a region previously reported for the same trait.

### Analysis of polygenic scores

**Fig. 5:**
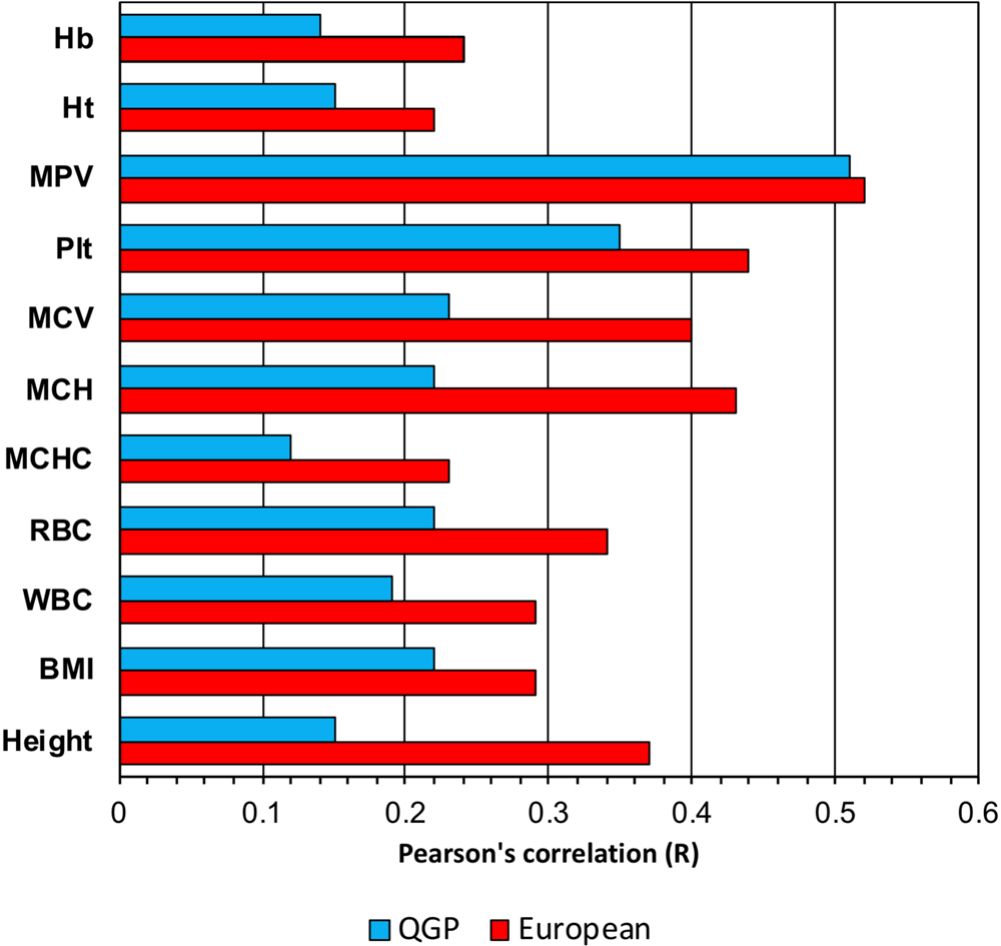
Performance of European-derived polygenic scores in QGP. Pearson’s correlation (R) between polygenic scores (PGS) and trait values are shown for European populations (red) and Qatari population (blue). Weighted PGS scores were based on those derived from European population.

To assess the translatability of polygenic scores (PGS) derived from other populations to the Qatari population, we assessed the predictive performance of PGS for traits with available scoring data in the Polygenic Score Catalog (http://www.pgscatalog.org). We focused our analysis on PGS derived from European populations since our heritability and allele frequency comparison showed higher correlation between QGP and Europeans. In addition, robust data with enough information to allow comparison with our data was mainly available for European populations. The predictive performance of PGS from 11 traits was tested on QGP data and results are presented in Fig. 5. All tested PGS showed lower performance when applied to QGP data with an average performance of 64.7% (SD = 15.8%) of that when applied to Europeans (Supplementary Table 5). The relative performance of the PGS when applied to Qataris compared to Europeans ranged from 40.5% for Height to 98.1% for Mean Platelet Volume.

### Novel loci associated with traits

Variants located in regions not previously reported for the trait and showing significant association (*P* < 5.0 × 10^−8^; *N* = 20) in the discovery set were tested for replication in an additional 7768 subjects from the second data release of QBB. Eight of the 20 novel loci replicated at a Bonferroni level (*P* < 0.05/20). Meta-analysis of discovery and replication results are shown in Table 3. Most signals were driven by genetic variants that were either monomorphic (*N* = 3) or with a minor allele frequency that is three- to seven-fold lower (*N* = 5) than what is observed in European population ancestries^13^. These include novel loci for coagulation-related traits (PT, and INR), blood cell traits (WBC) and other biochemical traits (Hcyst, and Tbil). Regional association plots (RAP) for novel loci are shown in Supplementary Data 6. For example, we identified a novel association with Homocysteine (Hcyst) on chromosome 21 (rs147242481) near the *CSTB* gene with a relatively large effect size (beta = 0.36 standard deviation units per allele; *P* = 1.0 × 10^−13^). *CSTB* is a member of the cystatin superfamily that encodes the Cystatin B protein that functions as protease inhibitor. It has been shown that Cystatin C, another member of cystatin superfamily, is a determinant of serum levels of Hcyst^14,15^. We also identified a novel locus associated with two related coagulation traits (PT and INR) on chromosome 13 situated near *LINC01070*, a gene that encodes a long noncoding RNA. Another novel association was identified with serum total bilirubin (Tbil) near *ARL4C* gene. Serum level of Tbil is routinely used to assess liver function and studies have shown that *ARL4C* is highly expressed in primary hepatocellular carcinoma tumors and its expression is associated with poor prognosis^16^. We also identified four novel loci for white blood cell count trait (Table 3). One of these loci is located near *NHLH1* and data from the international mouse phenotyping consortium (https://www.mousephenotype.org) shows that heterozygous *Nhlh1* knockout mice have decreased basophil white blood cell number compared to wild type (*P* = 3.1 × 10^−17^; Supplementary Fig. 4).

**Table 3:** Novel and Qatari-predominant association signals discovered form GWAS meta-analysis of QGP.

Trait^a^	Variant ID	Chr	Position	A1	A2	MAF (%)	GWAS		Replication		Meta-analysis			Nearest gene
							Beta (se)^b^	*P* value	Beta (se)^b^	*P* value	Beta (se)^b^	*P* value	P-het	
Novel associations with traits														
Hcyst	rs147242481	21	45192107	G	A	1.4	0.52 (0.08)	2.0 × 10^−11^	0.26 (0.06)	3.0 × 10^−5^	0.36 (0.07)	1.0 × 10^−13^	0.01	*CSTB*
INR	rs865897814	13	112900219	T	C	1.2	0.55 (0.08)	1.7 × 10^−11^	0.41 (0.08)	8.2 × 10^−8^	0.47 (0.08)	1.4 × 10^−17^	0.22	*LINC01070*
PT	rs865897814	13	112900219	T	C	1.2	0.58 (0.08)	6.4 × 10^−13^	0.45 (0.07)	8.3 × 10^−11^	0.51 (0.08)	7.7 × 10^−22^	0.24	*LINC01070*
Tbil	rs2204511	2	235503327	A	G	23.5	−0.12 (0.02)	1.3 × 10^−8^	−0.05 (0.02)	2.4 × 10^−3^	−0.08 (0.02)	2.0 × 10^−9^	0.02	*ARL4C*
WBC	rs12080243	1	160347420	C	T	13.5	−0.15 (0.03)	3.6 × 10^−8^	−0.15 (0.02)	1.8 × 10^−11^	−0.15 (0.03)	3.5 × 10^−18^	0.93	*NHLH1*
WBC	rs11809289	1	158082328	A	G	13	−0.17 (0.03)	6.9 × 10^−10^	−0.13 (0.02)	5.6 × 10^−9^	−0.15 (0.03)	5.3 × 10^−17^	0.30	*KIRREL*
WBC	rs76537384	1	158055475	T	C	2.4	−0.34 (0.06)	3.2 × 10^−8^	−0.23 (0.05)	5.7 × 10^−6^	−0.27 (0.06)	2.6 × 10^−12^	0.15	*KIRREL*
WBC	rs6677720	1	156835588	T	C	6.6	−0.22 (0.04)	5.3 × 10^−9^	−0.10 (0.03)	7.4 × 10^−4^	−0.15 (0.03)	3.4 × 10^−10^	0.01	*NTRK1*
Novel Qatari-predominant associations at known loci														
Hcyst	rs187169250	11	89192588	C	T	6.77	−0.21 (0.04)	2.8 × 10^−9^	−0.21 (0.03)	1.4 × 10^−11^	−0.21 (0.03)	1.7 × 10^−19^	0.94	*NOX4*
Hcyst	rs71469261	11	89138485	C	T	5.93	−0.22 (0.04)	1.5 × 10^−8^	−0.27 (0.03)	1.1 × 10^−16^	−0.25 (0.04)	1.3 × 10^−23^	0.27	*NOX4*
Mg	rs189260309	9	77439134	T	G	5.85	0.17 (0.04)	1.1 × 10^−5^	0.22 (0.03)	1.1 × 10^−11^	0.20 (0.04)	6.6 × 10^−16^	0.39	*TRPM6*
Tbil	rs182021046	2	234177849	C	G	5.73	−0.17 (0.04)	6.1 × 10^−6^	−0.18 (0.03)	9.0 × 10^−8^	−0.17 (0.04)	2.4 × 10^−12^	0.94	*ATG16L1*
Tbil	rs183884248	12	21085954	C	T	5.04	0.21 (0.04)	1.2 × 10^−7^	0.29 (0.04)	2.5 × 10^−15^	0.25 (0.04)	4.6 × 10^−21^	0.16	*SLCO1B3*
TG	rs376997679	11	115393226	G	A	5.92	0.16 (0.04)	7.3 × 10^−6^	0.14 (0.03)	2.6 × 10^−6^	0.15 (0.03)	1.1 × 10^−10^	0.67	*CADM1*
UA	rs143909619	4	10084946	G	A	5.27	0.19 (0.04)	1.1 × 10^−7^	0.22 (0.03)	1.5 × 10^−14^	0.21 (0.03)	1.1 × 10^−20^	0.42	*WDR1*
Vit-B12	rs143522487	11	59684469	C	T	5.28	−0.31 (0.04)	1.4 × 10^−14^	−0.30 (0.03)	8.2 × 10^−19^	−0.30 (0.04)	5.8 × 10^−32^	0.81	*OOSP1*
WBC	rs143969748	1	159529347	G	A	6.26	−0.35 (0.04)	1.4 × 10^−19^	−0.41 (0.03)	6.9 × 10^−38^	−0.38 (0.03)	5.0 × 10^−56^	0.21	*OR10J5*

^a^See Table 1 for trait abbreviation.

^b^Effect size (beta) for allele A2; se; standard error; MAF; Minor allele (A2) frequency. P-het, *p* value for Cochran’s Q heterogeneity statistic. *P* values obtained from GWAS analysis of QGP data using linear mixed models adjusting for age, sex, population principal components and relatedness.

### Qatari- predominant loci associated with traits

Population-specific signals have been identified for clinically relevant traits in previous GWAS^6,7^ but the existence of such signals in the Middle Eastern populations has not been studied. We identified 12,283 autosomal variants that are common (MAF > 5%) in QGP but rare (MAF < 1%) in all other population ancestries reported by 1000 Genome project^13^. These variants were pruned based on LD (*r*^2^ < 0.1; *N* = 4357; referred to as Qatari-predominant loci) and their association with the clinical traits was investigated. For these loci, we used Bonferroni-adjusted significance threshold of *P* < 1.15 × 10^−5^ correcting for the number of tested variants. Loci showing significant association in the discovery set were tested in the replication set to confirm their association. Meta-analysis of discovery and replication results identified 9 Qatari-predominantvariant-trait-associations (Table 3). All of these signals were located near genes that had been previously associated with the same trait.

## Discussion

We performed one of the largest GWAS using whole genome sequence data to date (*n* > 6200) and the first comprehensive GWAS of 45 clinically-relevant traits from a Middle Eastern population. Heritability estimates for the studied traits in QGP were generally correlated with previous estimates in other populations. However, significant differences in heritability were observed for some traits suggesting differences in the genetic architecture resulting from population-specific past events such as genetic drift and selection. In addition, variations in environmental factors and their interaction with genetic factors could explain the observed differences in heritability. For example, the heritability of GGT, an enzyme used clinically to assess liver function, was similar to that reported in European populations but significantly higher than in African populations. This observation could be due to a larger contribution of environmental factors explaining the phenotypic variations in GGT in African populations. High prevalence of liver diseases such as cirrhosis and Hepatitis B virus infection in sub-Saharan African populations^17^ indicating substantial contribution of environmental factors leading to lower heritability estimates. In addition, heritability estimates for cholesterol (TCH and LDL-C) in our data were significantly lower compared to values reported in European or African populations suggesting higher contribution of environmental influences such as diet and lifestyle. Consistent with this, heritability estimates for BMI in Qatar was also lower when compared to two European populations. The prevalence of obesity in Qatar is among the highest in the world^18^ and this obesity endemic caused (at least in part) by fat-rich diet and lifestyle factors plausibly leading to lower heritability estimates of BMI and cholesterol traits. However, technical variations in measurements could also contribute to the differences in heritability between the studies.

Our GWAS results replicated many loci that are known to have consistent association in studies across various population ancestry, highlighting shared components of genetic architecture for the studied traits. Comparison of replicated loci identified differences in both effect size and allele frequency of the associated variants, emphasizing the importance of performing further larger GWAS in the Middle Eastern populations to enable accurate polygenic score determination. Indeed, European-derived PGS had substantially reduced predictive performance when applied to QGP data. For some traits, we identified multiple distinct signals due to differences in LD patterns and/or differences in allele frequencies of the variants. Colocalization analysis showed that about 9% of replicated loci across the investigated traits showed evidence of distinct signals between QGP and BBJ. Since most previously published GWAS for these traits were performed using genotyping arrays followed by imputation, it is possible that some of the multiple distinct signals observed in our data could be due to higher coverage of whole genome sequencing as opposed to imputation with implication on fine mapping and identification of functional variants.

Our GWAS also identified novel signals providing new insights into the biological pathways regulating clinically relevant traits, such as *CSTB* for Hcyst and *NHLH1* for white blood cell count. We observed that most of the novel loci were driven by population-specific variants and the existence of Qatari-predominant signals within known loci emphasizes the differences in genetic architecture of these traits between populations. These findings provide strong arguments for performing larger GWAS in the Middle Eastern region to further define the genetic architecture of clinical traits and complex diseases with implication on future application in precision medicine. They also underscore the potential of discovering novel signals at lower sample sizes when using understudied populations, which may be relevant to future investments when searching for new drug targets.

In conclusion, we performed a comprehensive heritability analyses and GWAS studies of 45 clinically-relevant traits for the first time in a middle eastern population. We replicated many previously known loci for these traits demonstrating shared genetic components across populations. However, we identified differences in linkage disequilibrium patterns, effect size and allele frequency of associated signals. We showed that European-derived PGS has reduced predictive performance when applied to the Middle Eastern population of Qatar. We also identified 17 novel and Qatari-predominant signals across the studied traits which were mostly driven by population-specific variants providing an argument for further larger genetic association studies in Middle Eastern and other non-Caucasian populations to further characterize the genetic architecture of clinical traits and complex diseases.

## Methods

### Study subjects

The present study was performed on the QBB study participants. QBB is an on-going longitudinal population-based study aiming to recruit 60,000 subjects from the Qatari population with follow up every 5 years^9^. Individuals are eligible to participate in the study if they are Qatari nationals or long-term residents (≥15 years living in Qatar) aged 18 years and older. The study covers extensive baseline sociodemographic data, clinical and behavioral phenotypic data, biological samples, as well as clinical biomarkers. All QBB participants signed an informed consent form prior to their participation; and the study was approved by Hamad Medical Corporation Ethics Committee and QBB institutional review board. Heritability and GWAS analyses were performed on data from the first QBB data release (6218 QBB participants). Replication analyses were based on an additional 7768 QBB participants from the second QBB data release.

### Phenotype

All QBB participants attended an assessment session in which physical measurements were collected and each participant filled a standardized questionnaire reporting information on lifestyle, diet, and medical history. Collected physical measurements included anthropometry (sitting and standing height, weight, and waist and hip circumference), body composition, grip strength, arterial stiffness, blood pressure, electrocardiogram data, respiratory function and cardiorespiratory fitness. Additional phenotypes were also collected, such as 3D carotid ultrasound, full body dual energy X-ray absorptiometry (iDXA), “microscopic” features of the optic nerve and macula, and brain magnetic resonance imaging (MRI). During the assessment session, participants provided biological samples (blood, saliva and urine) for analysis and storage. Part of the biological samples were transferred to the diagnostic laboratories at Hamad General Hospital where clinical diagnostic biomarkers were measured. The present study focused on 45 clinically-relevant traits as listed in Table 1, details of their measurements are presented in Supplementary Data 1. All traits were normalized prior to the statistical analyses using rank-based inverse normal transformation using R ver. 3.4.0.

### Whole genome sequencing

DNA was extracted from peripheral blood using the automated QIASymphony SP instrument according to Qiagen MIDI kit protocol’s recommendations (Qiagen, Germany). Genomic DNA integrity was assessed using the Genomic DNA assay on the Caliper Labchip GXII (Perkin Elmer, USA). DNA quantification was done using Quant-iT dsDNA Assay (Invitrogen, USA) on the FlexStaion 3 (Molecular Devices, USA). Whole genome libraries were prepared from 150 ng of DNA using the Illumina TruSeq DNA Nano kit. Genomic libraries were sequenced on HiSeq X Ten (illumina, USA) following the manufacturer’s recommended protocol to achieve a minimum average coverage of 30x. Library construction and sequencing was performed at the Sidra Clinical Genomics Laboratory Sequencing Facility. Quality control of Fastq files was performed using FastQC (v0.11.2) (https://www.bioinformatics.babraham.ac.uk/projects/fastqc/). Reads were then aligned to GRCh37 (hs37d53) reference genome using bwa.kit (v0.7.12) (https://github.com/lh3/bwa/tree/master/bwakit). Quality control on mapped reads was performed using Picard (v1.117) [CollectWgsMetrics] (https://gatk.broadinstitute.org/hc/en-us). Variant calling was performed following GATK 3.4 best practices (https://software.broadinstitute.org/gatk/documentation/article?id=3238): Indel realignment and base recalibration was performed on the initial bam file then HaplotypeCaller was run on each sample to generate an intermediate genomic variant call file (gVCF). Joint variant calling was performed using all generated gVCF files at once. We first run GenomicsDB8 to combine the different samples by regions, then for each region, we ran GenotypeGVCFs, applied SNP/Indel recalibration and then merged all regions.

The combined gVCF file contained 77,867,351 variants for 6218 subjects. Quality control measures were applied to this file using PLINK ver. 2.0^19^. Indels and variants with MAF < 1%, genotype call rate < 90%, Hardy-Weinberg*P* value < 1 × 10^−6^, and those on chromosome X were removed leaving a total of 7,880,618 variants. We also removed samples with excess heterozygosity (*N* = 8), duplicates (*N* = 10), call rate < 95% (*N* = 1), and gender ambiguity (*N* = 65). To identify population ancestry outliers, we performed multidimensional scaling (mds) analysis as implemented in PLINK^19^. Pairwise identity by-state (IBS) matrix was determined based on a pruned set of independent autosomal SNPs (*N* = 62,475) using a window size of 200 SNPS and LD threshold of *r*^2^ = 0.05. Subjects with more than four standard deviation units (±4 SD) away from the mean of the first two mds components were identified as population outliers (*N* = 87) and removed before analysis (Supplementary Fig. 5). The final file used for genome-wide association analyses comprised 7,880,618 variants and 6047 subjects. Similar quality control measures were applied to the replication dataset where we only tested SNPs and traits showing evidence of novel associations from the discovery set.

### Heritability analysis

Heritability (*h*^*2*^*)* was defined as the proportion of phenotypic variance attributed to genetic factors estimated from genome-wide SNP genotype data. *h*^*2*^ was calculated using the polygenic model implemented in GenABEL ver. 1.8-0^20^. The model included age, sex, and the first four principal components (PC) as covariates. Genomic kinship matrix was used to correct for relatedness and was determined using IBS analysis implemented in GenABEL. To enable comparison of heritability with previously published work, we also calculated heritability using the restricted maximum likelihood (GREML) method^21^ implemented in the software package GCTA^22^. Age, sex, and the first four PCs were included as covariates in the GREML model. Linear regression analysis was used to assess correlation between heritability values across population ancestries.

### Genome-wide association analysis

Genome wide association testing was performed using the variance component-based method GRAMMAR-Gamma^23^ implemented in the R package GenABEL^20^. This model uses genomic kinship matrix to correct for relatedness and genetic substructure. For all tested traits, we included age, sex, and the first four PCs as covariates in the regression model. Principal component analysis was performed using PLINK. Genome-wide significance threshold was set as (*P* < 5 × 10^−8^)^24^. Regional association plots were generated using the locusZoom tool^25^ using linkage disequilibrium data calculated from QGP data using PLINK. Genomic inflation factor, Quantile-Quantile plots and Manhattan plots were generated using R ver. 3.4.0.

### Assessing genome-wide significant loci

We lumped all associations on a given trait based on LD (*r*^2^ < 0.1) within a window size of 10 Mb into distinct signals described by the SNP with the lowest *p* value. We annotated the SNPs representing the distinct loci using PhenoScanner^12^ allowing for proxy SNPs reported in five populations (AFR, AMR, EAS, EUR, SAS) using an LD cut-off of *r*^2^ > 0.1, a window size of ±500 kb, and we used Experimental Factor Ontology (EFO) terms (https://www.ebi.ac.uk/ols/ontologies/efo) to map phenotypes (Supplementary Data 3). Any locus that did not produce a hit in PhenoScanner was further manually checked using the GWAS catalog^2^ and PubMed literature searches (accessed on 31 January 2020).

### Assessing replication of known loci

To assess the degree of replication of known signals, we compared our results to previously published work focusing on a single large GWAS of similar traits from the Biobank Japan project (BBJ)^7^. We distinguished three groups of SNPs in our comparisons to the BBJ study. Group A: an identical SNP is present in the QGP population, Group B: a strong proxy SNP is available for replication (*r*^2^ ≥ 0.8), group C: a SNP with LD (0.1 < *r*^2^ < 0.8) is available. To identify any other signal, we also queried the region ±500 kb for association with the relevant trait. Correlation of effect size and allele frequency was performed using linear regression analysis. To assess differences in associated signals between QGP and BBJ we performed colocalization analysis using the Coloc R-package ver. 3.2-1^26^. We tested two hypotheses: H3; the locus is associated with the trait in both BBJ and QGP but the association is driven by different variants, H4; the locus is associated with the trait in both BBJ and QGP and the association is driven by the same variants. Meta-analysis of discovery and replication results was performed using the inverse variance-weighted method implemented in METAL^27^.

### Analysis of polygenic scores

Polygenic scores (PGS) scoring files were downloaded from the Polygenic Score catalog (http://www.pgscatalog.org) for traits with available data. We selected PGS scores derived from the largest published study in European populations. PGS were available for 11 traits from European populations and details are presented in Supplementary Table 5. Weighted PGS were calculated for each subject in QGP based on the scoring files using PLINK ver. 2.0^19^. Pearson’s correlation (R) between the trait values and PGS were calculated using R.

### Reporting summary

Further information on research design is available in the Nature Research Reporting Summary linked to this article.

## Supplementary information

Supplementary information

Descriptions of Additional Supplementary Files

Supplementary Data 1

Supplementary Data 2

Supplementary Data 3

Supplementary Data 4

Supplementary Data 5

Supplementary Data 6

Reporting Summary

## Data Availability

GWAS summary statistics generated in this study have been deposited in the NHGRI-EBI Catalog of human genome-wide association studies and can be accessed through [https://www.ebi.ac.uk/gwas/] under the accession codes GCST90013303, GCST90013304, …, GCST90013347. All other data supporting the findings of this study are available either within the article, the supplementary information and supplementary data files, or from the authors upon reasonable request. The raw whole genome sequence data are protected and are not available due to data privacy laws. Access to QBB/QGP genotype and phenotype data can be obtained through an established ISO-certified process by submitting a project request at [https://www.qatarbiobank.org.qa/research/how-to-apply-new/] which is subject to approval by the QBB IRB committee. A detailed description of the data management infrastructure for QBB was described previously^28^. Data used in this study to assess replication of known loci are available in the NHGRI-EBI Catalog of human genome-wide association studies [https://www.ebi.ac.uk/gwas/], the Phenoscanner database [http://www.phenoscanner.medschl.cam.ac.uk], and the Experimental Factor Ontology (EFO) terms database [https://www.ebi.ac.uk/ols/ontologies/efo]. The *Nhlh1* knockout mice data used in this study are available in the international mouse phenotyping consortium database [https://www.mousephenotype.org]. The polygenic scoring files used in this study are available in the Polygenic Score Catalog [http://www.pgscatalog.org].

## Code availability

The study utilized previously published analysis tools as described in the methods section. No custom tools were used in this study.
