# Supplementary material for: Whole genome sequencing in the Middle Eastern Qatari population identifies genetic associations with 45 clinically relevant traits
Source: Nat Commun. 2021 Feb 23;12:1250. doi: 10.1038/s41467-021-21381-3 (PMC7902658; doi:10.1038/s41467-021-21381-3)
Supplement: Supplementary file 1 — Supplementary information [file 41467_2021_21381_MOESM1_ESM.pdf]

Supplementary Information

Whole genome sequencing in the Middle Eastern Qatari population identifies genetic associations with 45 clinically relevant traits.

Gaurav Thareja[1,19], Yasser Al-Sarraj[2,3,19], Aziz Belkadi[1,4], Maryam Almotawa[2,3], The Qatar Genome Program Research (QGPR) Consortium*, Karsten Suhre[1,4,20] & Omar M.E. Albagha[2,3,5,20]

---

[1]Bioinformatics Core, Weill Cornell Medicine-Qatar, Education City, 24144 Doha, Qatar

[2]College of Health and Life Sciences, Hamad Bin Khalifa University, Doha, Qatar

[3]Qatar Biomedical Research Institute (QBRI), Hamad Bin Khalifa University, Doha, Qatar

[4]Department of Biophysics and Physiology, Weill Cornell Medicine, New York, U.S.A.

[5]Centre for Genomic and Experimental Medicine, Institute of Genetics and Molecular Medicine, University of Edinburgh, Edinburgh, UK

[19]These authors contributed equally: Gaurav Thareja, Yasser Al-Sarraj

[20]These authors jointly supervised this work: Karsten Suhre, Omar M. E. Albagha,

*A list of authors and their affiliations appears at the end of the paper.

email: kas2049@qatar-med.cornell.edu; oalbagha@hbku.edu.qa

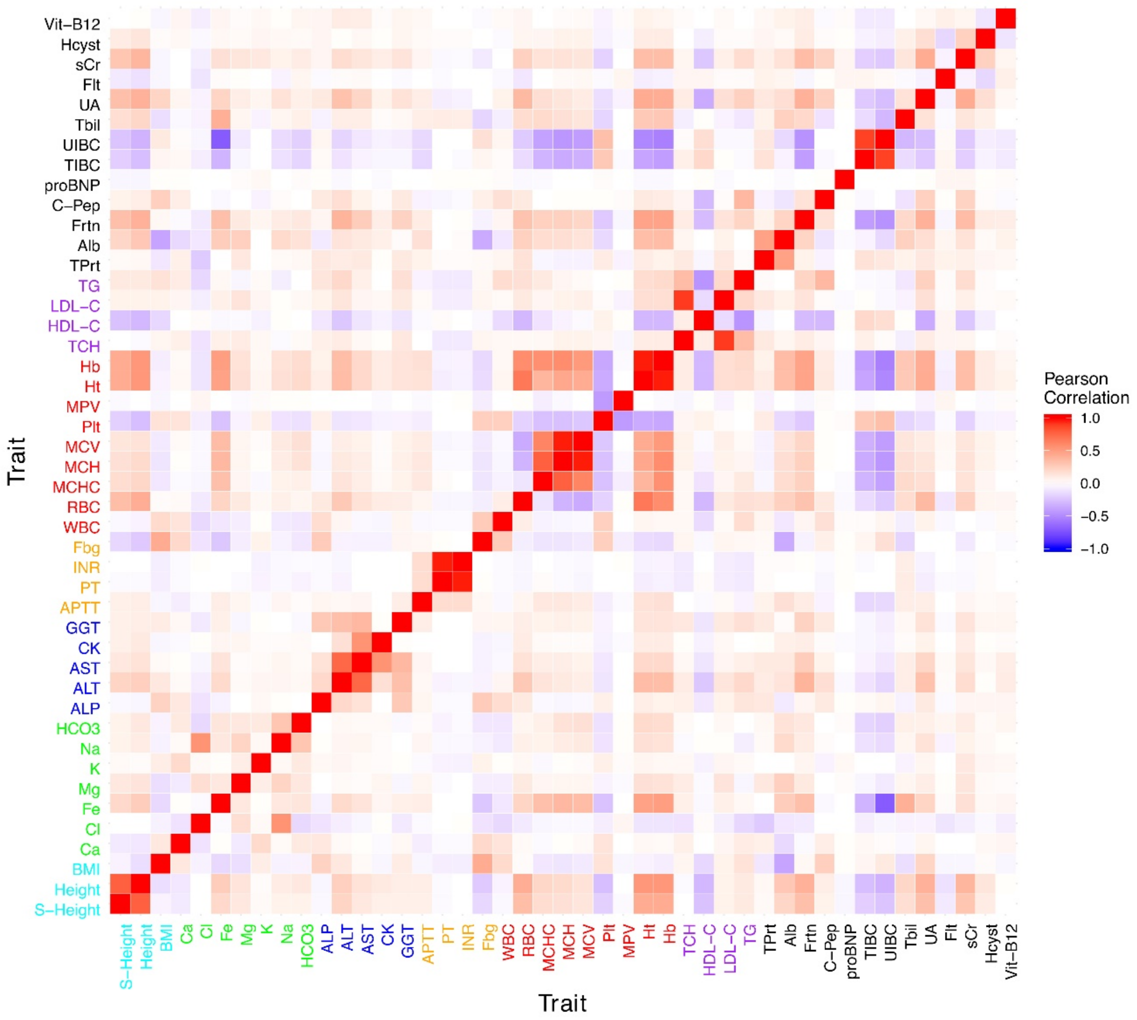

Supplementary Fig. 1 Pairwise correlations between 45 clinically relevant quantitative traits. Positive correlations are shown in red, while negative correlations are in blue.

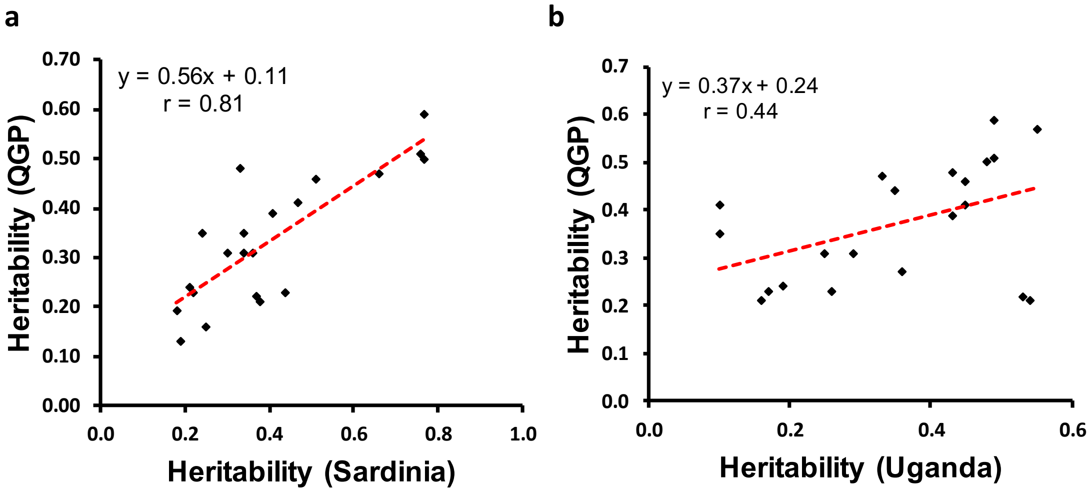

Supplementary Fig. 2 Correlation of heritability estimates between QGP and European (a) or African (b) populations. Linear regression equations and correlation coefficient r are shown in the top left corner. Dotted red line represents the line of best fit from regression analysis.

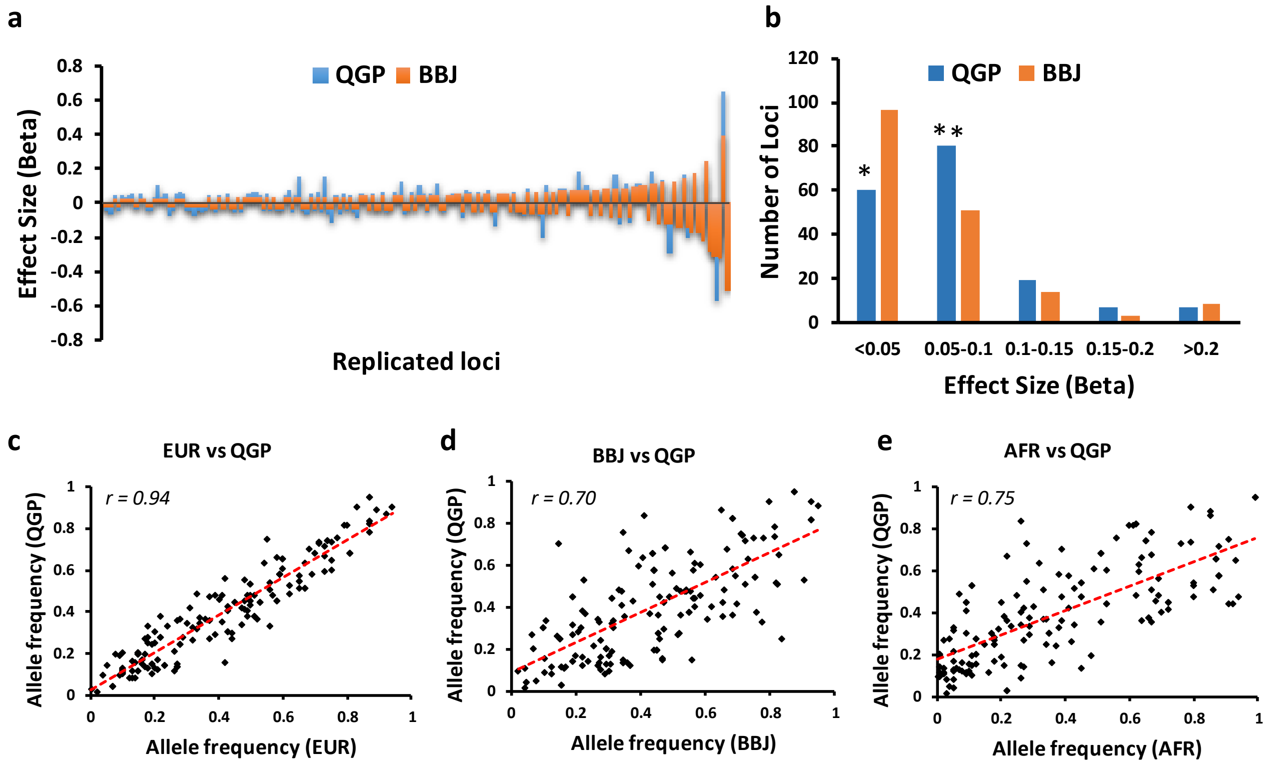

Supplementary Fig. 3. Comparison of effect size and allele frequency for loci showing nominal replication (P< 0.05) in QGP compared to Biobank Japan project (BBJ) data (Kani et al, 2018). (**a**) variant-level effect size (beta) comparison between QGP and BBJ. (**b**) Distribution of replicated loci grouped by effect size in QGP compared to BBJ using chi-square test. (**c**,**d)** Correlation of allele frequency of replicated loci between QGP and European (**c**), BBJ (**d**) and African (**e**) populations. Dotted red line indicates line of best fit from linear regression. *P=2.7 x 10^{-5}; **P=6.0x10^{-4}.

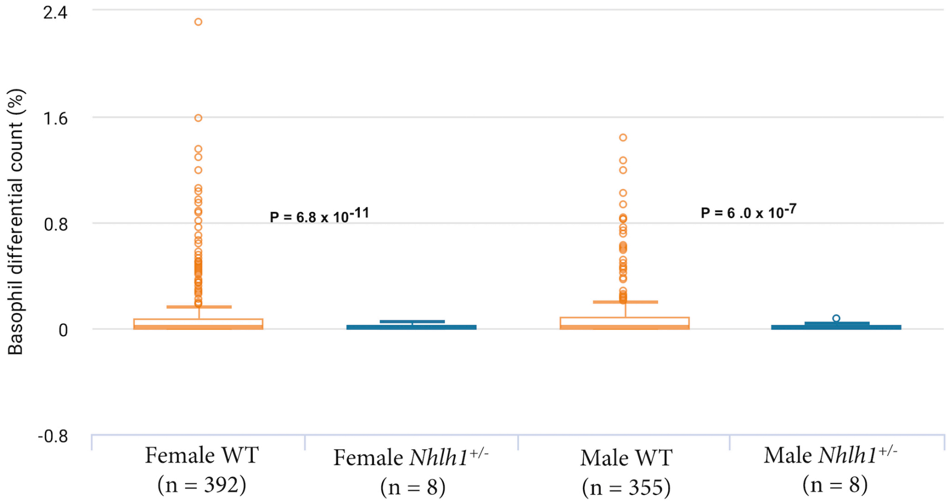

Supplementary Fig. 4. Basophil white blood cell count *in Nhlh1*$^{+/-}$ mice. The genotype level P-value was P = 3.1 x 10^{-17}. Analysis was performed using linear models. Data are presented as box and whiskers plots with median and interquartile range (IQR) and outliers (single points). Error bars represent minimum (Lower quartile – 1.5 x IQR) and maximum (upper quartile + 1.5 x IQR). Figure was adapted from the International Mouse Phenotyping Consortium (IMPC). https://www.mousephenotype.org.

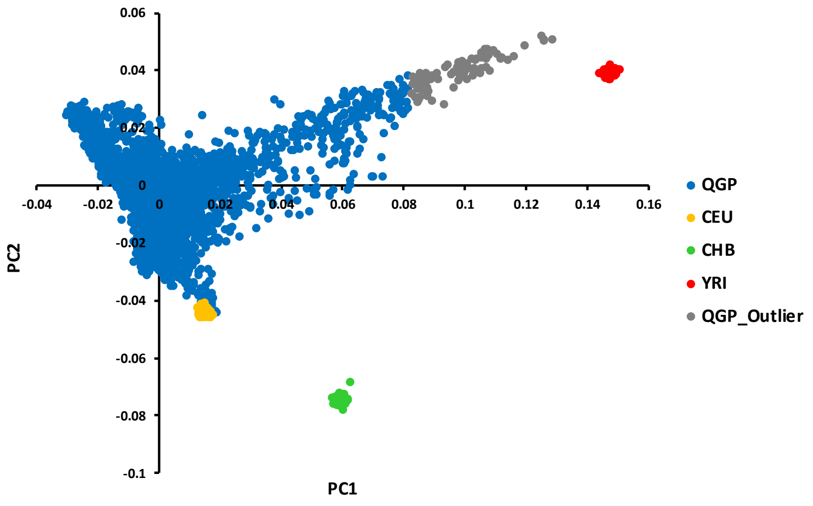

Supplementary Fig. 5. Multi-dimensional scaling results showing population clusters of QGP compared to other populations. populations. The first component (PC1) is plotted against the second component (PC2). Population outliers (shown in grey) were removed before analysis. The HapMap European (CEU), East Asian (CHB), and African (YRI) populations were used as a reference.

Supplementary Table 1. Descriptive statistics for traits invesigated in this study

Category	Trait	Abbreviation	Number	Mean	SD	Unit
Anthropometric	Sitting height	S-Height	6034	86.19	4.80	cm
	Height	Height	6044	163.90	9.38	cm
	Body mass index	BMI	6039	29.40	6.04	kg/m^2
Electrolyte	Calcium	Ca	6020	2.29	0.08	mmol/L
	Chloride	Cl	6018	101.13	2.33	mmol/L
	Iron	Fe	6010	14.77	6.25	µmol/L
	Magnesium	Mg	5996	0.83	0.07	mmol/L
	Potassium	K	6017	4.32	0.32	mmol/L
	Sodium	Na	6018	140.37	2.25	mmol/L
	Bicarbonate	HCO3	6017	26.02	2.19	mmol/L
Enzymes	Alkaline phosphatase	ALP	6012	70.50	20.94	U/L
	Alanine aminotransferase	ALT	6018	22.60	16.96	U/L
	Aspartate aminotransferase	AST	6018	19.61	10.38	U/L
	Creatine kinase	CK	5344	128.08	303.88	U/L
	Gamma glutamyl transferase	GGT	4650	28.24	28.28	U/L
Coagulation	Activated partial thromboplastin time	APTT	5988	34.40	3.43	Seconds
	Prothrombin time	PT	5989	11.78	2.06	Seconds
	International normalization ratio	INR	5987	1.05	0.18	--
	Fibrinogen	Fbg	5984	3.33	0.70	g/L
Blood Cells	White blood cell count	WBC	6007	6.72	1.95	10^3 Cell/µl
	Red blood cell count	RBC	6007	4.94	0.57	10^6 Cell/µl
	Mean corpuscular hemoglobin concentration	MCHC	6007	33.15	1.07	g/dL
	Mean corpuscular hemoglobin	MCH	6007	27.32	2.94	pg/Cell
	Mean corpuscular volume	MCV	6007	82.28	7.18	Femtoliters
	Platelet count	Plt	5935	246.51	62.96	10^3 Cell/µl
	Mean platelet volume	MPV	6006	9.09	0.94	Femtoliters
	Hematocrit	Ht	6007	40.56	4.74	%
	Hemoglobin	Hb	6006	13.46	1.76	g/dL
Lipids	Total cholesterol	TCH	6017	4.94	0.97	mmol/L
	High density lipoprotein cholesterol	HDL-C	6013	1.38	0.38	mmol/L
	Low density lipoprotein cholesterol	LDL-C	5972	2.96	0.89	mmol/L
	Triglycerides	TG	6017	1.36	0.91	mmol/L
Other biochemical	Total protein	TPrt	6017	73.03	3.86	g/L
	Albumin	Alb	6018	44.94	2.89	g/L
	Ferritin	Frtn	5947	70.88	82.74	µg/L
	C-peptide	C-Pep	5925	2.54	1.71	ng/mL
	Pro B-type natriuretic peptide	proBNP	5516	36.35	78.66	pg/mL
	Total iron binding capacity	TIBC	6010	61.23	9.76	µmol/L
	Unsaturated iron binding capacity	UIBC	5956	46.42	13.13	µmol/L
	Total bilirubin	Tbil	6018	7.38	4.61	µmol/L
	Uric acid	UA	6013	294.01	80.72	µmol/L
	Folate	Flt	5944	23.01	7.84	nmol/L
	Serum creatinine	sCr	6018	66.24	21.71	µmol/L
	Homocysteine	Hcyst	5727	8.96	4.45	µmol/L
	Vitamin B12	Vit-B12	5865	294.44	157.73	pmol/L

Supplementary Table 2. Comparison of adjusted heritability between QGP and other populations

	QGP[1]		Uganda[2]		Sardinia[3]		*P* value[4]	
Trait	h^2	SE	h^2	SE	h^2	SE	QGP vs Uganda	QGP vs Sardinia
Height	0.59	0.03	0.49	0.05	0.77	0.02	8.64E-02	**6.05E-07**
BMI	0.31	0.03	0.29	0.05	0.36	0.02	7.32E-01	1.66E-01
Fe	0.13	0.03	NA	NA	0.19	0.02	NA	9.61E-02
K	0.19	0.03	NA	NA	0.18	0.02	NA	7.82E-01
Na	0.16	0.03	NA	NA	0.25	0.02	NA	1.26E-02
ALP	0.41	0.03	0.10	0.05	NA	NA	**1.08E-07**	NA
ALT	0.23	0.03	0.26	0.05	0.22	0.02	6.07E-01	7.82E-01
AST	0.24	0.03	0.19	0.05	0.21	0.02	3.91E-01	4.05E-01
GGT	0.35	0.03	0.10	0.04	0.34	0.02	**5.84E-07**	7.82E-01
Fbg	0.35	0.03	NA	NA	0.24	0.02	NA	2.29E-03
WBC	0.48	0.03	0.43	0.10	0.33	0.02	6.32E-01	**3.20E-05**
RBC	0.47	0.03	0.33	0.11	0.66	0.02	2.20E-01	**1.39E-07**
MCHC	0.44	0.03	0.35	0.09	NA	NA	3.43E-01	NA
MCH	0.50	0.03	0.48	0.12	0.77	0.02	8.72E-01	**7.45E-14**
MCV	0.51	0.03	0.49	0.11	0.76	0.02	8.61E-01	**4.31E-12**
Plt	0.46	0.03	0.45	0.10	0.51	0.02	9.24E-01	1.66E-01
MPV	0.57	0.03	0.55	0.09	NA	NA	8.33E-01	NA
Ht	0.21	0.03	0.16	0.10	NA	NA	6.32E-01	NA
Hb	0.23	0.03	0.17	0.09	0.44	0.02	5.27E-01	**5.88E-09**
TCH	0.22	0.03	0.53	0.05	0.37	0.02	**1.08E-07**	**3.20E-05**
HDL-C	0.41	0.03	0.45	0.05	0.47	0.02	4.93E-01	9.61E-02
LDL-C	0.21	0.03	0.54	0.05	0.38	0.02	**1.56E-08**	**2.44E-06**
TG	0.31	0.03	0.25	0.05	0.30	0.02	3.04E-01	7.82E-01
Alb	0.27	0.03	0.36	0.05	NA	NA	1.23E-01	NA
Tbil	0.39	0.03	0.43	0.04	0.41	0.02	4.24E-01	5.79E-01
UA	0.31	0.03	NA	NA	0.34	0.02	NA	4.05E-01

[1]Heritability was determined using the polygenic GenABEL method adjusted for age, sex, genomic kinship and the first four principal components. [2]Gurdasani et al. 2019. Cell 179:984-1002. [3]GPilia et al. 2006. PLoS Genet 8:e132. [4]Comparion of heritability between populations was performed using two-tailed Z-test. Bold text indicates statistical significance after correction for multiple testing P<0.002.

Supplementary Table 3. Comparison of heritability between QGP and other populations

	QGP[1]		Uganda[2]		Sardinia[3]		Iceland[4]		*P* value[5]		
Trait	h^2	SE	h^2	SE	h^2	SE	h^2	SE	QGP vs Uganda	QGP vs Sardinia	QGP vs Iceland
Height	0.64	0.03	0.50	0.05	0.80	0.02	0.69	0.02	1.64E-02	**9.18E-06**	1.66E-01
BMI	0.31	0.03	0.37	0.04	0.43	0.02	0.42	0.02	2.30E-01	**8.77E-04**	**2.28E-03**
Fe	0.14	0.03	NA	NA	0.19	0.02	NA	NA	NA	1.66E-01	NA
K	0.21	0.03	NA	NA	0.18	0.02	NA	NA	NA	4.05E-01	NA
Na	0.16	0.03	NA	NA	0.25	0.02	NA	NA	NA	1.26E-02	NA
ALT	0.24	0.03	0.37	0.05	0.24	0.02	NA	NA	2.58E-02	1.00E+00	NA
AST	0.24	0.03	0.25	0.05	0.24	0.02	NA	NA	8.64E-01	1.00E+00	NA
GGT	0.30	0.03	0.11	0.04	0.34	0.02	NA	NA	**1.46E-04**	2.67E-01	NA
Fbg	0.35	0.03	NA	NA	0.27	0.02	NA	NA	NA	2.65E-02	NA
WBC	0.47	0.03	0.44	0.10	0.38	0.02	NA	NA	7.74E-01	1.26E-02	NA
RBC	0.51	0.03	0.39	0.10	0.67	0.02	NA	NA	2.50E-01	**9.18E-06**	NA
MCH	0.54	0.03	0.53	0.11	0.78	0.02	NA	NA	9.30E-01	**2.93E-11**	NA
MCV	0.57	0.03	0.57	0.10	0.76	0.02	NA	NA	1.00E+00	**1.39E-07**	NA
Plt	0.48	0.03	0.48	0.09	0.53	0.02	NA	NA	1.00E+00	1.66E-01	NA
Hb	0.23	0.03	0.20	0.09	0.47	0.02	NA	NA	7.52E-01	**2.93E-11**	NA
TCH	0.23	0.03	0.60	0.04	0.42	0.02	NA	NA	**1.46E-13**	**1.39E-07**	NA
HDL-C	0.41	0.03	0.51	0.05	0.49	0.02	0.45	0.02	8.64E-02	2.65E-02	2.67E-01
LDL-C	0.22	0.03	0.60	0.05	0.43	0.02	0.20	0.06	**7.50E-11**	**5.88E-09**	7.66E-01
TG	0.30	0.03	0.27	0.05	0.32	0.02	NA	NA	6.07E-01	5.79E-01	NA
Tbil	0.41	0.03	0.45	0.04	0.42	0.02	NA	NA	4.24E-01	7.82E-01	NA
UA	0.35	0.03	NA	NA	0.34	0.02	NA	NA	NA	7.82E-01	NA

[1]Heritability was determined using GCTA GREML method adjusted for age, sex, and the first four principal components

[2]Gurdasani et al. 2019. Cell 179:984-1002. [3]GPilia et al. 2006. PLoS Genet 2(8):e132. [4]Zaitlen et al. 2013. PLoS Genet 9(5):e1003520.

[5]Comparion of heritability between populations was performed using two-tailed Z-test. Bold text indicates statistical significance after correction for multiple testing $P<0.002$.

Supplementary Table 4. Genomic inflation factor for traits.

Trait	lambda	SE labmda
Alb	1.02	5.16E-07
ALP	1.04	5.49E-07
ALT	1.01	1.07E-06
APTT	1.03	9.61E-07
AST	1.01	4.51E-07
BMI	1.03	8.01E-07
Ca	1.01	1.72E-07
CK	1.00	5.76E-07
Cl	1.00	4.74E-07
C-Pep	0.99	3.39E-07
Fbg	1.03	2.93E-07
Fe	1.00	2.95E-07
Flt	1.02	3.86E-07
Frtn	0.99	8.12E-07
GGT	1.00	1.05E-06
Hb	1.00	3.58E-07
HCO3	1.02	6.19E-07
Hcyst	1.01	1.89E-07
HDL-C	1.04	3.35E-07
Height	1.13	8.23E-07
Ht	1.00	3.44E-07
INR	1.02	2.41E-07
K	0.99	8.03E-07
LDL-C	1.00	4.55E-07
MCH	1.07	2.95E-07
MCHC	1.03	3.82E-07
MCV	1.08	2.72E-07
Mg	1.01	4.35E-07
MPV	1.09	3.64E-07
Na	0.99	8.03E-07
Plt	1.06	5.86E-07
proBNP	1.02	8.51E-07
PT	1.04	7.59E-07
RBC	1.07	4.61E-07
sCr	1.02	4.54E-07
S-Height	1.08	2.66E-07
Tbil	1.04	4.03E-07
TCH	1.00	2.40E-07
TG	1.02	4.85E-07
TIBC	1.02	5.82E-07
TPrt	1.04	4.37E-07
UA	1.03	8.86E-07
UIBC	1.01	2.50E-07
Vit-B12	1.03	8.24E-07
WBC	1.06	8.17E-07

Supplementary Table 5. Performance of European-derived Polygenic scores (PGS) in QGP

Trait	EFO Term	Polygenic Score (PGS) ID	Sample size	Population	Reference	Number of Variants in PGS	R in European	R in QGP	Perfromance QGP/European (%)
Height	EFO_0004339	PGS000297	456,426	European	PMID:30124842	3,290	0.37	0.15	40.54%
BMI	EFO_0004340	PGS000027	288,016	European	PMID:31002795	2,100,302	0.29	0.22	75.86%
WBC	EFO_0004308	PGS000191	408,112	European	PMID:32888494	636	0.29	0.19	65.52%
RBC	EFO_0004305	PGS000187	408,112	European	PMID:32888494	678	0.34	0.22	64.71%
MCHC	EFO_0004528	PGS000175	408,112	European	PMID:32888494	224	0.23	0.12	52.17%
MCH	EFO_0004527	PGS000174	408,112	European	PMID:32888494	628	0.43	0.22	51.16%
MCV	EFO_0004526	PGS000176	408,112	European	PMID:32888494	685	0.4	0.23	57.50%
Plt	EFO_0004309	PGS000186	408,112	European	PMID:32888494	739	0.44	0.35	79.55%
MPV	EFO_0004584	PGS000179	408,112	European	PMID:32888494	654	0.52	0.51	98.08%
Ht	EFO_0004348	PGS000167	408,112	European	PMID:32888494	502	0.22	0.15	68.18%
Hb	EFO_0004509	PGS000168	408,112	European	PMID:32888494	515	0.24	0.14	58.33%

R is Pearson's correlation between the trait values and PGS.
