# Supplementary material for: Whole genome sequencing in the Middle Eastern Qatari population identifies genetic associations with 45 clinically relevant traits
Source: Nat Commun. 2021 Feb 23;12:1250. doi: 10.1038/s41467-021-21381-3 (PMC7902658; doi:10.1038/s41467-021-21381-3)
Supplement: Supplementary file 2 — Descriptions of Additional Supplementary Files [file 41467_2021_21381_MOESM2_ESM.pdf]

Descriptions of Additional Supplementary Files

Supplementary Data 1

Description: Summary of experimental methods for studied traits.

Supplementary Data 2

Description: Manhattan and quantile-quantile plots for the 45 clinically- relevant traits analyzed in this study.

Supplementary Data 3

Description: List of genome-wide significant distinct signals associated with traits.

Supplementary Data 4

Description: Replication of loci identified by Biobank Japan Project.

Supplementary Data 5

Description: Colocalization analysis of known loci showing nominal significance for replication.

Supplementary Data 6

Description: Regional association plots for the novel loci identified in this study
