# Supplementary material for: Whole genome sequencing in the Middle Eastern Qatari population identifies genetic associations with 45 clinically relevant traits
Source: Nat Commun. 2021 Feb 23;12:1250. doi: 10.1038/s41467-021-21381-3 (PMC7902658; doi:10.1038/s41467-021-21381-3)

Supplementary Data 2. Manhattan and quantile-quantile plots for the 45 clinically- relevant traits analyzed in this study. Manhattan plots of GWAS results conducted using linear mixed models adjusting for age, gender, the first four principal components and relatedness. The chromosomal position of 7,880,618 variants plotted against $-\log_{10}$ P. The horizontal red line indicates the threshold for genome-wide significance ($P< 5 \times 10^{-8}$). Quantile-quantile plots show expected versus observed $-\log_{10}$ P.

Index

Trait	Abbreviation	Page
Sitting height	S-Height	3
Height	Height	3
Body mass index	BMI	3
Calcium	Ca	4
Chloride	Cl	4
Iron	Fe	4
Magnesium	Mg	5
Potassium	K	5
Sodium	Na	5
Bicarbonate	HCO3	6
Alkaline phosphatase	ALP	6
Alanine aminotransferase	ALT	6
Aspartate aminotransferase	AST	7
Creatine kinase	CK	7
Gamma glutamyl transferase	GGT	7
Activated partial thromboplastin time	APTT	8
Prothrombin time	PT	8
International normalization ratio	INR	8
Fibrinogen	Fbg	9
White blood cell count	WBC	9
Red blood cell count	RBC	9
Mean corpuscular hemoglobin concentration	MCHC	10
Mean corpuscular hemoglobin	MCH	10
Mean corpuscular volume	MCV	10
Platelet count	Plt	11
Mean platelet volume	MPV	11
Hematocrit	Ht	11
Hemoglobin	Hb	12
Total cholesterol	TCH	12
High density lipoprotein cholesterol	HDL-C	12
Low density lipoprotein cholesterol	LDL-C	13
Triglycerides	TG	13
Total protein	TPrt	13
Albumin	Alb	14

Trait	Abbreviation	Page
Ferritin	Frtn	14
C-peptide	C-Pep	14
Pro B-type natriuretic peptide	proBNP	15
Total iron binding capacity	TIBC	15
Unsaturated iron binding capacity	UIBC	15
Total bilirubin	Tbil	16
Uric acid	UA	16
Folate	Flt	16
Serum creatinine	sCr	17
Homocysteine	Hcyst	17
Vitamin B12	Vit-B12	17

Manhattan and Q-Q plots for 45 quantitative traits

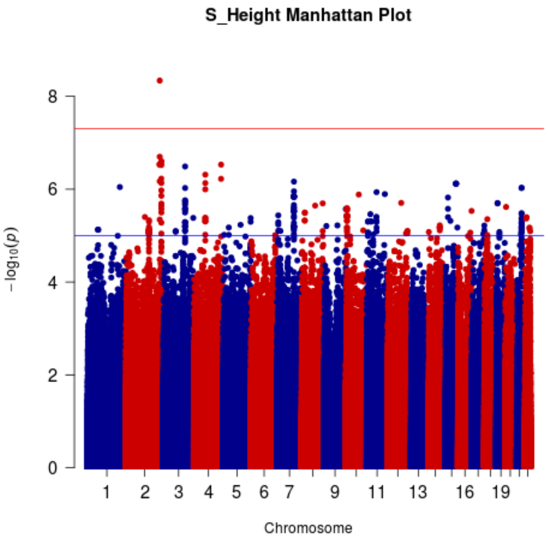

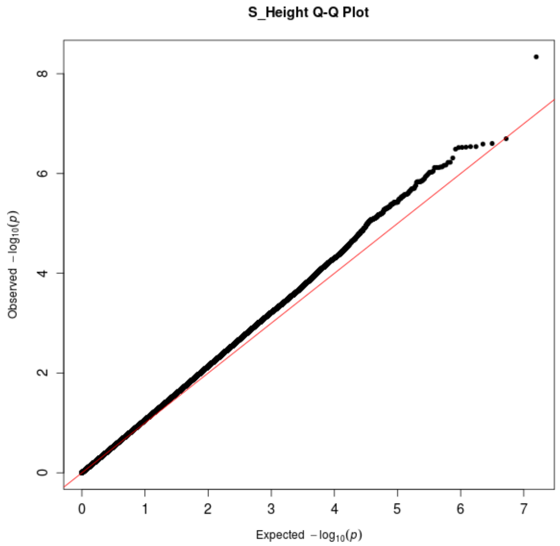

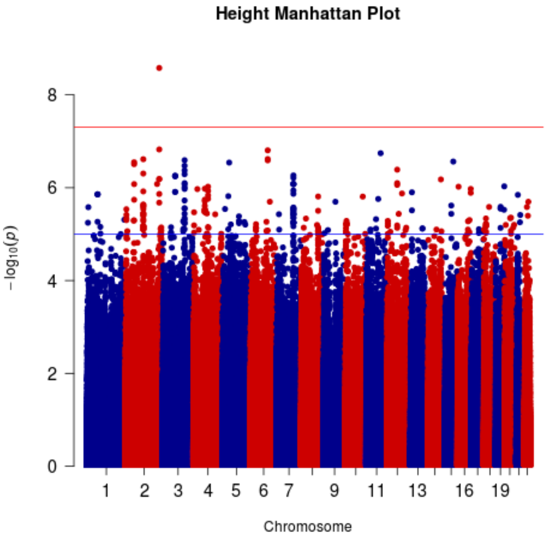

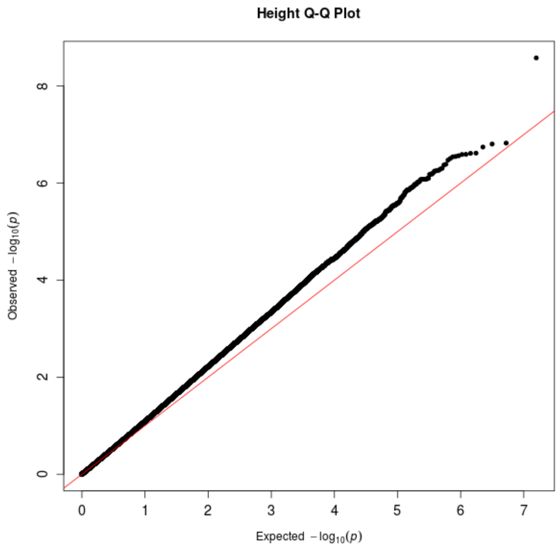

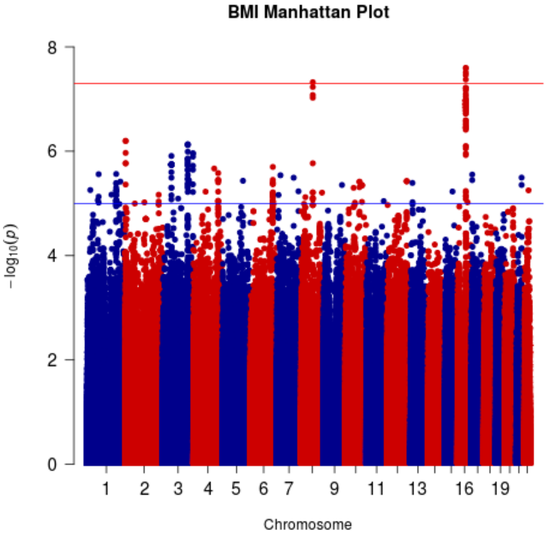

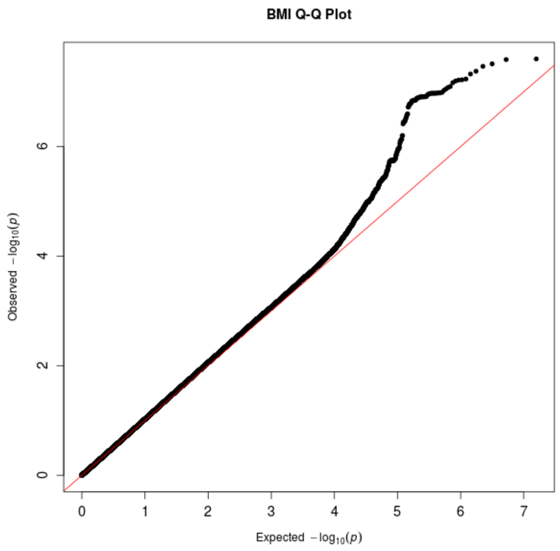

Manhattan and Q-Q plots for 45 quantitative traits (continued)

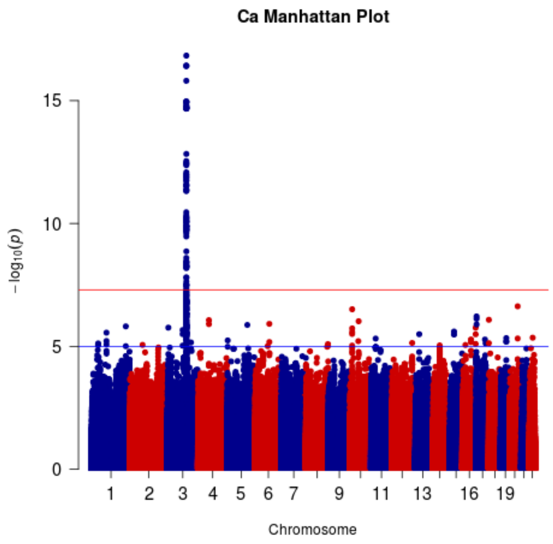

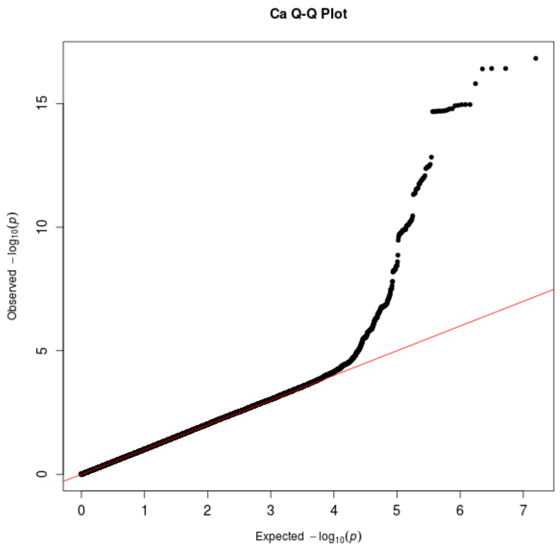

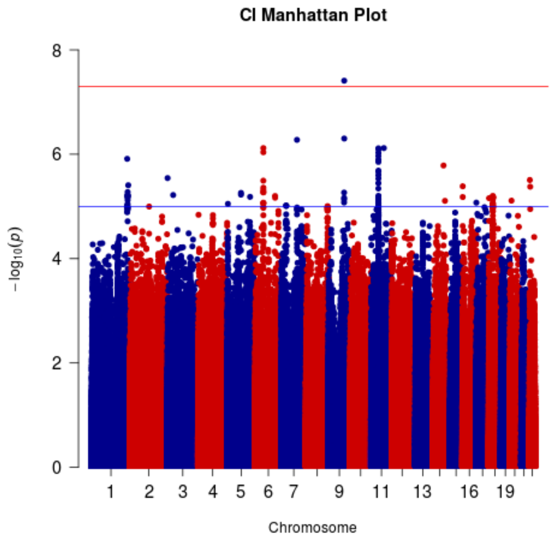

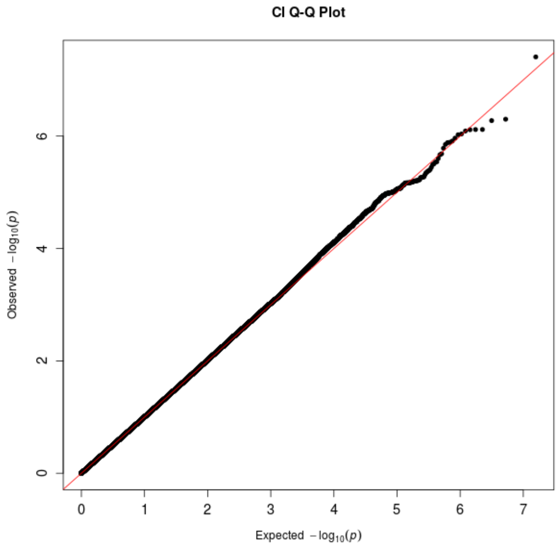

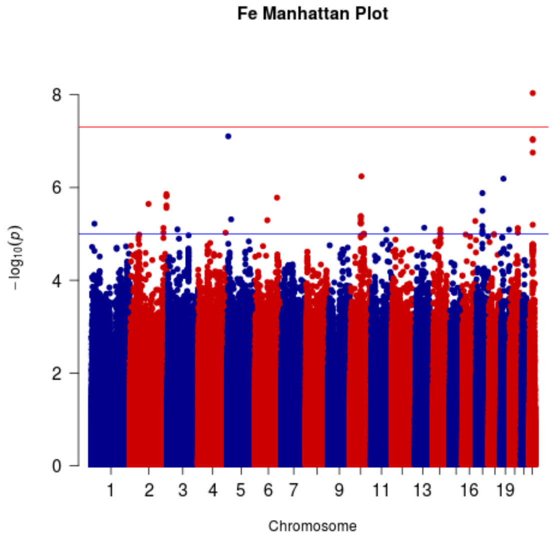

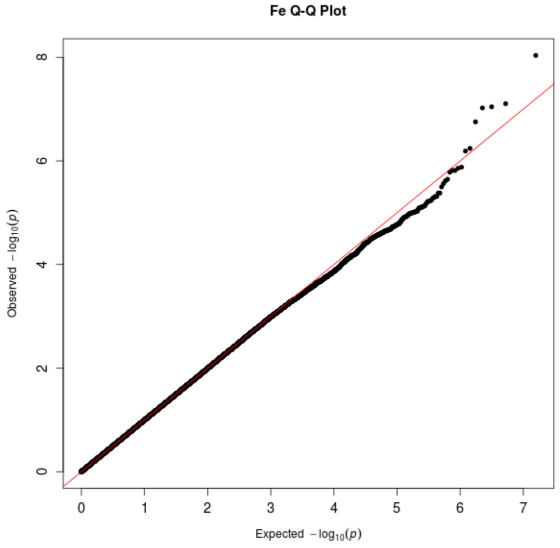

Manhattan and Q-Q plots for 45 quantitative traits (continued)

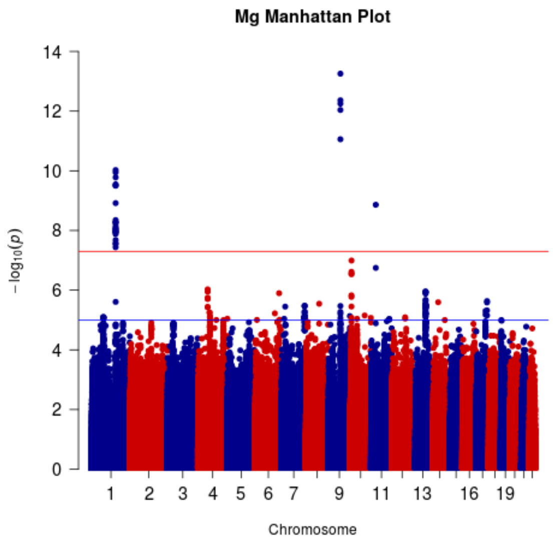

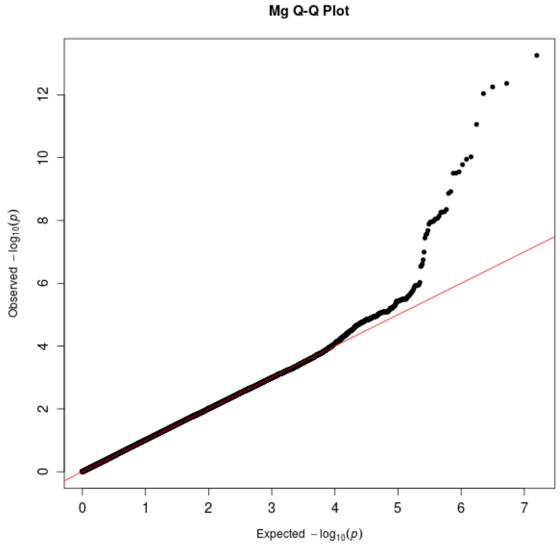

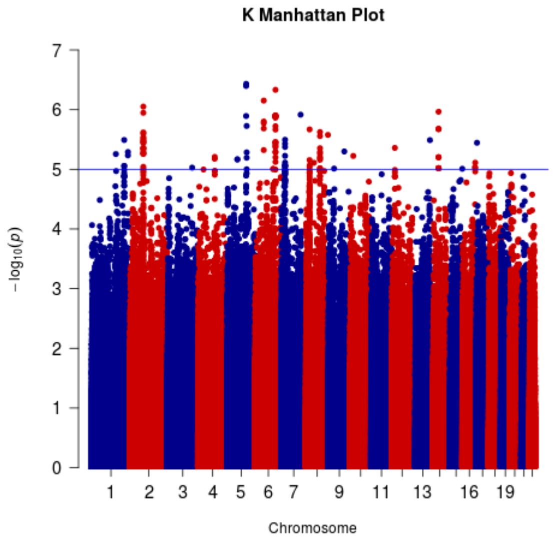

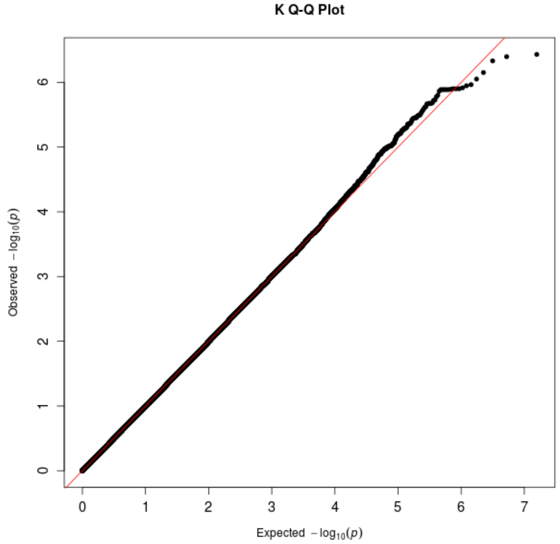

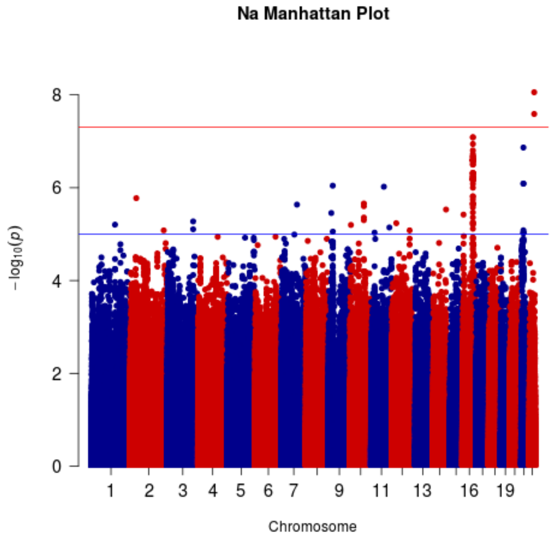

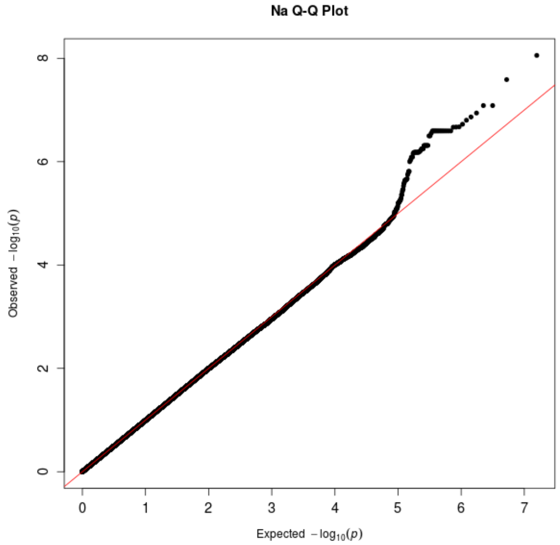

Manhattan and Q-Q plots for 45 quantitative traits (continued)

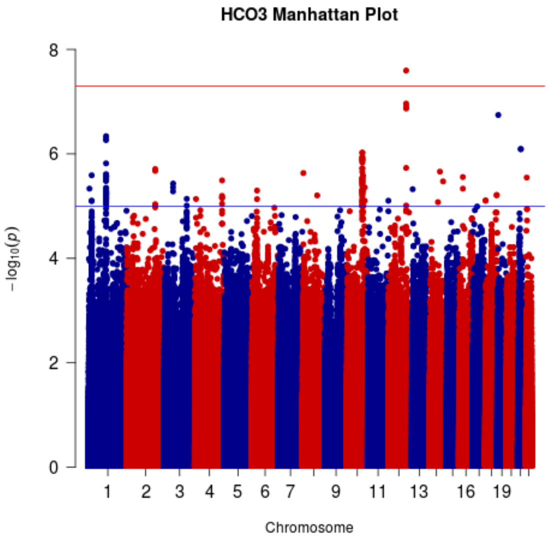

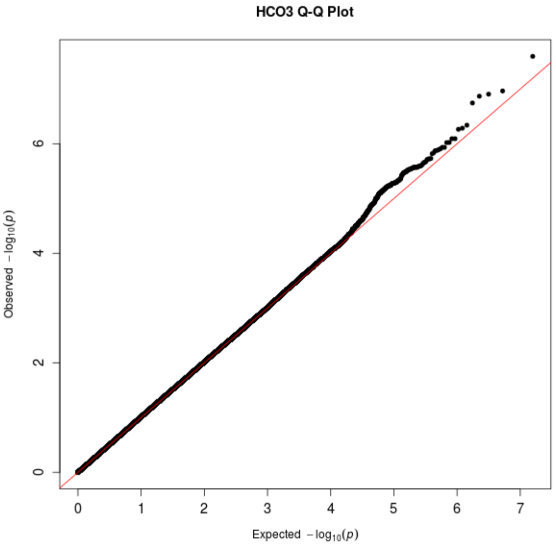

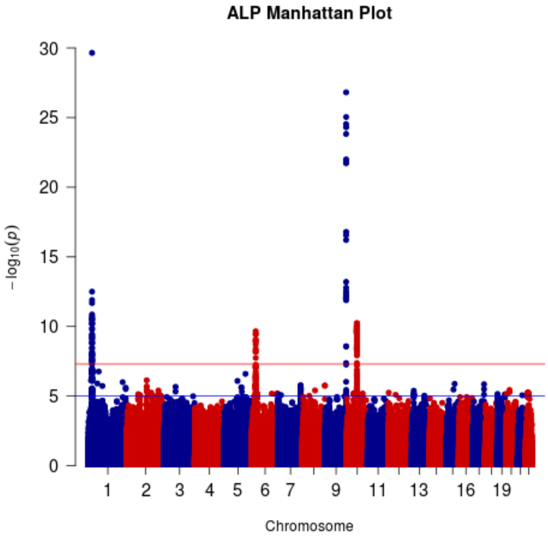

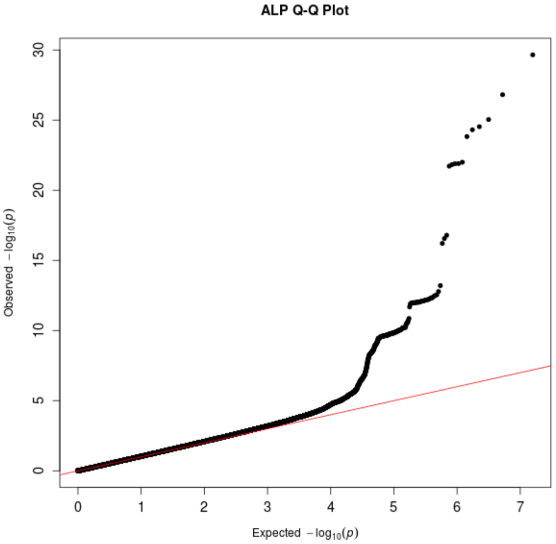

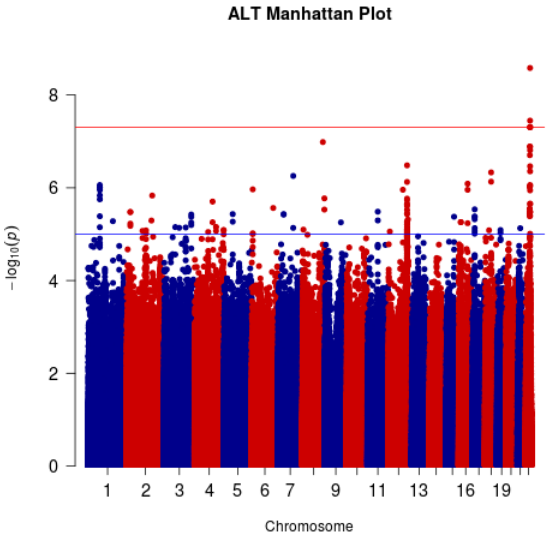

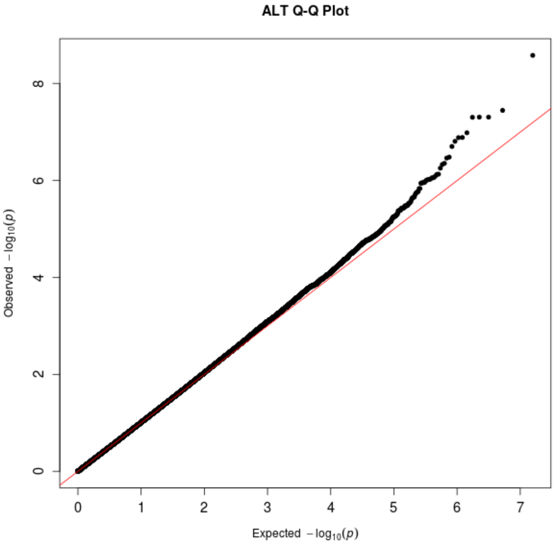

Manhattan and Q-Q plots for 45 quantitative traits (continued)

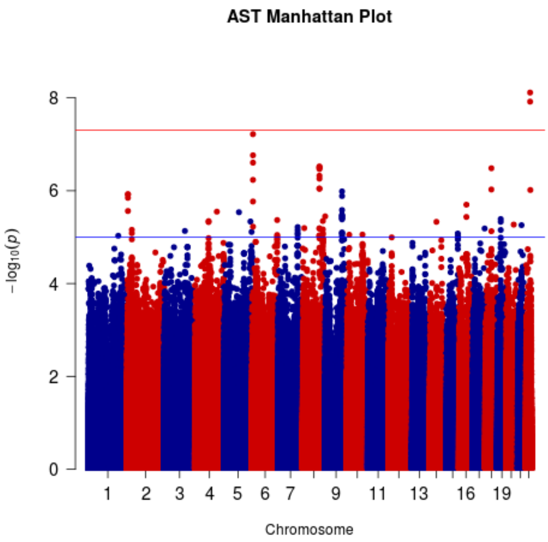

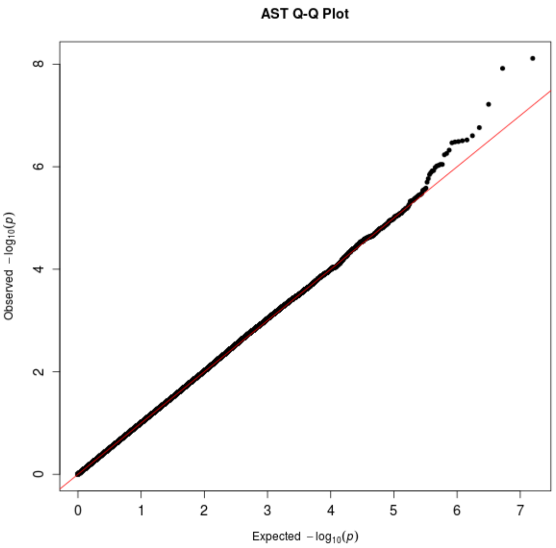

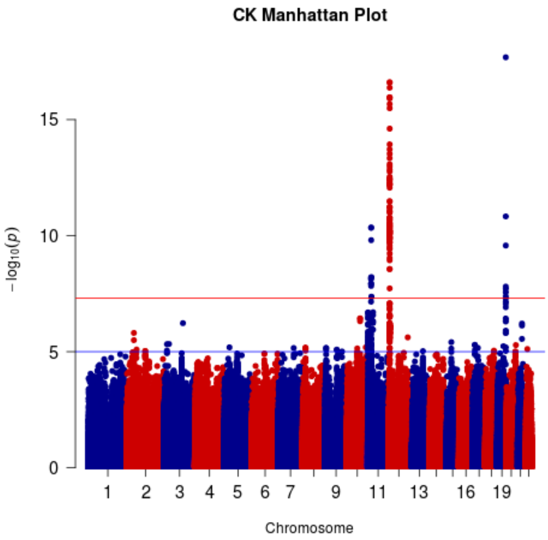

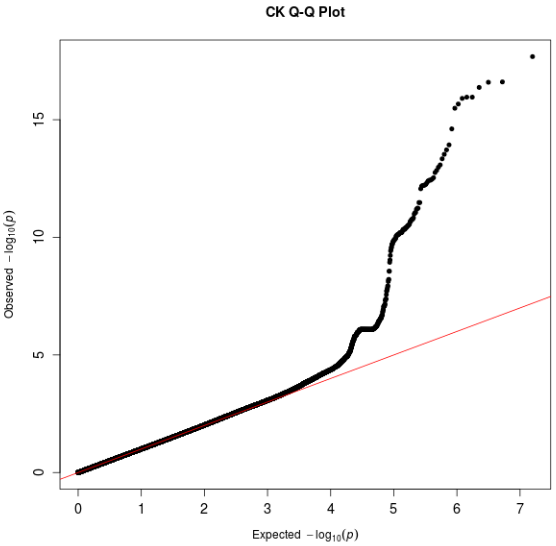

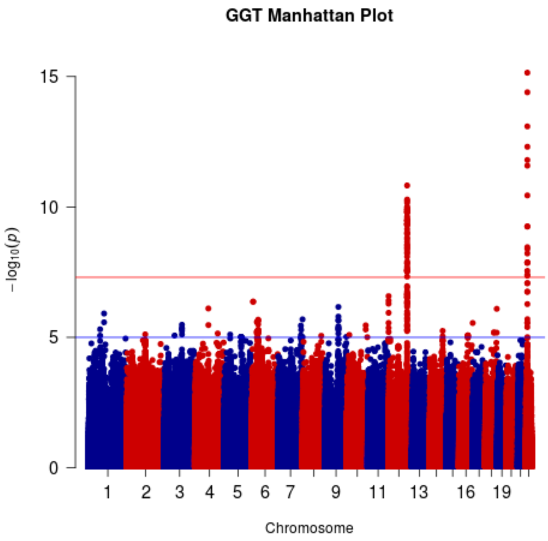

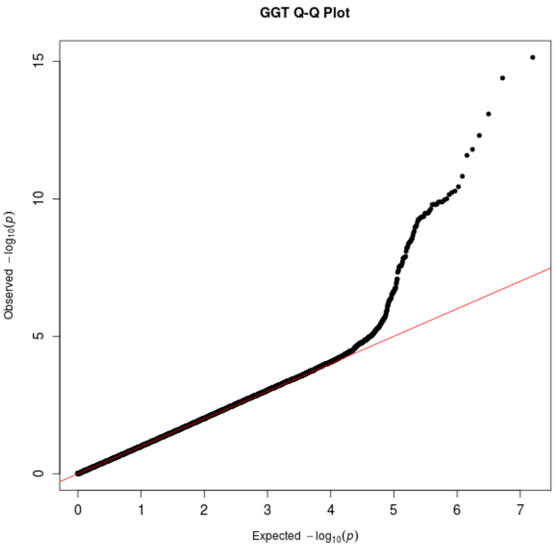

Manhattan and Q-Q plots for 45 quantitative traits (continued)

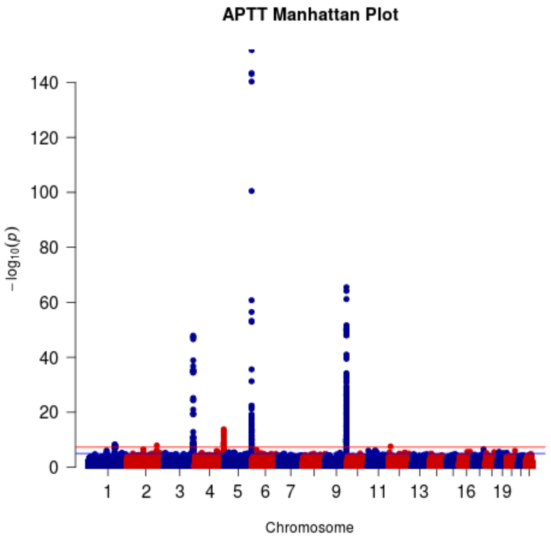

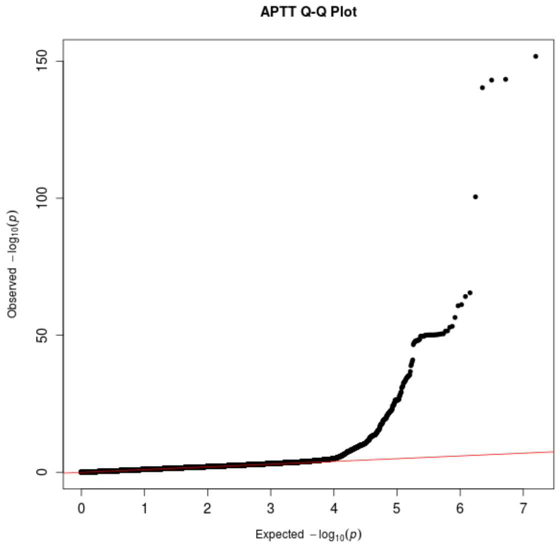

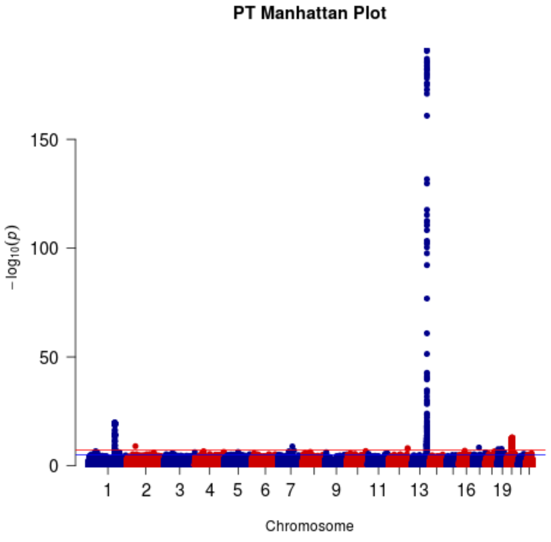

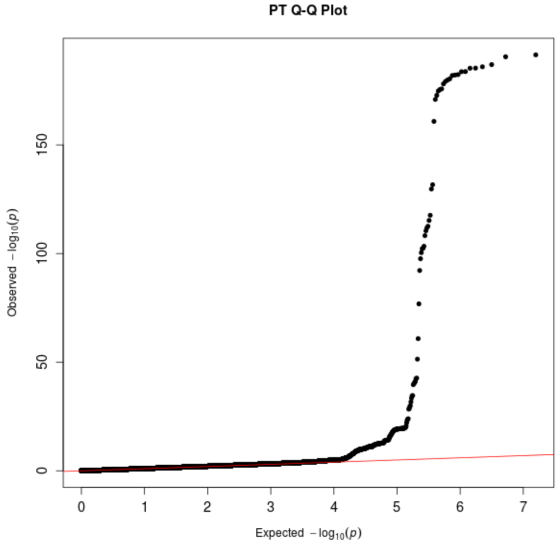

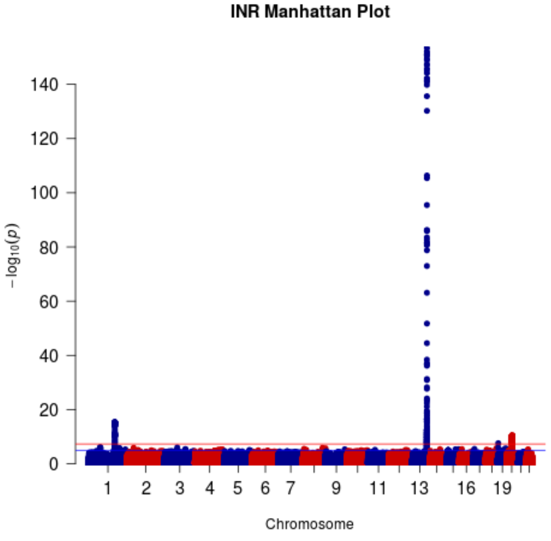

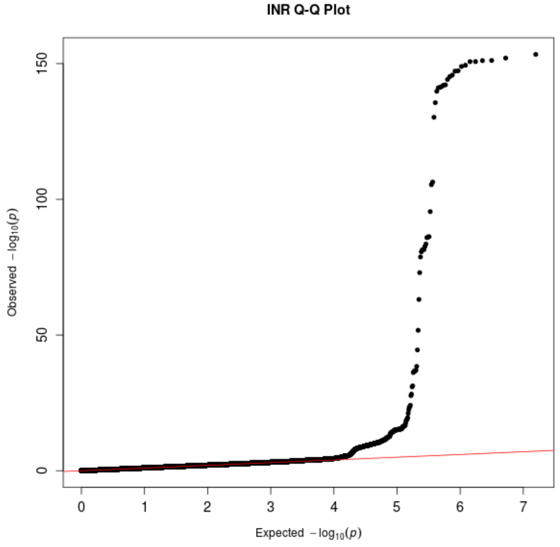

Manhattan and Q-Q plots for 45 quantitative traits (continued)

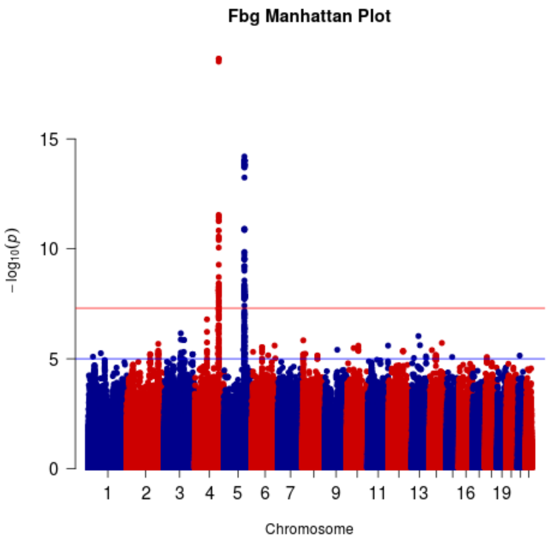

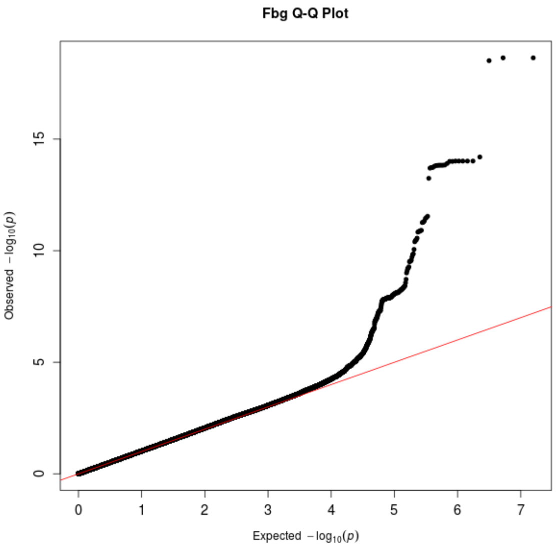

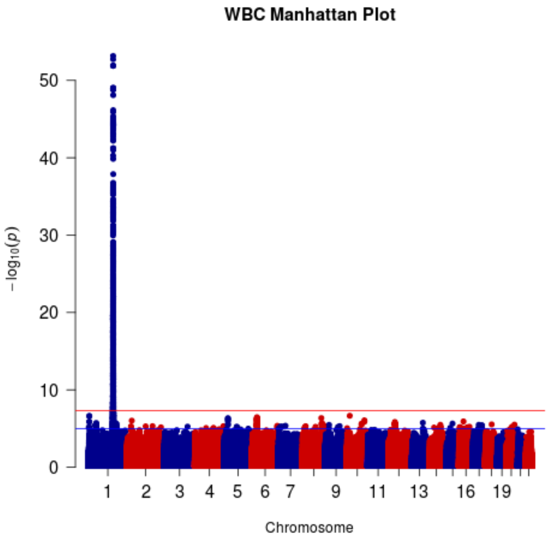

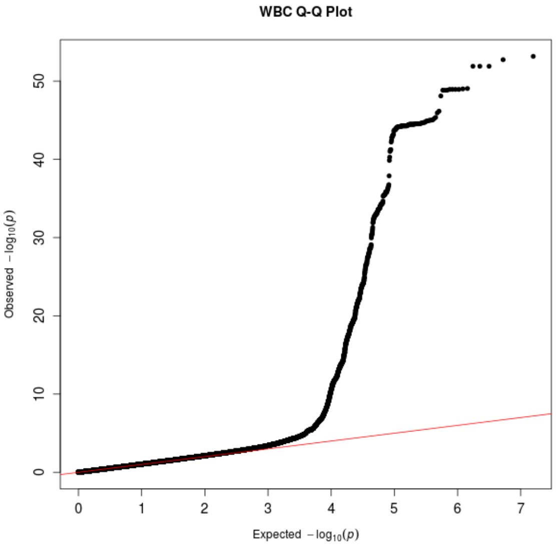

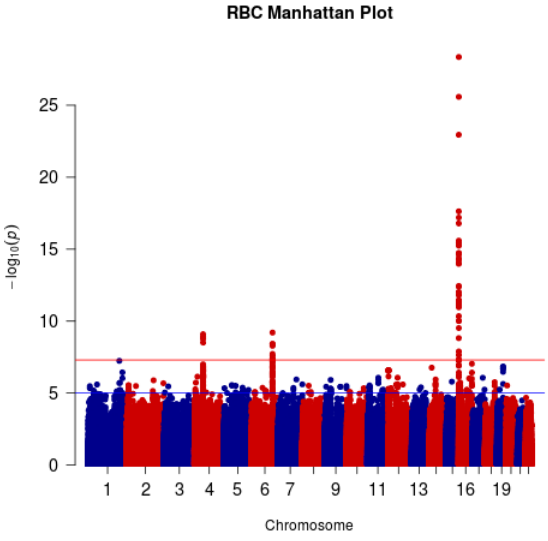

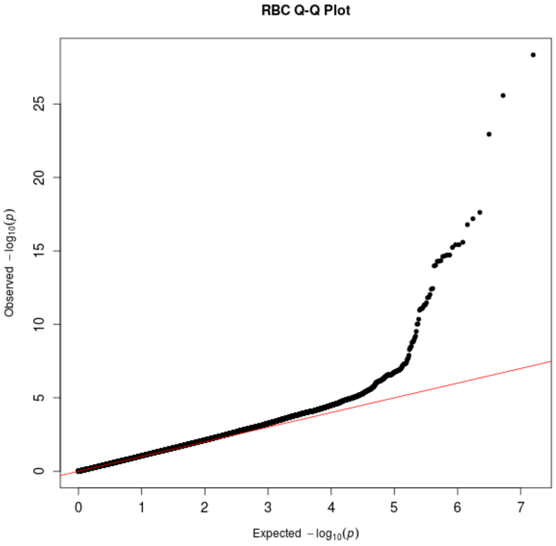

Manhattan and Q-Q plots for 45 quantitative traits (continued)

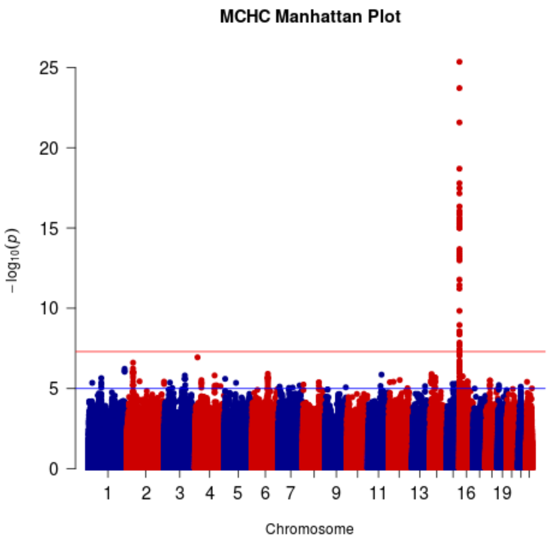

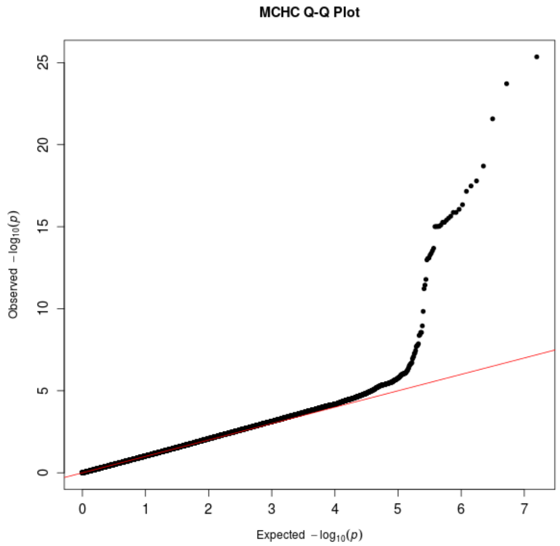

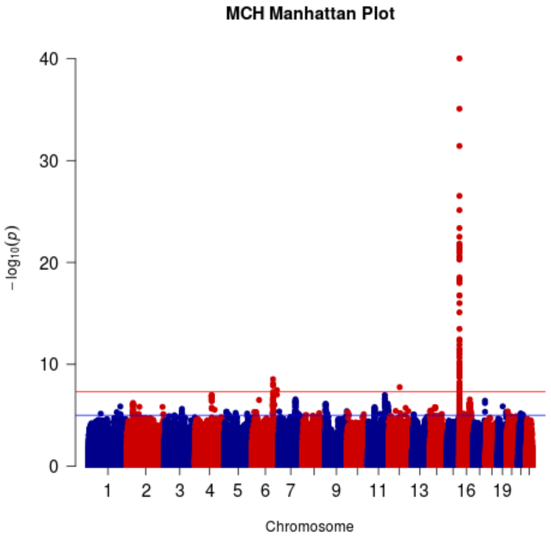

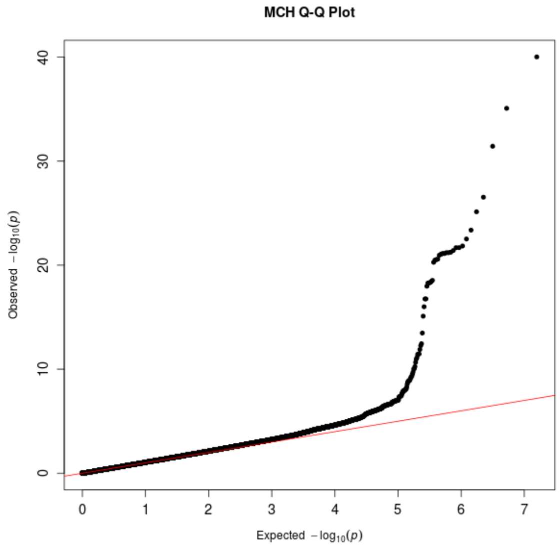

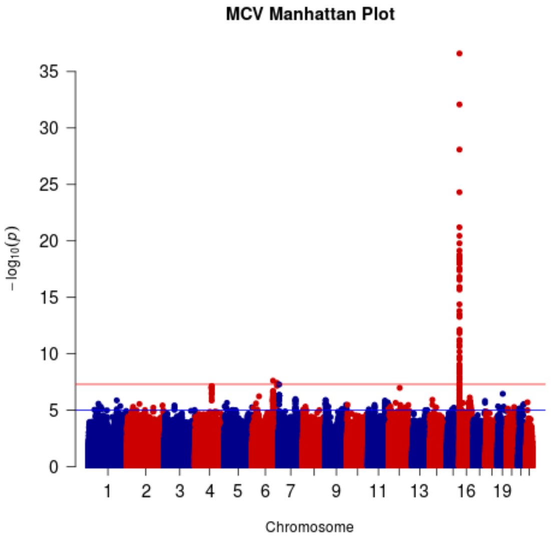

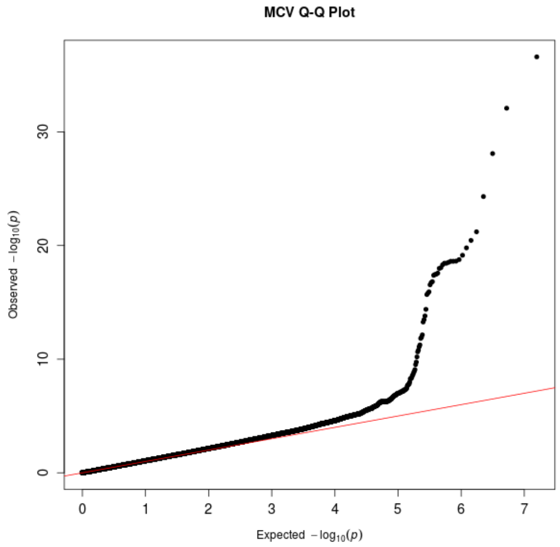

Manhattan and Q-Q plots for 45 quantitative traits (continued)

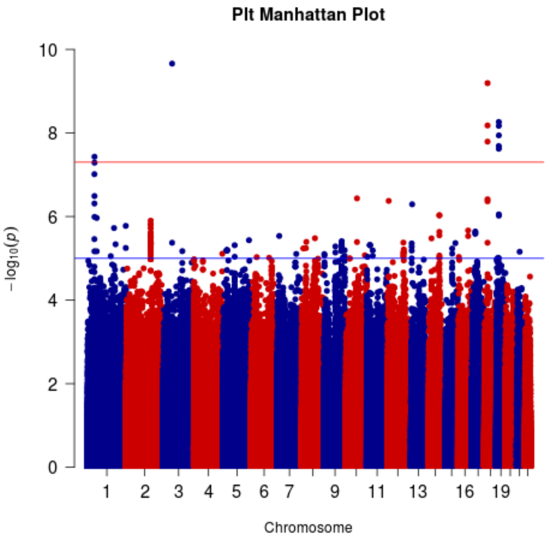

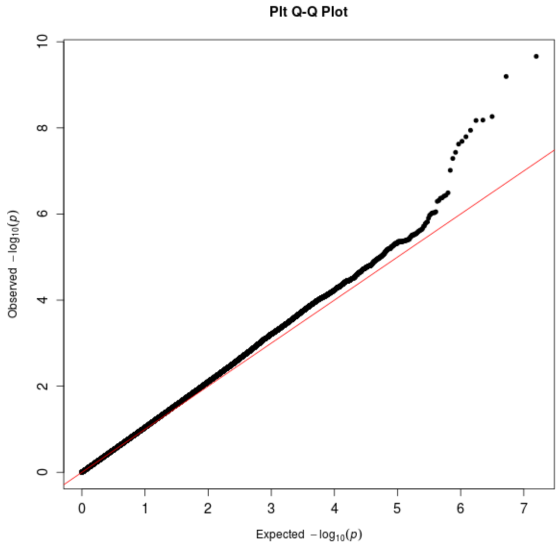

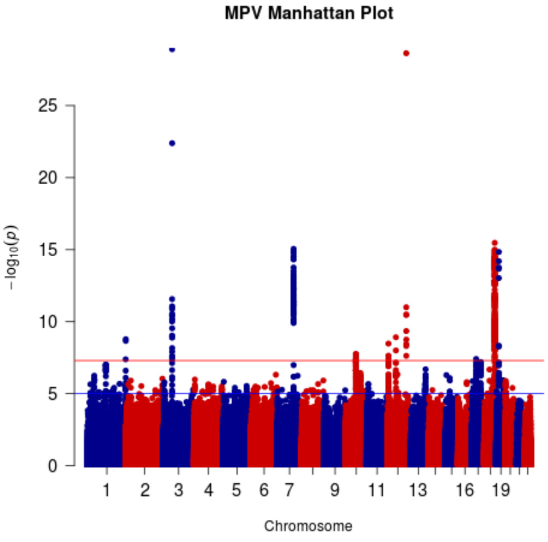

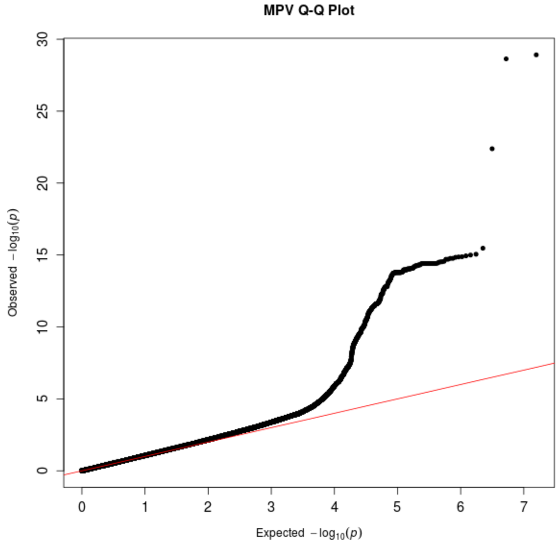

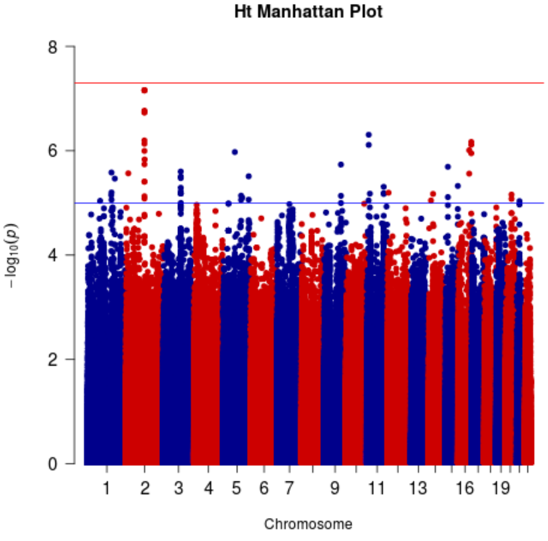

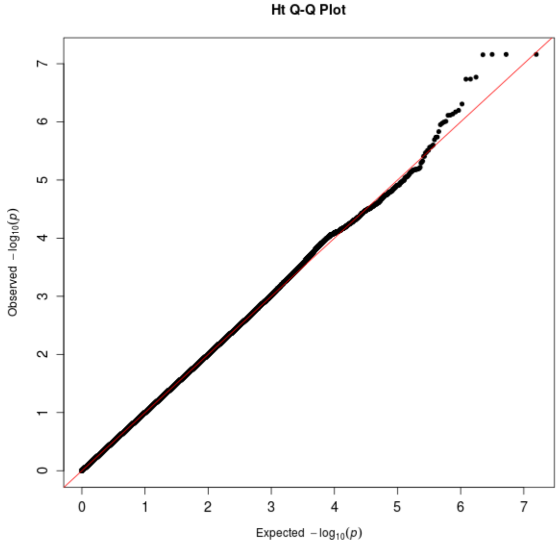

Manhattan and Q-Q plots for 45 quantitative traits (continued)

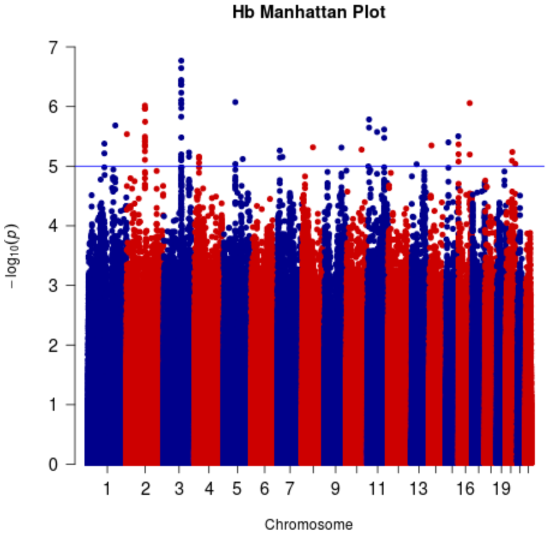

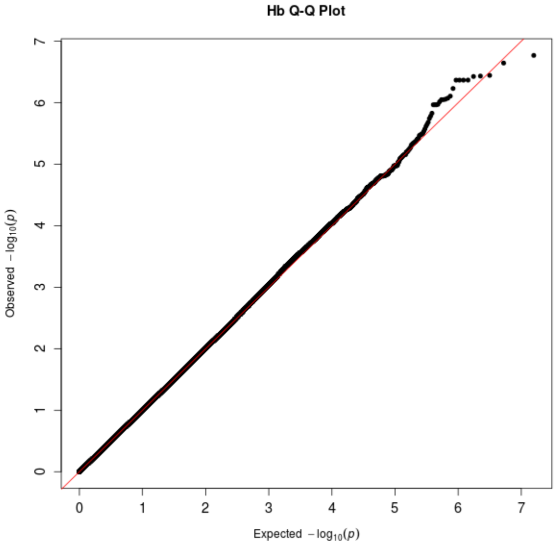

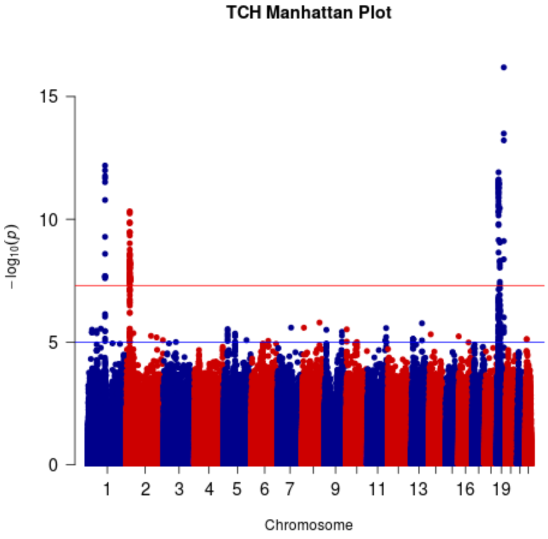

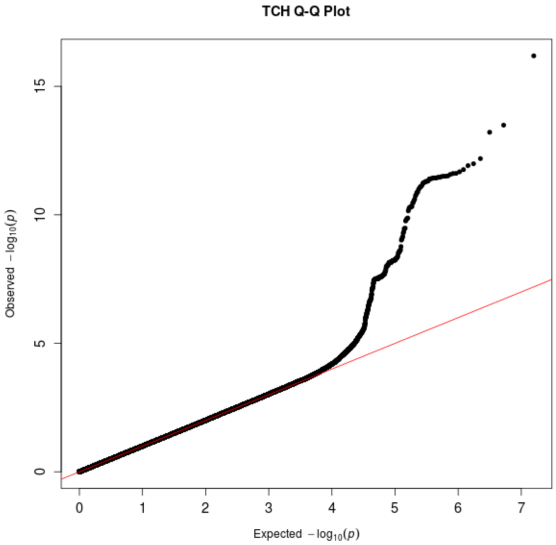

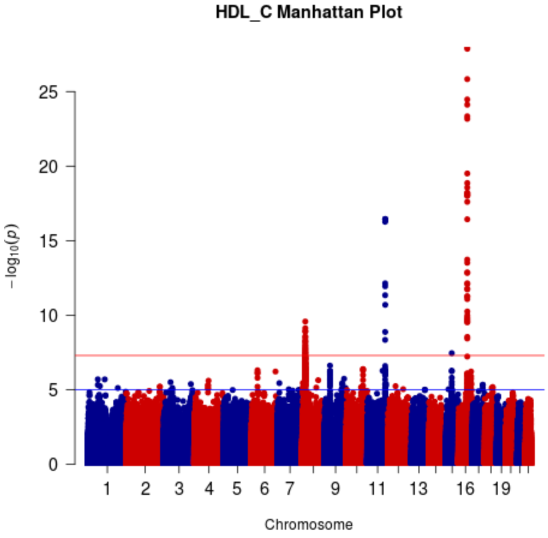

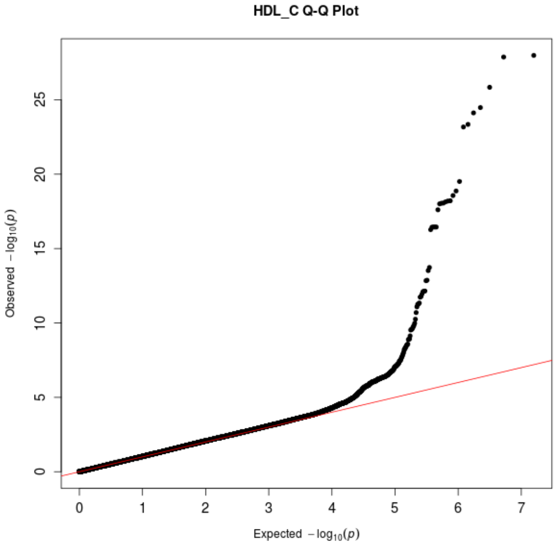

Manhattan and Q-Q plots for 45 quantitative traits (continued)

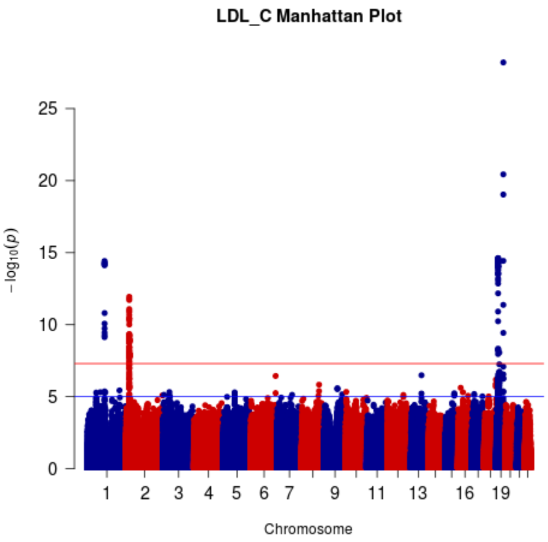

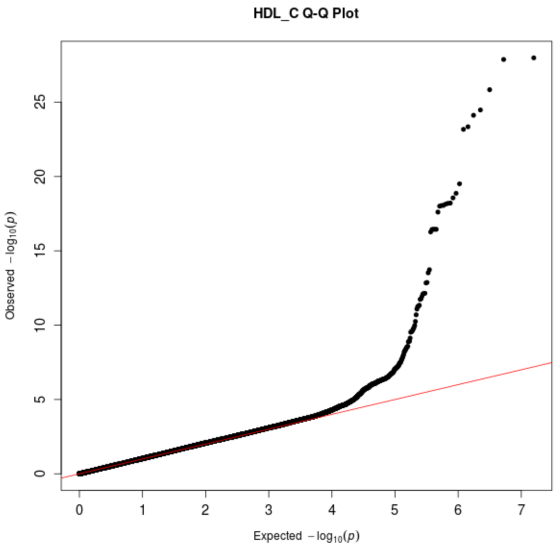

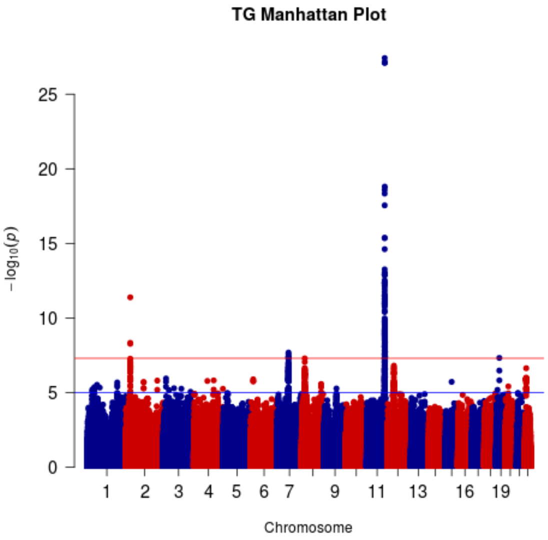

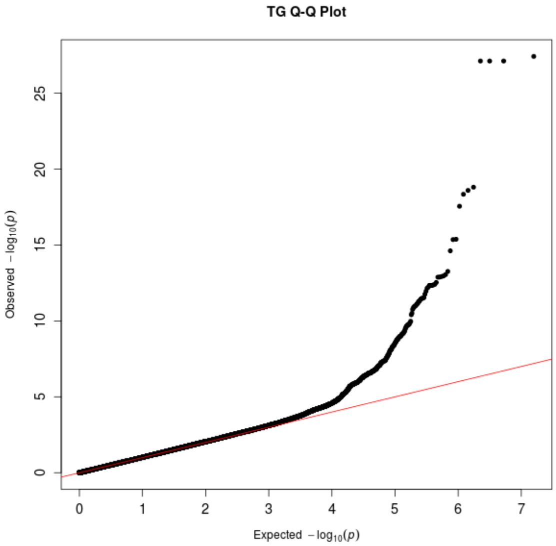

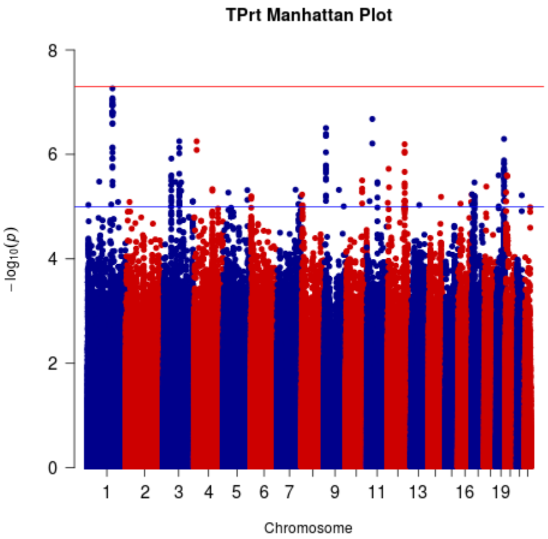

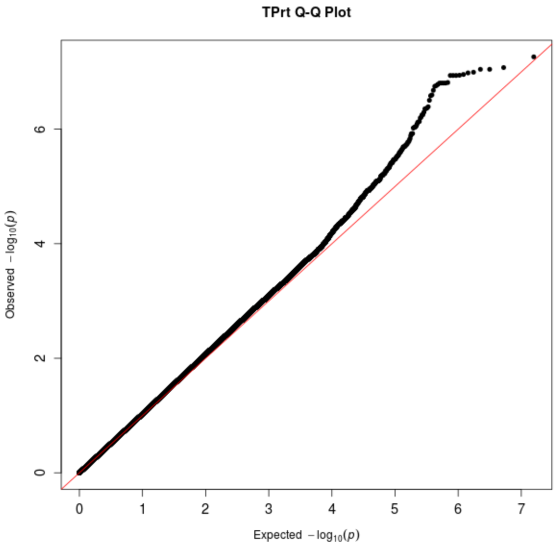

Manhattan and Q-Q plots for 45 quantitative traits (continued)

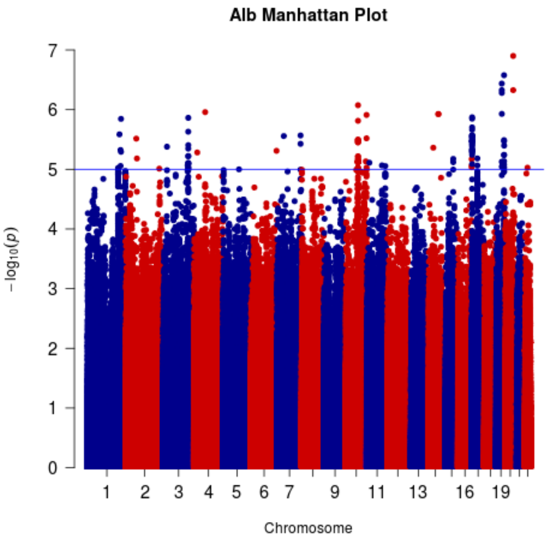

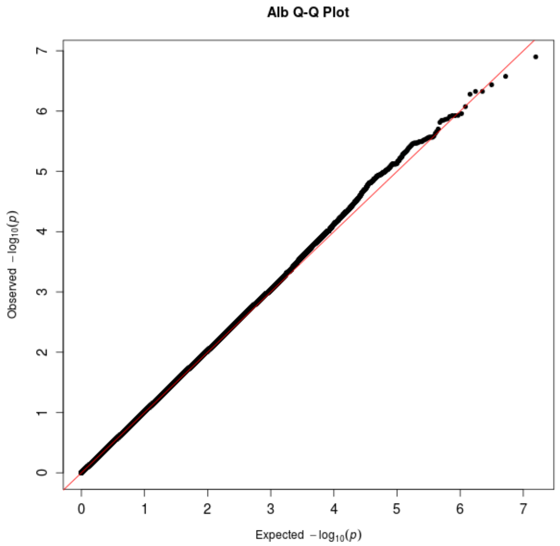

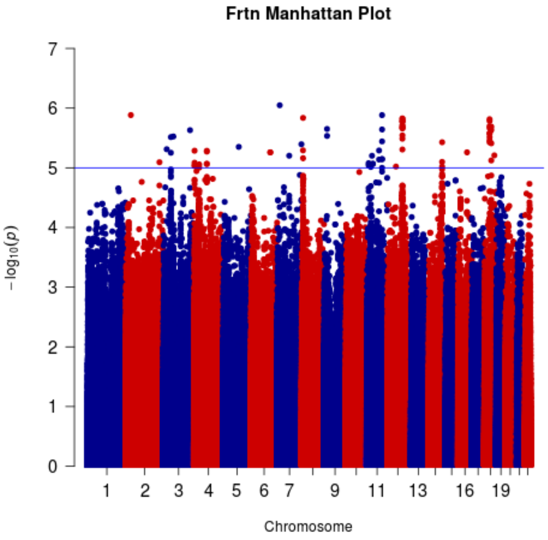

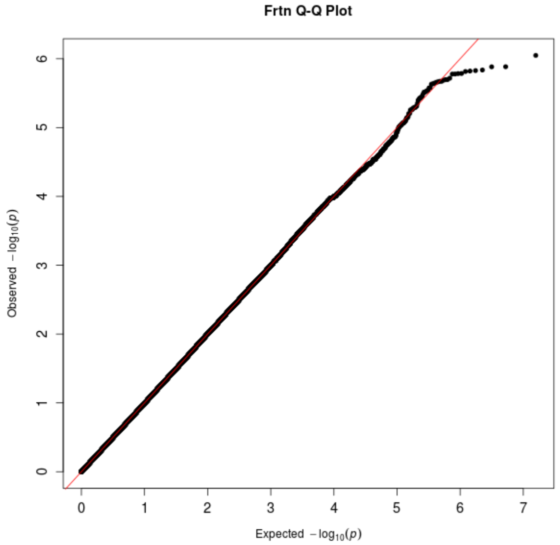

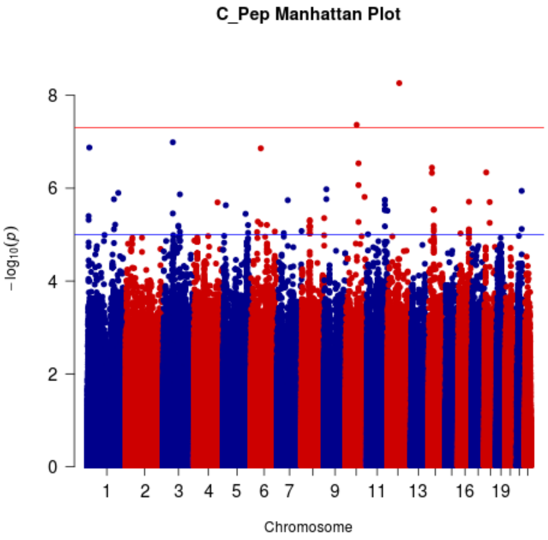

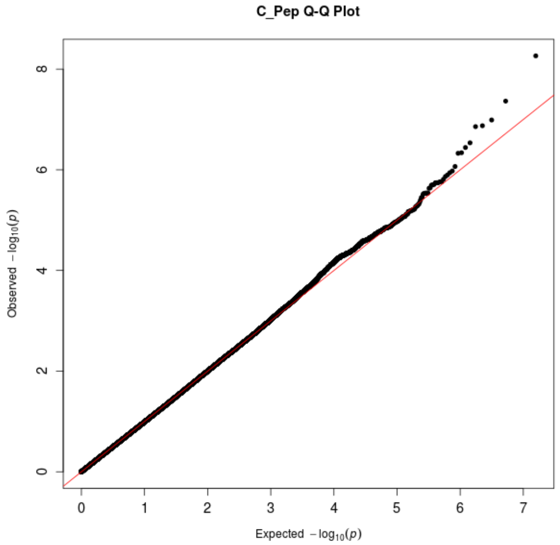

Manhattan and Q-Q plots for 45 quantitative traits (continued)

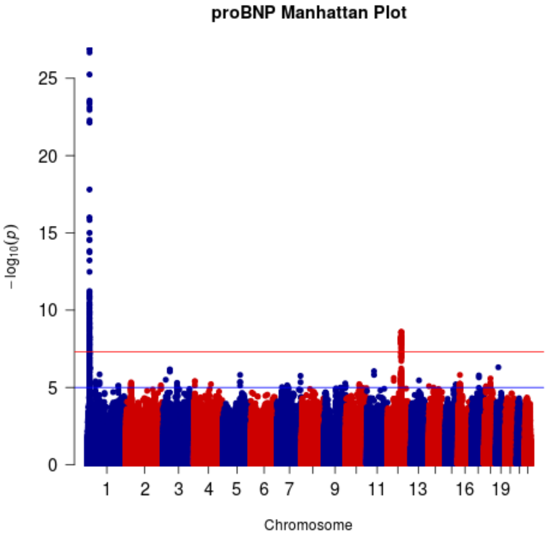

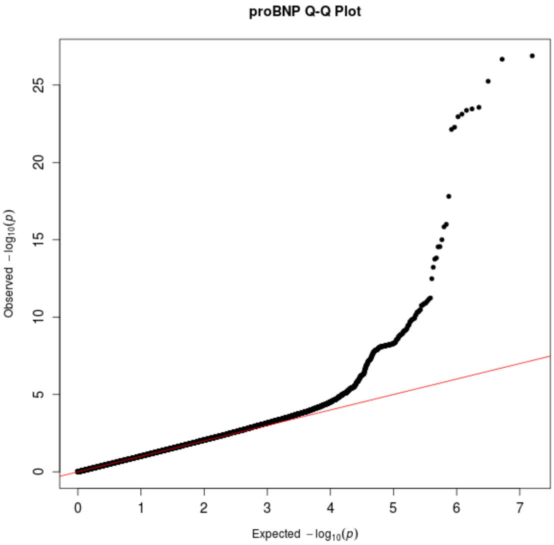

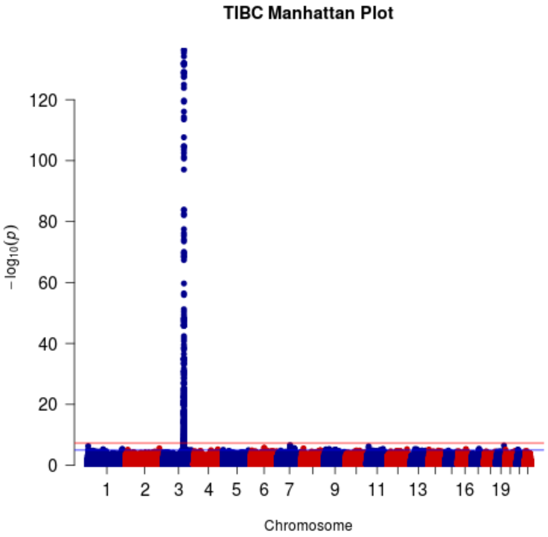

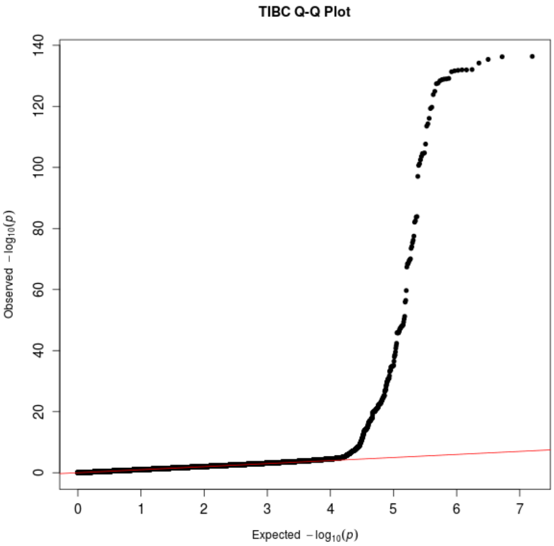

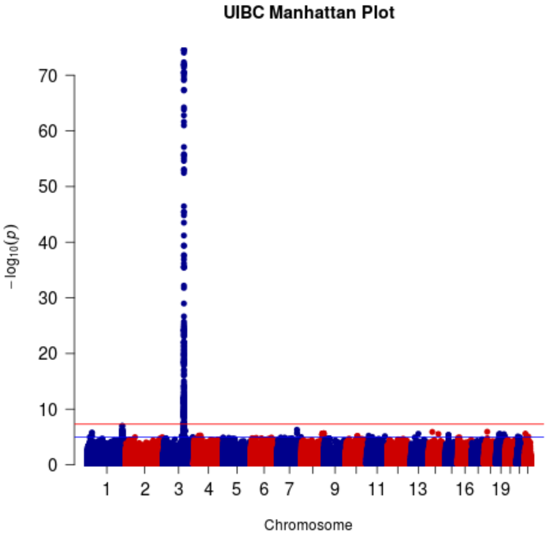

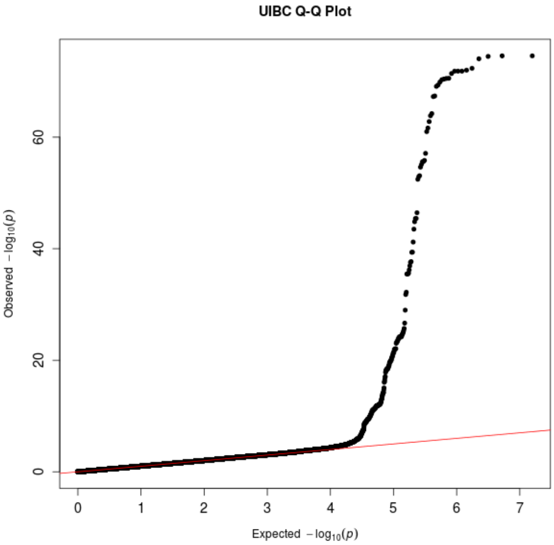

Manhattan and Q-Q plots for 45 quantitative traits (continued)

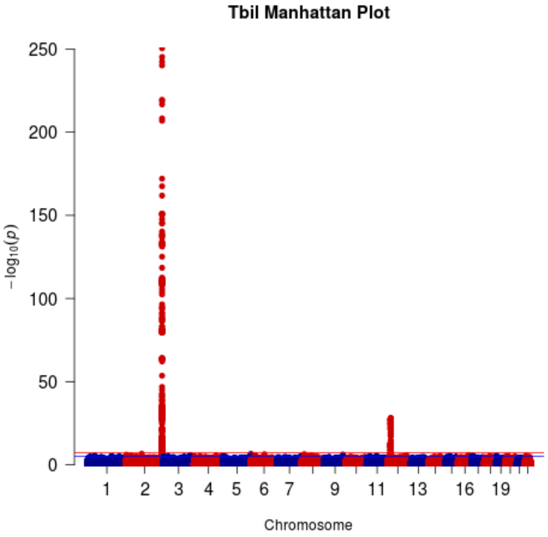

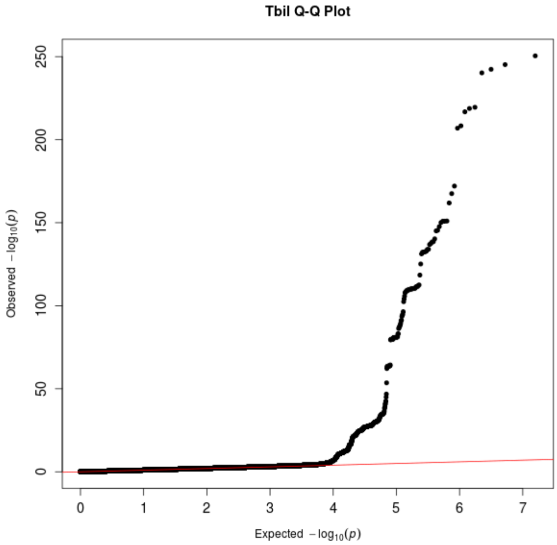

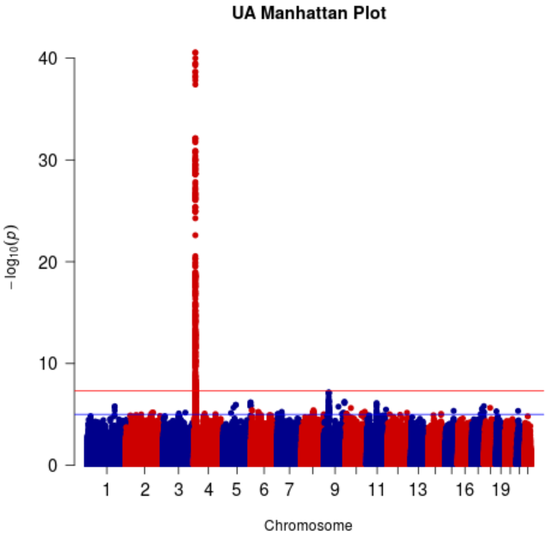

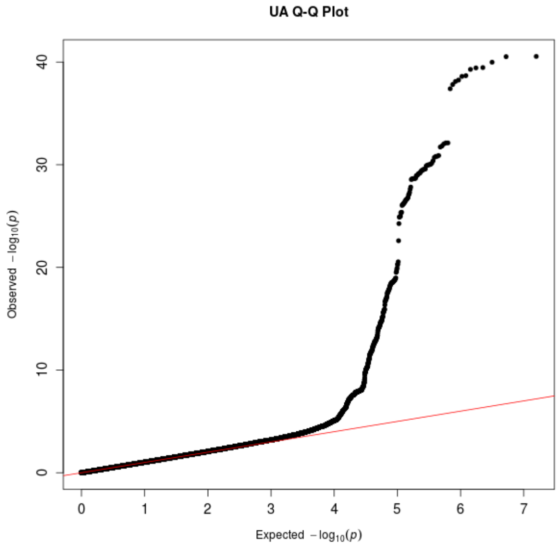

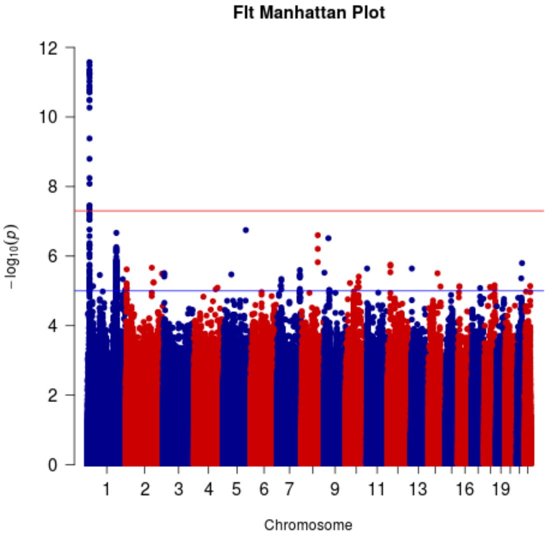

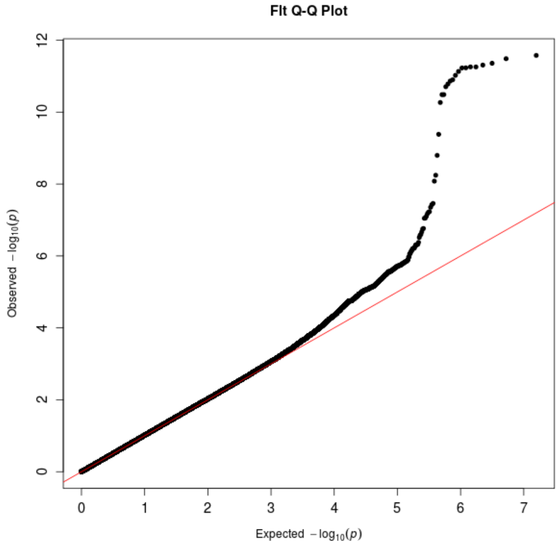

Manhattan and Q-Q plots for 45 quantitative traits (continued)

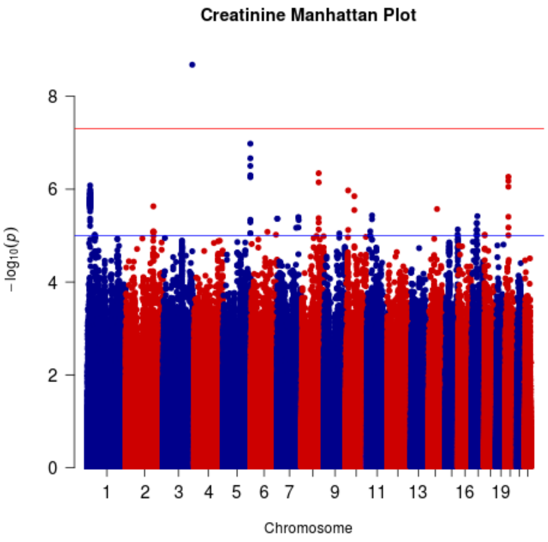

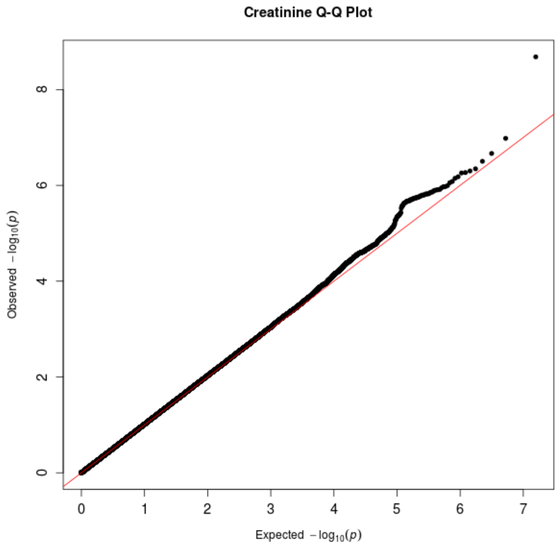

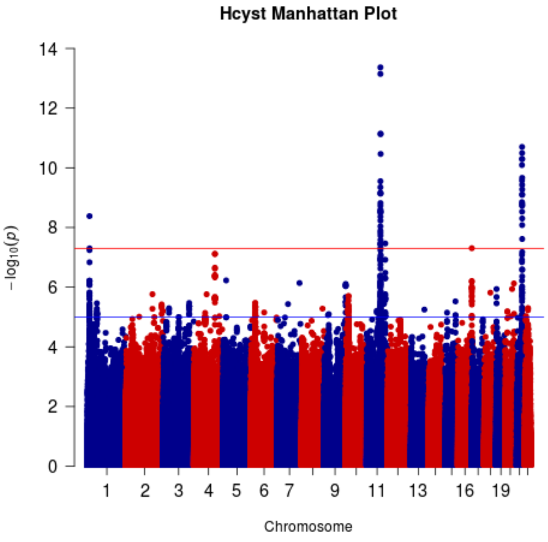

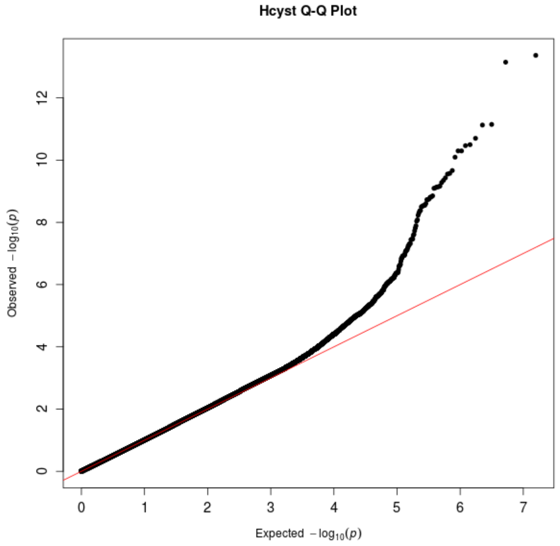

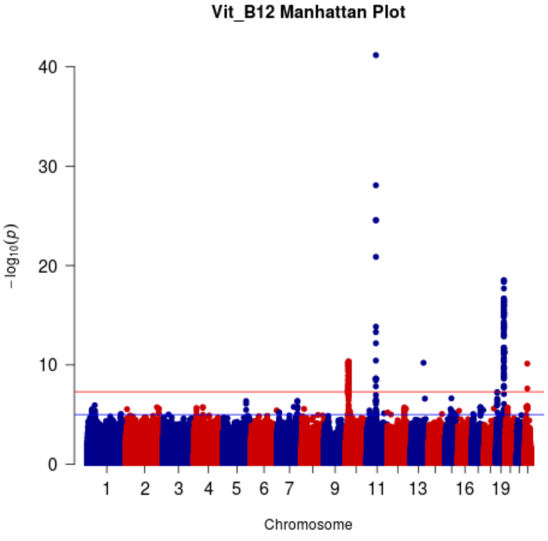

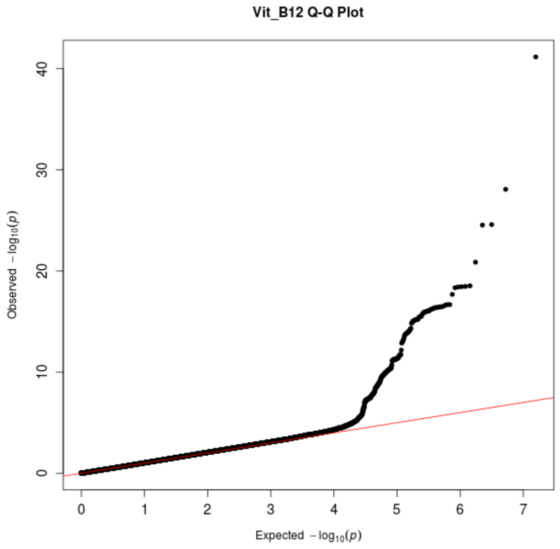

Supplement: Supplementary file 4 — Supplementary Data 2 [file 41467_2021_21381_MOESM4_ESM.pdf]
