# Supplementary material for: Whole genome sequencing in the Middle Eastern Qatari population identifies genetic associations with 45 clinically relevant traits
Source: Nat Commun. 2021 Feb 23;12:1250. doi: 10.1038/s41467-021-21381-3 (PMC7902658; doi:10.1038/s41467-021-21381-3)
Supplement: Supplementary file 7 — Supplementary Data 5 [file 41467_2021_21381_MOESM7_ESM.pdf]

Supplementary Data 5. Colocalization analysis of known loci showing nominal significance for replication. The graphs below (N=180) show the -log10(p) obtained from GWAS analysis in QGP compared to -log(P) obtained from GWAS analysis in BBJ. The P-value for testing the H3 and H4 hypothesis of colocalization are shown at the top of each graph. H3 tests if the locus is associated with the trait in both QGP and BBJ but the association is driven by different variants. H4 tests if the locus is associated with the trait in both QGP and BBJ but the association is driven by the same variants.

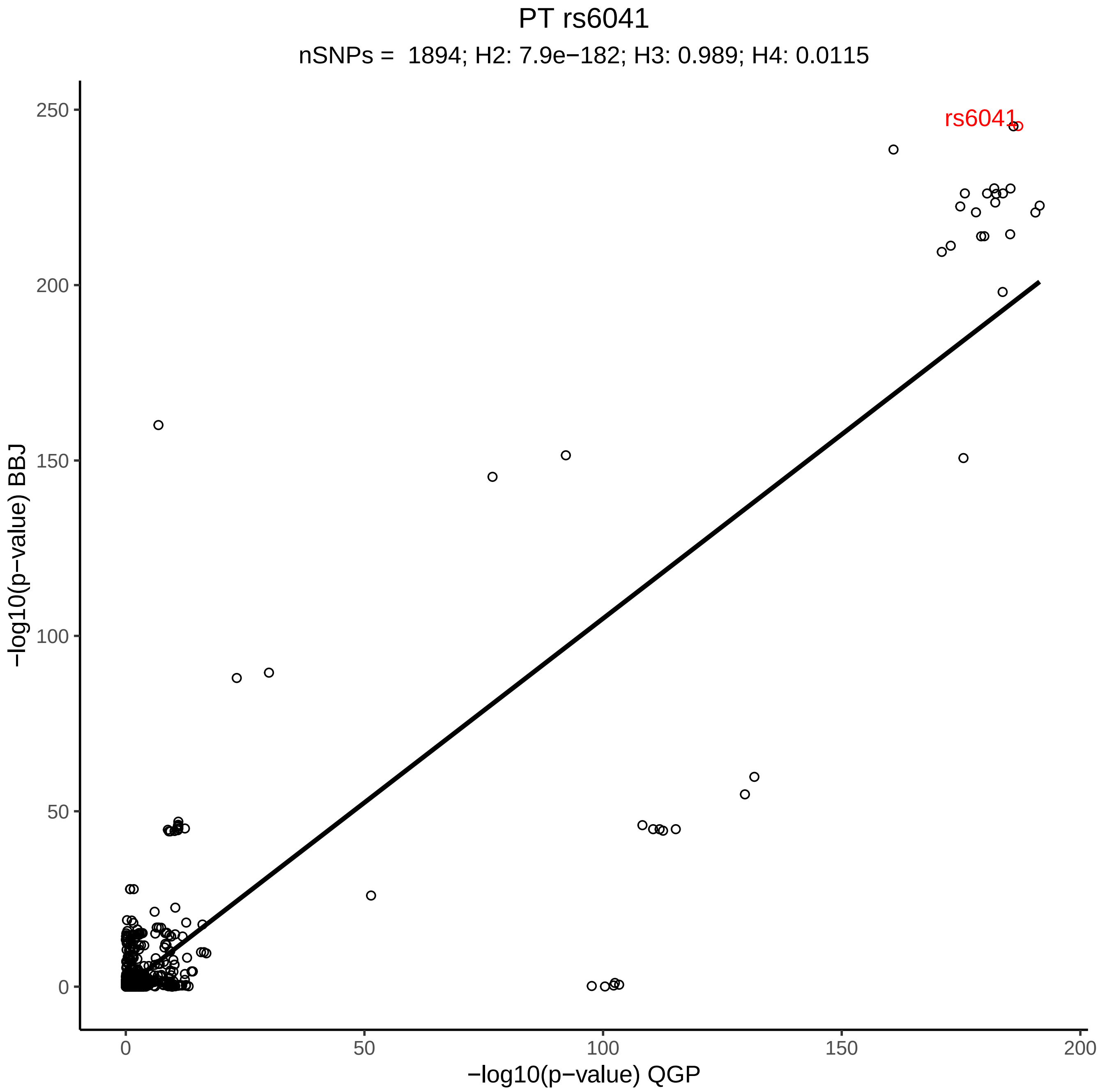

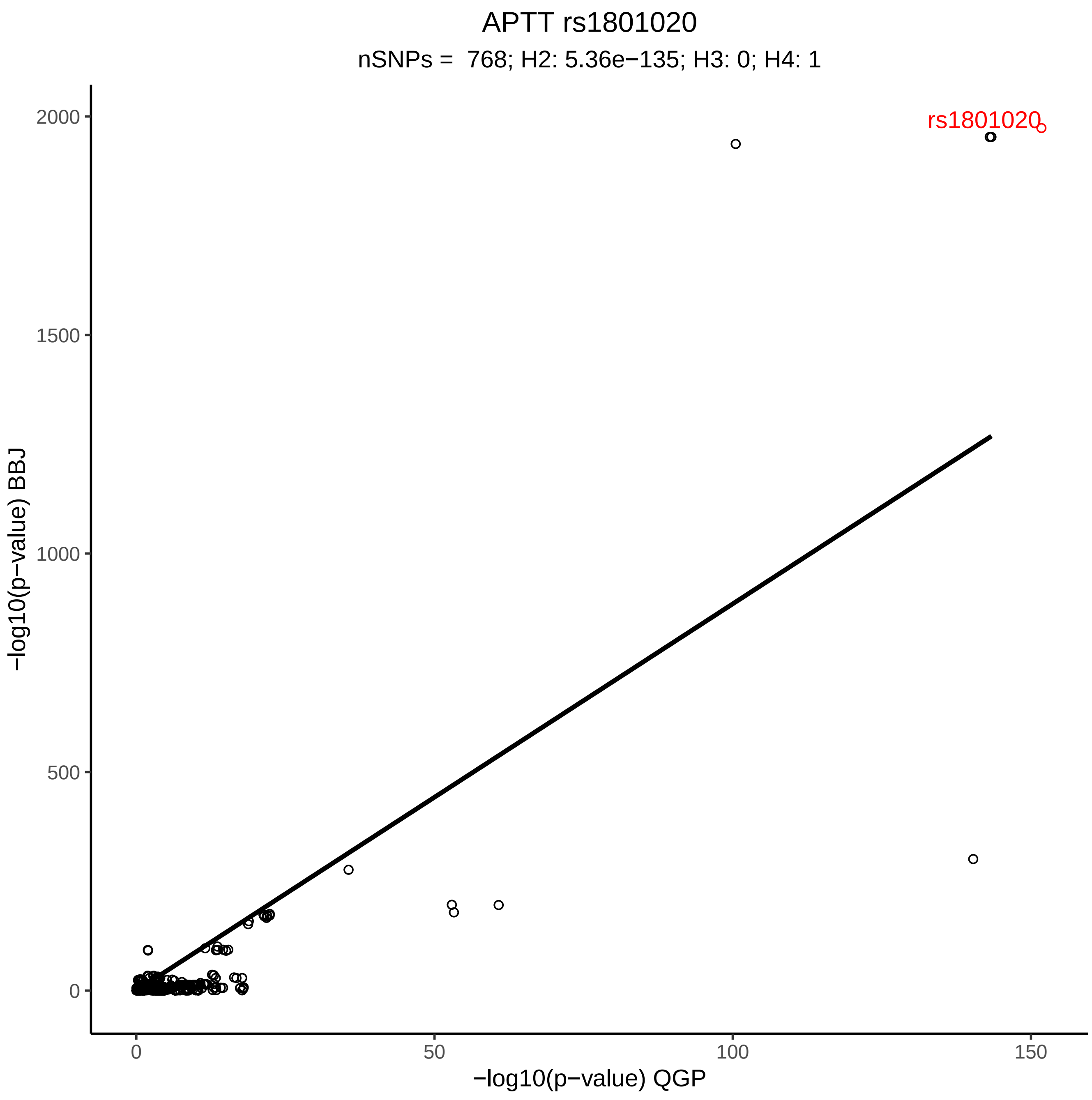
APTT rs1801020
nSNPs = 768; H2: 5.36e−135; H3: 0; H4: 1
rs1801020
2000
1500
1000
500
0
−log10(p−value) BBJ
0
50
100
150
−log10(p−value) QGP

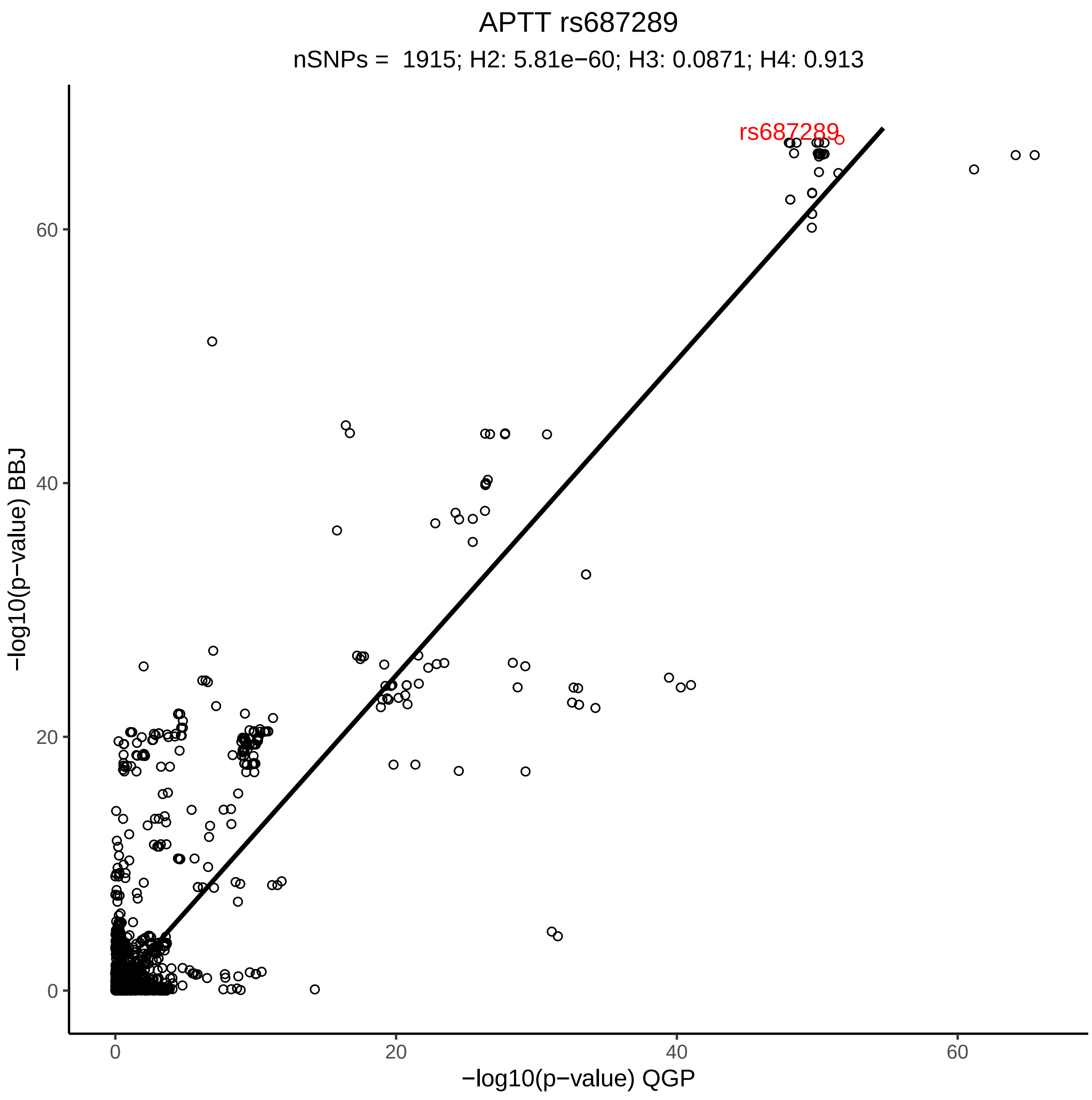

APTT rs687289
nSNPs = 1915; H2: 5.81e−60; H3: 0.0871; H4: 0.913
rs687289
−log10(p−value) BBJ
−log10(p−value) QGP
0
20
40
60
0
20
40
60

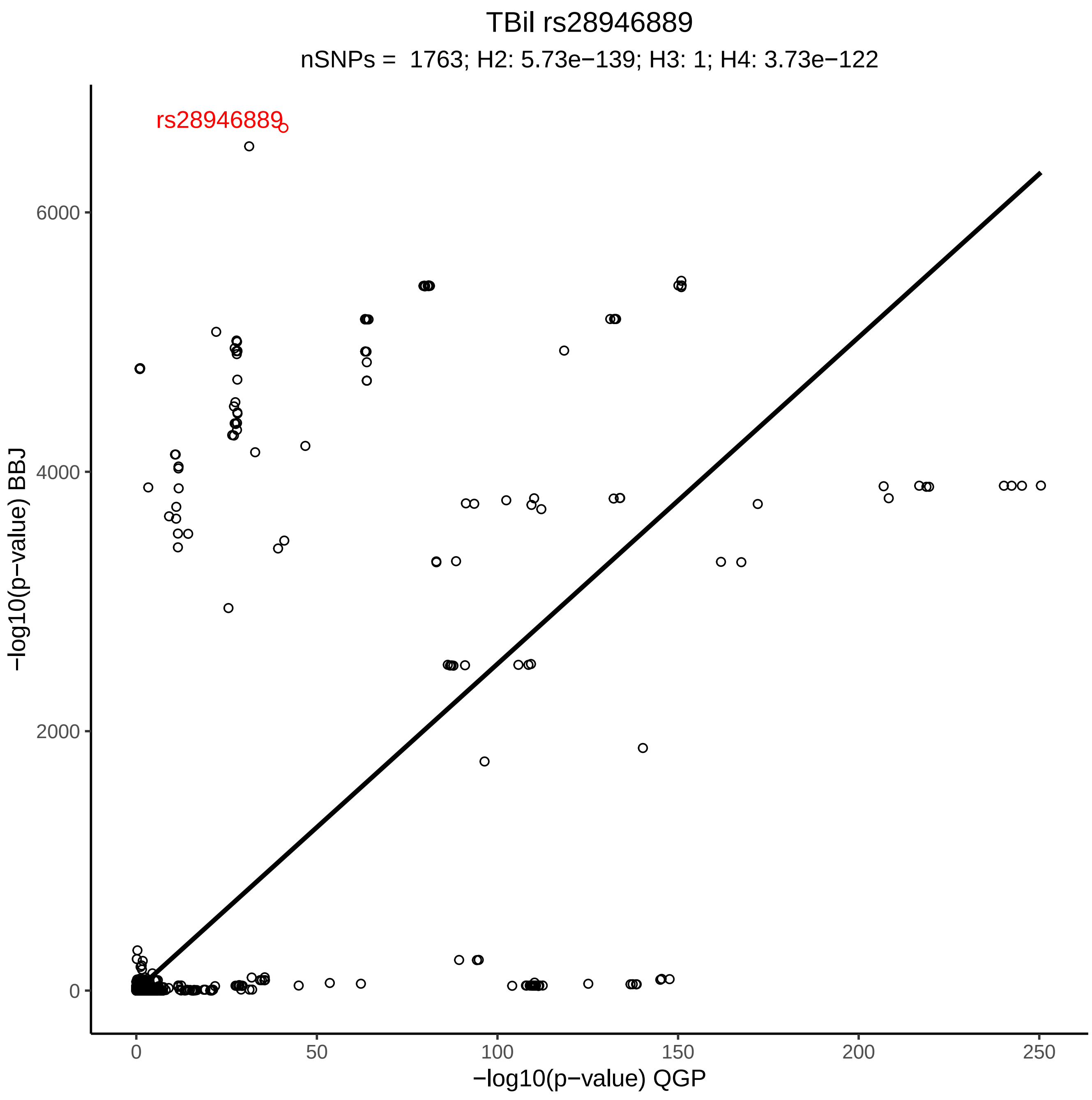
TBil rs28946889
nSNPs = 1763; H2: 5.73e−139; H3: 1; H4: 3.73e−122
rs28946889
6000
4000
2000
0
0
50
100
150
200
250
−log10(p−value) QGP
−log10(p−value) BBJ

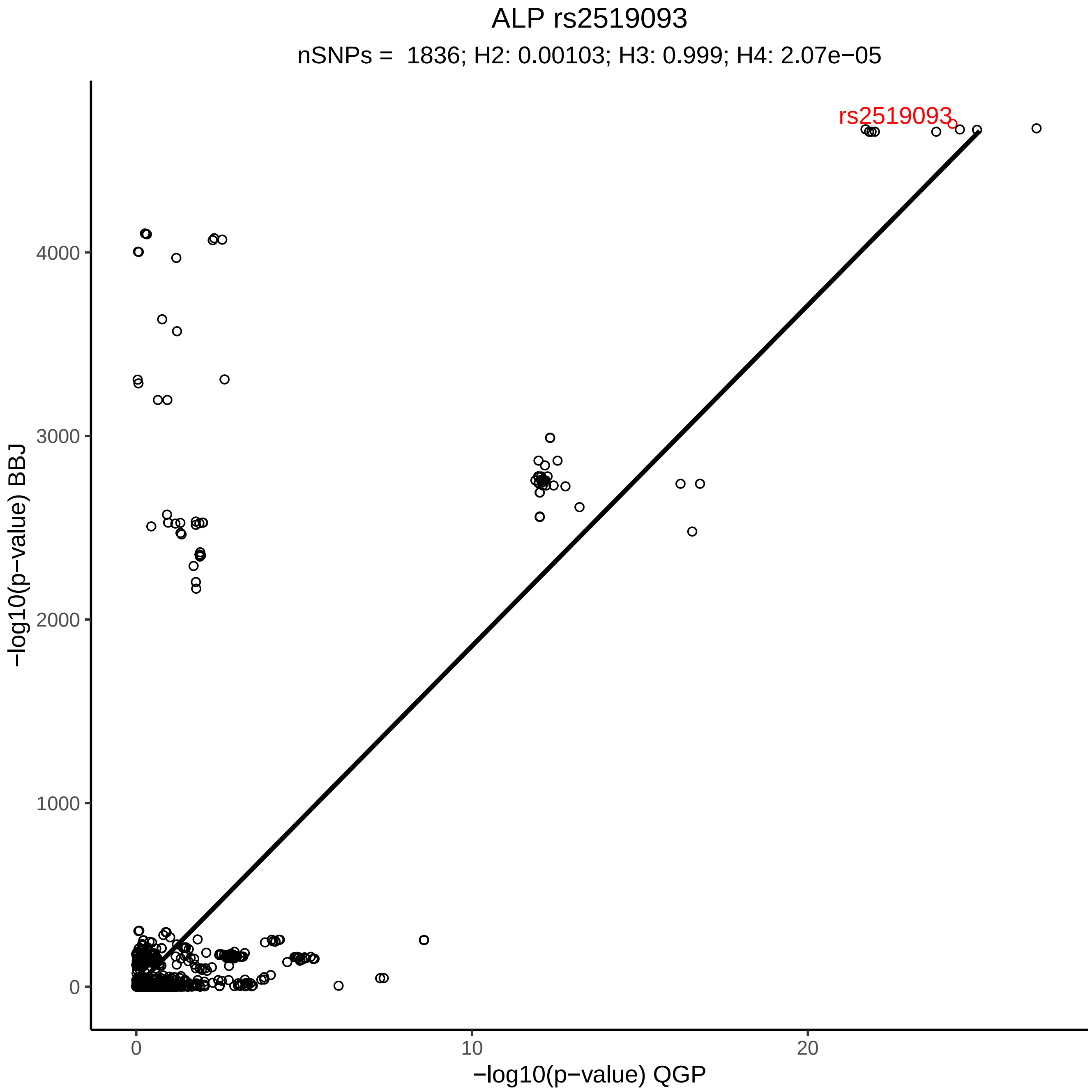
ALP rs2519093
nSNPs = 1836; H2: 0.00103; H3: 0.999; H4: 2.07e−05
rs2519093
4000
3000
2000
1000
0
−log10(p−value) BBJ
0
10
20
−log10(p−value) QGP

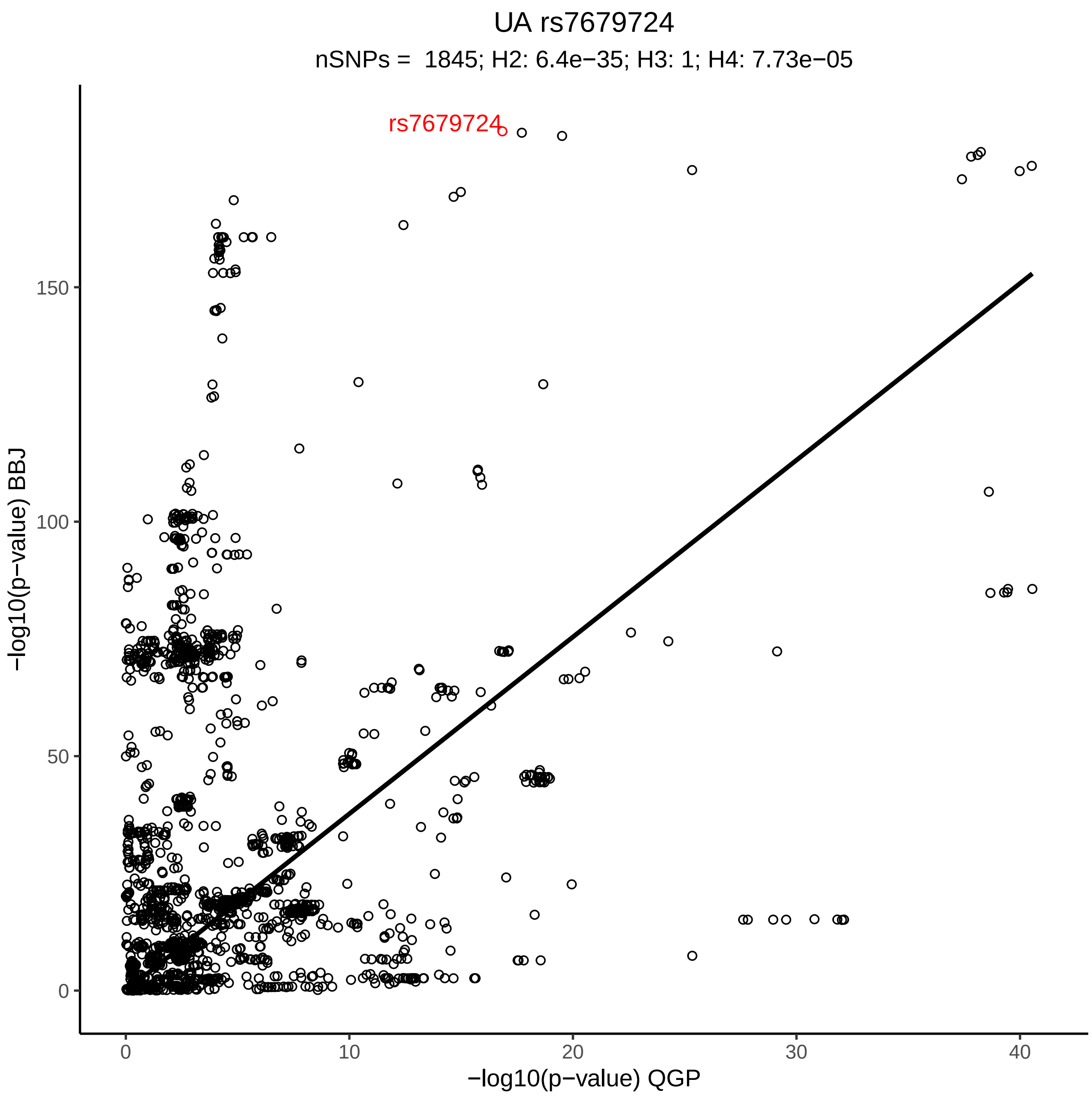
UA rs7679724
nSNPs = 1845; H2: 6.4e−35; H3: 1; H4: 7.73e−05
rs7679724
−log10(p−value) BBJ
−log10(p−value) QGP
0
50
100
150
0
10
20
30
40

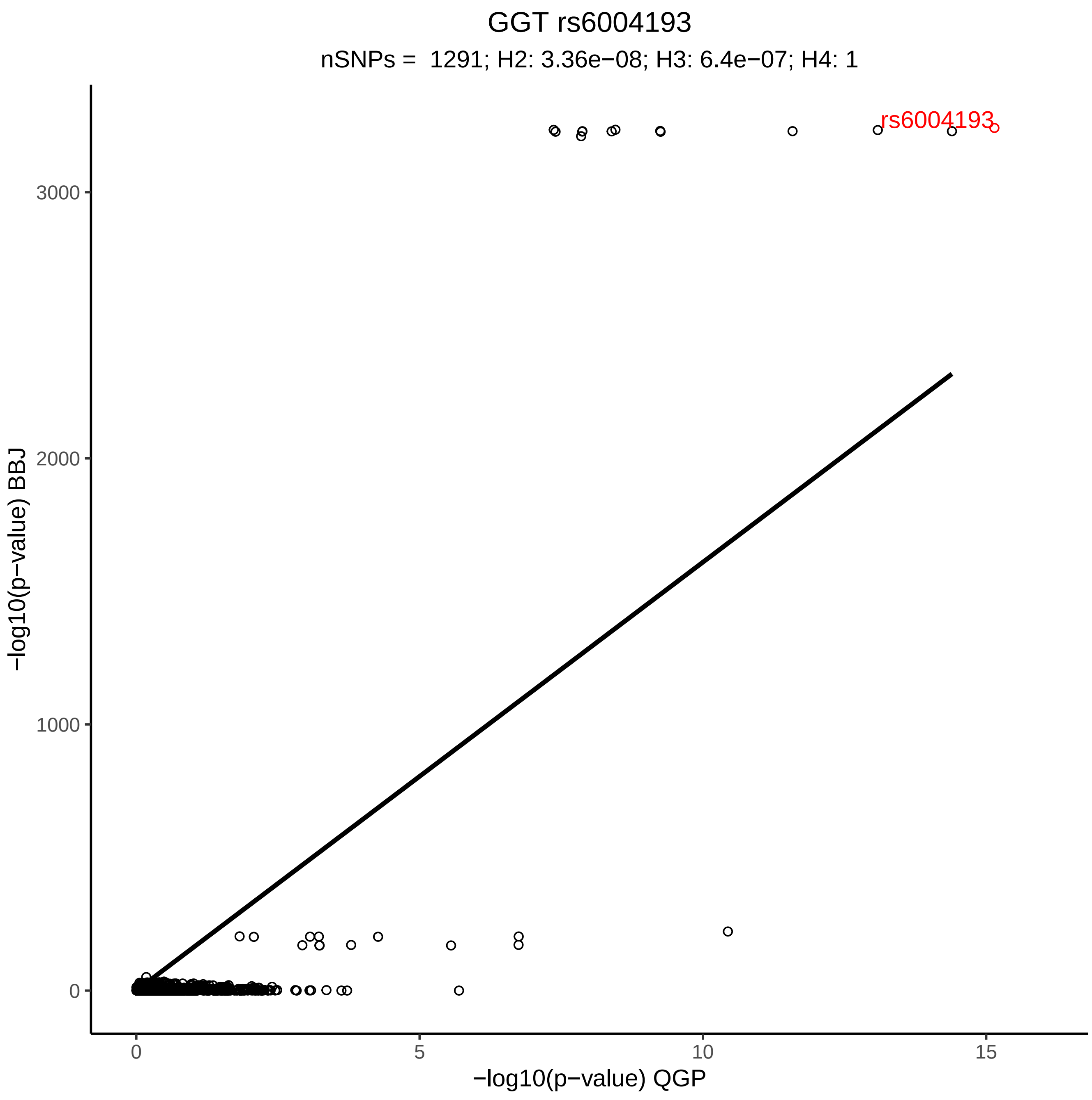
GGT rs6004193
nSNPs = 1291; H2: 3.36e−08; H3: 6.4e−07; H4: 1
rs6004193
3000
2000
1000
0
−log10(p−value) BBJ
0
5
10
15
−log10(p−value) QGP

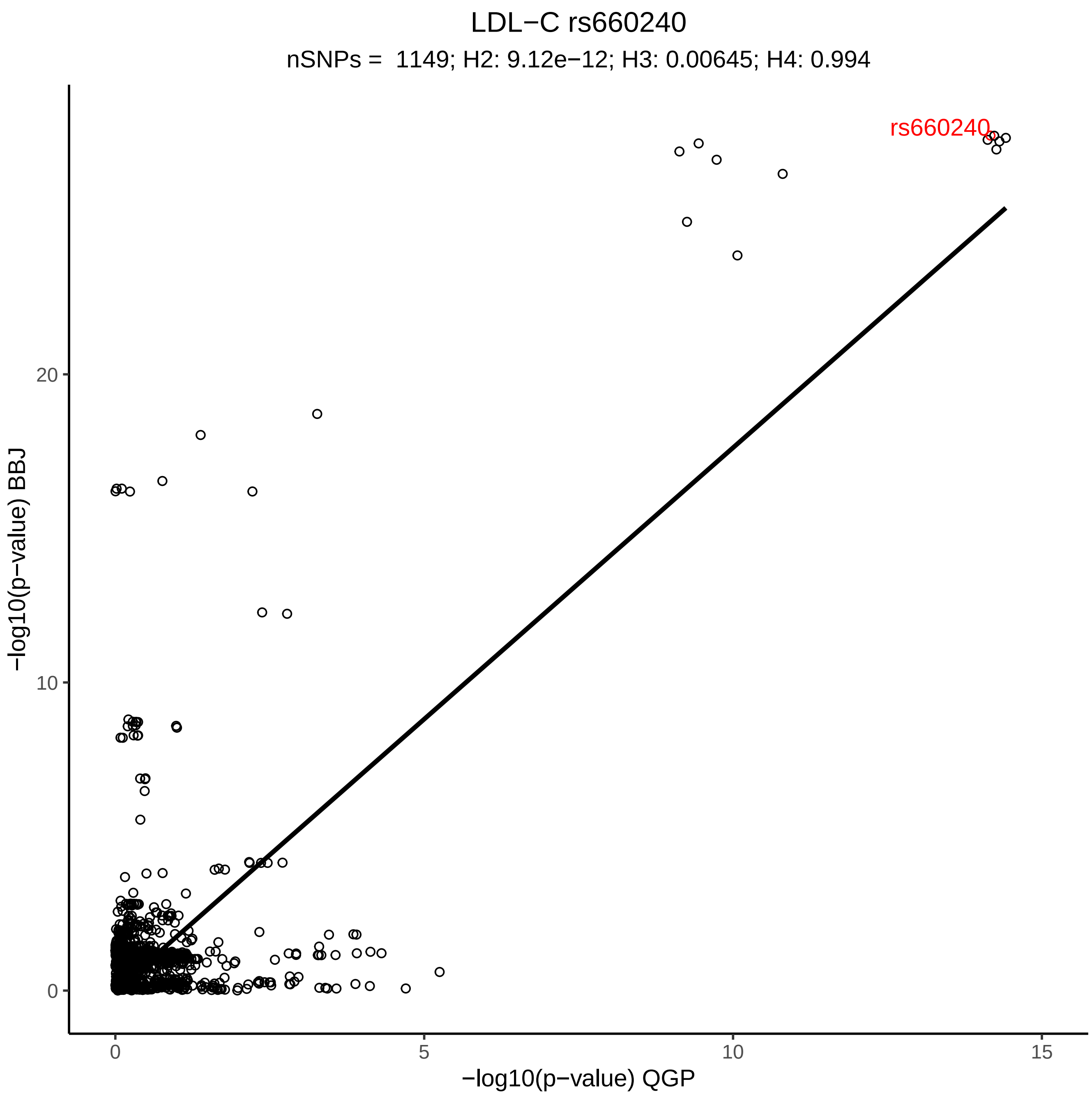
LDL−C rs660240
nSNPs = 1149; H2: 9.12e−12; H3: 0.00645; H4: 0.994
rs660240
−log10(p−value) BBJ
−log10(p−value) QGP
0
10
20
0
5
10
15

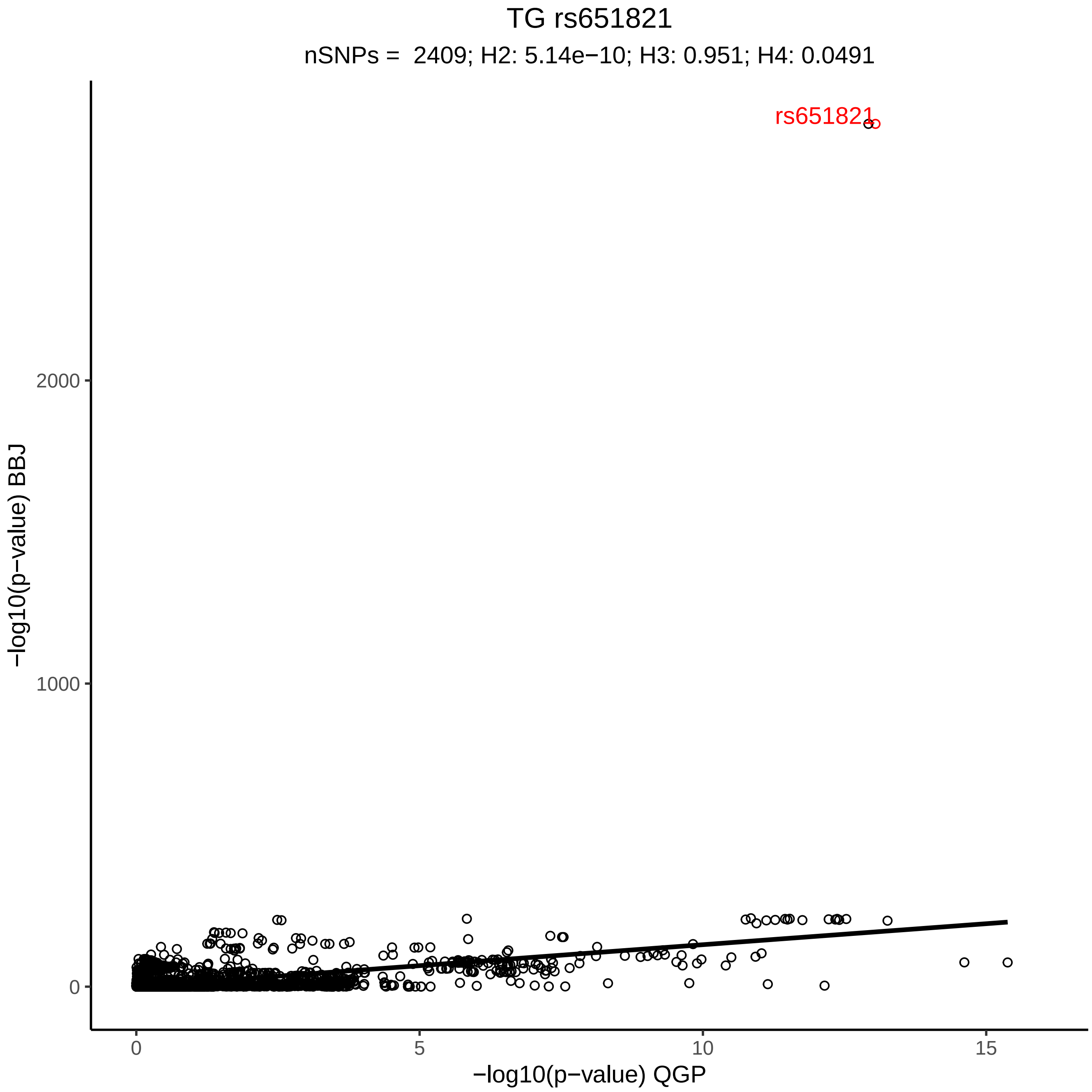
TG rs651821
nSNPs = 2409; H2: 5.14e−10; H3: 0.951; H4: 0.0491
rs651821
2000
1000
0
−log10(p−value) BBJ
0
5
10
15
−log10(p−value) QGP

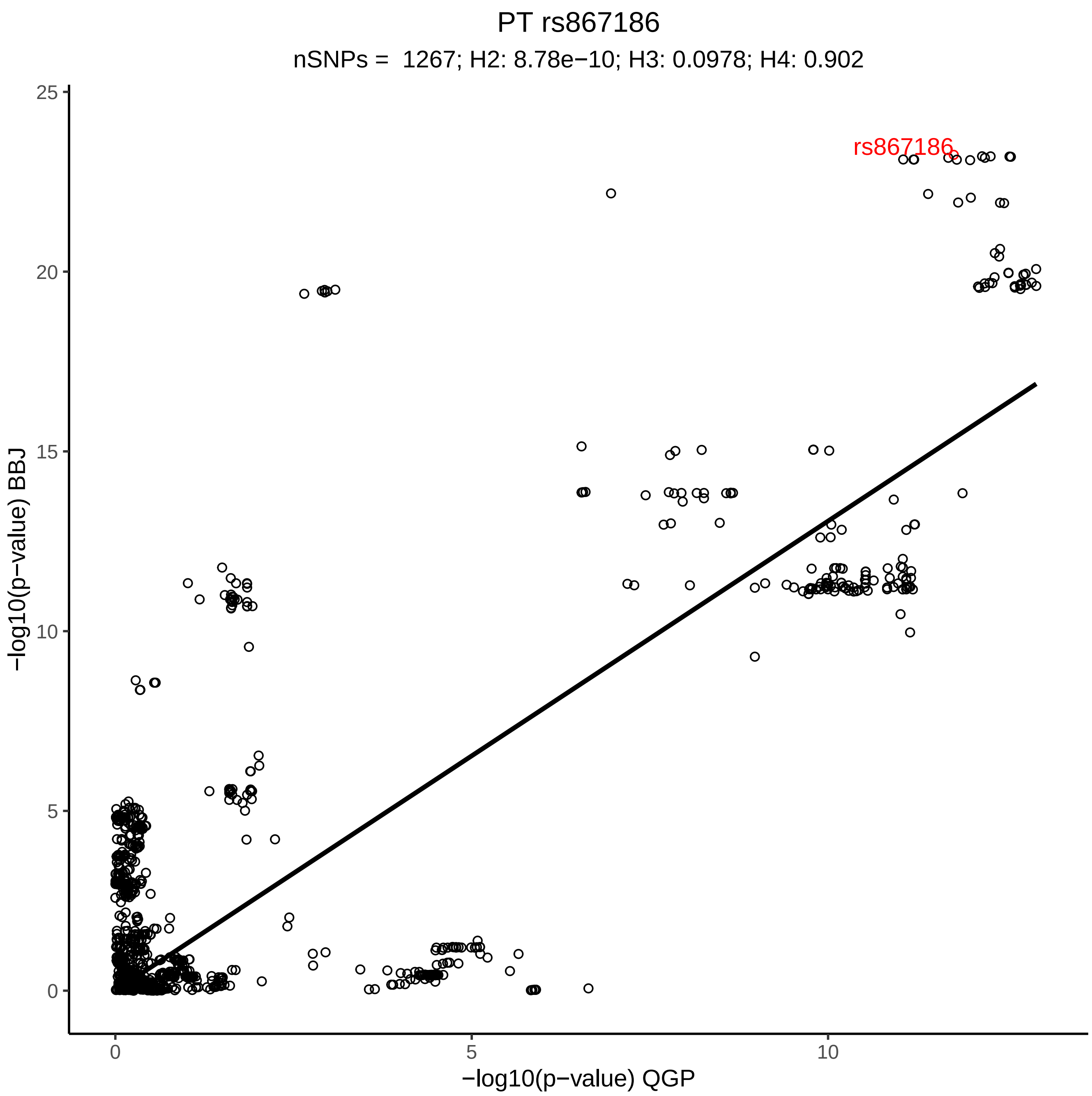

PT rs867186
nSNPs = 1267; H2: 8.78e−10; H3: 0.0978; H4: 0.902
rs867186
25
20
15
10
5
0
0
5
10
−log10(p−value) BBJ
−log10(p−value) QGP

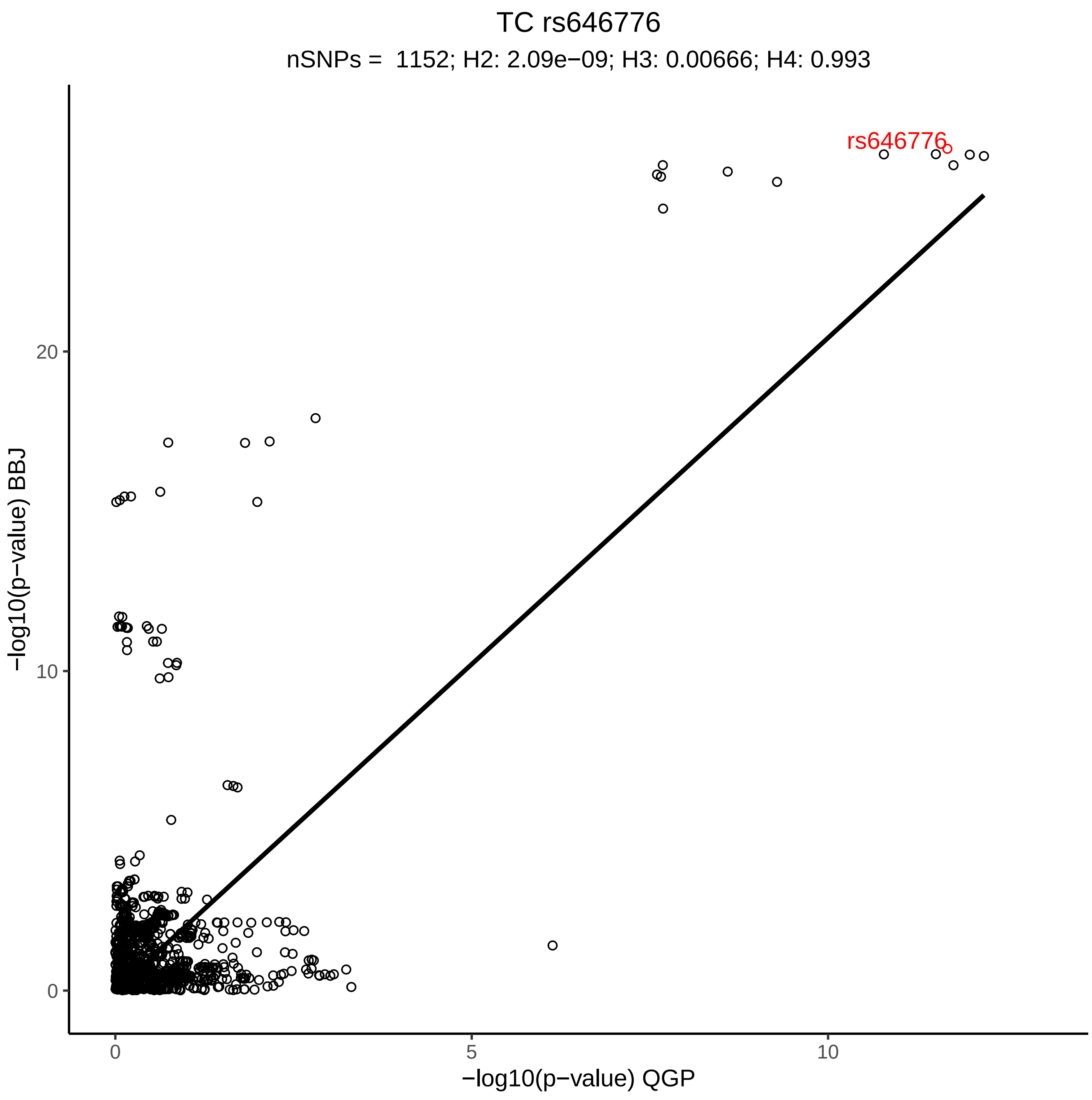
TC rs646776
nSNPs = 1152; H2: 2.09e−09; H3: 0.00666; H4: 0.993
rs646776
−log10(p−value) BBJ
−log10(p−value) QGP
0
10
20
0
5
10

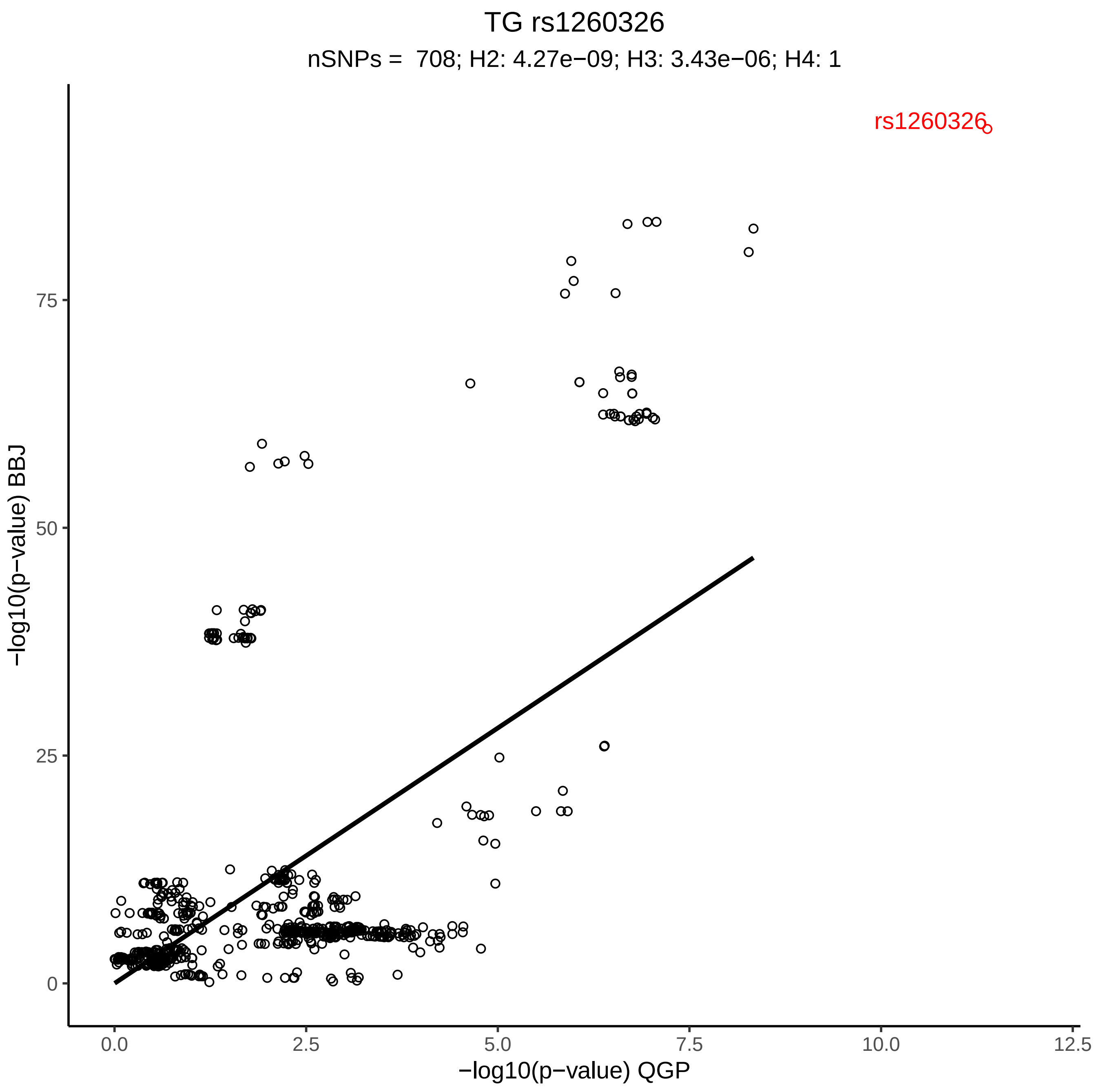

TG rs1260326
nSNPs = 708; H2: 4.27e−09; H3: 3.43e−06; H4: 1
rs1260326
75
50
25
0
−log10(p−value) BBJ
0.0
2.5
5.0
7.5
10.0
12.5
−log10(p−value) QGP

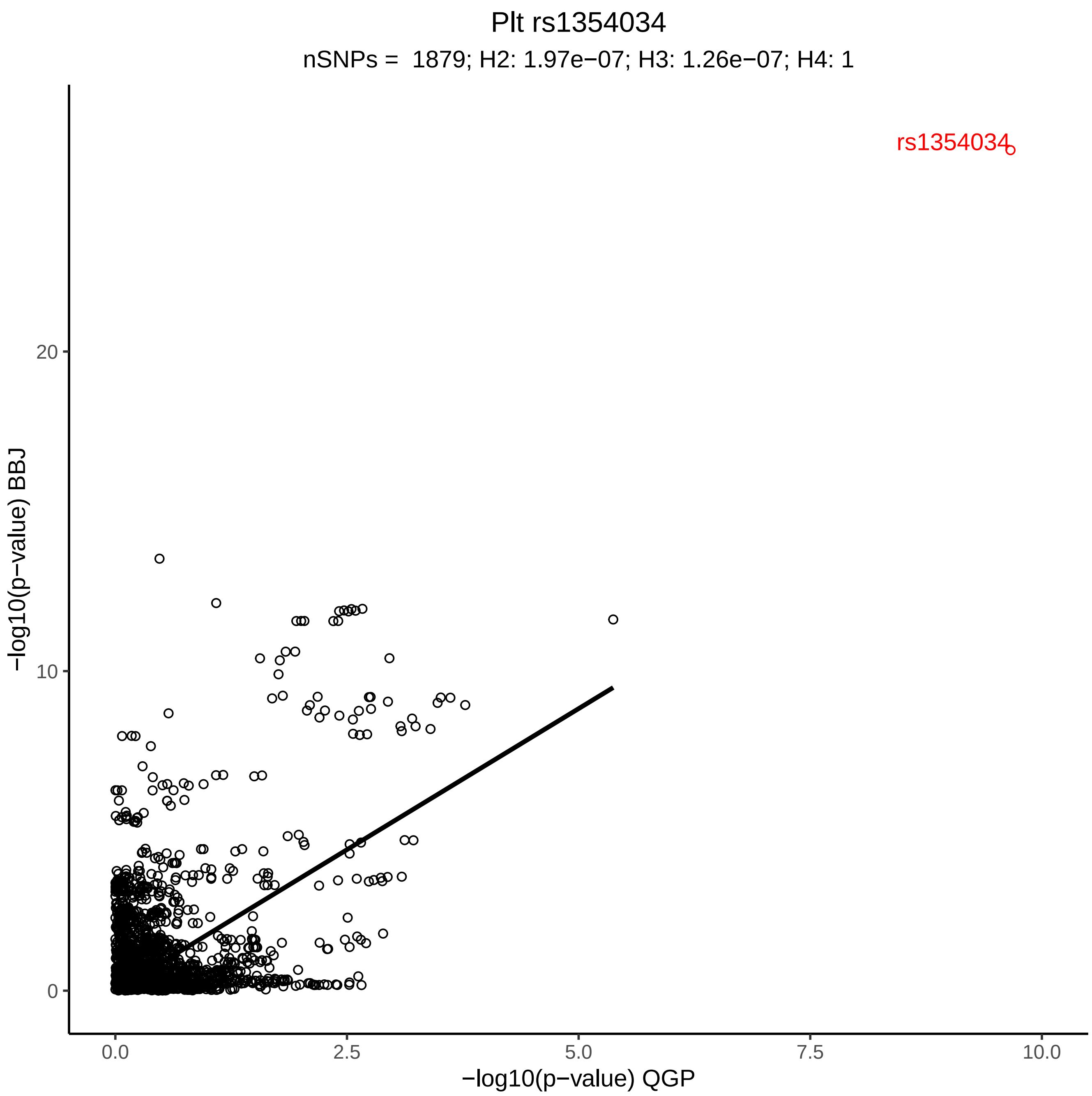
Plt rs1354034
nSNPs = 1879; H2: 1.97e−07; H3: 1.26e−07; H4: 1
rs1354034
20
10
0
−log10(p−value) BBJ
0.0
2.5
5.0
7.5
10.0
−log10(p−value) QGP

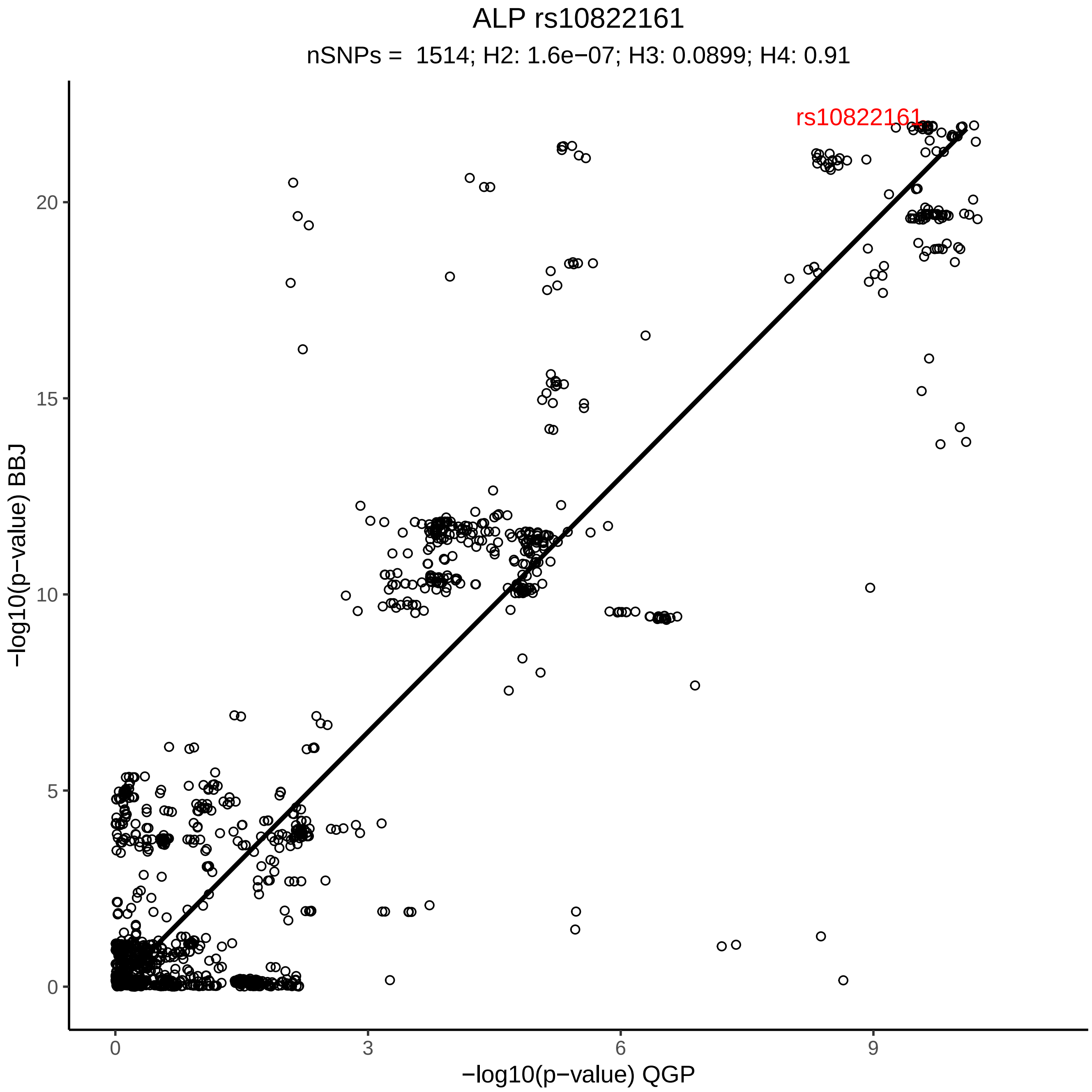
ALP rs10822161
nSNPs = 1514; H2: 1.6e−07; H3: 0.0899; H4: 0.91
rs10822161
−log10(p−value) BBJ
−log10(p−value) QGP
0
5
10
15
20
0
3
6
9

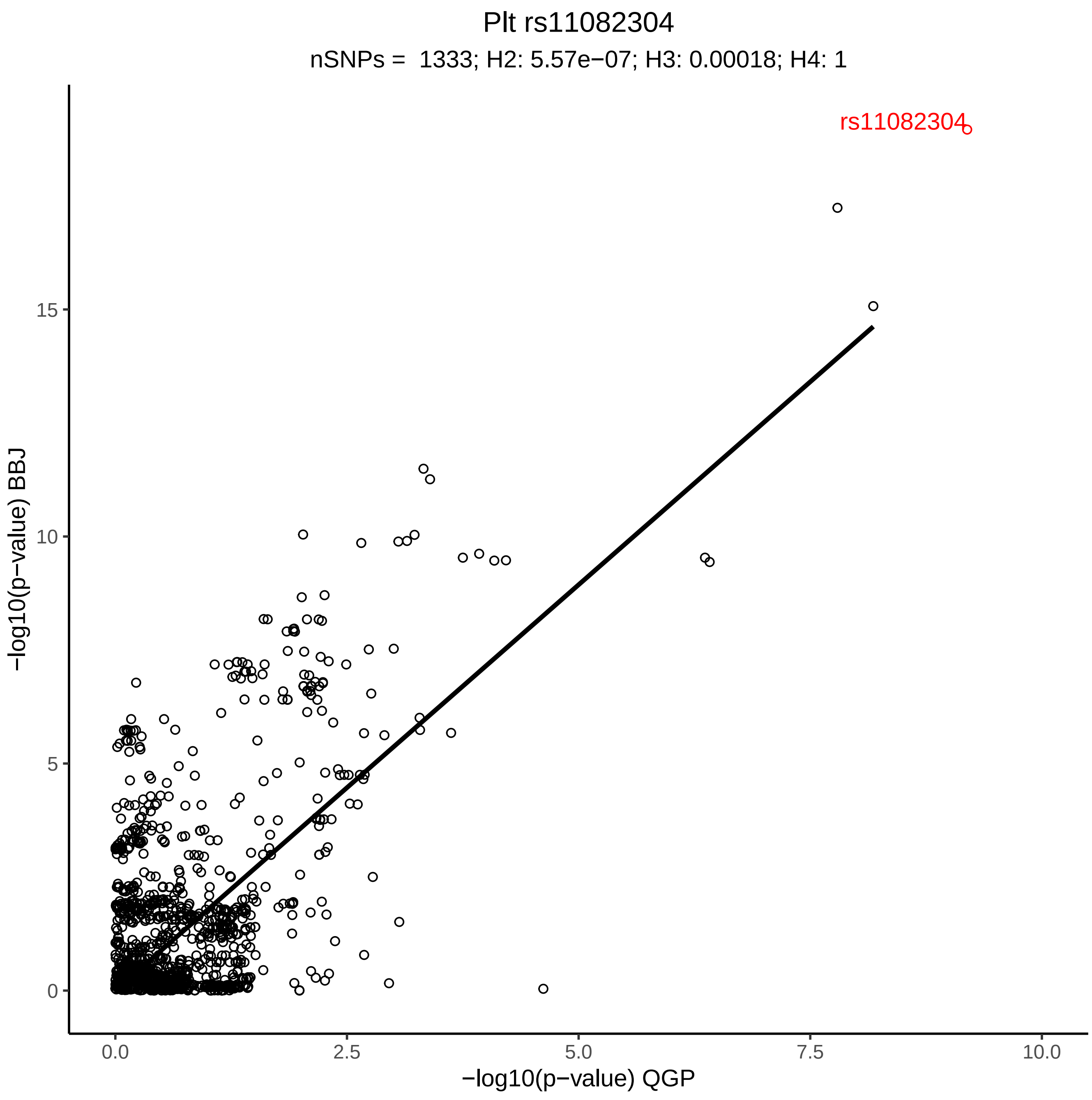
Plt rs11082304
nSNPs = 1333; H2: 5.57e−07; H3: 0.00018; H4: 1
rs11082304
−log10(p−value) BBJ
0
5
10
15
0.0
2.5
5.0
7.5
10.0
−log10(p−value) QGP

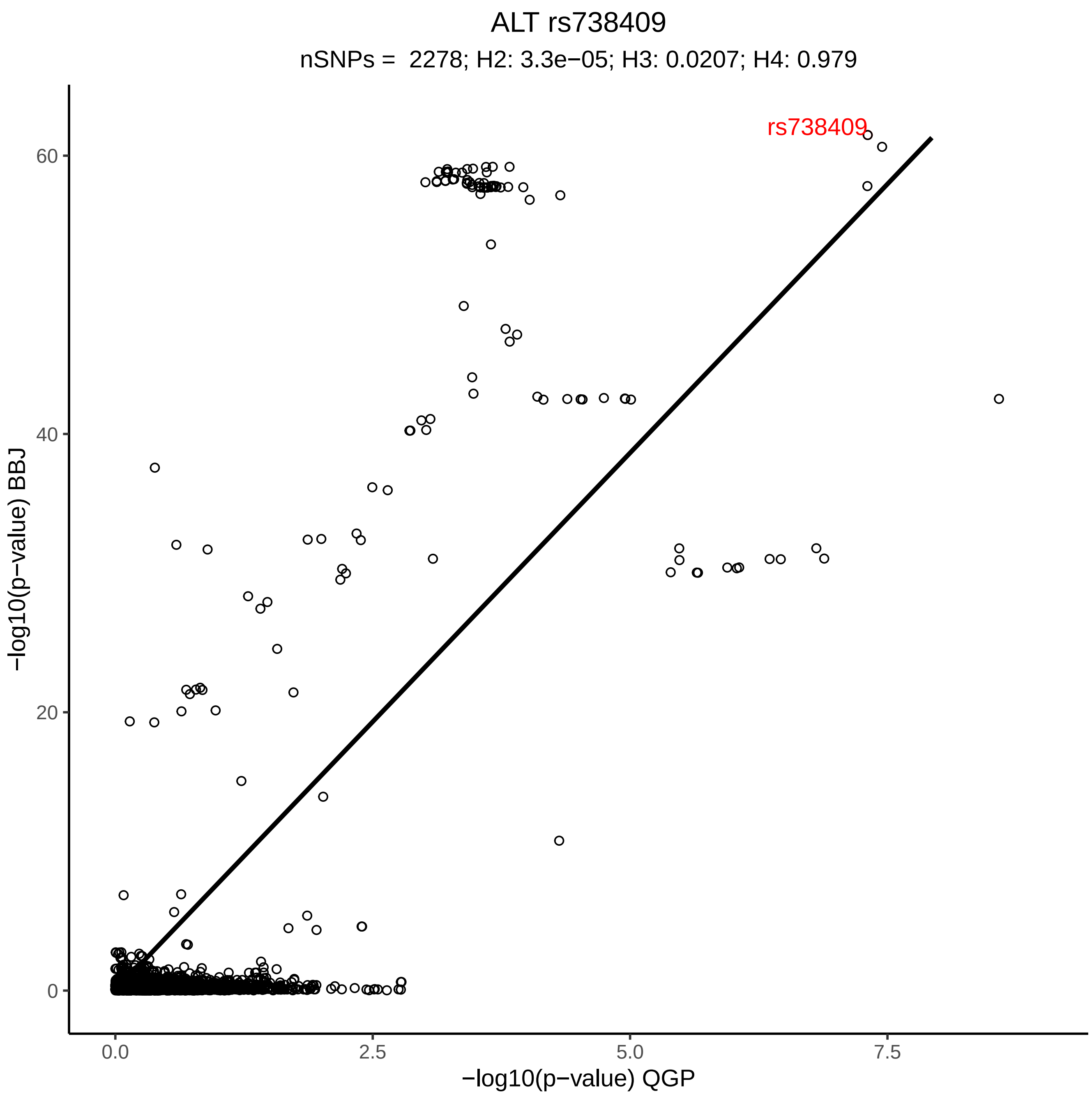
ALT rs738409
nSNPs = 2278; H2: 3.3e−05; H3: 0.0207; H4: 0.979
rs738409
−log10(p−value) BBJ
−log10(p−value) QGP
0
20
40
60
0.0
2.5
5.0
7.5

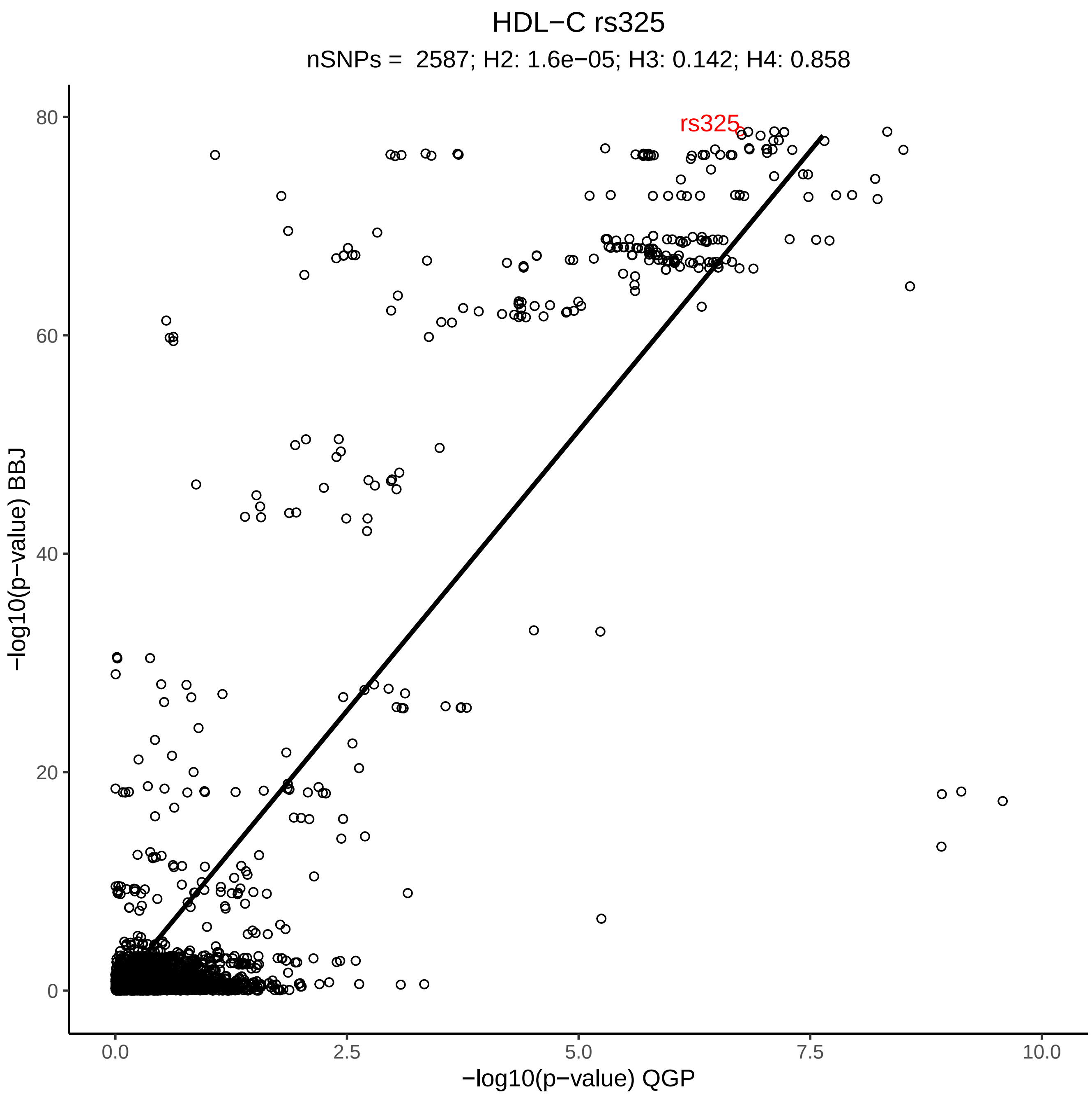

HDL−C rs325
nSNPs = 2587; H2: 1.6e−05; H3: 0.142; H4: 0.858
rs325
−log10(p−value) BBJ
−log10(p−value) QGP
80
60
40
20
0
0.0
2.5
5.0
7.5
10.0

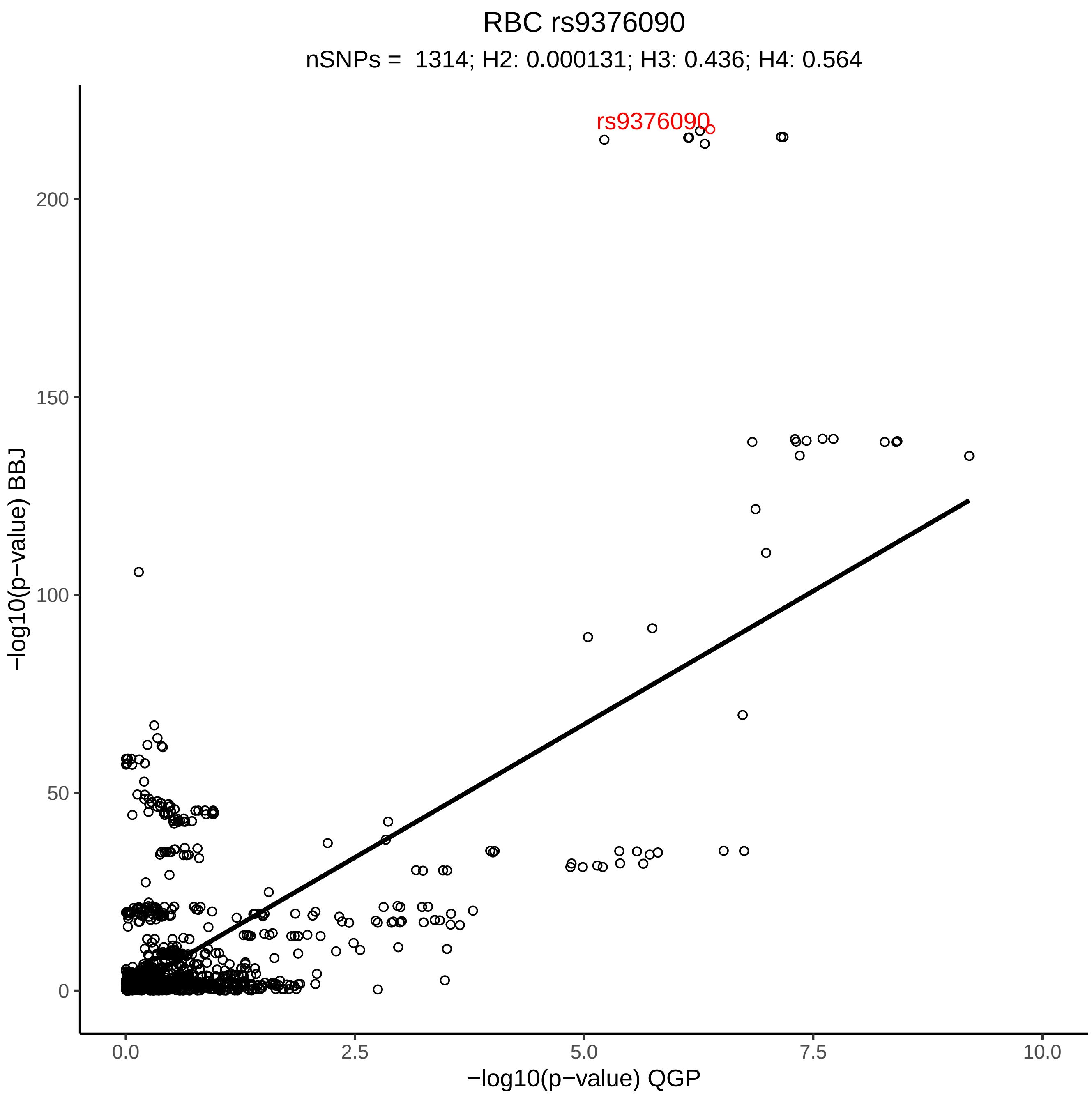

RBC rs9376090
nSNPs = 1314; H2: 0.000131; H3: 0.436; H4: 0.564
rs9376090
−log10(p−value) BBJ
0
50
100
150
200
0.0
2.5
5.0
7.5
10.0
−log10(p−value) QGP

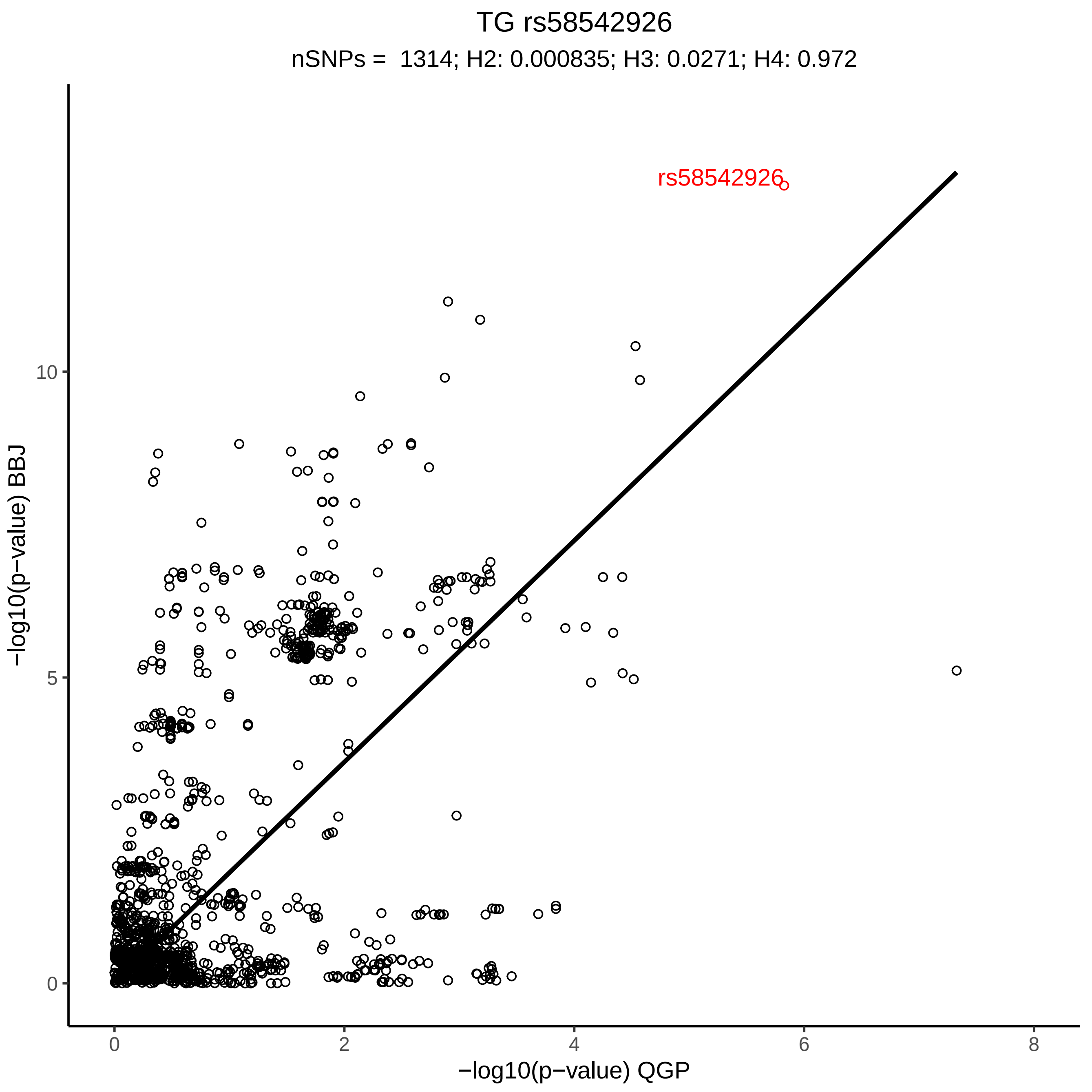

TG rs58542926
nSNPs = 1314; H2: 0.000835; H3: 0.0271; H4: 0.972
rs58542926
−log10(p−value) BBJ
−log10(p−value) QGP
0
5
10
0
2
4
6
8

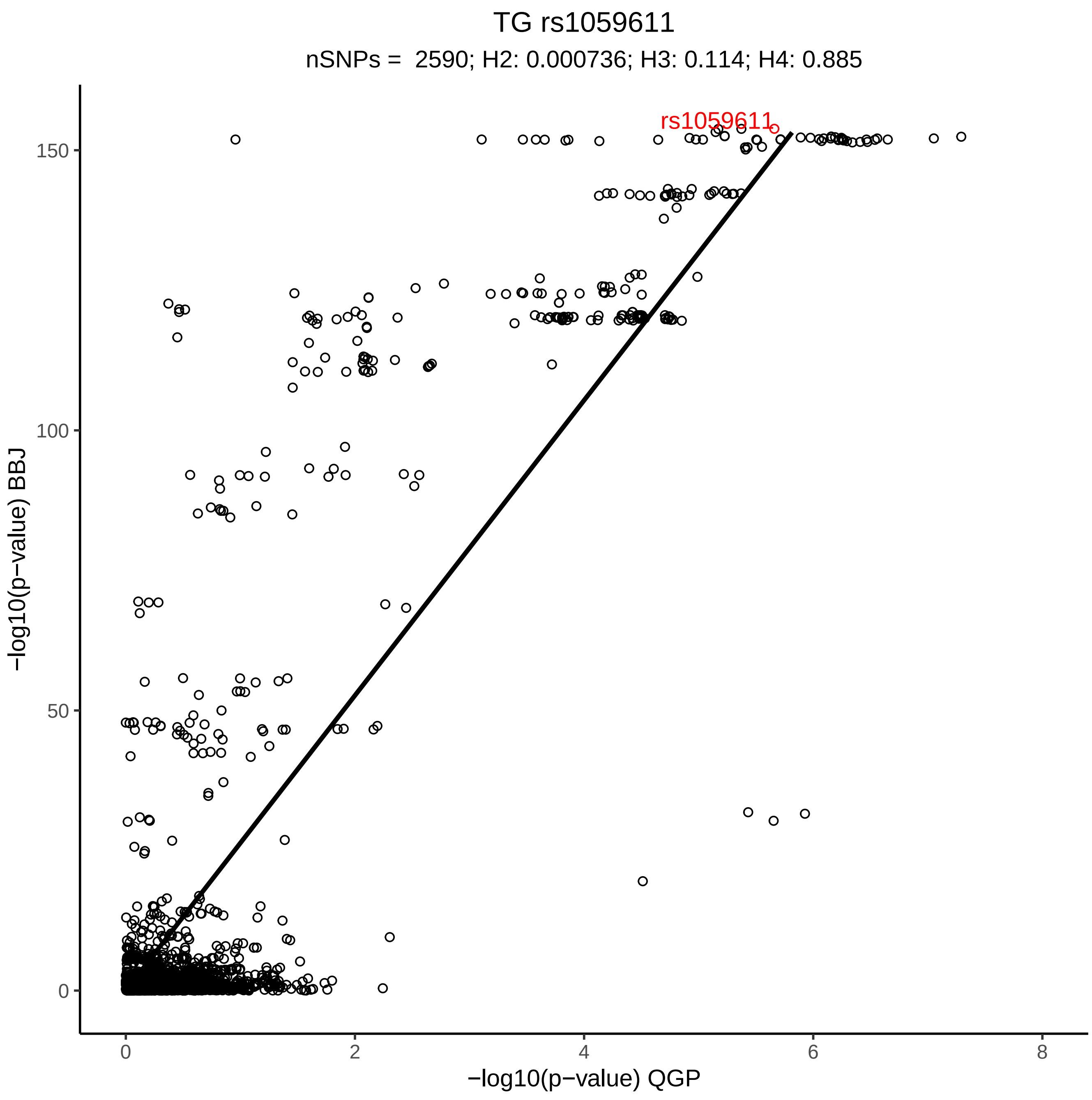

TG rs1059611
nSNPs = 2590; H2: 0.000736; H3: 0.114; H4: 0.885
rs1059611
150
100
50
0
−log10(p−value) BBJ
0
2
4
6
8
−log10(p−value) QGP

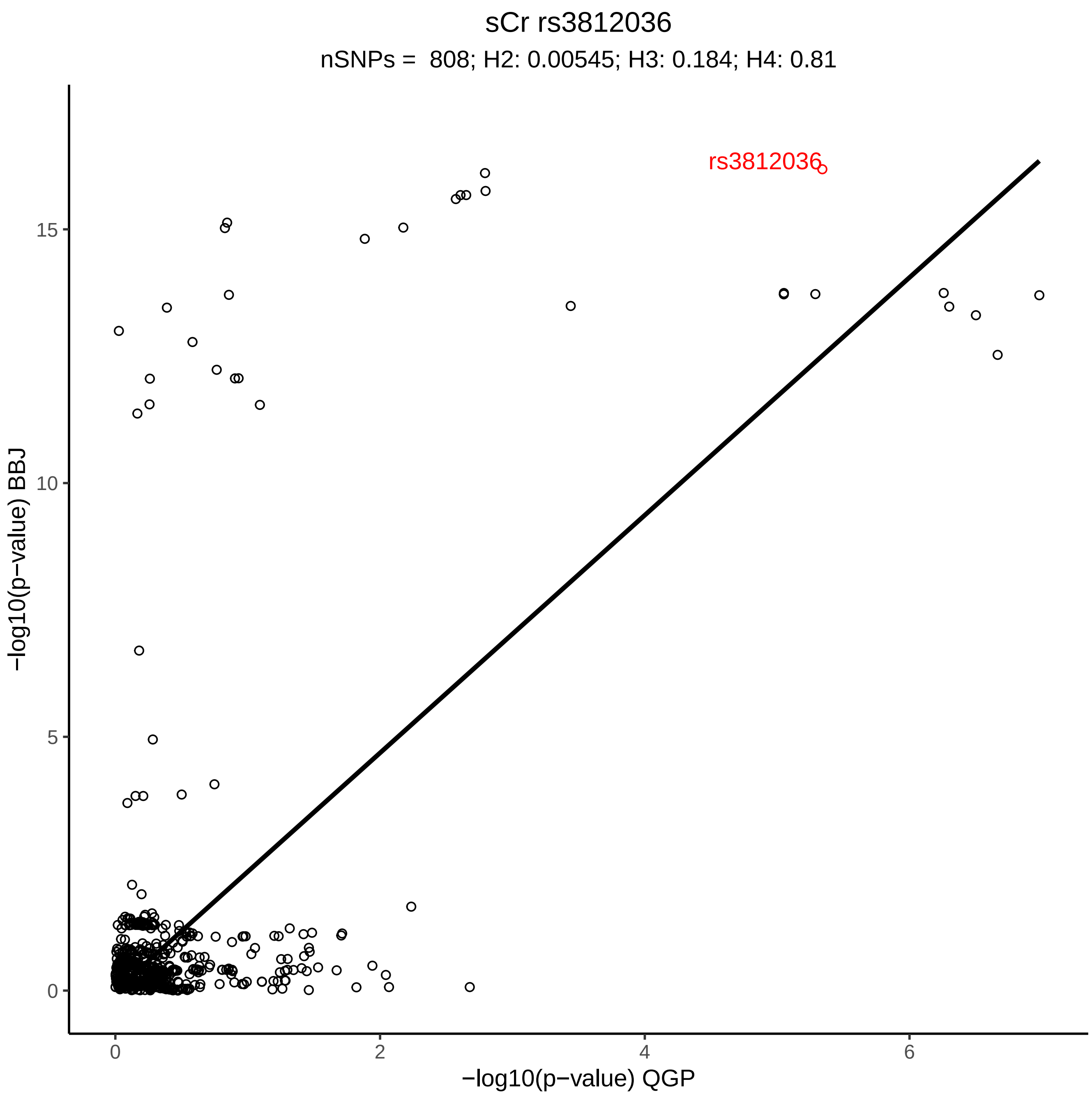
sCr rs3812036
nSNPs = 808; H2: 0.00545; H3: 0.184; H4: 0.81
rs3812036
−log10(p−value) BBJ
−log10(p−value) QGP
0
5
10
15
0
2
4
6

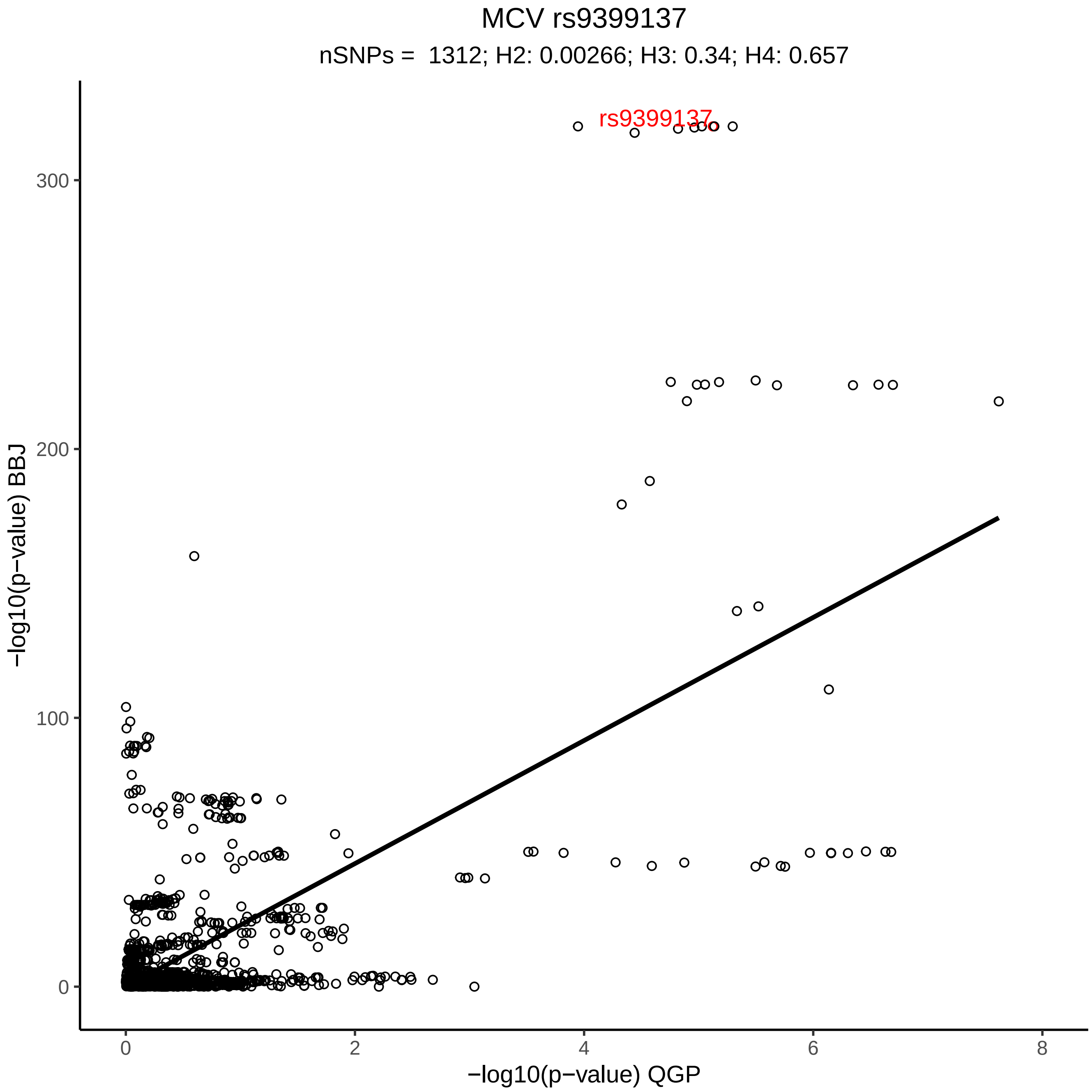
MCV rs9399137
nSNPs = 1312; H2: 0.00266; H3: 0.34; H4: 0.657
rs9399137
300
200
100
0
−log10(p−value) BBJ
0
2
4
6
8
−log10(p−value) QGP

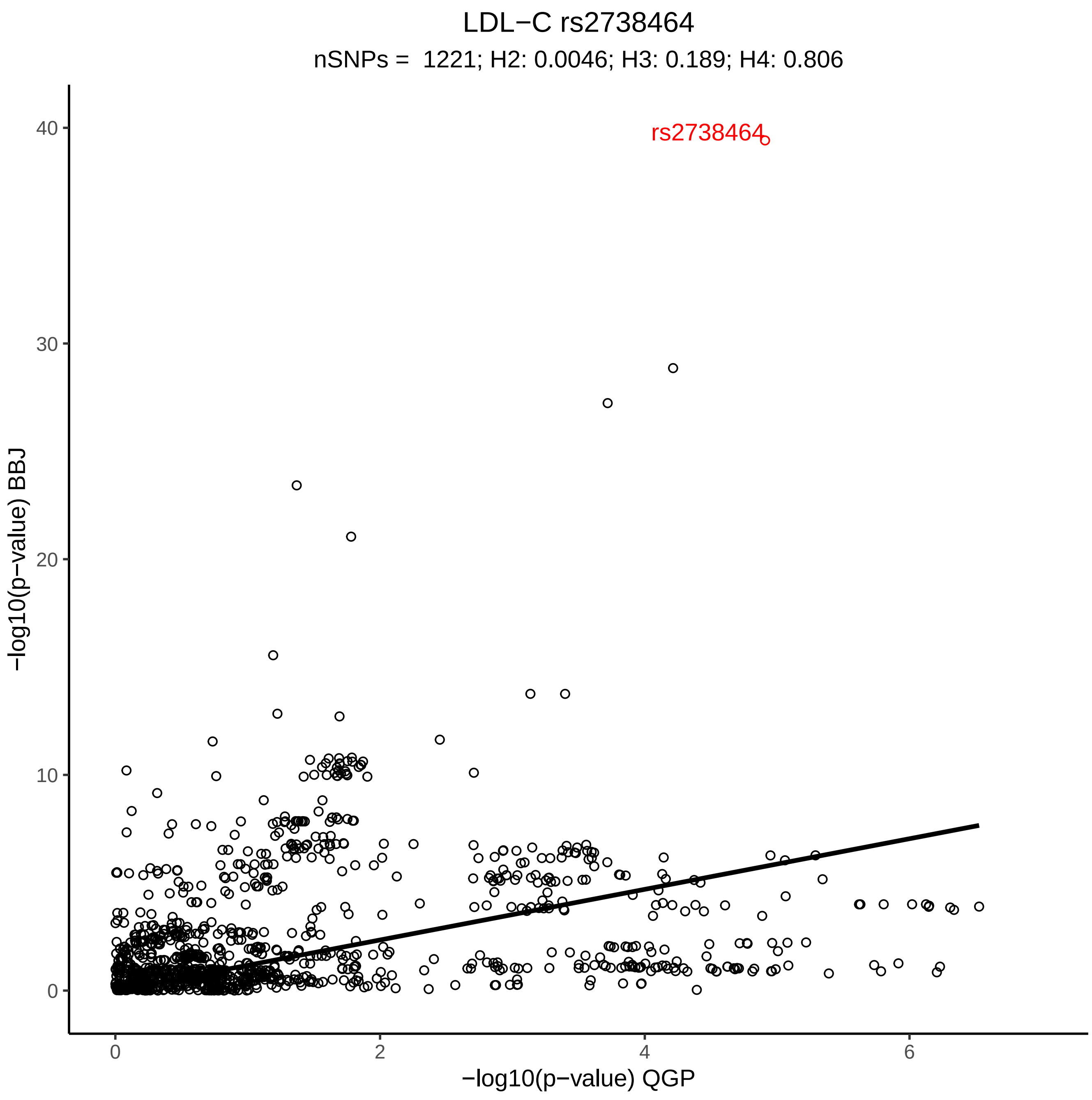

LDL−C rs2738464
nSNPs = 1221; H2: 0.0046; H3: 0.189; H4: 0.806
rs2738464
−log10(p−value) BBJ
−log10(p−value) QGP
0
10
20
30
40
0
2
4
6

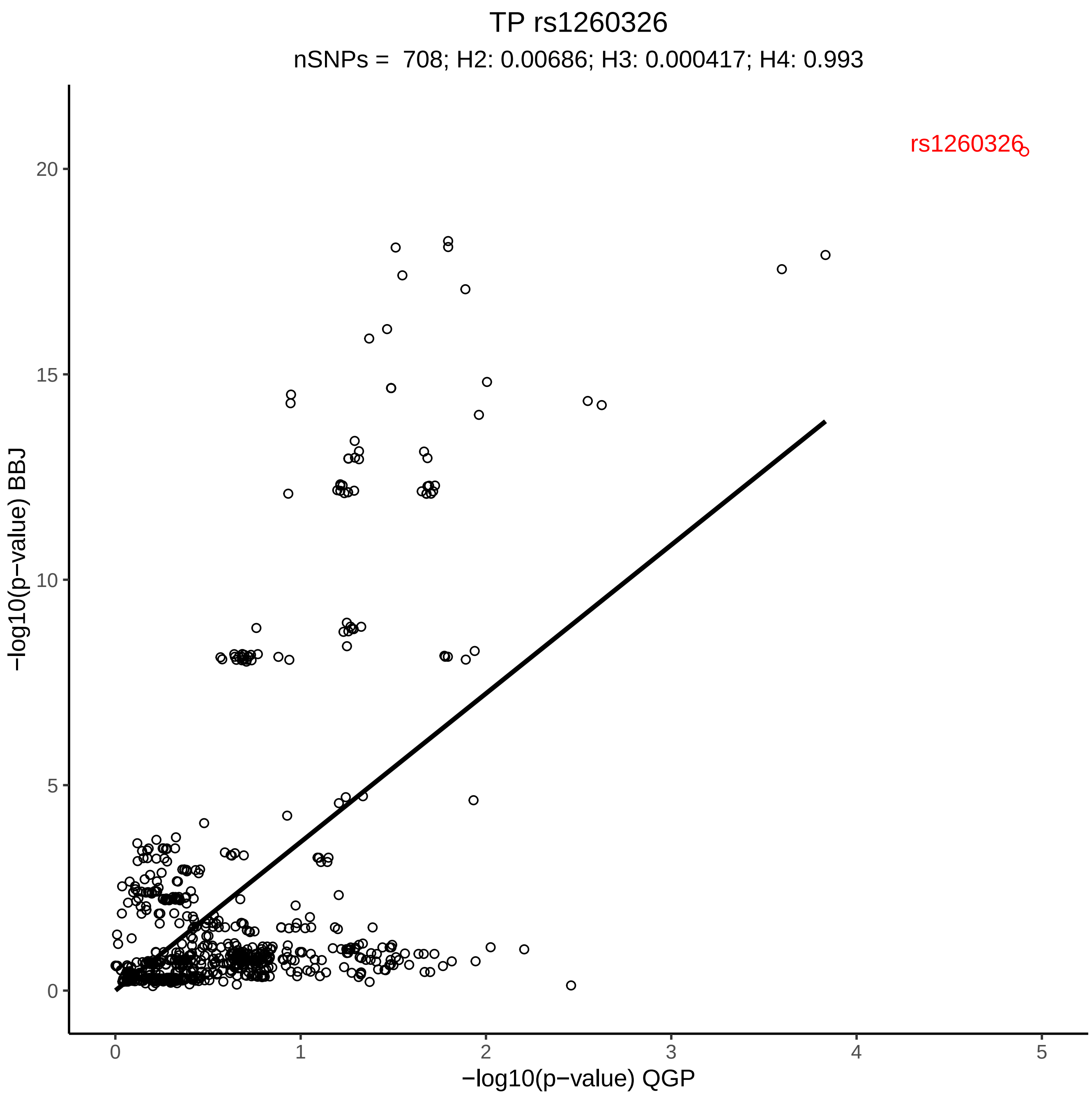
TP rs1260326
nSNPs = 708; H2: 0.00686; H3: 0.000417; H4: 0.993
rs1260326
20
15
10
5
0
−log10(p−value) BBJ
0
1
2
3
4
5
−log10(p−value) QGP

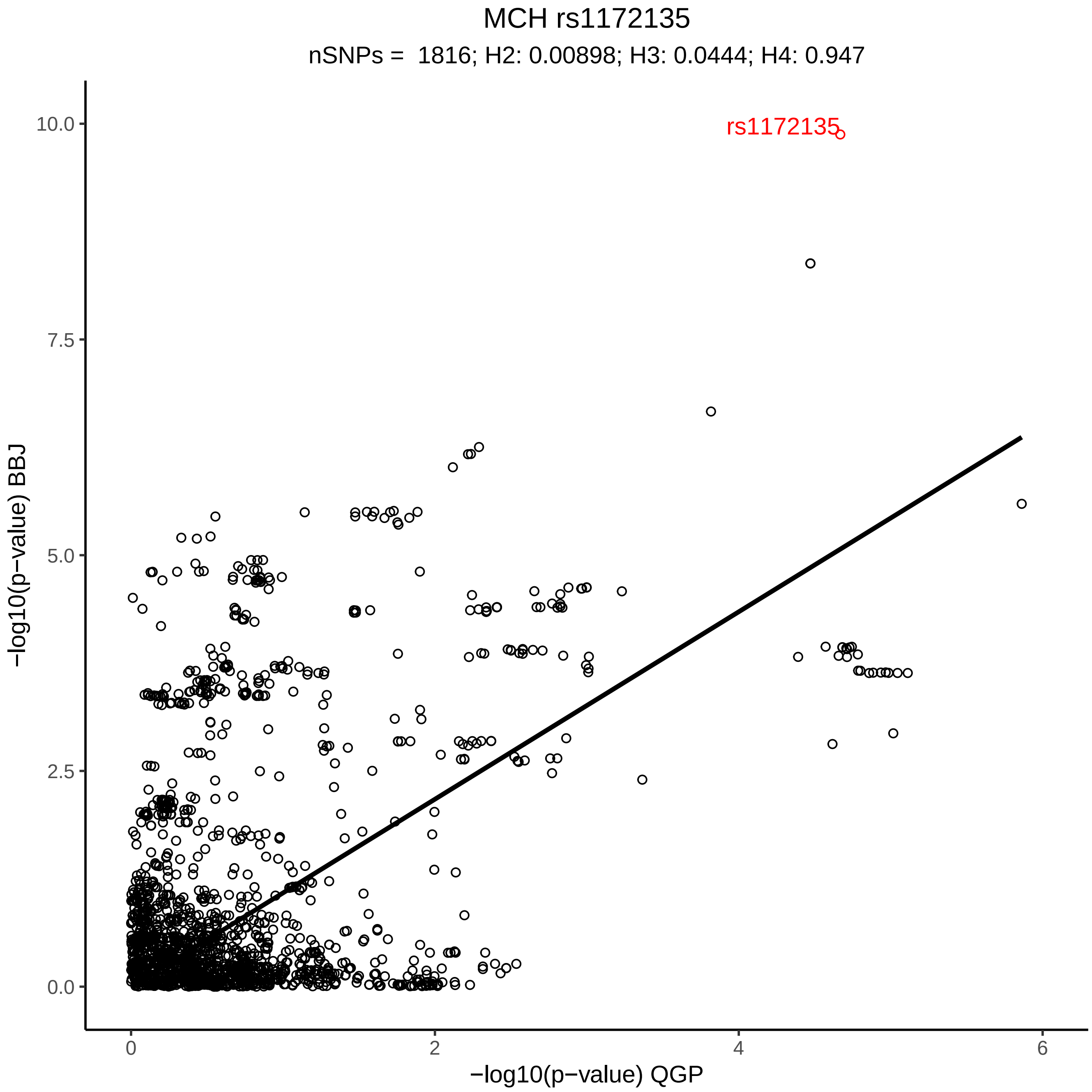

MCH rs1172135
nSNPs = 1816; H2: 0.00898; H3: 0.0444; H4: 0.947
rs1172135
10.0
7.5
5.0
2.5
0.0
−log10(p−value) BBJ
0
2
4
6
−log10(p−value) QGP

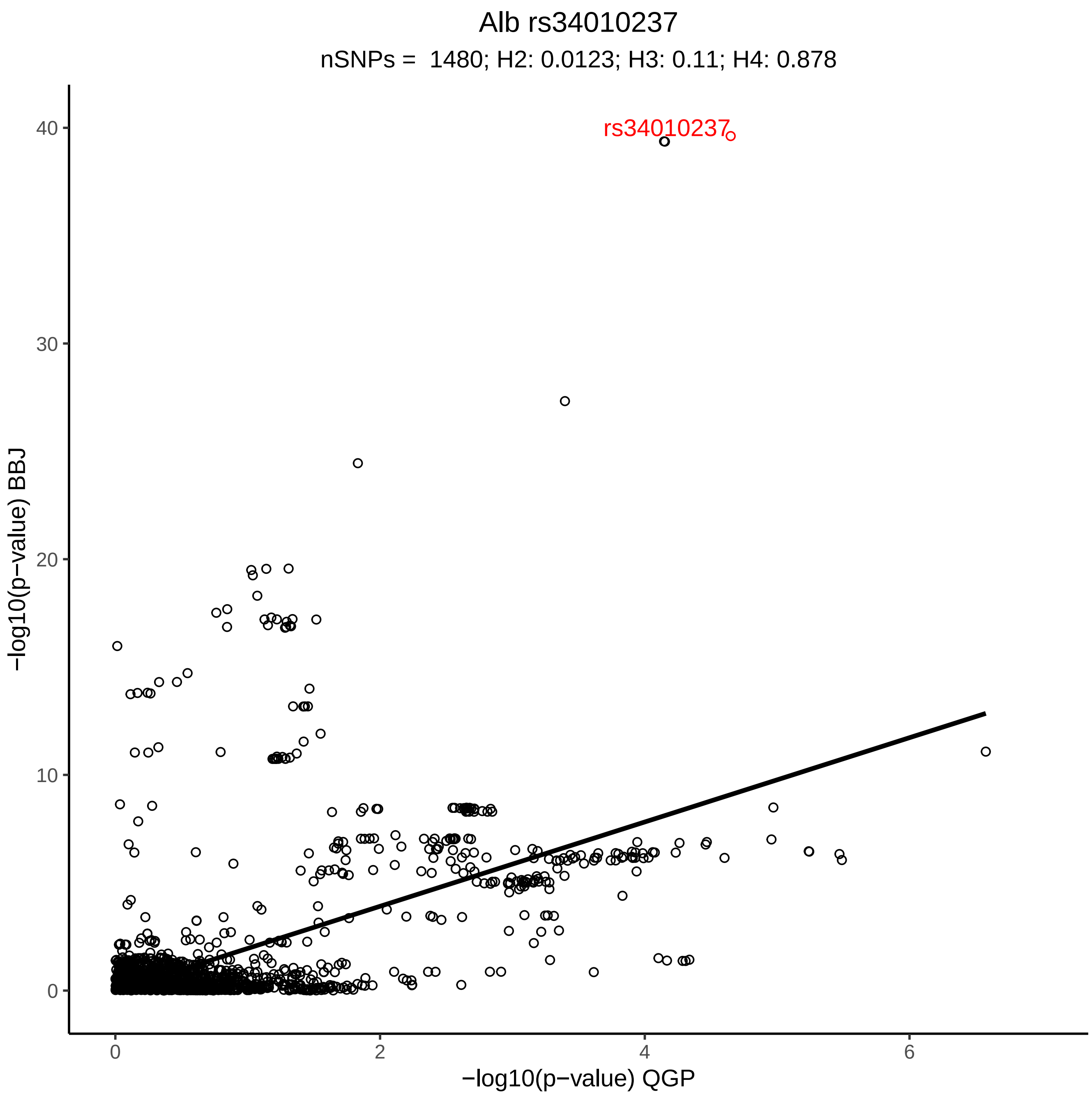

Alb rs34010237
nSNPs = 1480; H2: 0.0123; H3: 0.11; H4: 0.878
rs34010237
40
30
20
10
0
−log10(p−value) BBJ
0
2
4
6
−log10(p−value) QGP

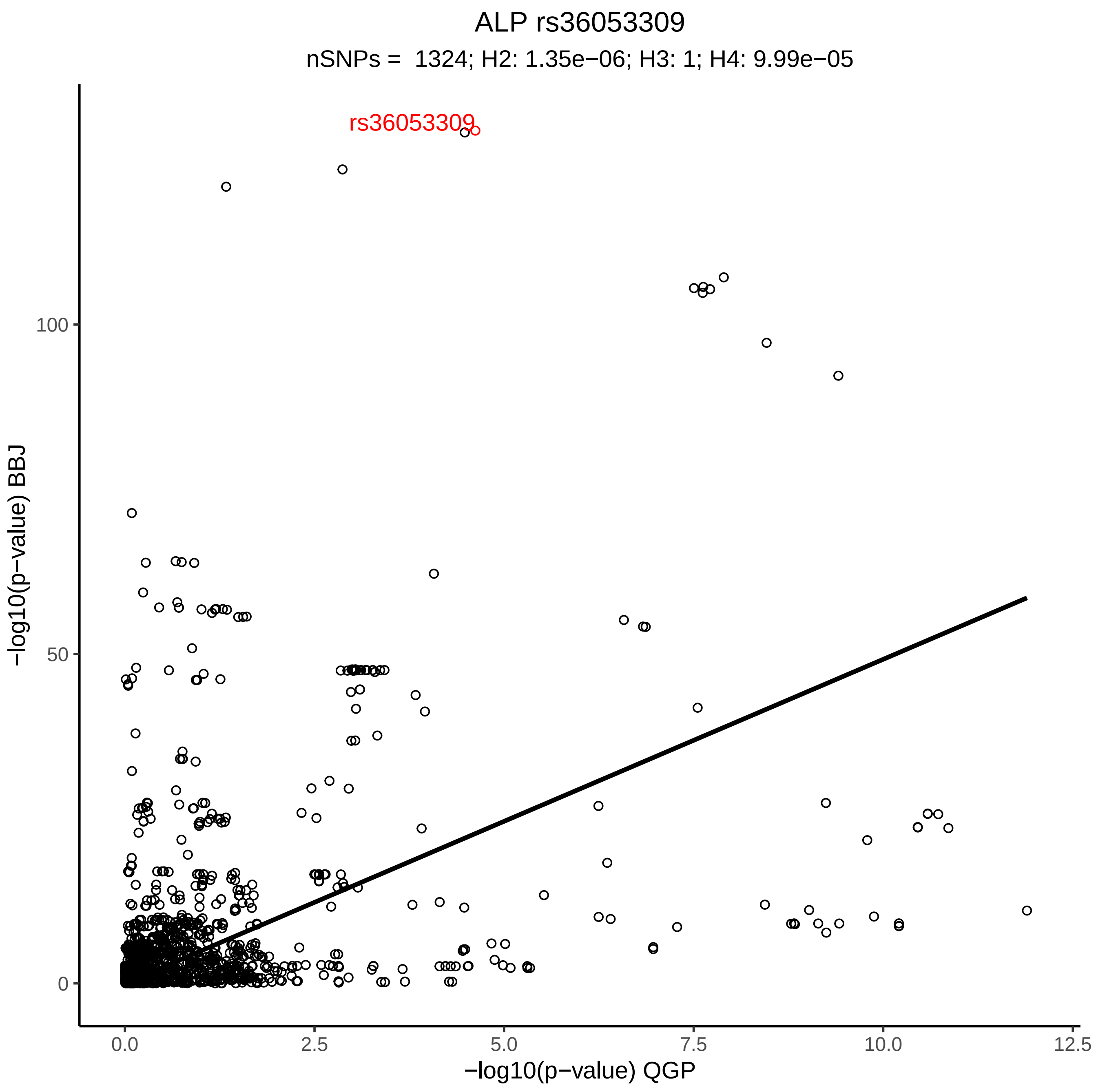
ALP rs36053309
nSNPs = 1324; H2: 1.35e−06; H3: 1; H4: 9.99e−05
rs36053309
−log10(p−value) BBJ
100
50
0
0.0
2.5
5.0
7.5
10.0
12.5
−log10(p−value) QGP

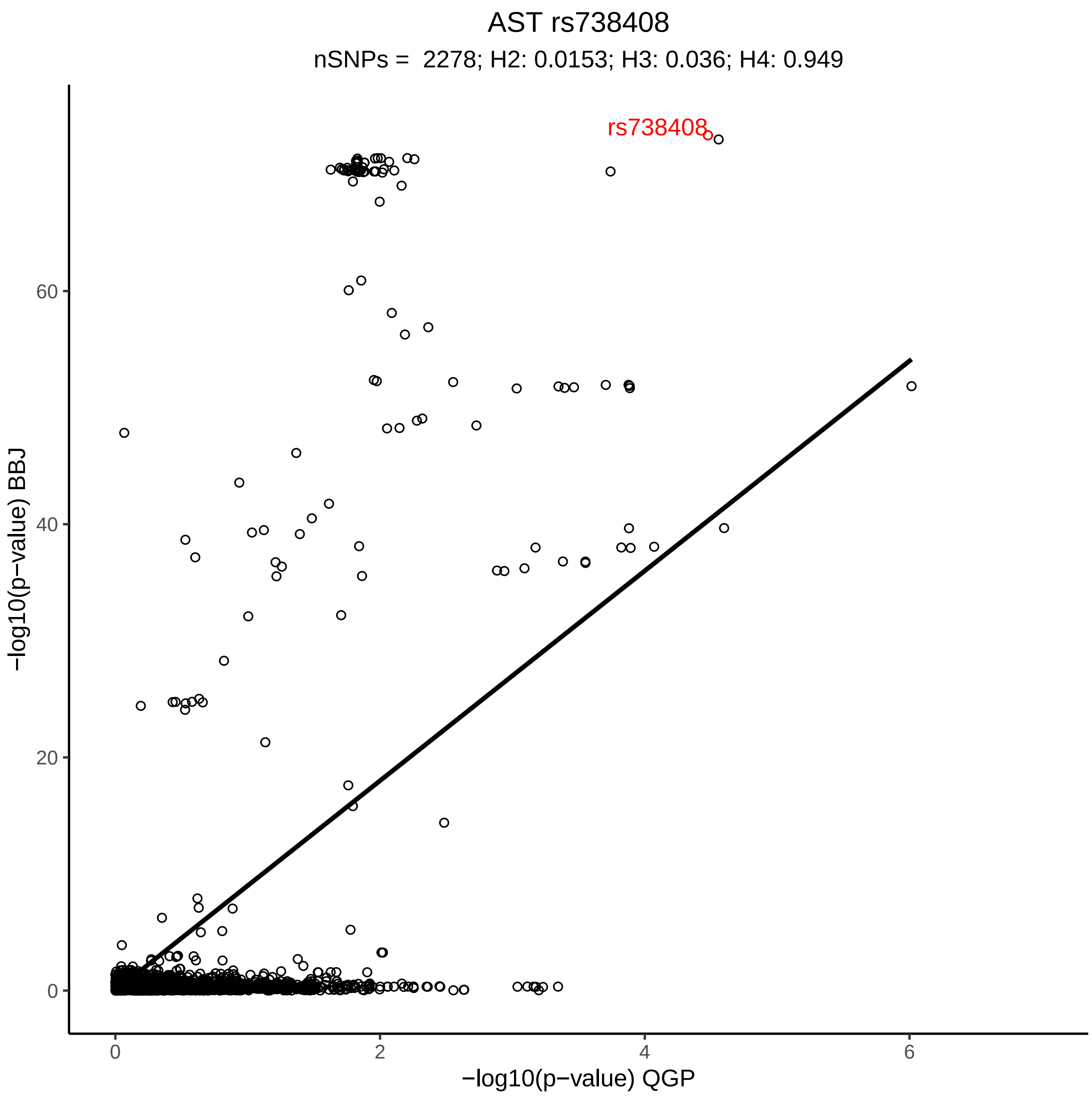

AST rs738408
nSNPs = 2278; H2: 0.0153; H3: 0.036; H4: 0.949
rs738408
60
40
20
0
0
2
4
6
−log10(p−value) BBJ
−log10(p−value) QGP

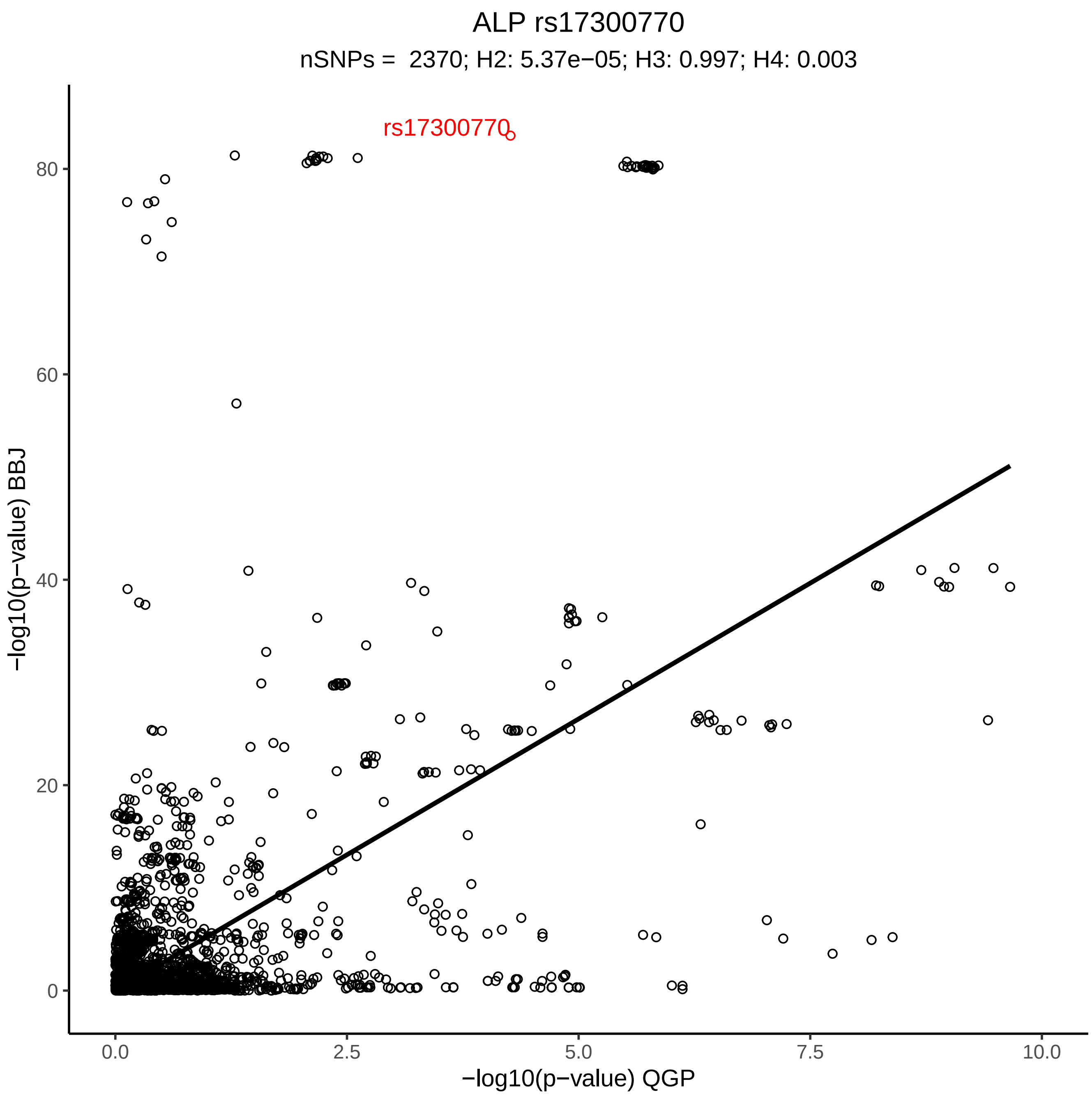
ALP rs17300770
nSNPs = 2370; H2: 5.37e−05; H3: 0.997; H4: 0.003
rs17300770
−log10(p−value) BBJ
−log10(p−value) QGP
0
20
40
60
80
0.0
2.5
5.0
7.5
10.0

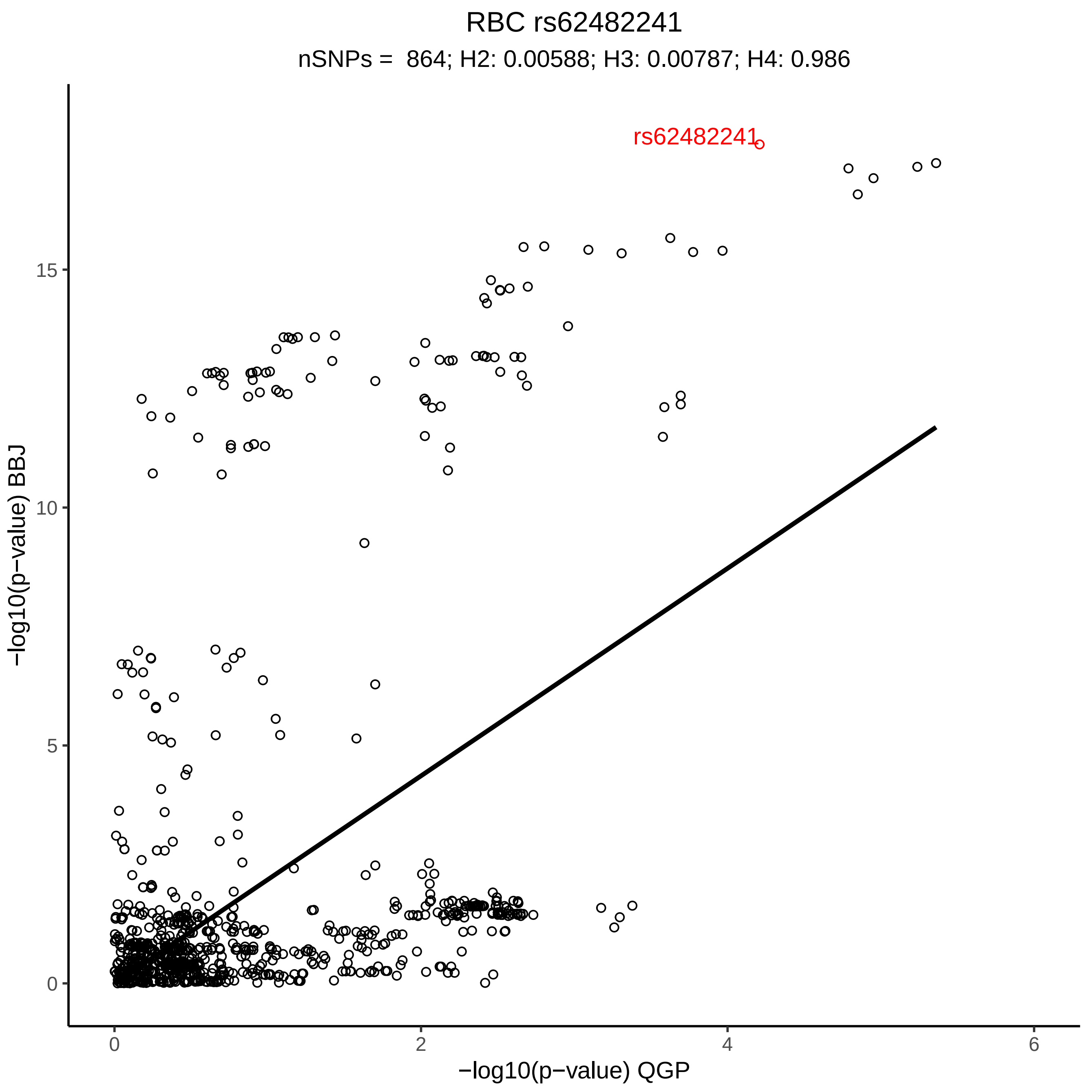
RBC rs62482241
nSNPs = 864; H2: 0.00588; H3: 0.00787; H4: 0.986
rs62482241
−log10(p−value) BBJ
−log10(p−value) QGP
0
5
10
15
0
2
4
6

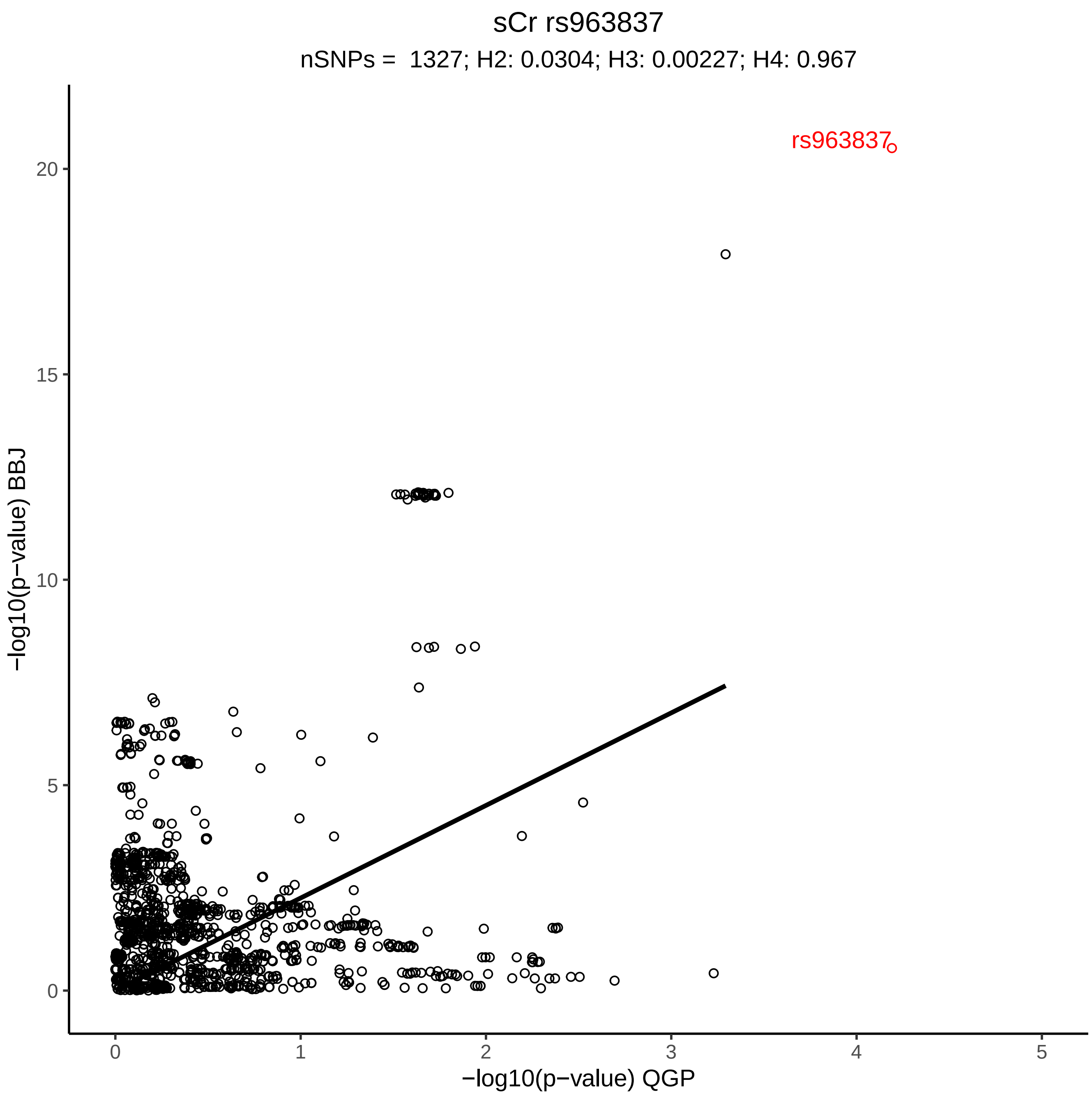
sCr rs963837
nSNPs = 1327; H2: 0.0304; H3: 0.00227; H4: 0.967
rs963837
−log10(p−value) BBJ
−log10(p−value) QGP
0
5
10
15
20
0
1
2
3
4
5

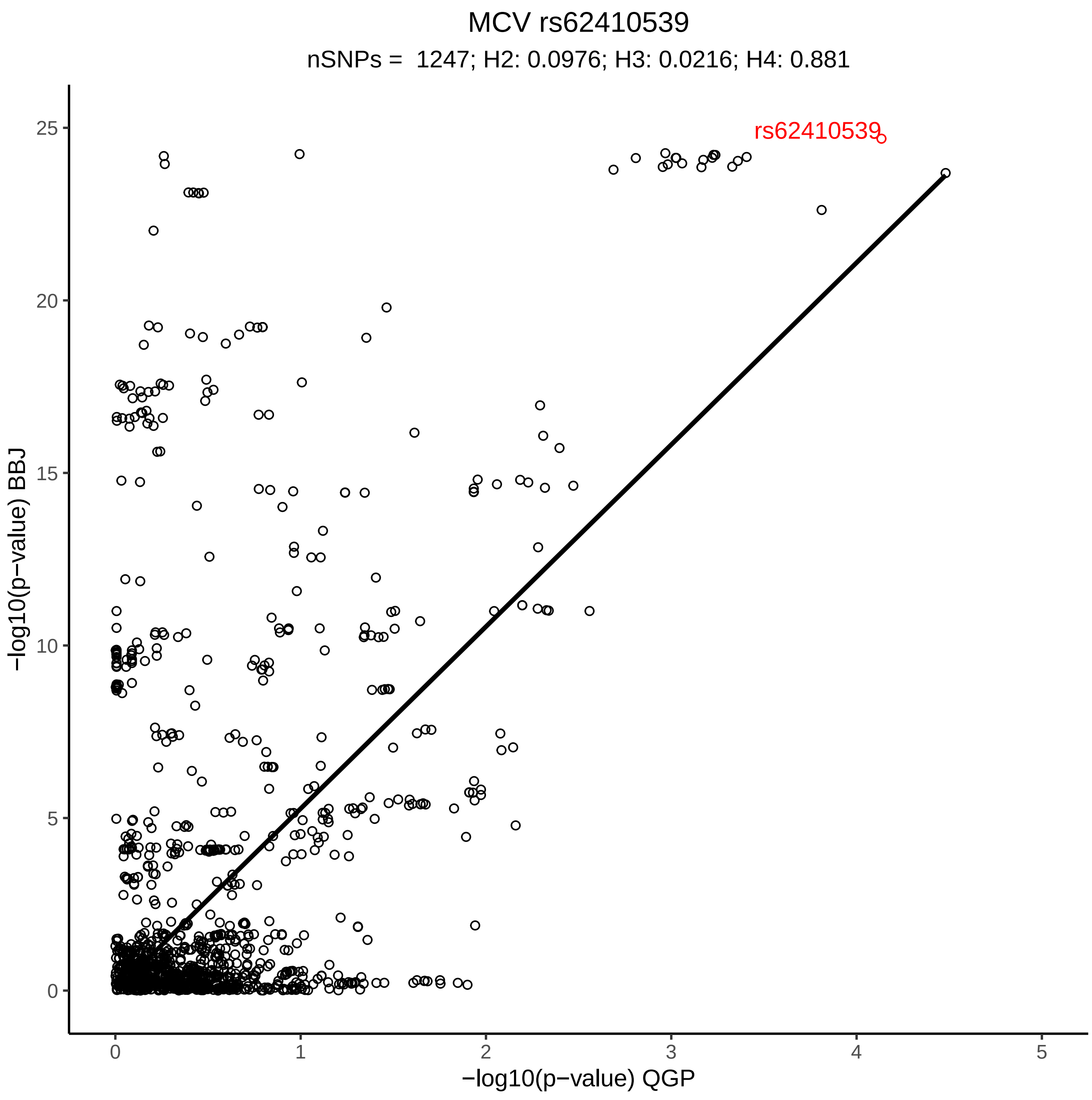
MCV rs62410539
nSNPs = 1247; H2: 0.0976; H3: 0.0216; H4: 0.881
rs62410539
−log10(p−value) BBJ
−log10(p−value) QGP
0
5
10
15
20
25
0
1
2
3
4
5

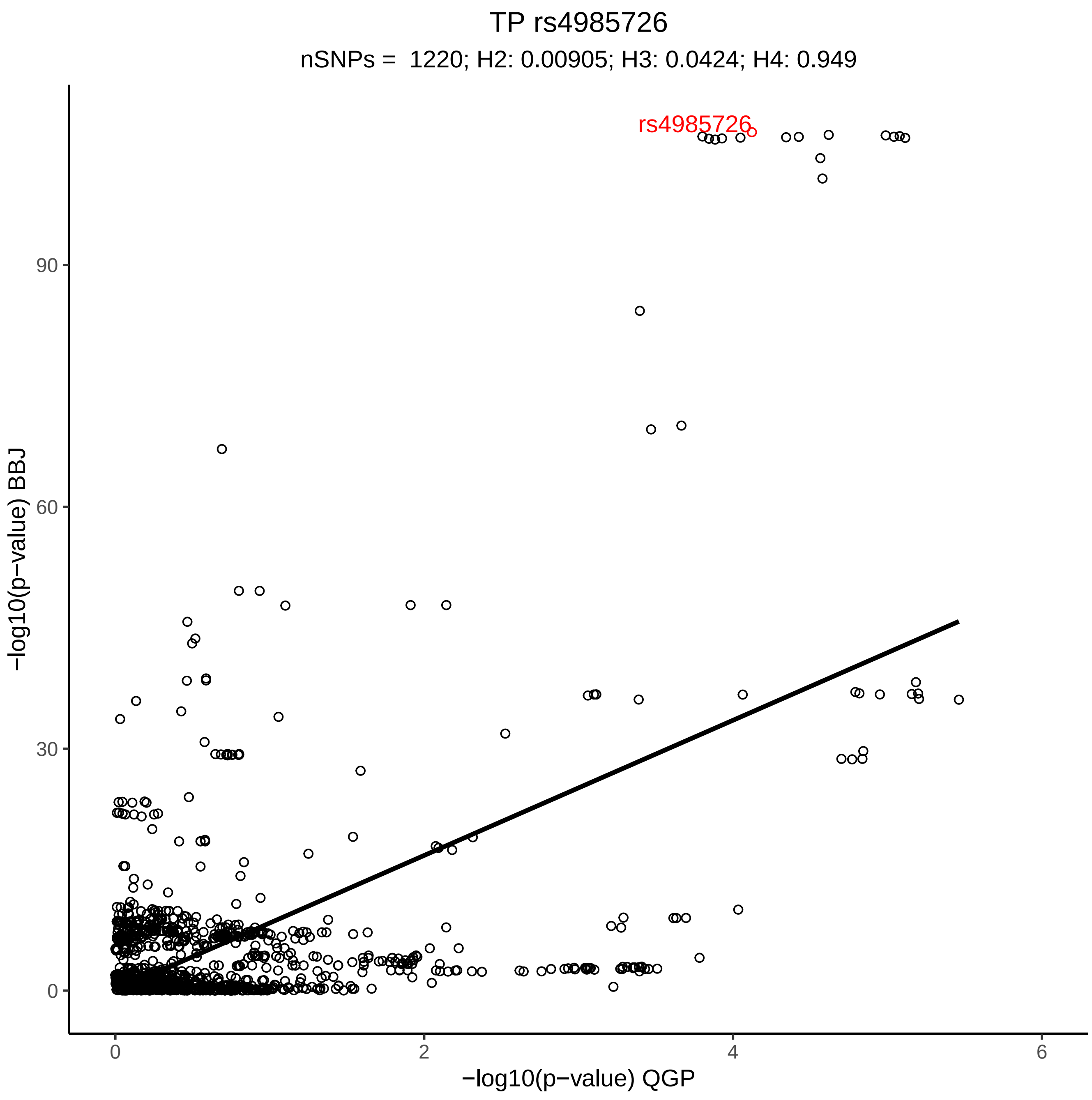

TP rs4985726
nSNPs = 1220; H2: 0.00905; H3: 0.0424; H4: 0.949
rs4985726
−log10(p−value) BBJ
−log10(p−value) QGP
0
30
60
90
0
2
4
6

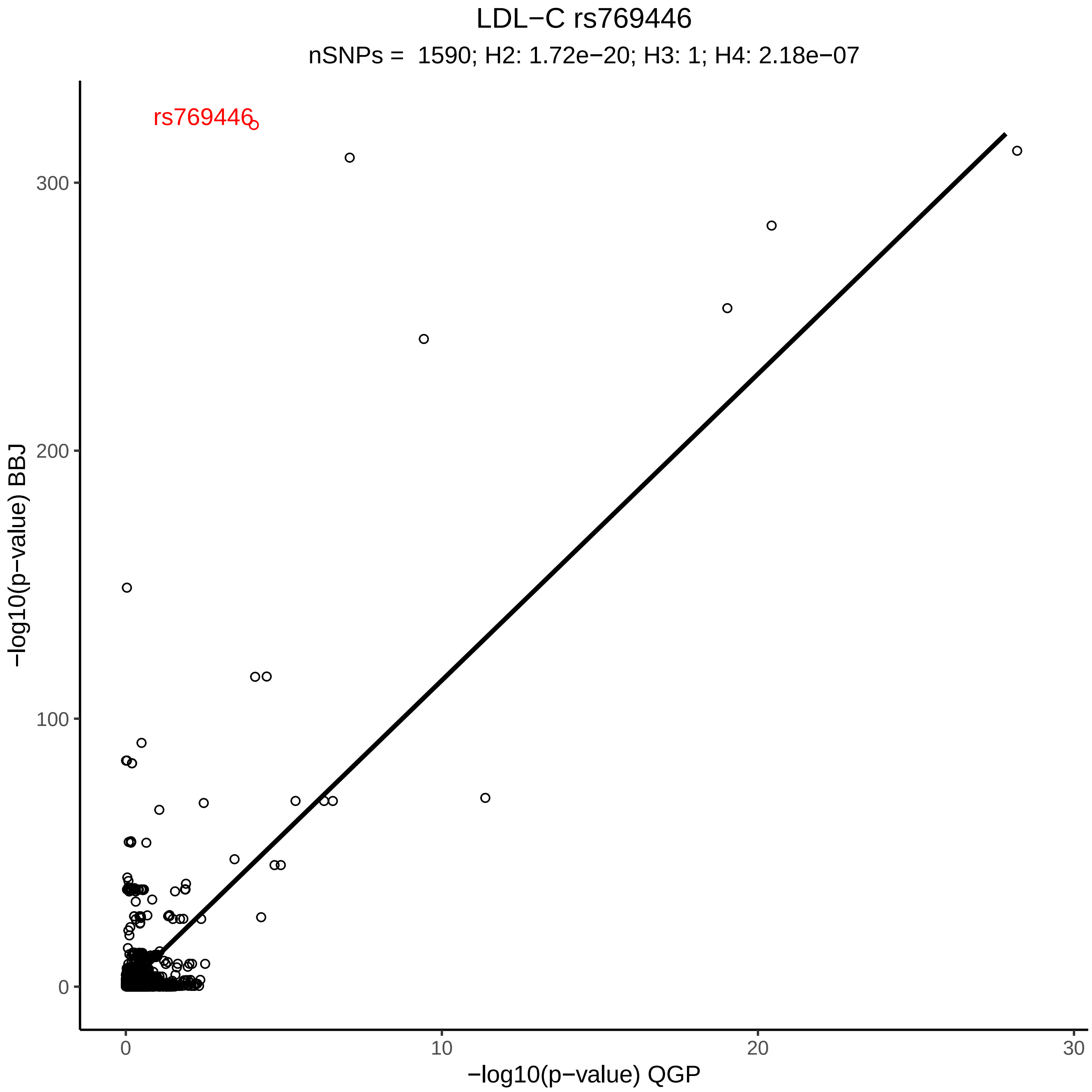

LDL−C rs769446
nSNPs = 1590; H2: 1.72e−20; H3: 1; H4: 2.18e−07
rs769446
300
200
100
0
−log10(p−value) BBJ
0
10
20
30
−log10(p−value) QGP

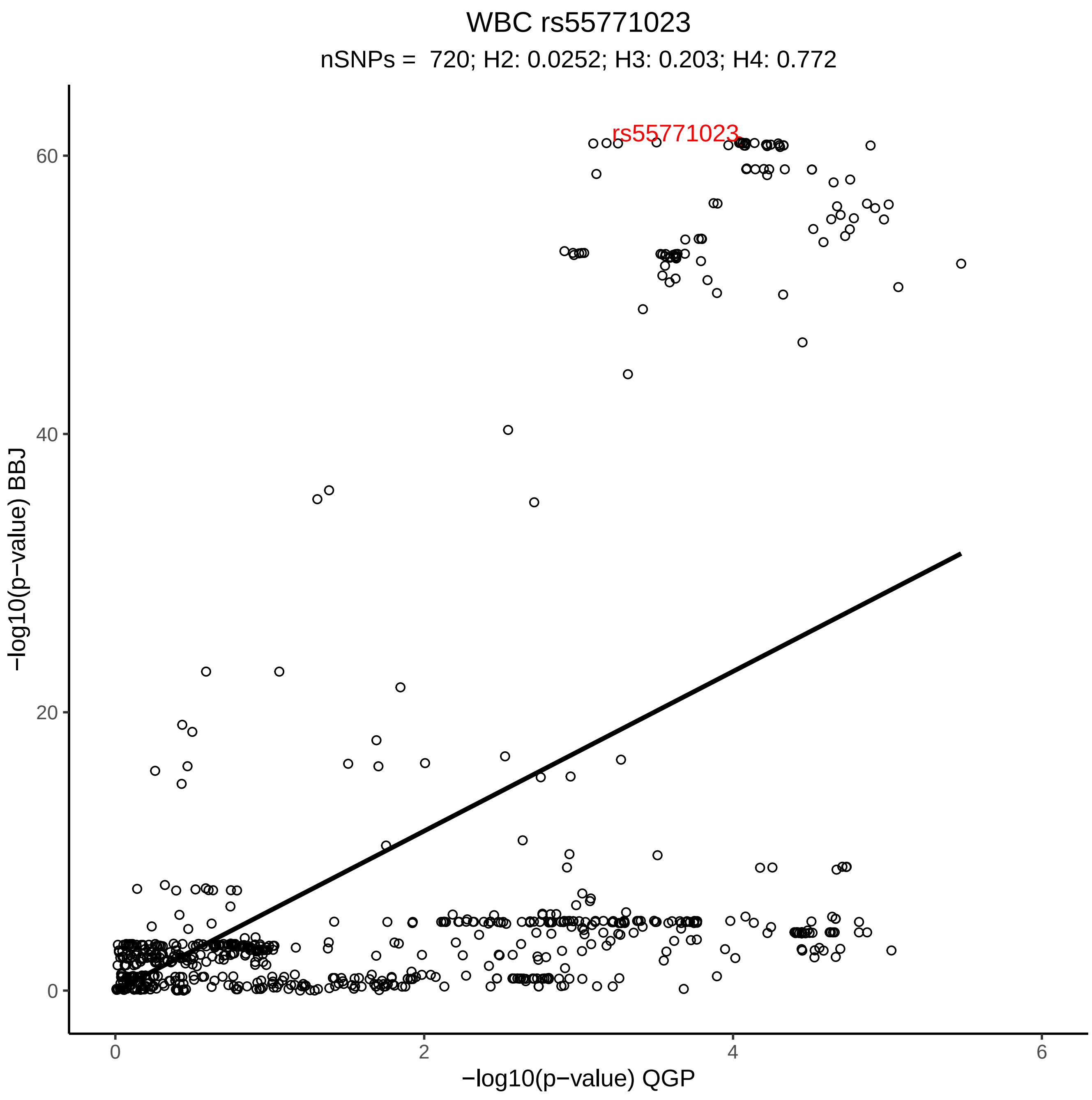
WBC rs55771023
nSNPs = 720; H2: 0.0252; H3: 0.203; H4: 0.772
rs55771023
−log10(p−value) BBJ
−log10(p−value) QGP
0
20
40
60
0
2
4
6

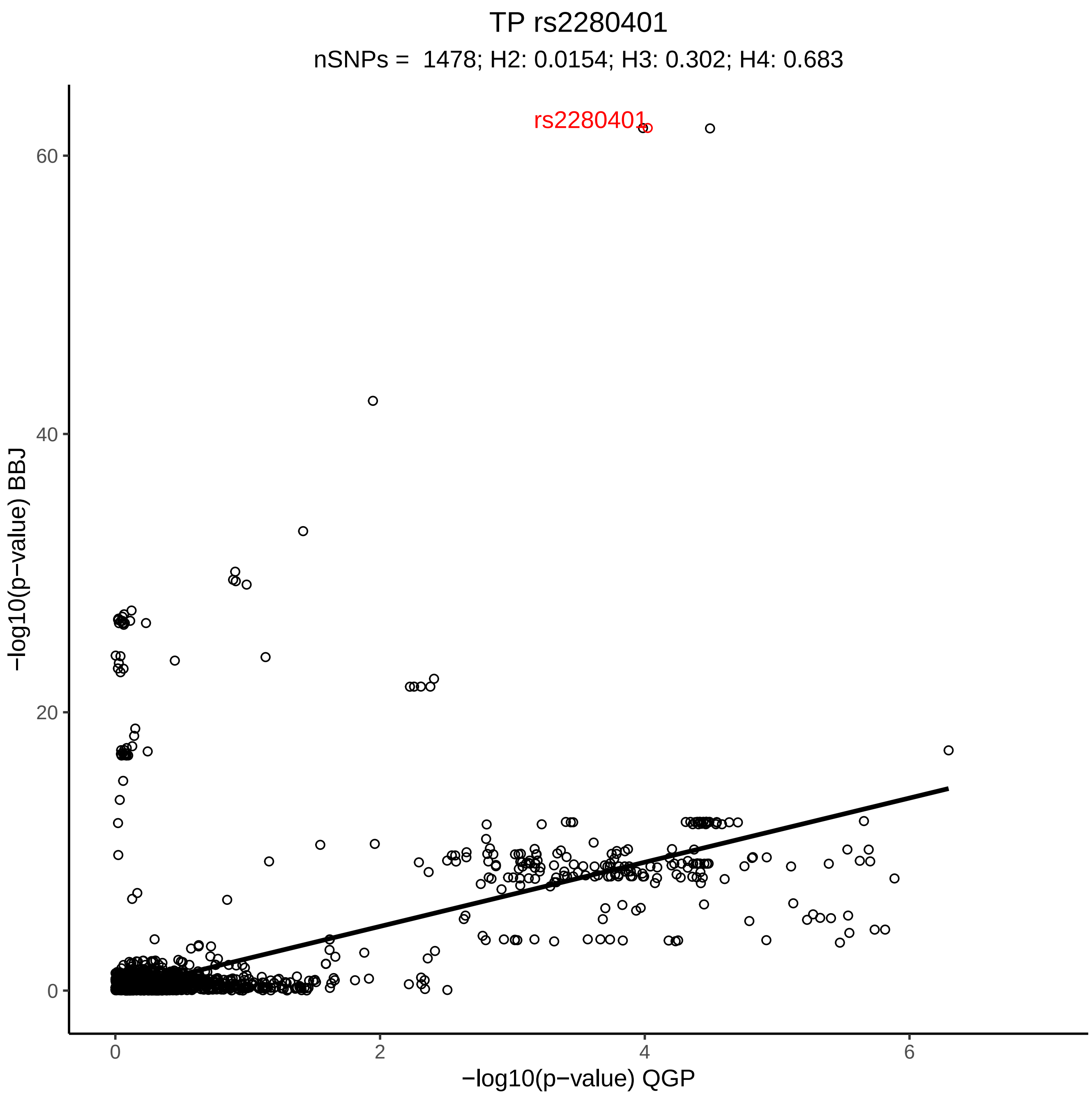
TP rs2280401
nSNPs = 1478; H2: 0.0154; H3: 0.302; H4: 0.683
rs2280401
−log10(p−value) BBJ
60
40
20
0
0
2
4
6
−log10(p−value) QGP

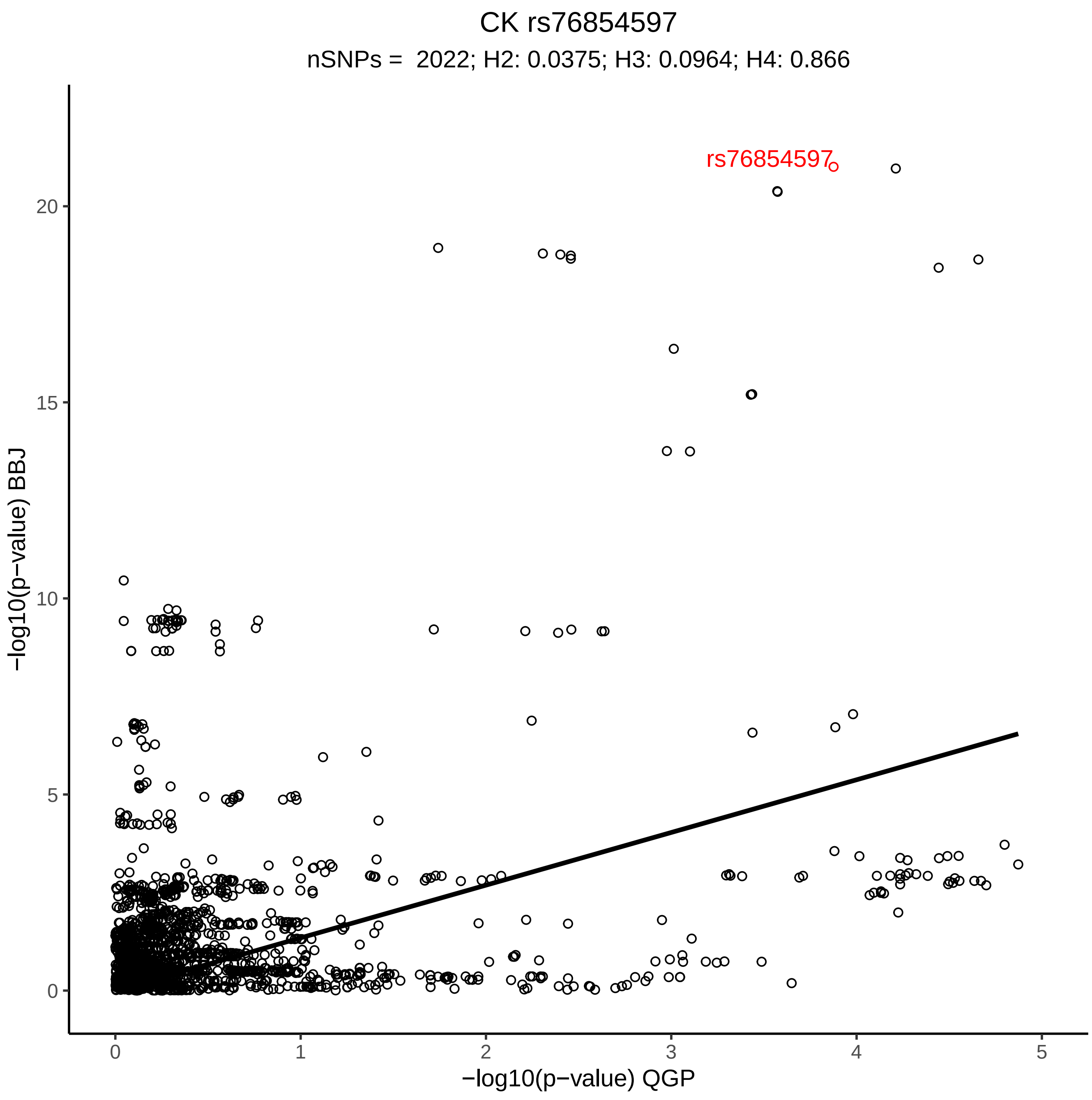
CK rs76854597
nSNPs = 2022; H2: 0.0375; H3: 0.0964; H4: 0.866
rs76854597
−log10(p−value) BBJ
−log10(p−value) QGP
0
5
10
15
20
0
1
2
3
4
5

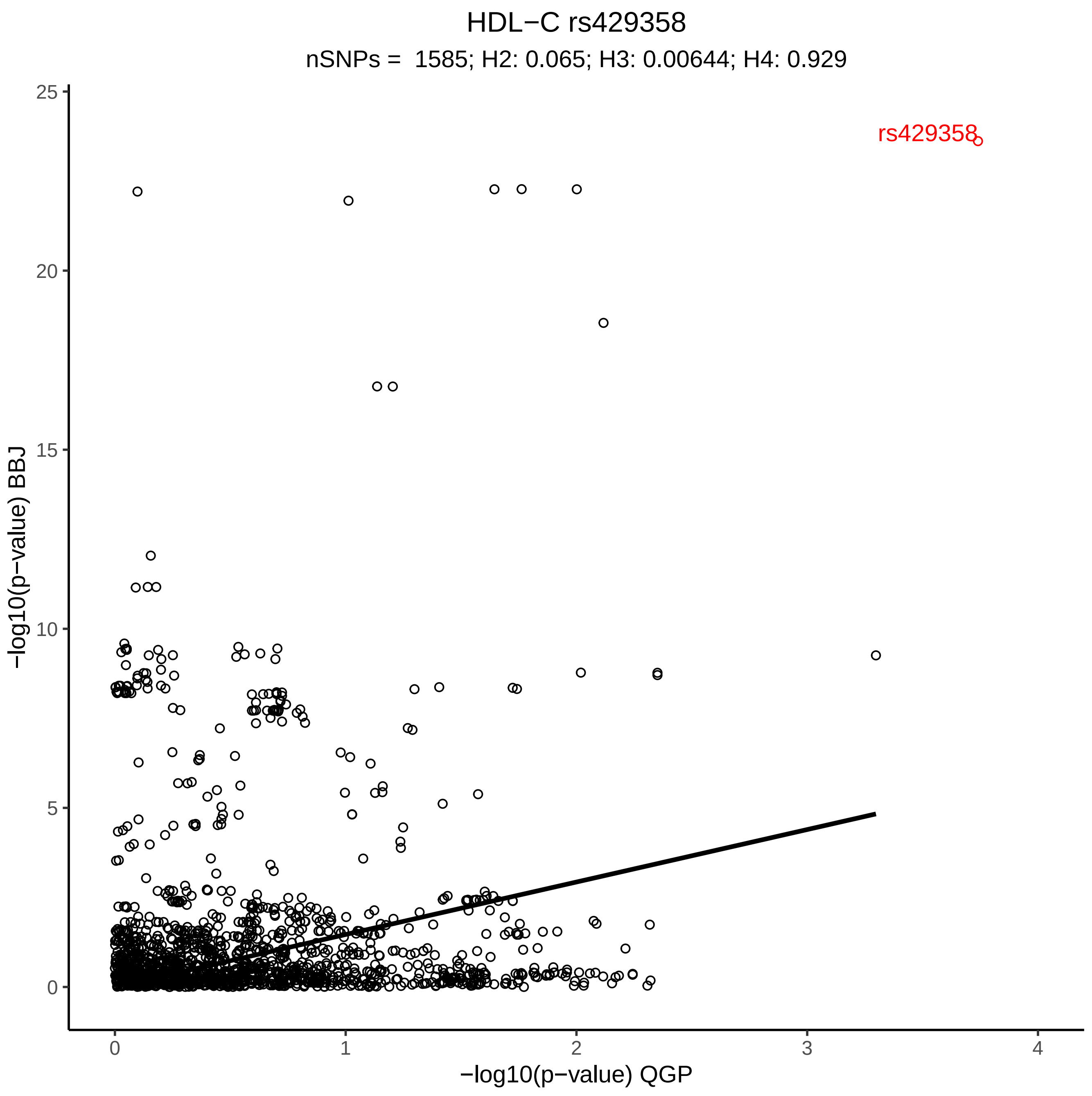

HDL−C rs429358
nSNPs = 1585; H2: 0.065; H3: 0.00644; H4: 0.929
rs429358
−log10(p−value) BBJ
−log10(p−value) QGP
25
20
15
10
5
0
0
1
2
3
4

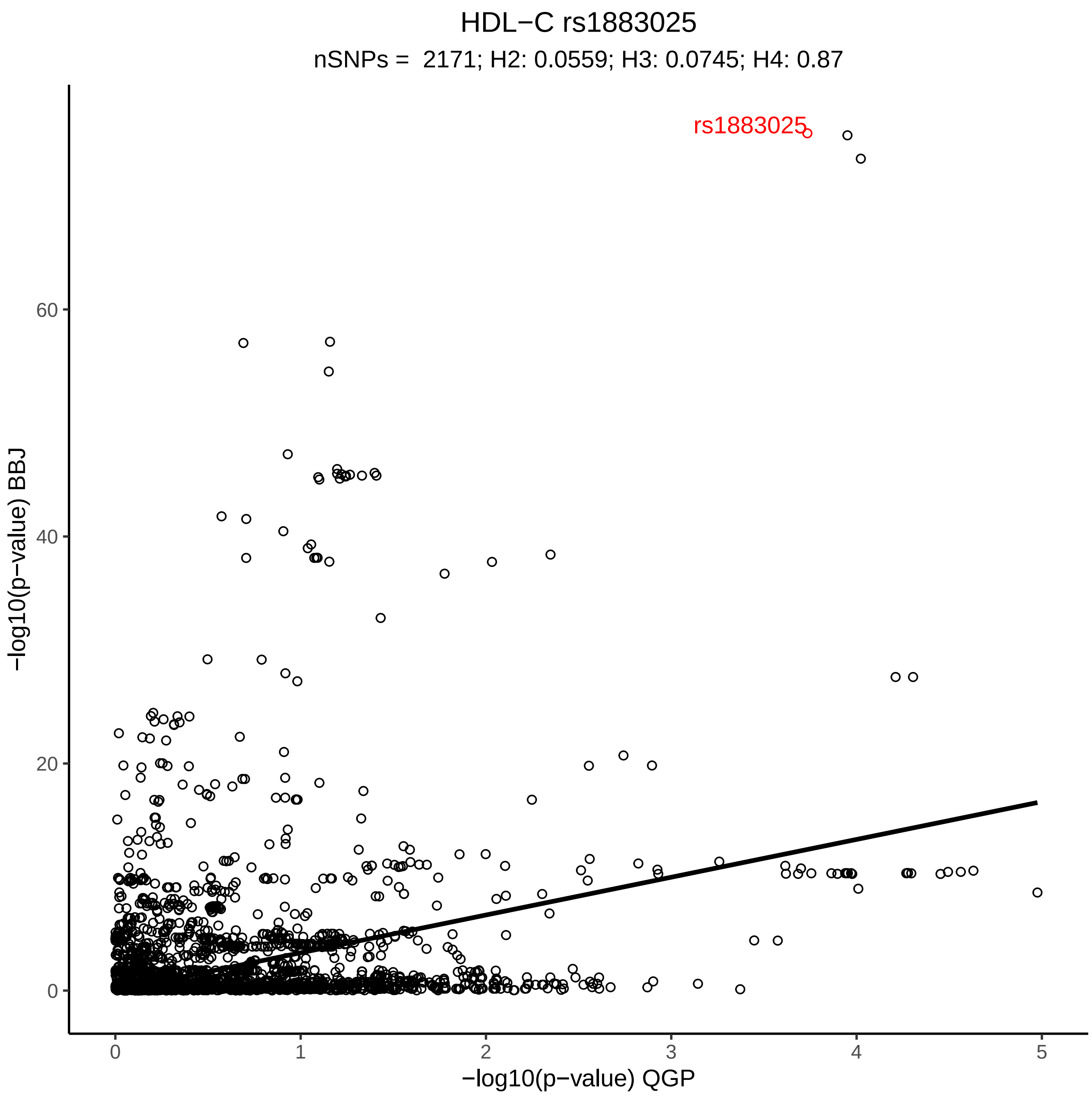
HDL−C rs1883025
nSNPs = 2171; H2: 0.0559; H3: 0.0745; H4: 0.87
rs1883025
−log10(p−value) BBJ
−log10(p−value) QGP
0
20
40
60
0
1
2
3
4
5

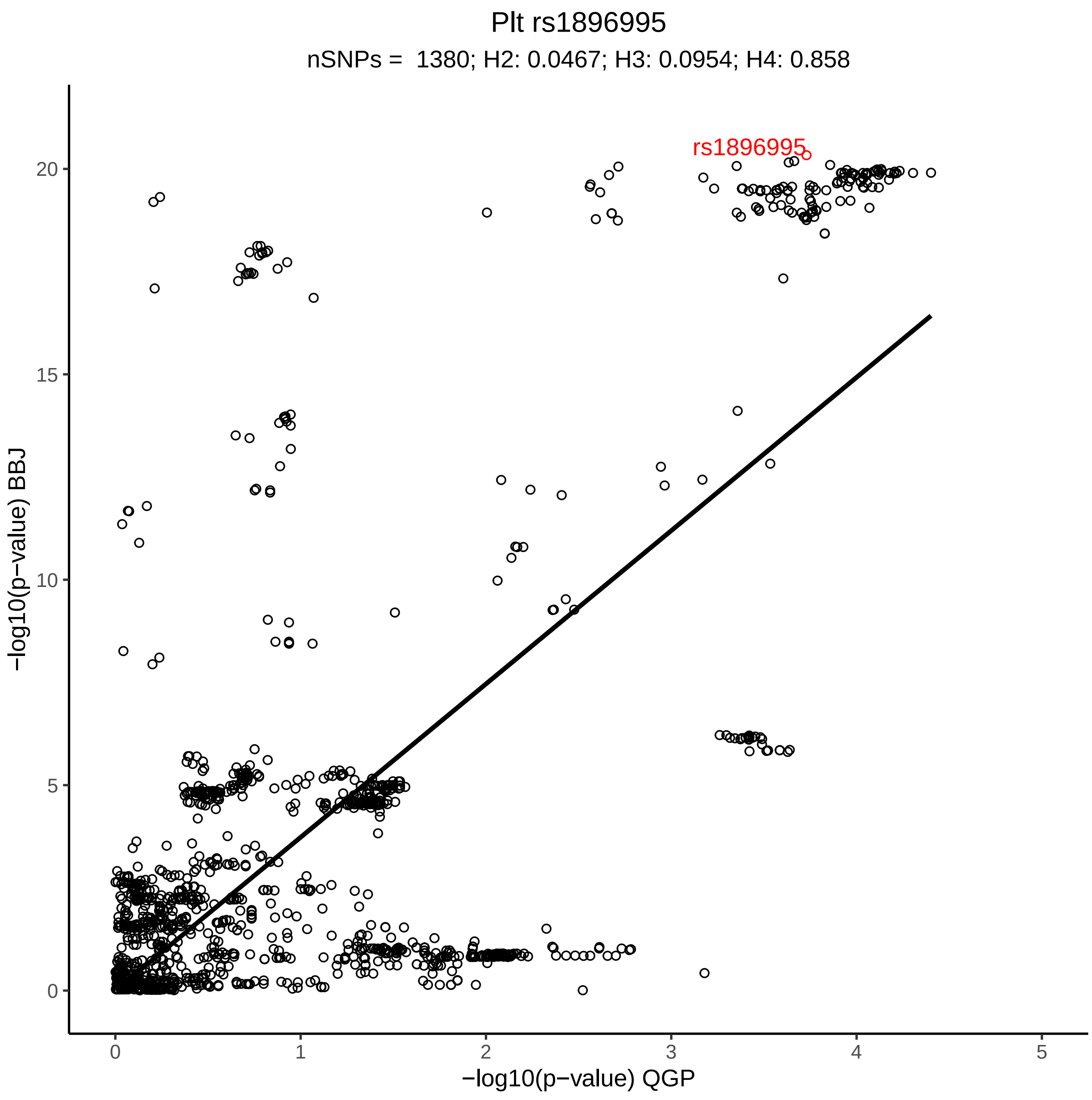

Plt rs1896995
nSNPs = 1380; H2: 0.0467; H3: 0.0954; H4: 0.858
rs1896995
−log10(p−value) BBJ
−log10(p−value) QGP
0
5
10
15
20
0
1
2
3
4
5

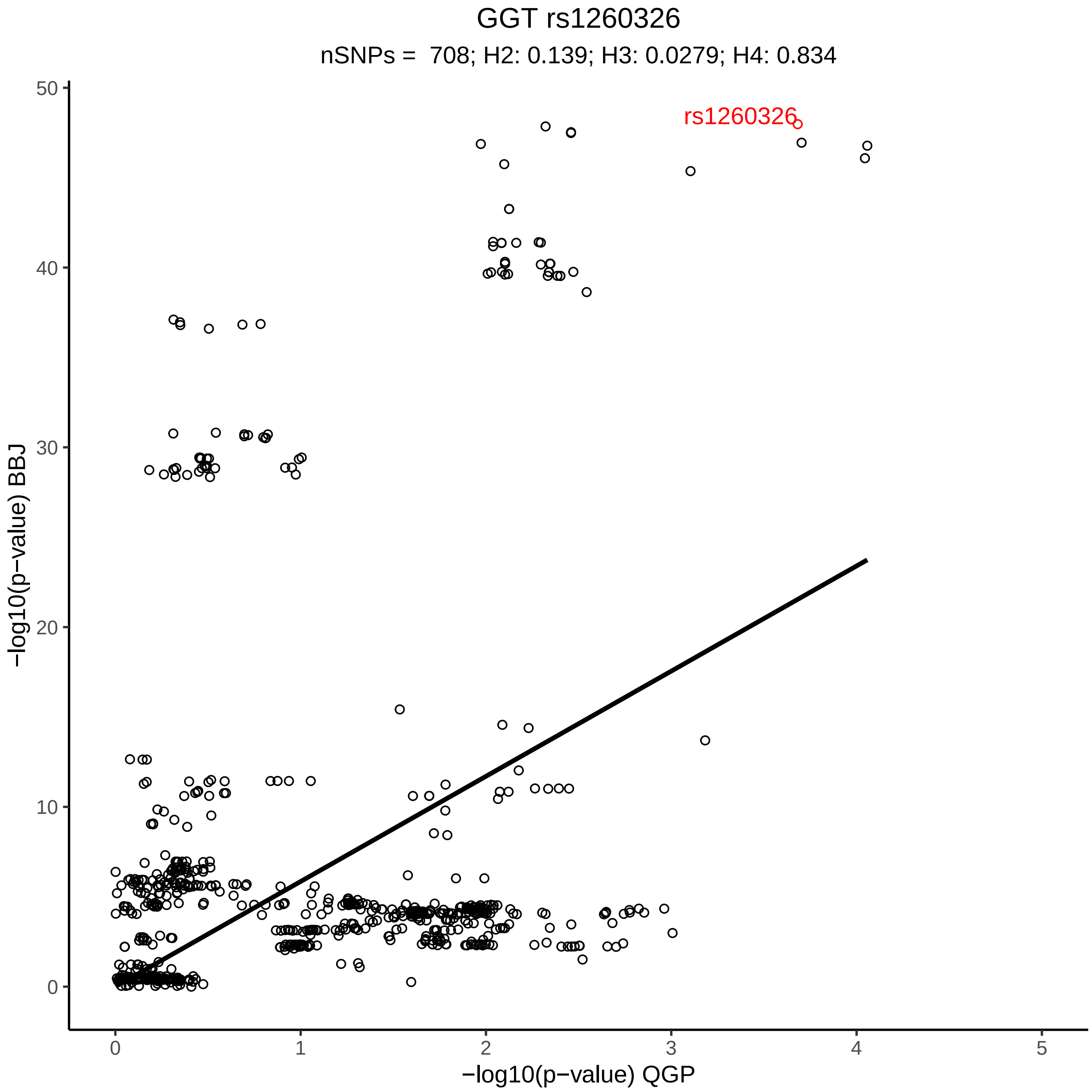
GGT rs1260326
nSNPs = 708; H2: 0.139; H3: 0.0279; H4: 0.834
rs1260326
−log10(p−value) BBJ
−log10(p−value) QGP
0
10
20
30
40
50
0
1
2
3
4
5

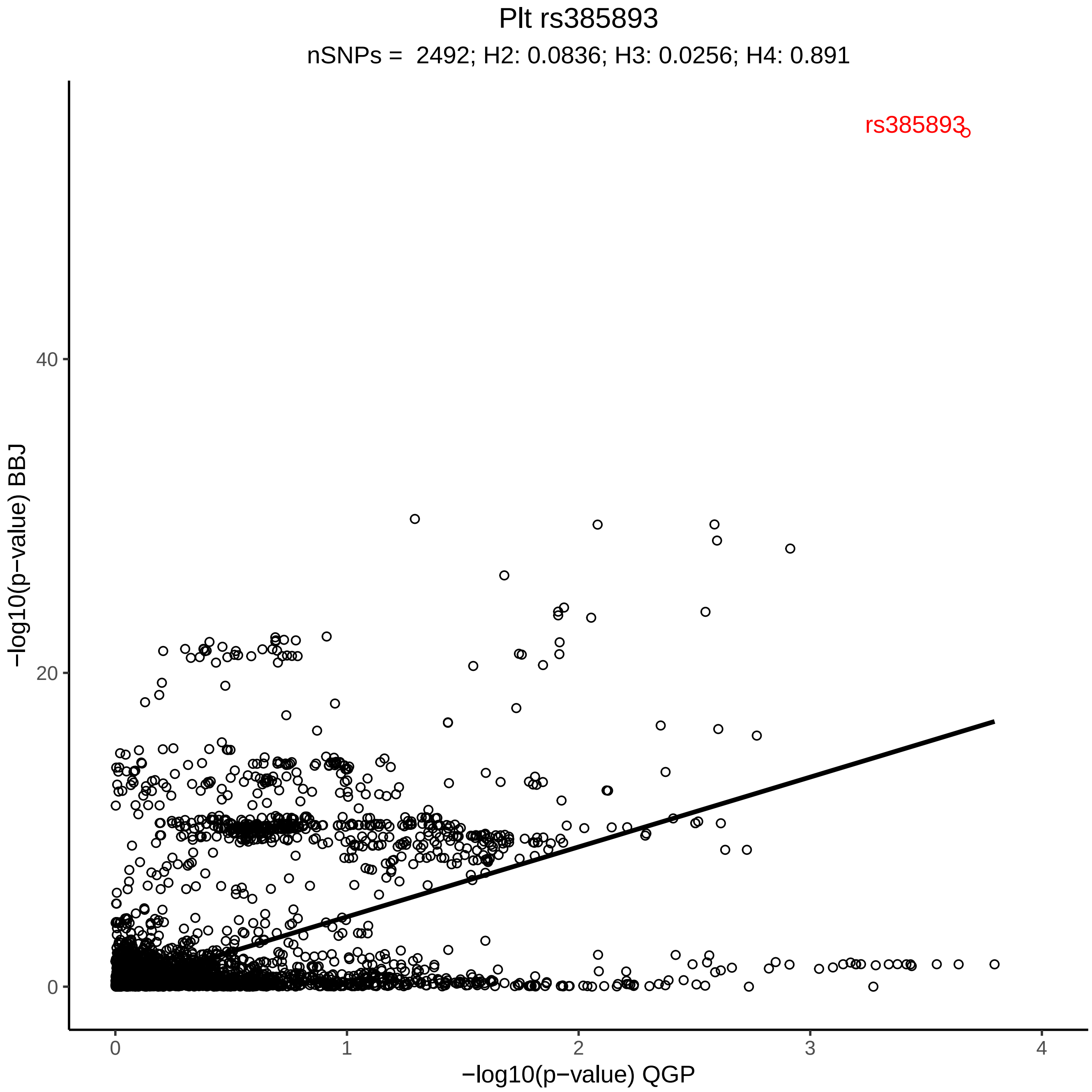

Plt rs385893
nSNPs = 2492; H2: 0.0836; H3: 0.0256; H4: 0.891
rs385893
−log10(p−value) BBJ
−log10(p−value) QGP
0
20
40
0
1
2
3
4

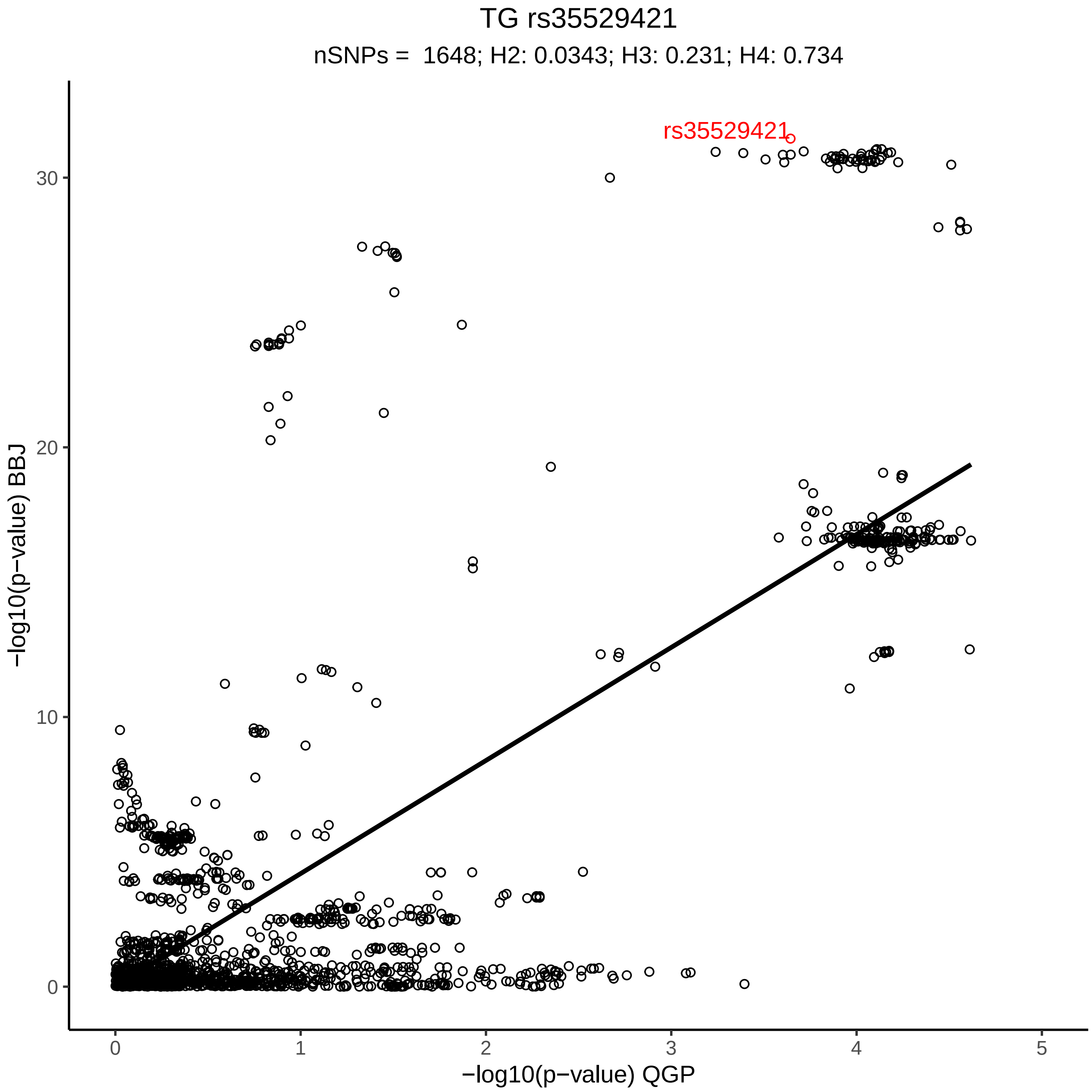

TG rs35529421
nSNPs = 1648; H2: 0.0343; H3: 0.231; H4: 0.734
rs35529421
−log10(p−value) BBJ
−log10(p−value) QGP
0
10
20
30
0
1
2
3
4
5

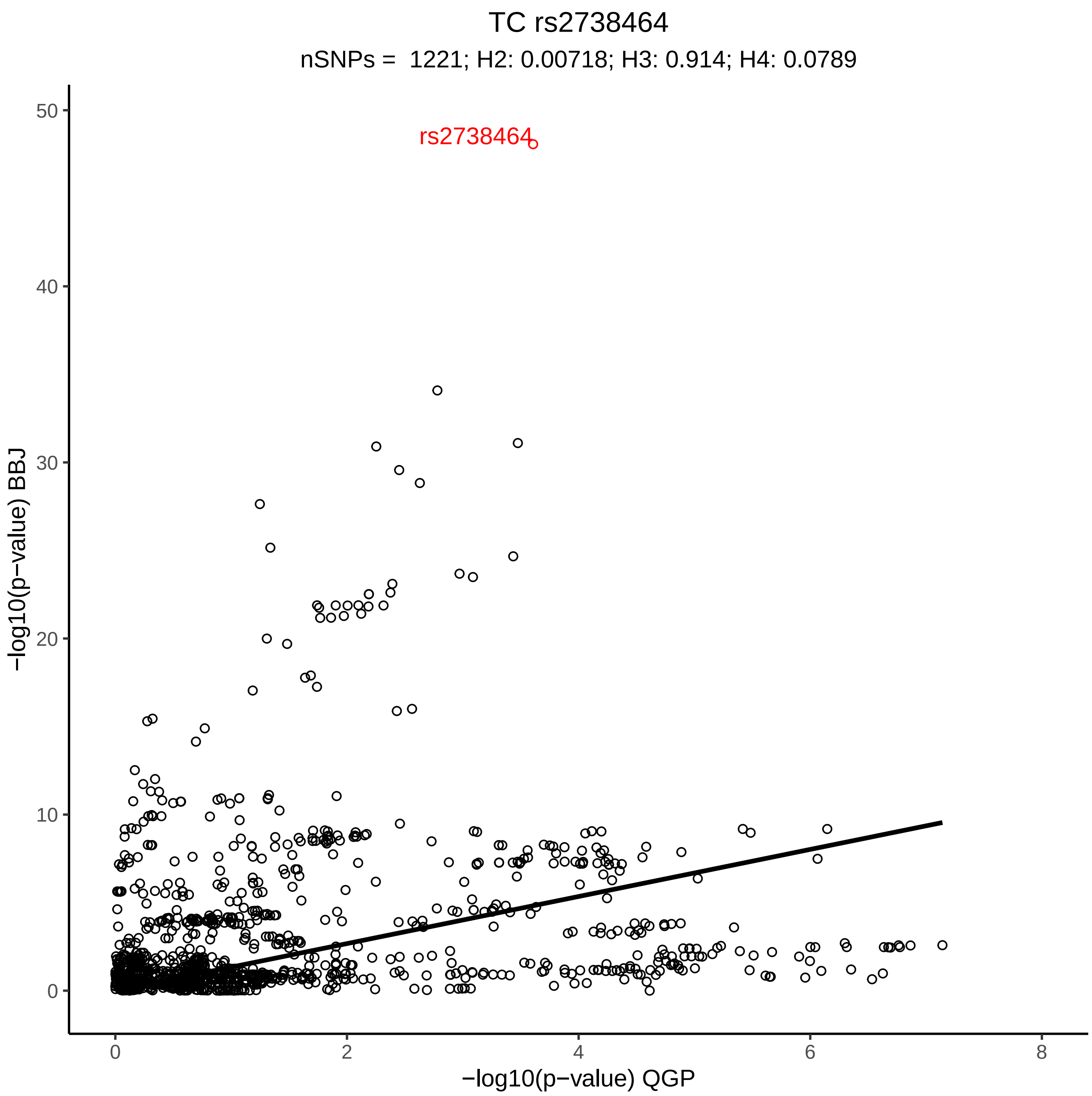

TC rs2738464
nSNPs = 1221; H2: 0.00718; H3: 0.914; H4: 0.0789
rs2738464
−log10(p−value) BBJ
−log10(p−value) QGP
0
10
20
30
40
50
0
2
4
6
8

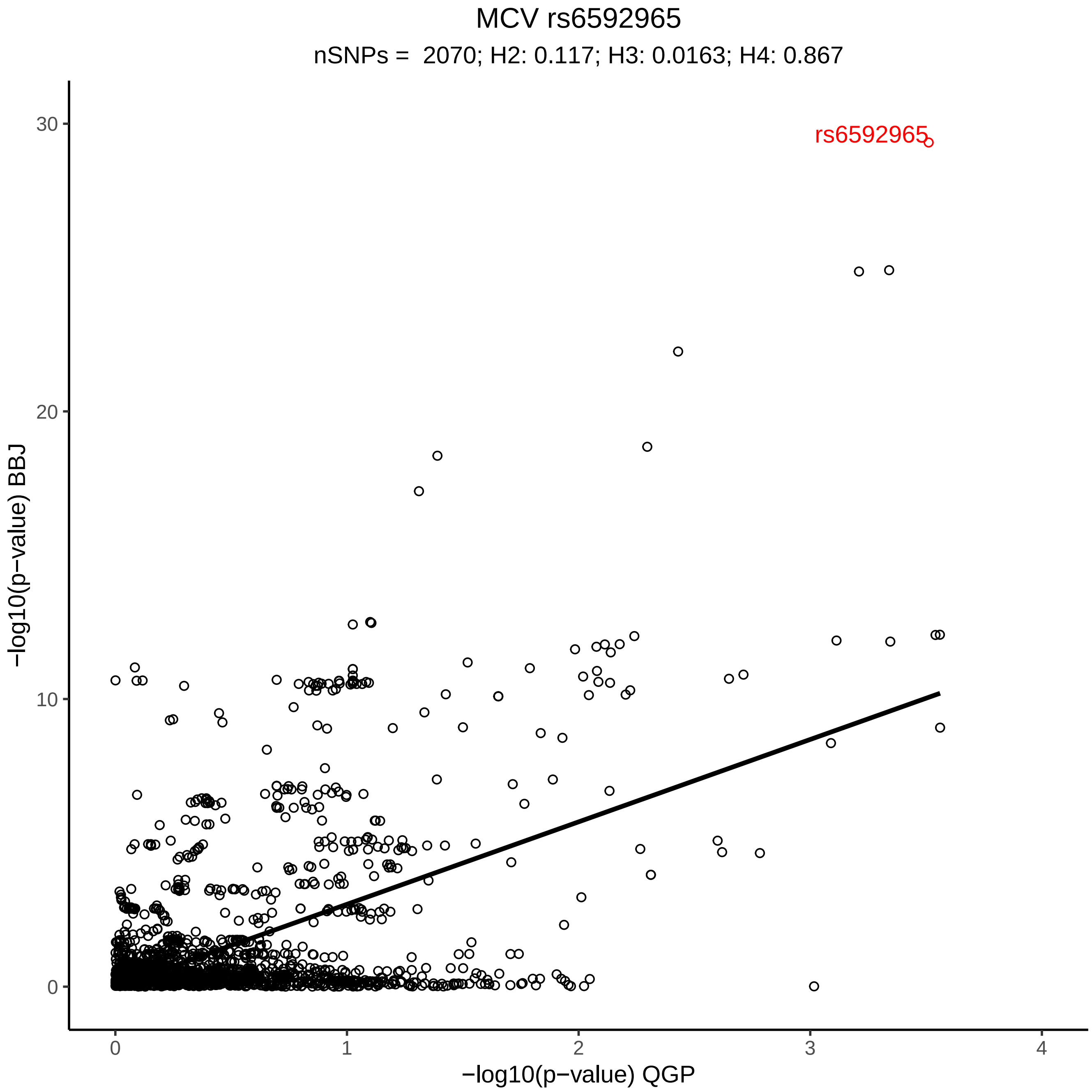

MCV rs6592965
nSNPs = 2070; H2: 0.117; H3: 0.0163; H4: 0.867
rs6592965
30
20
10
0
0
1
2
3
4
−log10(p−value) BBJ
−log10(p−value) QGP

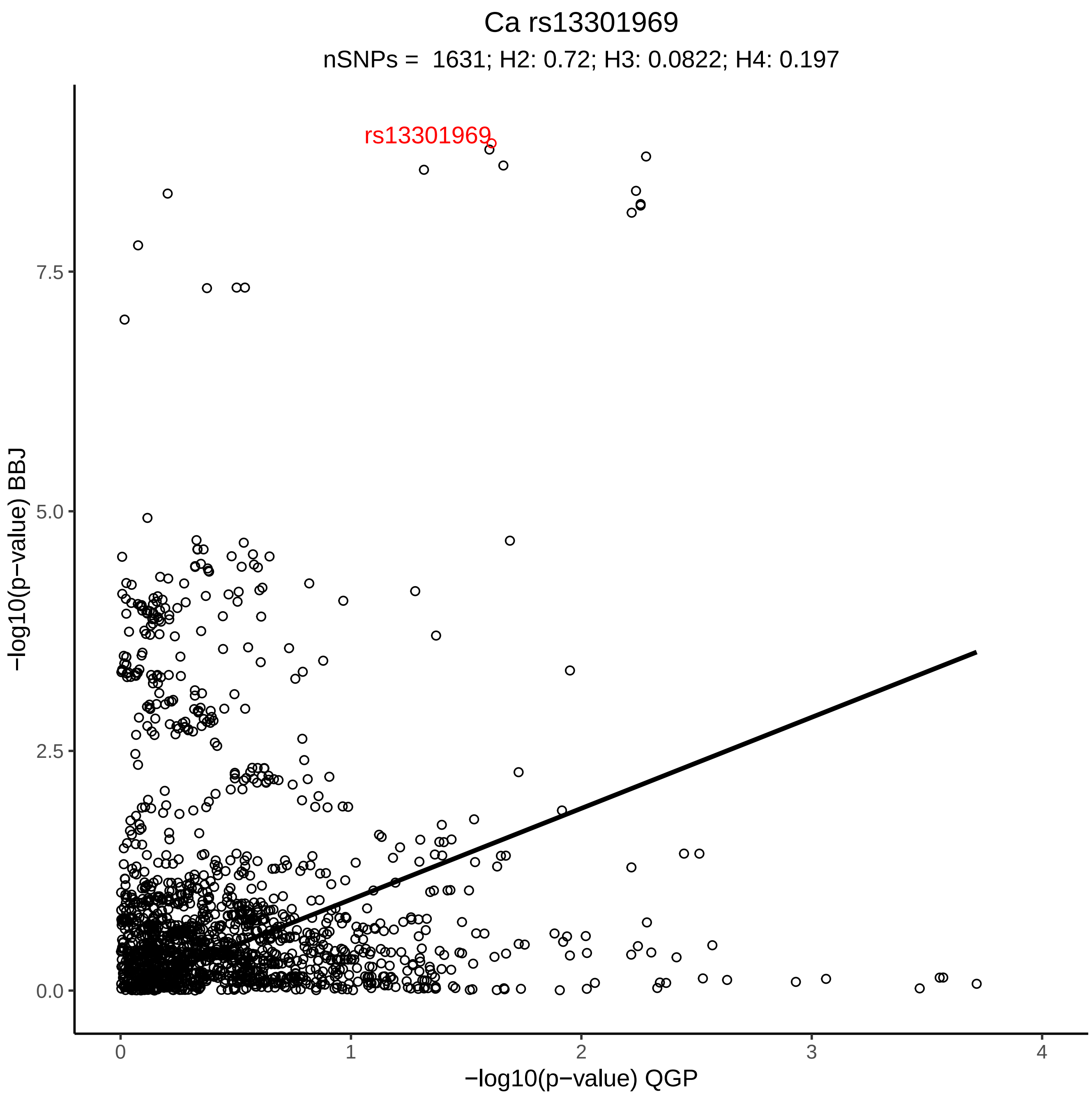

Ca rs13301969
nSNPs = 1631; H2: 0.72; H3: 0.0822; H4: 0.197
rs13301969
−log10(p−value) BBJ
−log10(p−value) QGP
0.0
2.5
5.0
7.5
0
1
2
3
4

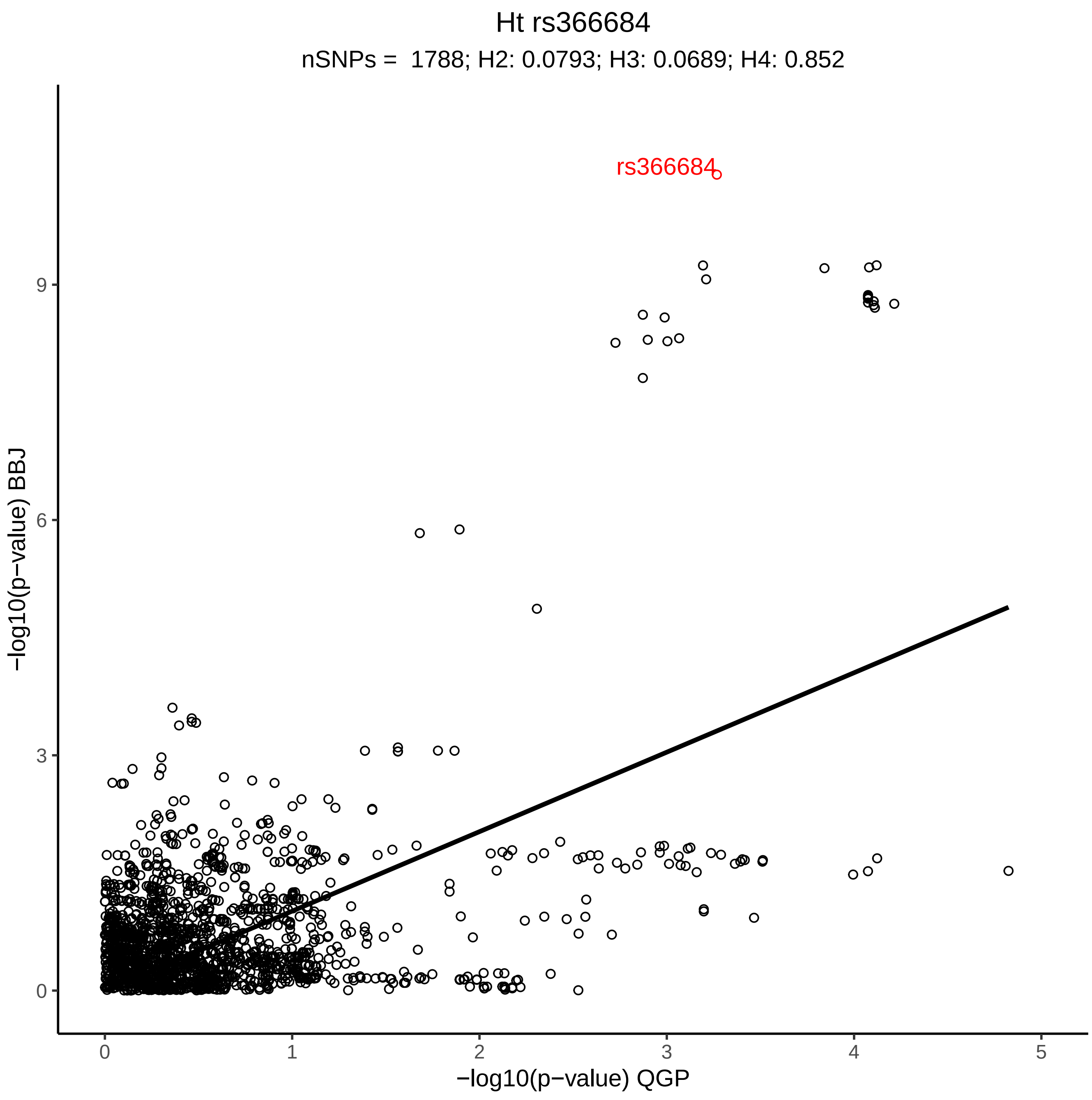
Ht rs366684
nSNPs = 1788; H2: 0.0793; H3: 0.0689; H4: 0.852
rs366684
9
6
3
0
−log10(p−value) BBJ
0
1
2
3
4
5
−log10(p−value) QGP

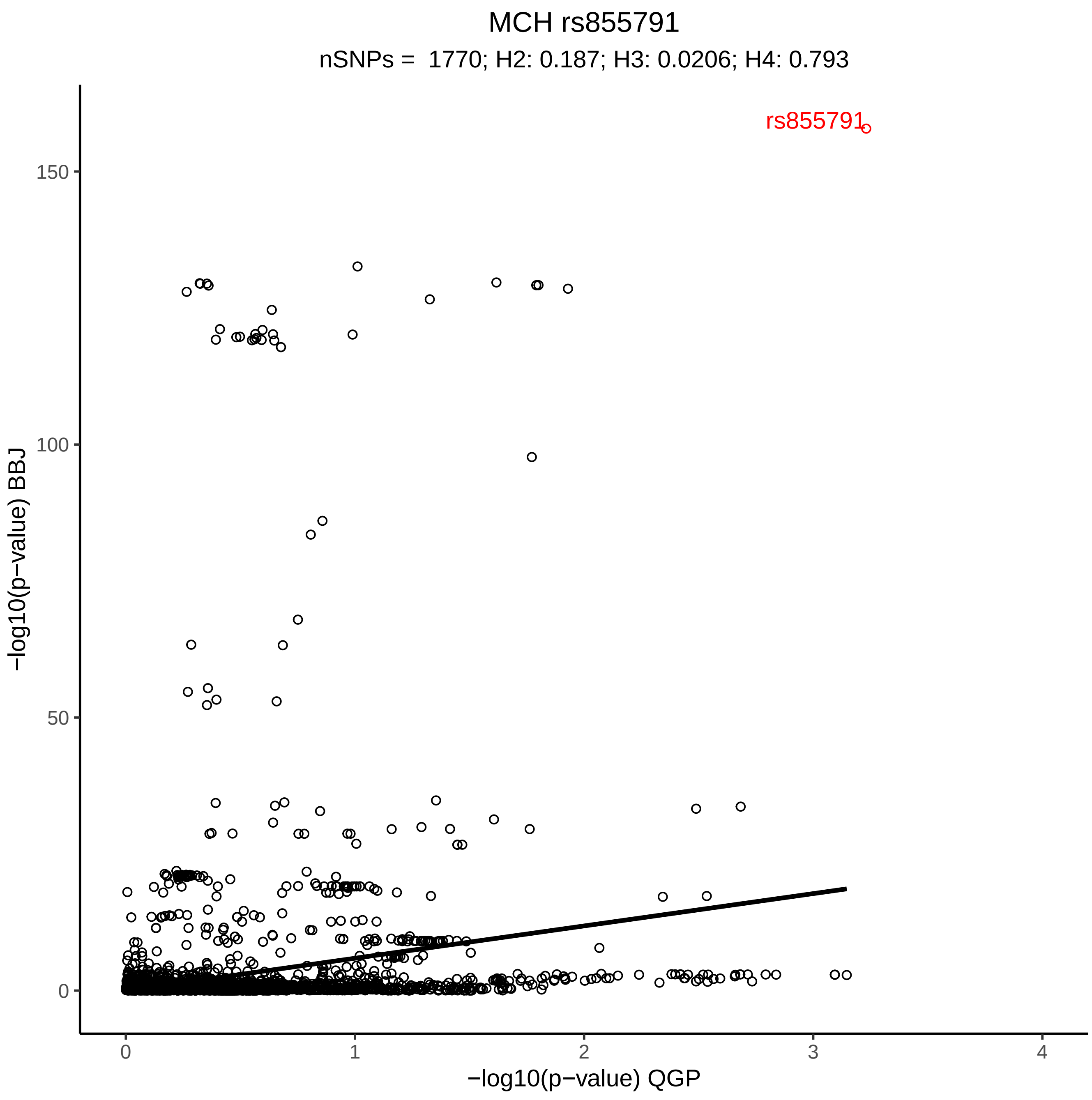
MCH rs855791
nSNPs = 1770; H2: 0.187; H3: 0.0206; H4: 0.793
rs855791
150
100
50
0
−log10(p−value) BBJ
0
1
2
3
4
−log10(p−value) QGP

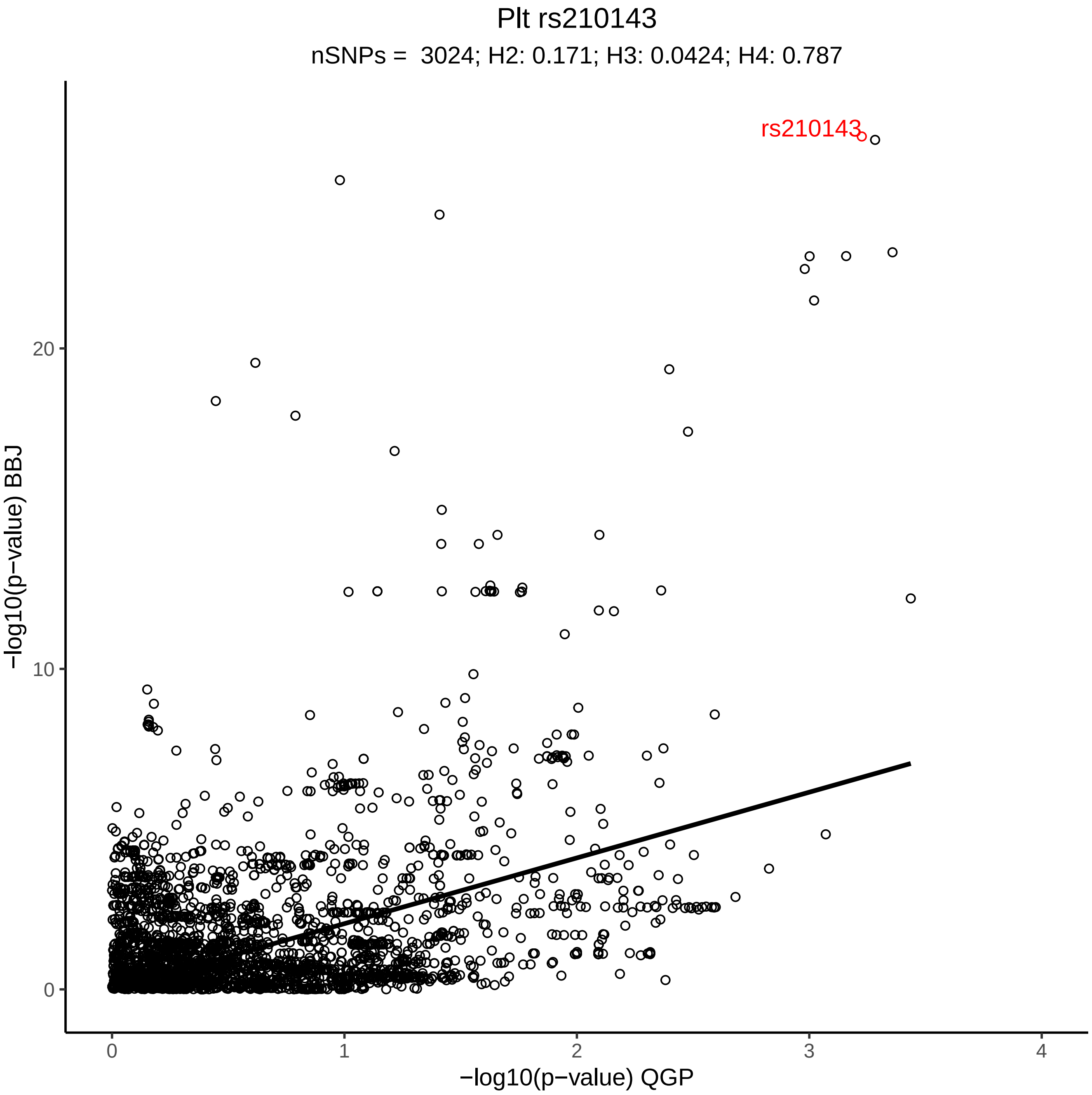
Plt rs210143
nSNPs = 3024; H2: 0.171; H3: 0.0424; H4: 0.787
rs210143
−log10(p−value) BBJ
−log10(p−value) QGP
0
10
20
0
1
2
3
4

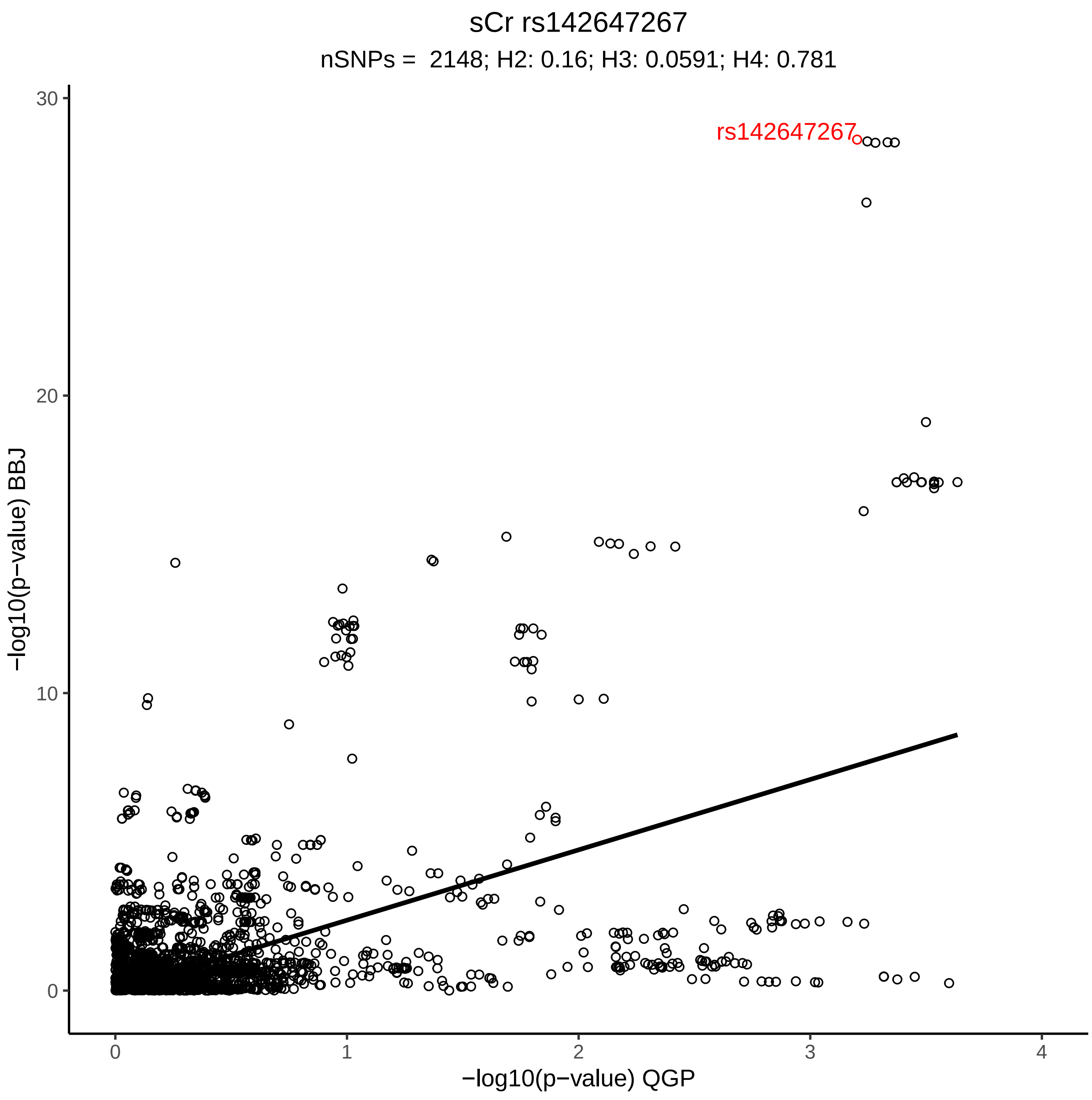

sCr rs142647267
nSNPs = 2148; H2: 0.16; H3: 0.0591; H4: 0.781
rs142647267
30
20
10
0
−log10(p−value) BBJ
0
1
2
3
4
−log10(p−value) QGP

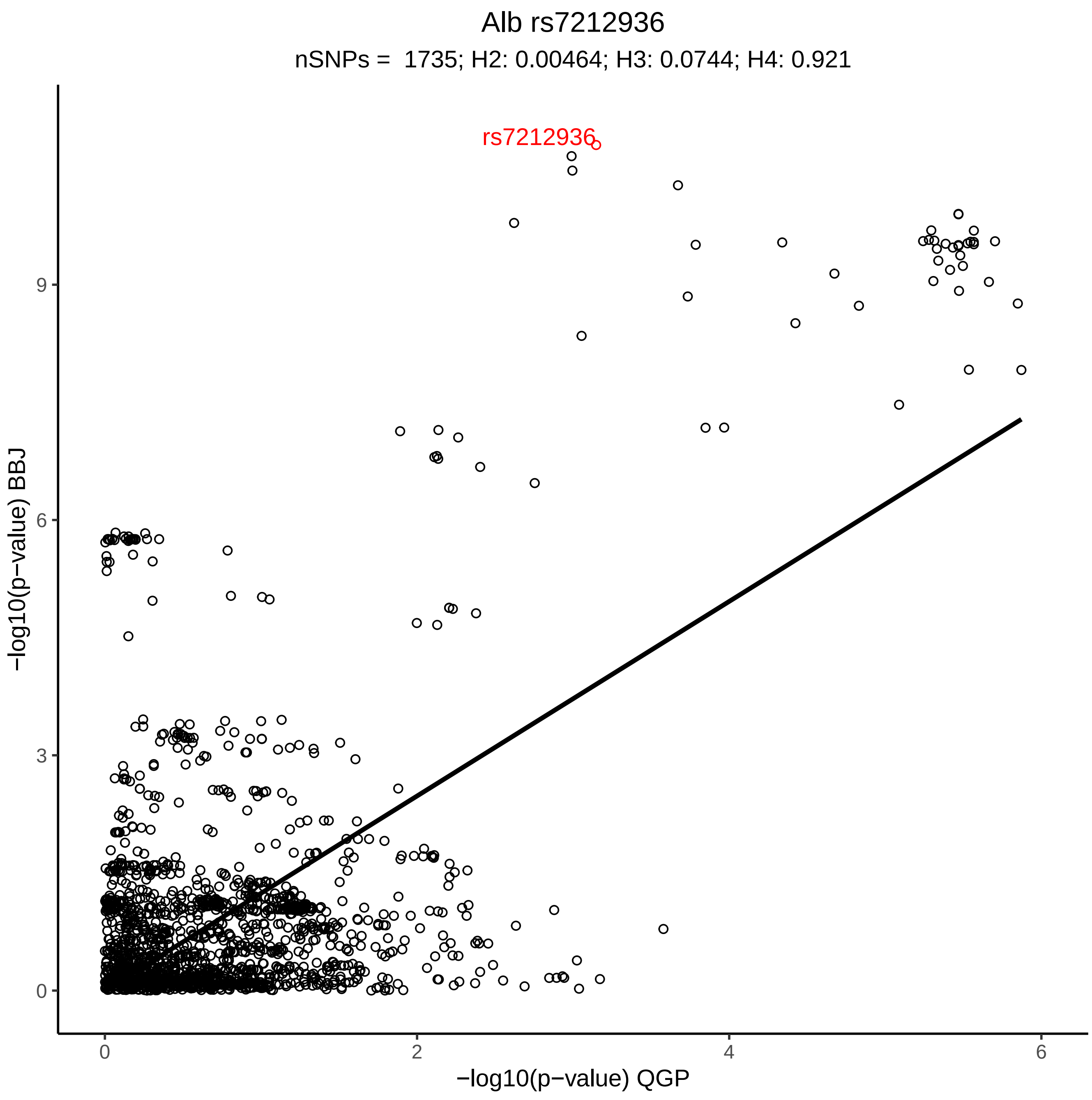
Alb rs7212936
nSNPs = 1735; H2: 0.00464; H3: 0.0744; H4: 0.921
rs7212936
−log10(p−value) BBJ
−log10(p−value) QGP
0
3
6
9
0
2
4
6

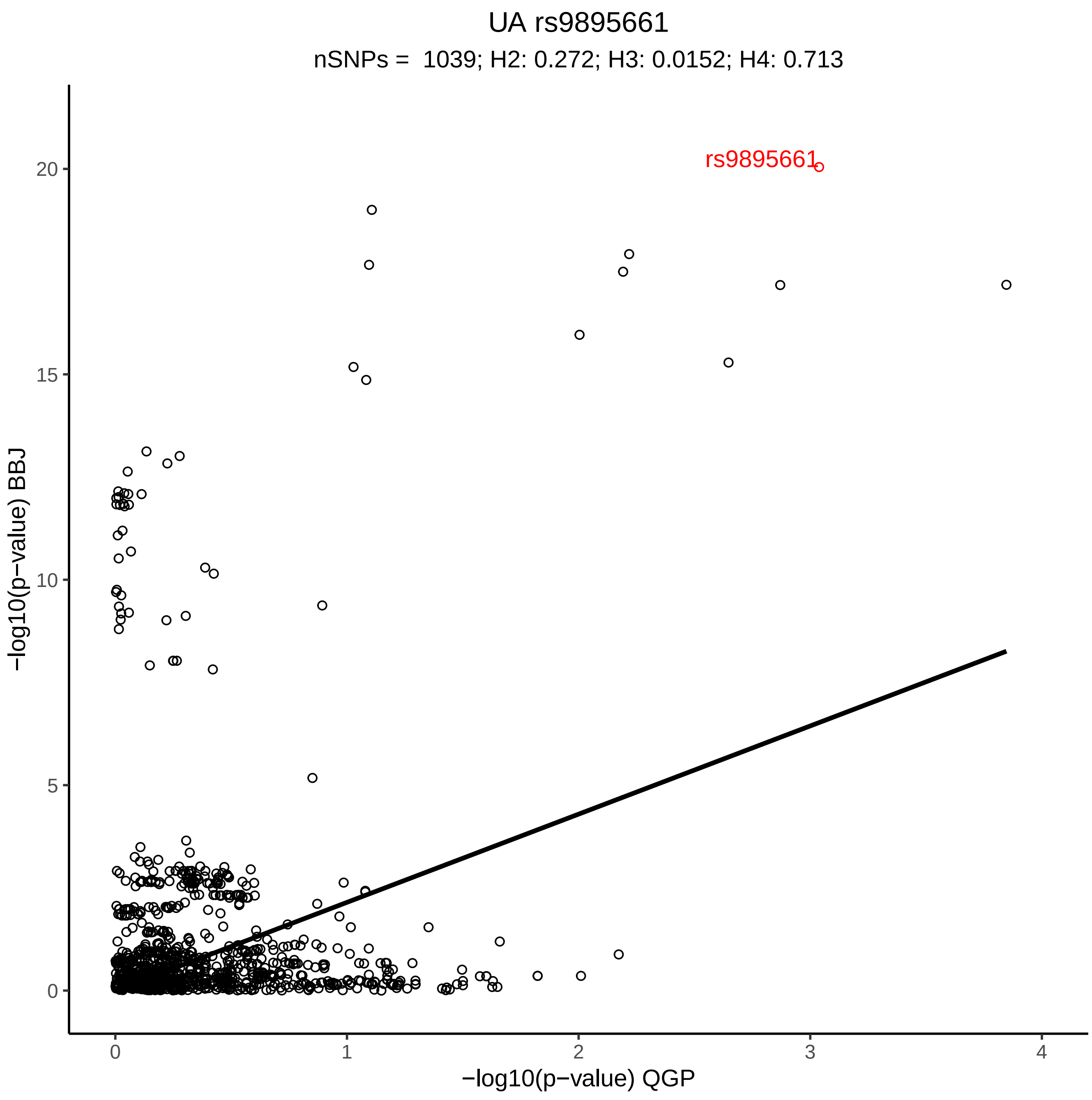
UA rs9895661
nSNPs = 1039; H2: 0.272; H3: 0.0152; H4: 0.713
rs9895661
−log10(p−value) BBJ
−log10(p−value) QGP
0
5
10
15
20
0
1
2
3
4

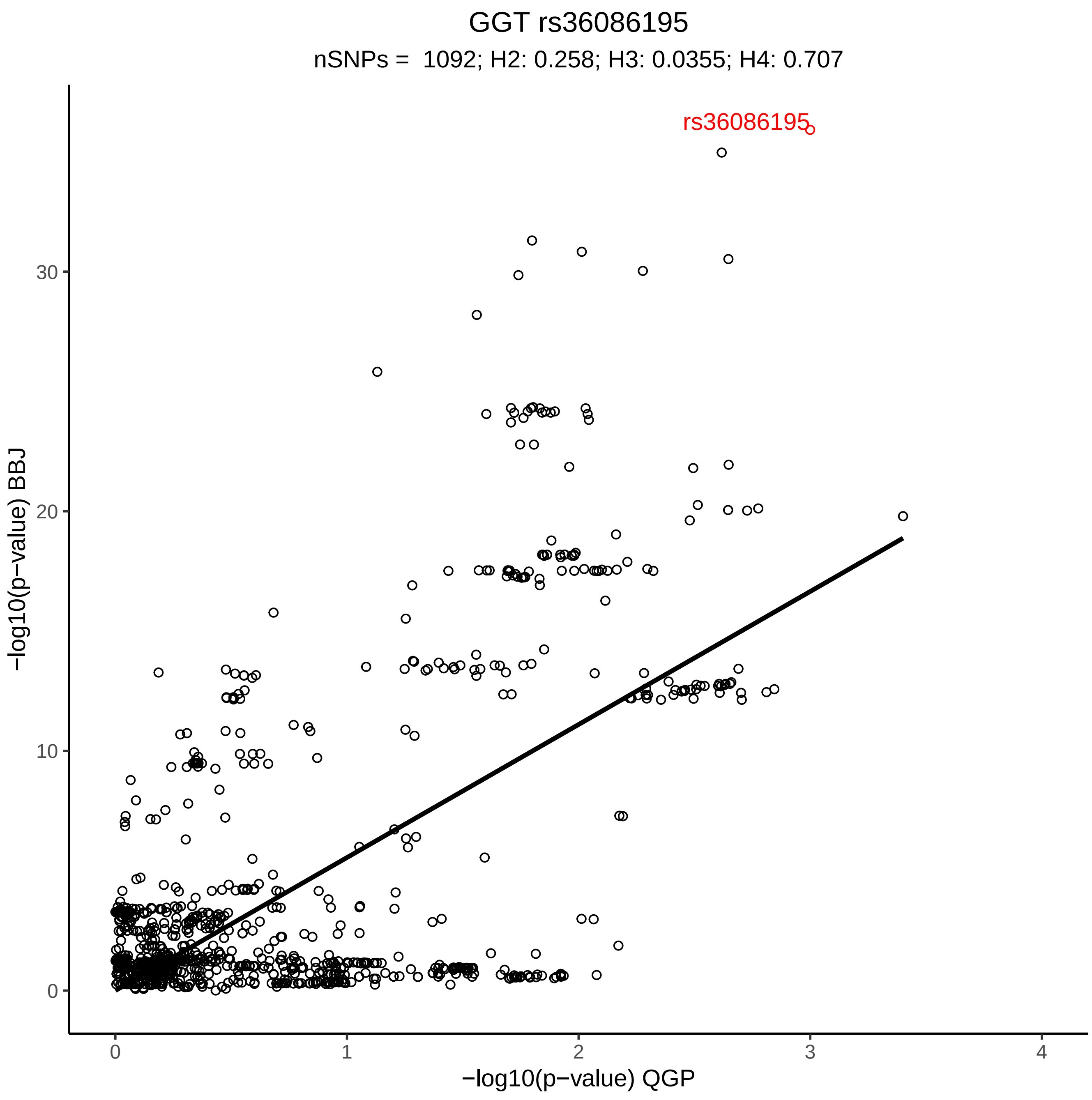
GGT rs36086195
nSNPs = 1092; H2: 0.258; H3: 0.0355; H4: 0.707
rs36086195
30
20
10
0
0
1
2
3
4
−log10(p−value) BBJ
−log10(p−value) QGP

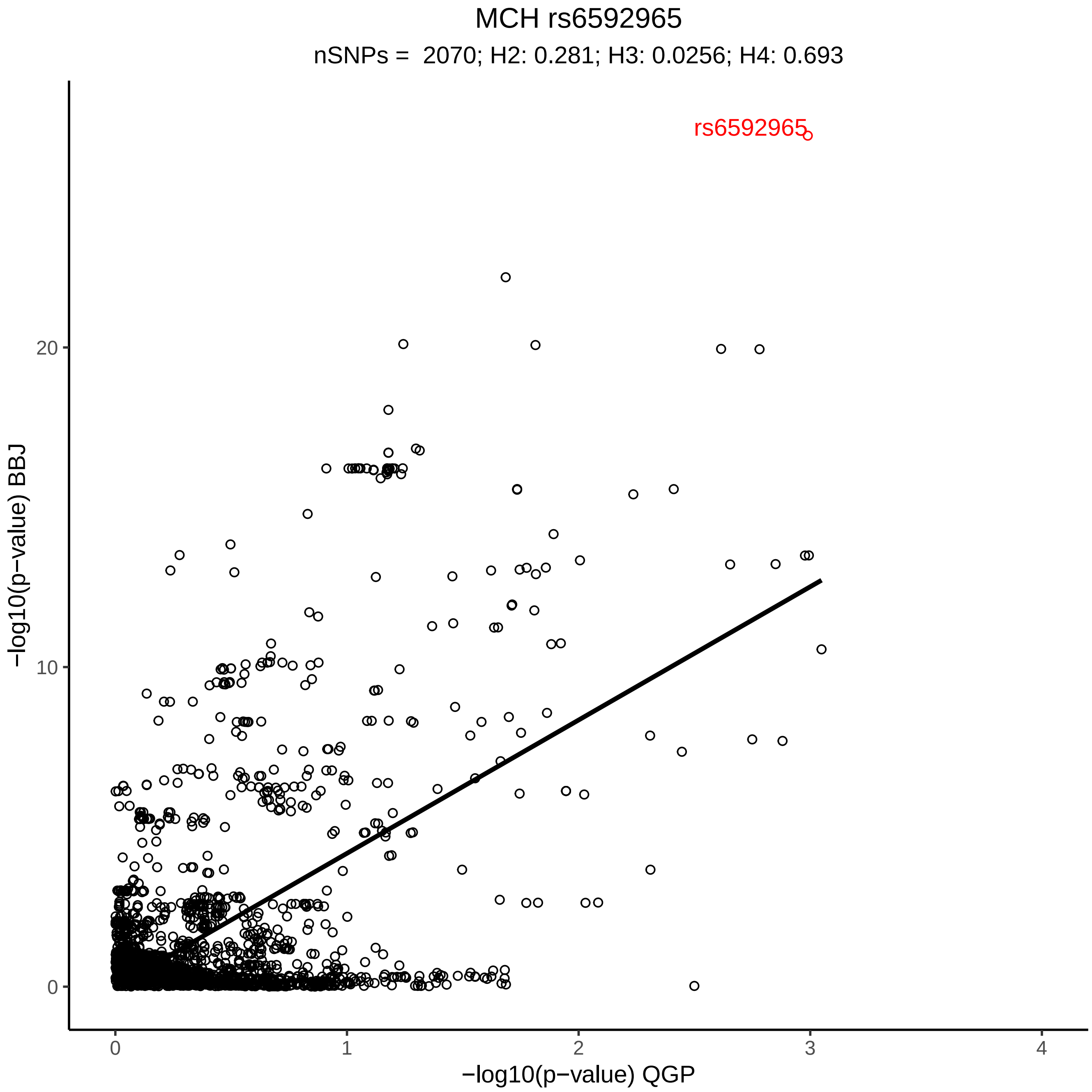
MCH rs6592965
nSNPs = 2070; H2: 0.281; H3: 0.0256; H4: 0.693
rs6592965
20
10
0
−log10(p−value) BBJ
0
1
2
3
4
−log10(p−value) QGP

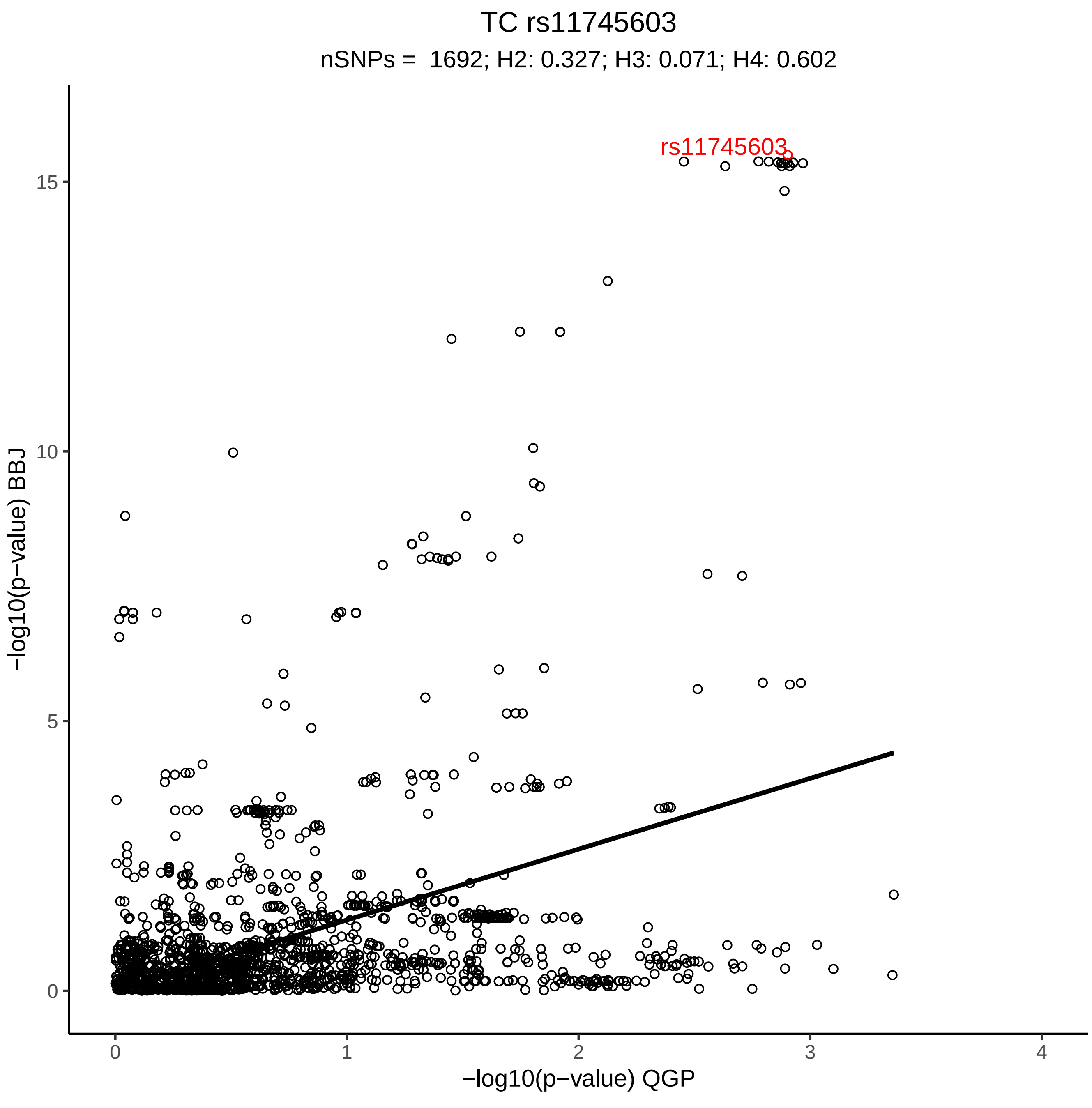
TC rs11745603
nSNPs = 1692; H2: 0.327; H3: 0.071; H4: 0.602
rs11745603
−log10(p−value) BBJ
−log10(p−value) QGP
0
5
10
15
0
1
2
3
4

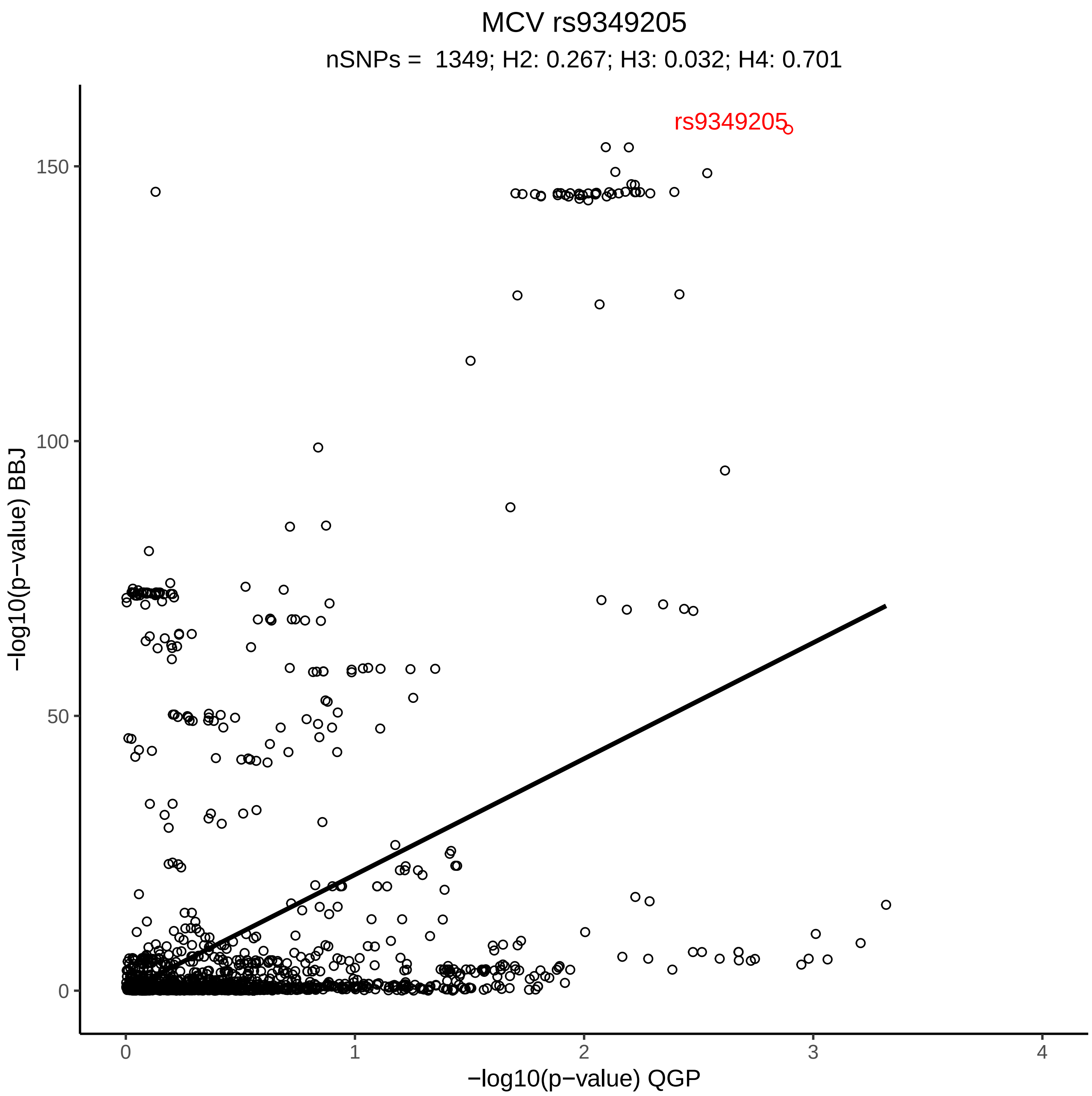

MCV rs9349205
nSNPs = 1349; H2: 0.267; H3: 0.032; H4: 0.701
rs9349205
150
100
50
0
−log10(p−value) BBJ
0
1
2
3
4
−log10(p−value) QGP

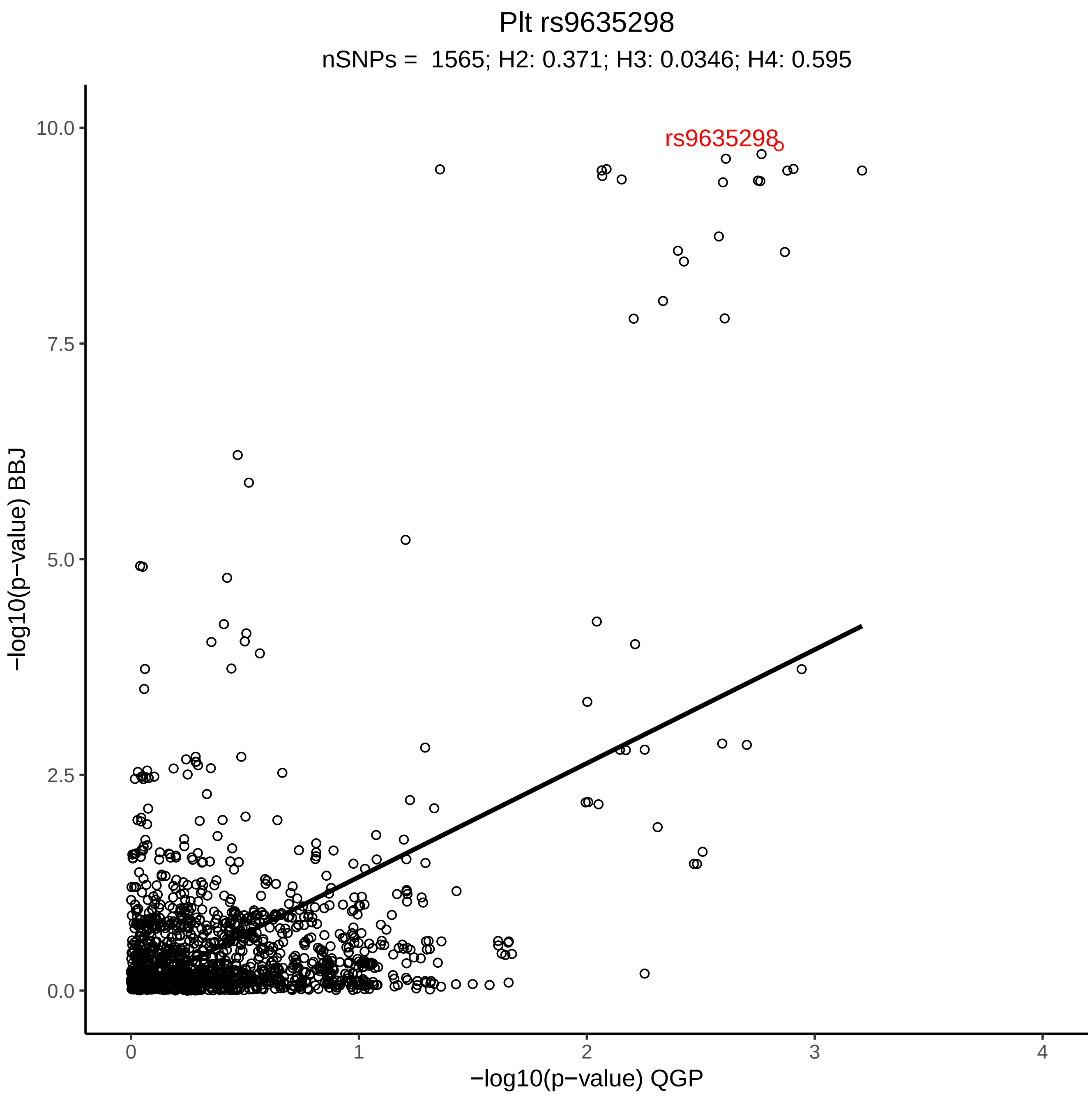
Plt rs9635298
nSNPs = 1565; H2: 0.371; H3: 0.0346; H4: 0.595
rs9635298
−log10(p−value) BBJ
−log10(p−value) QGP
10.0
7.5
5.0
2.5
0.0
0
1
2
3
4

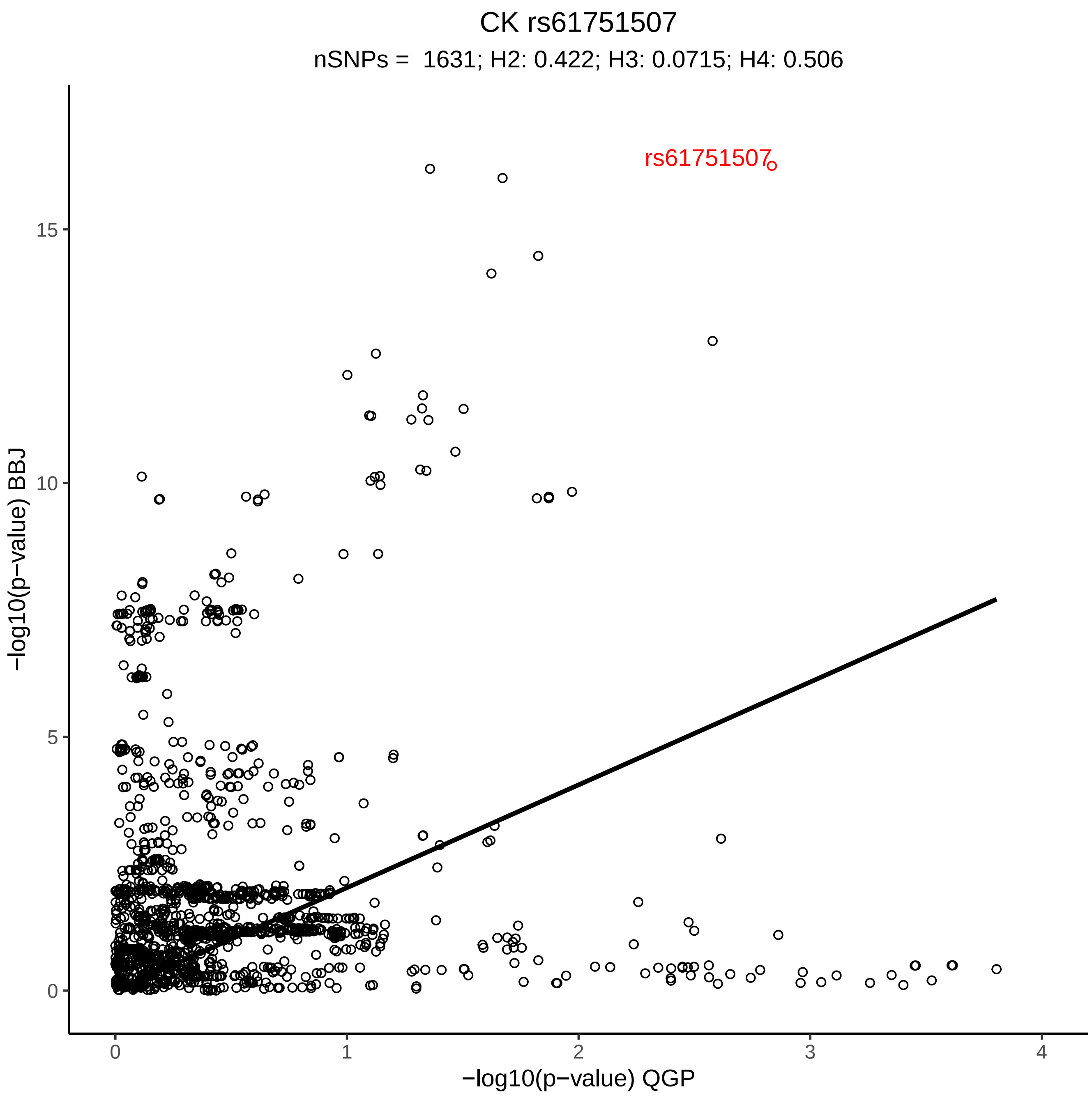
CK rs61751507
nSNPs = 1631; H2: 0.422; H3: 0.0715; H4: 0.506
rs61751507
15
10
5
0
−log10(p−value) BBJ
0
1
2
3
4
−log10(p−value) QGP

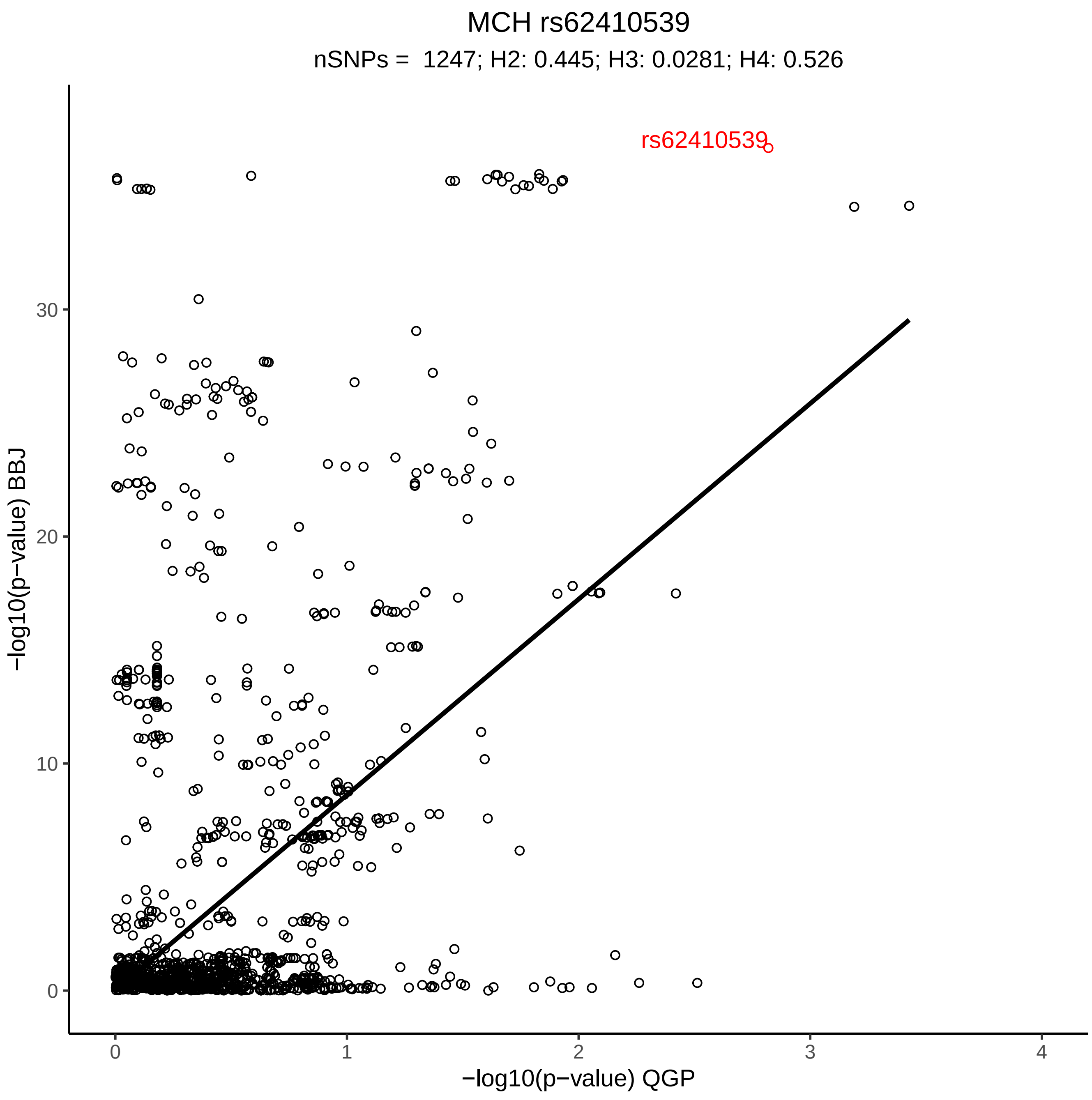

MCH rs62410539
nSNPs = 1247; H2: 0.445; H3: 0.0281; H4: 0.526
rs62410539
−log10(p−value) BBJ
−log10(p−value) QGP
0
10
20
30
0
1
2
3
4

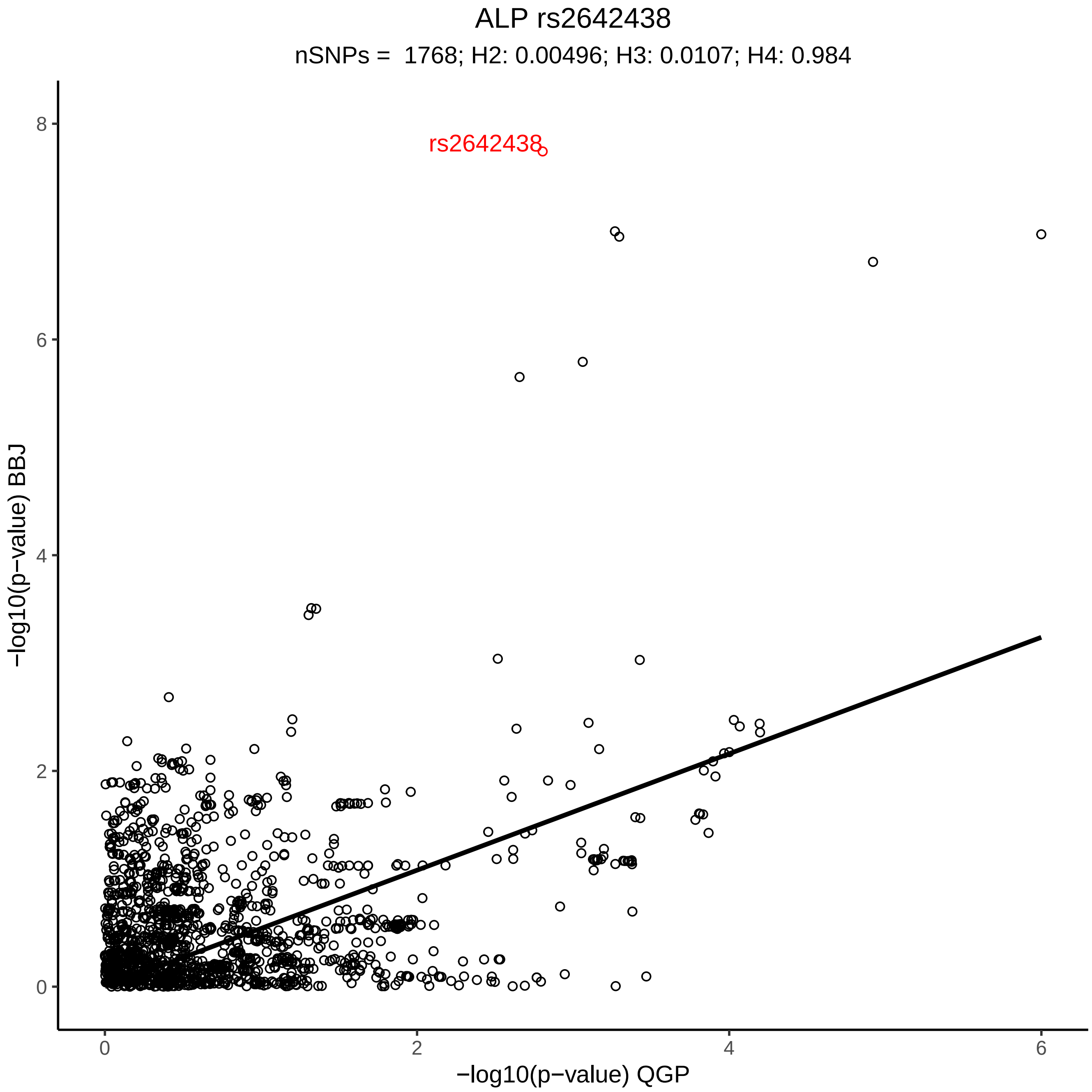

ALP rs2642438
nSNPs = 1768; H2: 0.00496; H3: 0.0107; H4: 0.984
rs2642438
−log10(p−value) BBJ
−log10(p−value) QGP
0
2
4
6
8
0
2
4
6

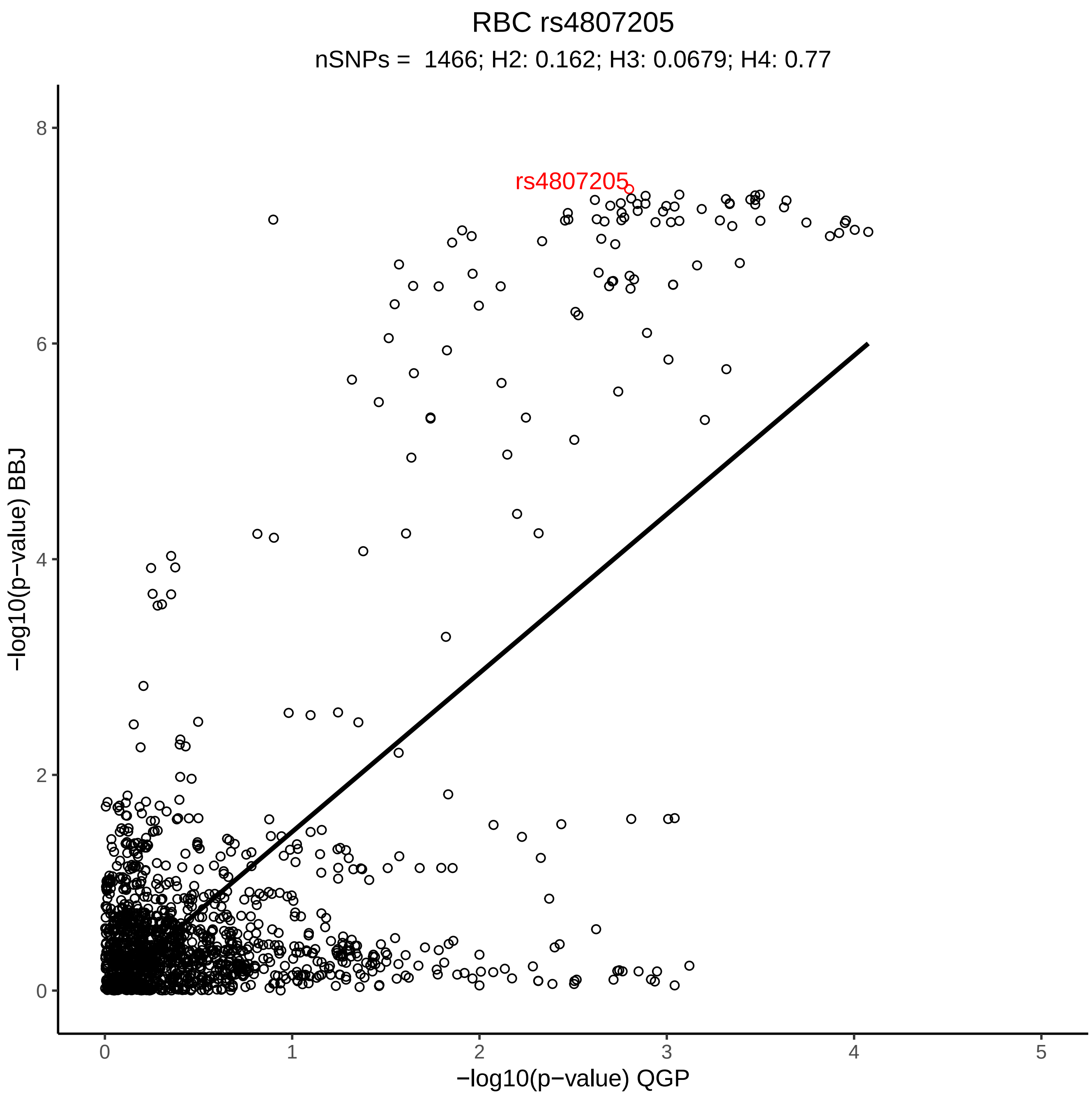
RBC rs4807205
nSNPs = 1466; H2: 0.162; H3: 0.0679; H4: 0.77
rs4807205
−log10(p−value) BBJ
−log10(p−value) QGP
0
2
4
6
8
0
1
2
3
4
5

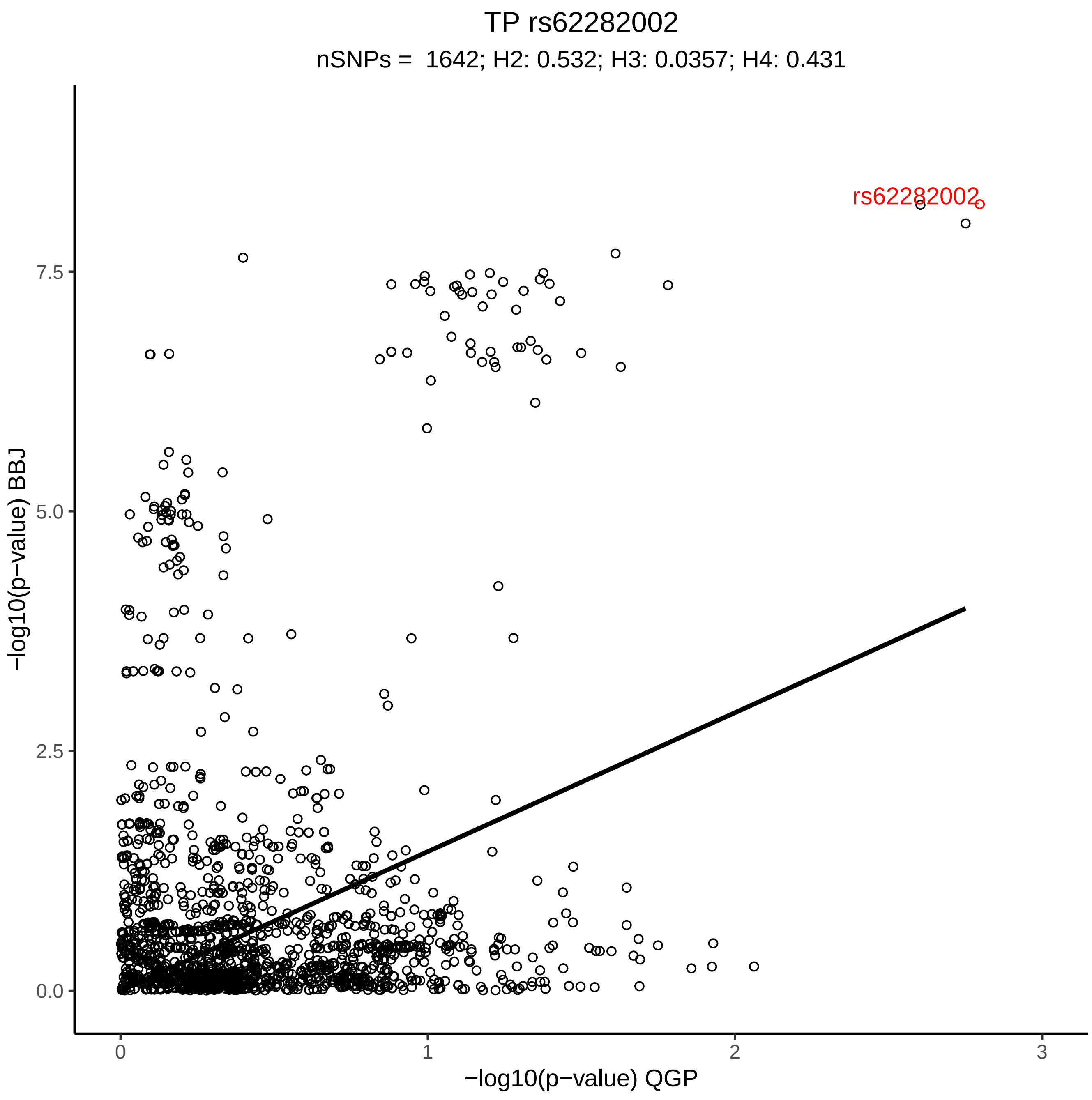
TP rs62282002
nSNPs = 1642; H2: 0.532; H3: 0.0357; H4: 0.431
rs62282002
7.5
5.0
2.5
0.0
−log10(p−value) BBJ
0
1
2
3
−log10(p−value) QGP

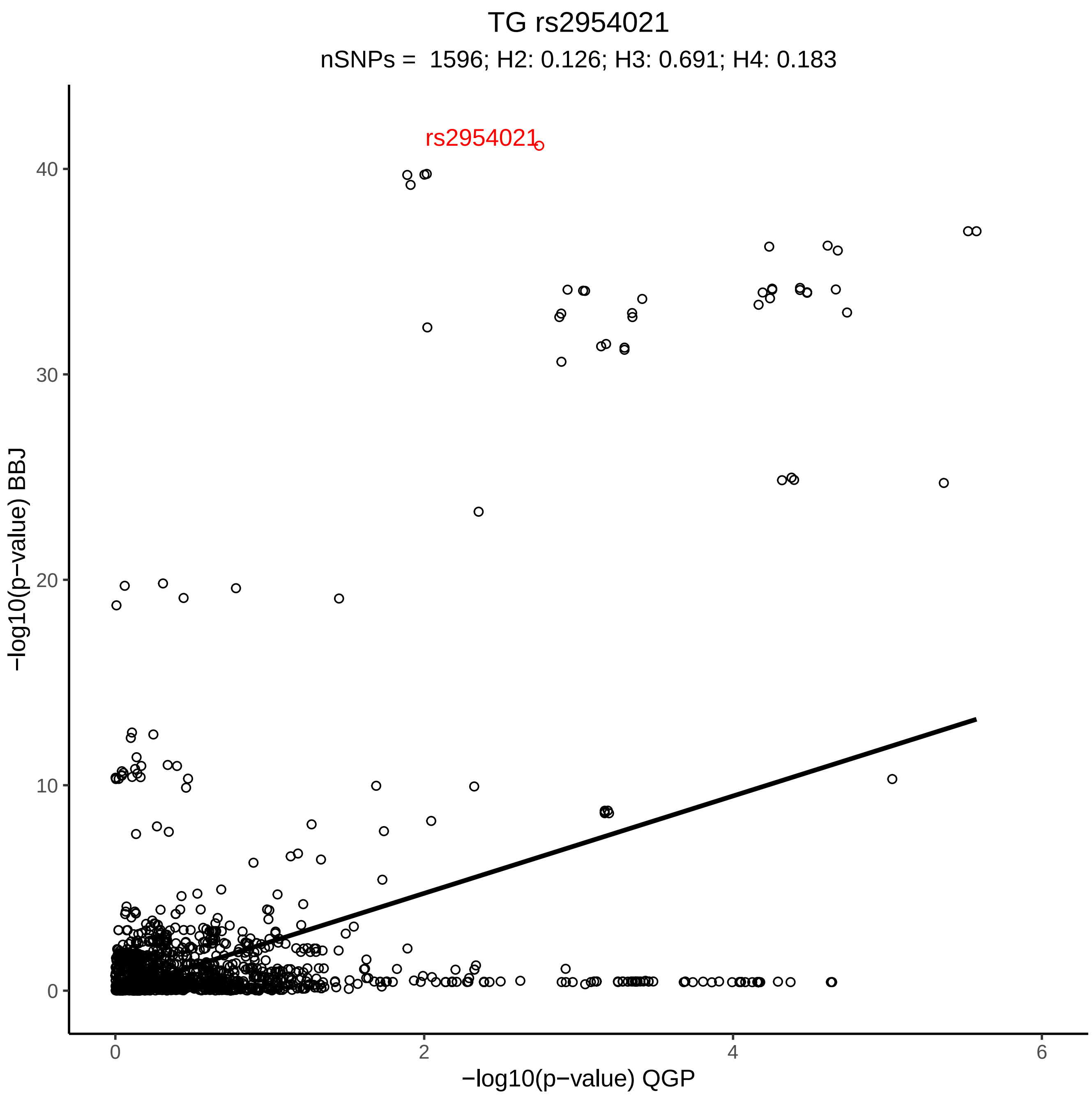
TG rs2954021
nSNPs = 1596; H2: 0.126; H3: 0.691; H4: 0.183
rs2954021
−log10(p−value) BBJ
−log10(p−value) QGP
0
10
20
30
40
0
2
4
6

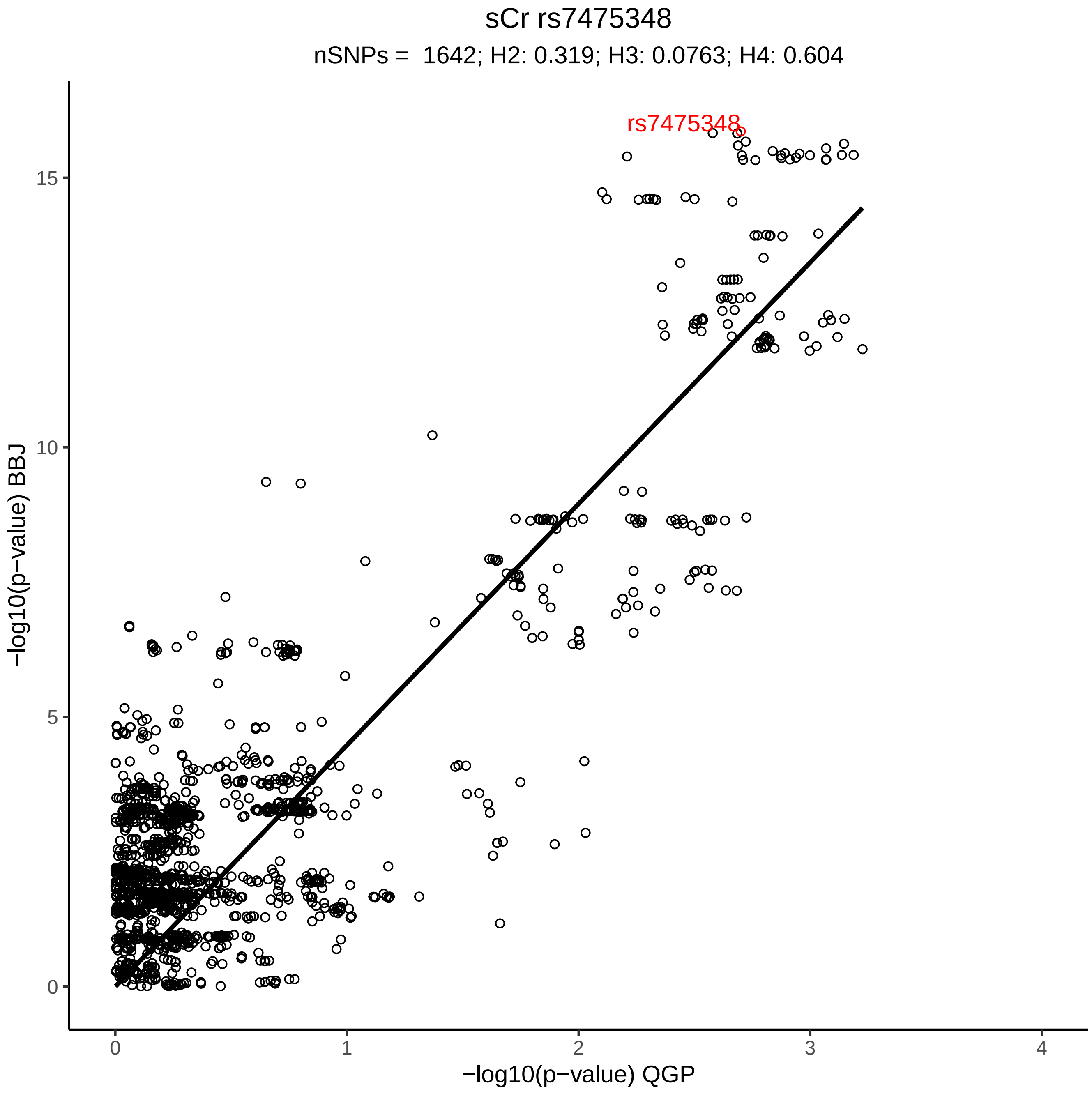
sCr rs7475348
nSNPs = 1642; H2: 0.319; H3: 0.0763; H4: 0.604
rs7475348
−log10(p−value) BBJ
−log10(p−value) QGP
0
5
10
15
0
1
2
3
4

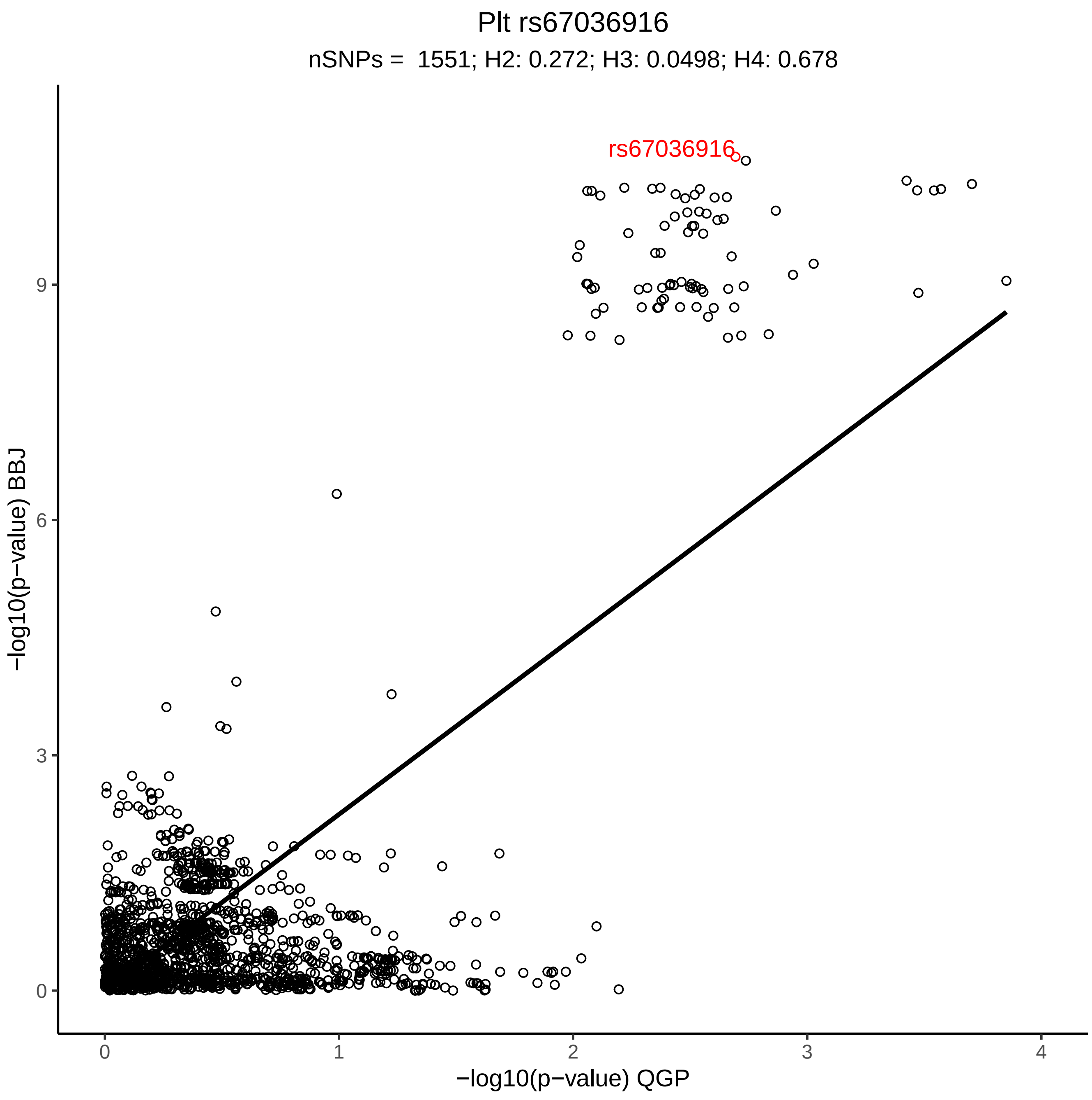

Plt rs67036916
nSNPs = 1551; H2: 0.272; H3: 0.0498; H4: 0.678
rs67036916
−log10(p−value) BBJ
−log10(p−value) QGP
0
3
6
9
0
1
2
3
4

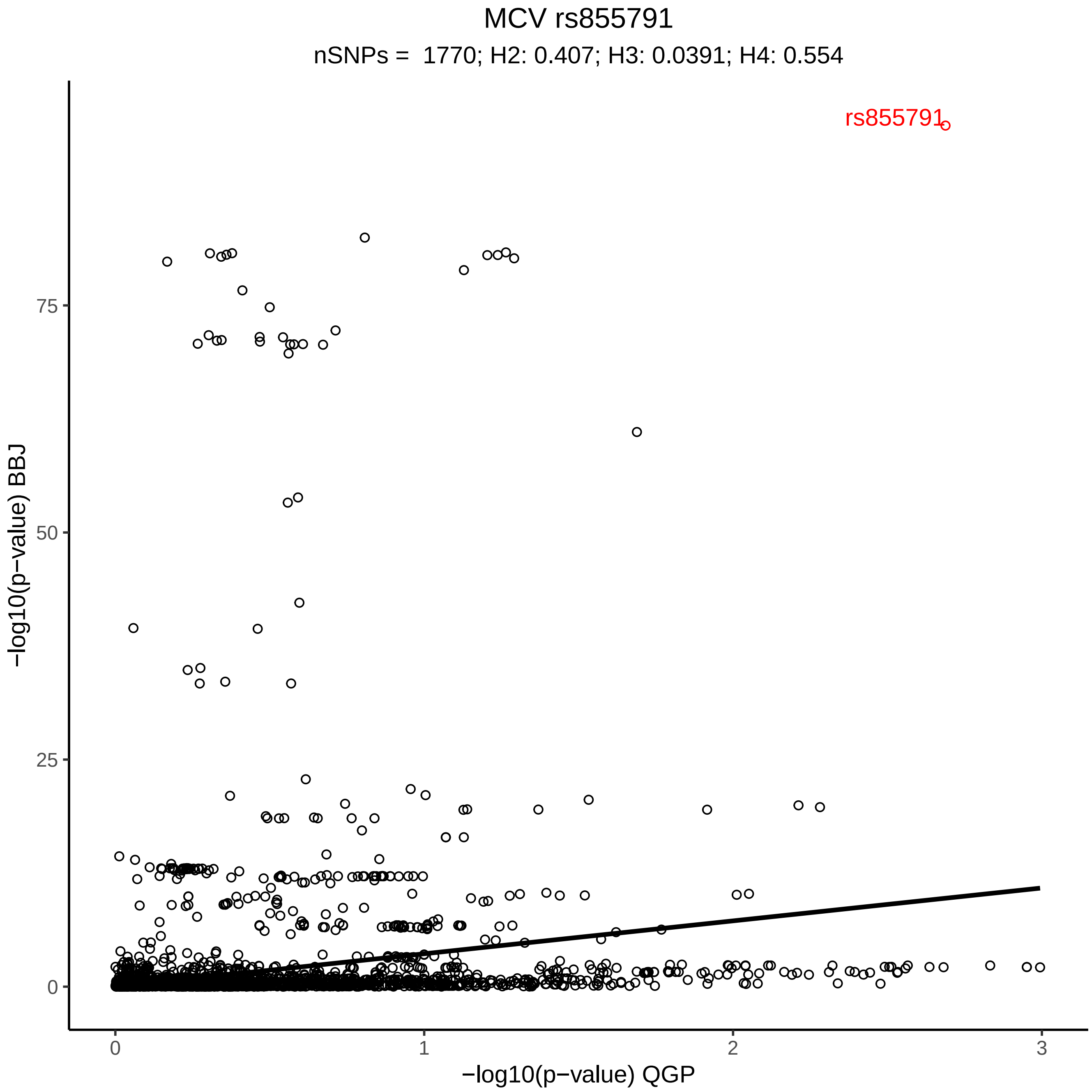

MCV rs855791
nSNPs = 1770; H2: 0.407; H3: 0.0391; H4: 0.554
rs855791
−log10(p−value) BBJ
0
25
50
75
0
1
2
3
−log10(p−value) QGP

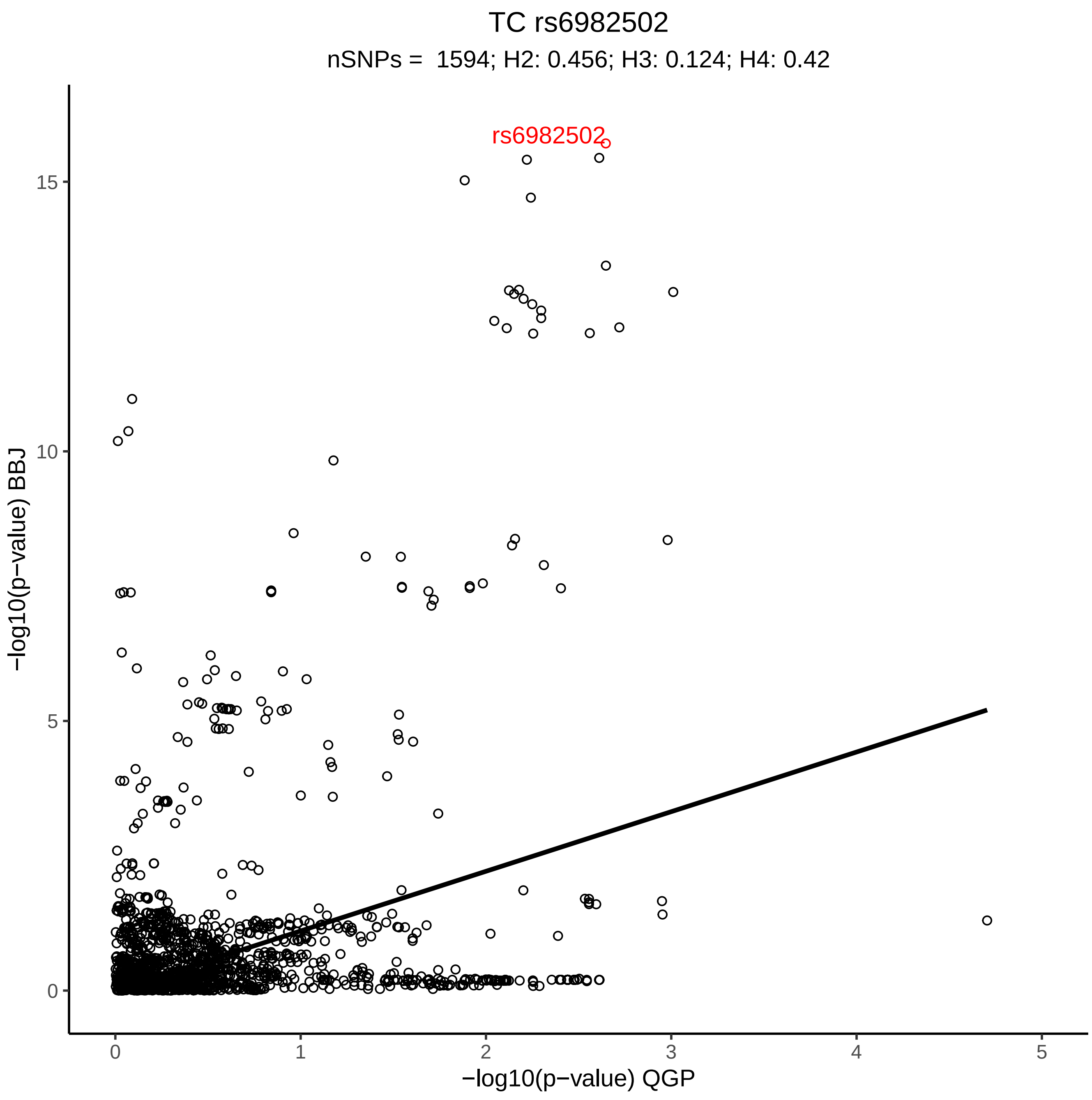
TC rs6982502
nSNPs = 1594; H2: 0.456; H3: 0.124; H4: 0.42
rs6982502
15
10
5
0
0
1
2
3
4
5
−log10(p−value) BBJ
−log10(p−value) QGP

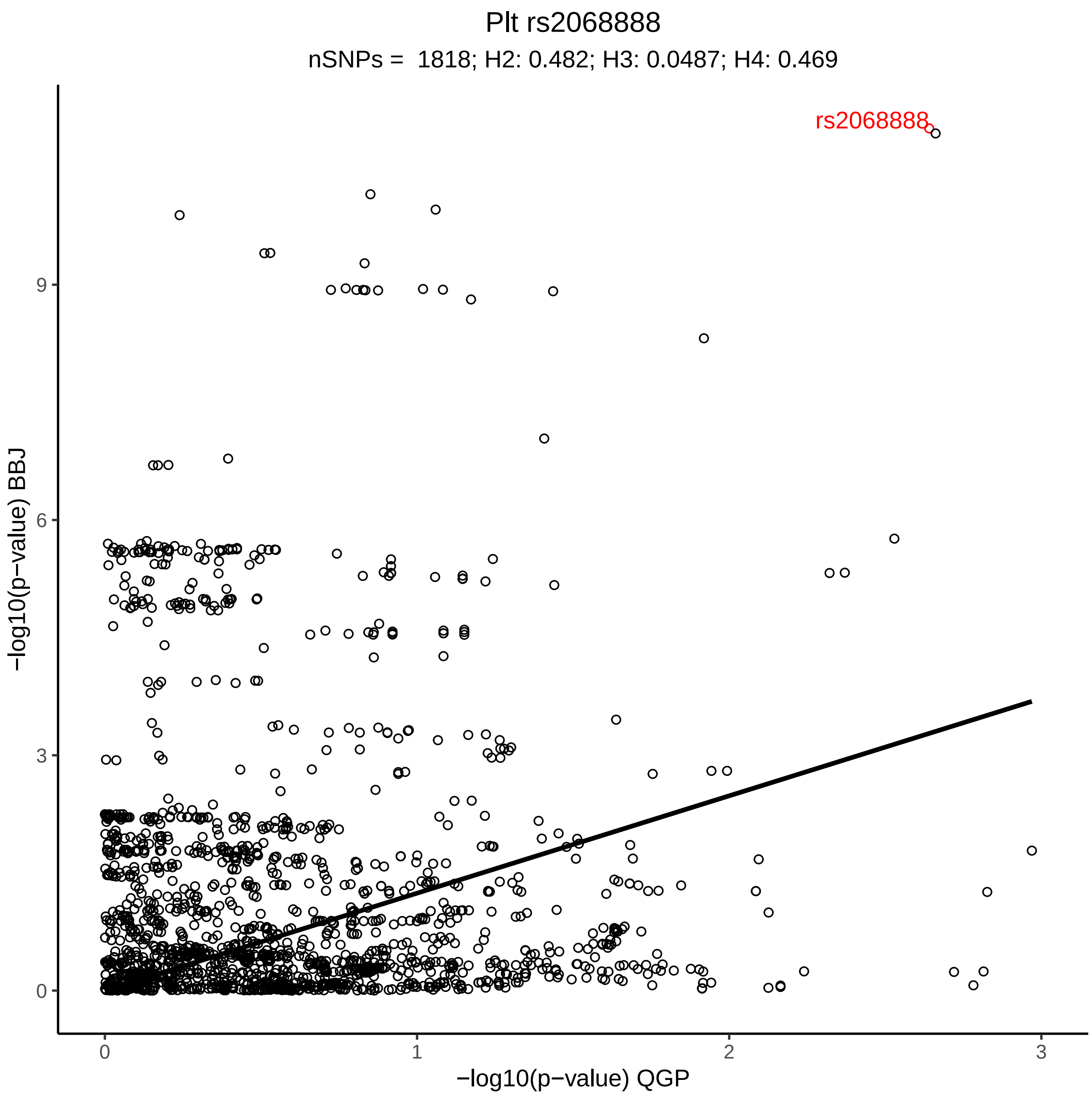

Plt rs2068888
nSNPs = 1818; H2: 0.482; H3: 0.0487; H4: 0.469
rs2068888
−log10(p−value) BBJ
0
3
6
9
0
1
2
3
−log10(p−value) QGP

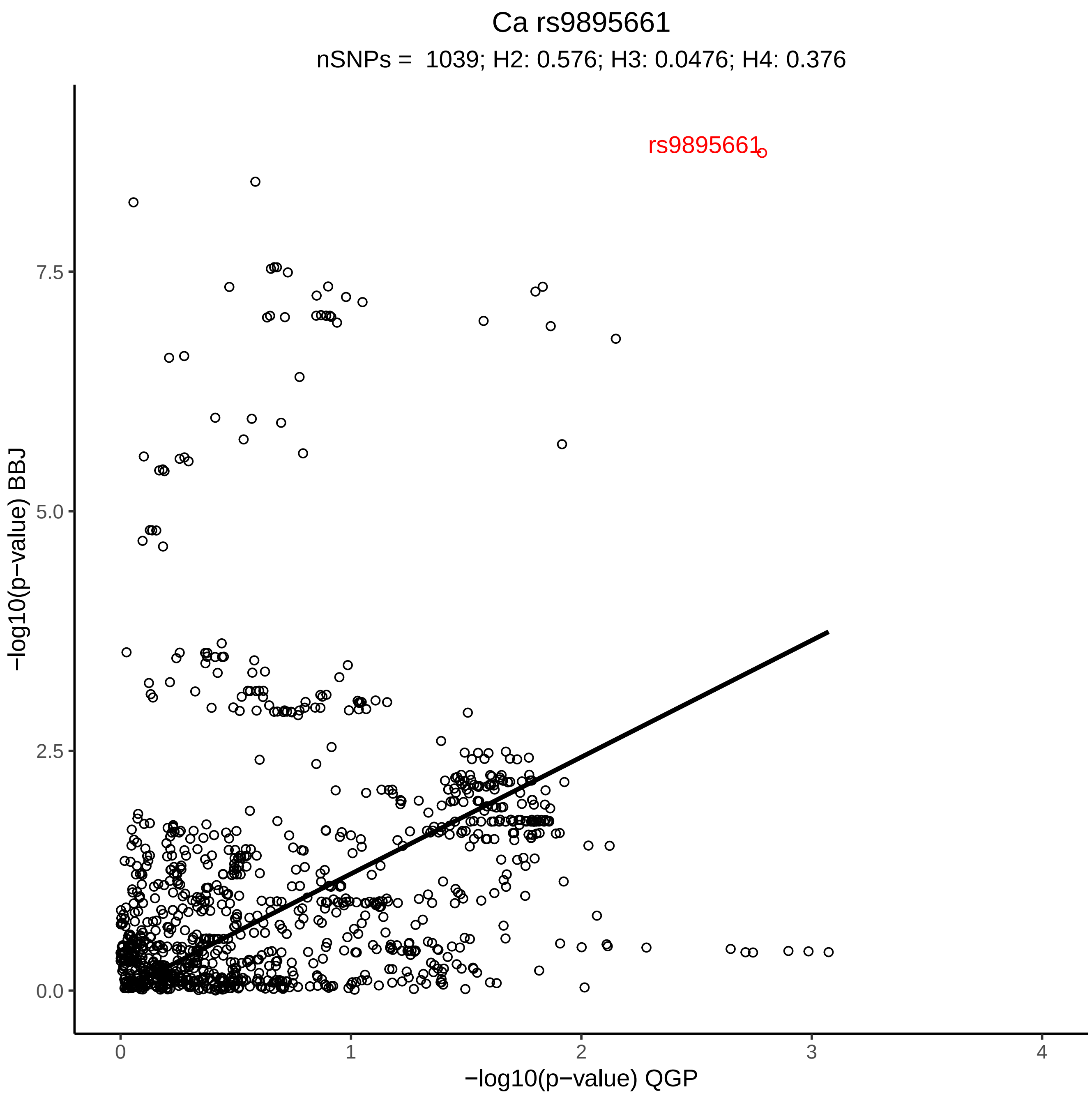
Ca rs9895661
nSNPs = 1039; H2: 0.576; H3: 0.0476; H4: 0.376
rs9895661
−log10(p−value) BBJ
7.5
5.0
2.5
0.0
0
1
2
3
4
−log10(p−value) QGP

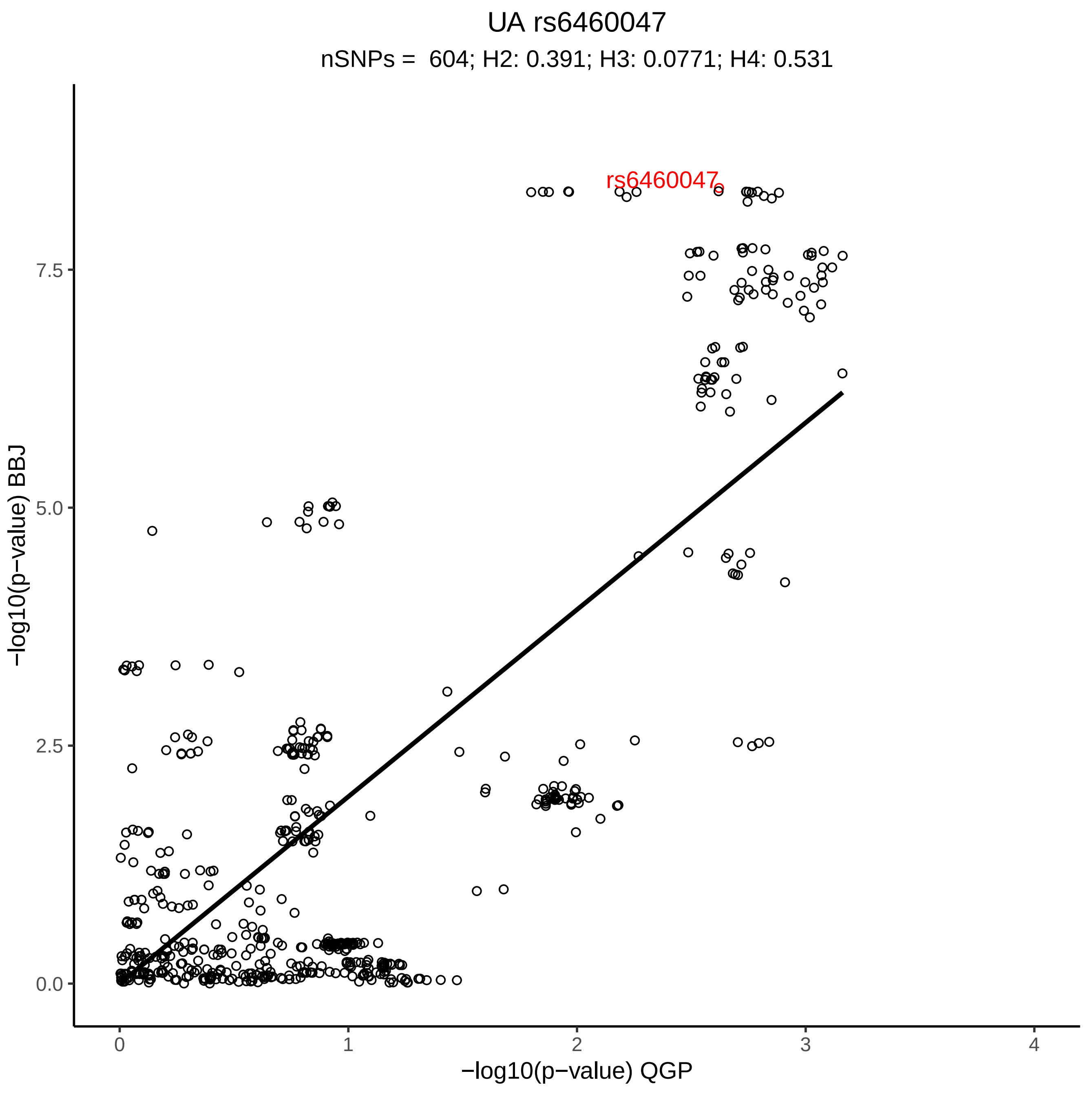

UA rs6460047
nSNPs = 604; H2: 0.391; H3: 0.0771; H4: 0.531
rs6460047
7.5
5.0
2.5
0.0
−log10(p−value) BBJ
0
1
2
3
4
−log10(p−value) QGP

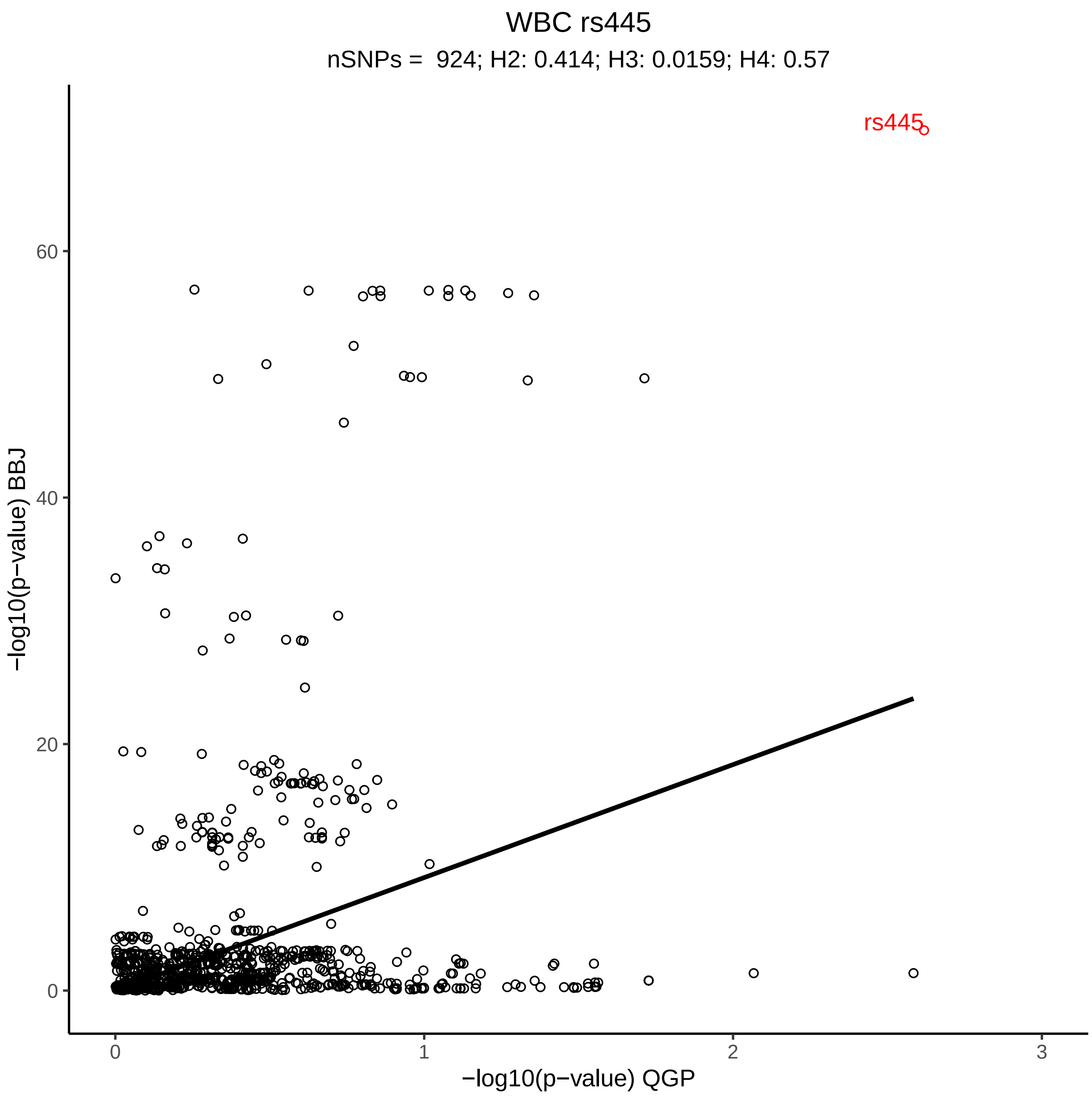
WBC rs445
nSNPs = 924; H2: 0.414; H3: 0.0159; H4: 0.57
rs445
−log10(p−value) BBJ
0
20
40
60
0
1
2
3
−log10(p−value) QGP

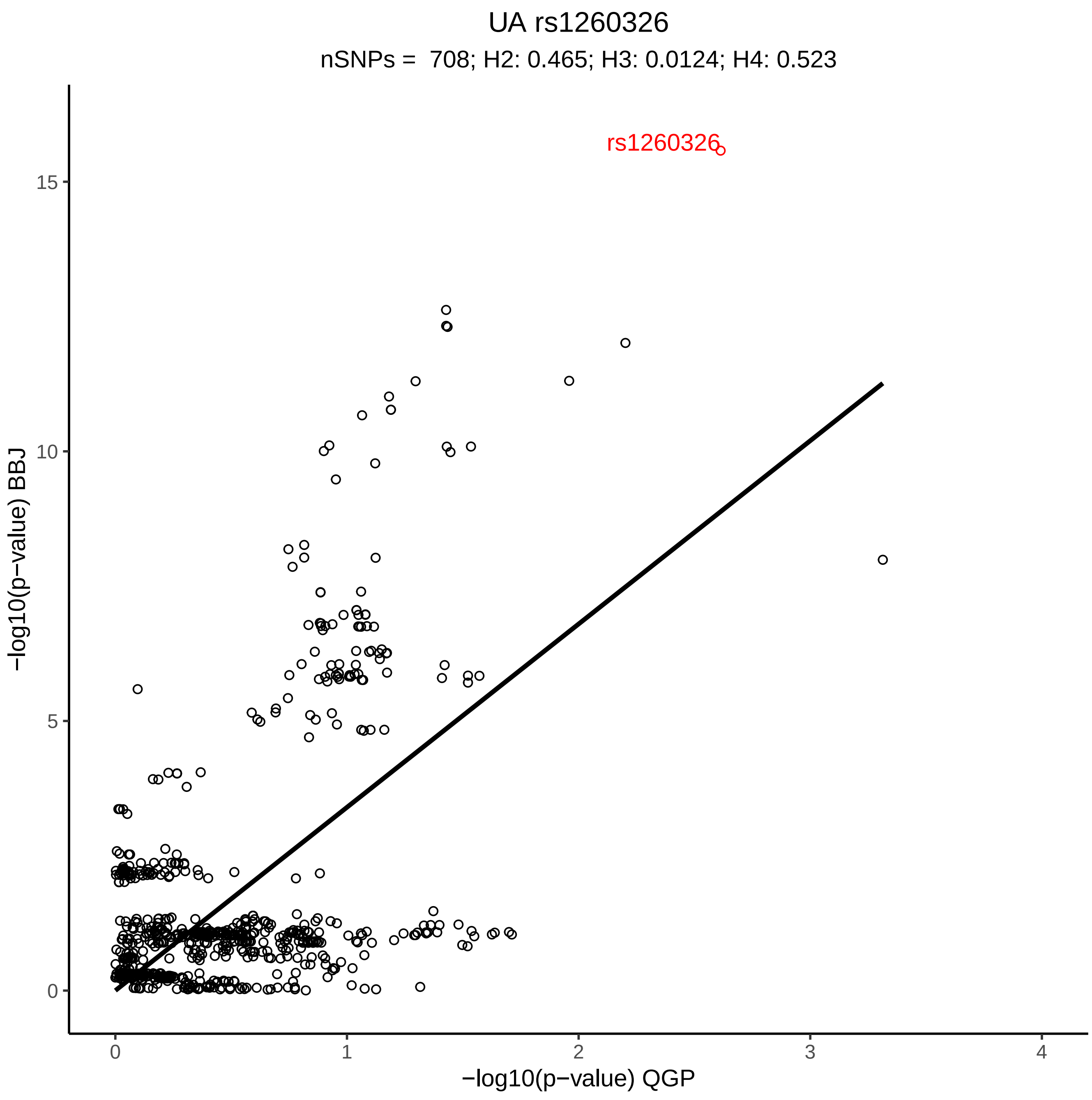

UA rs1260326
nSNPs = 708; H2: 0.465; H3: 0.0124; H4: 0.523
rs1260326
15
10
5
0
0
1
2
3
4
−log10(p−value) BBJ
−log10(p−value) QGP

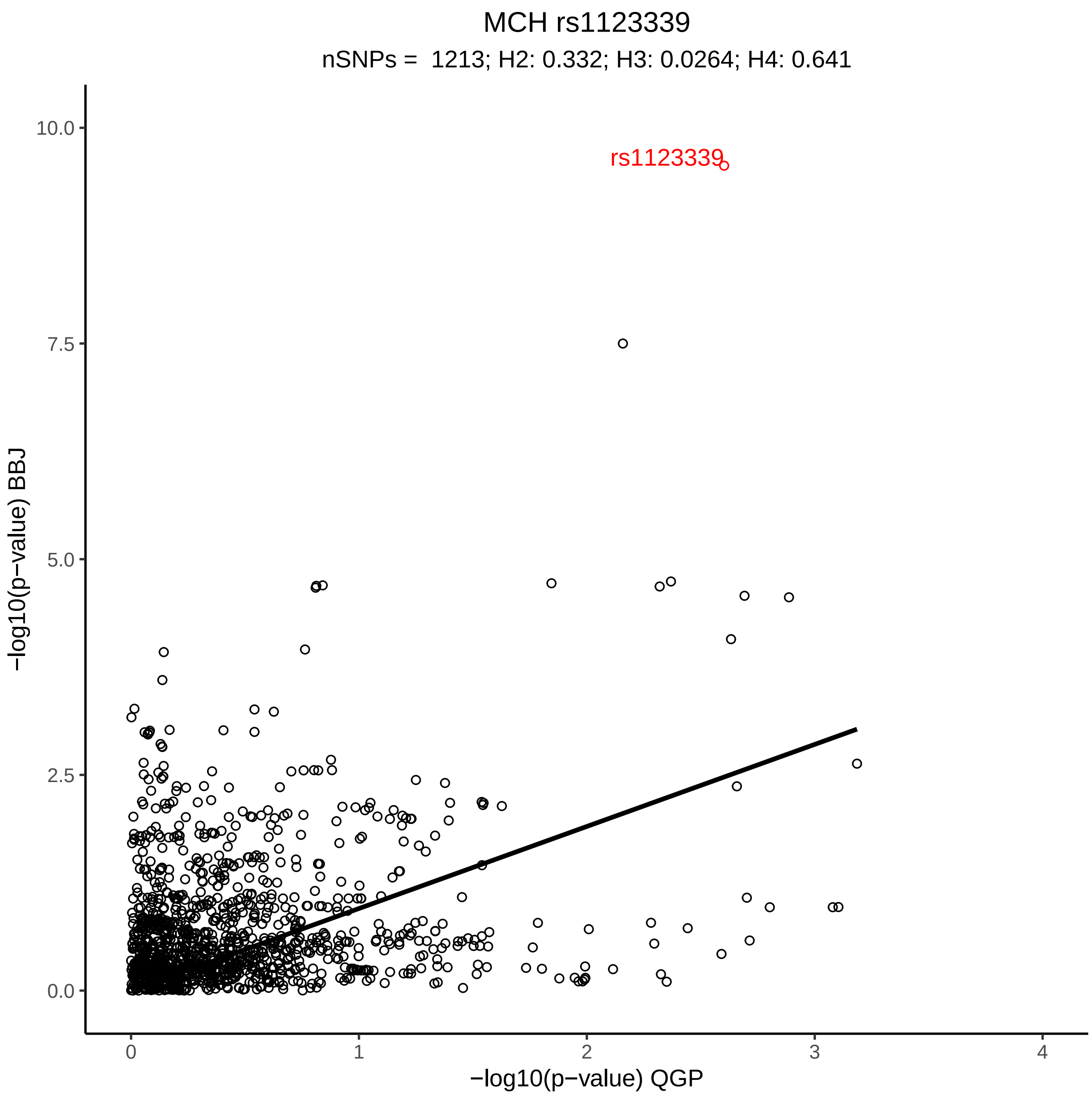

MCH rs1123339
nSNPs = 1213; H2: 0.332; H3: 0.0264; H4: 0.641
rs1123339
10.0
7.5
5.0
2.5
0.0
−log10(p−value) BBJ
0
1
2
3
4
−log10(p−value) QGP

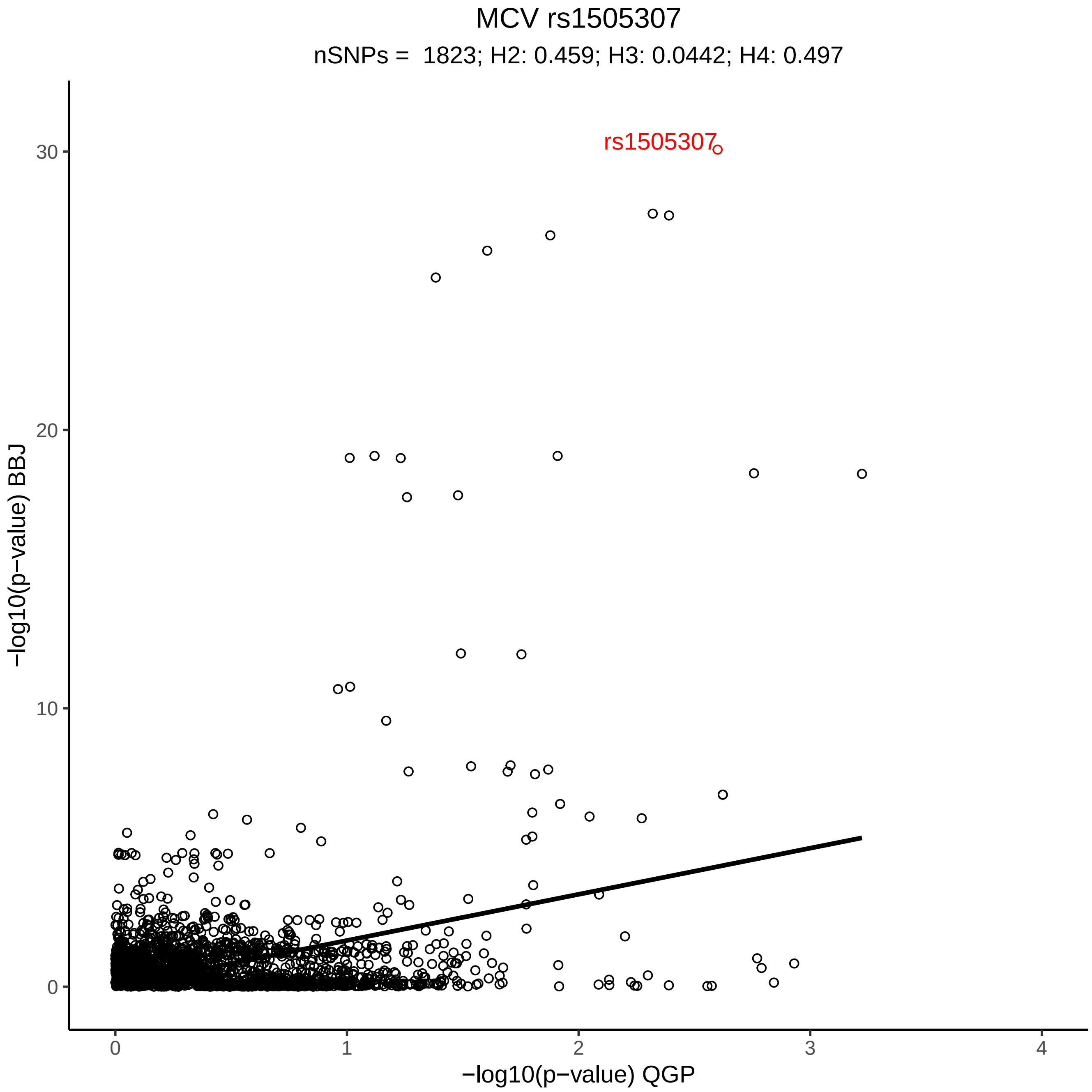
MCV rs1505307
nSNPs = 1823; H2: 0.459; H3: 0.0442; H4: 0.497
rs1505307
−log10(p−value) BBJ
−log10(p−value) QGP
0
10
20
30
0
1
2
3
4

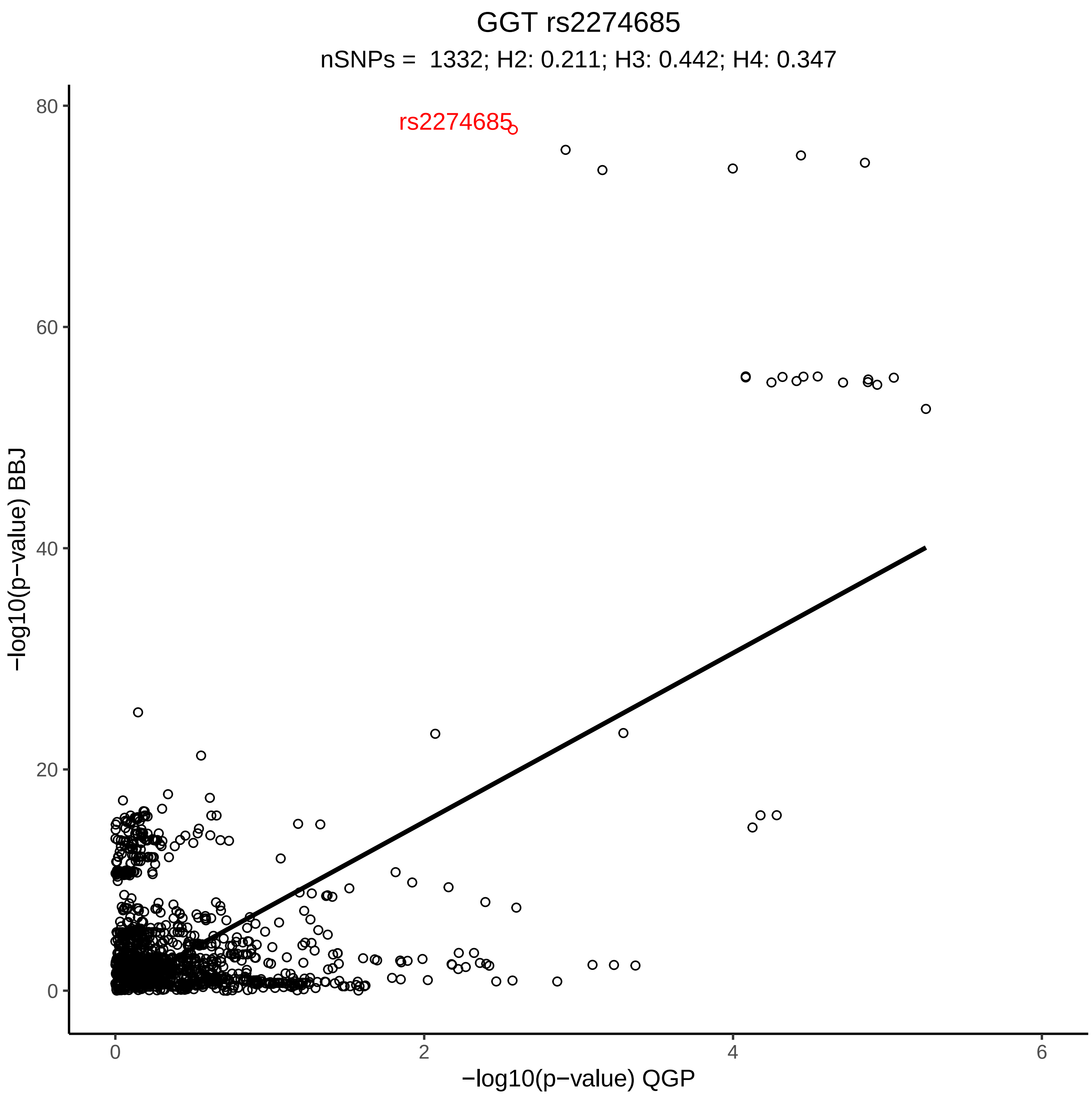

GGT rs2274685
nSNPs = 1332; H2: 0.211; H3: 0.442; H4: 0.347
rs2274685
80
60
40
20
0
0
2
4
6
−log10(p−value) BBJ
−log10(p−value) QGP

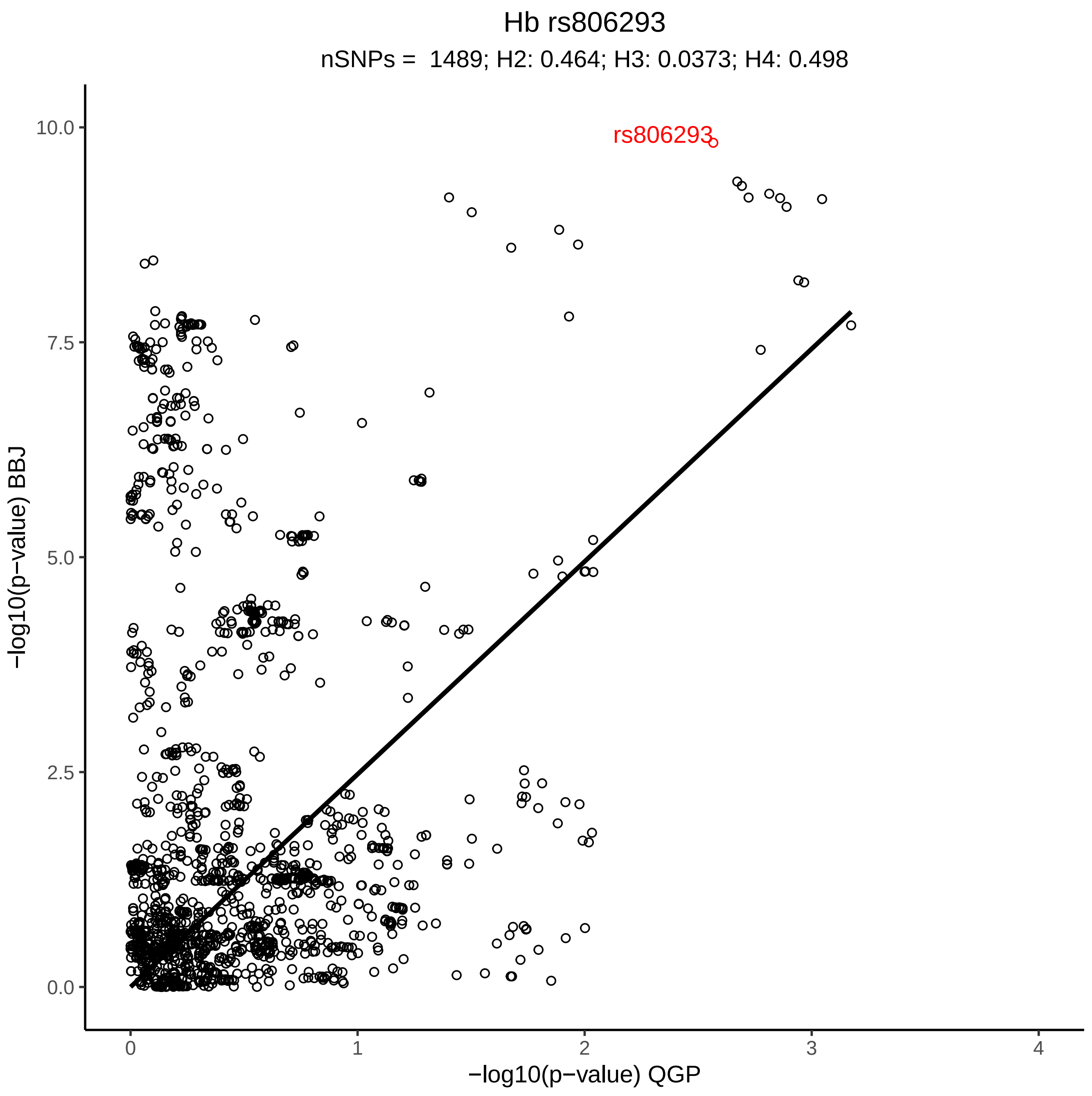
Hb rs806293
nSNPs = 1489; H2: 0.464; H3: 0.0373; H4: 0.498
rs806293
−log10(p−value) BBJ
−log10(p−value) QGP
10.0
7.5
5.0
2.5
0.0
0
1
2
3
4

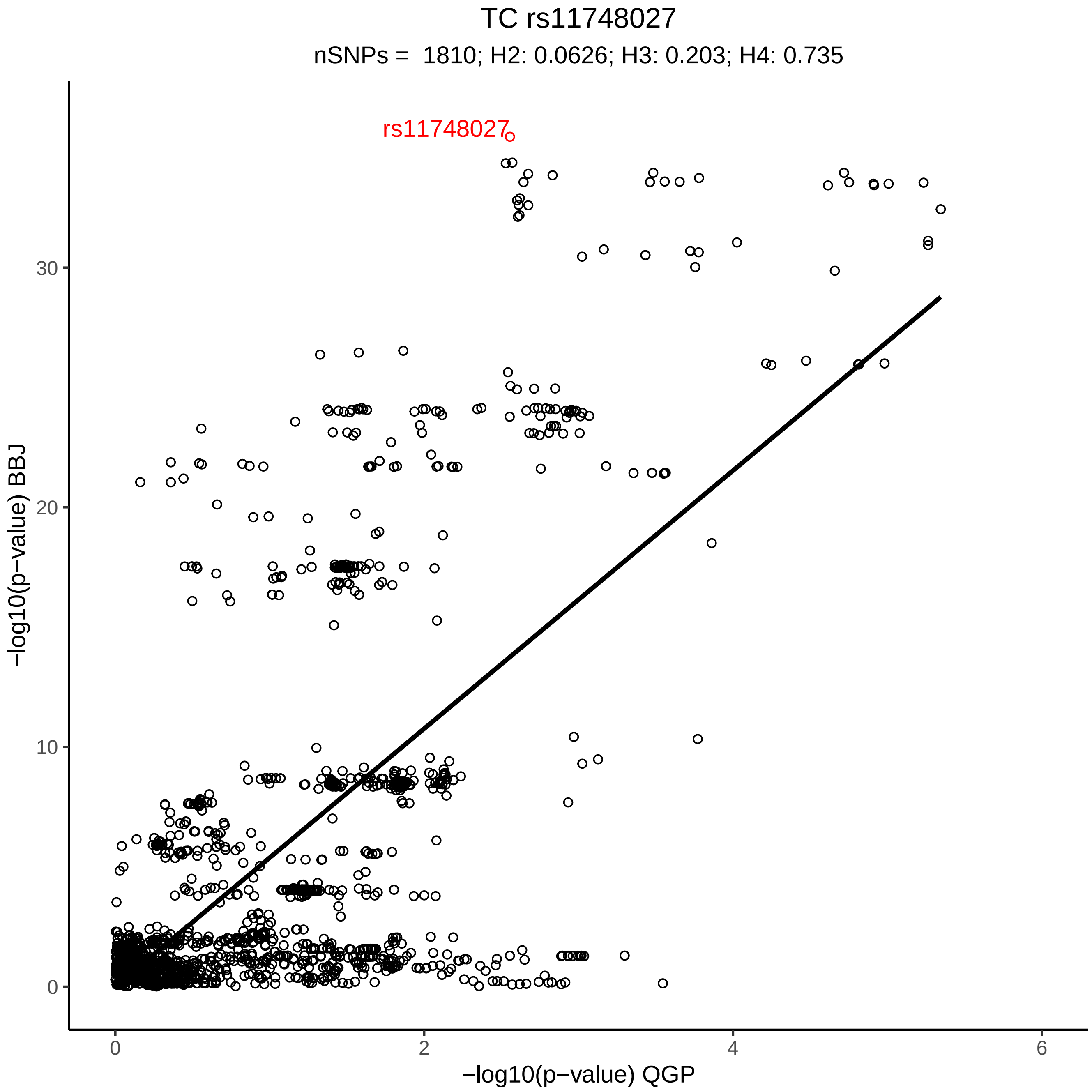

TC rs11748027
nSNPs = 1810; H2: 0.0626; H3: 0.203; H4: 0.735
rs11748027
−log10(p−value) BBJ
−log10(p−value) QGP
0
10
20
30
0
2
4
6

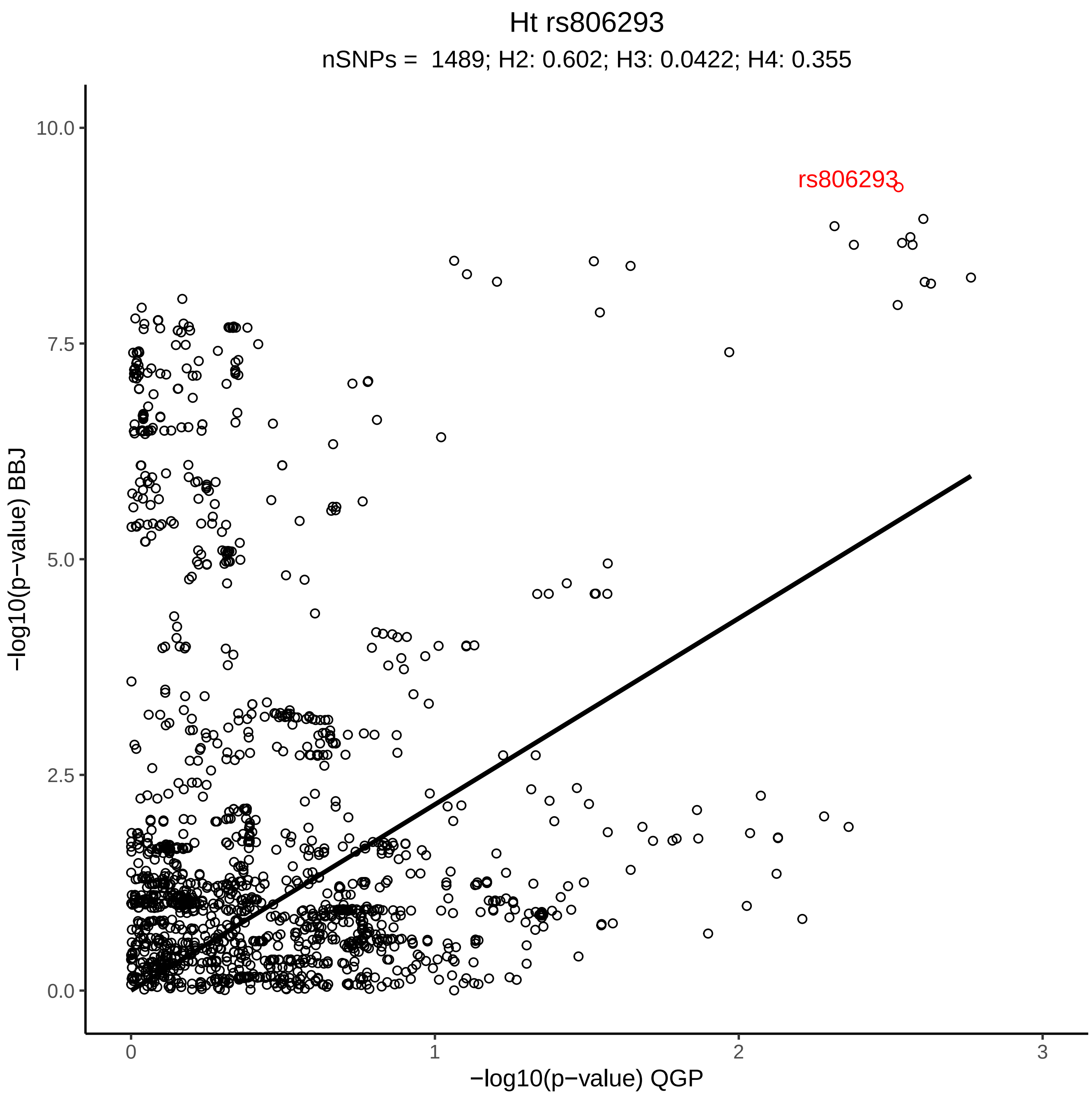

Ht rs806293
nSNPs = 1489; H2: 0.602; H3: 0.0422; H4: 0.355
rs806293
10.0
7.5
5.0
2.5
0.0
−log10(p−value) BBJ
0
1
2
3
−log10(p−value) QGP

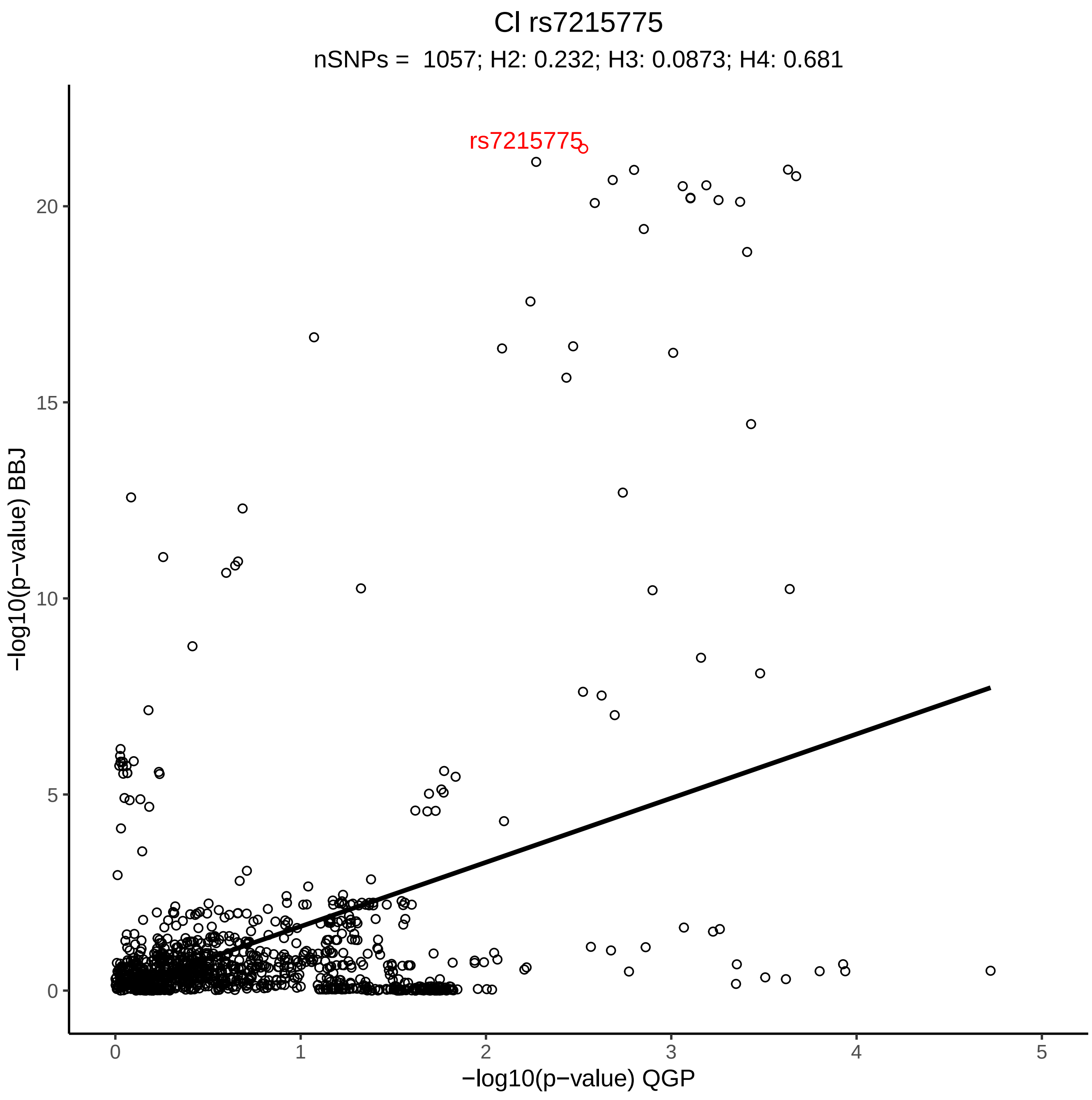
CI rs7215775
nSNPs = 1057; H2: 0.232; H3: 0.0873; H4: 0.681
rs7215775
−log10(p−value) BBJ
0
5
10
15
20
0
1
2
3
4
5
−log10(p−value) QGP

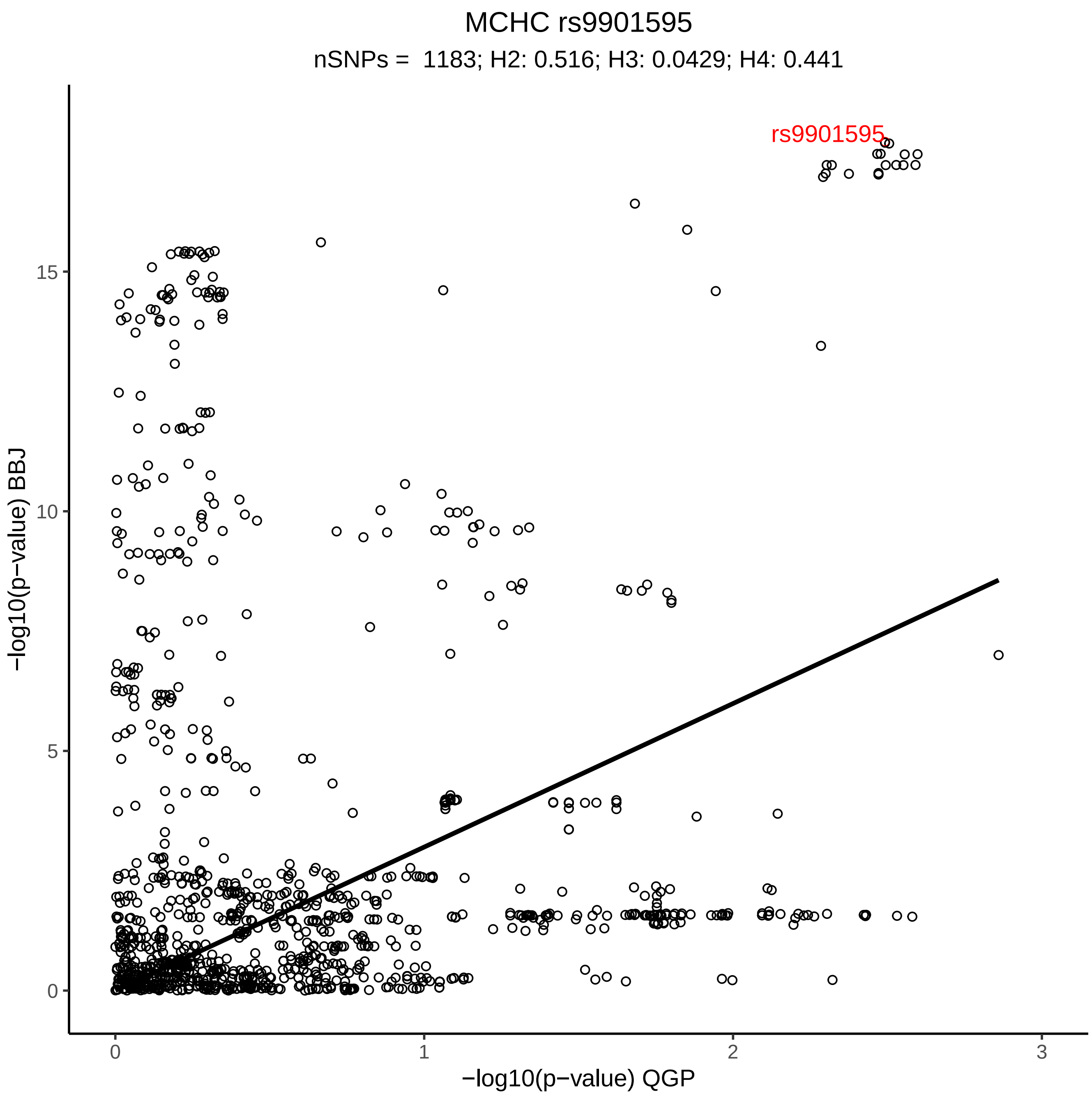
MCHC rs9901595
nSNPs = 1183; H2: 0.516; H3: 0.0429; H4: 0.441
rs9901595
−log10(p−value) BBJ
−log10(p−value) QGP
0
5
10
15
0
1
2
3

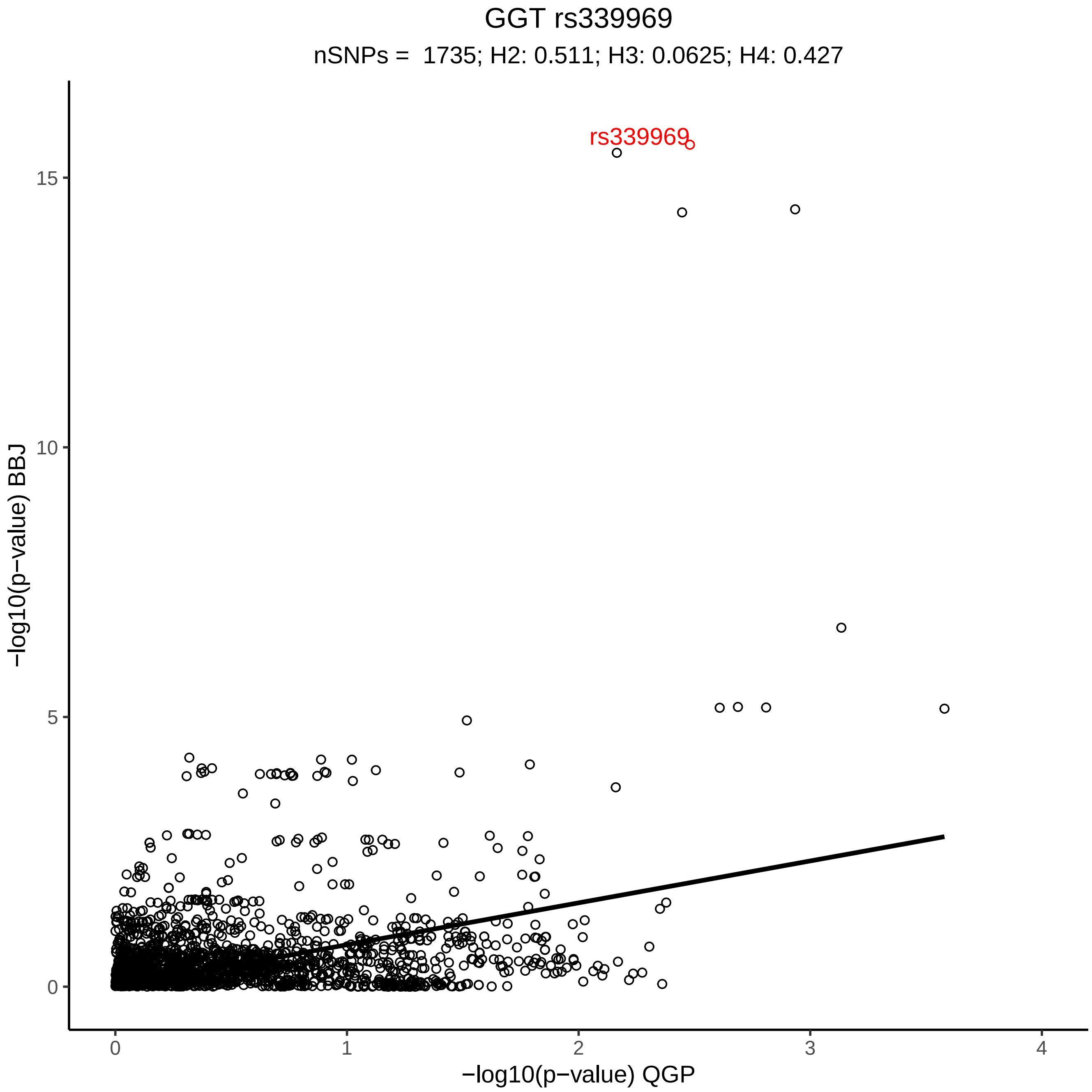
GGT rs339969
nSNPs = 1735; H2: 0.511; H3: 0.0625; H4: 0.427
rs339969
−log10(p−value) BBJ
−log10(p−value) QGP
0
5
10
15
0
1
2
3
4

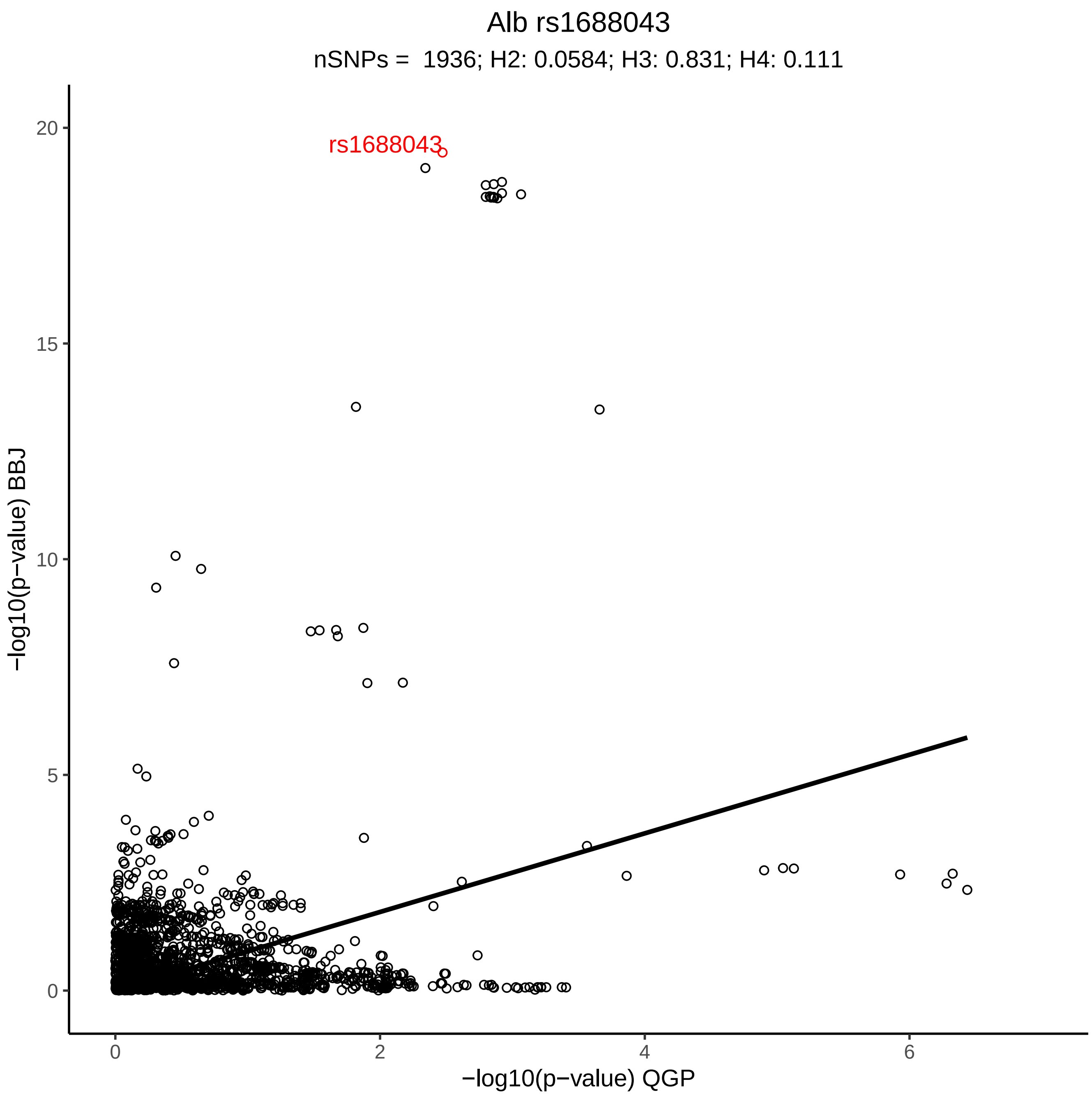

Alb rs1688043
nSNPs = 1936; H2: 0.0584; H3: 0.831; H4: 0.111
rs1688043
−log10(p−value) BBJ
−log10(p−value) QGP
0
5
10
15
20
0
2
4
6

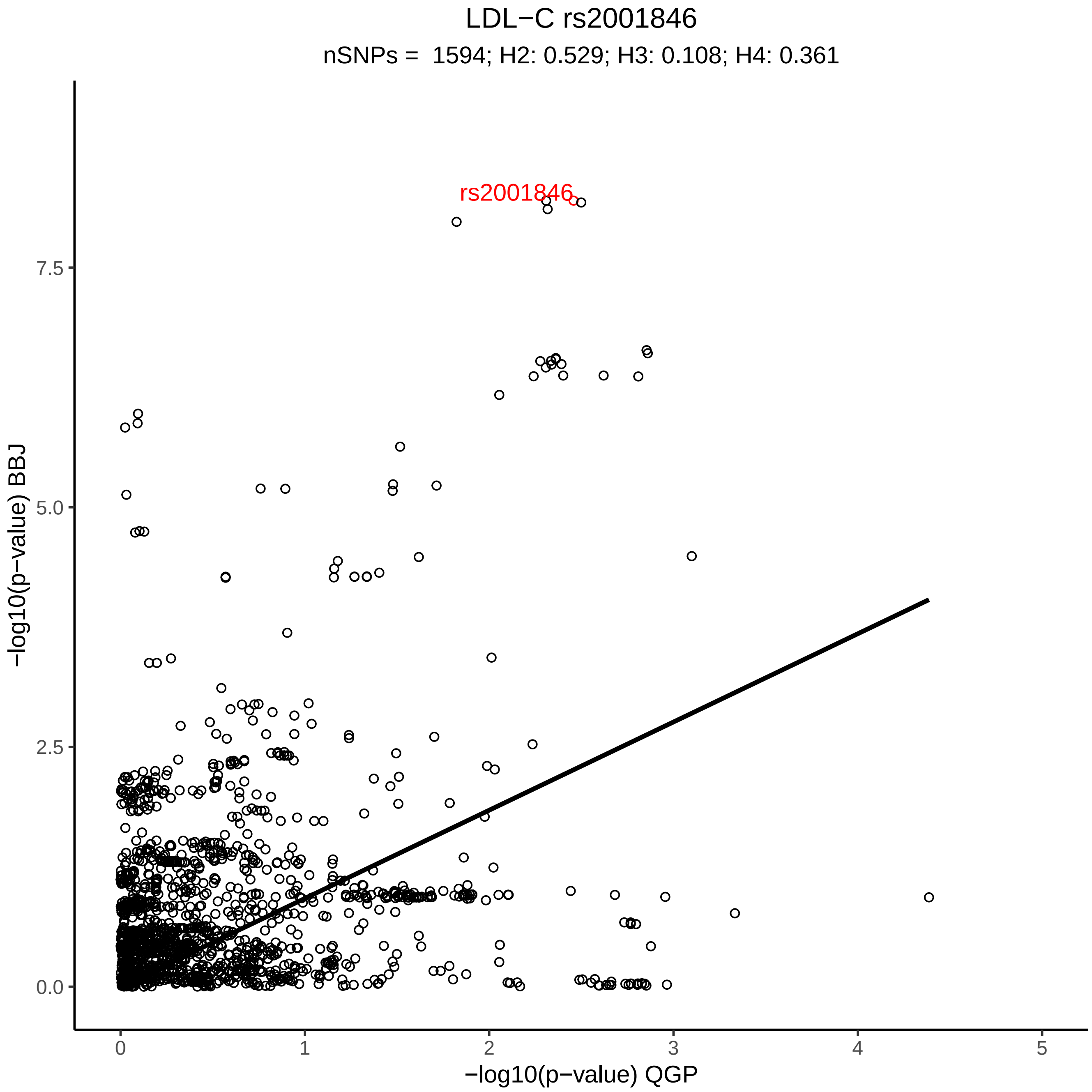

LDL−C rs2001846
nSNPs = 1594; H2: 0.529; H3: 0.108; H4: 0.361
rs2001846
−log10(p−value) BBJ
7.5
5.0
2.5
0.0
0
1
2
3
4
5
−log10(p−value) QGP

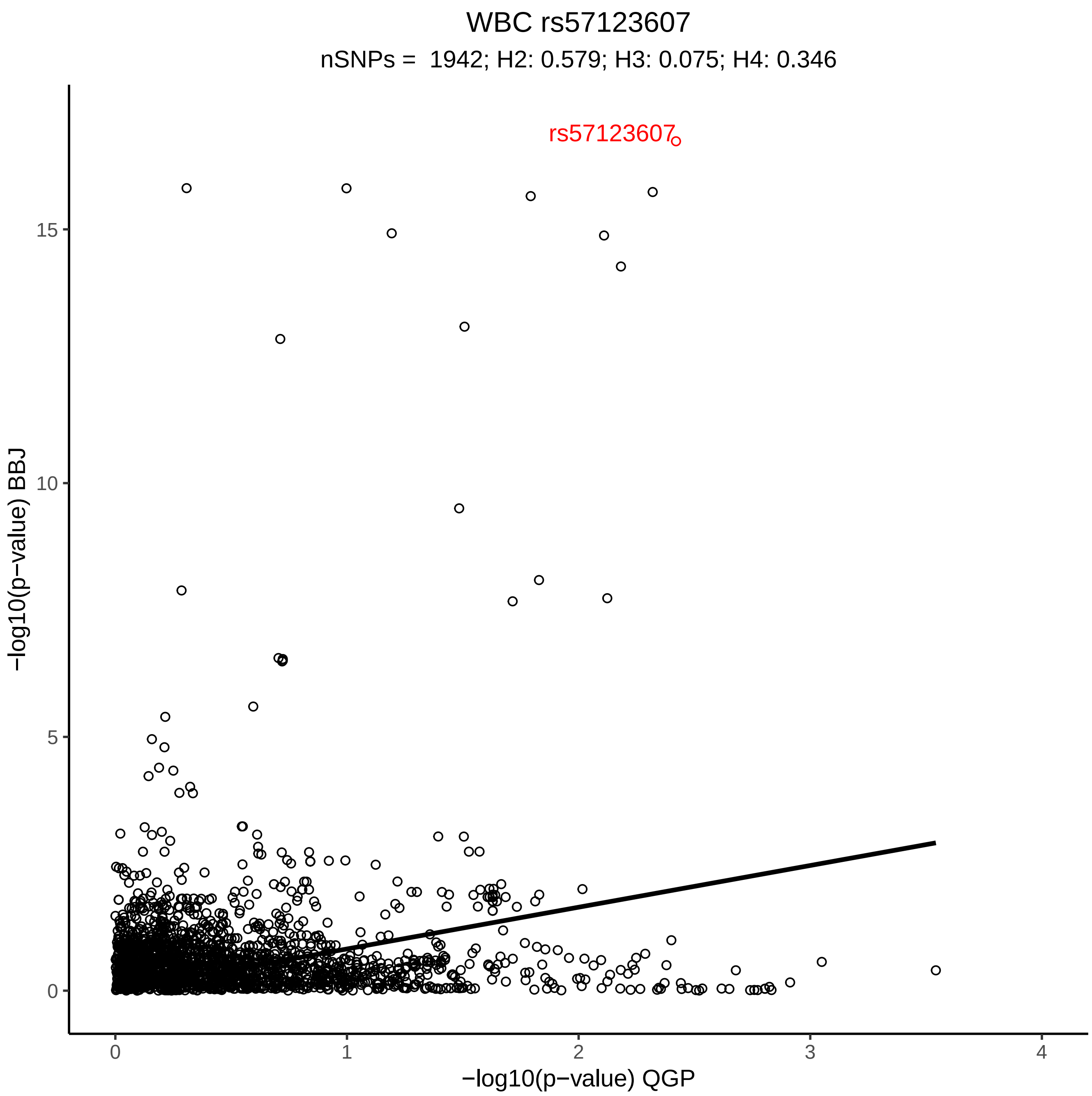

WBC rs57123607
nSNPs = 1942; H2: 0.579; H3: 0.075; H4: 0.346
rs57123607
−log10(p−value) BBJ
−log10(p−value) QGP
0
5
10
15
0
1
2
3
4

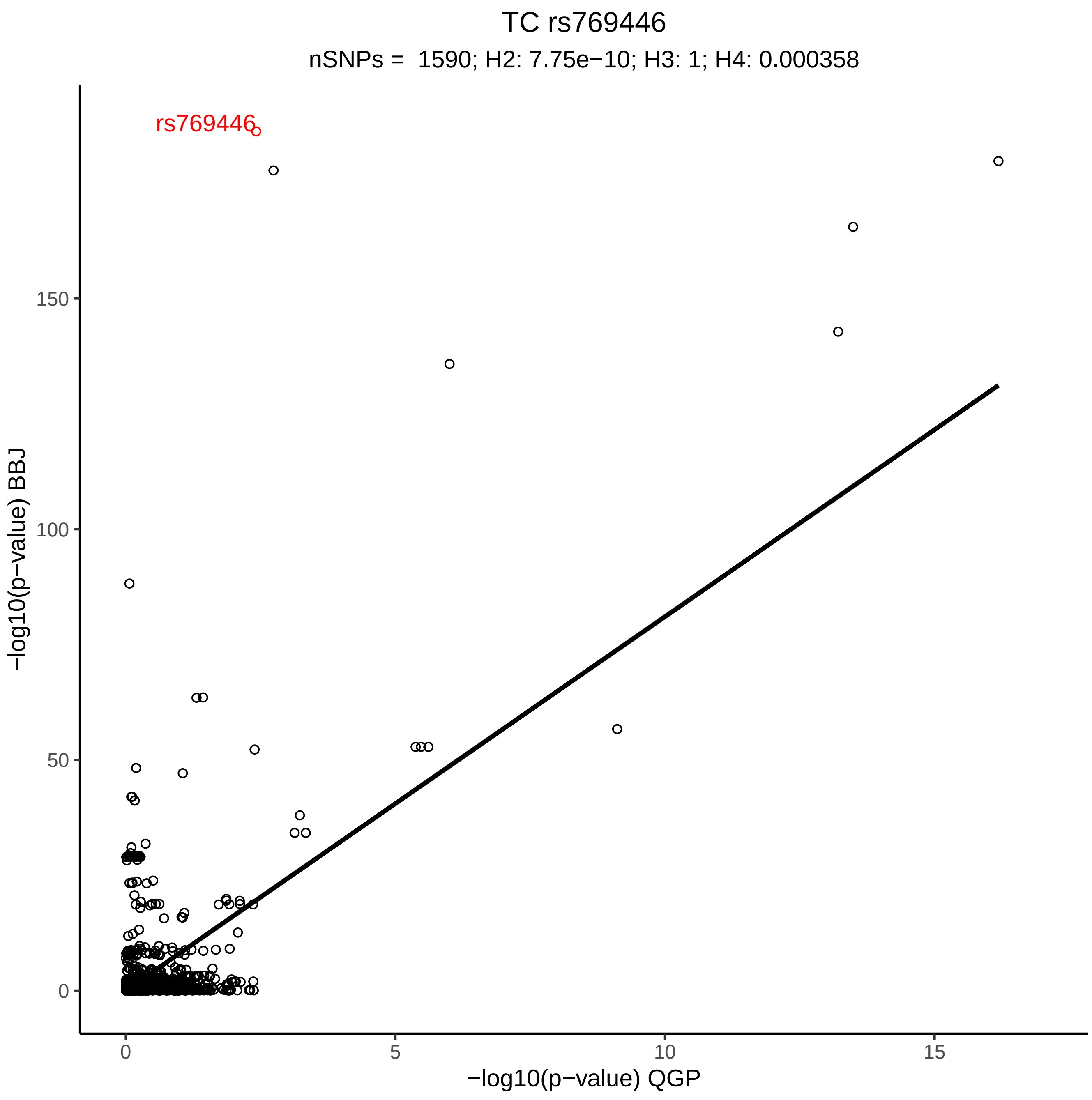
TC rs769446
nSNPs = 1590; H2: 7.75e−10; H3: 1; H4: 0.000358
rs769446
−log10(p−value) BBJ
0
50
100
150
−log10(p−value) QGP
0
5
10
15

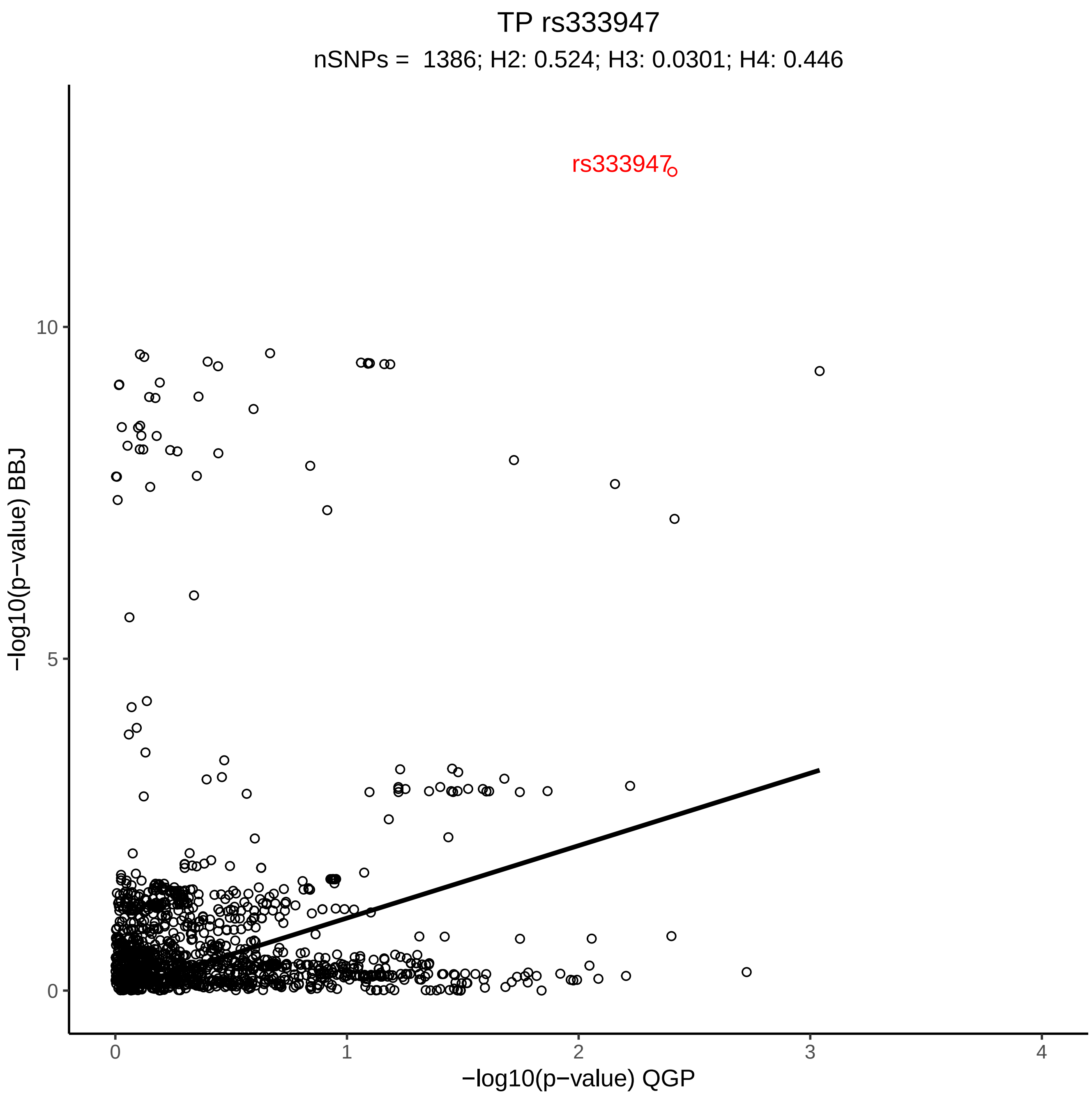

TP rs333947
nSNPs = 1386; H2: 0.524; H3: 0.0301; H4: 0.446
rs333947
−log10(p−value) BBJ
10
5
0
0
1
2
3
4
−log10(p−value) QGP

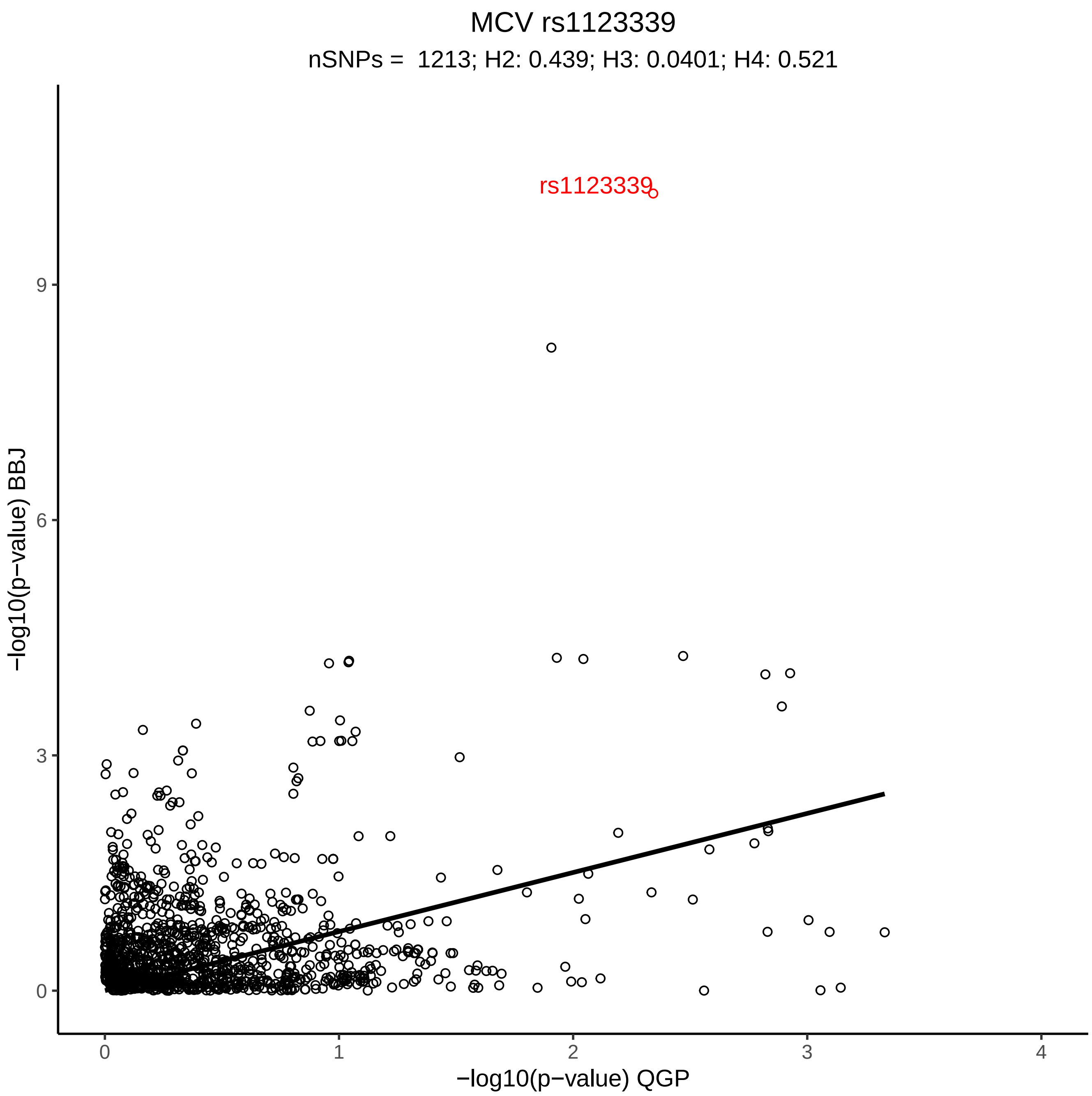
MCV rs1123339
nSNPs = 1213; H2: 0.439; H3: 0.0401; H4: 0.521
rs1123339
−log10(p−value) BBJ
−log10(p−value) QGP
0
3
6
9
0
1
2
3
4

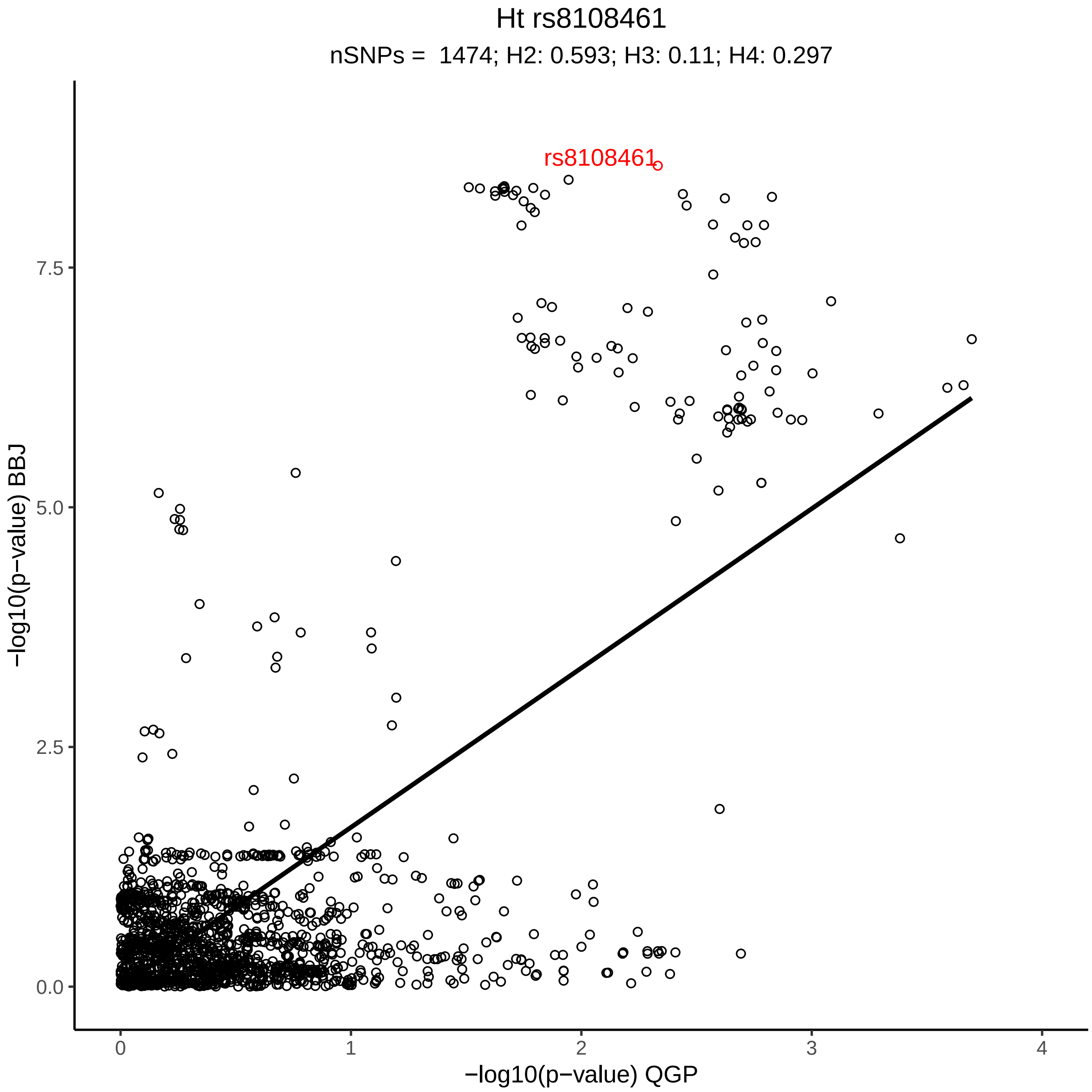
Ht rs8108461
nSNPs = 1474; H2: 0.593; H3: 0.11; H4: 0.297
rs8108461
−log10(p−value) BBJ
0.0
2.5
5.0
7.5
0
1
2
3
4
−log10(p−value) QGP

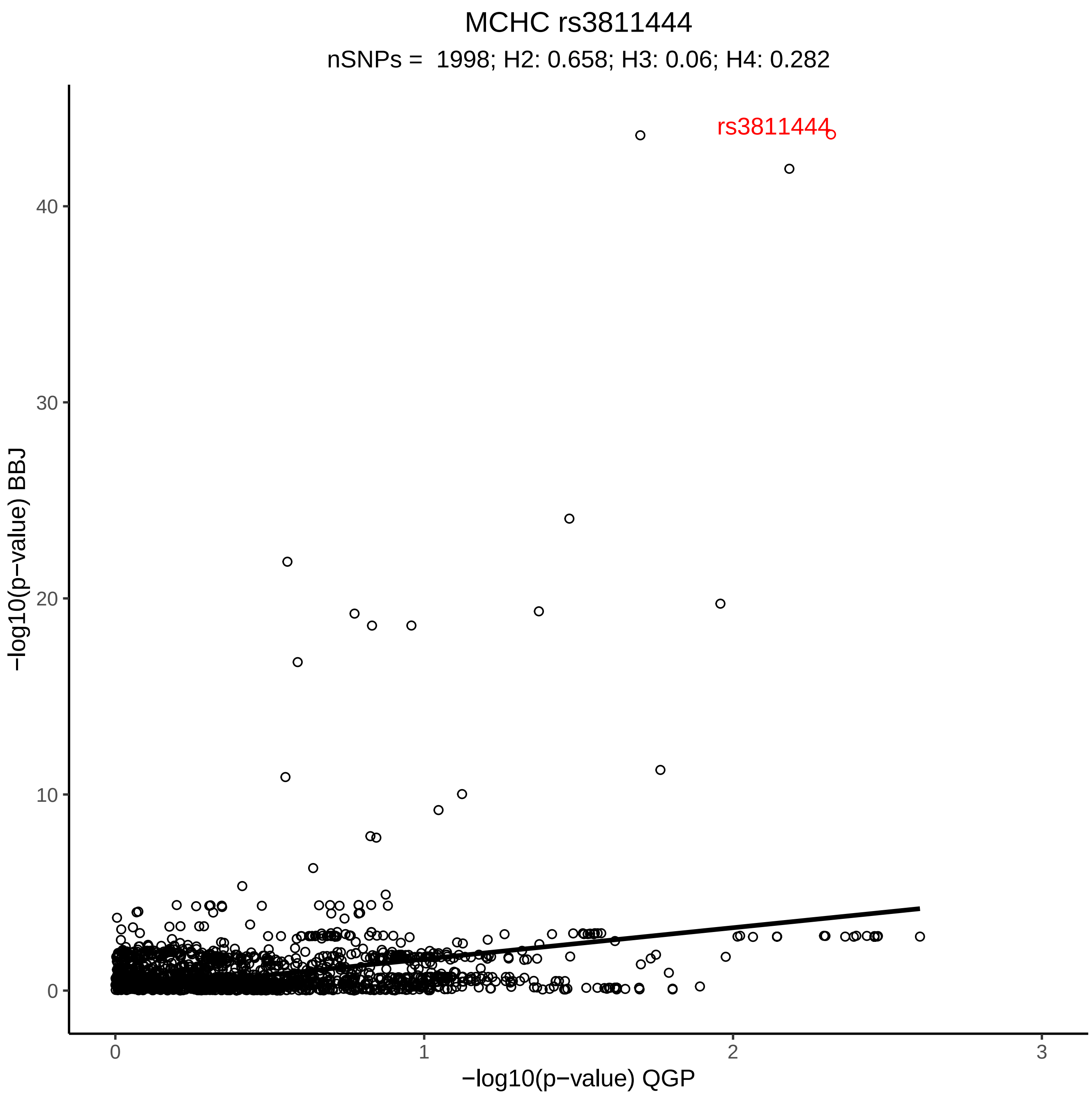
MCHC rs3811444
nSNPs = 1998; H2: 0.658; H3: 0.06; H4: 0.282
rs3811444
−log10(p−value) BBJ
−log10(p−value) QGP
0
10
20
30
40
0
1
2
3

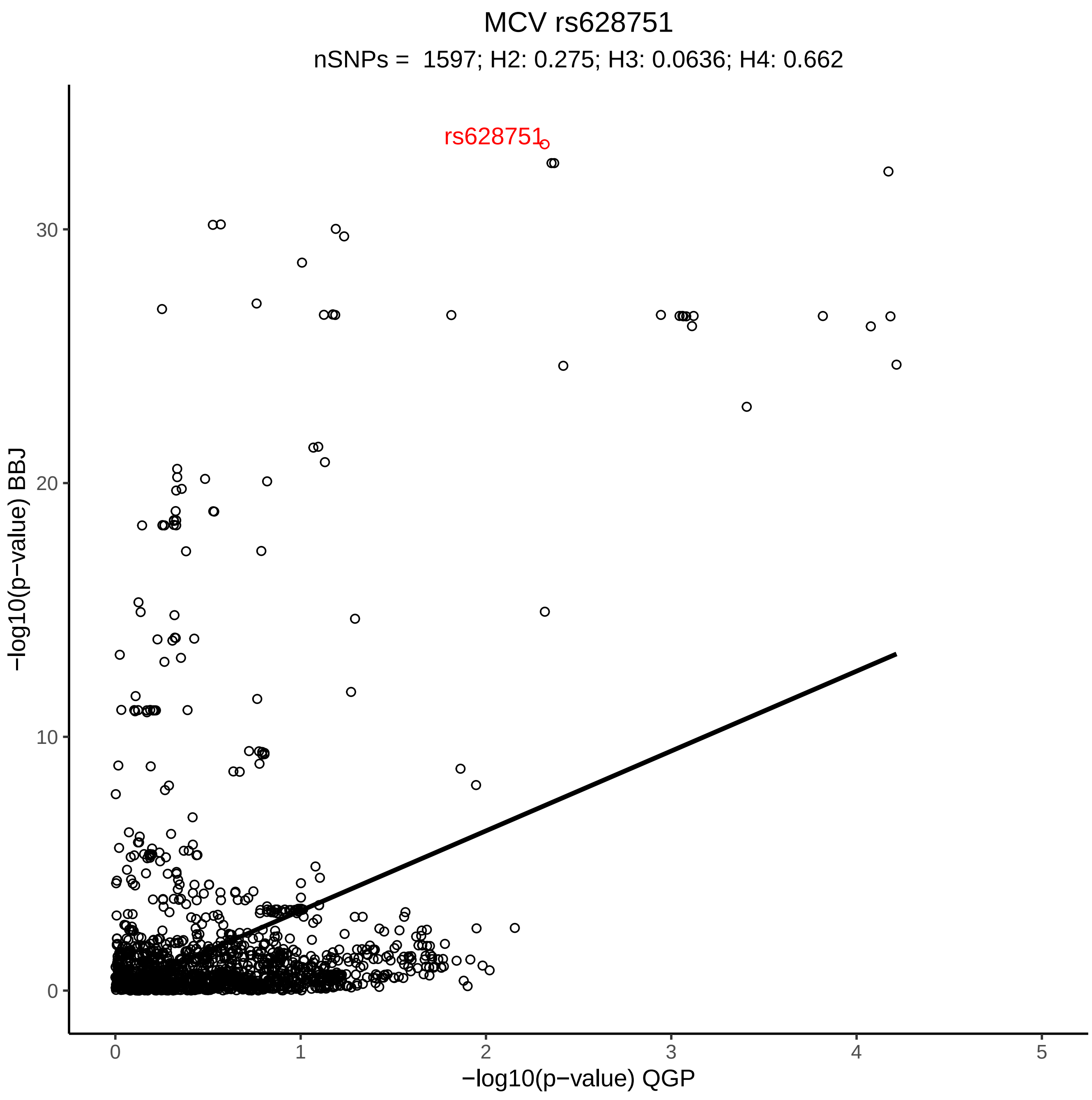
MCV rs628751
nSNPs = 1597; H2: 0.275; H3: 0.0636; H4: 0.662
rs628751
30
20
10
0
−log10(p−value) BBJ
0
1
2
3
4
5
−log10(p−value) QGP

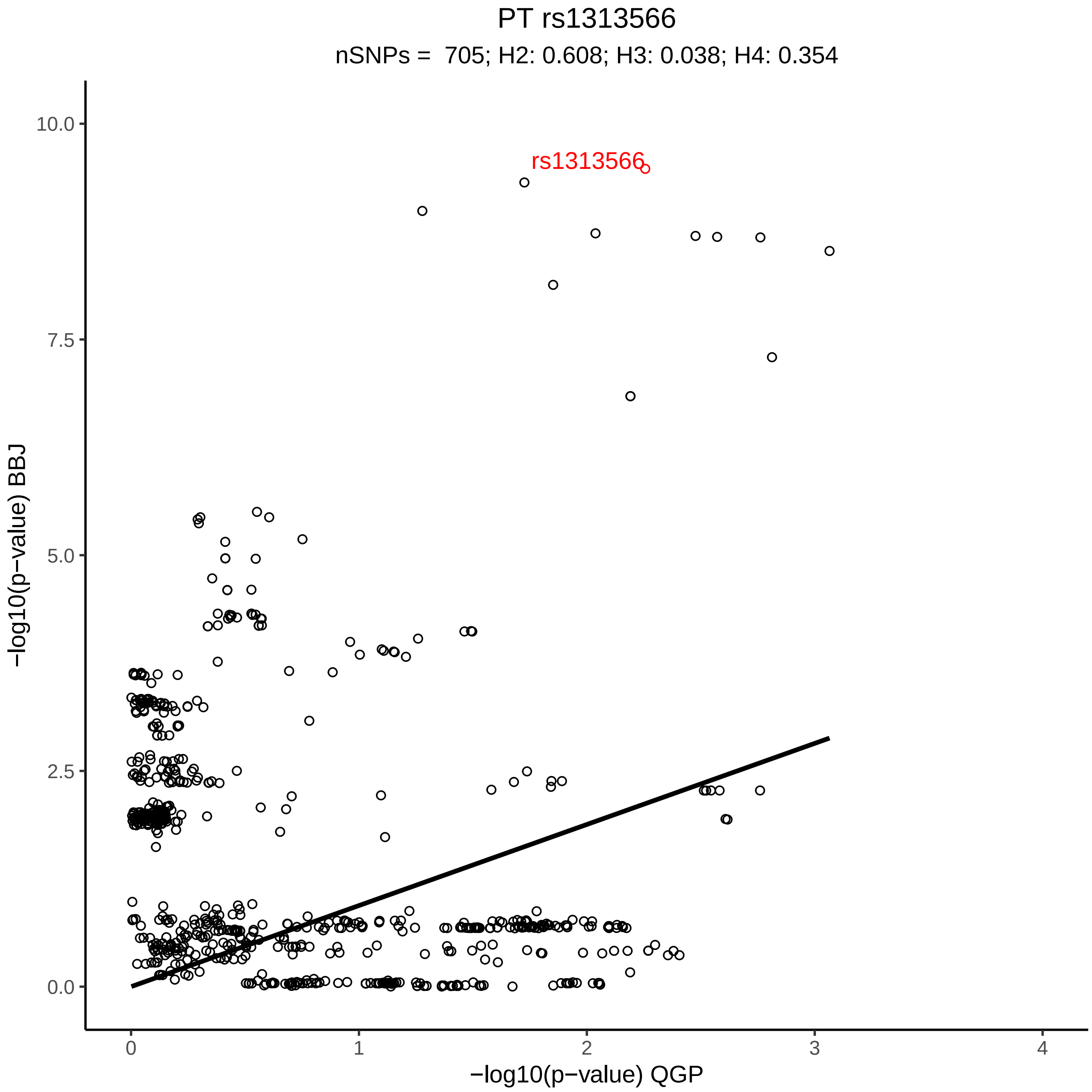
PT rs1313566
nSNPs = 705; H2: 0.608; H3: 0.038; H4: 0.354
rs1313566
10.0
7.5
5.0
2.5
0.0
−log10(p−value) BBJ
0
1
2
3
4
−log10(p−value) QGP

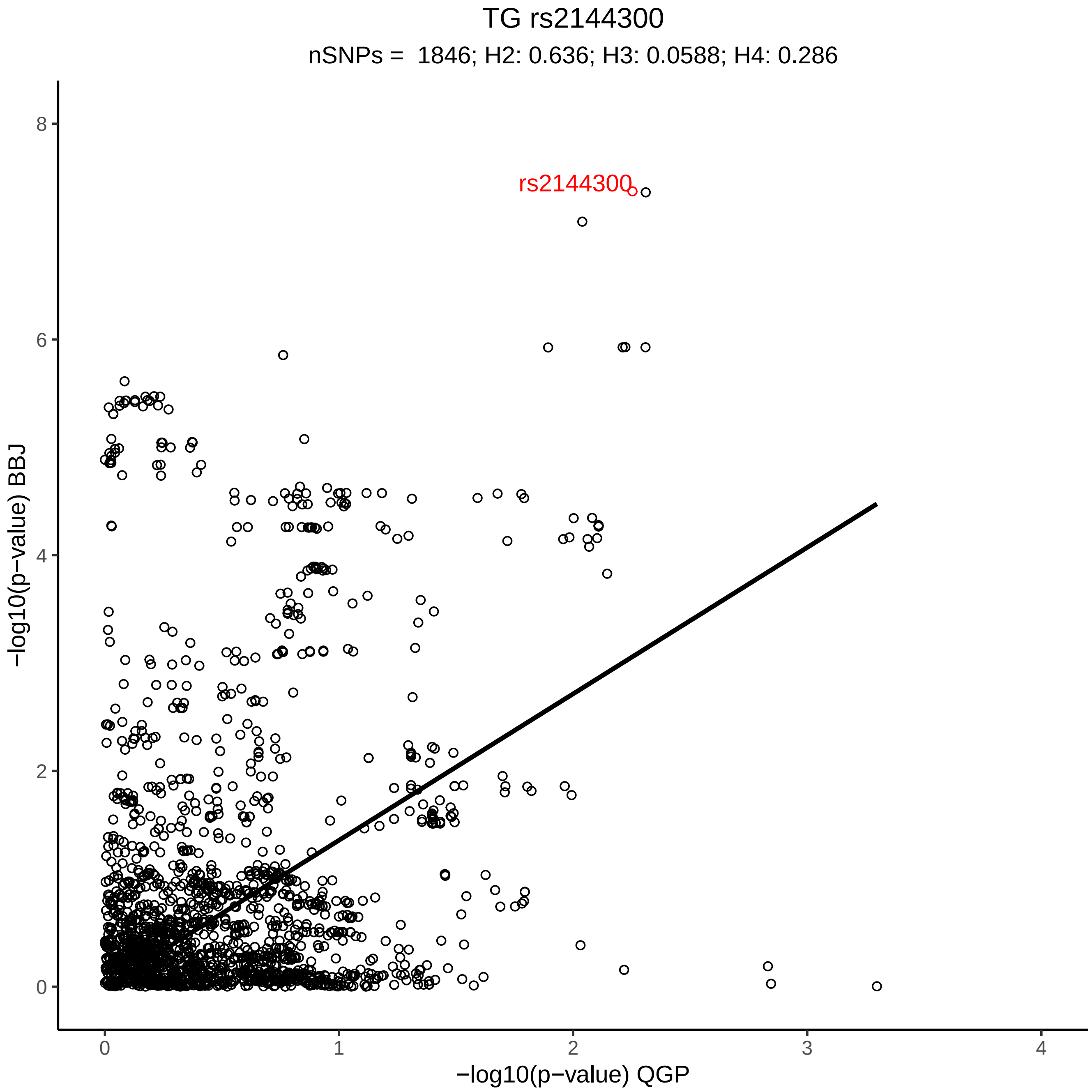

TG rs2144300
nSNPs = 1846; H2: 0.636; H3: 0.0588; H4: 0.286
rs2144300
−log10(p−value) BBJ
−log10(p−value) QGP
0
2
4
6
8
0
1
2
3
4

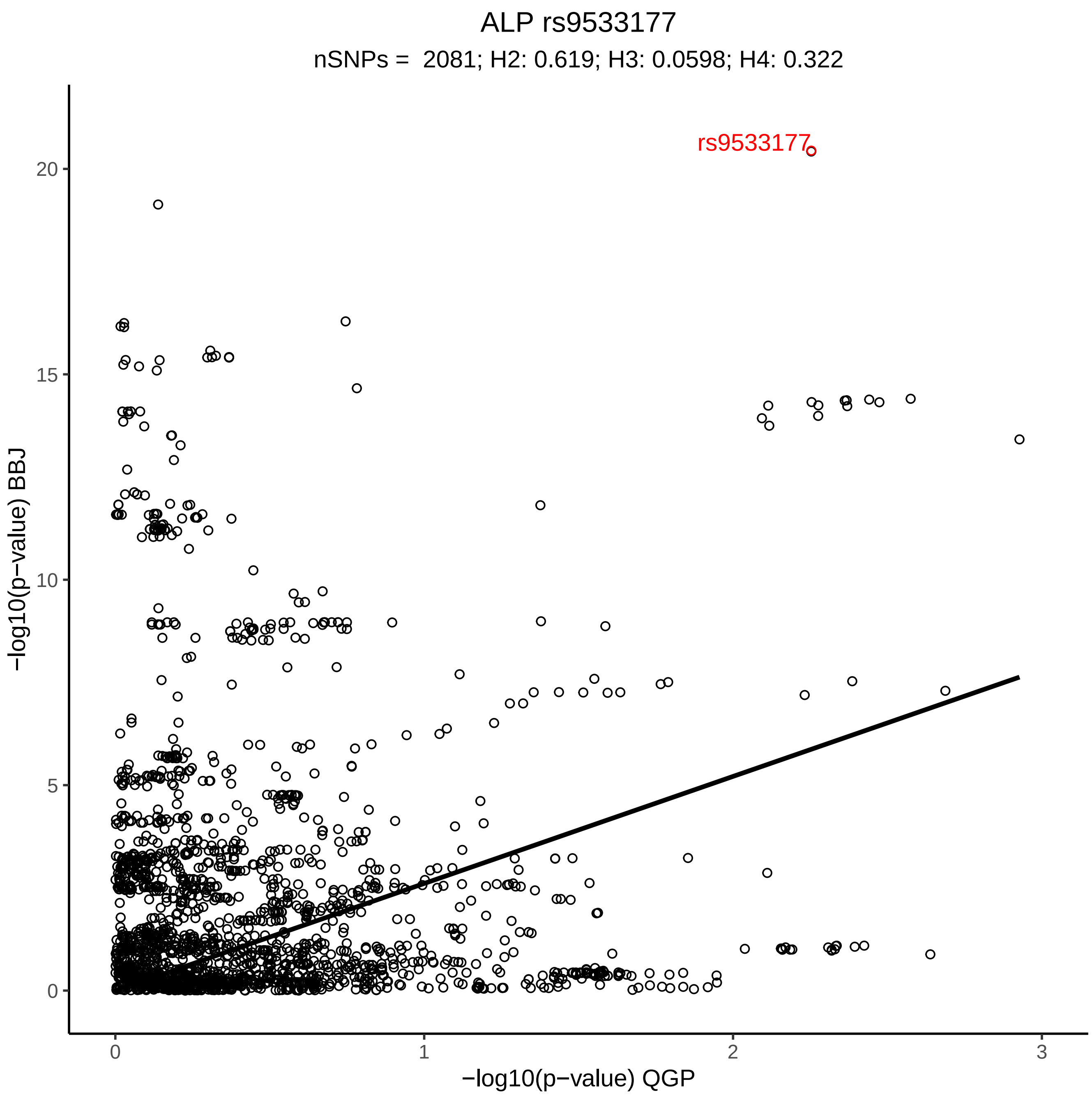

ALP rs9533177
nSNPs = 2081; H2: 0.619; H3: 0.0598; H4: 0.322
rs9533177
−log10(p−value) BBJ
0
5
10
15
20
−log10(p−value) QGP
0
1
2
3

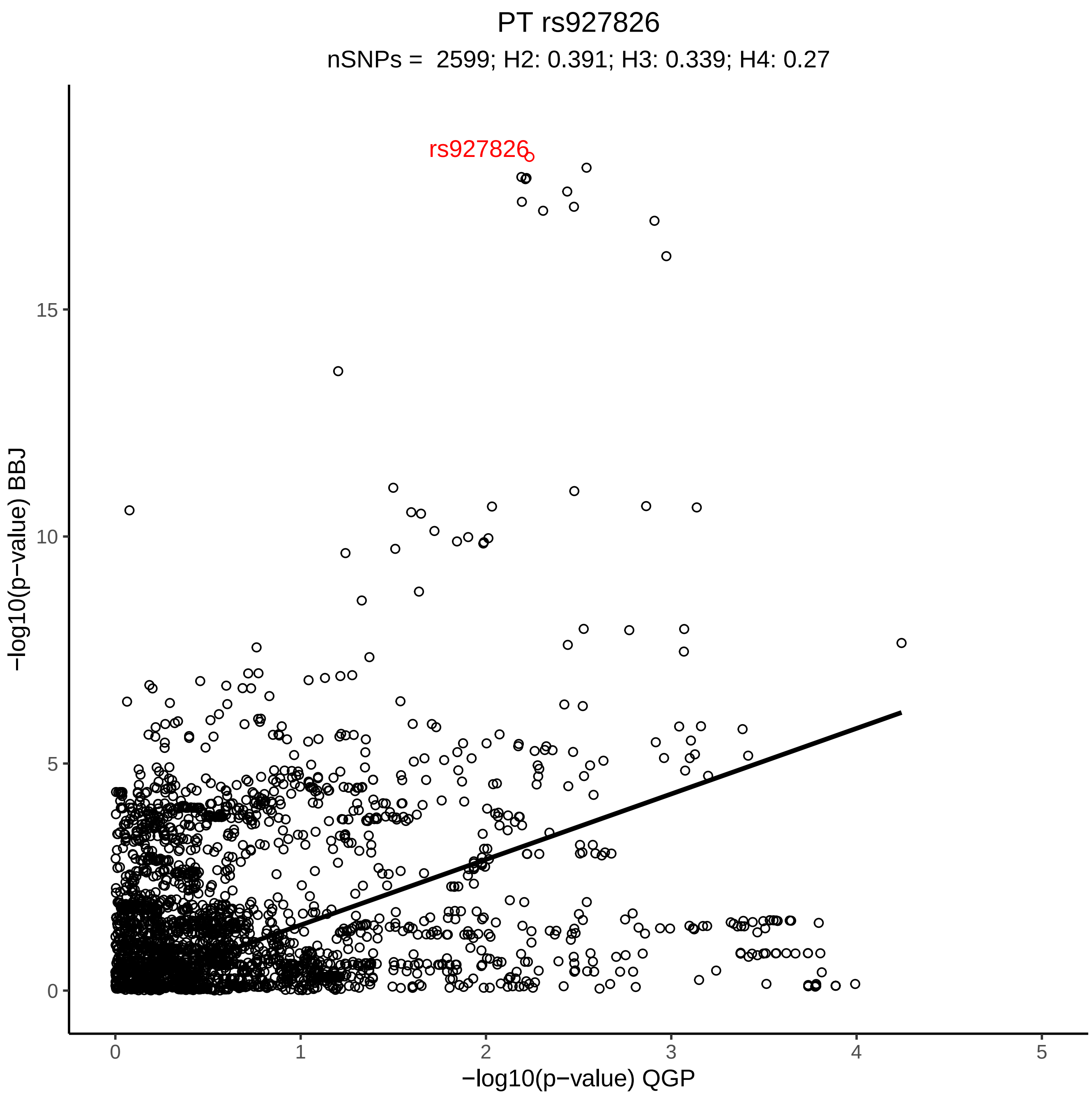

PT rs927826
nSNPs = 2599; H2: 0.391; H3: 0.339; H4: 0.27
rs927826
−log10(p−value) BBJ
0
5
10
15
0
1
2
3
4
5
−log10(p−value) QGP

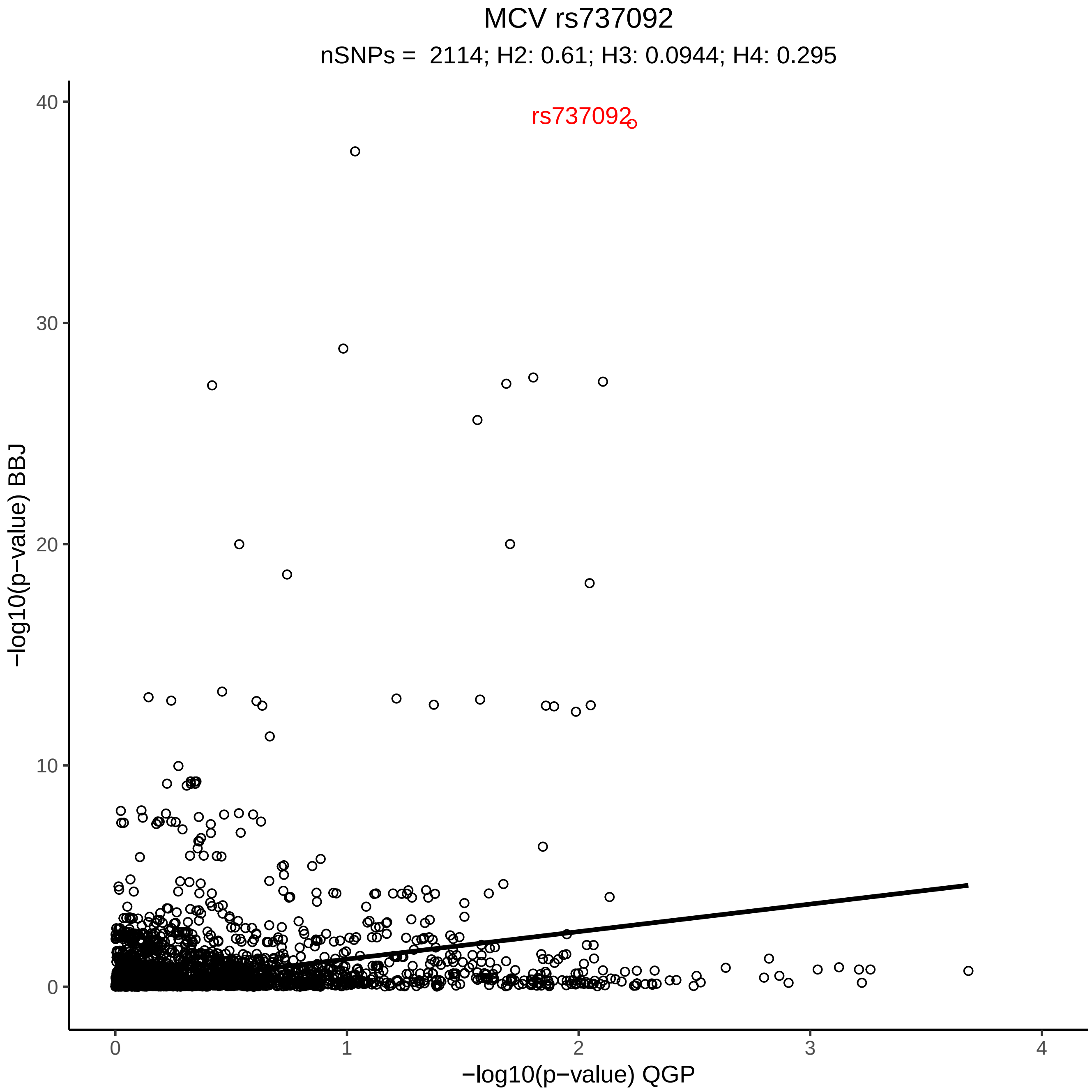
MCV rs737092
nSNPs = 2114; H2: 0.61; H3: 0.0944; H4: 0.295
rs737092
40
30
20
10
0
−log10(p−value) BBJ
0
1
2
3
4
−log10(p−value) QGP

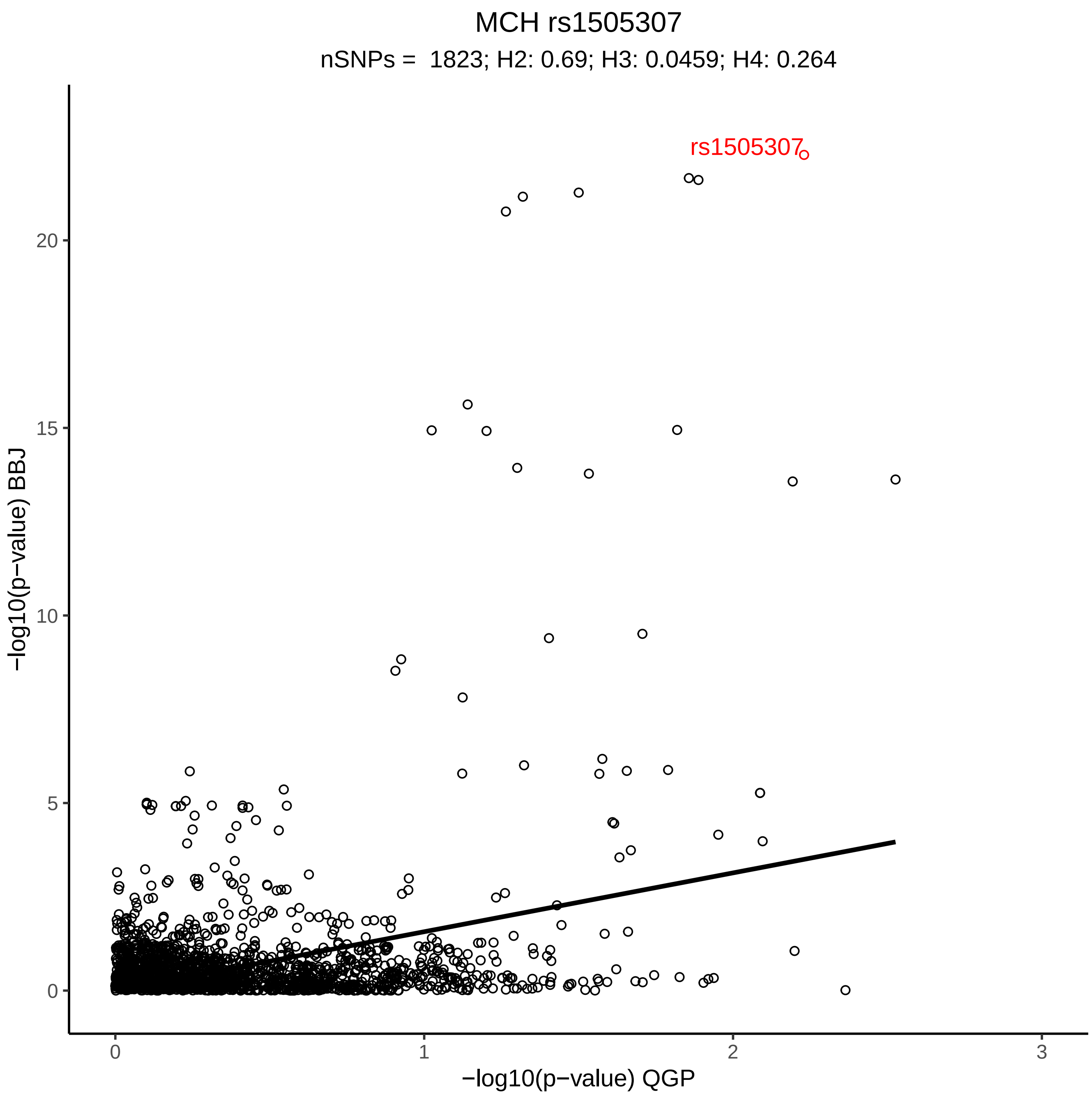

MCH rs1505307
nSNPs = 1823; H2: 0.69; H3: 0.0459; H4: 0.264
rs1505307
−log10(p−value) BBJ
0
5
10
15
20
0
1
2
3
−log10(p−value) QGP

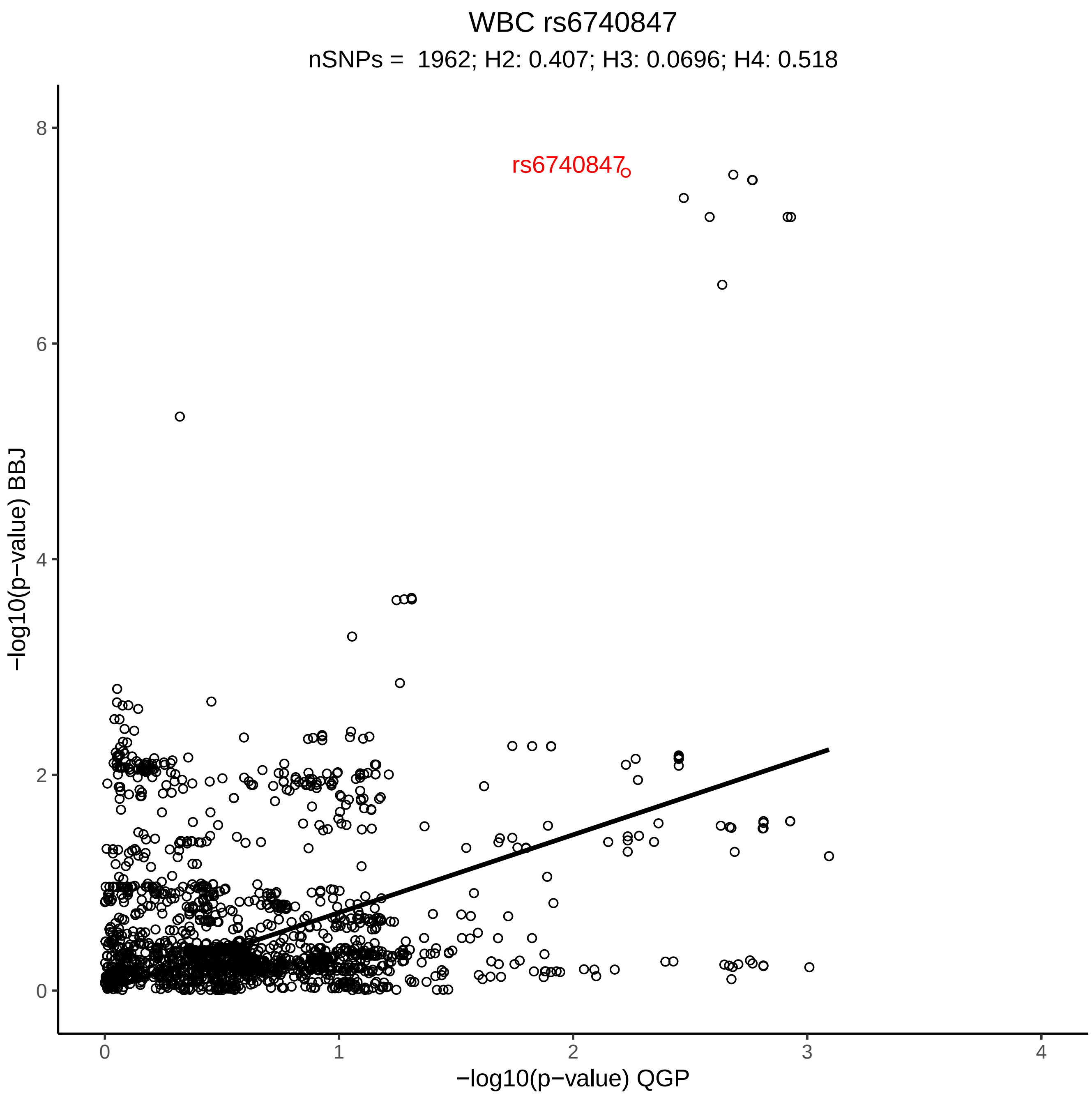
WBC rs6740847
nSNPs = 1962; H2: 0.407; H3: 0.0696; H4: 0.518
rs6740847
−log10(p−value) BBJ
−log10(p−value) QGP
0
2
4
6
8
0
1
2
3
4

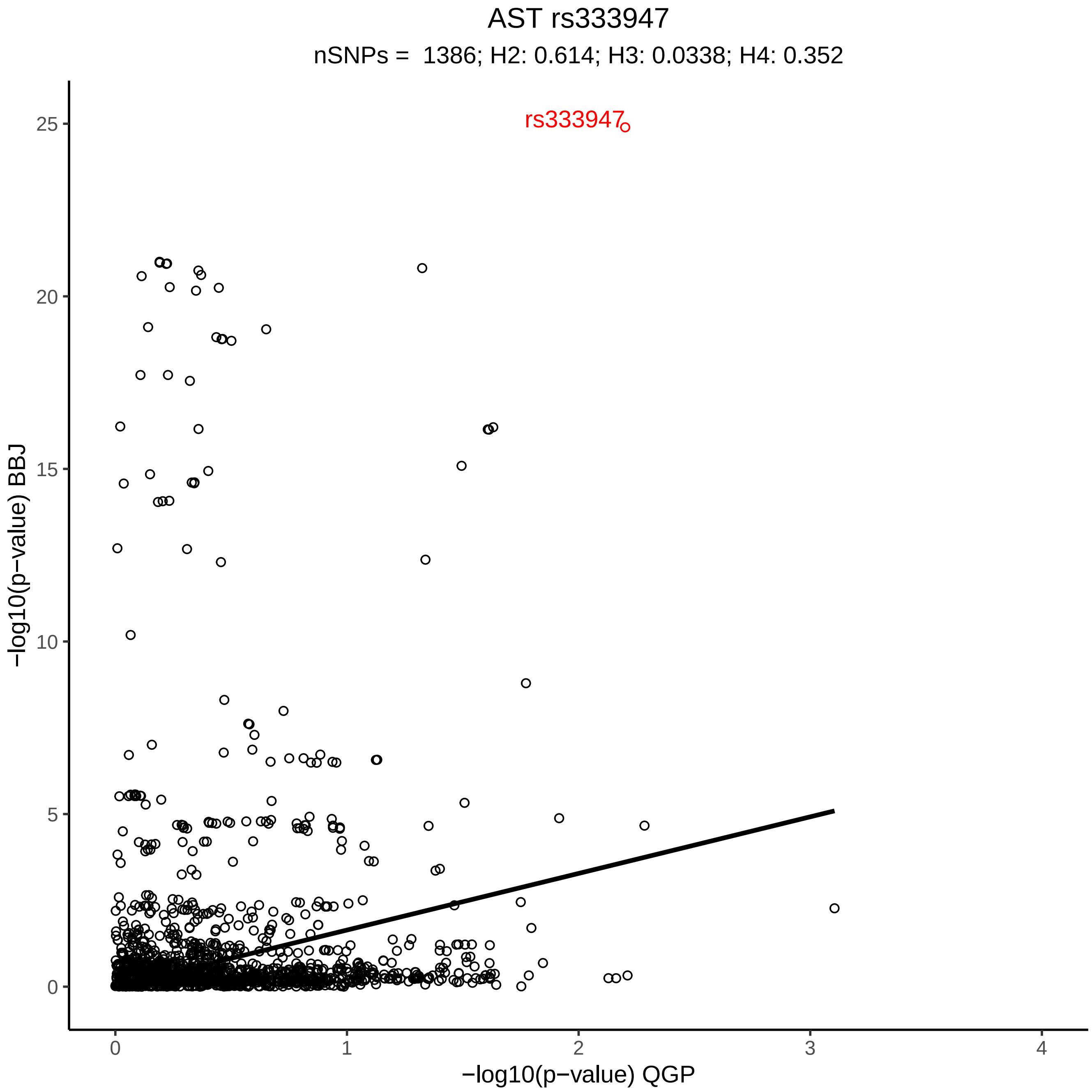
AST rs333947
nSNPs = 1386; H2: 0.614; H3: 0.0338; H4: 0.352
rs333947
−log10(p−value) BBJ
−log10(p−value) QGP
0
5
10
15
20
25
0
1
2
3
4

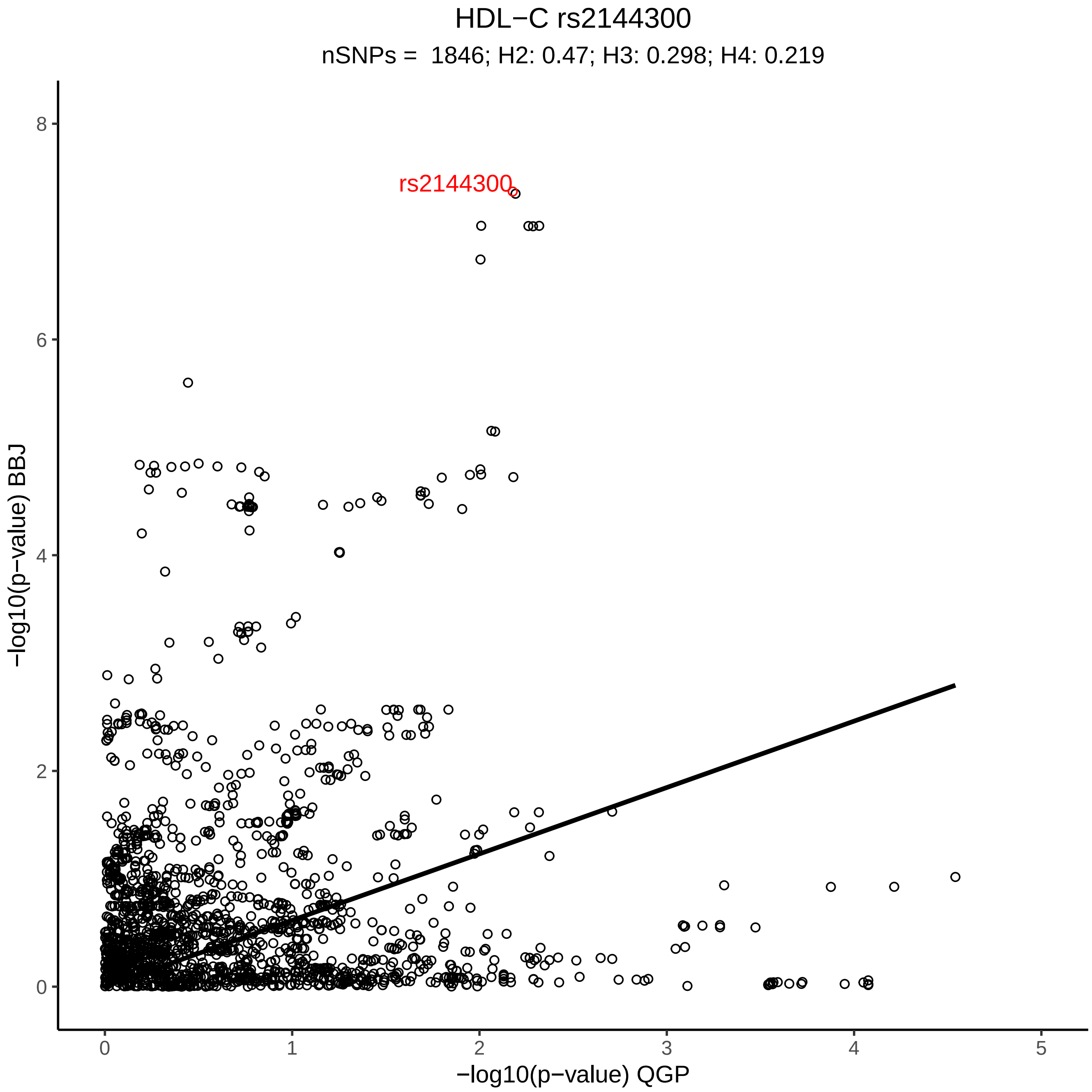

HDL−C rs2144300
nSNPs = 1846; H2: 0.47; H3: 0.298; H4: 0.219
rs2144300
−log10(p−value) BBJ
−log10(p−value) QGP
0
2
4
6
8
0
1
2
3
4
5

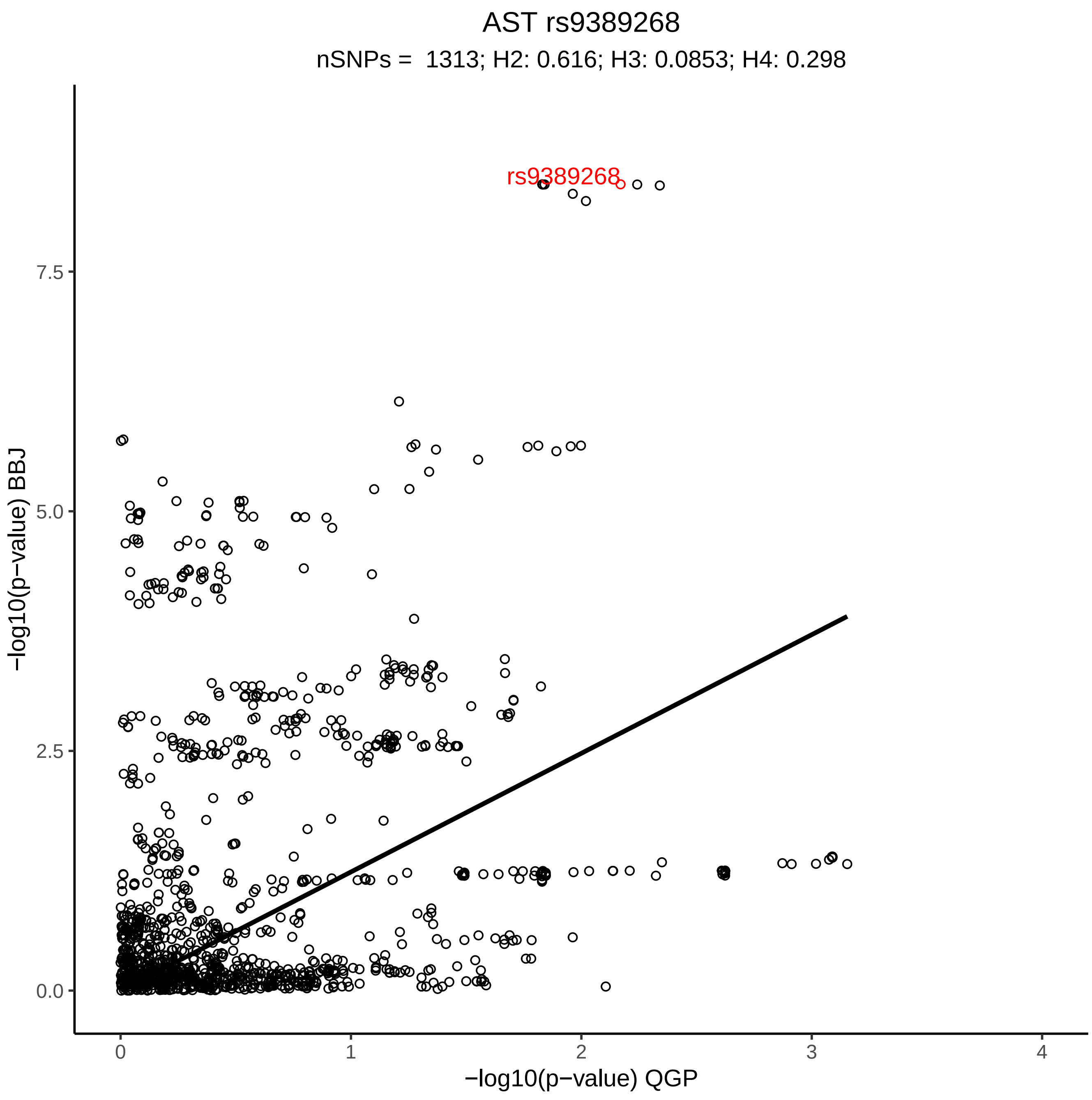
AST rs9389268
nSNPs = 1313; H2: 0.616; H3: 0.0853; H4: 0.298
rs9389268
−log10(p−value) BBJ
−log10(p−value) QGP
0.0
2.5
5.0
7.5
0
1
2
3
4

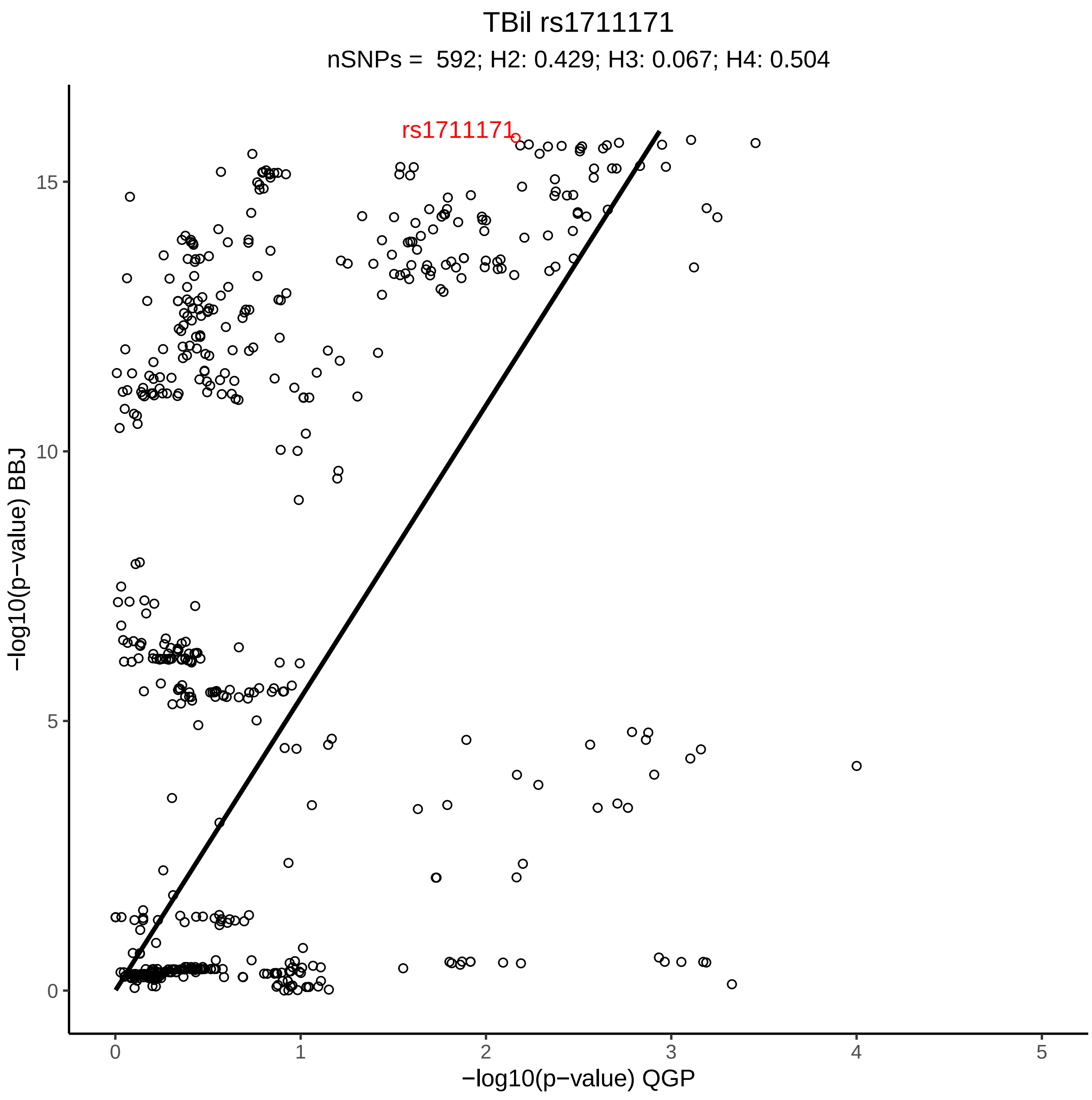

TBil rs1711171
nSNPs = 592; H2: 0.429; H3: 0.067; H4: 0.504
rs1711171
−log10(p−value) BBJ
−log10(p−value) QGP
0
5
10
15
0
1
2
3
4
5

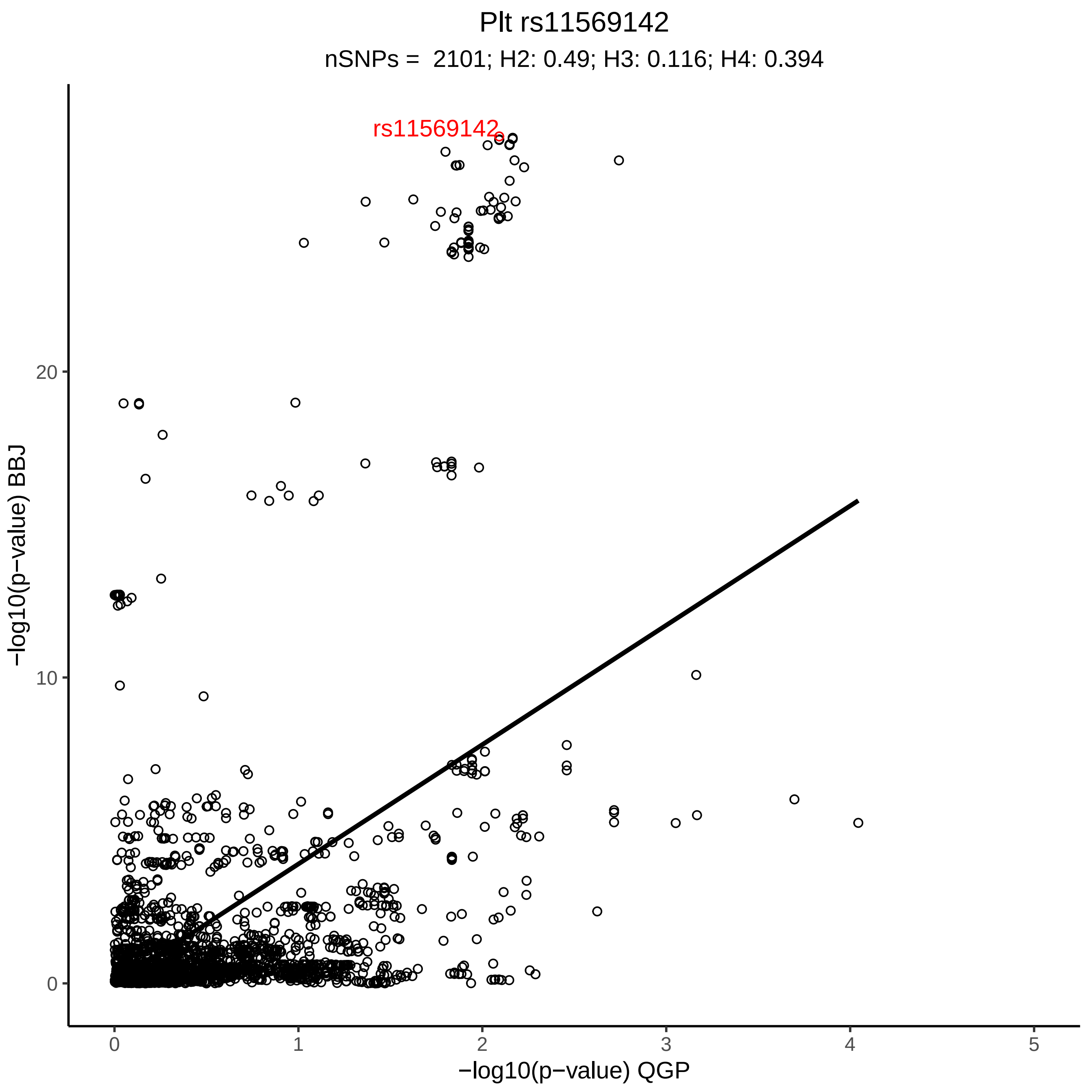
Plt rs11569142
nSNPs = 2101; H2: 0.49; H3: 0.116; H4: 0.394
rs11569142
−log10(p−value) BBJ
−log10(p−value) QGP
0
10
20
0
1
2
3
4
5

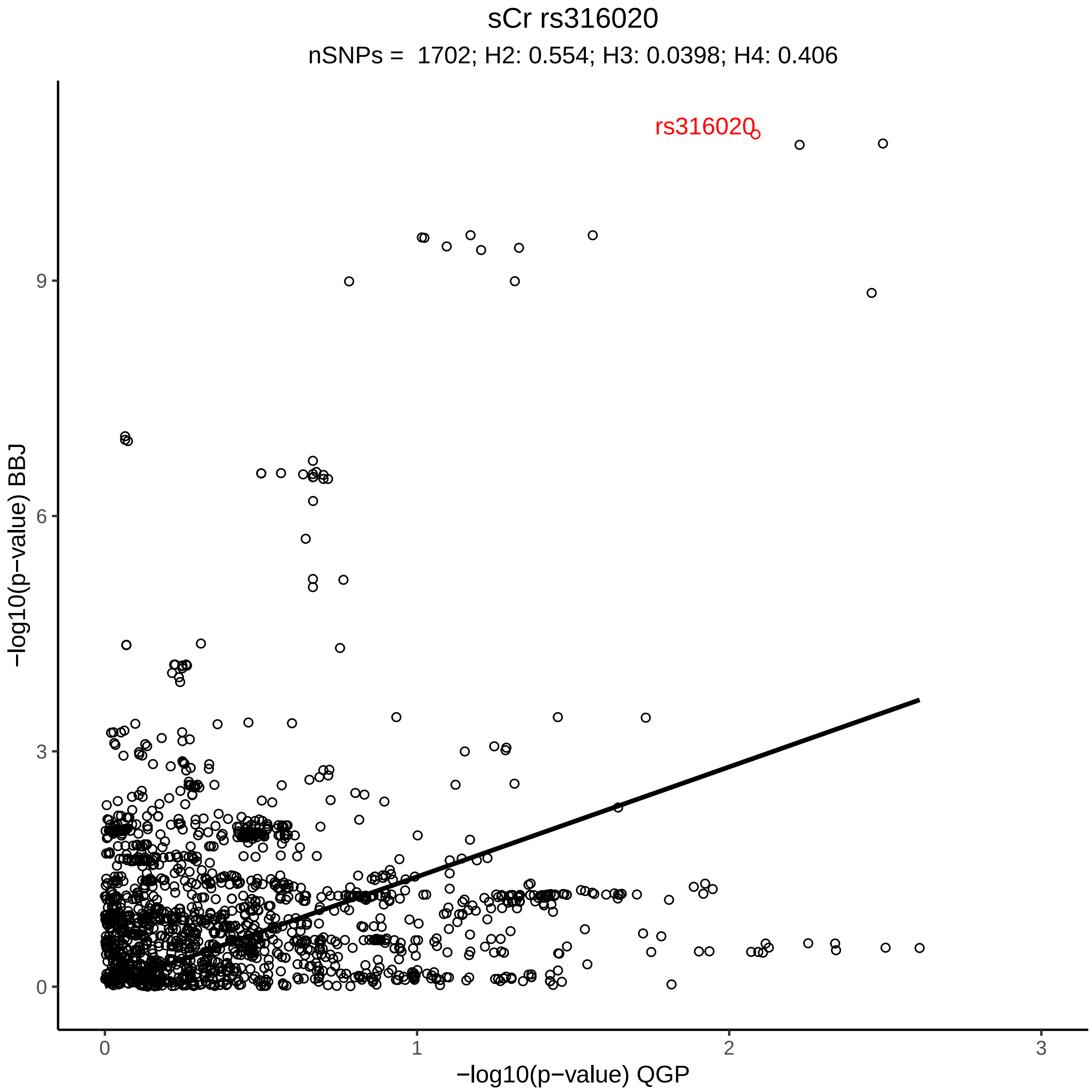
sCr rs316020
nSNPs = 1702; H2: 0.554; H3: 0.0398; H4: 0.406
rs316020
−log10(p−value) BBJ
−log10(p−value) QGP
0
3
6
9
0
1
2
3

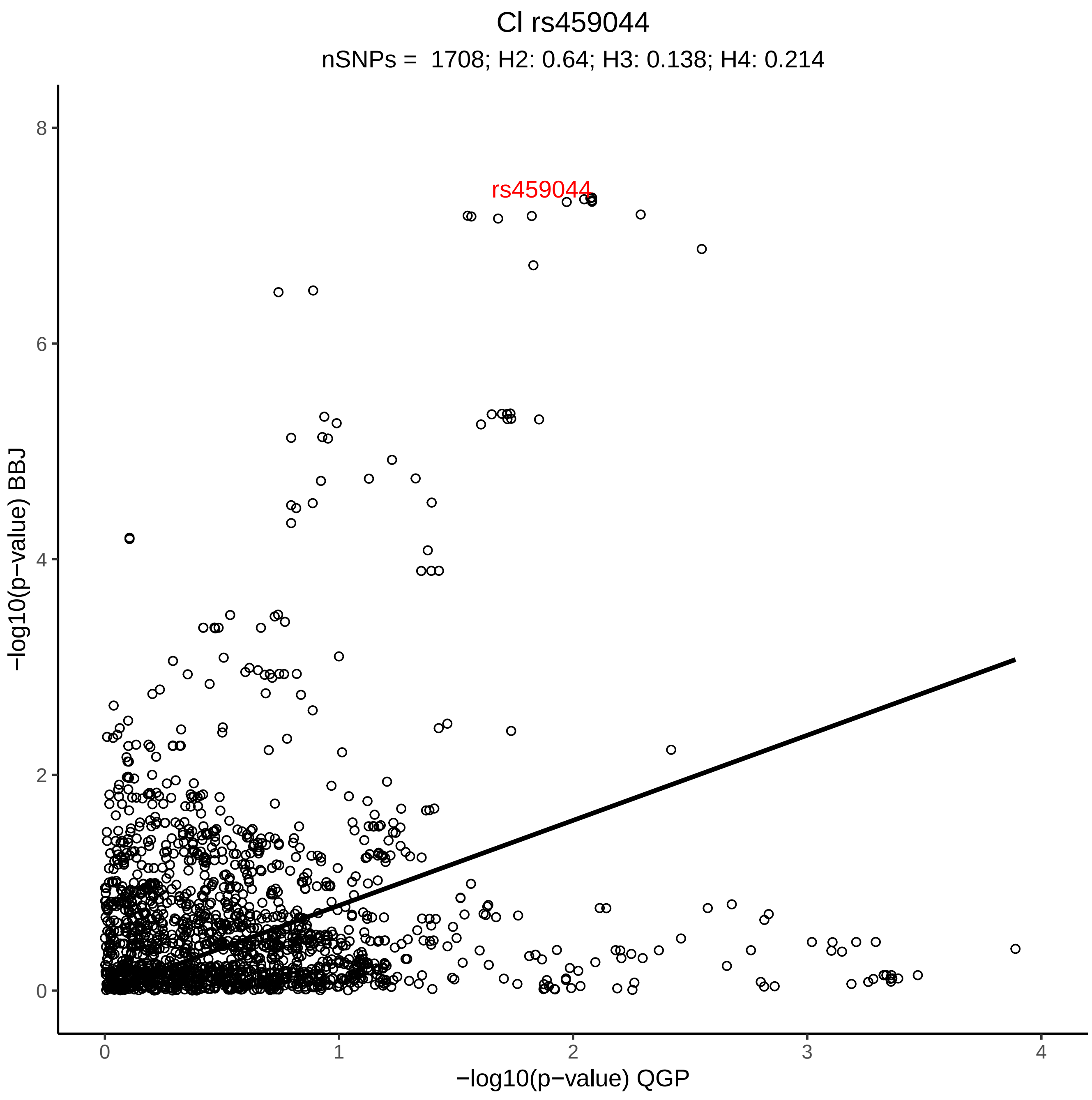
Cl rs459044
nSNPs = 1708; H2: 0.64; H3: 0.138; H4: 0.214
rs459044
−log10(p−value) BBJ
−log10(p−value) QGP
0
2
4
6
8
0
1
2
3
4

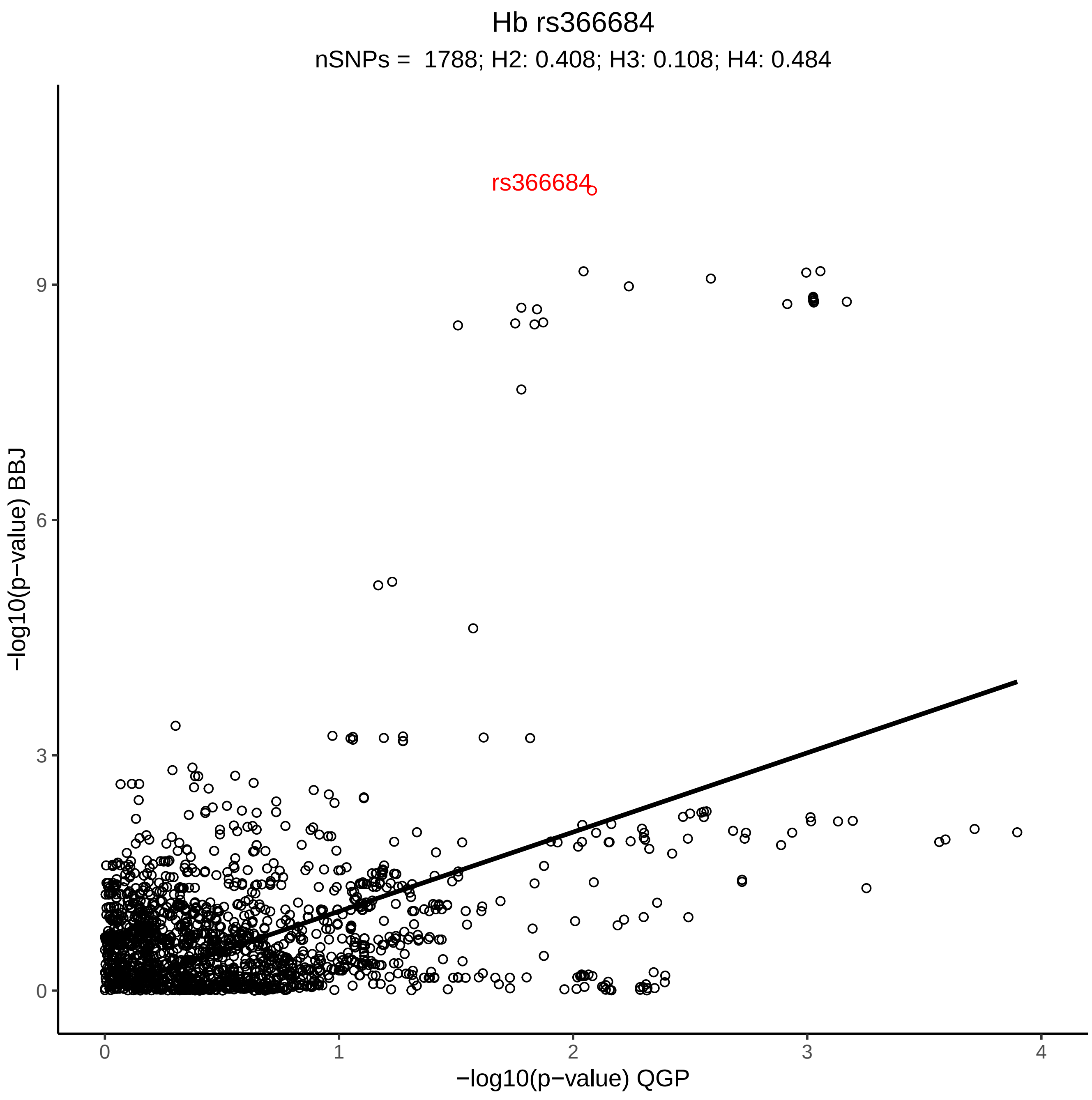

Hb rs366684
nSNPs = 1788; H2: 0.408; H3: 0.108; H4: 0.484
rs366684
−log10(p−value) BBJ
9
6
3
0
0
1
2
3
4
−log10(p−value) QGP

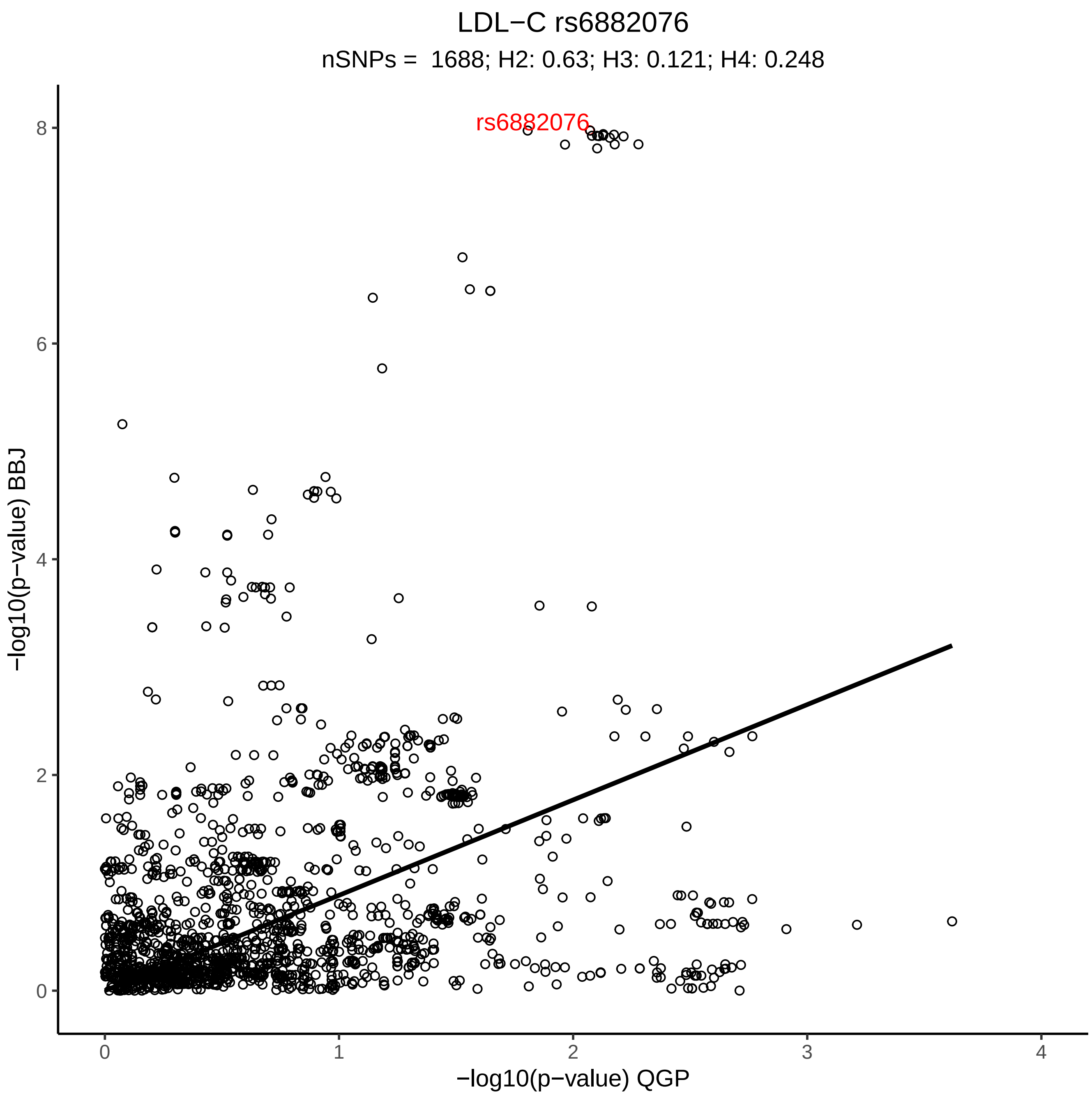

LDL−C rs6882076
nSNPs = 1688; H2: 0.63; H3: 0.121; H4: 0.248
rs6882076
−log10(p−value) BBJ
−log10(p−value) QGP
0
2
4
6
8
0
1
2
3
4

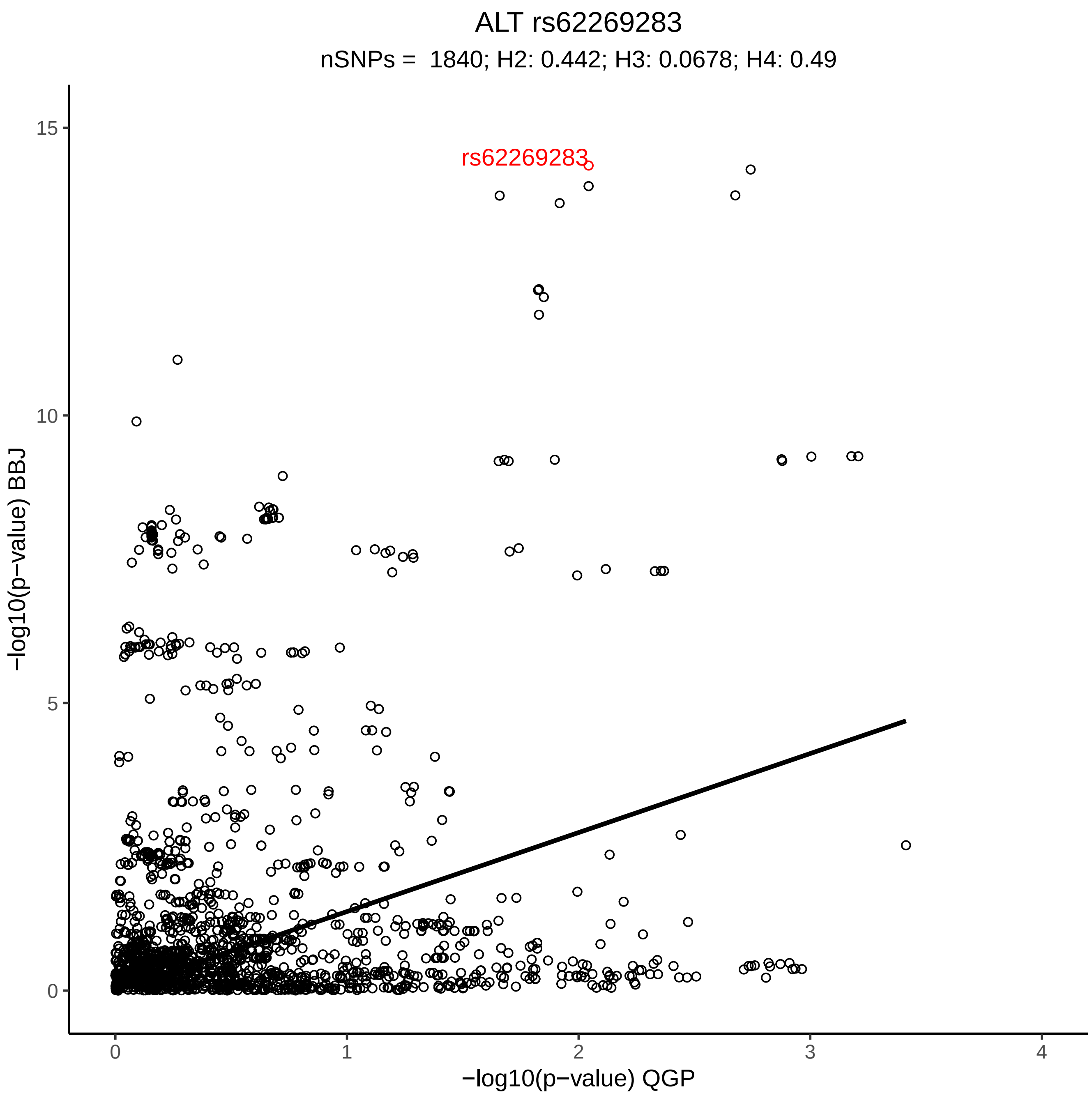
ALT rs62269283
nSNPs = 1840; H2: 0.442; H3: 0.0678; H4: 0.49
rs62269283
−log10(p−value) BBJ
−log10(p−value) QGP
0
5
10
15
0
1
2
3
4

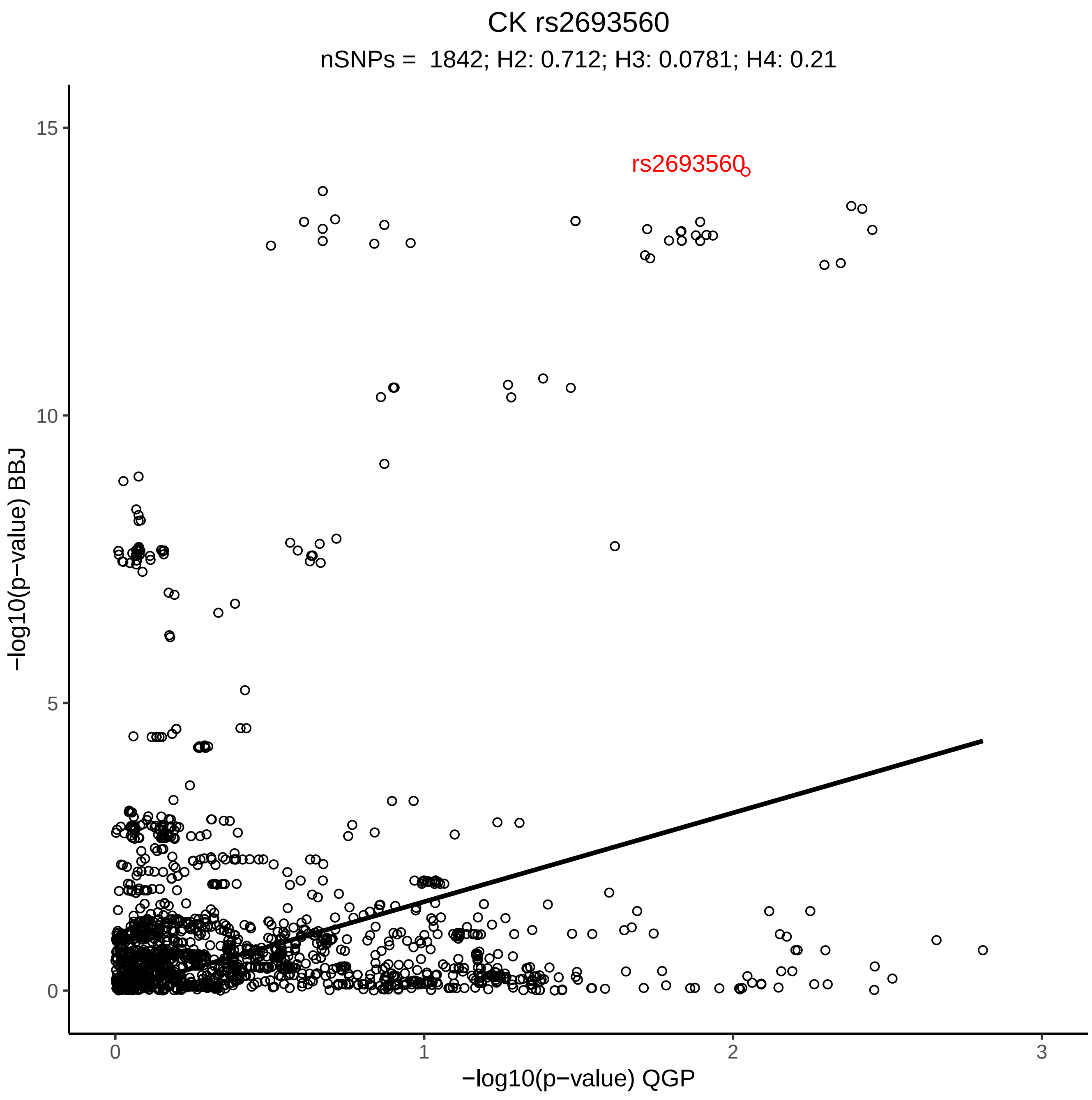
CK rs2693560
nSNPs = 1842; H2: 0.712; H3: 0.0781; H4: 0.21
rs2693560
15
10
5
0
−log10(p−value) BBJ
0
1
2
3
−log10(p−value) QGP

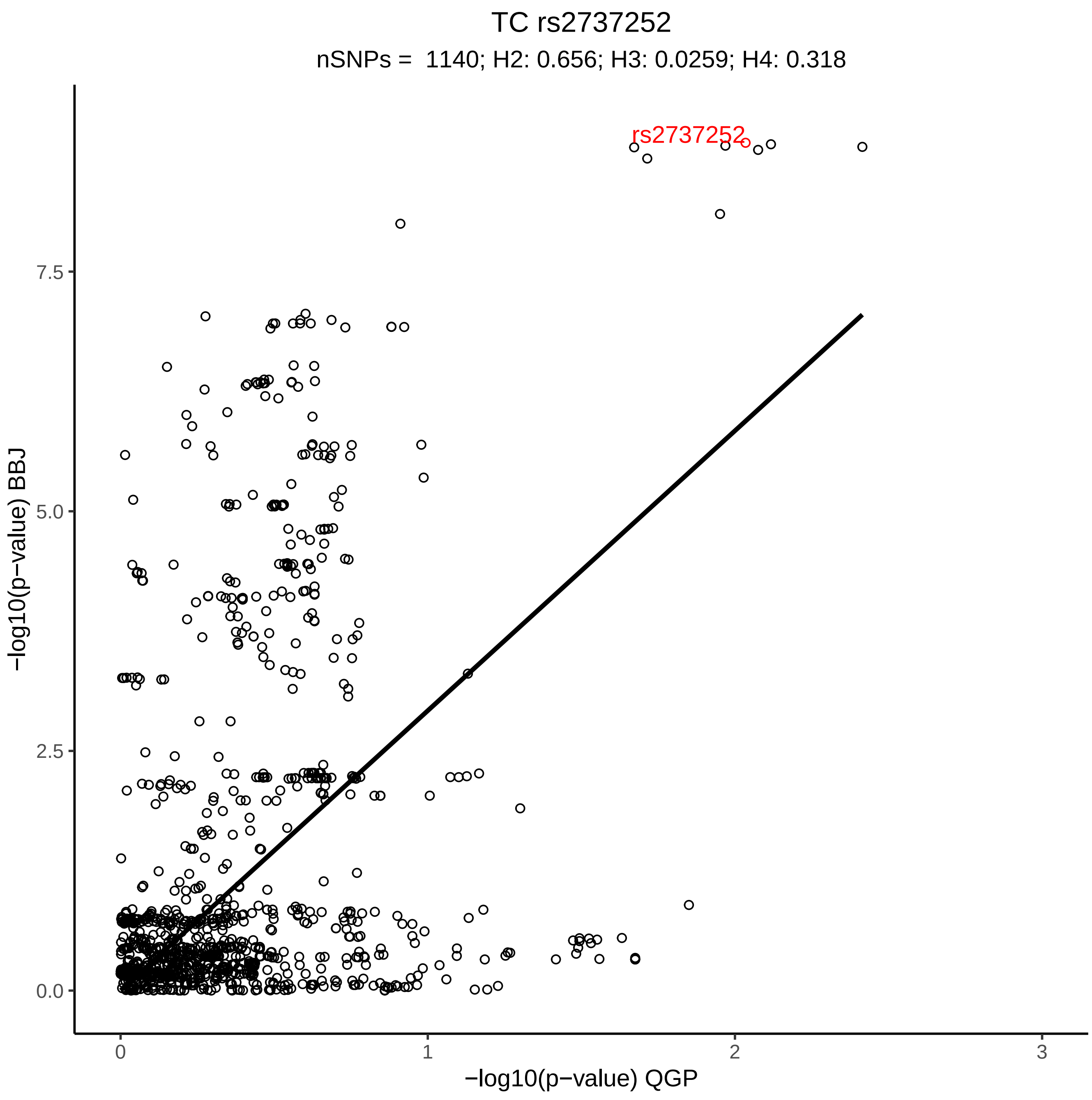
TC rs2737252
nSNPs = 1140; H2: 0.656; H3: 0.0259; H4: 0.318
rs2737252
7.5
5.0
2.5
0.0
−log10(p−value) BBJ
0
1
2
3
−log10(p−value) QGP

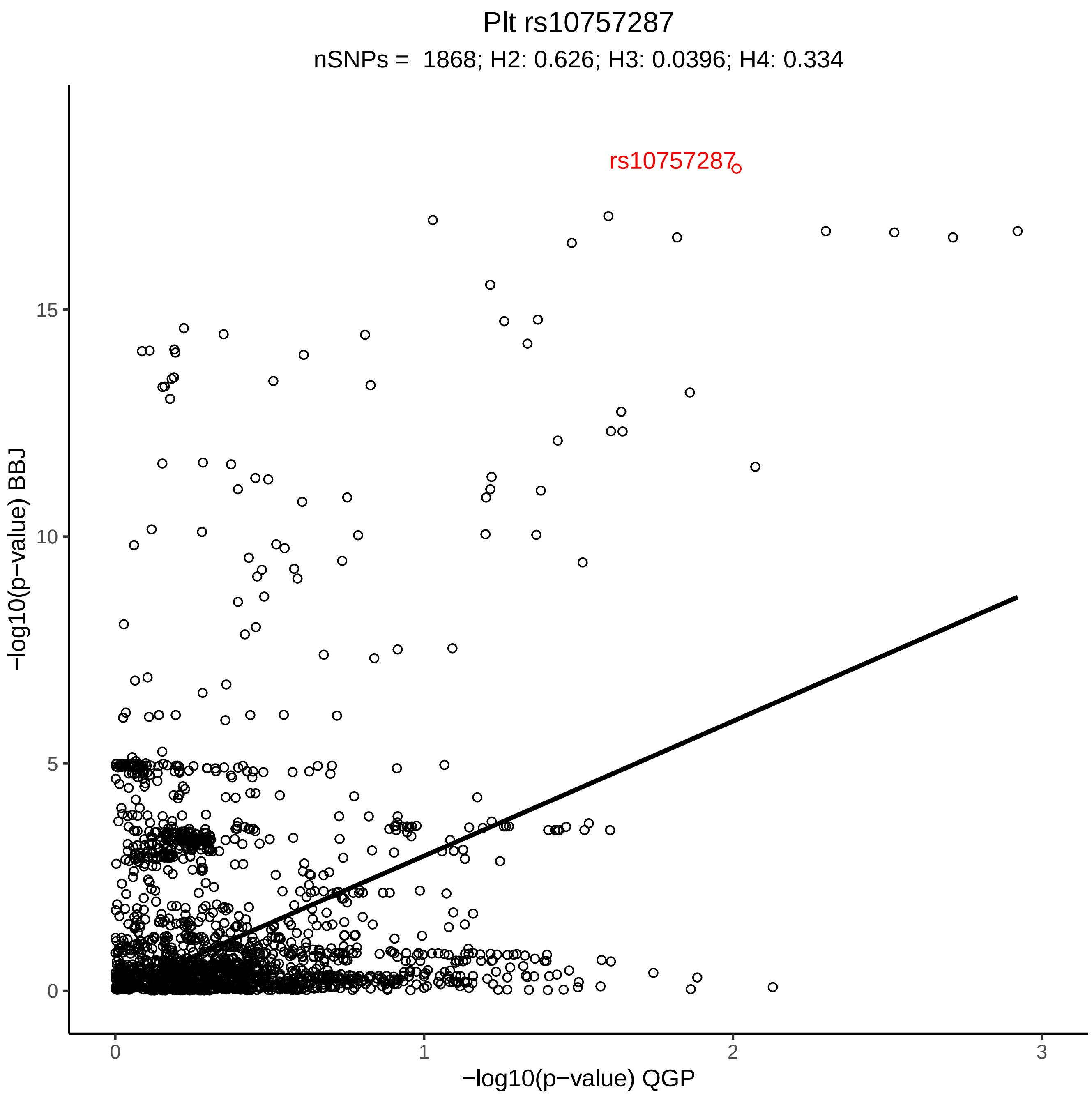
Plt rs10757287
nSNPs = 1868; H2: 0.626; H3: 0.0396; H4: 0.334
rs10757287
−log10(p−value) BBJ
−log10(p−value) QGP
0
5
10
15
0
1
2
3

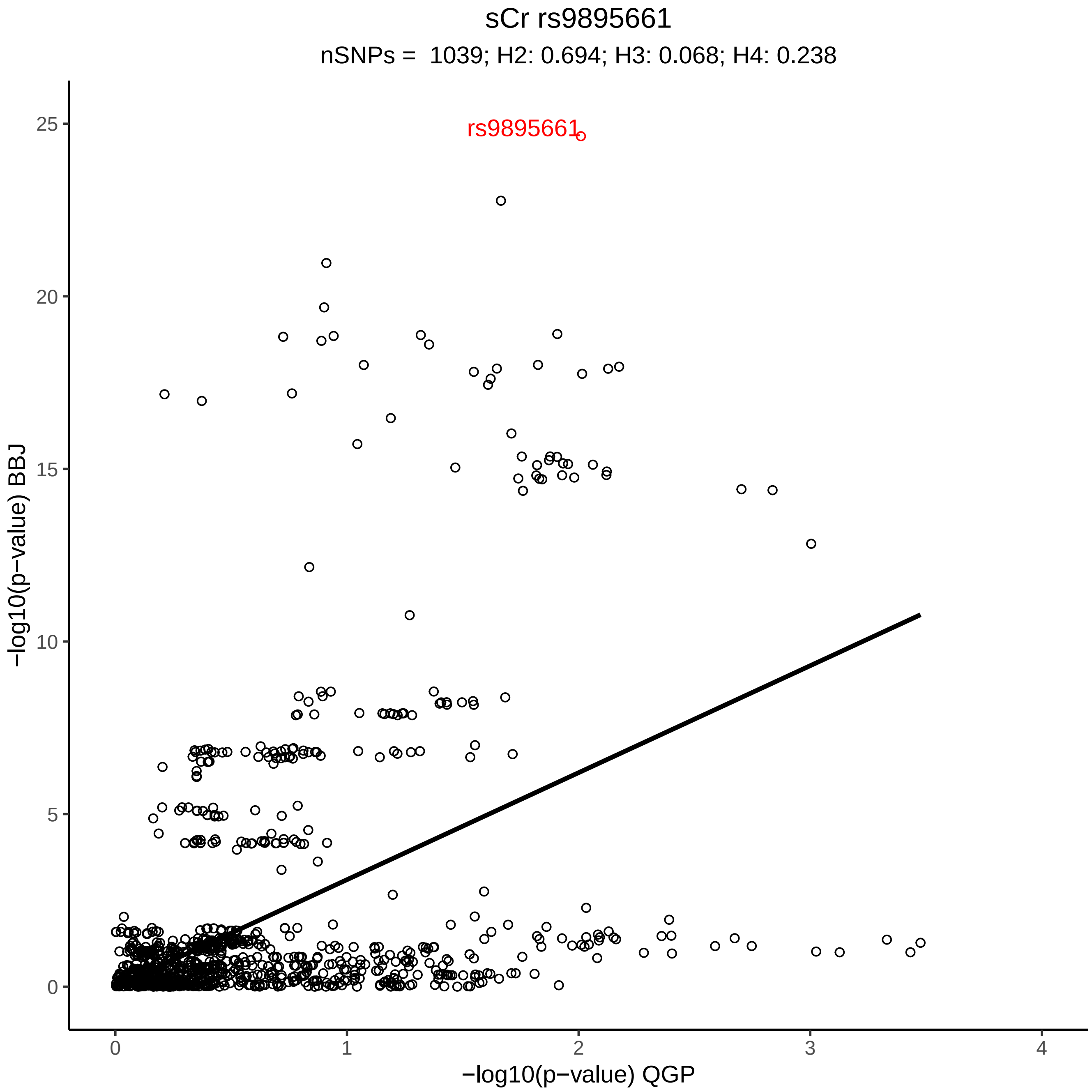
sCr rs9895661
nSNPs = 1039; H2: 0.694; H3: 0.068; H4: 0.238
rs9895661
−log10(p−value) BBJ
−log10(p−value) QGP
0
5
10
15
20
25
0
1
2
3
4

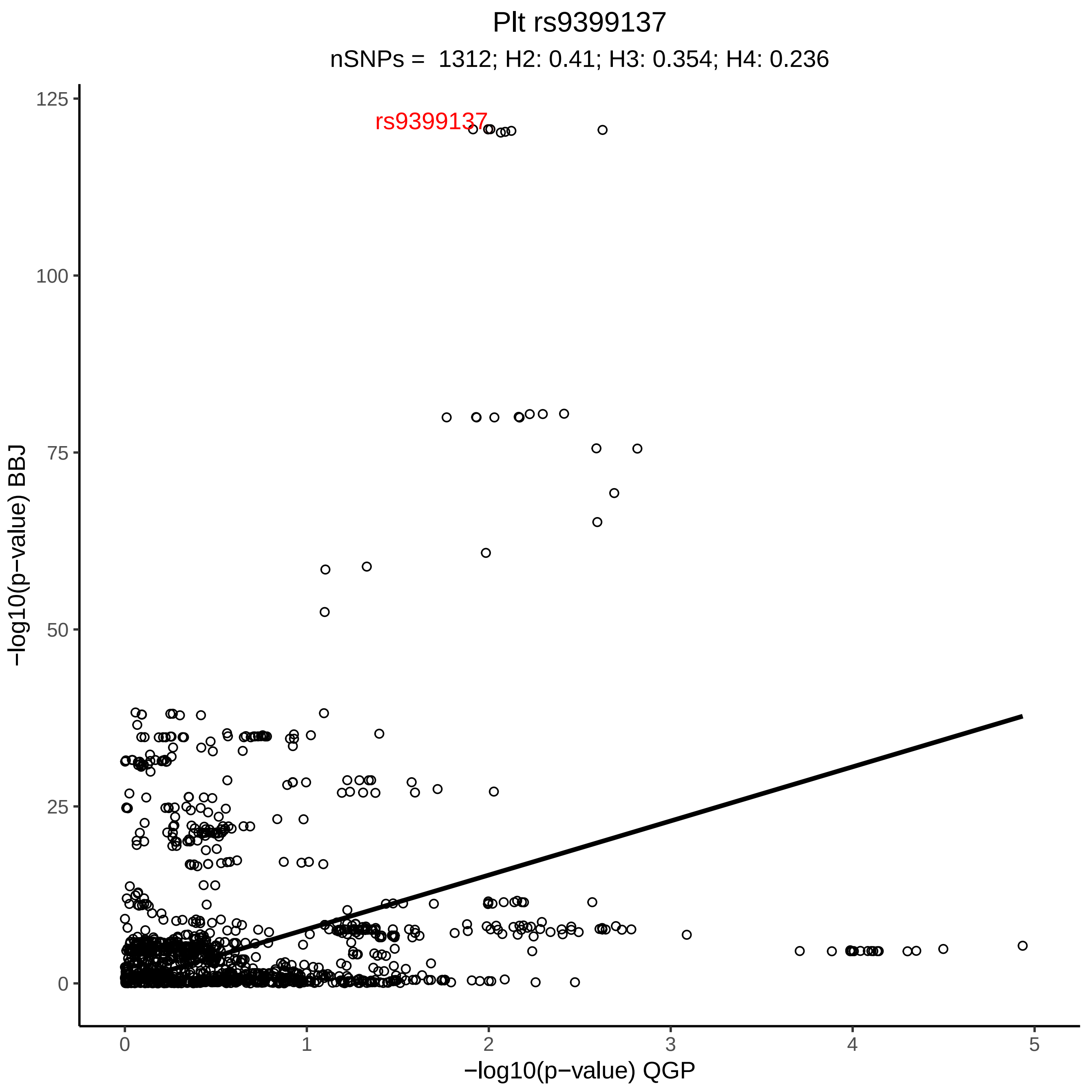
Plt rs9399137
nSNPs = 1312; H2: 0.41; H3: 0.354; H4: 0.236
rs9399137
−log10(p−value) BBJ
−log10(p−value) QGP
0
25
50
75
100
125
0
1
2
3
4
5

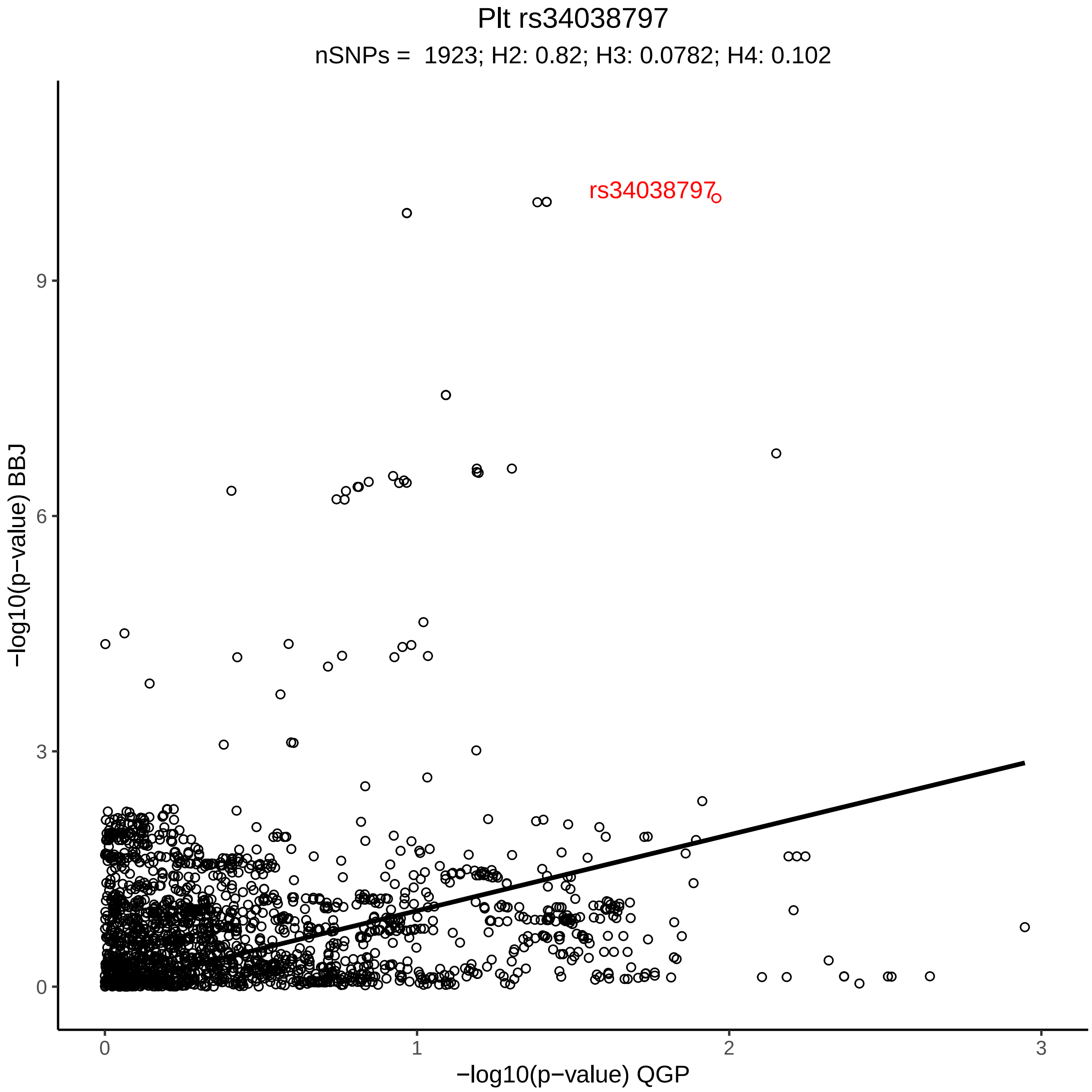

Plt rs34038797
nSNPs = 1923; H2: 0.82; H3: 0.0782; H4: 0.102
rs34038797
−log10(p−value) BBJ
−log10(p−value) QGP
0
3
6
9
0
1
2
3

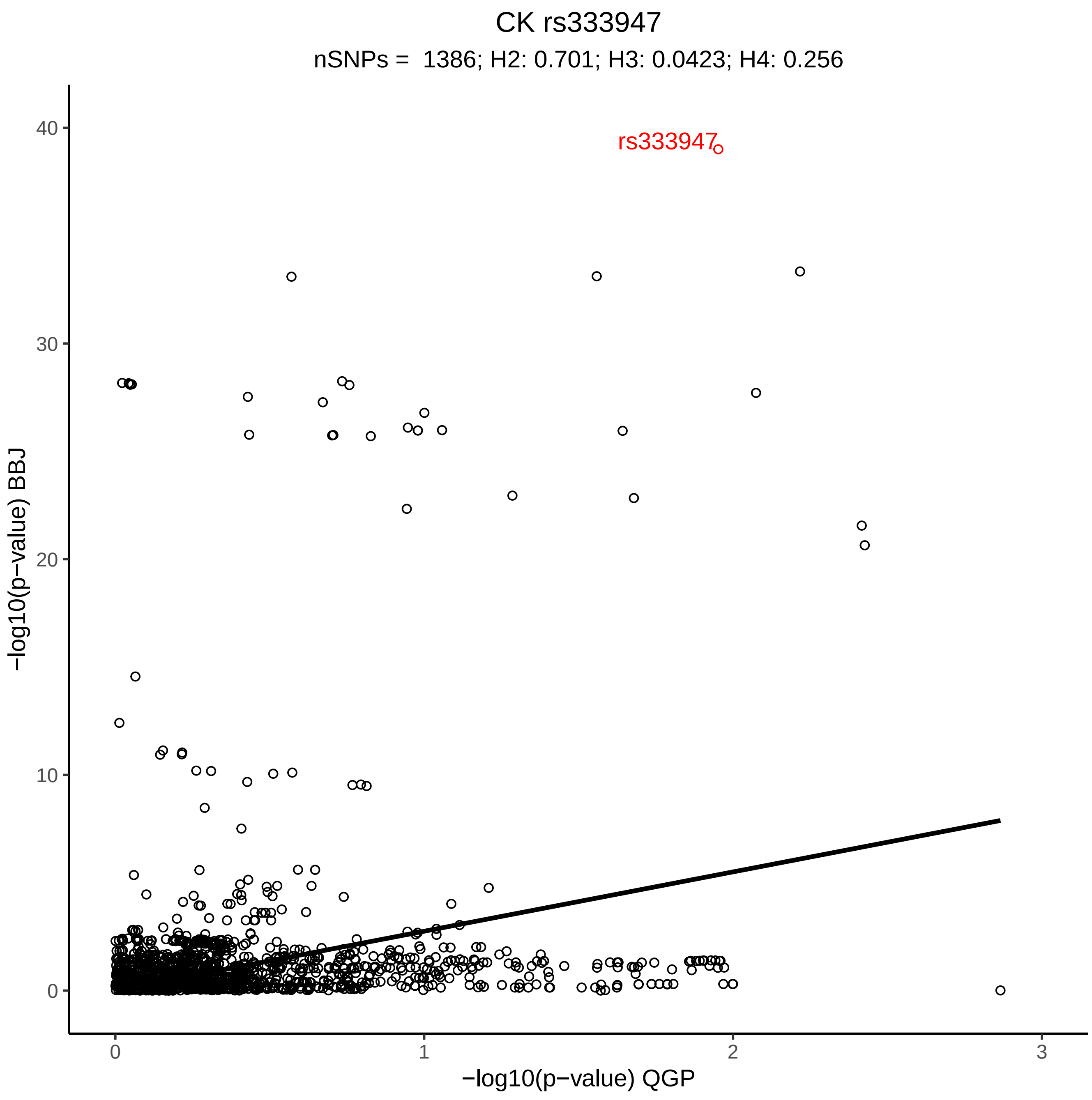
CK rs333947
nSNPs = 1386; H2: 0.701; H3: 0.0423; H4: 0.256
rs333947
−log10(p−value) BBJ
−log10(p−value) QGP
0
10
20
30
40
0
1
2
3

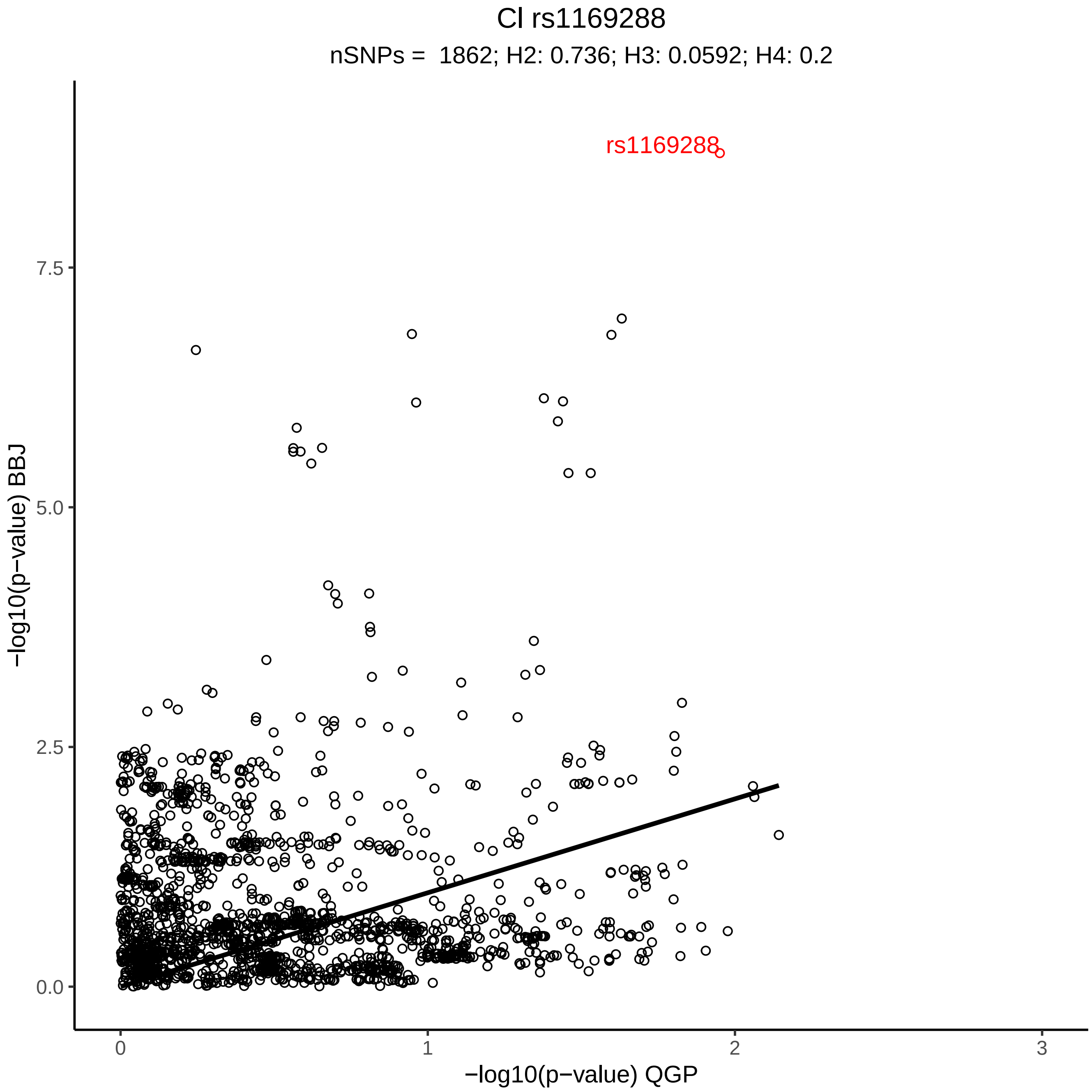

CI rs1169288
nSNPs = 1862; H2: 0.736; H3: 0.0592; H4: 0.2
rs1169288
−log10(p−value) BBJ
−log10(p−value) QGP
0.0
2.5
5.0
7.5
0
1
2
3

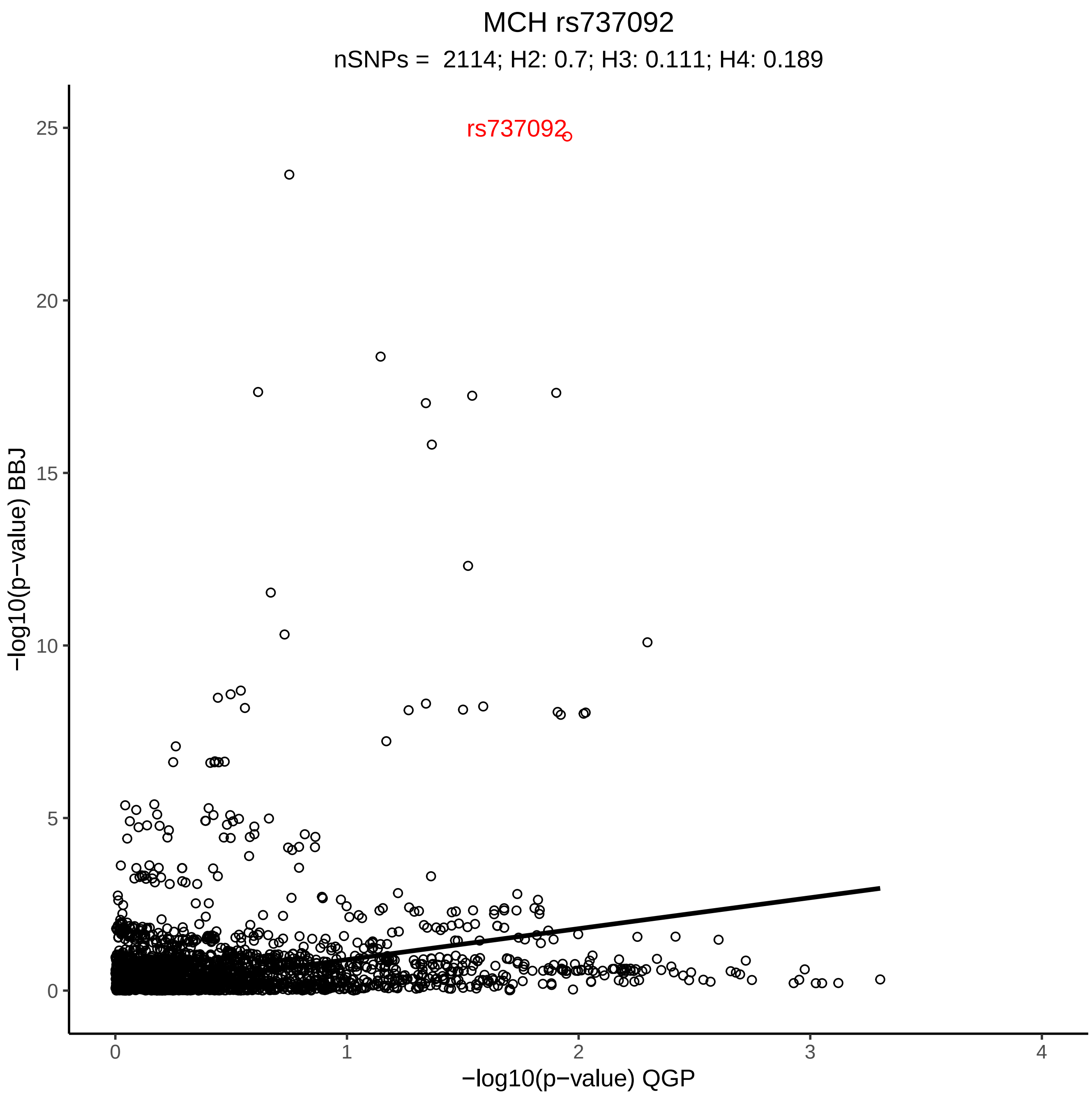

MCH rs737092
nSNPs = 2114; H2: 0.7; H3: 0.111; H4: 0.189
rs737092
−log10(p−value) BBJ
−log10(p−value) QGP
0
5
10
15
20
25
0
1
2
3
4

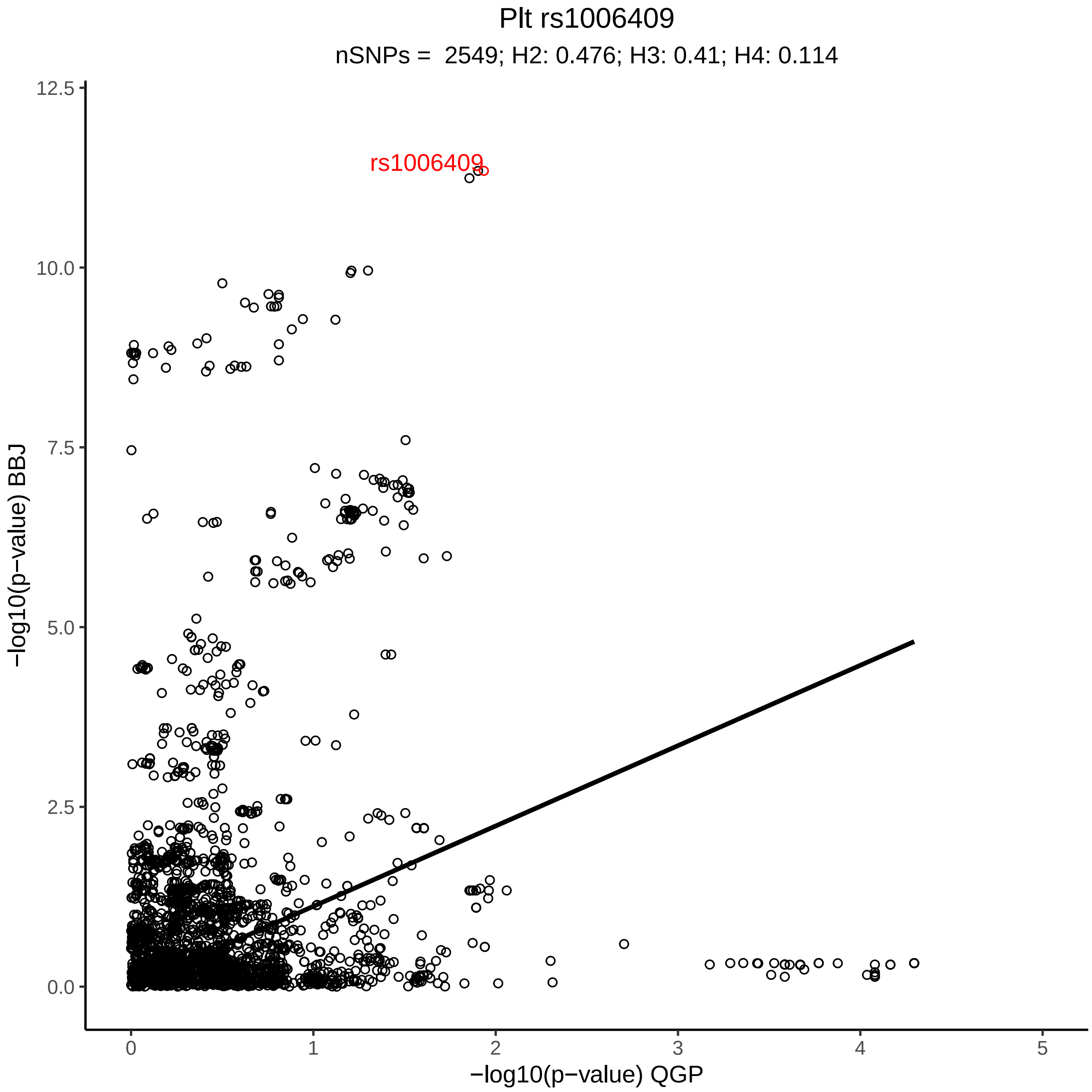
Plt rs1006409
nSNPs = 2549; H2: 0.476; H3: 0.41; H4: 0.114
rs1006409
12.5
10.0
7.5
5.0
2.5
0.0
−log10(p−value) BBJ
0
1
2
3
4
5
−log10(p−value) QGP

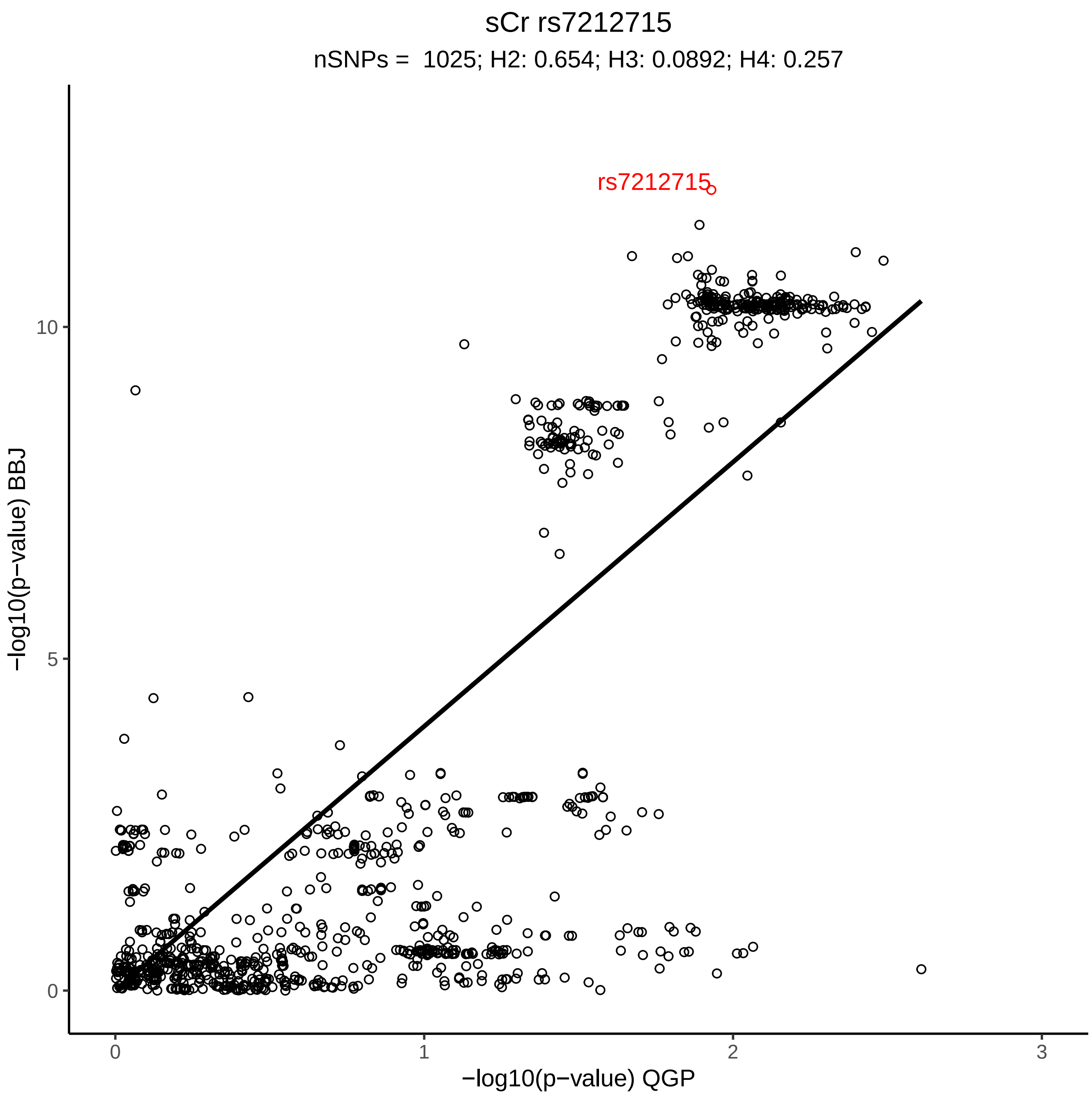
sCr rs7212715
nSNPs = 1025; H2: 0.654; H3: 0.0892; H4: 0.257
rs7212715
−log10(p−value) BBJ
−log10(p−value) QGP
0
5
10
0
1
2
3

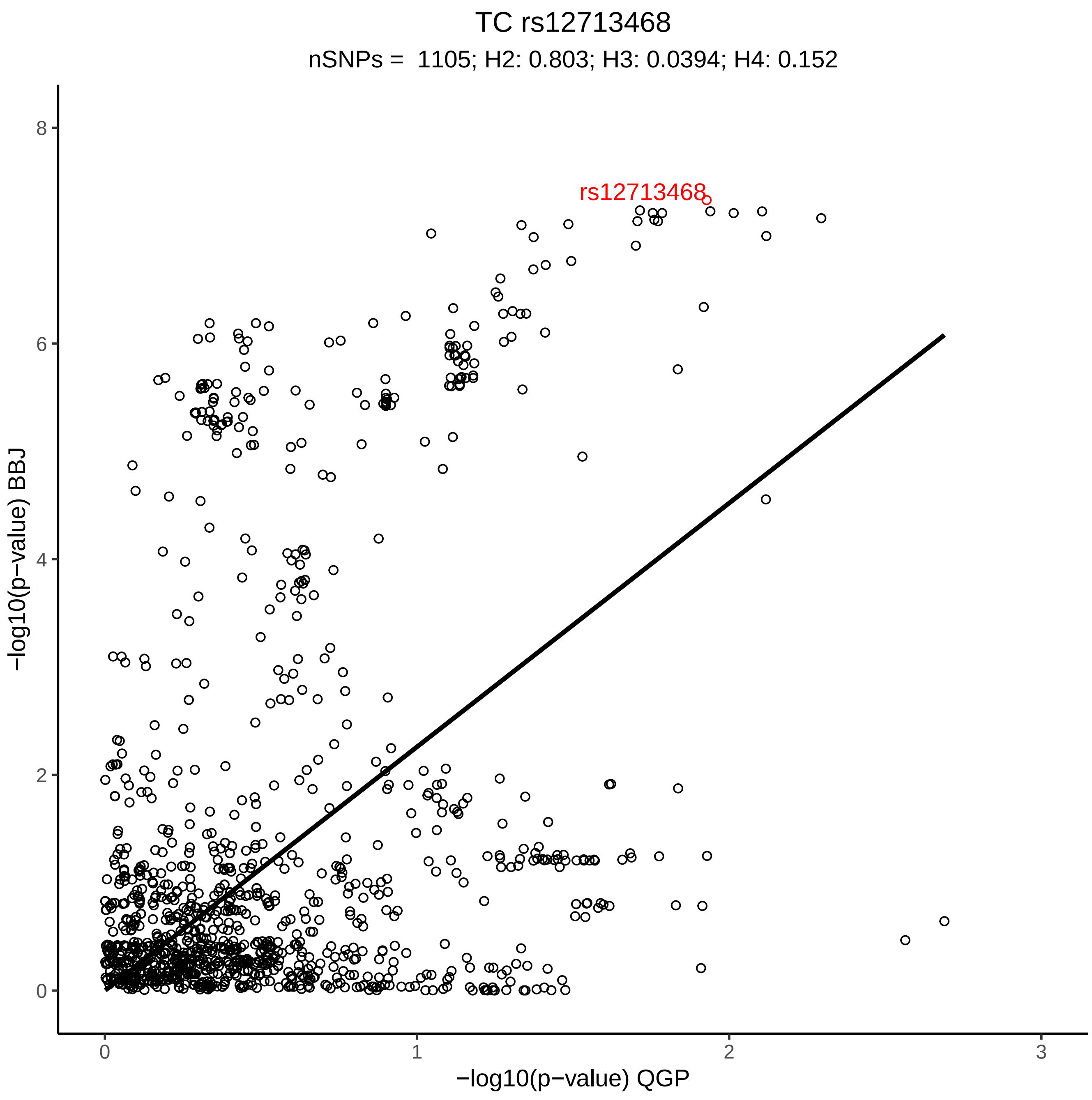
TC rs12713468
nSNPs = 1105; H2: 0.803; H3: 0.0394; H4: 0.152
rs12713468
−log10(p−value) BBJ
−log10(p−value) QGP
0
2
4
6
8
0
1
2
3

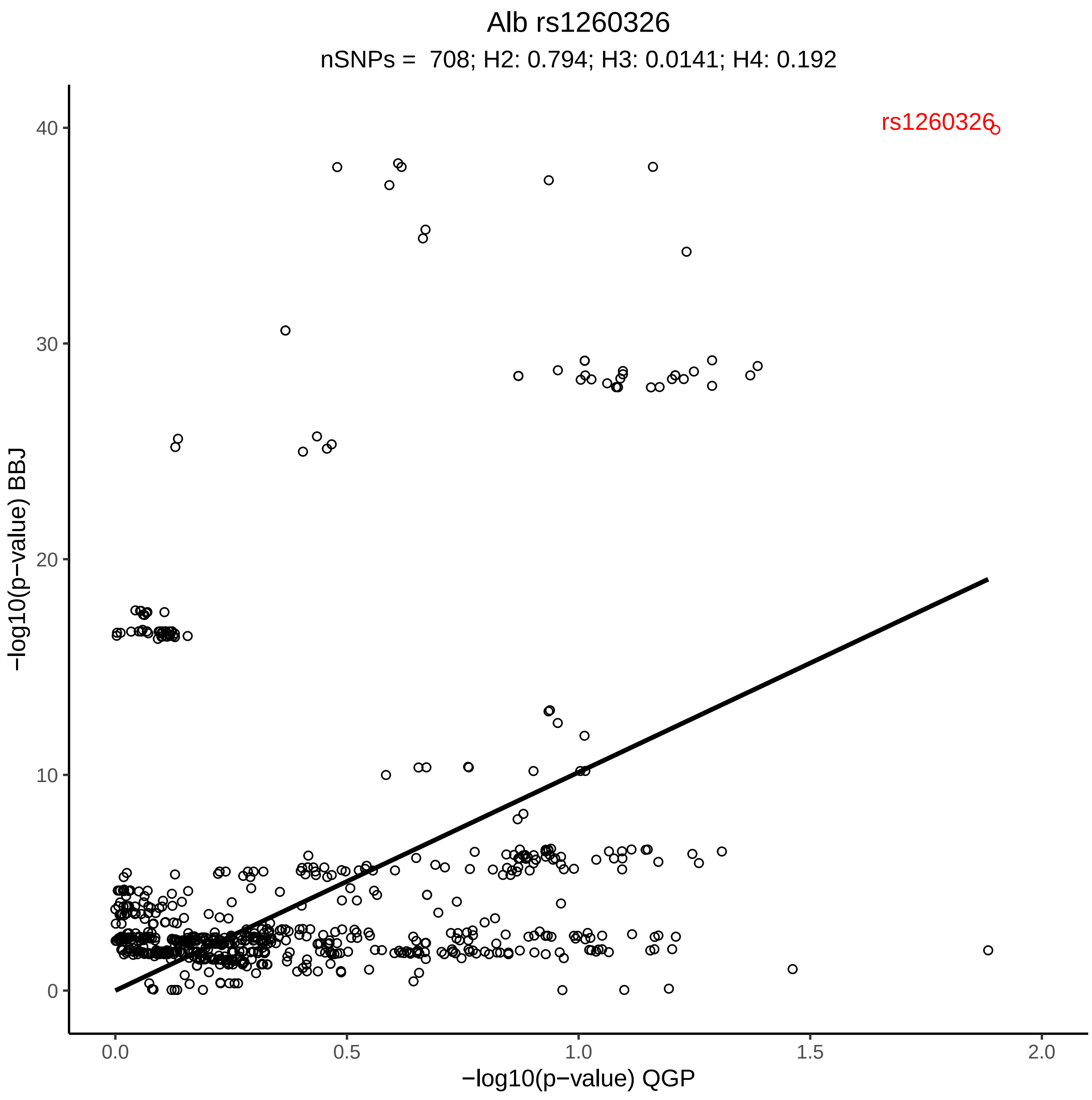

Alb rs1260326
nSNPs = 708; H2: 0.794; H3: 0.0141; H4: 0.192
rs1260326
−log10(p−value) BBJ
0
10
20
30
40
0.0
0.5
1.0
1.5
2.0
−log10(p−value) QGP

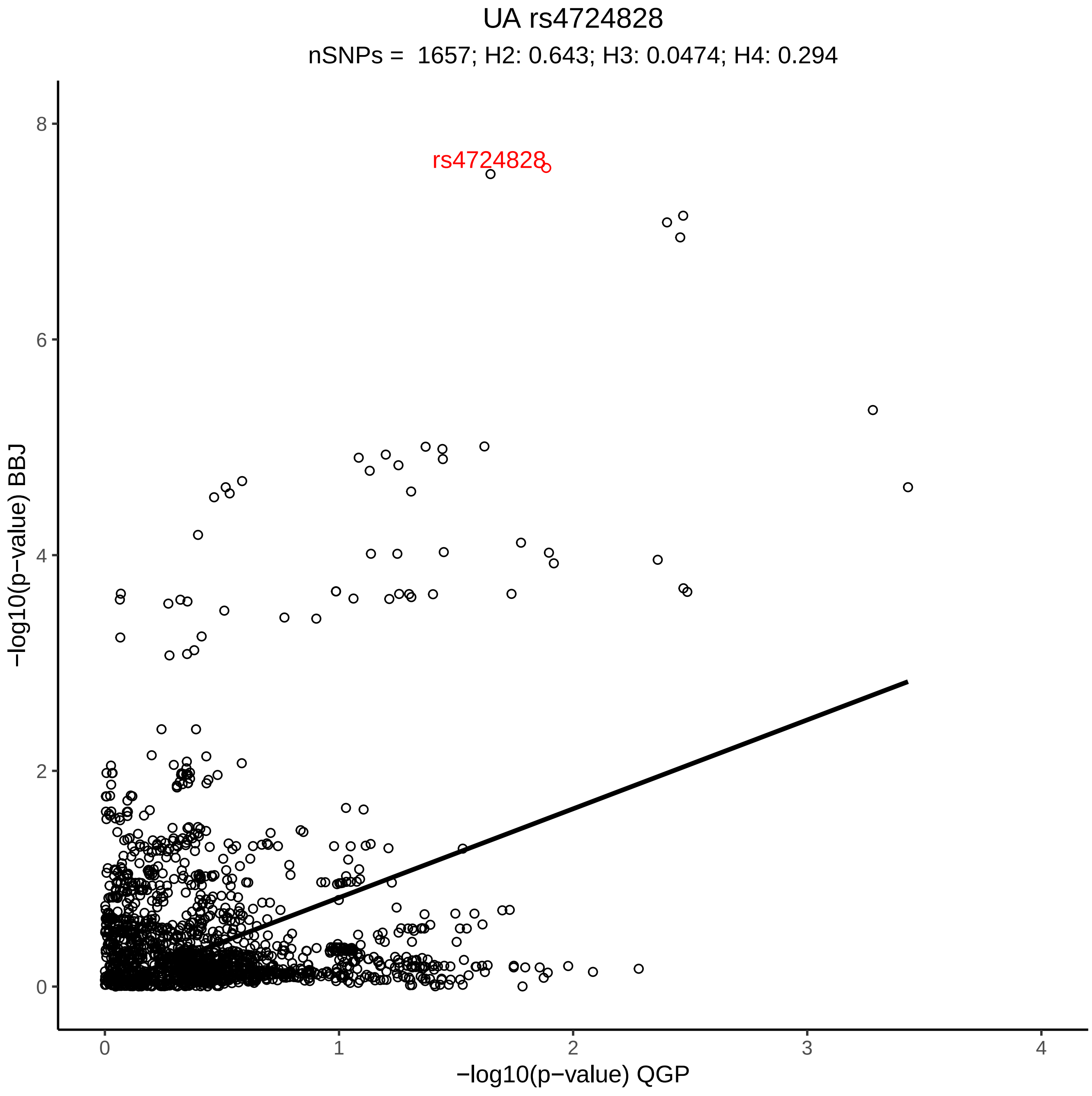
UA rs4724828
nSNPs = 1657; H2: 0.643; H3: 0.0474; H4: 0.294
rs4724828
−log10(p−value) BBJ
−log10(p−value) QGP
0
2
4
6
8
0
1
2
3
4

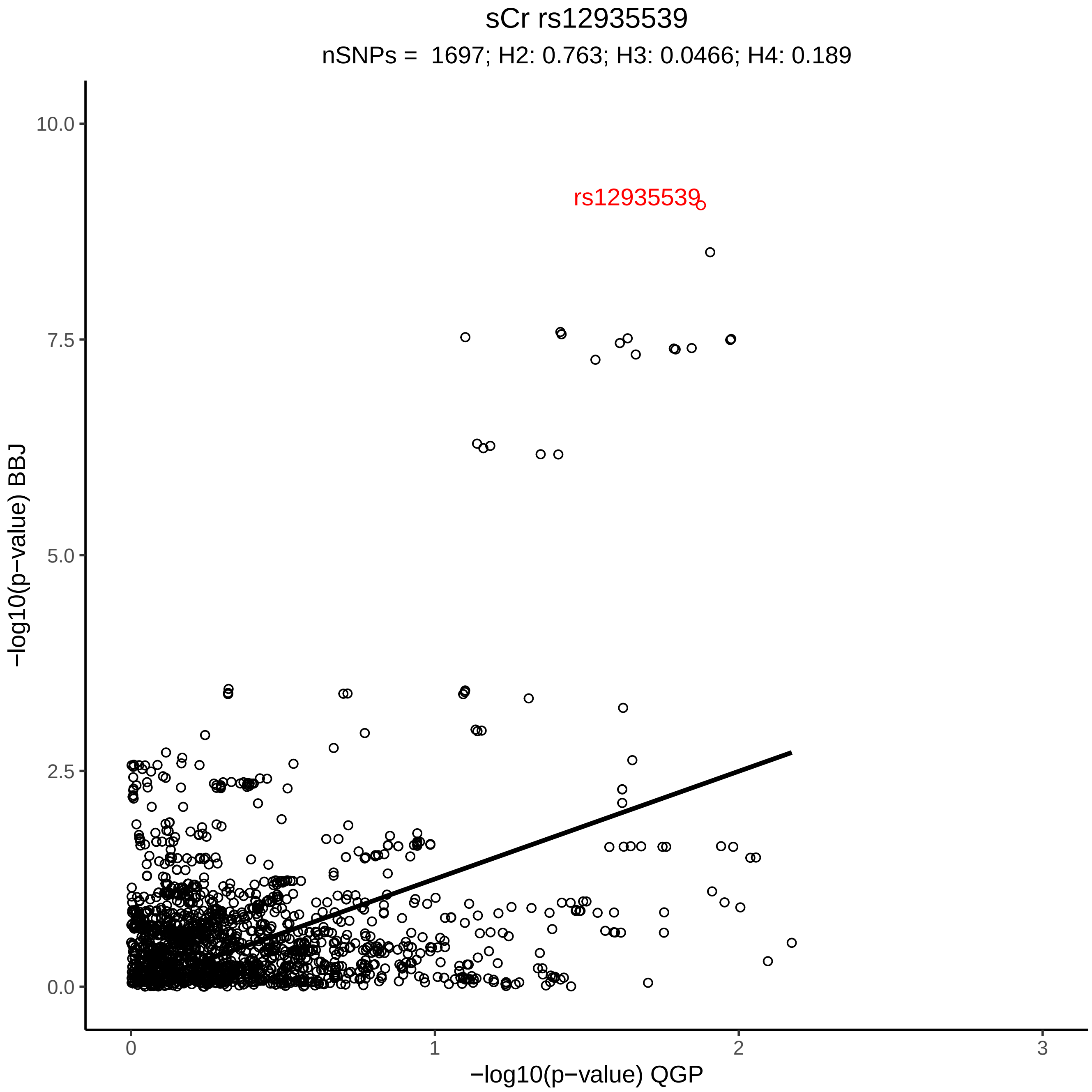
sCr rs12935539
nSNPs = 1697; H2: 0.763; H3: 0.0466; H4: 0.189
rs12935539
10.0
7.5
5.0
2.5
0.0
−log10(p−value) BBJ
0
1
2
3
−log10(p−value) QGP

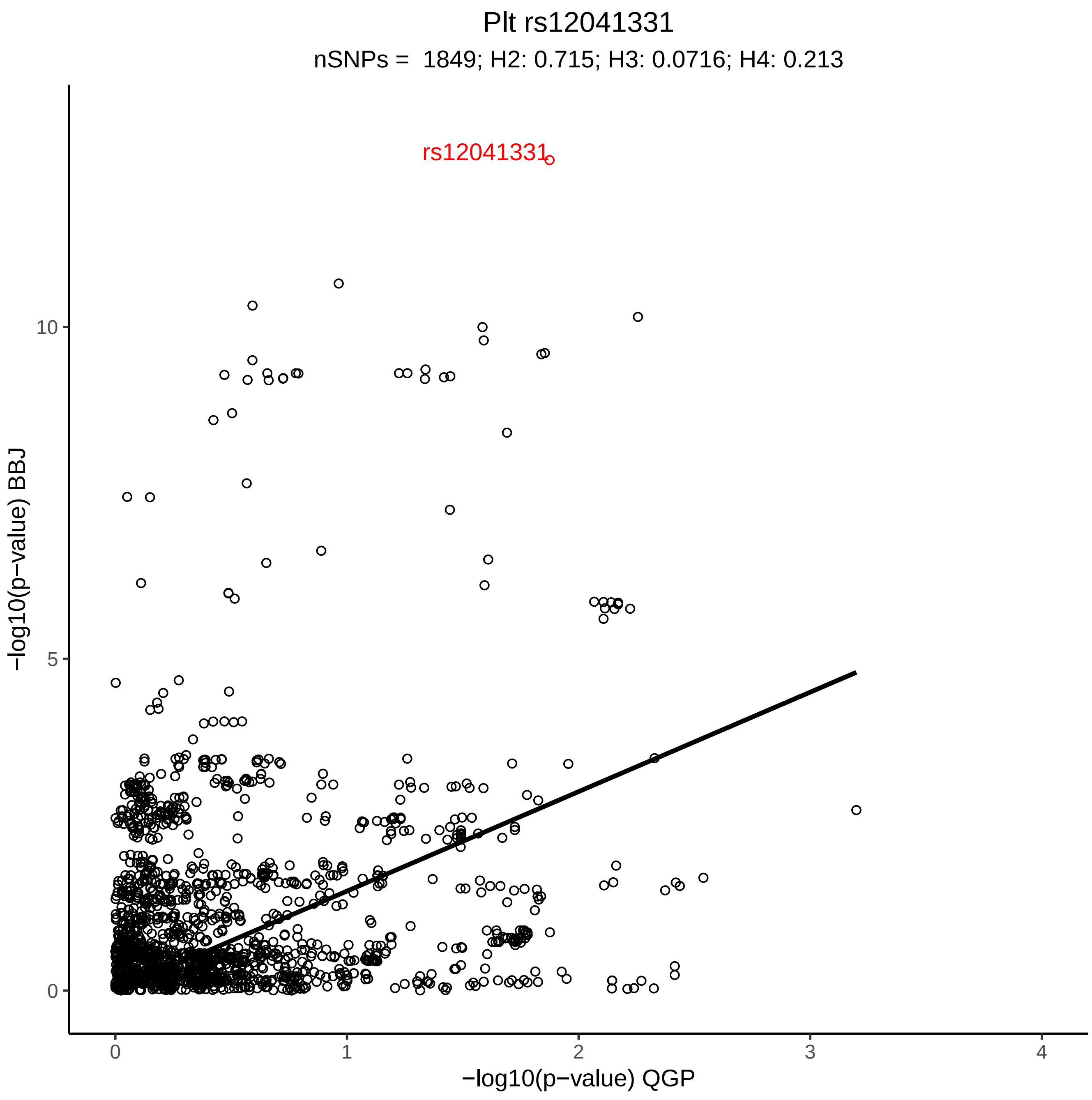
Plt rs12041331
nSNPs = 1849; H2: 0.715; H3: 0.0716; H4: 0.213
rs12041331
−log10(p−value) BBJ
−log10(p−value) QGP
0
5
10
0
1
2
3
4

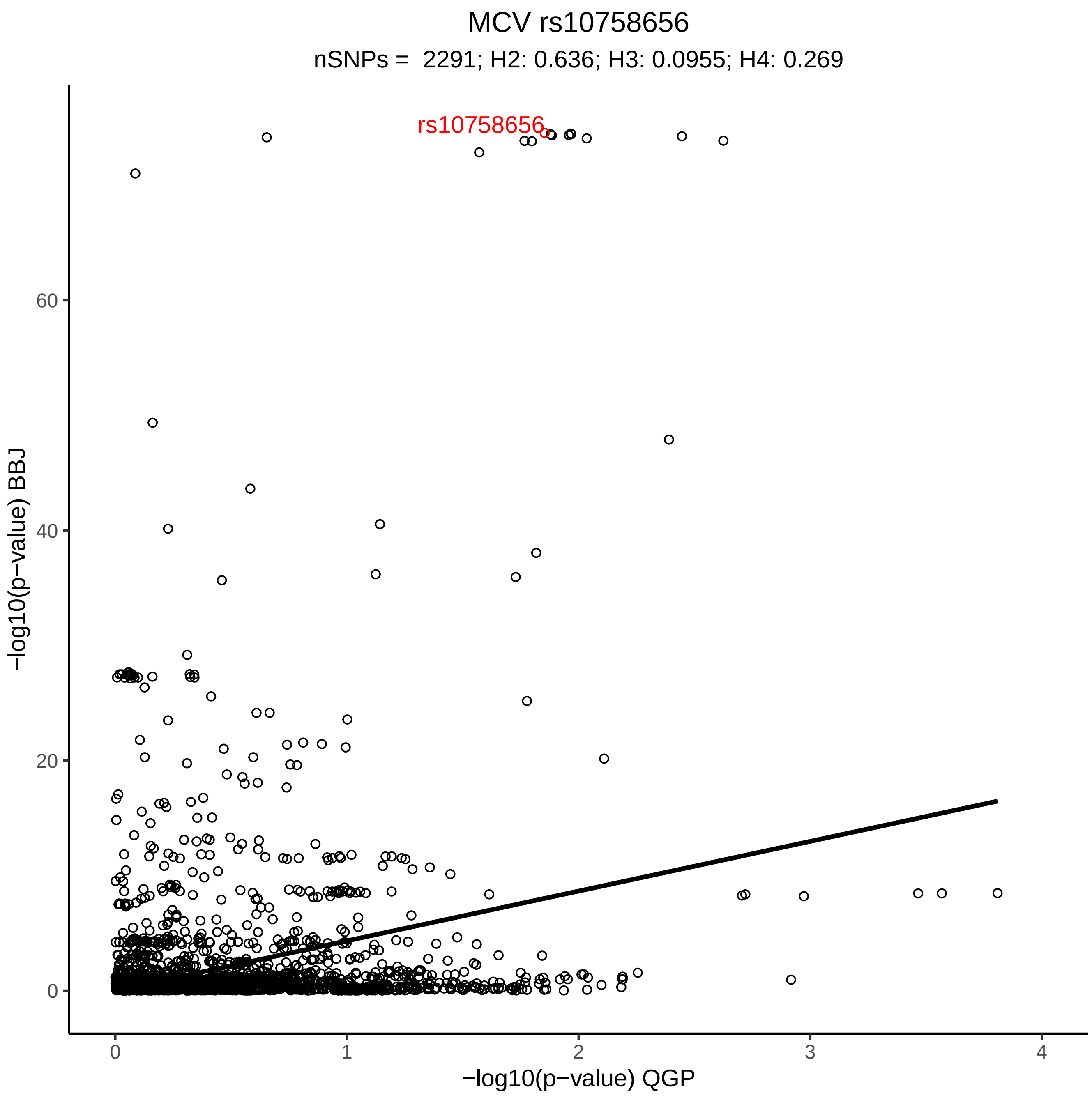

MCV rs10758656
nSNPs = 2291; H2: 0.636; H3: 0.0955; H4: 0.269
rs10758656
−log10(p−value) BBJ
0
20
40
60
0
1
2
3
4
−log10(p−value) QGP

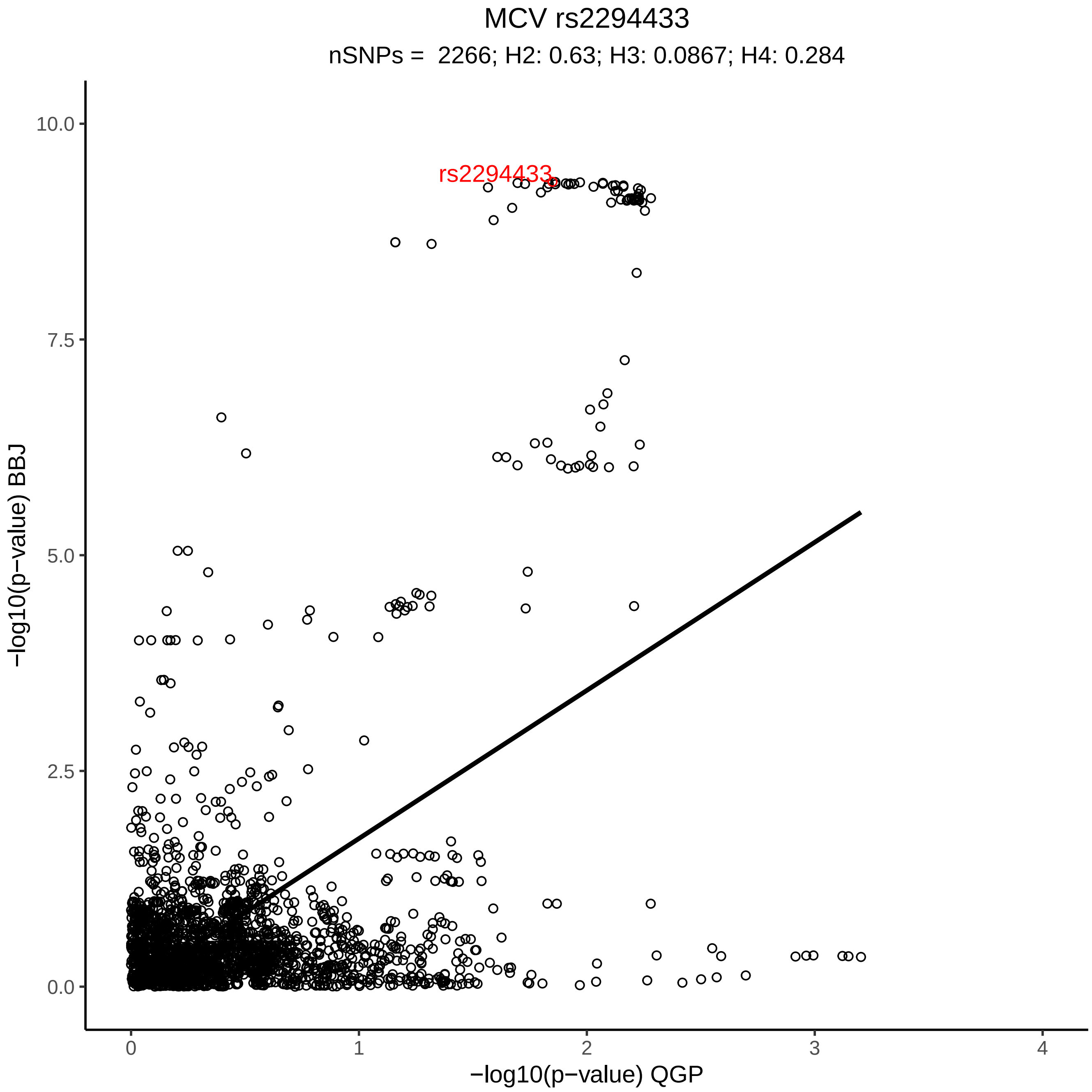

MCV rs2294433
nSNPs = 2266; H2: 0.63; H3: 0.0867; H4: 0.284
rs2294433
10.0
7.5
5.0
2.5
0.0
−log10(p−value) BBJ
0
1
2
3
4
−log10(p−value) QGP

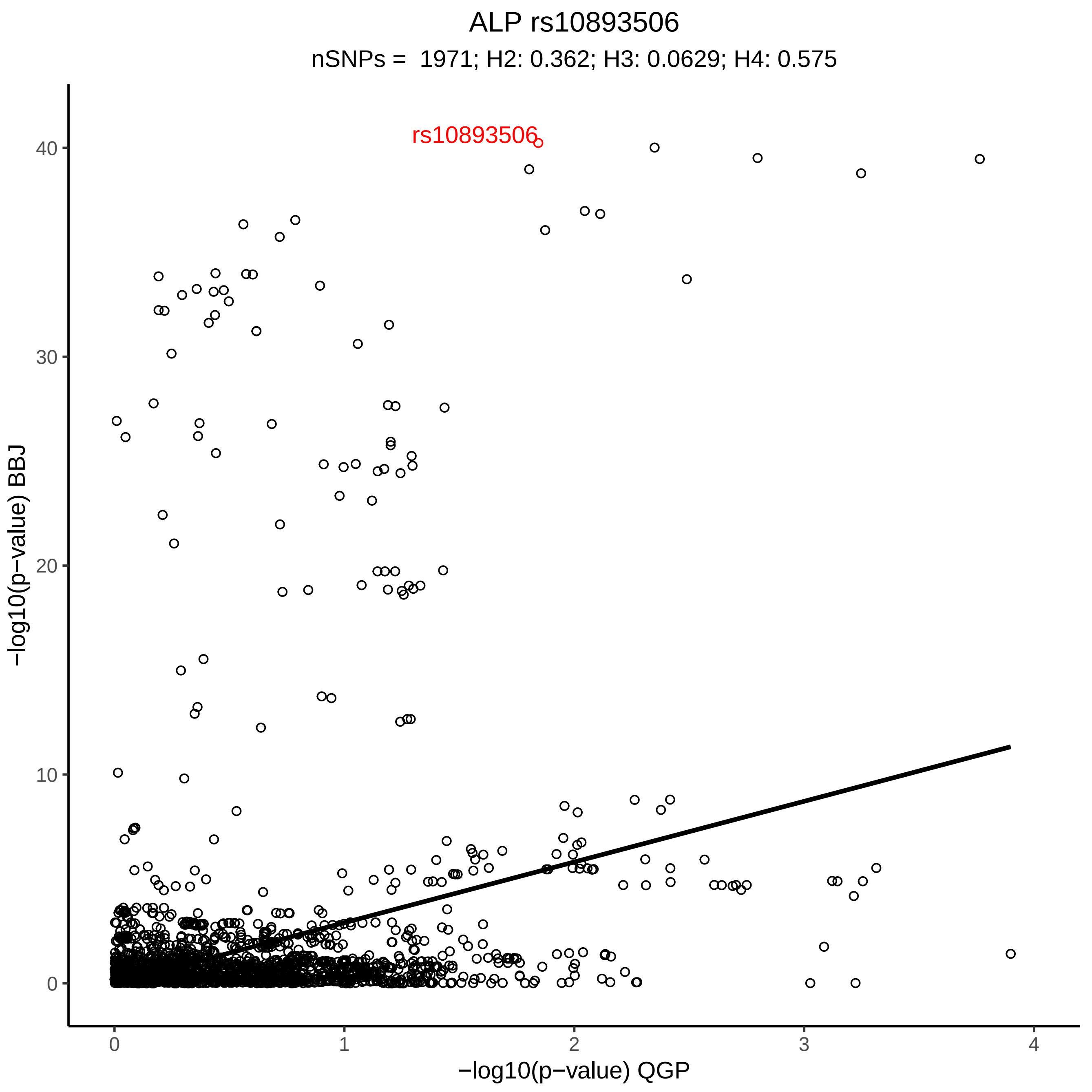
ALP rs10893506
nSNPs = 1971; H2: 0.362; H3: 0.0629; H4: 0.575
rs10893506
−log10(p−value) BBJ
−log10(p−value) QGP
0
10
20
30
40
0
1
2
3
4

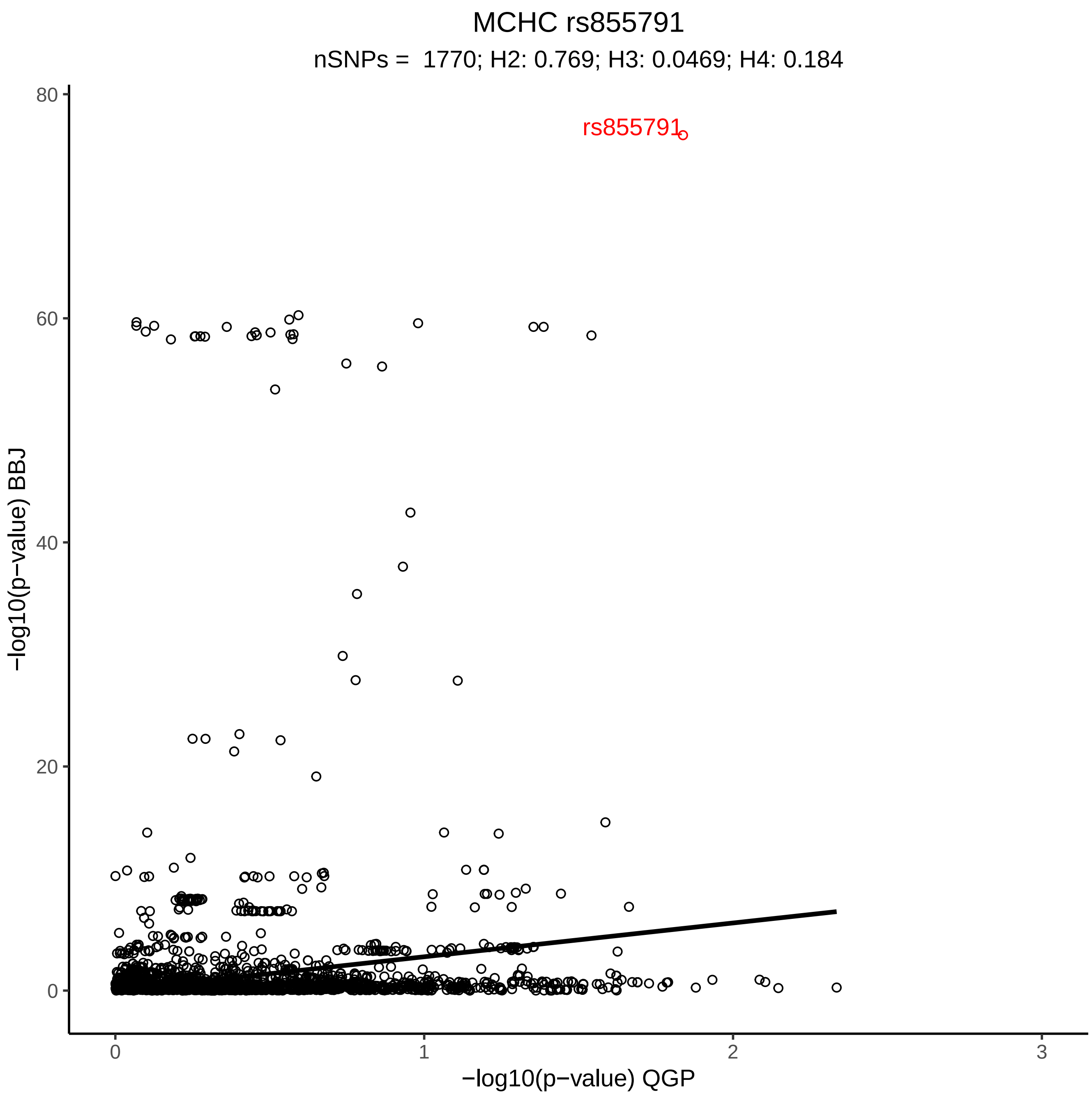
MCHC rs855791
nSNPs = 1770; H2: 0.769; H3: 0.0469; H4: 0.184
rs855791
−log10(p−value) BBJ
−log10(p−value) QGP
0
20
40
60
80
0
1
2
3

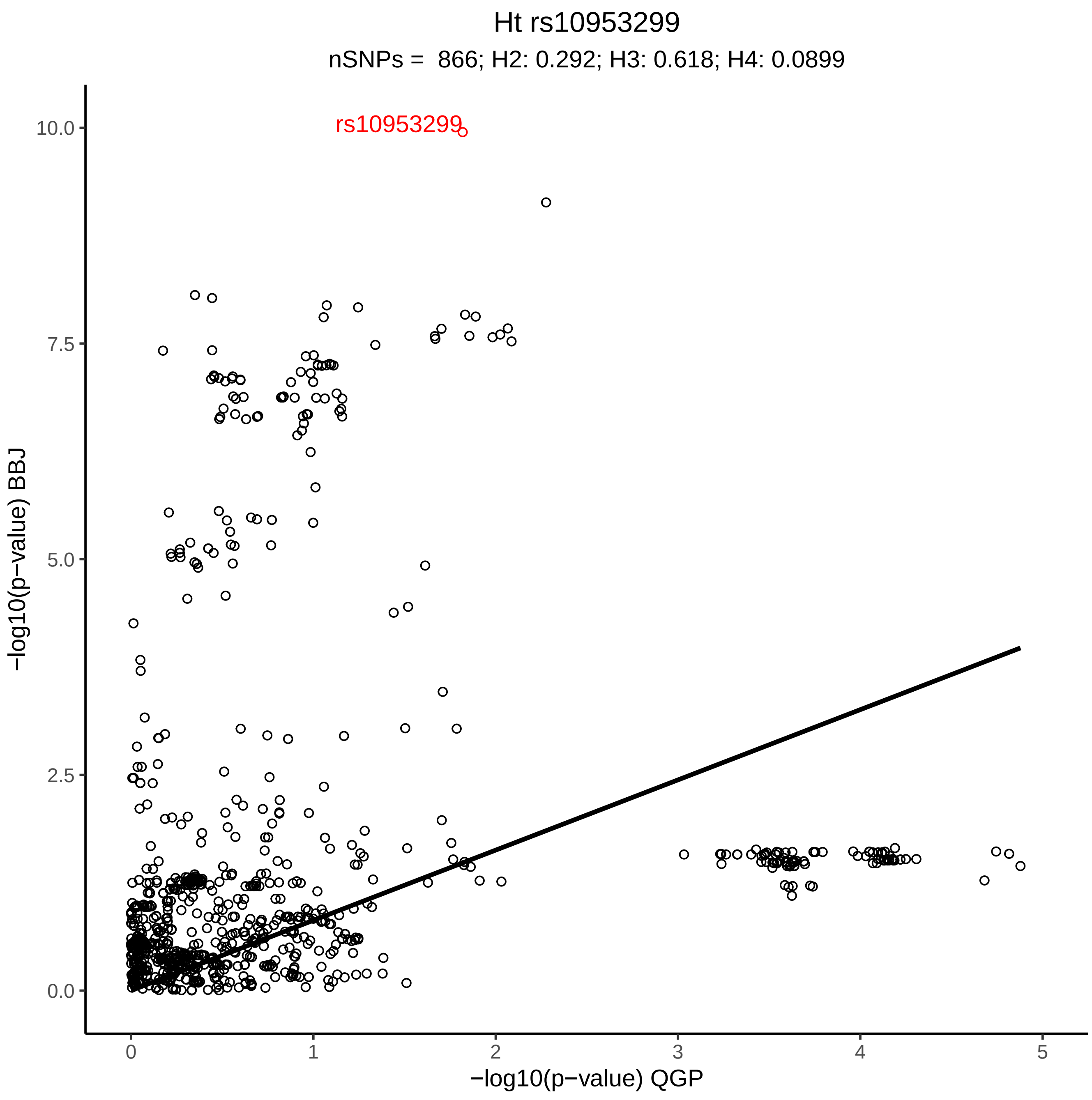
Ht rs10953299
nSNPs = 866; H2: 0.292; H3: 0.618; H4: 0.0899
rs10953299
10.0
7.5
5.0
2.5
0.0
−log10(p−value) BBJ
0
1
2
3
4
5
−log10(p−value) QGP

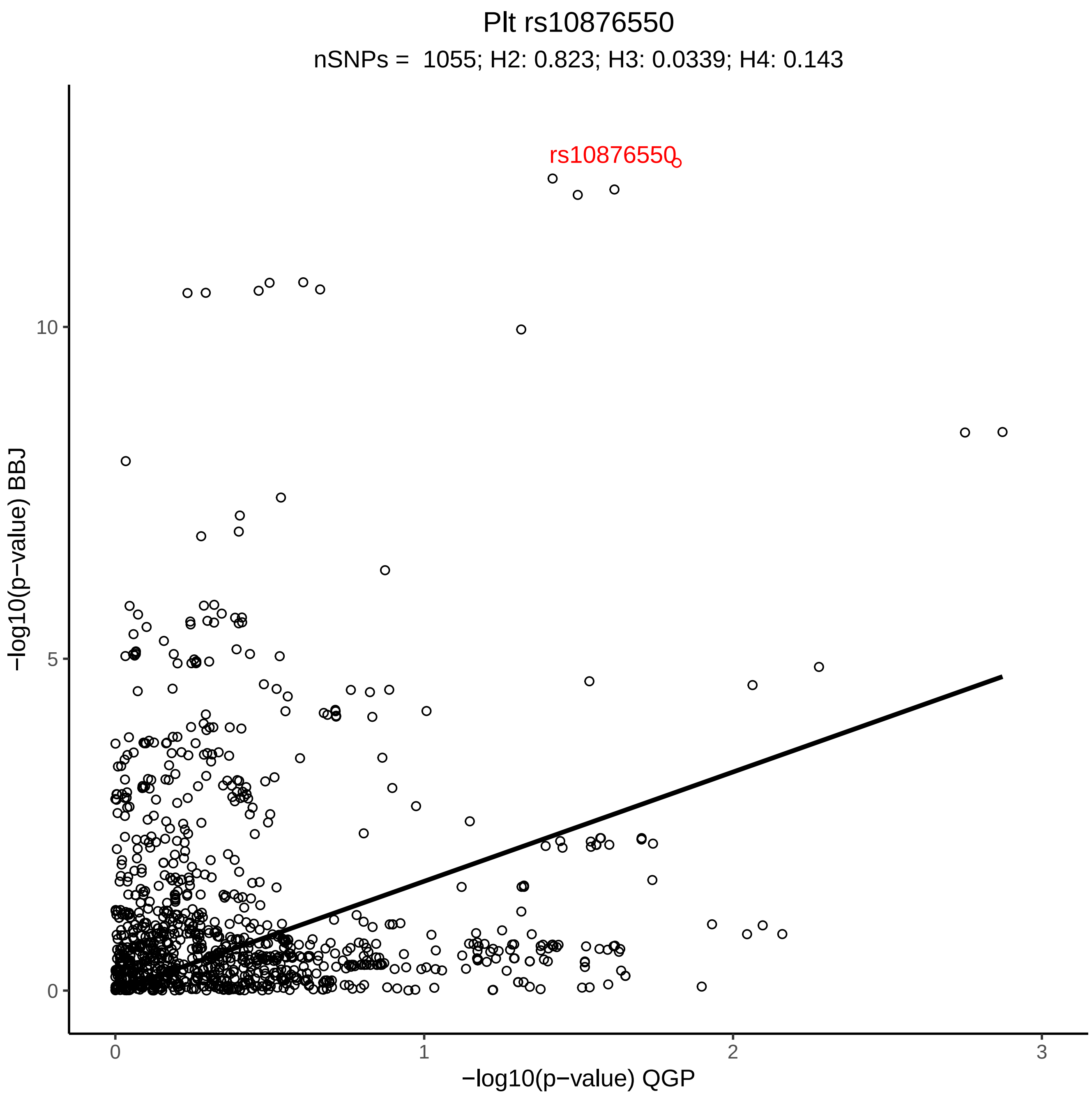

Plt rs10876550
nSNPs = 1055; H2: 0.823; H3: 0.0339; H4: 0.143
rs10876550
−log10(p−value) BBJ
−log10(p−value) QGP
0
5
10
0
1
2
3

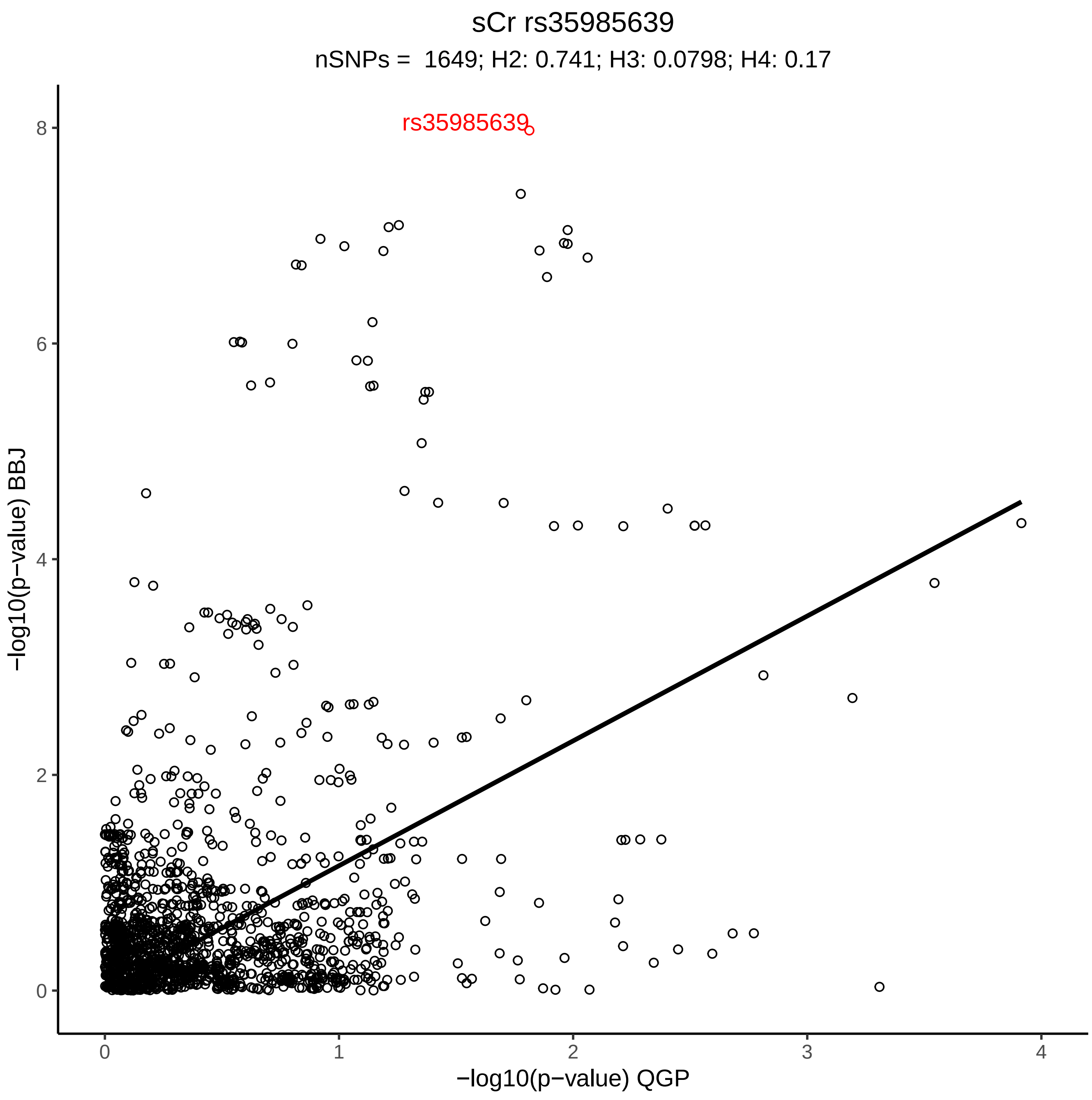
sCr rs35985639
nSNPs = 1649; H2: 0.741; H3: 0.0798; H4: 0.17
rs35985639
−log10(p−value) BBJ
−log10(p−value) QGP
0
2
4
6
8
0
1
2
3
4

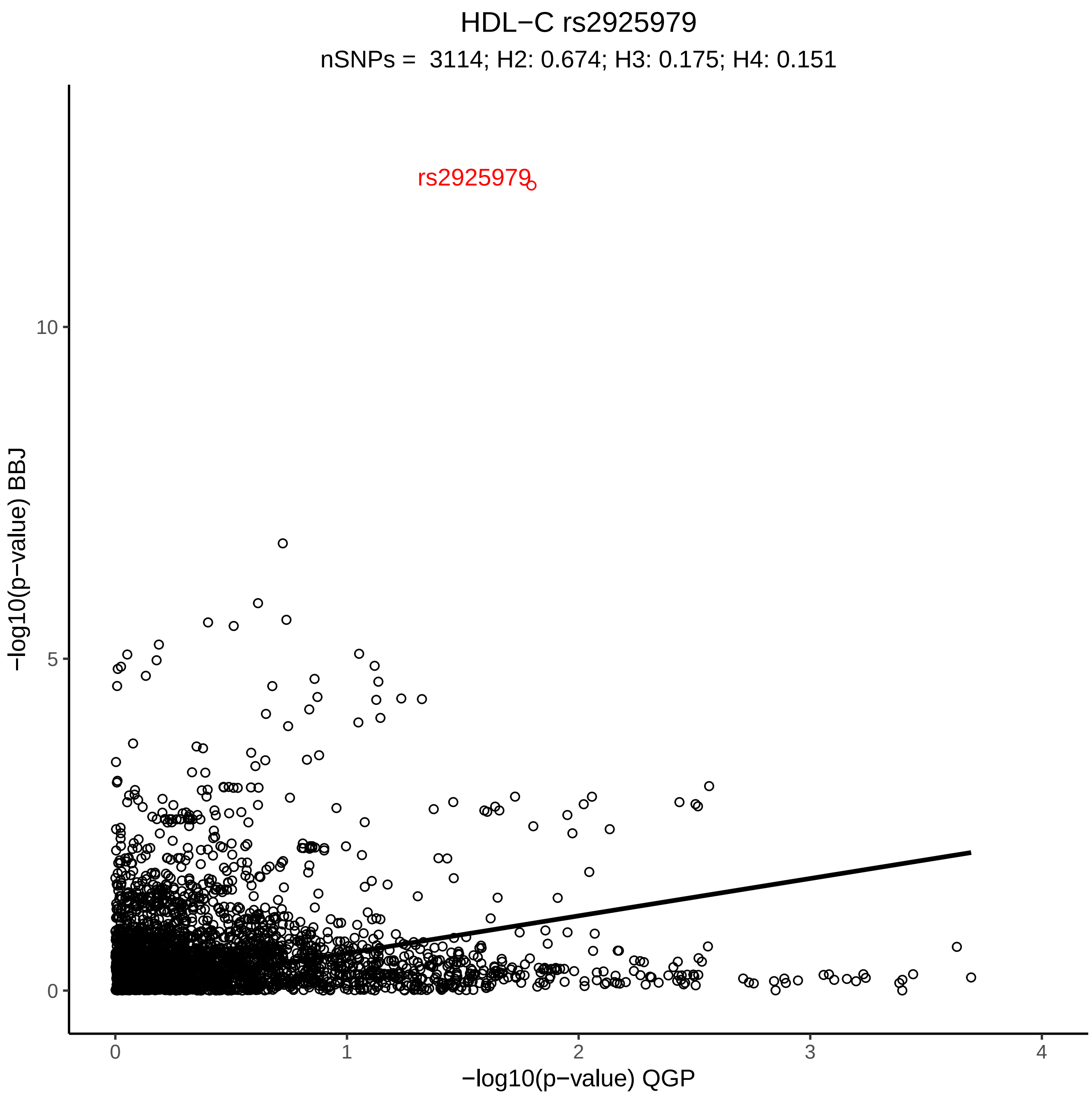

HDL−C rs2925979
nSNPs = 3114; H2: 0.674; H3: 0.175; H4: 0.151
rs2925979
10
5
0
−log10(p−value) BBJ
0
1
2
3
4
−log10(p−value) QGP

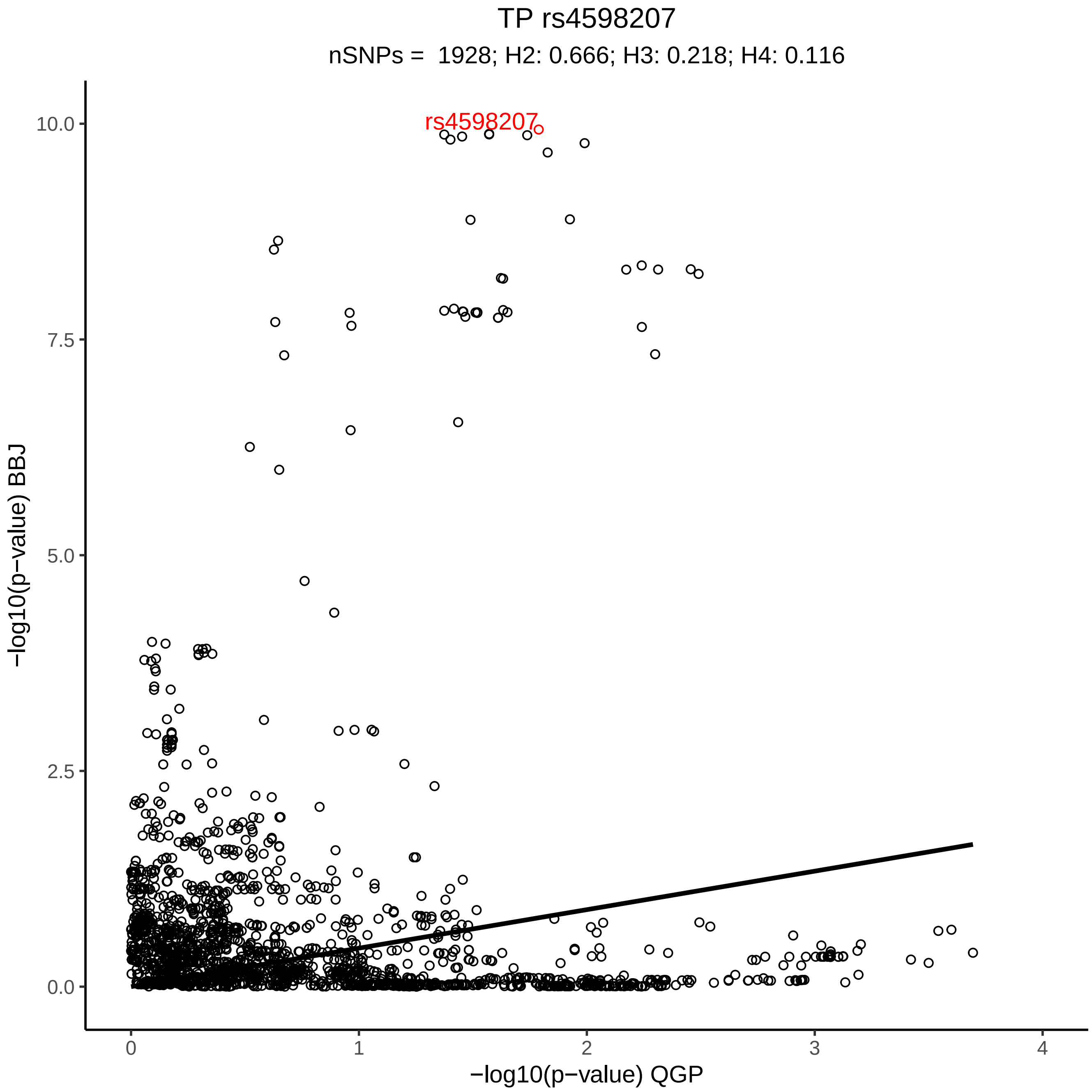
TP rs4598207
nSNPs = 1928; H2: 0.666; H3: 0.218; H4: 0.116
rs4598207
−log10(p−value) BBJ
10.0
7.5
5.0
2.5
0.0
0
1
2
3
4
−log10(p−value) QGP

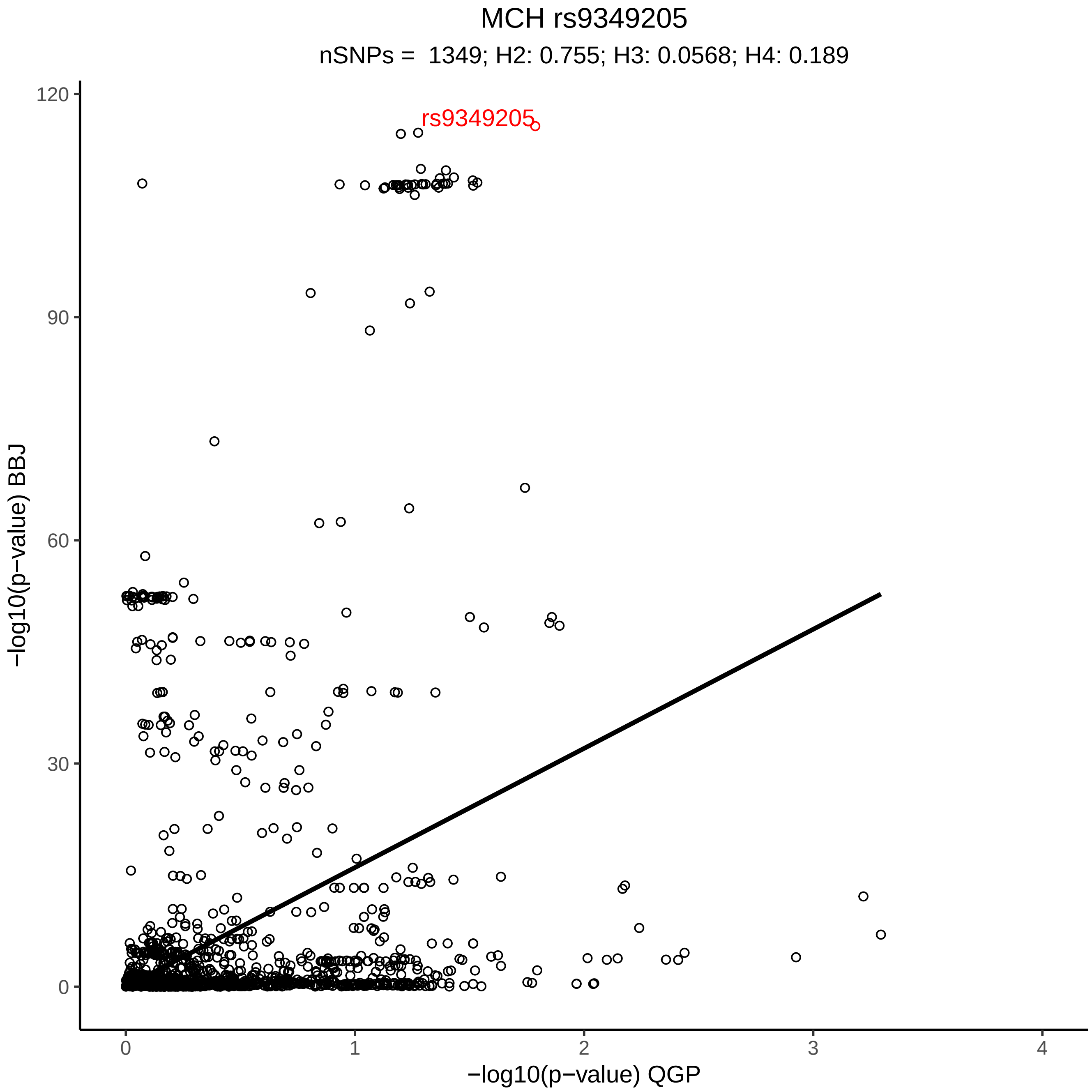

MCH rs9349205
nSNPs = 1349; H2: 0.755; H3: 0.0568; H4: 0.189
rs9349205
−log10(p−value) BBJ
−log10(p−value) QGP
120
90
60
30
0
0
1
2
3
4

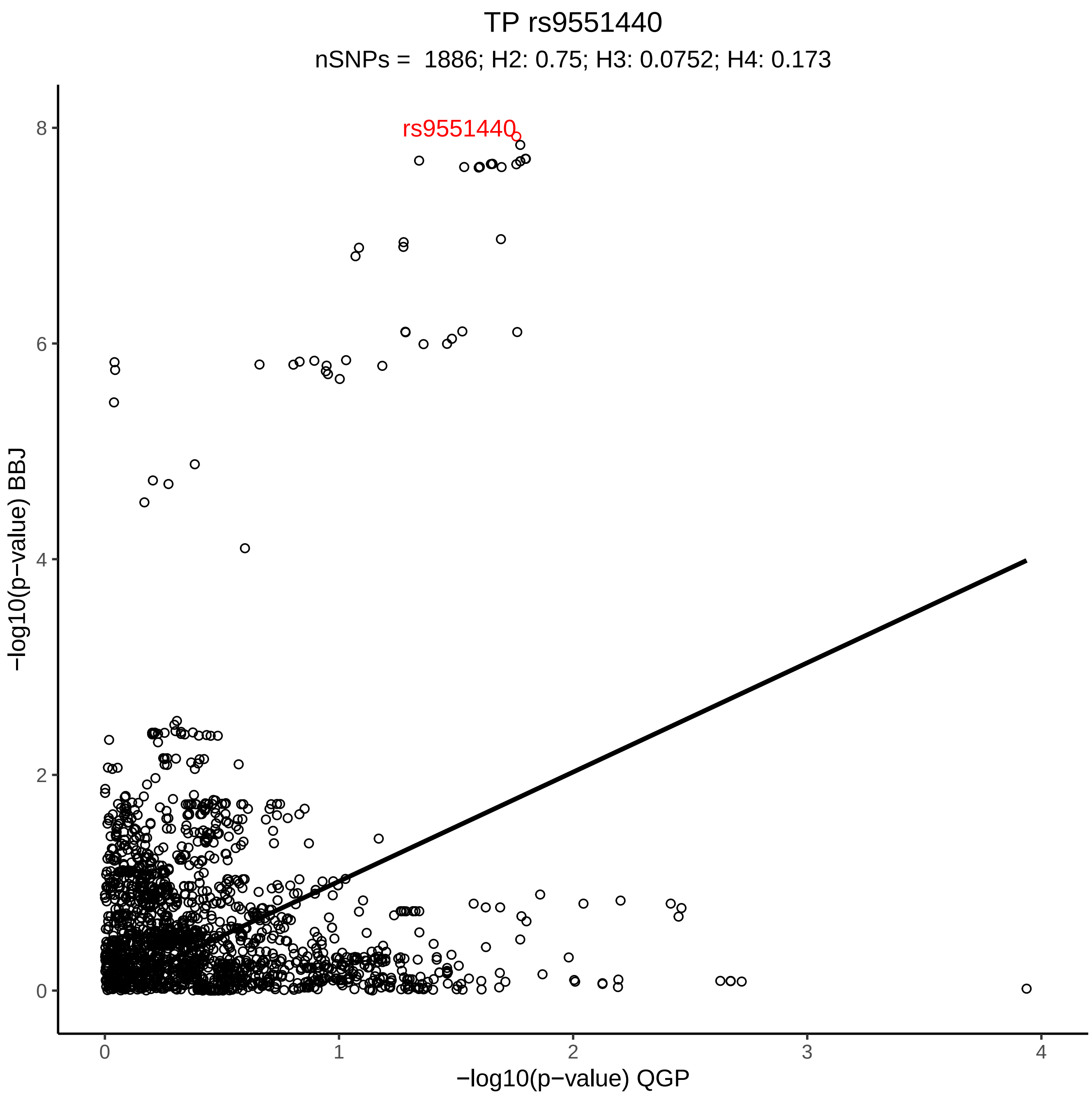
TP rs9551440
nSNPs = 1886; H2: 0.75; H3: 0.0752; H4: 0.173
rs9551440
−log10(p−value) BBJ
−log10(p−value) QGP
0
2
4
6
8
0
1
2
3
4

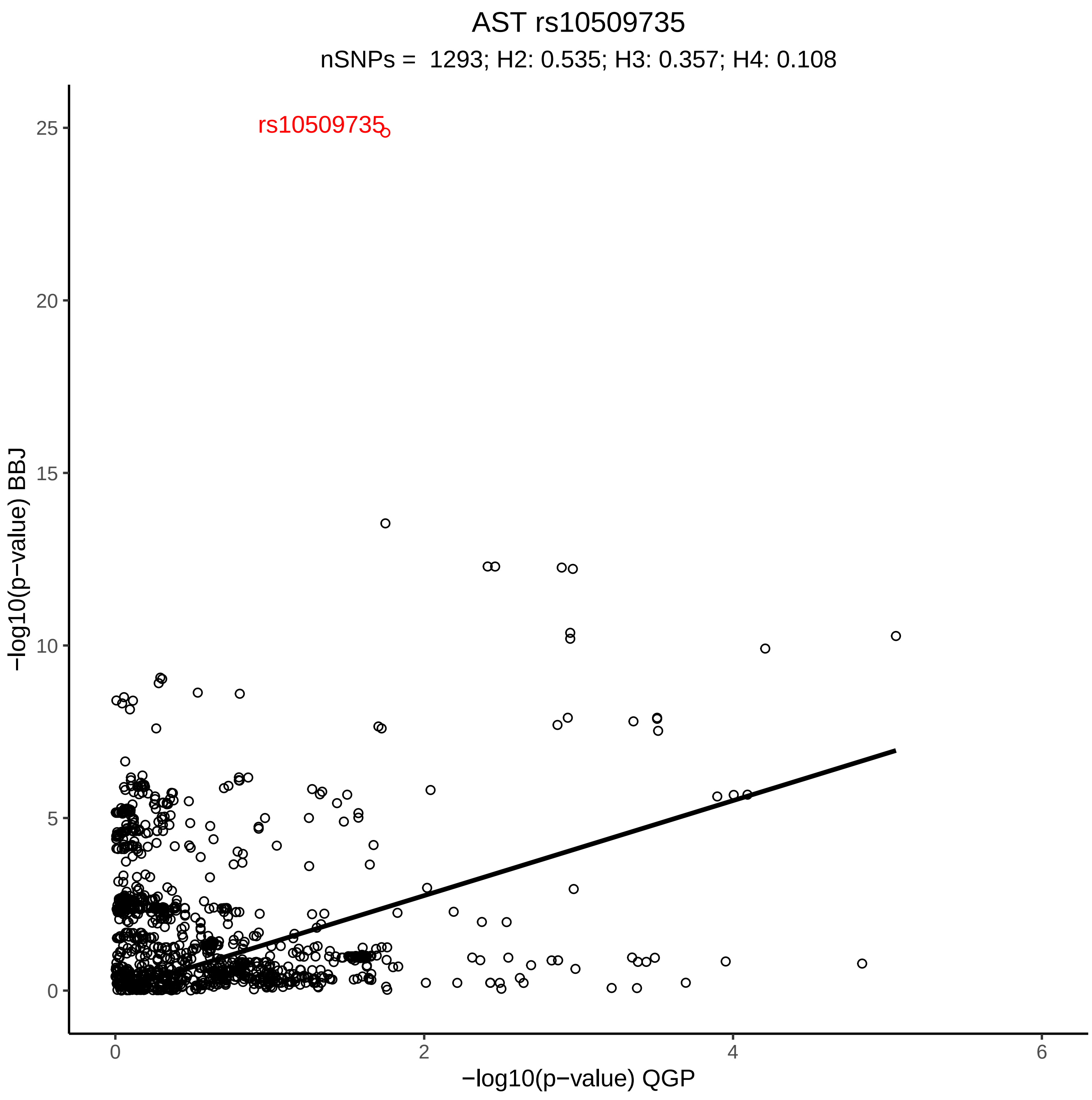

AST rs10509735
nSNPs = 1293; H2: 0.535; H3: 0.357; H4: 0.108
rs10509735
−log10(p−value) BBJ
−log10(p−value) QGP
0
5
10
15
20
25
0
2
4
6

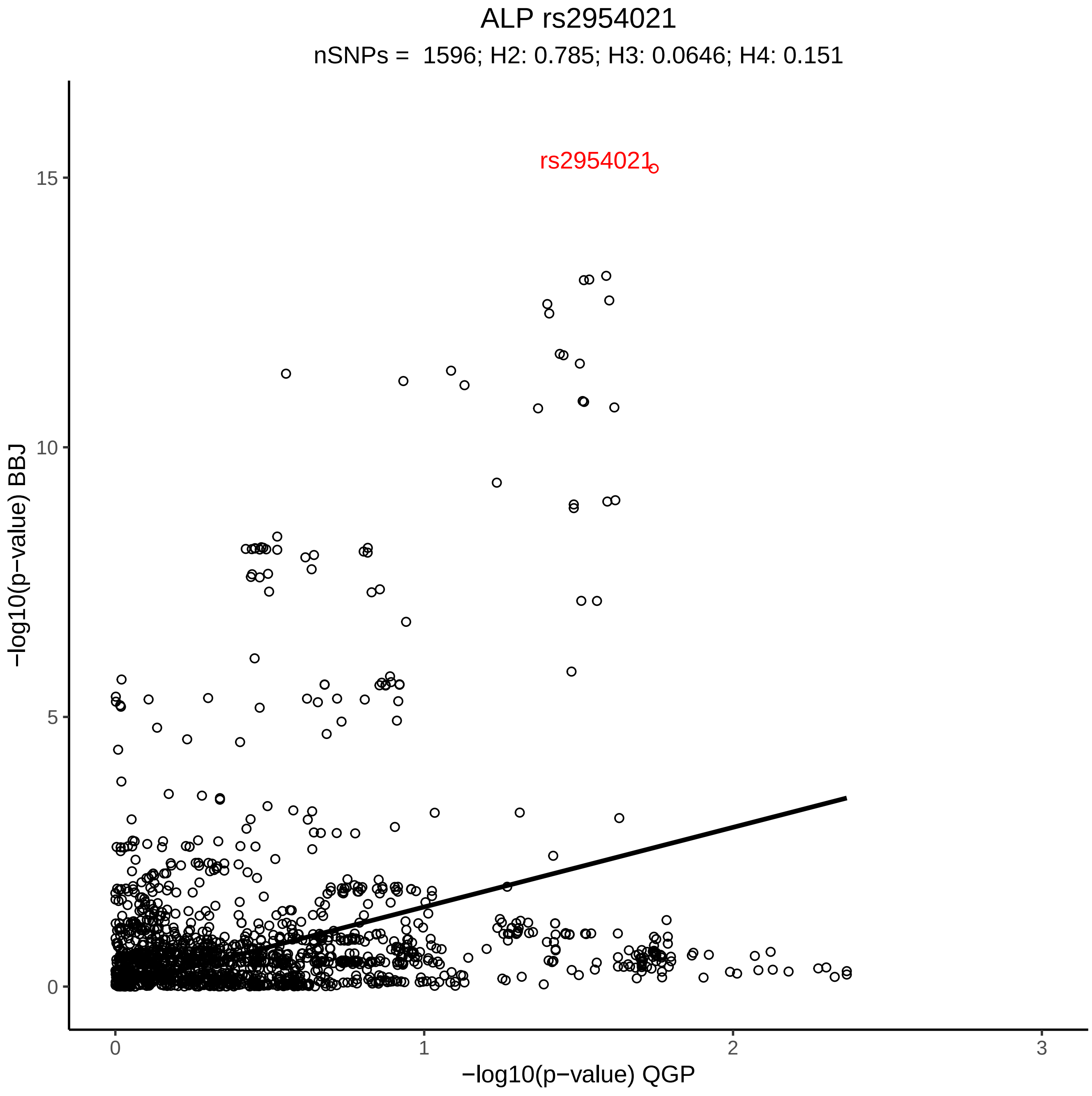

ALP rs2954021
nSNPs = 1596; H2: 0.785; H3: 0.0646; H4: 0.151
rs2954021
−log10(p−value) BBJ
−log10(p−value) QGP
0
5
10
15
0
1
2
3

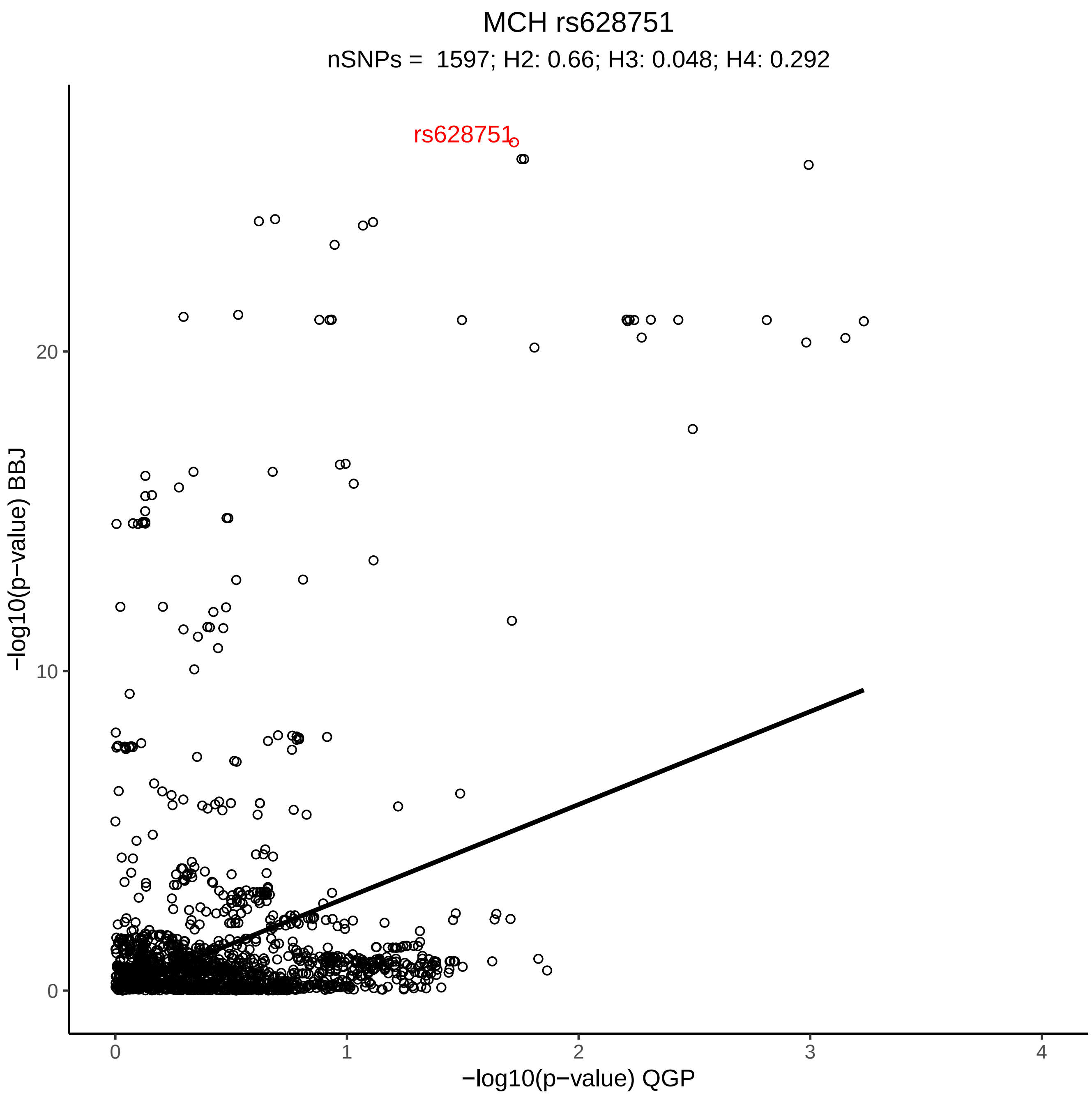

MCH rs628751
nSNPs = 1597; H2: 0.66; H3: 0.048; H4: 0.292
rs628751
−log10(p−value) BBJ
−log10(p−value) QGP
0
10
20
0
1
2
3
4

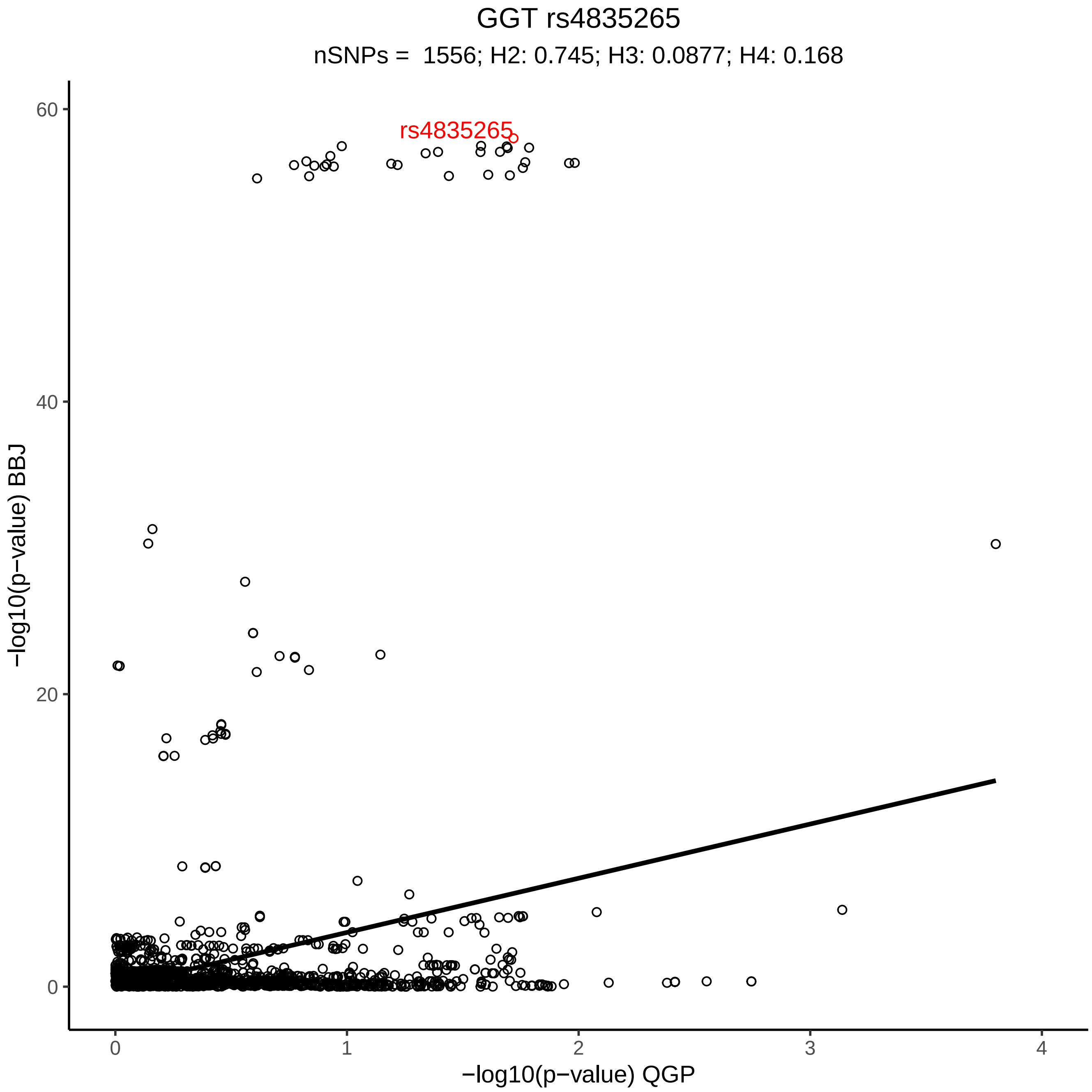

GGT rs4835265
nSNPs = 1556; H2: 0.745; H3: 0.0877; H4: 0.168
rs4835265
60
40
20
0
−log10(p−value) BBJ
0
1
2
3
4
−log10(p−value) QGP

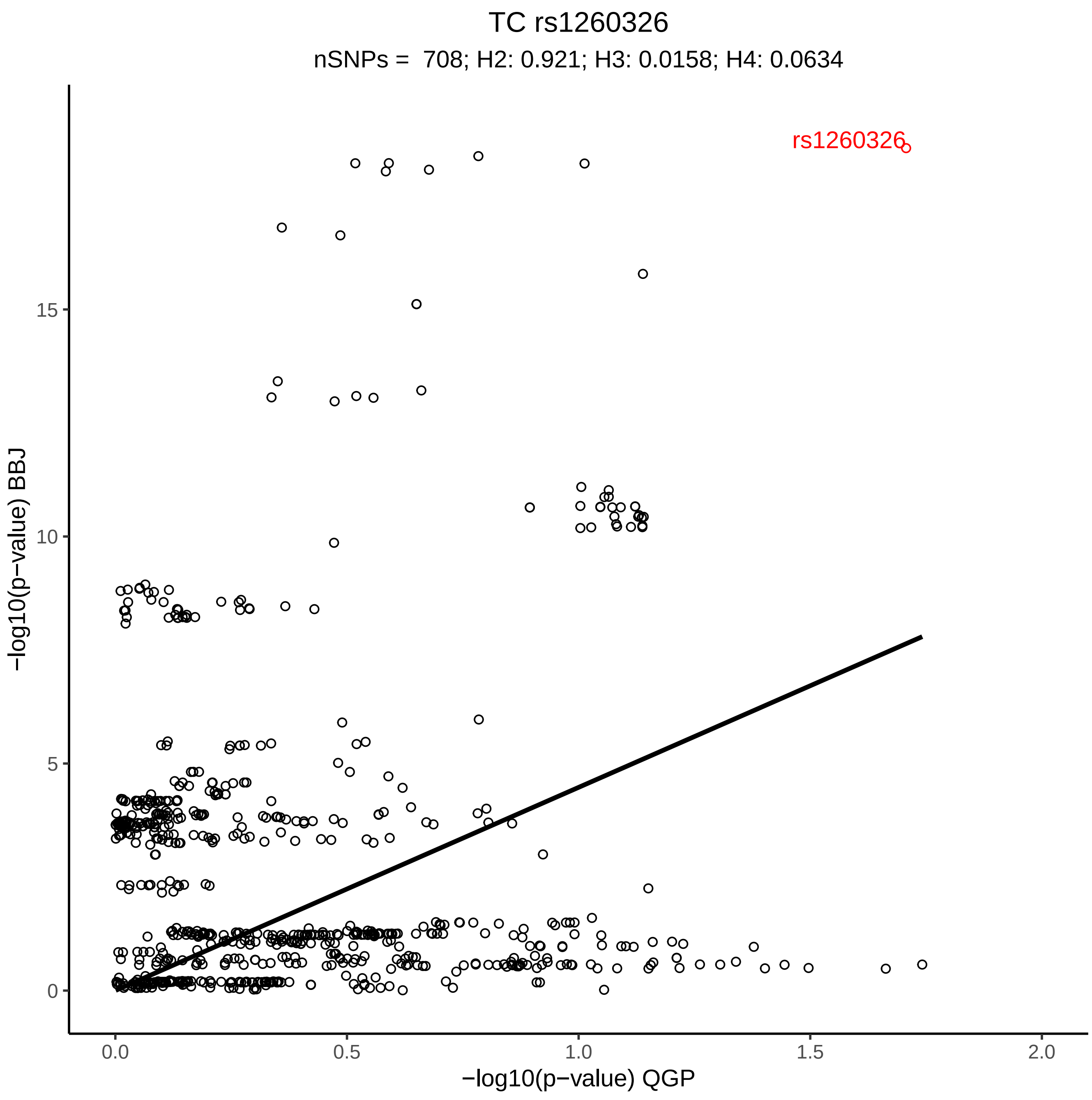
TC rs1260326
nSNPs = 708; H2: 0.921; H3: 0.0158; H4: 0.0634
rs1260326
−log10(p−value) BBJ
0
5
10
15
−log10(p−value) QGP
0.0
0.5
1.0
1.5
2.0

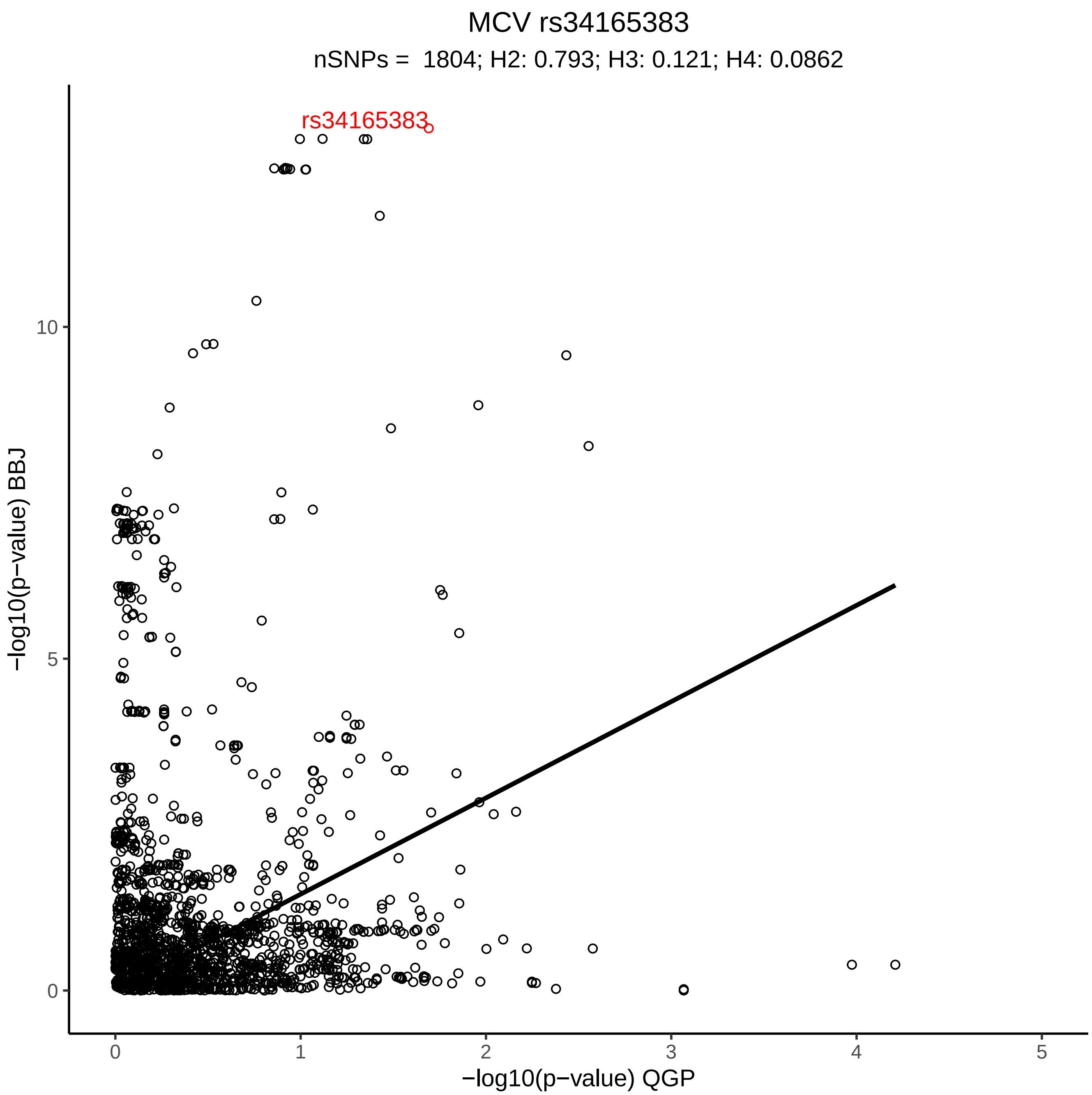

MCV rs34165383
nSNPs = 1804; H2: 0.793; H3: 0.121; H4: 0.0862
rs34165383
−log10(p−value) BBJ
−log10(p−value) QGP
0
5
10
0
1
2
3
4
5

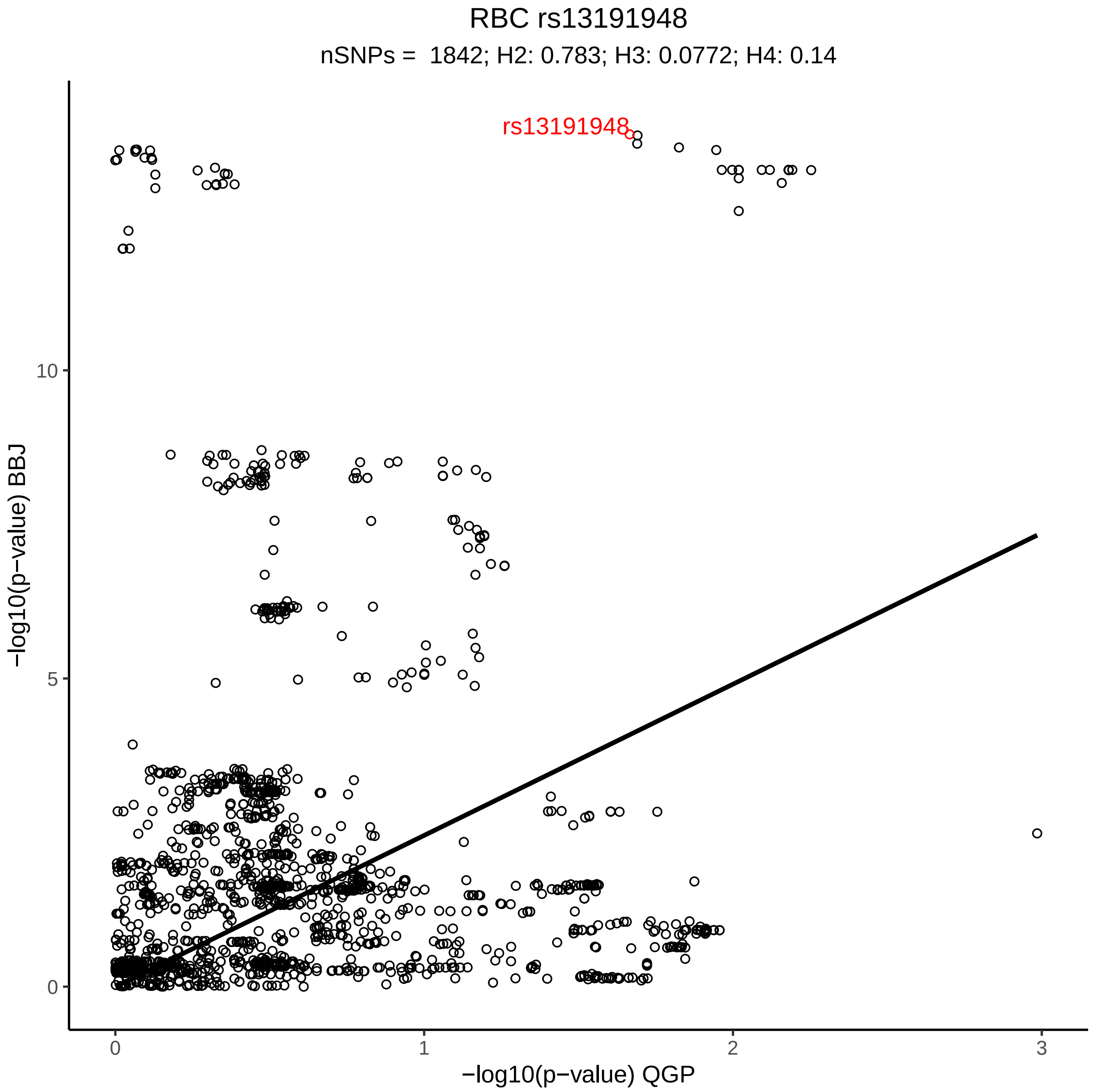
RBC rs13191948
nSNPs = 1842; H2: 0.783; H3: 0.0772; H4: 0.14
rs13191948
10
5
0
−log10(p−value) BBJ
0
1
2
3
−log10(p−value) QGP

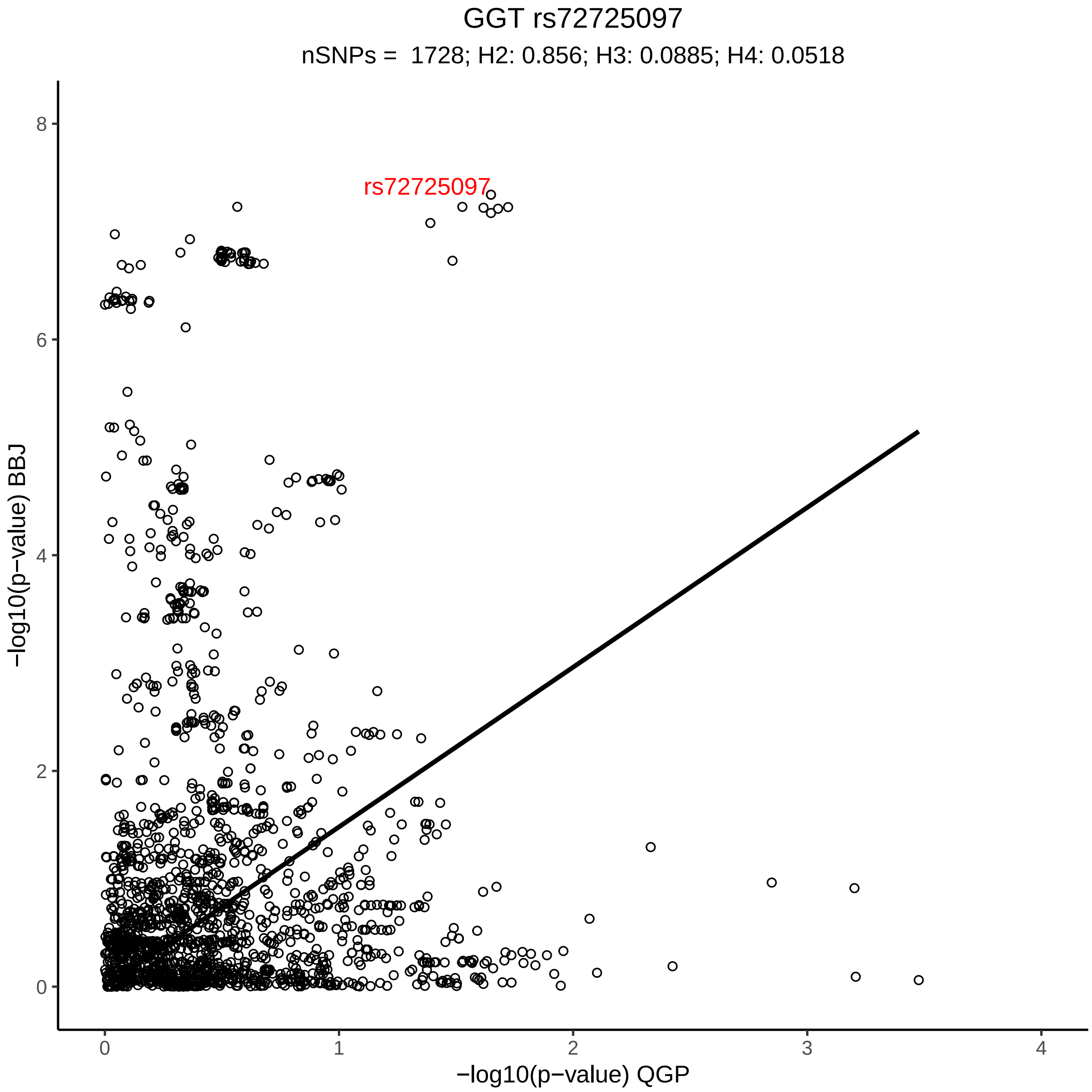
GGT rs72725097
nSNPs = 1728; H2: 0.856; H3: 0.0885; H4: 0.0518
rs72725097
−log10(p−value) BBJ
−log10(p−value) QGP
0
2
4
6
8
0
1
2
3
4

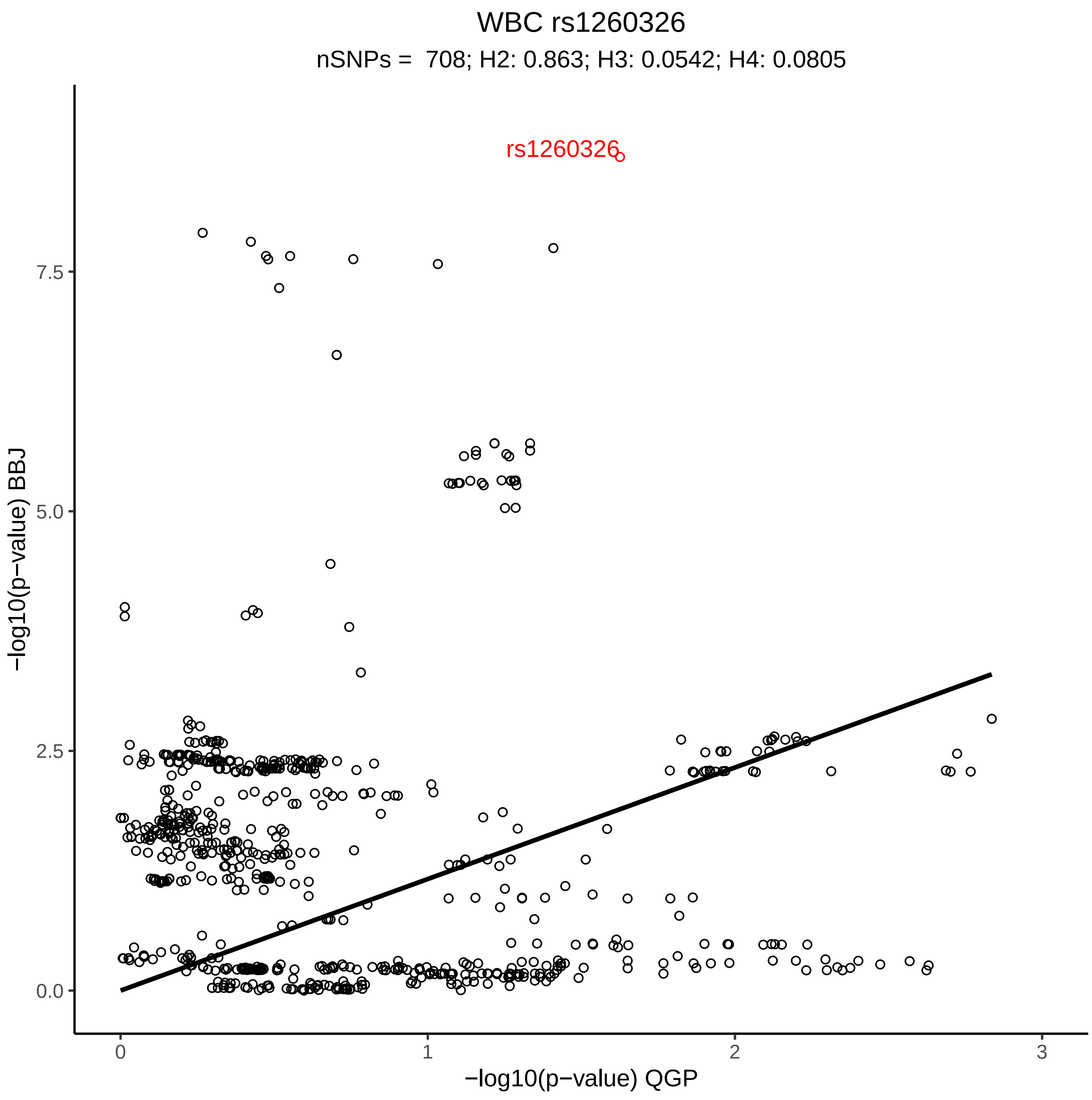

WBC rs1260326
nSNPs = 708; H2: 0.863; H3: 0.0542; H4: 0.0805
rs1260326
7.5
5.0
2.5
0.0
−log10(p−value) BBJ
0
1
2
3
−log10(p−value) QGP

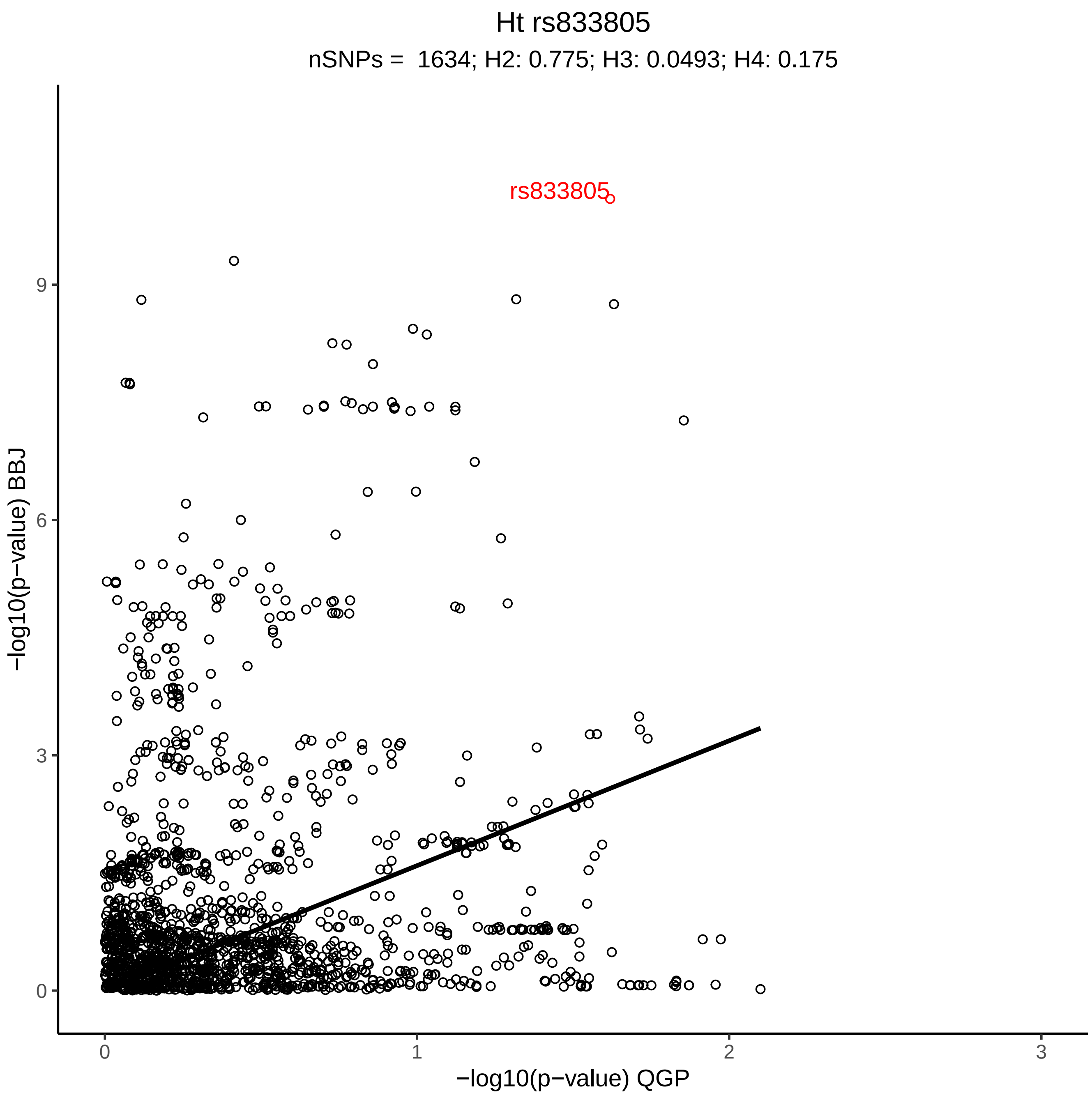
Ht rs833805
nSNPs = 1634; H2: 0.775; H3: 0.0493; H4: 0.175
rs833805
9
6
3
0
−log10(p−value) BBJ
0
1
2
3
−log10(p−value) QGP

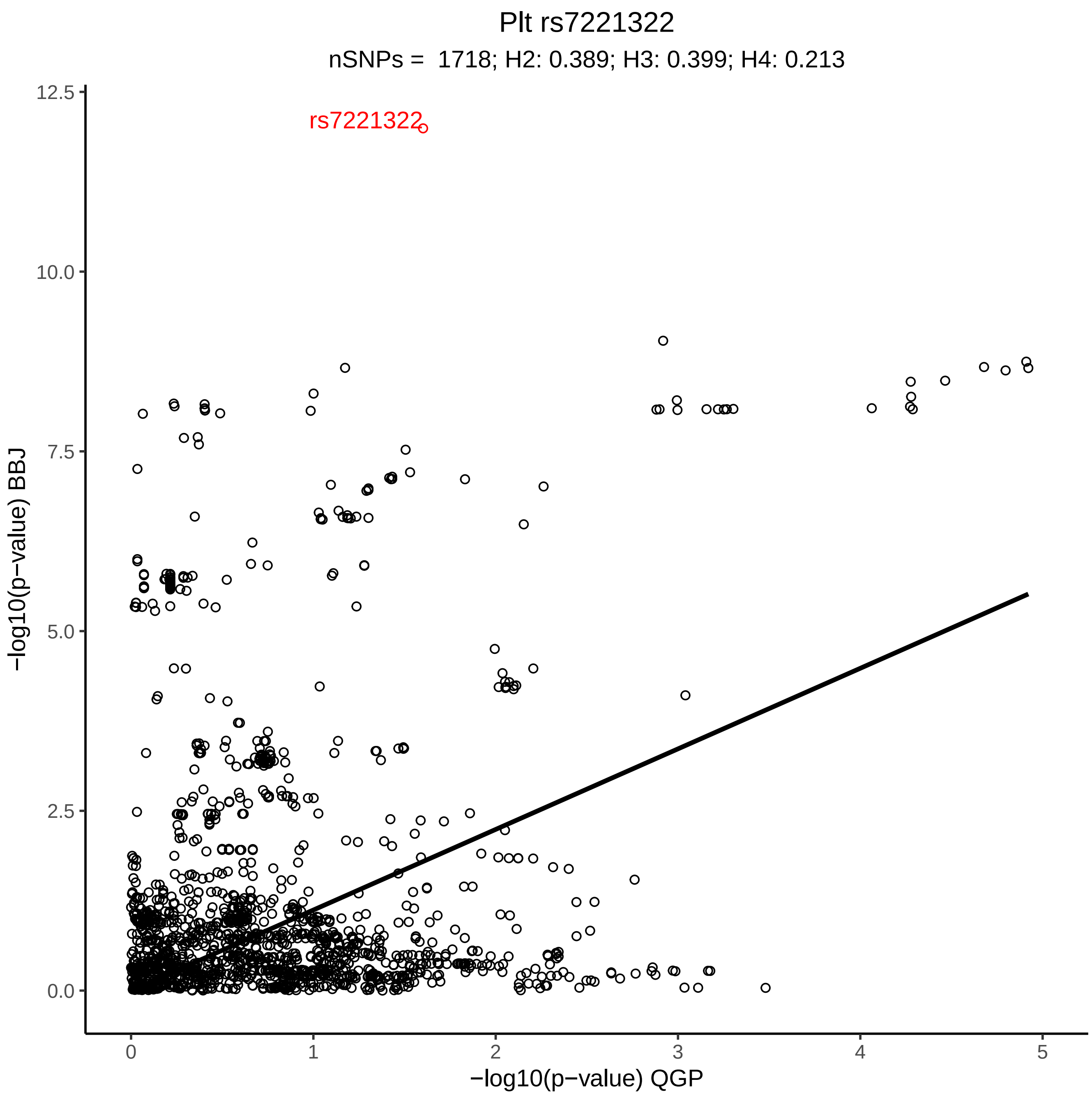
Plt rs7221322
nSNPs = 1718; H2: 0.389; H3: 0.399; H4: 0.213
rs7221322
−log10(p−value) BBJ
−log10(p−value) QGP
0.0
2.5
5.0
7.5
10.0
12.5
0
1
2
3
4
5

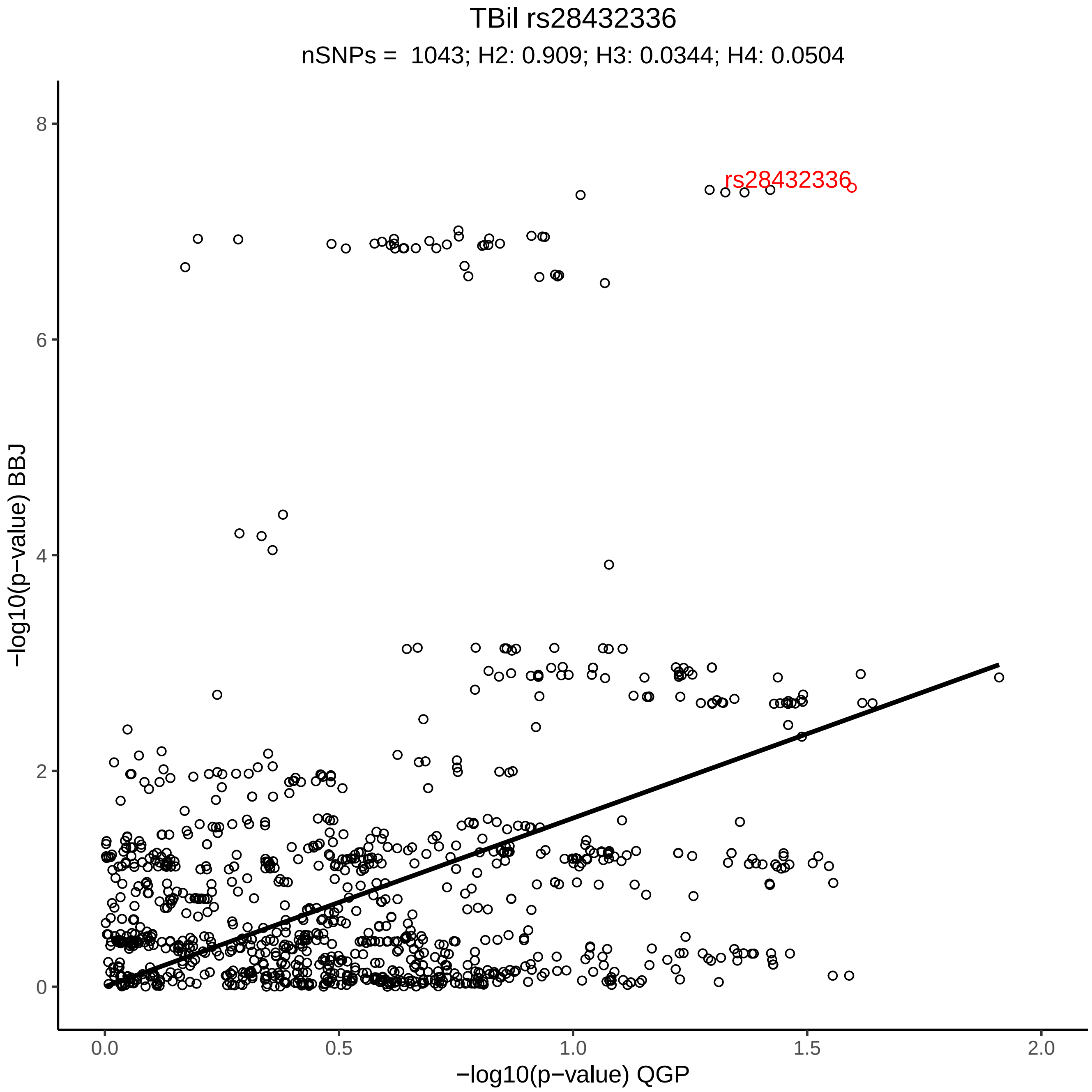
TBil rs28432336
nSNPs = 1043; H2: 0.909; H3: 0.0344; H4: 0.0504
rs28432336
−log10(p−value) BBJ
−log10(p−value) QGP
0
2
4
6
8
0.0
0.5
1.0
1.5
2.0

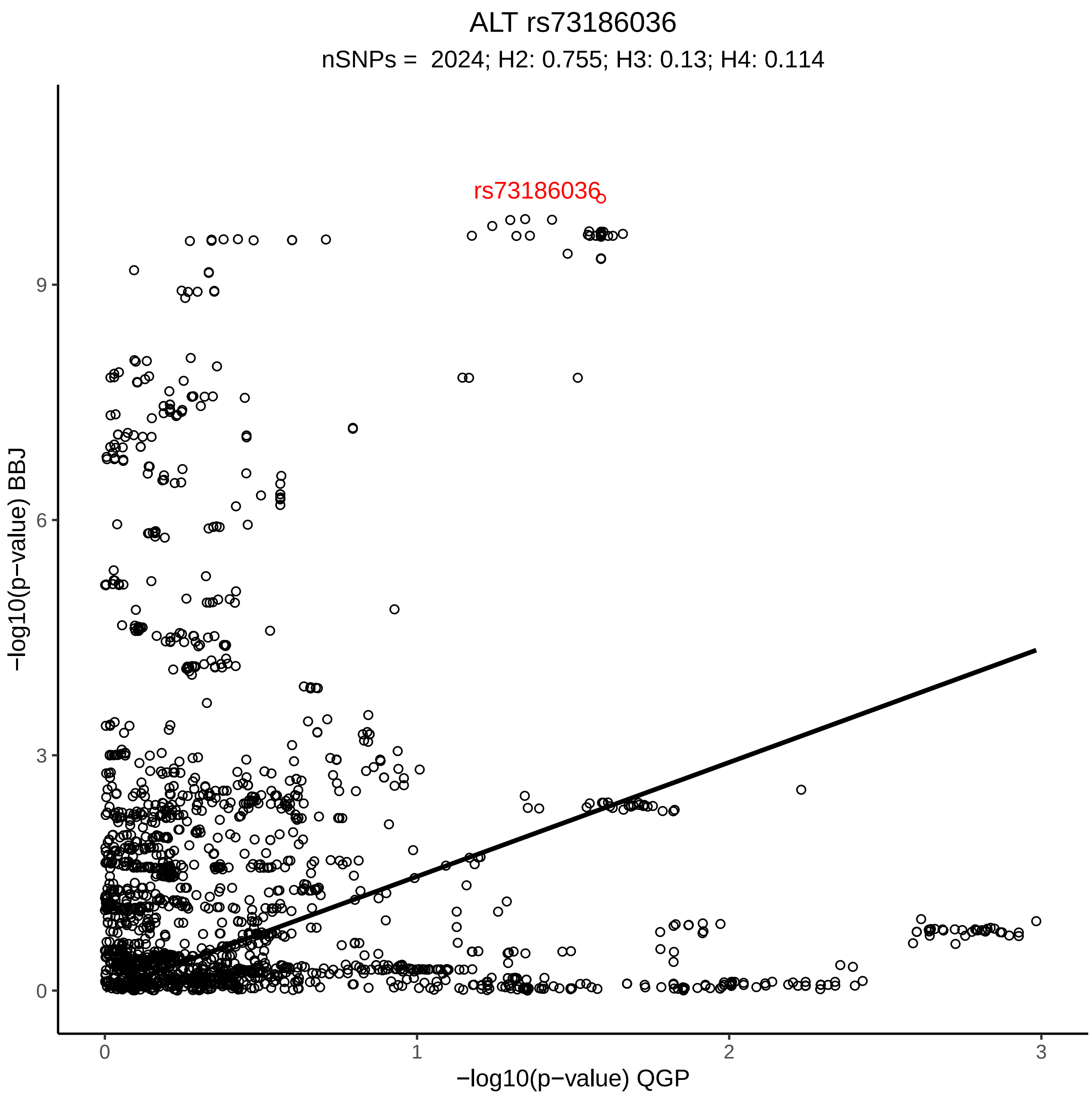
ALT rs73186036
nSNPs = 2024; H2: 0.755; H3: 0.13; H4: 0.114
rs73186036
−log10(p−value) BBJ
−log10(p−value) QGP
0
3
6
9
0
1
2
3

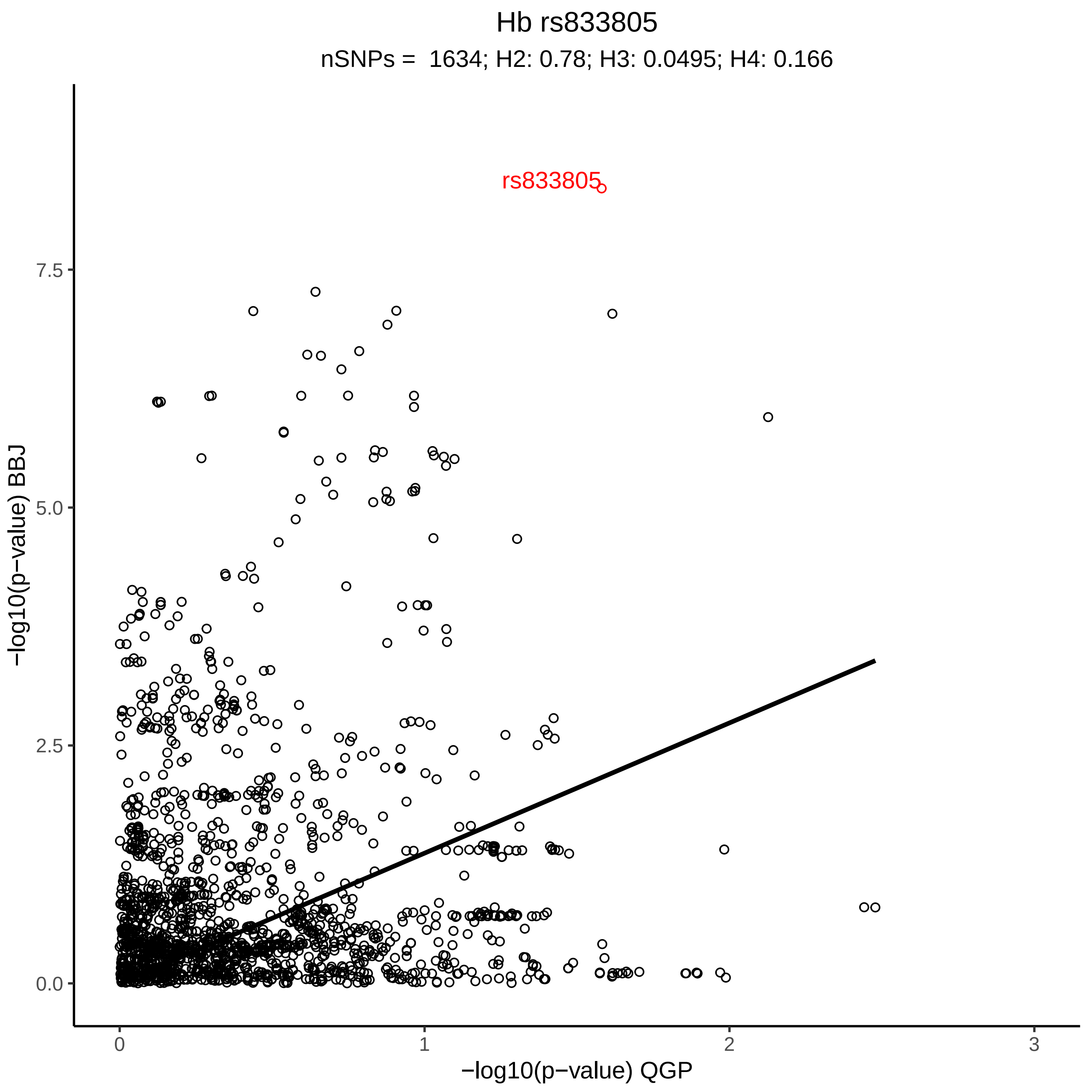

Hb rs833805
nSNPs = 1634; H2: 0.78; H3: 0.0495; H4: 0.166
rs833805
7.5
5.0
2.5
0.0
−log10(p−value) BBJ
0
1
2
3
−log10(p−value) QGP

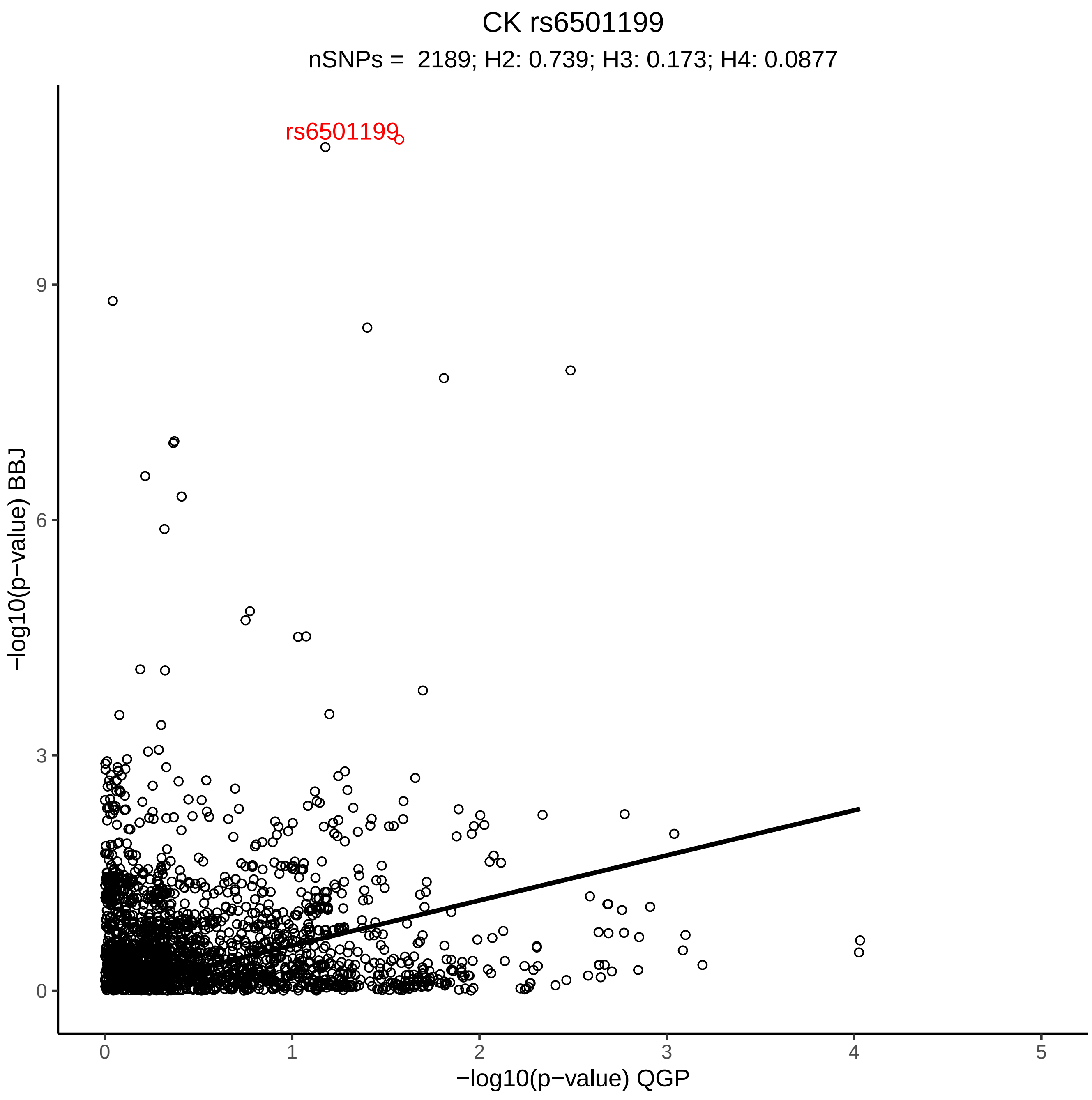

CK rs6501199
nSNPs = 2189; H2: 0.739; H3: 0.173; H4: 0.0877
rs6501199
−log10(p−value) BBJ
−log10(p−value) QGP
0
3
6
9
0
1
2
3
4
5

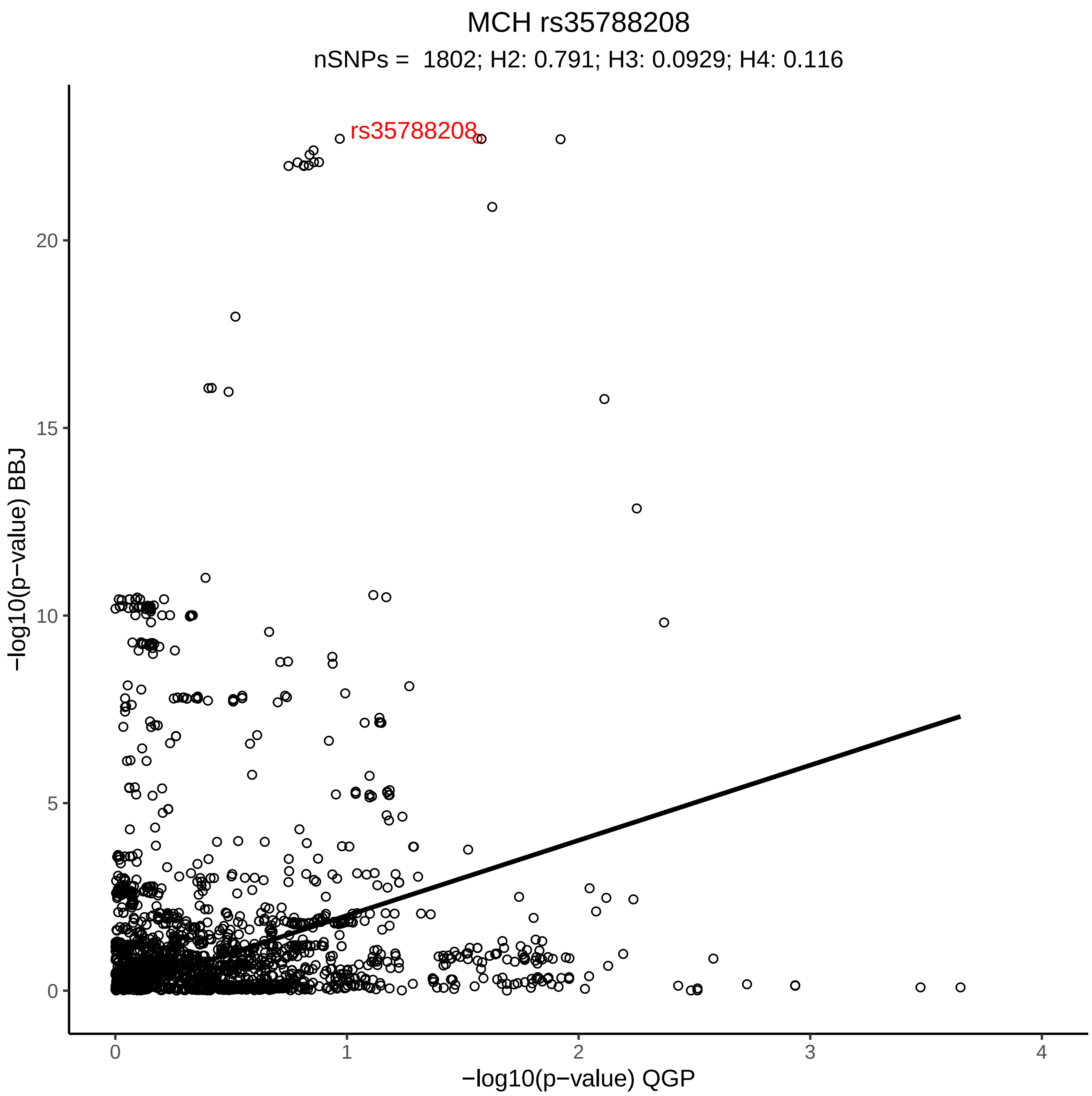

MCH rs35788208
nSNPs = 1802; H2: 0.791; H3: 0.0929; H4: 0.116
rs35788208
−log10(p−value) BBJ
−log10(p−value) QGP
0
5
10
15
20
0
1
2
3
4

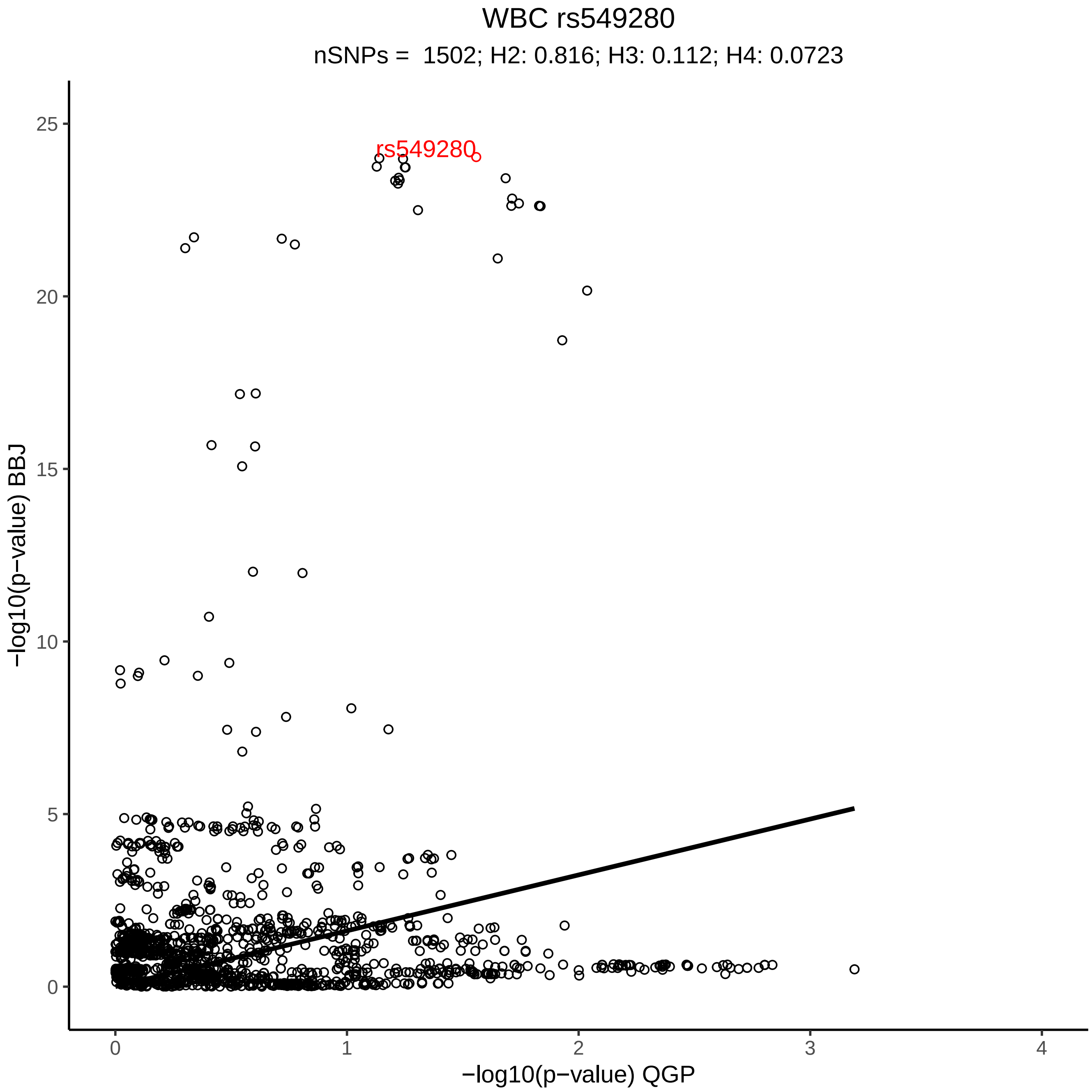

WBC rs549280
nSNPs = 1502; H2: 0.816; H3: 0.112; H4: 0.0723
rs549280
−log10(p−value) BBJ
0
5
10
15
20
25
0
1
2
3
4
−log10(p−value) QGP

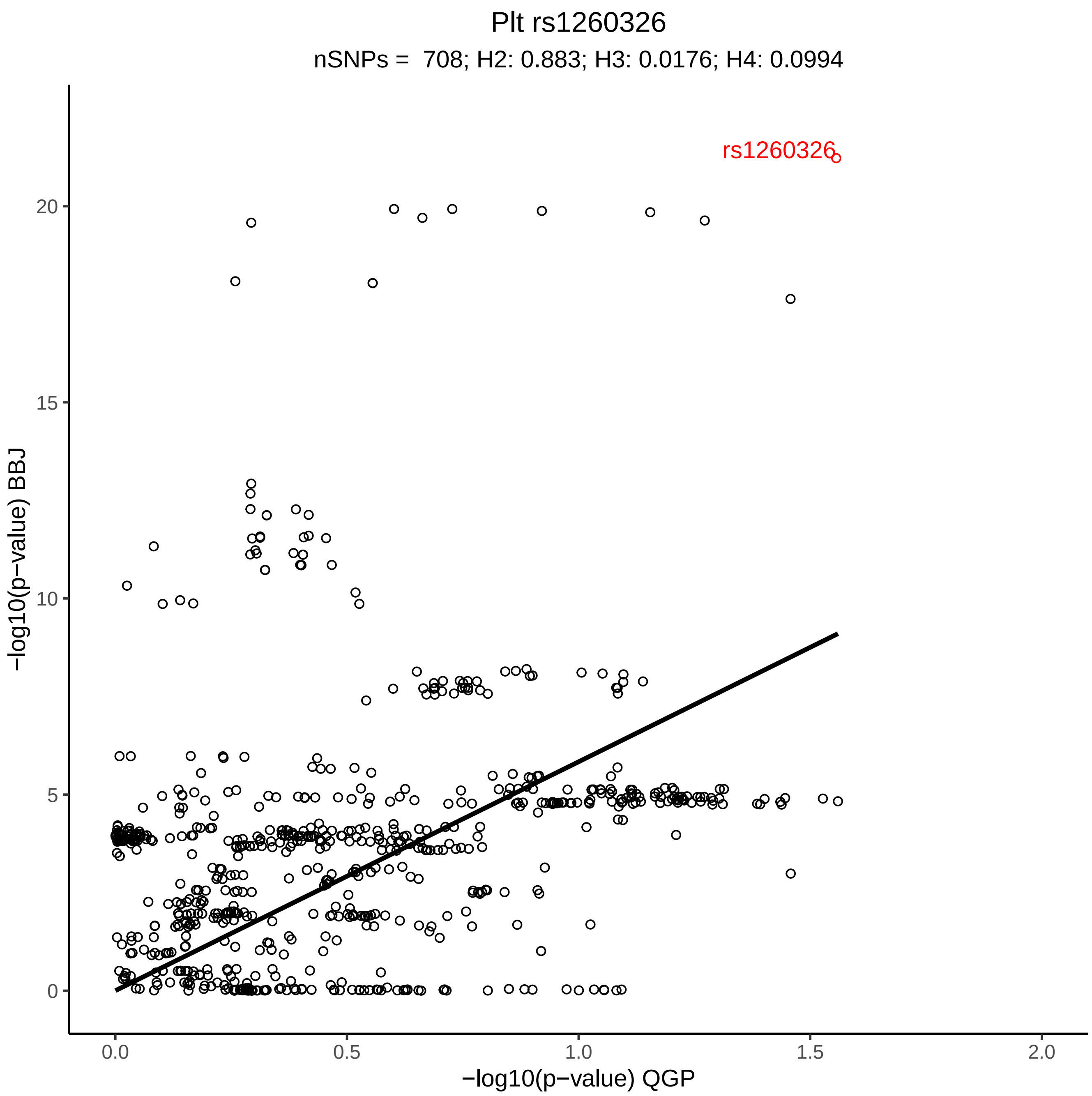
Plt rs1260326
nSNPs = 708; H2: 0.883; H3: 0.0176; H4: 0.0994
rs1260326
−log10(p−value) BBJ
−log10(p−value) QGP
0
5
10
15
20
0.0
0.5
1.0
1.5
2.0

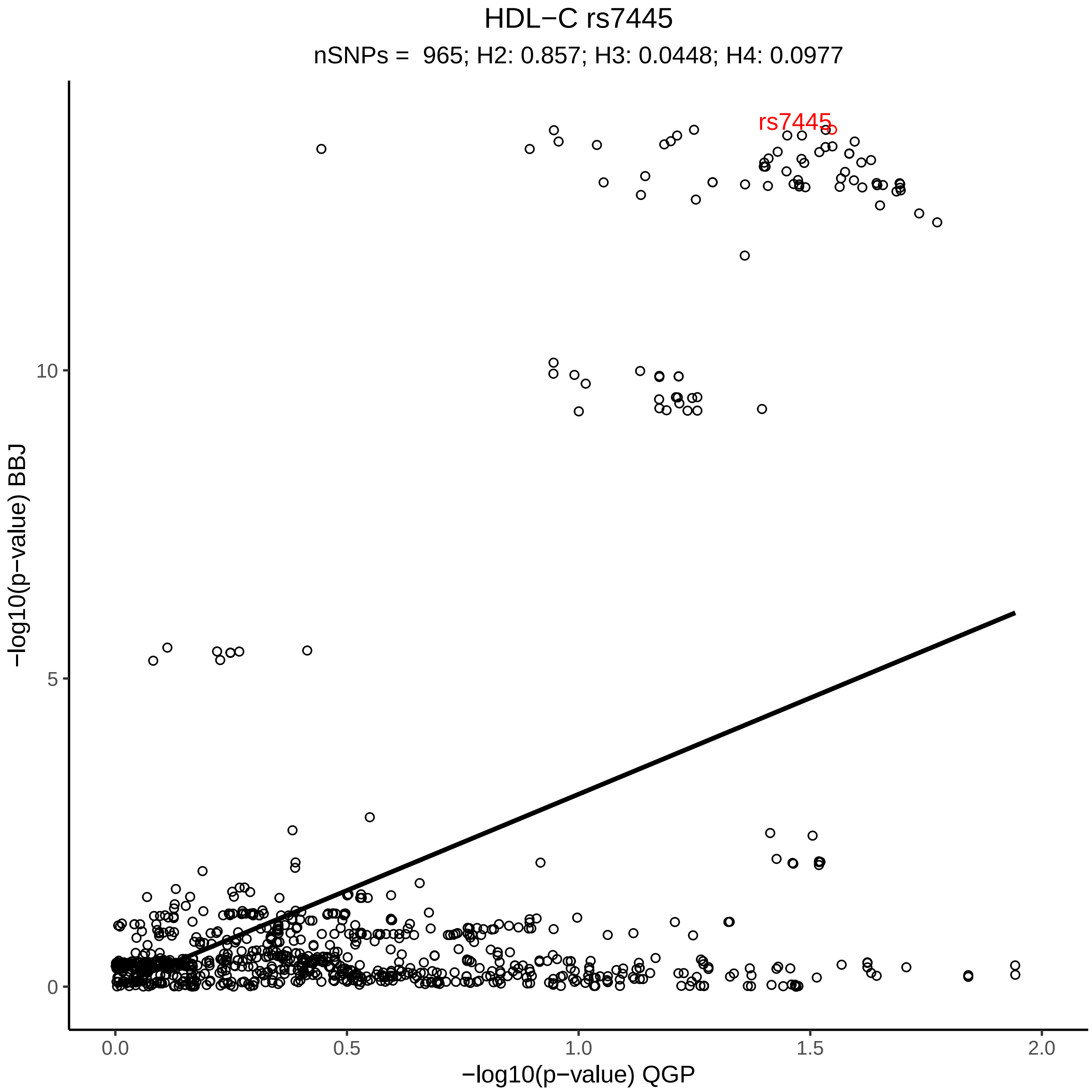
HDL−C rs7445
nSNPs = 965; H2: 0.857; H3: 0.0448; H4: 0.0977
rs7445
−log10(p−value) BBJ
−log10(p−value) QGP
0
5
10
0.0
0.5
1.0
1.5
2.0

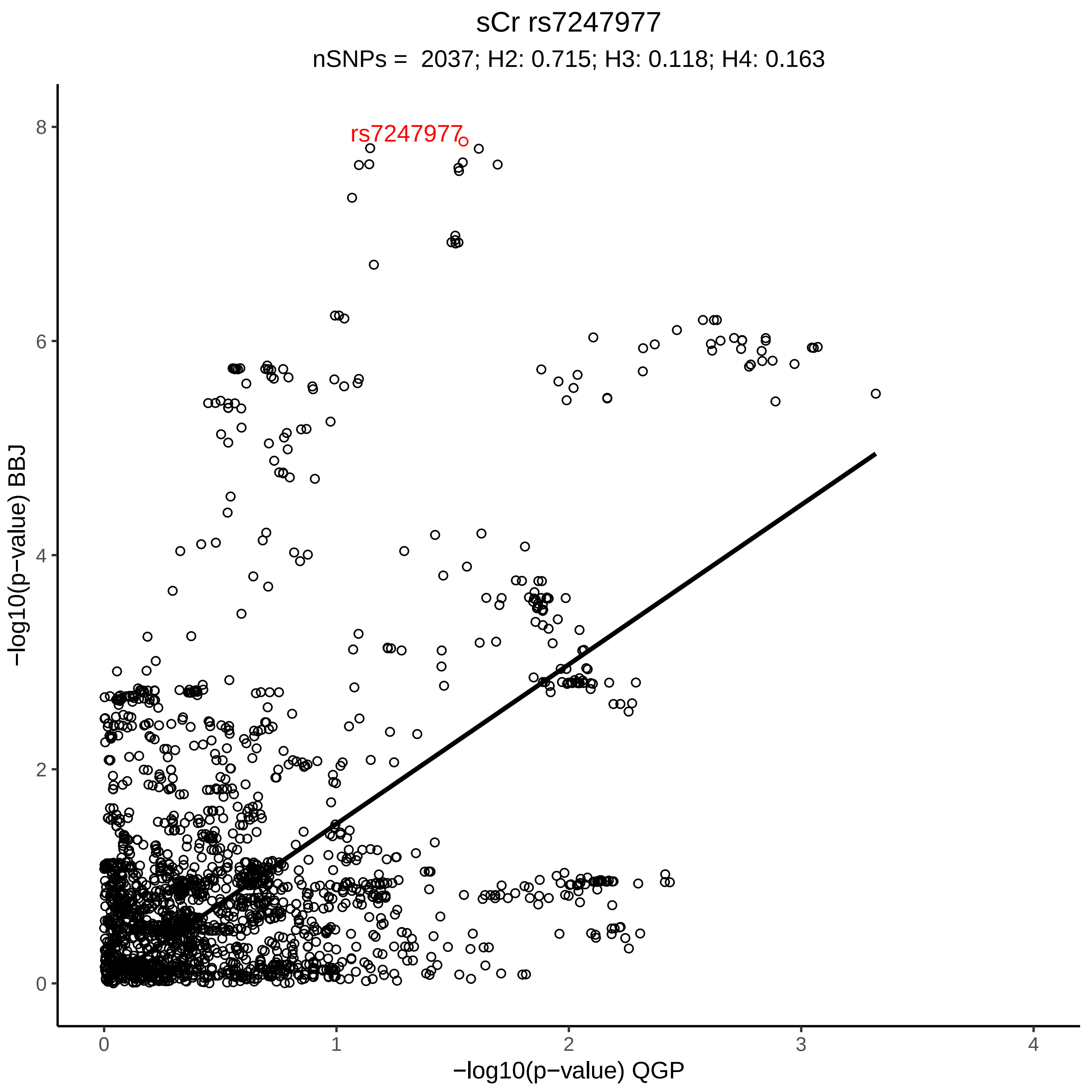
sCr rs7247977
nSNPs = 2037; H2: 0.715; H3: 0.118; H4: 0.163
rs7247977
−log10(p−value) BBJ
−log10(p−value) QGP
0
2
4
6
8
0
1
2
3
4

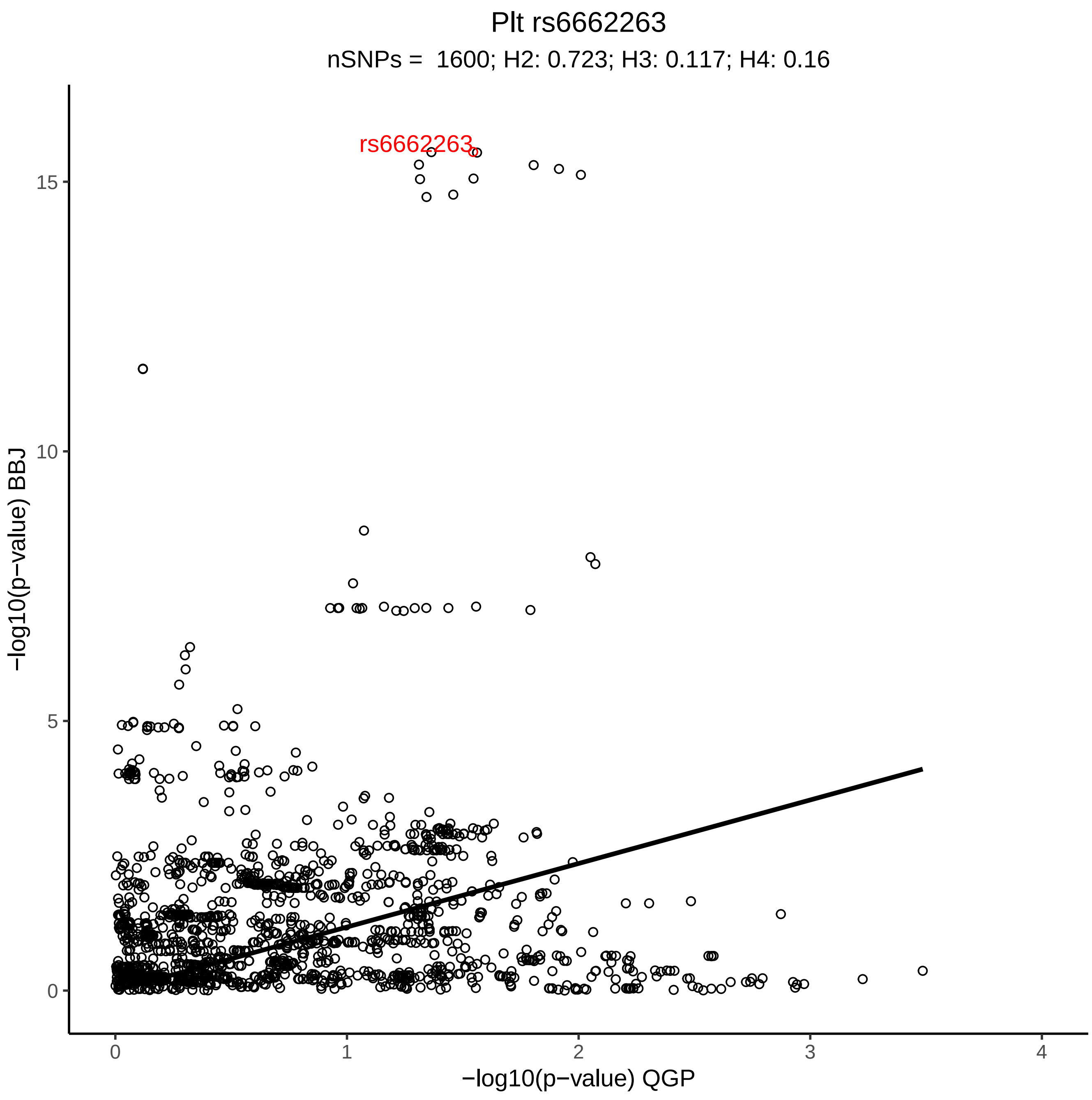
Plt rs6662263
nSNPs = 1600; H2: 0.723; H3: 0.117; H4: 0.16
rs6662263
−log10(p−value) BBJ
−log10(p−value) QGP
0
5
10
15
0
1
2
3
4

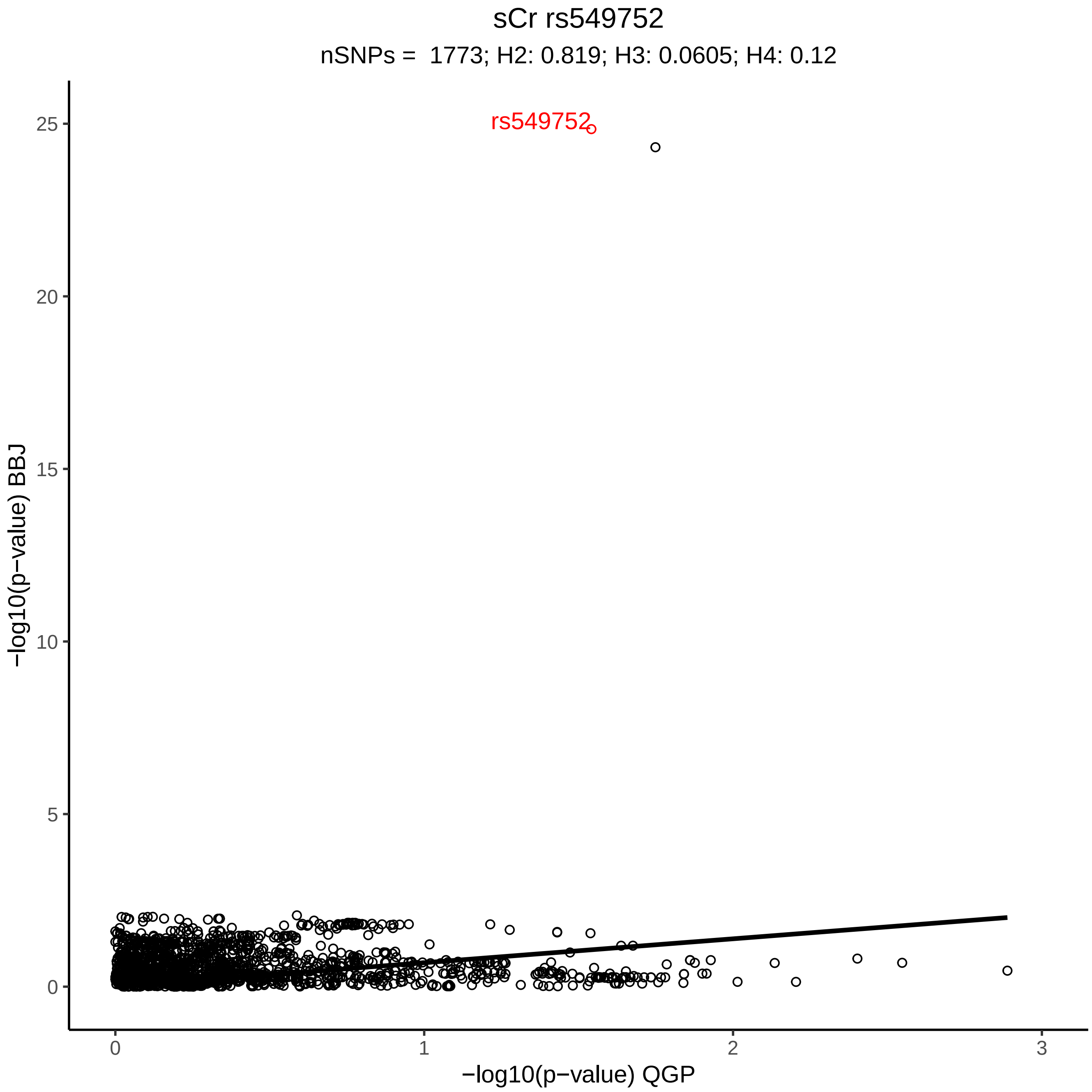
sCr rs549752
nSNPs = 1773; H2: 0.819; H3: 0.0605; H4: 0.12
rs549752
−log10(p−value) BBJ
−log10(p−value) QGP
0
5
10
15
20
25
0
1
2
3

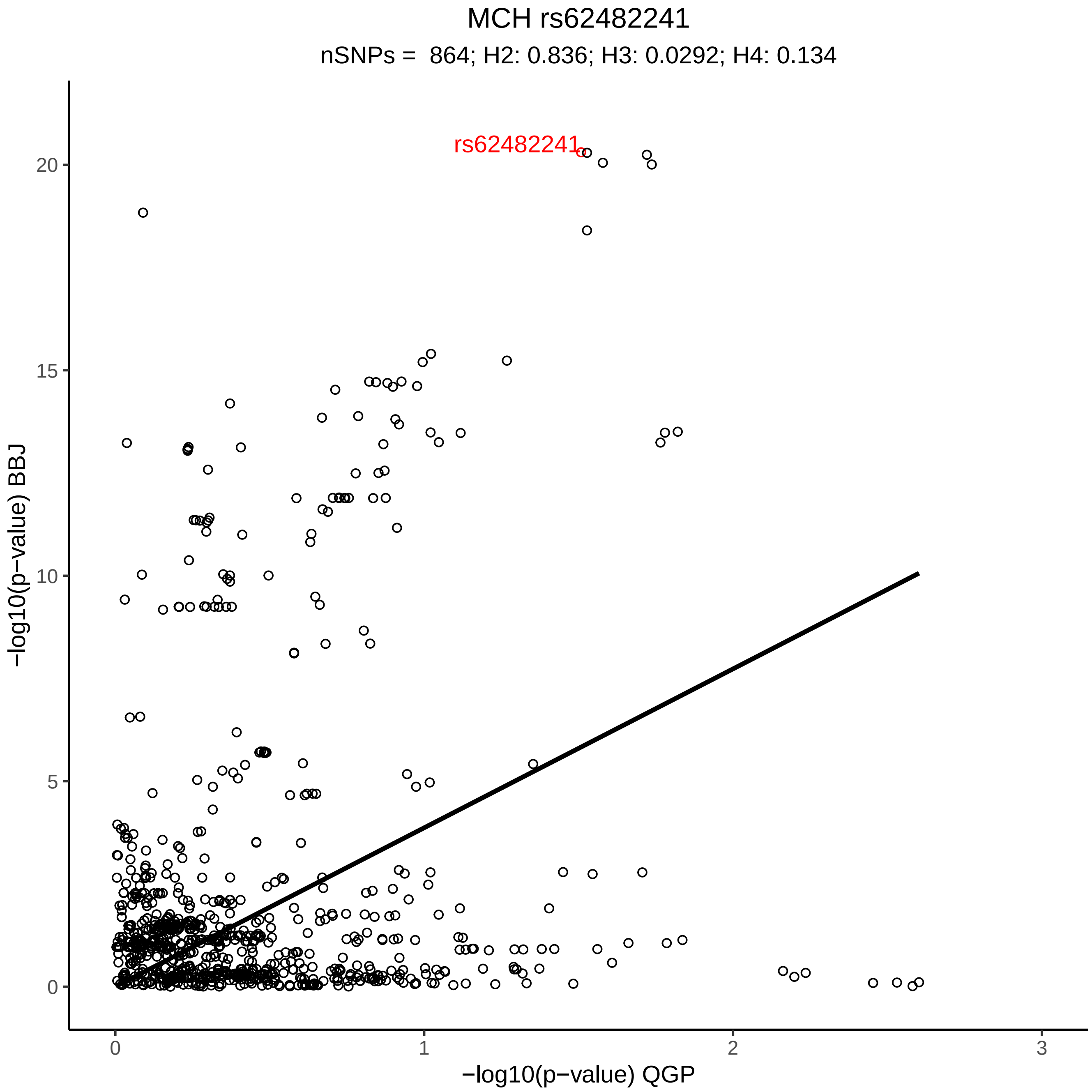

MCH rs62482241
nSNPs = 864; H2: 0.836; H3: 0.0292; H4: 0.134
rs62482241
−log10(p−value) BBJ
−log10(p−value) QGP
0
5
10
15
20
0
1
2
3

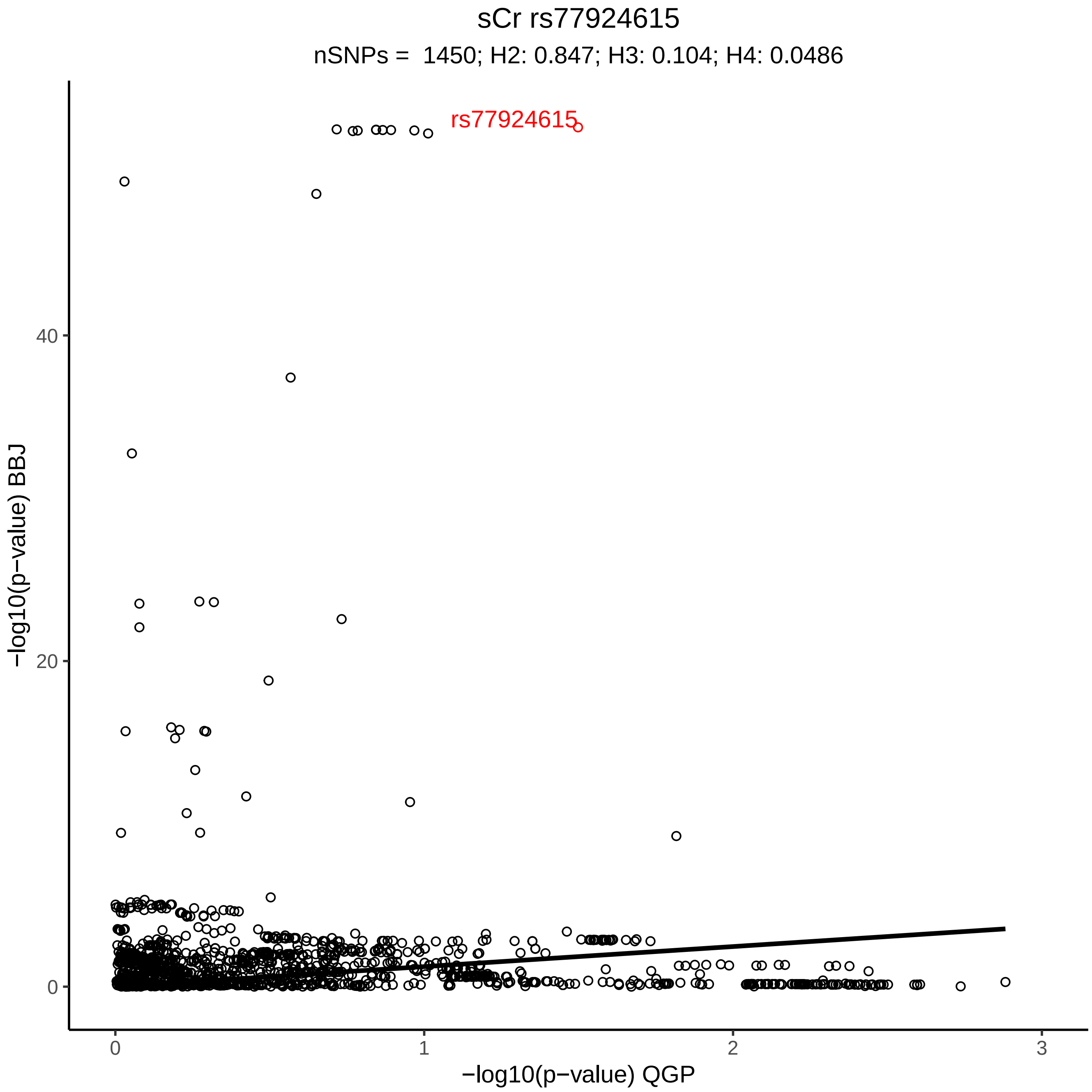
sCr rs77924615
nSNPs = 1450; H2: 0.847; H3: 0.104; H4: 0.0486
rs77924615
−log10(p−value) BBJ
40
20
0
0
1
2
3
−log10(p−value) QGP

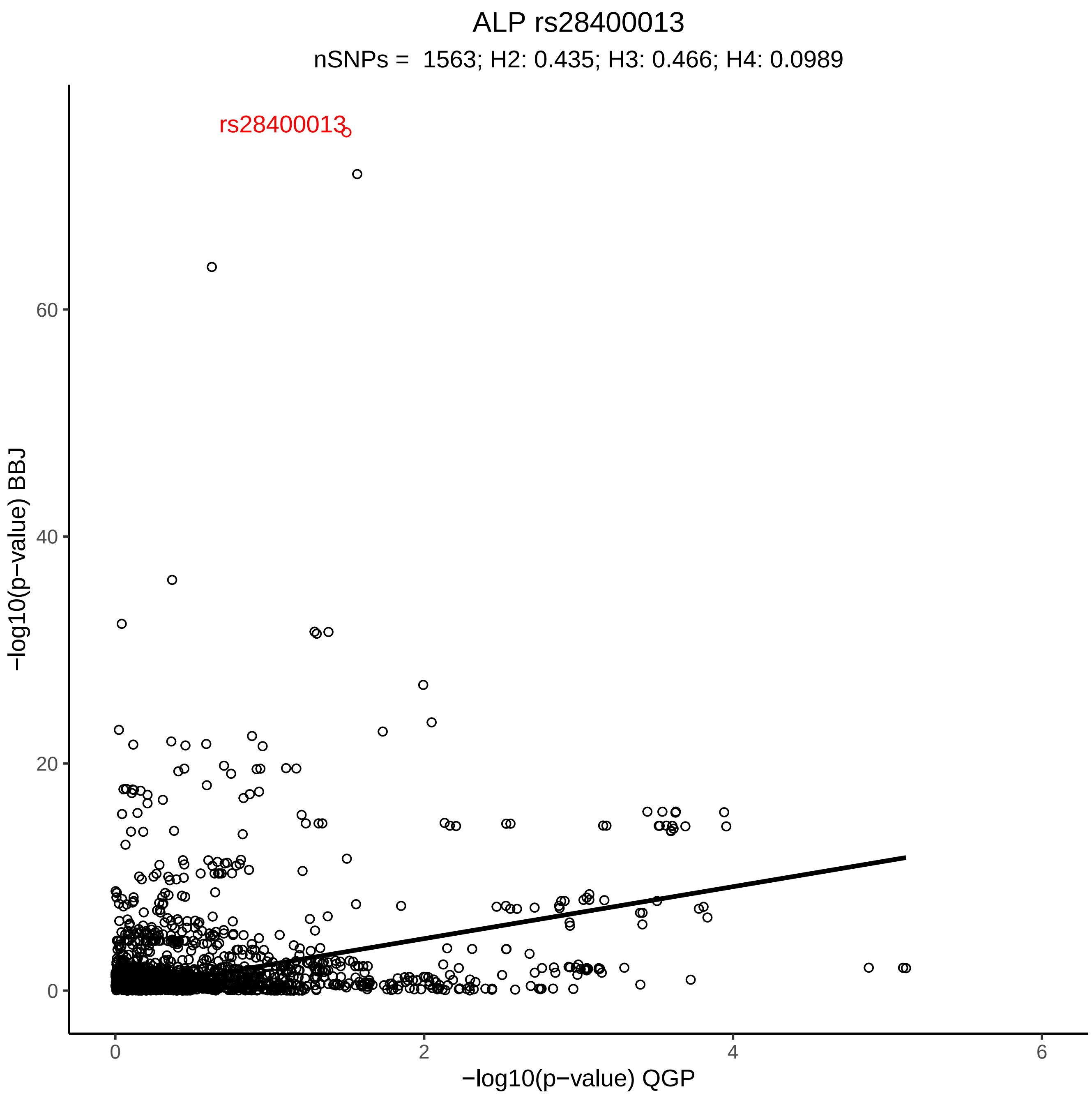

ALP rs28400013
nSNPs = 1563; H2: 0.435; H3: 0.466; H4: 0.0989
rs28400013
−log10(p−value) BBJ
0
20
40
60
−log10(p−value) QGP
0
2
4
6

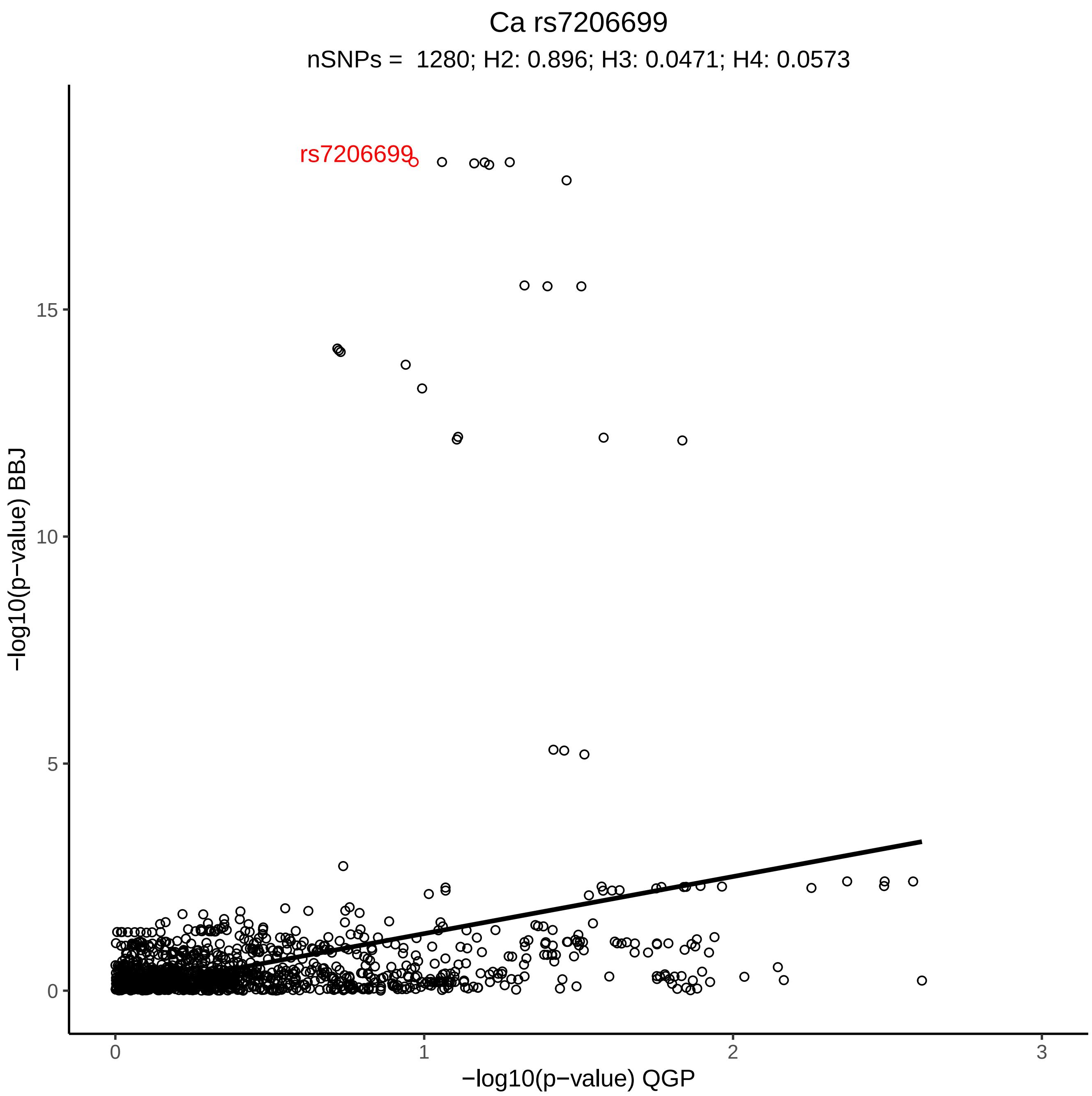
Ca rs7206699
nSNPs = 1280; H2: 0.896; H3: 0.0471; H4: 0.0573
rs7206699
−log10(p−value) BBJ
0
5
10
15
0
1
2
3
−log10(p−value) QGP

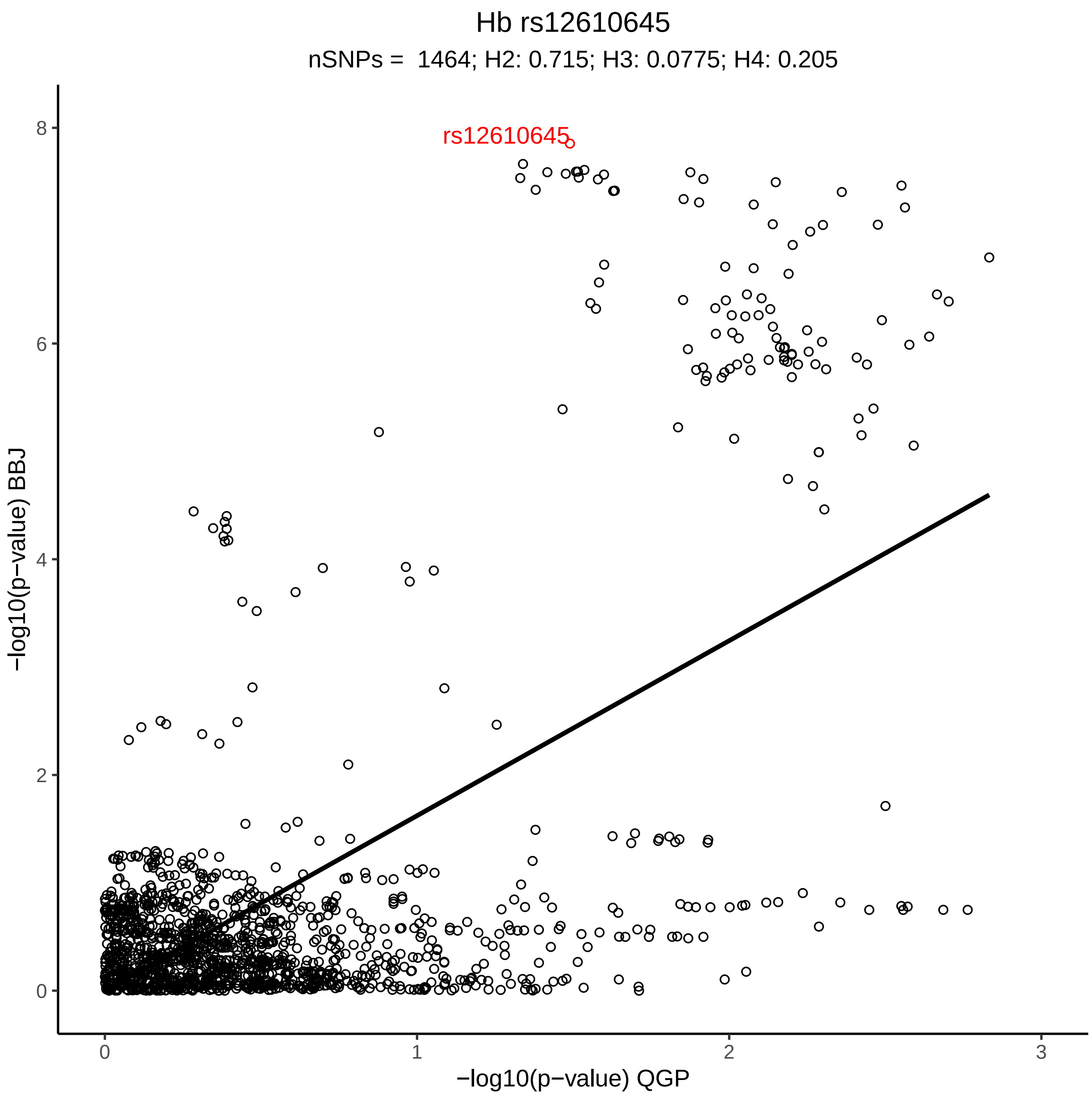
Hb rs12610645
nSNPs = 1464; H2: 0.715; H3: 0.0775; H4: 0.205
rs12610645
8
6
4
2
0
−log10(p−value) BBJ
0
1
2
3
−log10(p−value) QGP

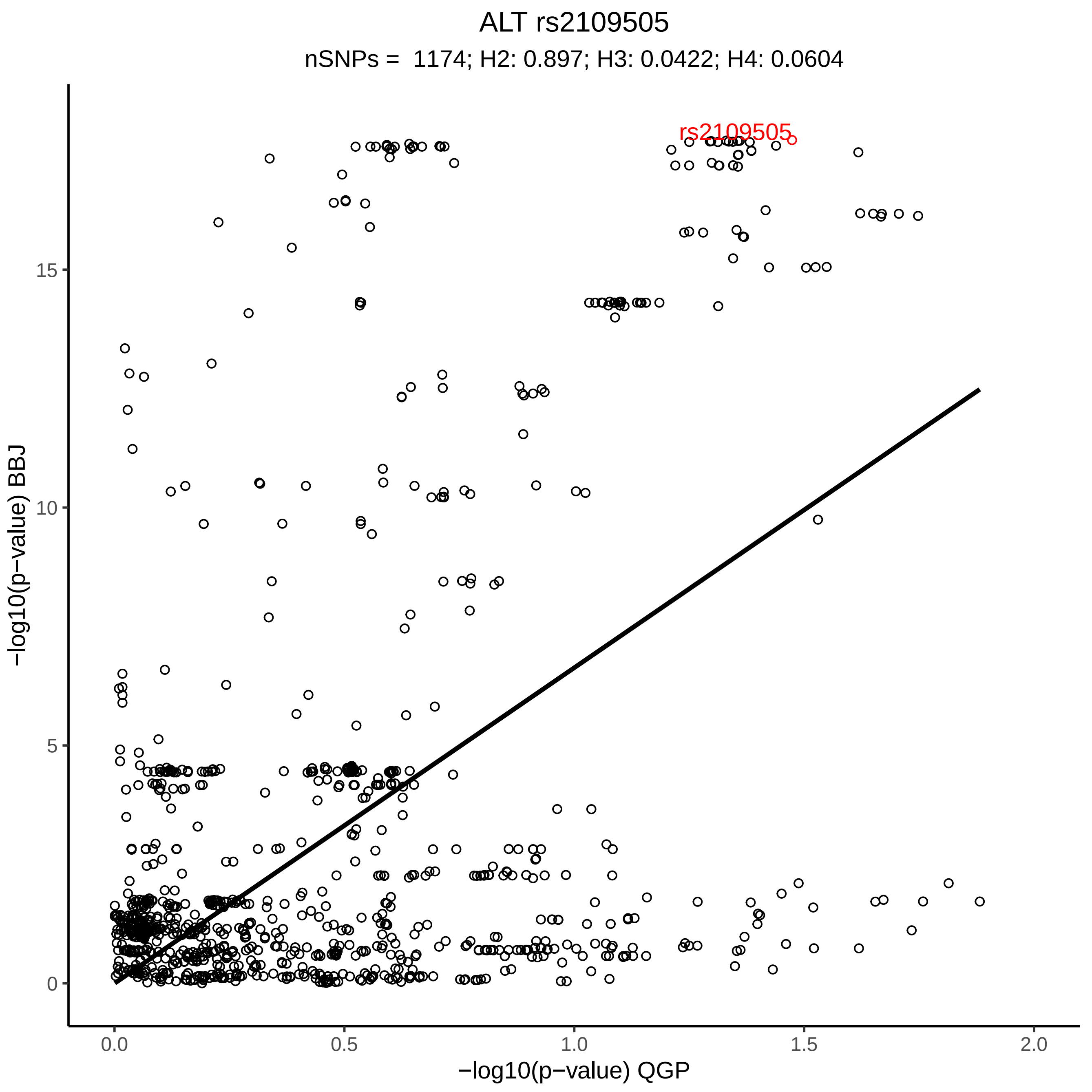
ALT rs2109505
nSNPs = 1174; H2: 0.897; H3: 0.0422; H4: 0.0604
rs2109505
−log10(p−value) BBJ
0
5
10
15
0.0
0.5
1.0
1.5
2.0
−log10(p−value) QGP

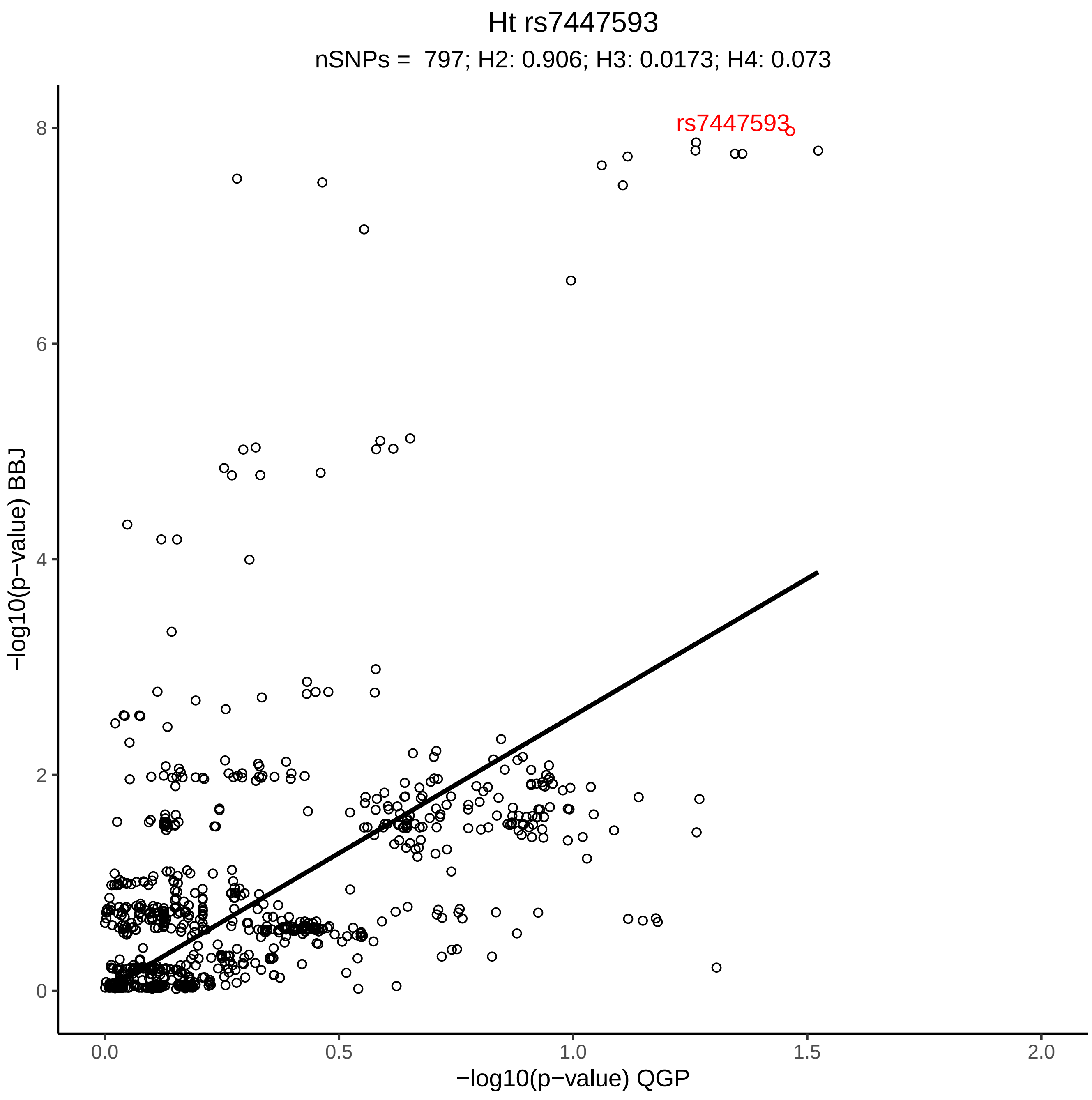

Ht rs7447593
nSNPs = 797; H2: 0.906; H3: 0.0173; H4: 0.073
rs7447593
−log10(p−value) BBJ
−log10(p−value) QGP
0
2
4
6
8
0.0
0.5
1.0
1.5
2.0

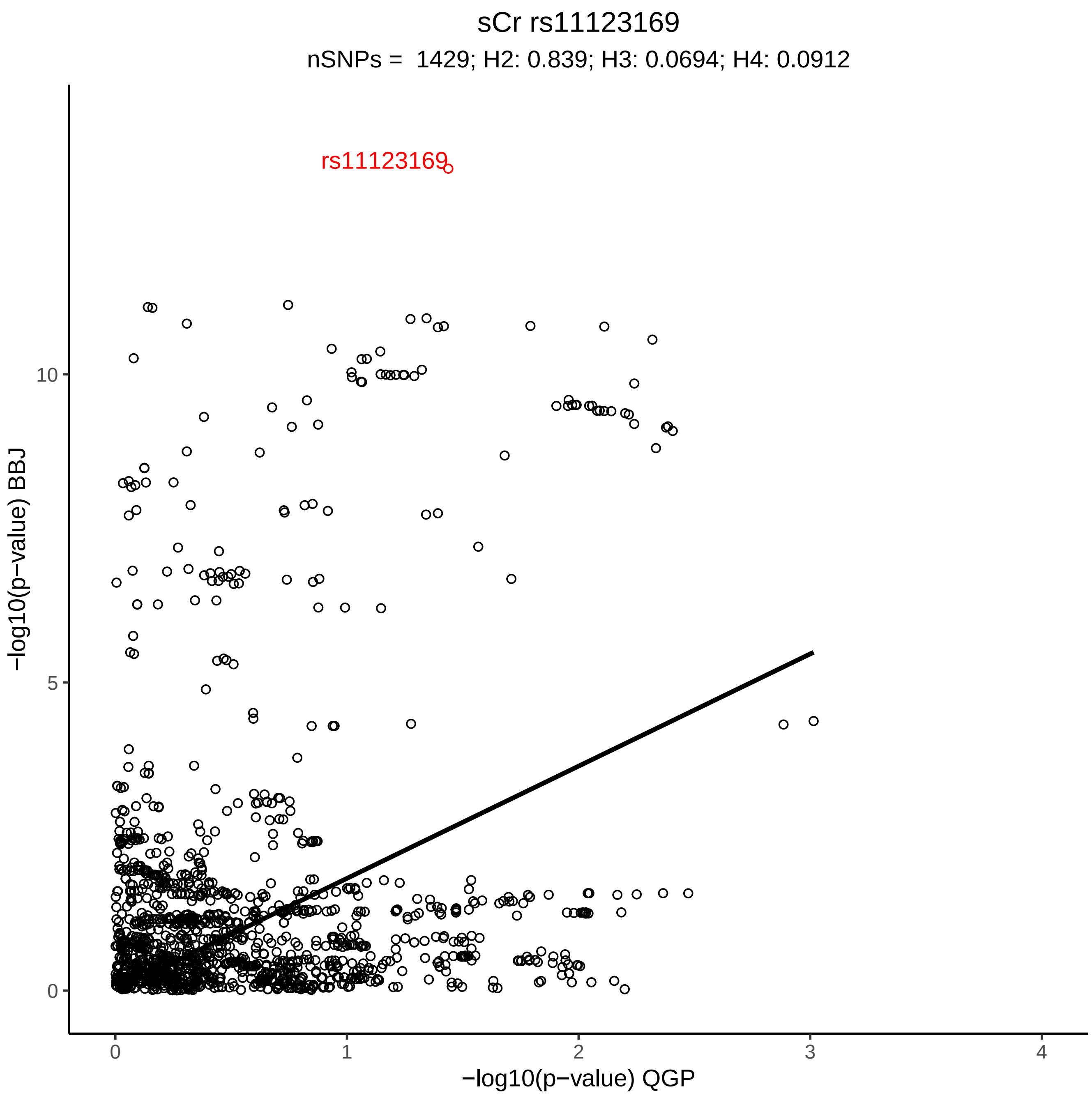
sCr rs11123169
nSNPs = 1429; H2: 0.839; H3: 0.0694; H4: 0.0912
rs11123169
−log10(p−value) BBJ
0
5
10
−log10(p−value) QGP
0
1
2
3
4

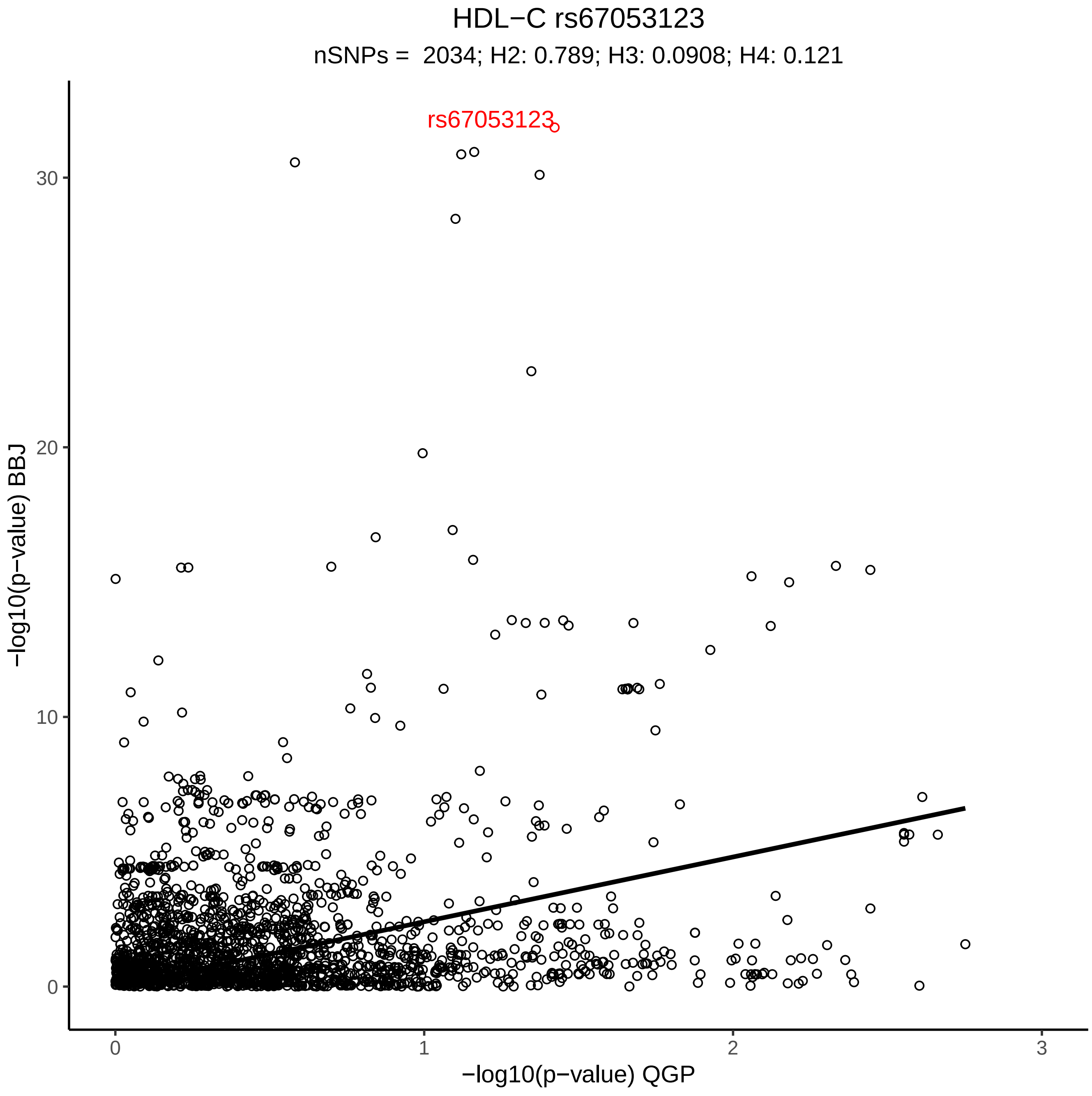
HDL−C rs67053123
nSNPs = 2034; H2: 0.789; H3: 0.0908; H4: 0.121
rs67053123
−log10(p−value) BBJ
−log10(p−value) QGP
0
10
20
30
0
1
2
3

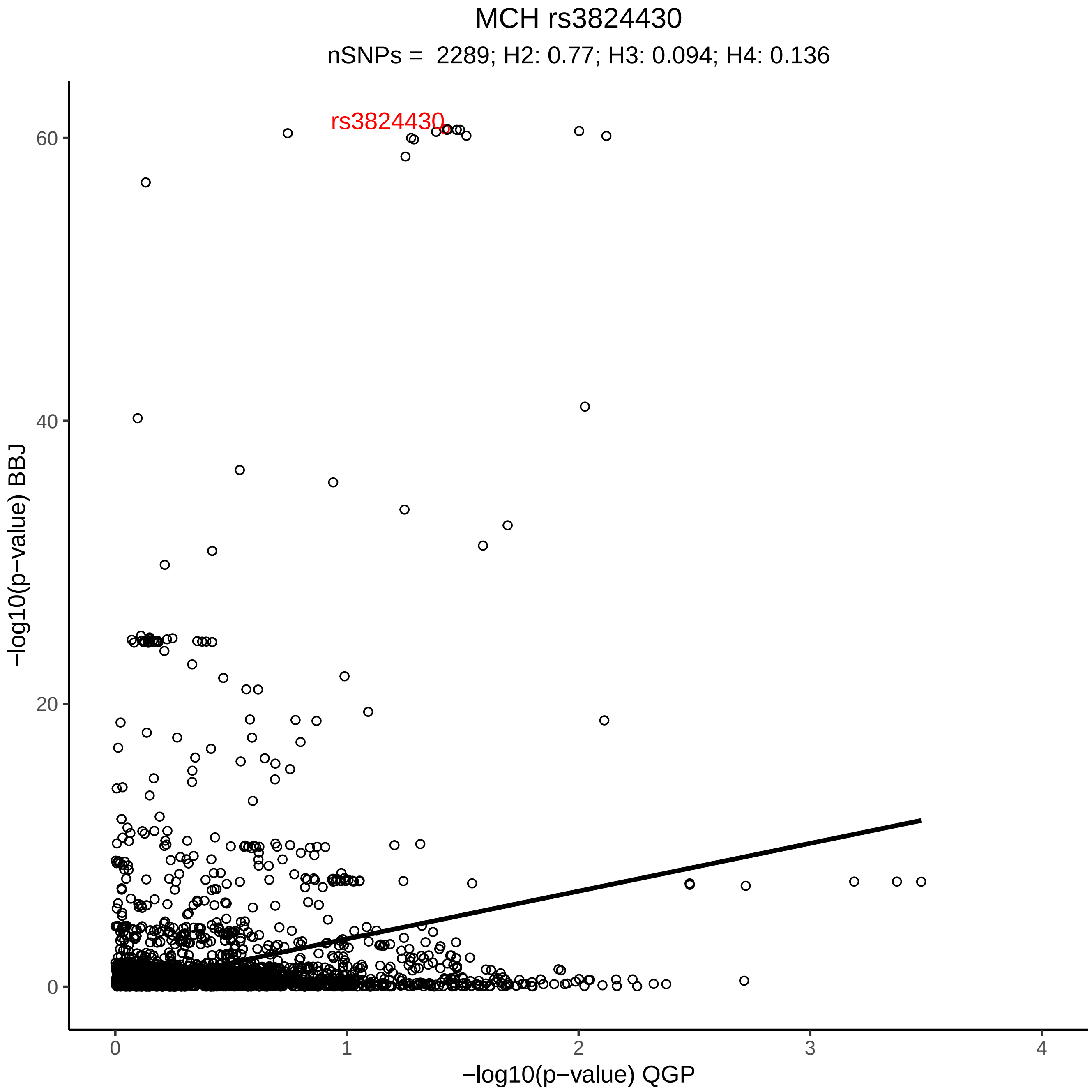
MCH rs3824430
nSNPs = 2289; H2: 0.77; H3: 0.094; H4: 0.136
rs3824430
−log10(p−value) BBJ
−log10(p−value) QGP
0
20
40
60
0
1
2
3
4

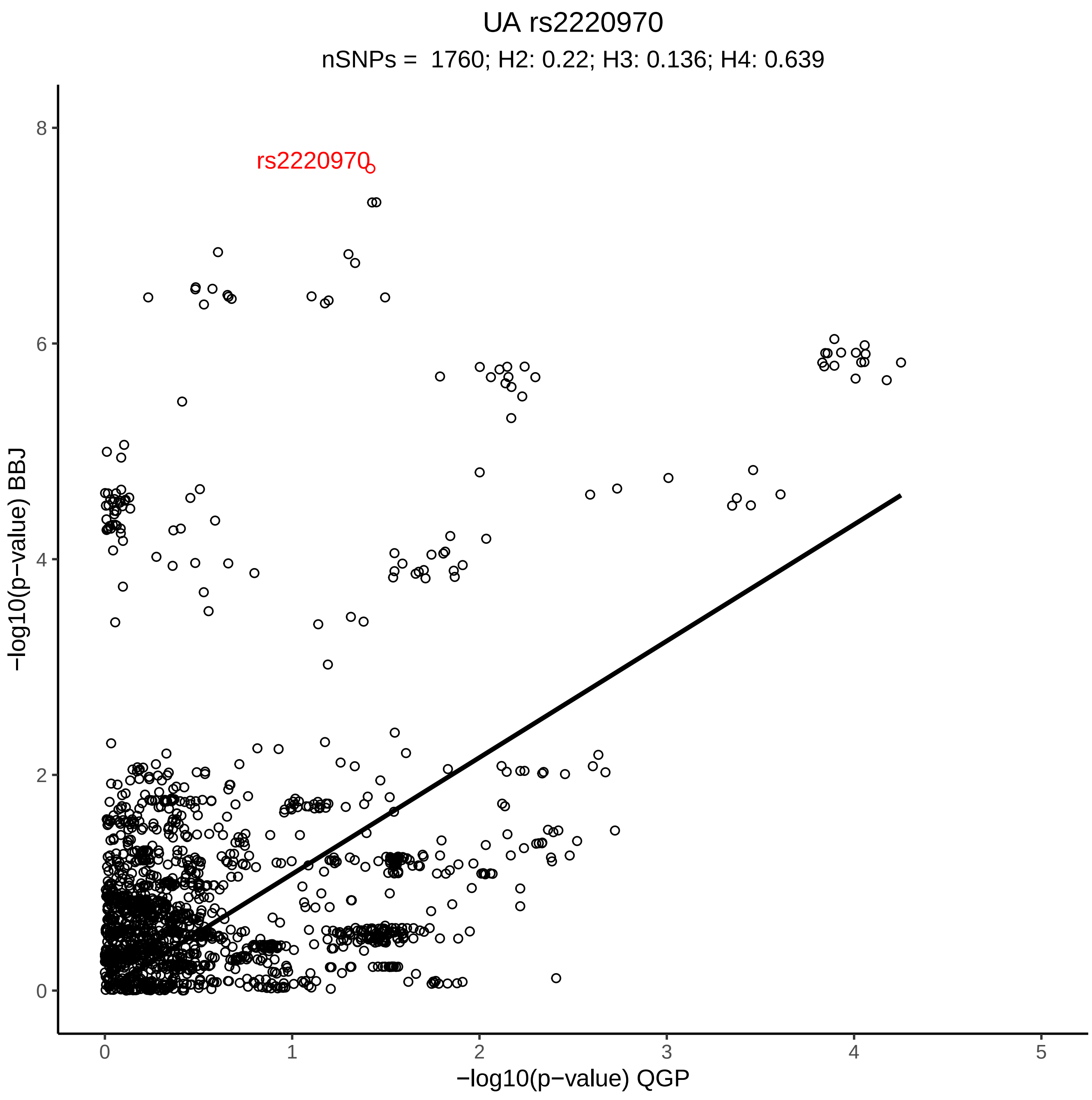
UA rs2220970
nSNPs = 1760; H2: 0.22; H3: 0.136; H4: 0.639
rs2220970
−log10(p−value) BBJ
−log10(p−value) QGP
0
2
4
6
8
0
1
2
3
4
5

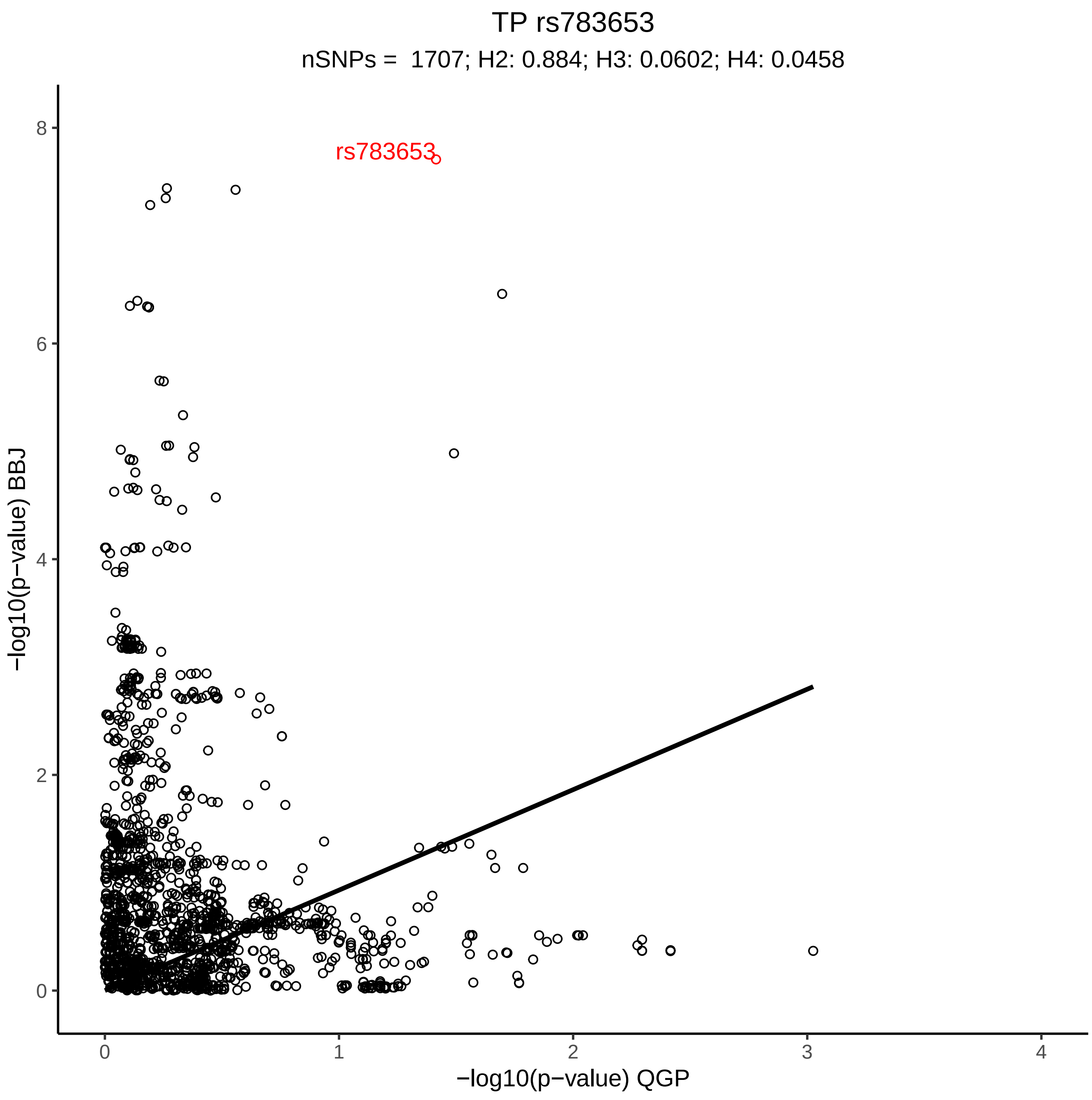

TP rs783653
nSNPs = 1707; H2: 0.884; H3: 0.0602; H4: 0.0458
rs783653
−log10(p−value) BBJ
−log10(p−value) QGP
0
2
4
6
8
0
1
2
3
4

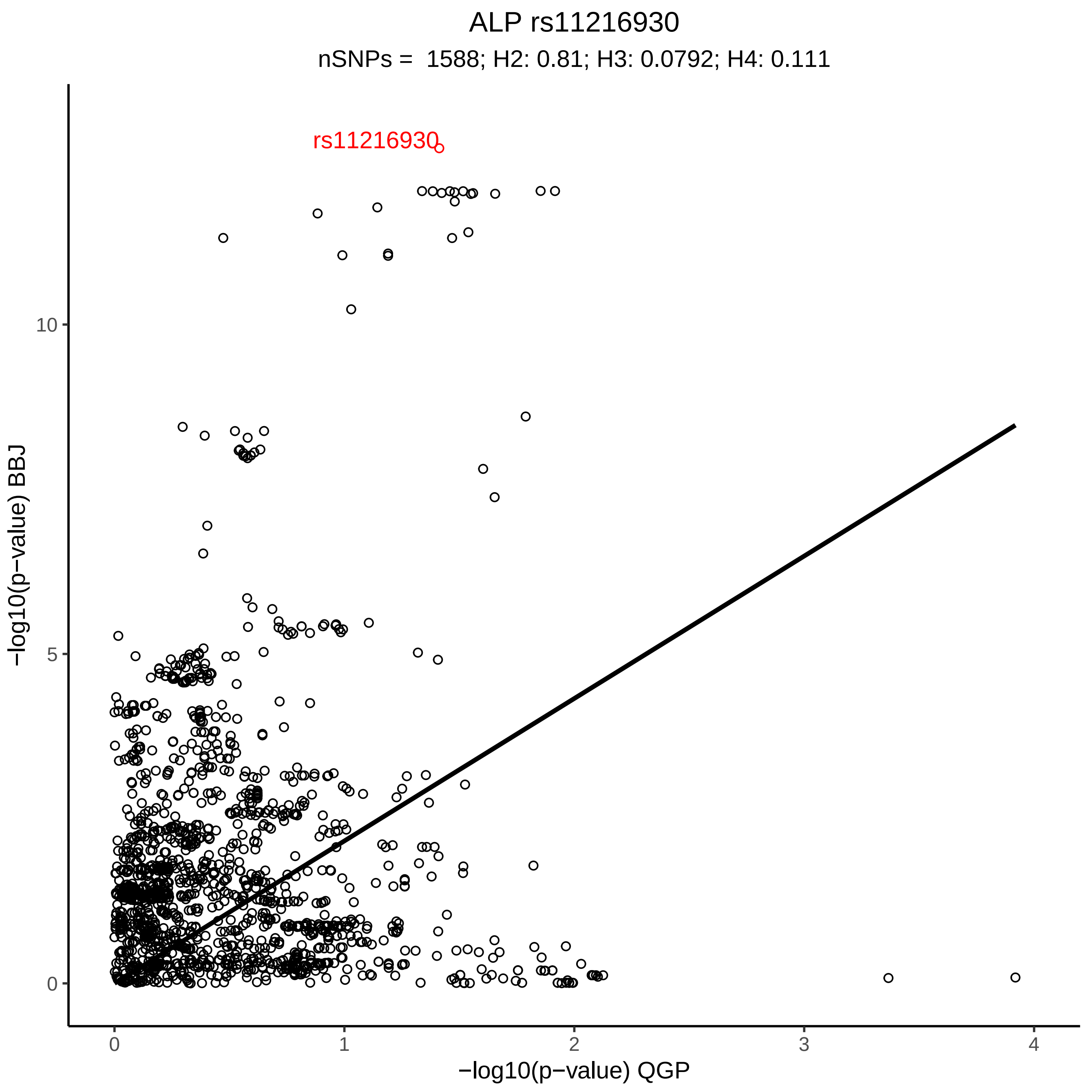

ALP rs11216930
nSNPs = 1588; H2: 0.81; H3: 0.0792; H4: 0.111
rs11216930
−log10(p−value) BBJ
−log10(p−value) QGP
0
5
10
0
1
2
3
4

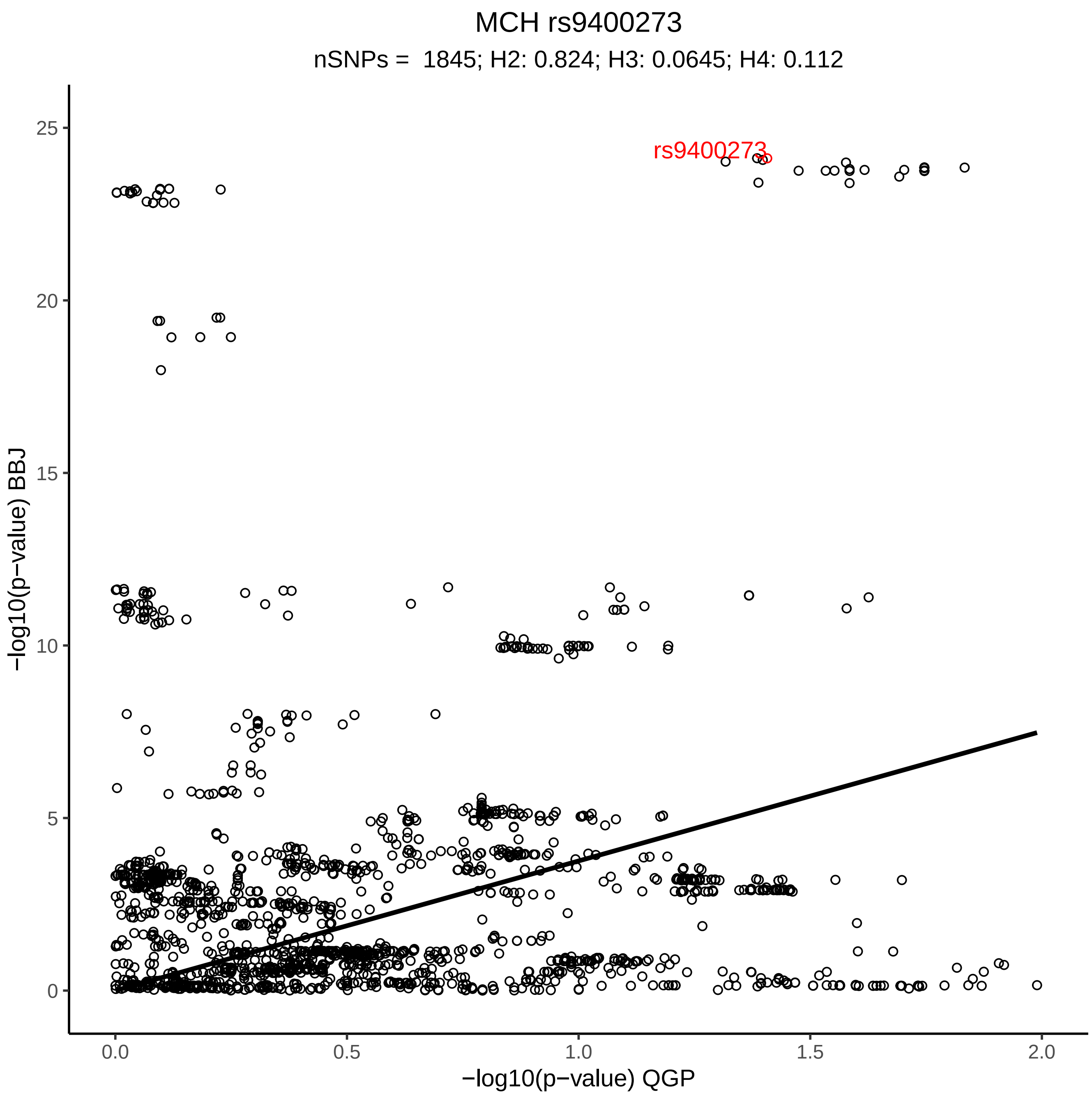
MCH rs9400273
nSNPs = 1845; H2: 0.824; H3: 0.0645; H4: 0.112
rs9400273
−log10(p−value) BBJ
25
20
15
10
5
0
0.0
0.5
1.0
1.5
2.0
−log10(p−value) QGP

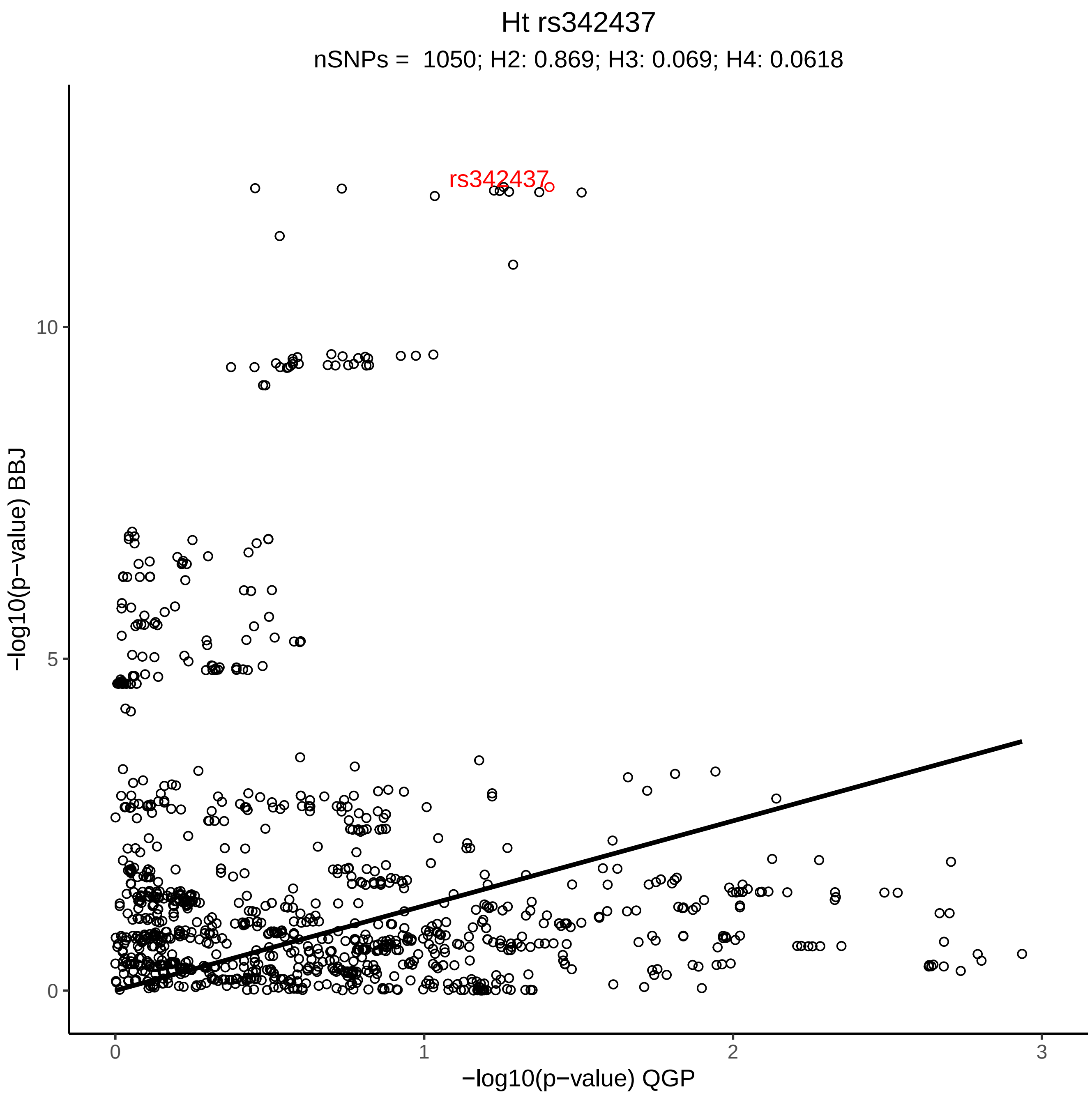

Ht rs342437
nSNPs = 1050; H2: 0.869; H3: 0.069; H4: 0.0618
rs342437
−log10(p−value) BBJ
−log10(p−value) QGP
0
5
10
0
1
2
3

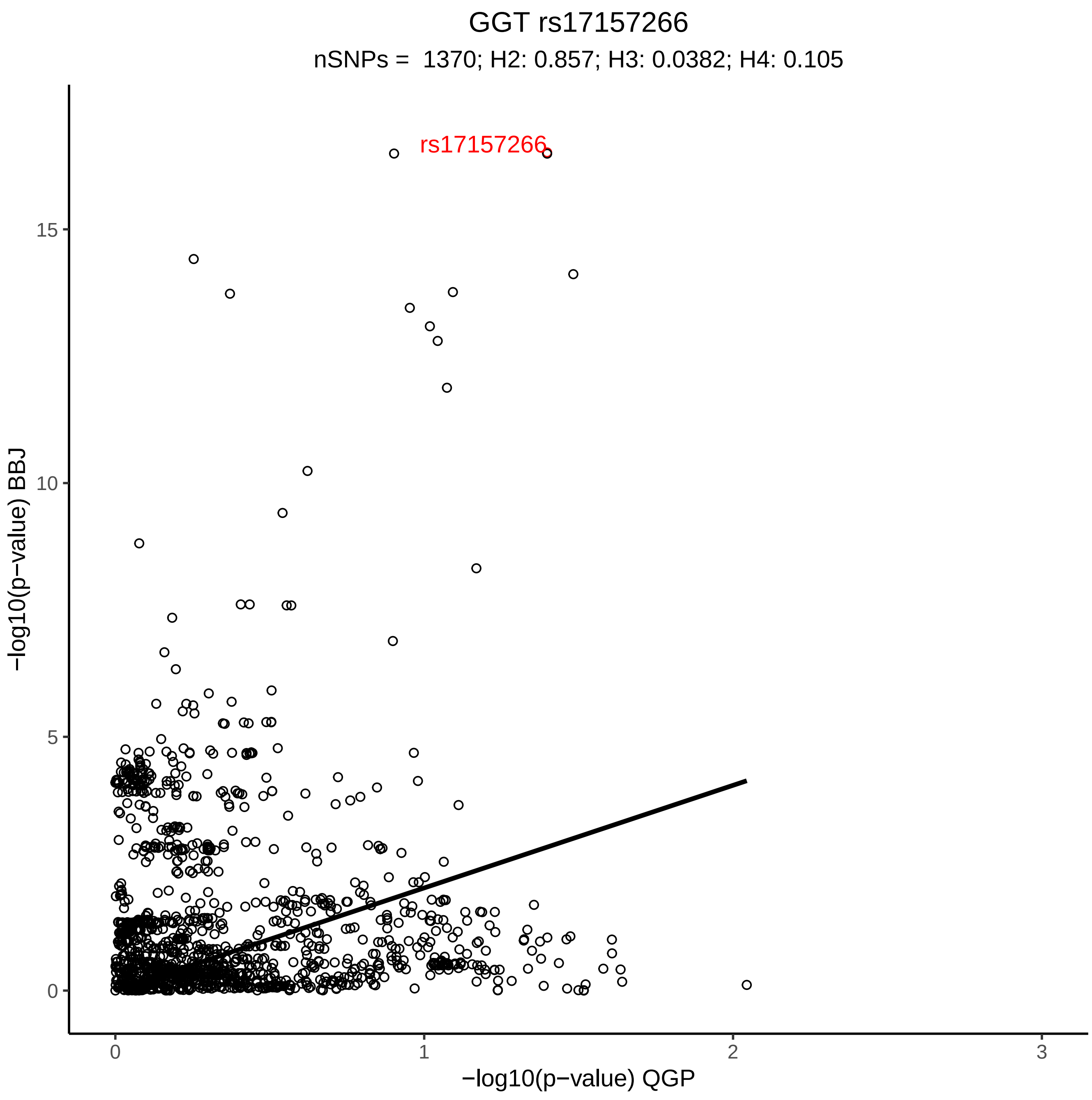
GGT rs17157266
nSNPs = 1370; H2: 0.857; H3: 0.0382; H4: 0.105
rs17157266
−log10(p−value) BBJ
−log10(p−value) QGP
0
5
10
15
0
1
2
3

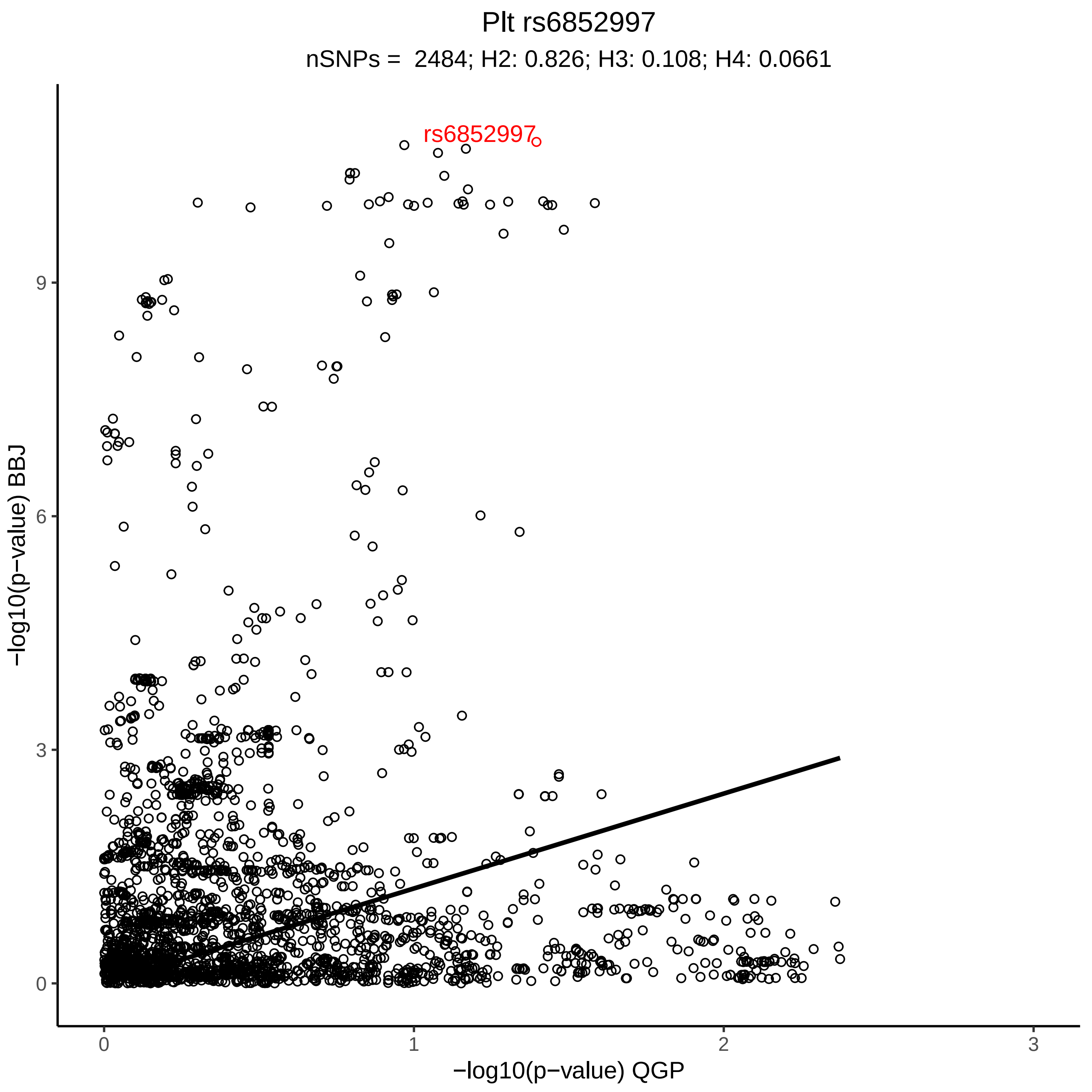
Plt rs6852997
nSNPs = 2484; H2: 0.826; H3: 0.108; H4: 0.0661
rs6852997
−log10(p−value) BBJ
0
3
6
9
0
1
2
3
−log10(p−value) QGP

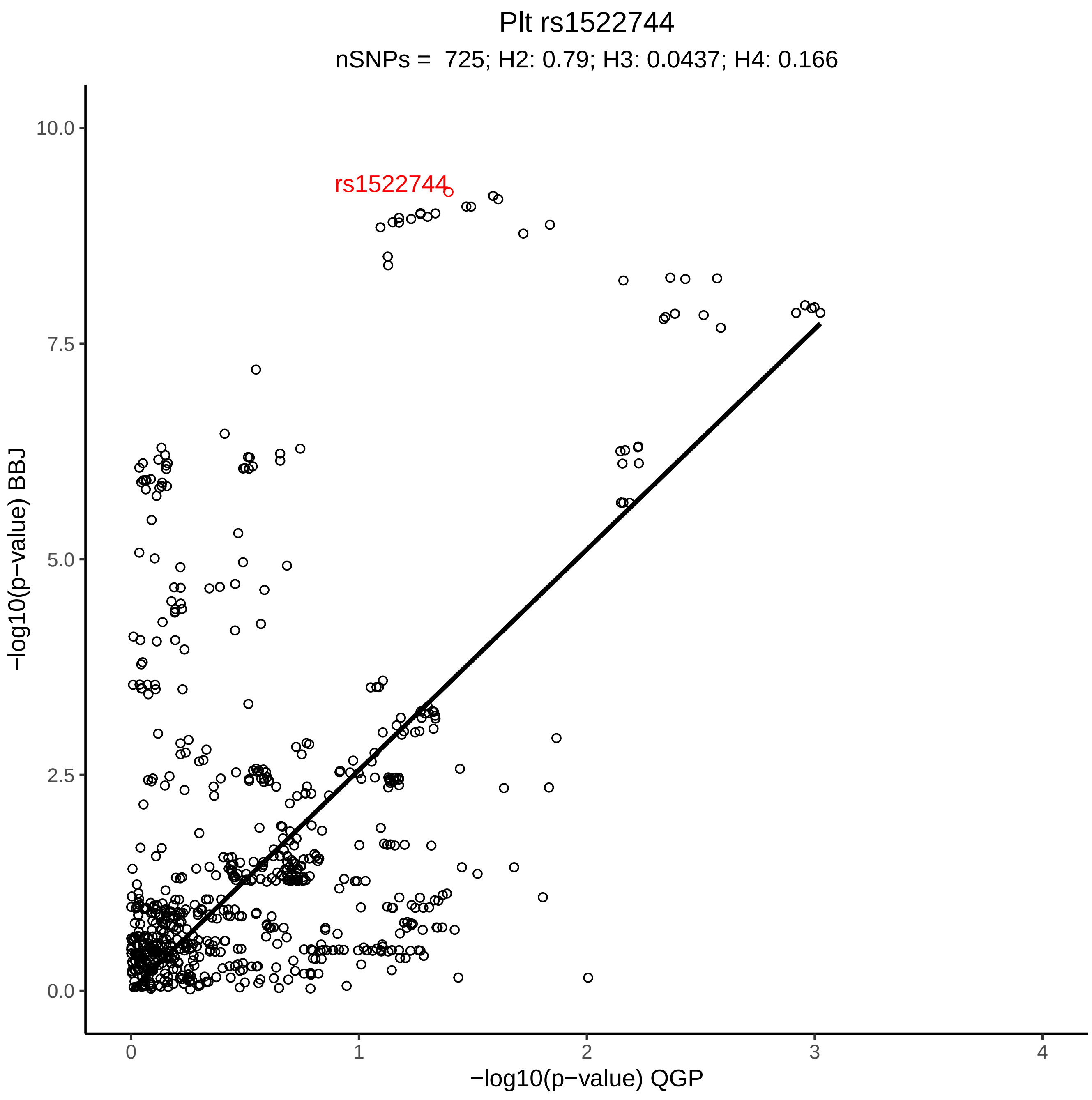
Plt rs1522744
nSNPs = 725; H2: 0.79; H3: 0.0437; H4: 0.166
rs1522744
−log10(p−value) BBJ
−log10(p−value) QGP
0.0
2.5
5.0
7.5
10.0
0
1
2
3
4

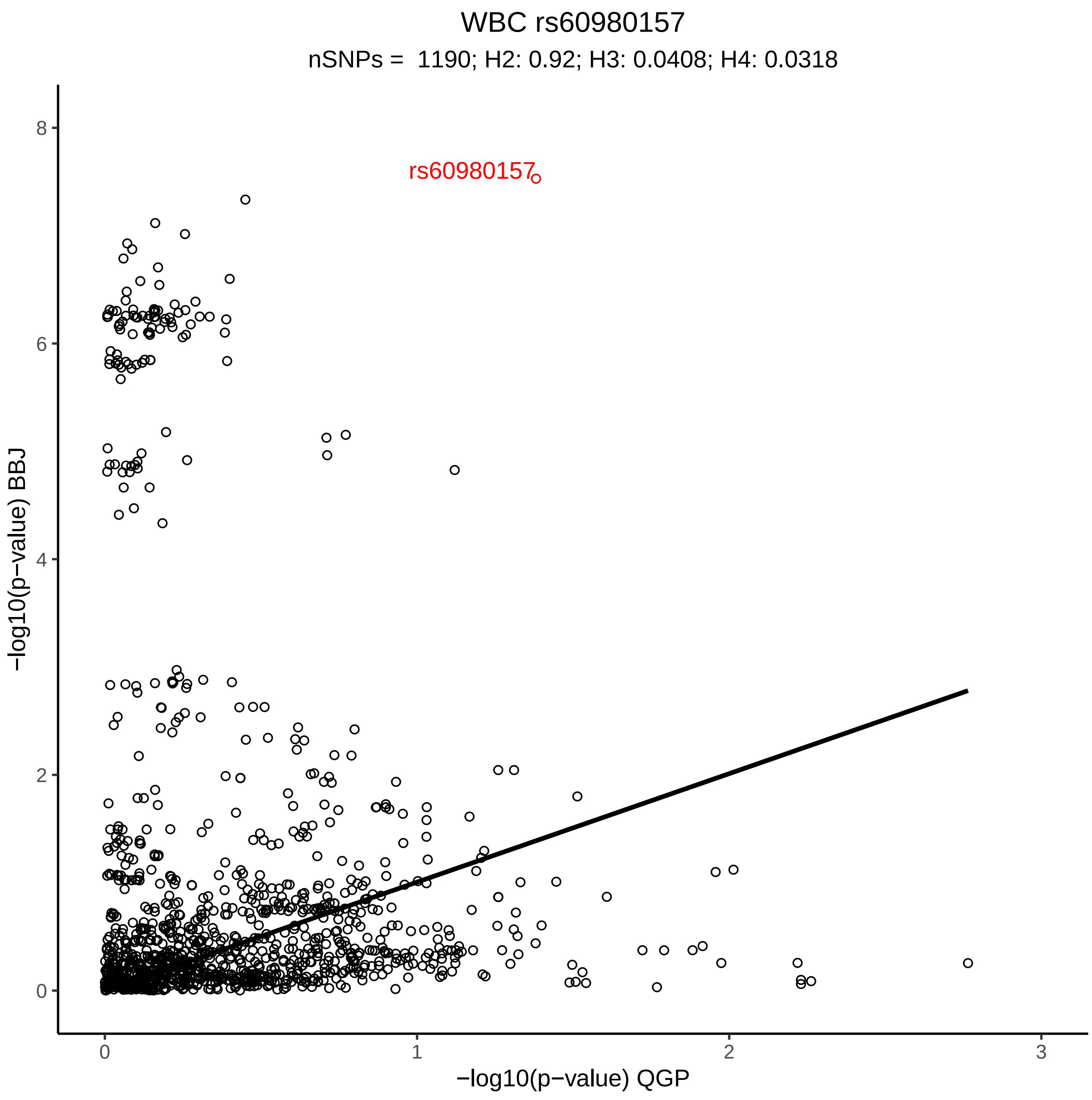

WBC rs60980157
nSNPs = 1190; H2: 0.92; H3: 0.0408; H4: 0.0318
rs60980157
−log10(p−value) BBJ
−log10(p−value) QGP
0
2
4
6
8
0
1
2
3

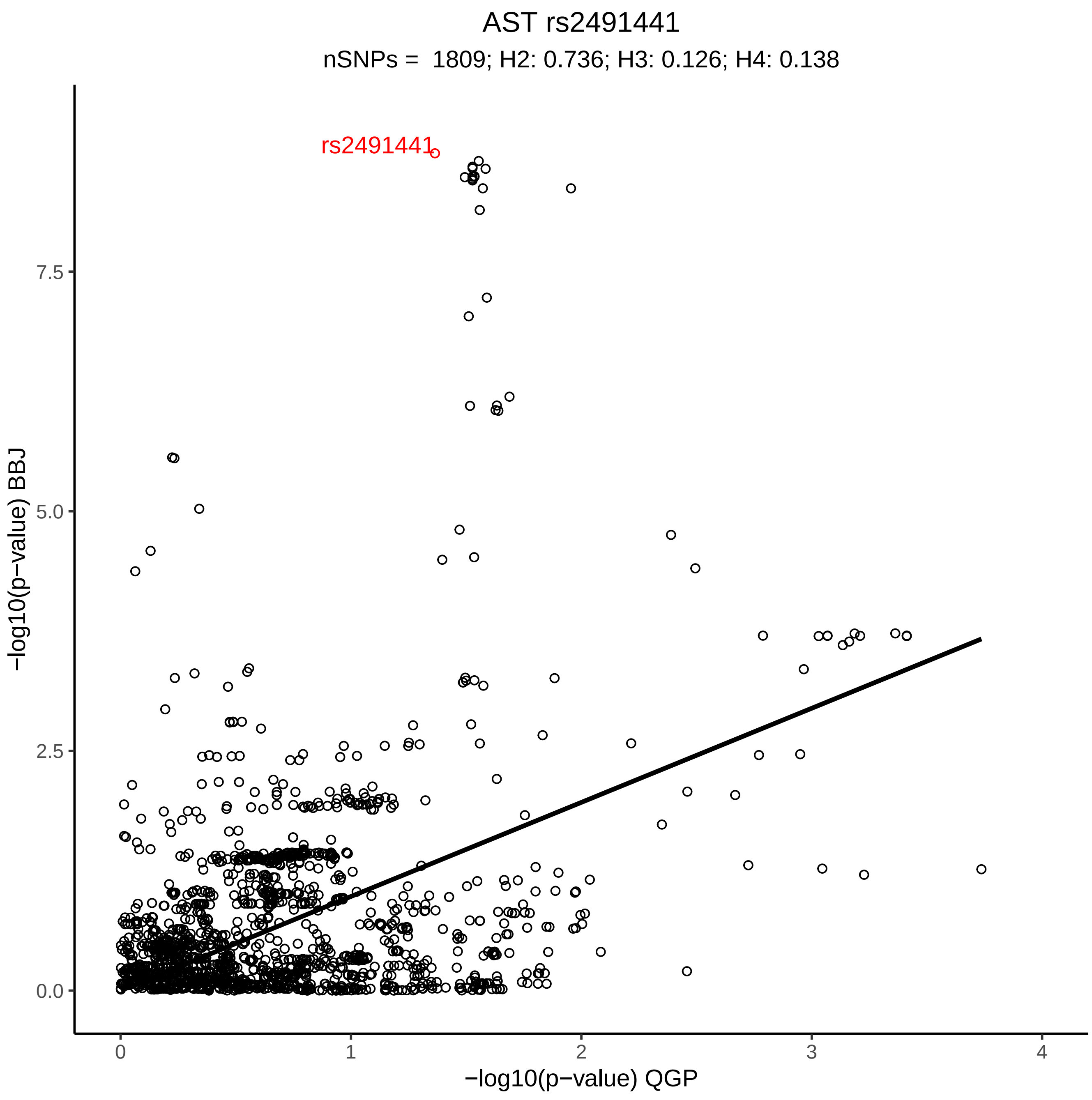

AST rs2491441
nSNPs = 1809; H2: 0.736; H3: 0.126; H4: 0.138
rs2491441
−log10(p−value) BBJ
0.0
2.5
5.0
7.5
0
1
2
3
4
−log10(p−value) QGP

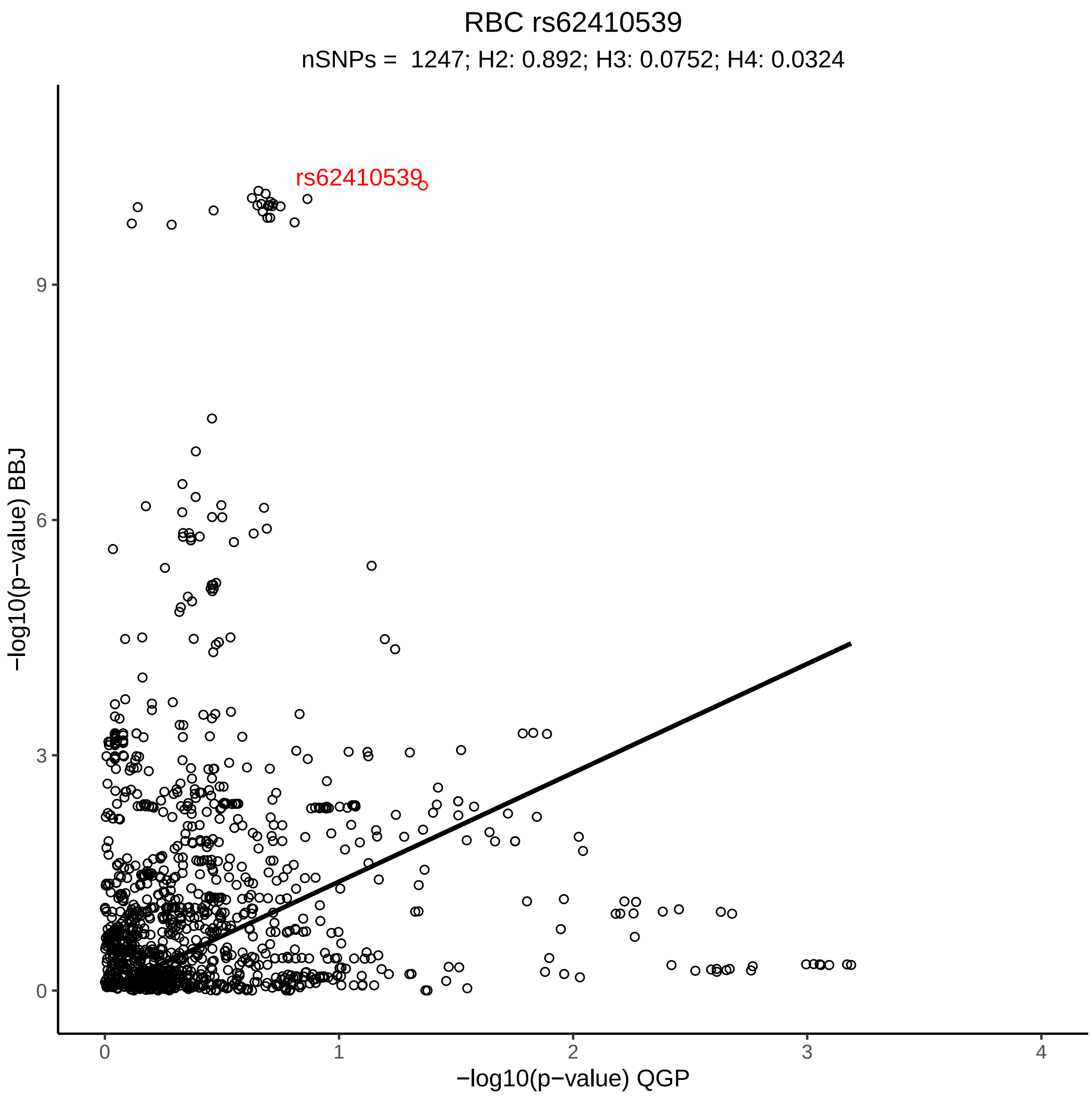
RBC rs62410539
nSNPs = 1247; H2: 0.892; H3: 0.0752; H4: 0.0324
rs62410539
−log10(p−value) BBJ
−log10(p−value) QGP
0
3
6
9
0
1
2
3
4

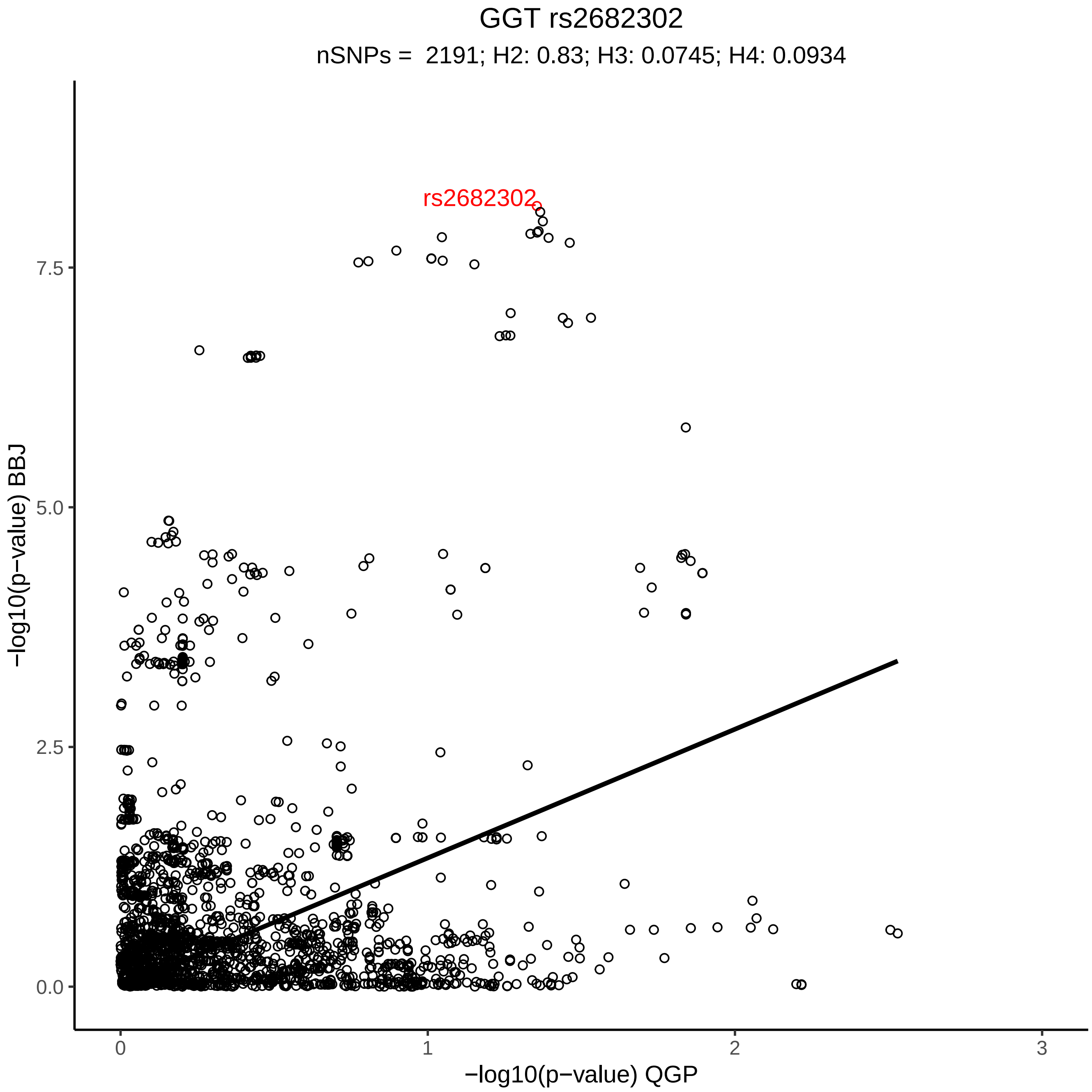
GGT rs2682302
nSNPs = 2191; H2: 0.83; H3: 0.0745; H4: 0.0934
rs2682302
7.5
5.0
2.5
0.0
−log10(p−value) BBJ
0
1
2
3
−log10(p−value) QGP

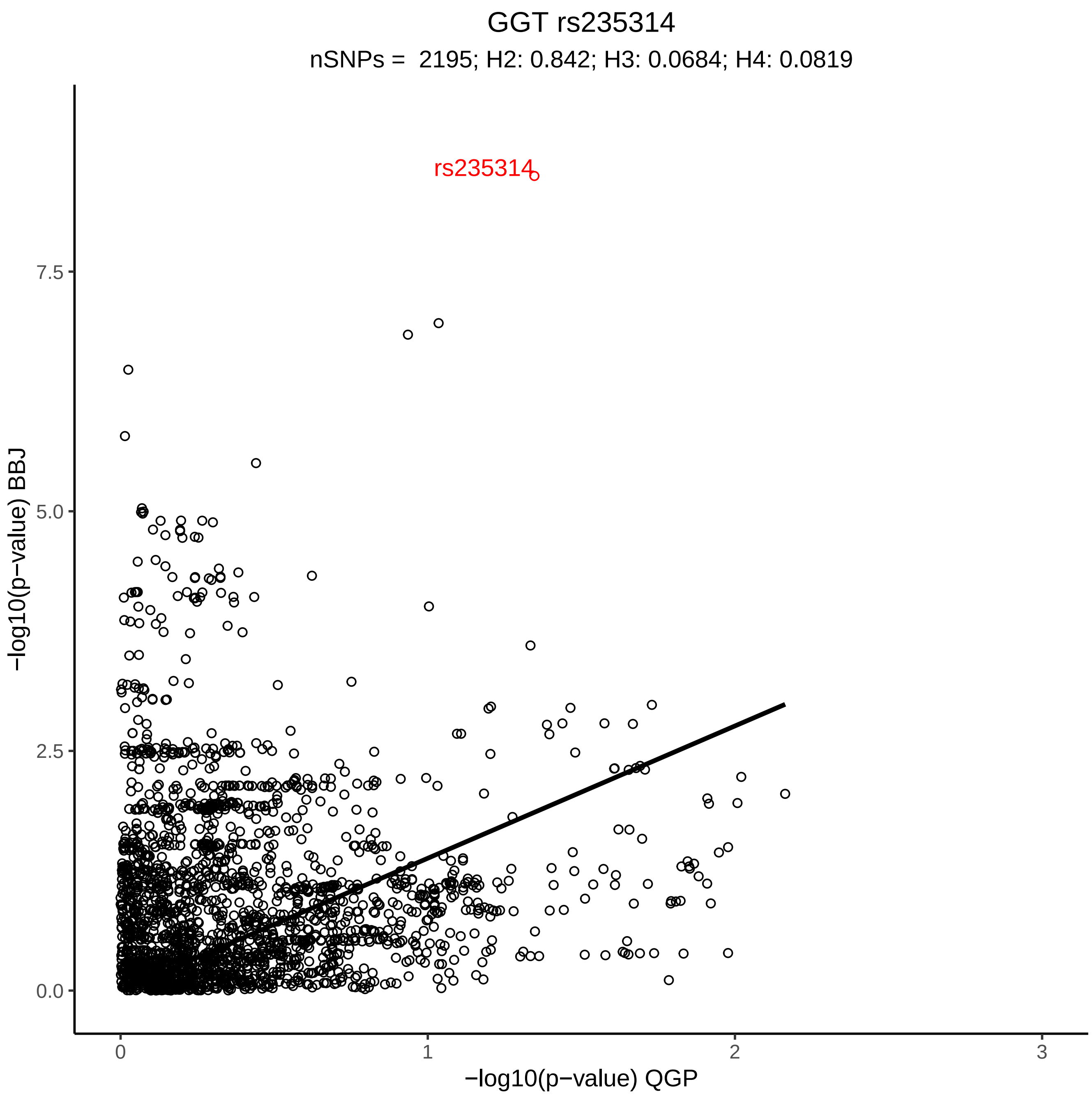
GGT rs235314
nSNPs = 2195; H2: 0.842; H3: 0.0684; H4: 0.0819
rs235314
7.5
5.0
2.5
0.0
−log10(p−value) BBJ
0
1
2
3
−log10(p−value) QGP

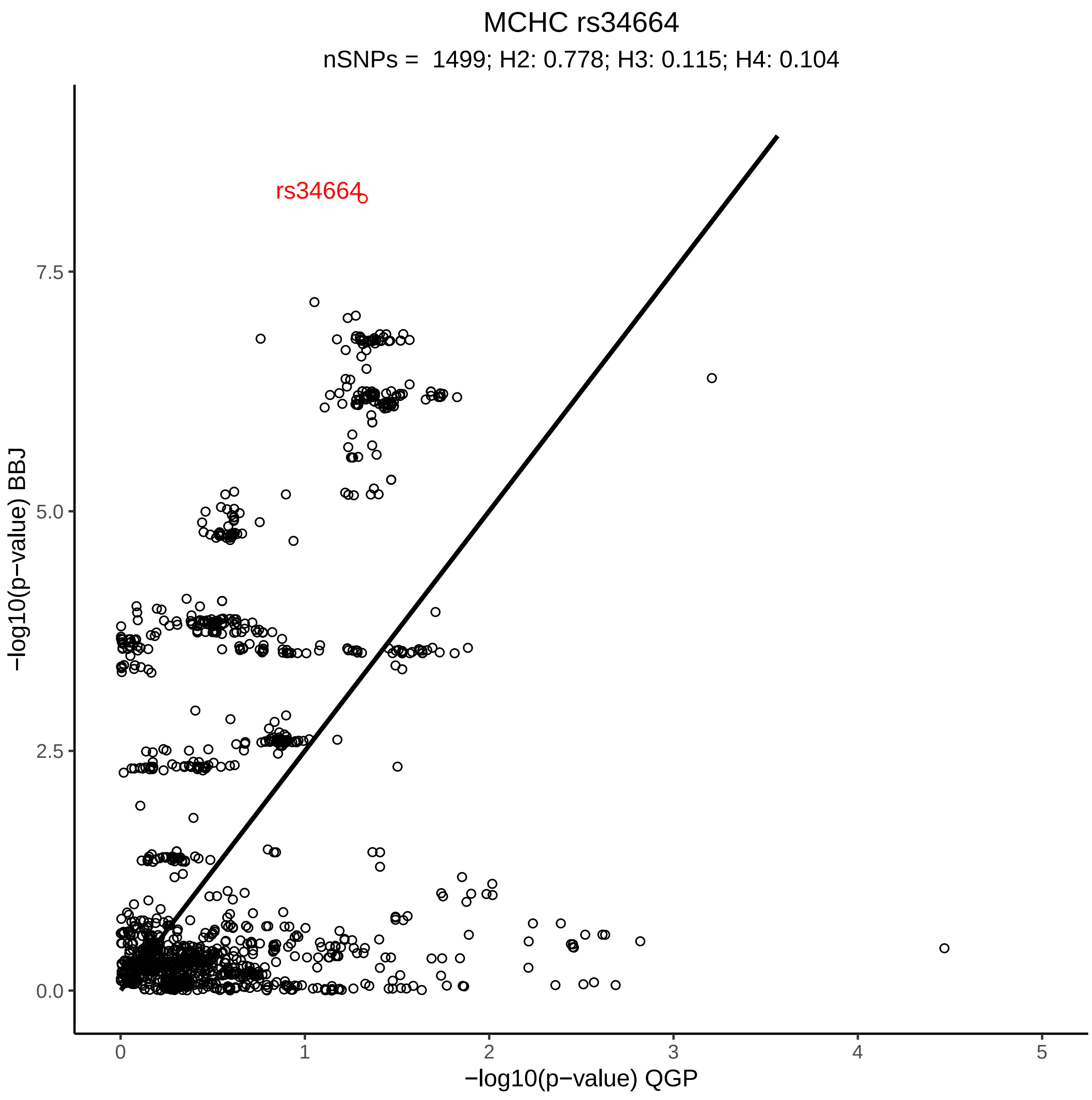

MCHC rs34664
nSNPs = 1499; H2: 0.778; H3: 0.115; H4: 0.104
rs34664
7.5
5.0
2.5
0.0
0
1
2
3
4
5
−log10(p−value) BBJ
−log10(p−value) QGP
