# Supplementary material for: Whole genome sequencing in the Middle Eastern Qatari population identifies genetic associations with 45 clinically relevant traits
Source: Nat Commun. 2021 Feb 23;12:1250. doi: 10.1038/s41467-021-21381-3 (PMC7902658; doi:10.1038/s41467-021-21381-3)

Supplementary Data 6. Regional association plots for the novel loci identified in this study. The chromosomal positions of variants are plotted against $-\log_{10}$ (P). P-values obtained from GWAS analysis of QGP data using linear mixed models adjusting for age, sex, population principal components and relatedness. Variants are colored based on linkage disequilibrium with the lead variant (diamond shape). Blue lines represent recombination rate.

Index

Trait	Variant ID	Page
Hcyst	rs147242481	2
Hcyst	rs187169250	2
Hcyst	rs71469261	2
INR	rs865897814	2
Mg	rs189260309	2
PT	rs865897814	2
Tbil	rs2204511	3
Tbil	rs182021046	3
Tbil	rs183884248	3
TG	rs376997679	3
UA	rs143909619	3
Vit-B12	rs143522487	3
WBC	rs12080243	4
WBC	rs11809289	4
WBC	rs76537384	4
WBC	rs6677720	4
WBC	rs143969748	4

Hcyst

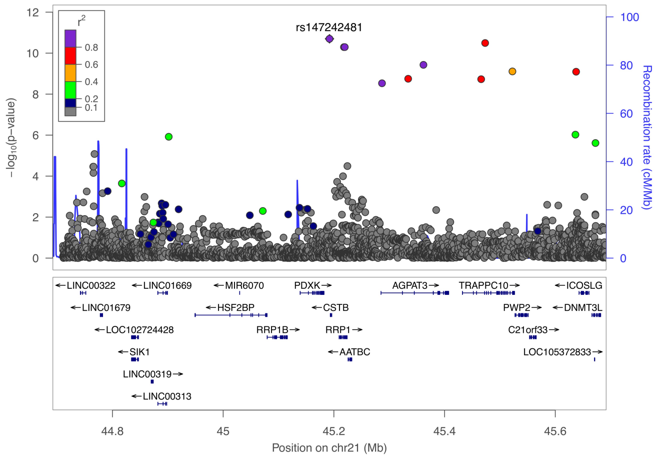

Hcyst

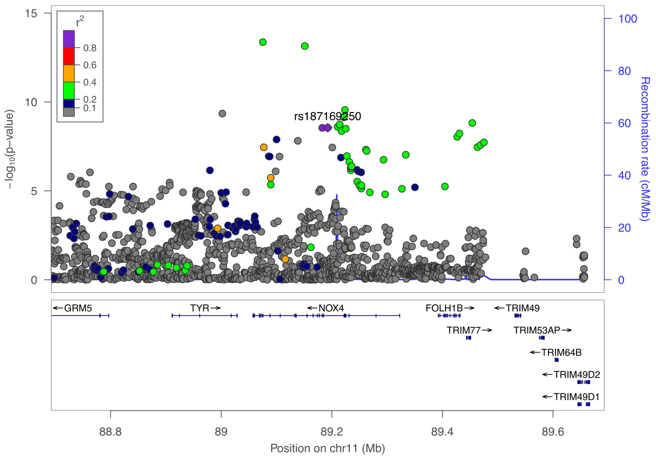

Hcyst

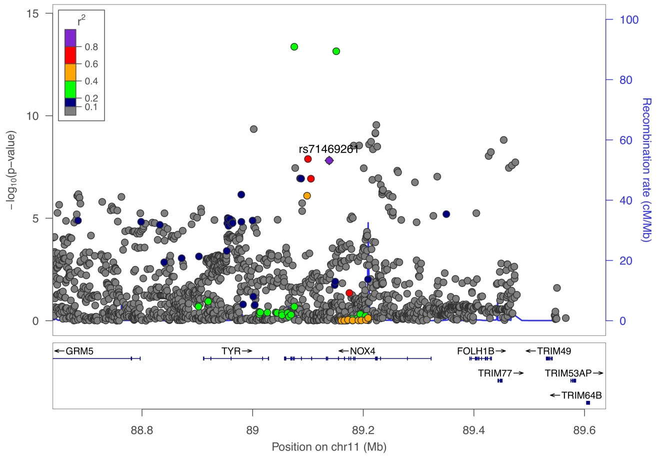

INR

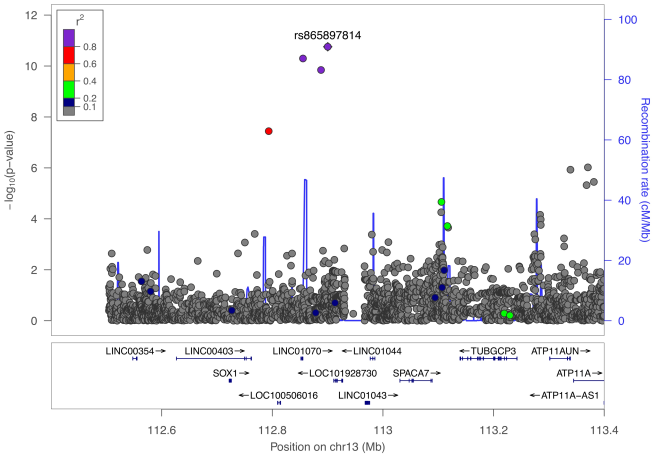

Mg

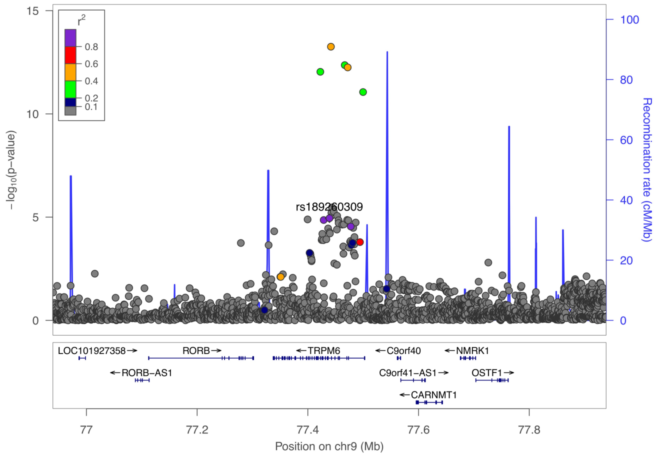

PT

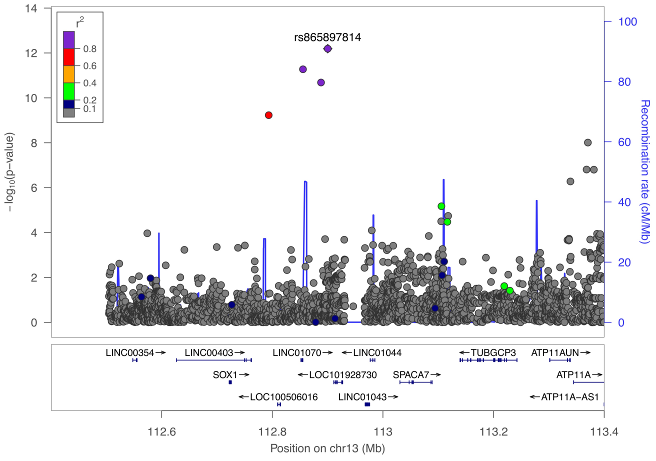

Tbil

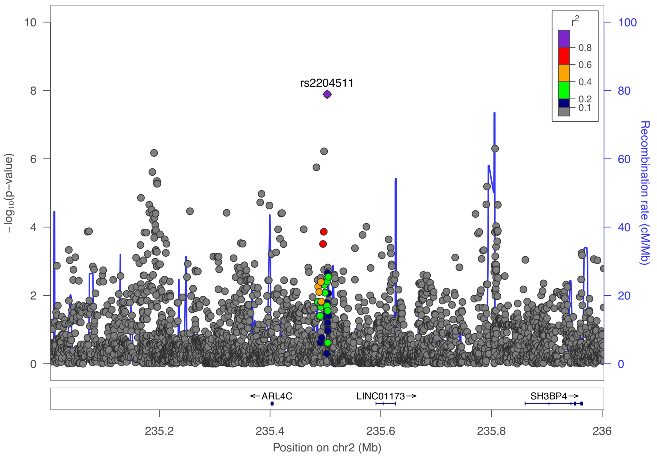

Tbil

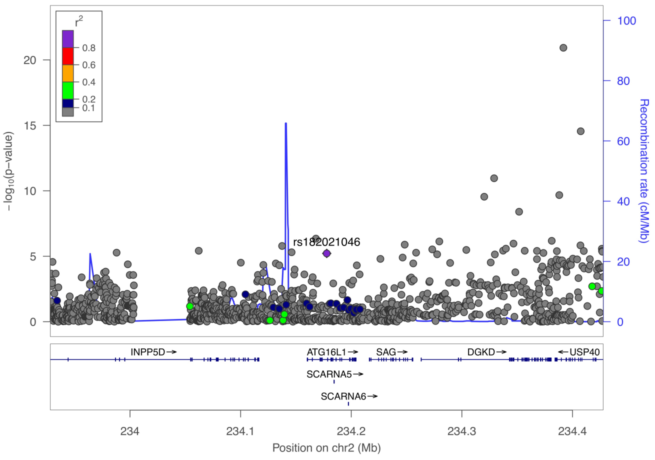

Tbil

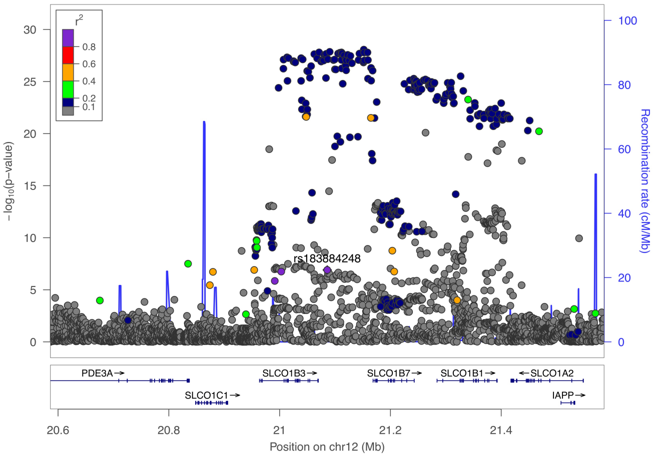

TG

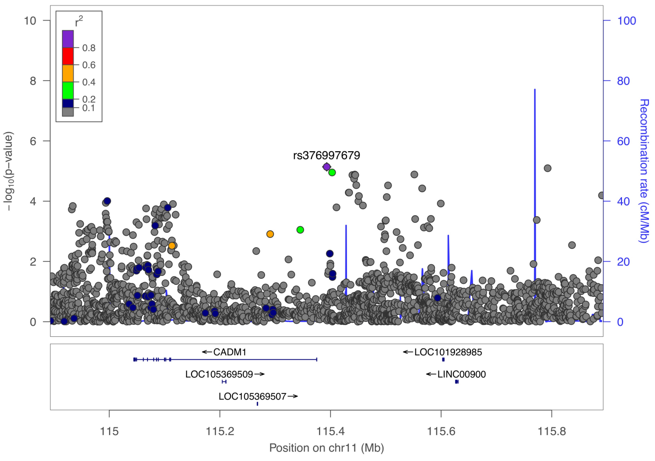

UA

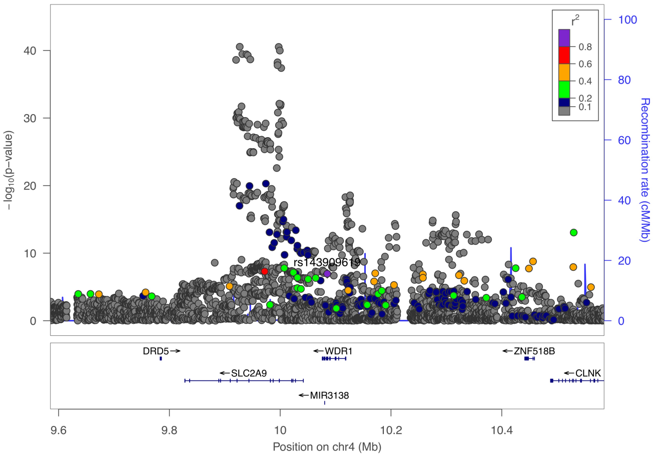

Vit-B12

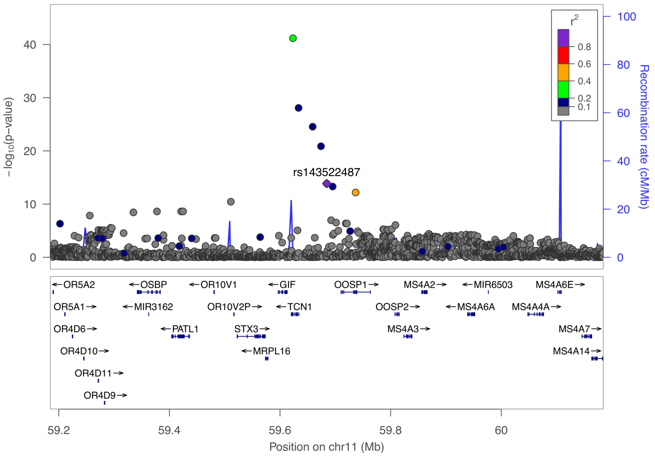

WBC

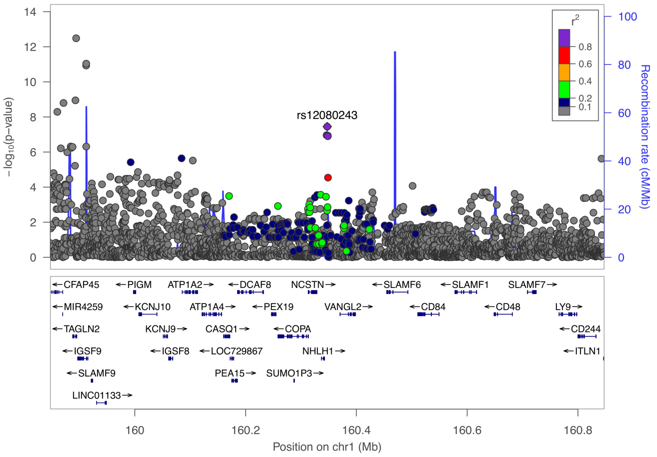

WBC

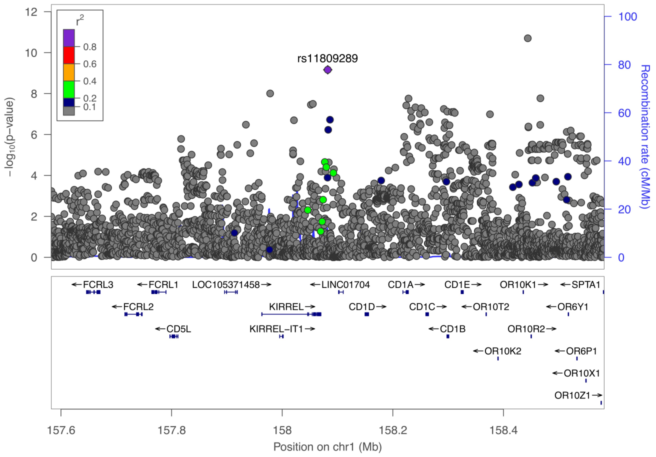

WBC

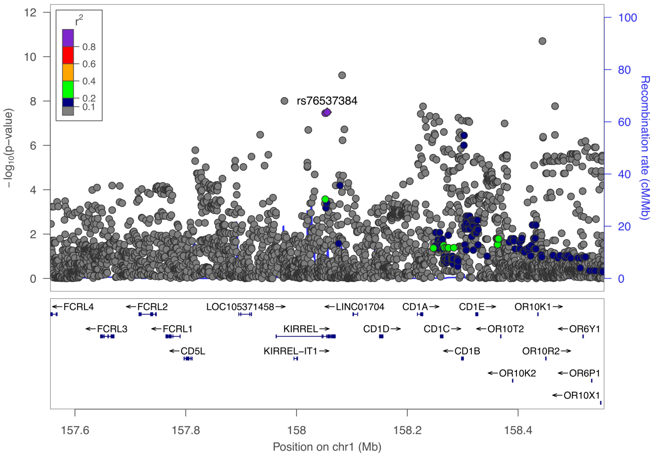

WBC

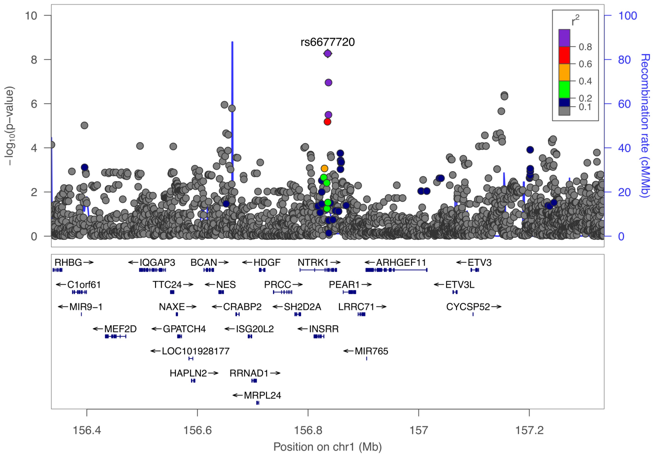

WBC

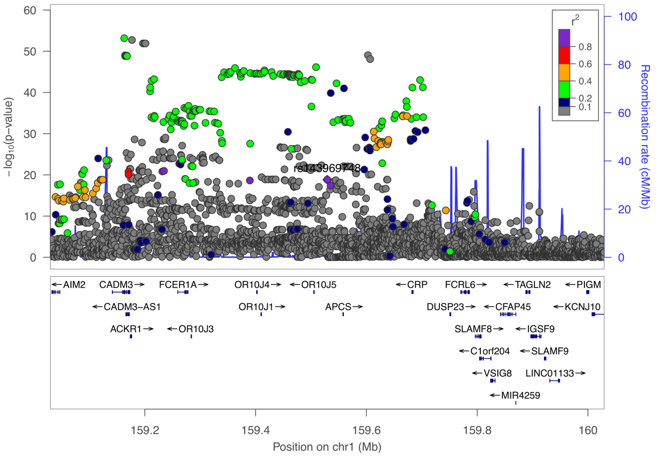

Supplement: Supplementary file 8 — Supplementary Data 6 [file 41467_2021_21381_MOESM8_ESM.pdf]
